# Supplementary material for: Photocascade chemoselective controlling of ambident thio(seleno)cyanates with alkenes via catalyst modulation
Source: Nat Commun. 2024 Jul 9;15:5739. doi: 10.1038/s41467-024-49279-w (PMC11233607; doi:10.1038/s41467-024-49279-w)
Supplement: Supplementary file 1 — Supplementary Information [file 41467_2024_49279_MOESM1_ESM.pdf]

# SUPPLEMENTARY INFORMATION

## Photocascade Chemoselective Controlling of Ambident Thio(Seleno)cyanates with Alkenes via Catalyst Modulation

Injamam Ul Hoque[1], Apurba Samanta[1], Shyamal Pramanik[1], Soumyadeep Roy Chowdhury[1], Rabindranath Lo[2] and Soumitra Maity[1] *

[1]Department of Chemistry and Chemical Biology, Indian Institute of Technology (ISM) Dhanbad, JH 826004, India

[2]Institute of Organic Chemistry and Biochemistry, Czech Academy of Sciences, v.v.i., Flemingovo nám. 2, 16000 Prague 6, Czech Republic.

Email: smaity@iitism.ac.in

| S. no | List of Contents | | Page |
|---|---|---|---|
| 1. | General Considerations | | S3 |
| 2. | General Procedure of Synthesis of *N*-Arylphenothiazines | | S4 |
| 3. | Preparation of Starting Materials | | S6 |
| | 3.1. | Preparation of Alkene Substrates | S6 |
| | 3.2. | Preparation of Alkylthiocyanate/Alkylselenocyanate Substrates | S13 |
| 4. | Reaction Optimization | | S20 |
| 5. | Reaction Generality | | S22 |
| | 5.1. | Synthetic Procedures | S22 |
| | 5.2. | Compound Characterization Data | S26 |
| 6. | Photocatalytic Three-Component Chemodivergent Reaction | | S60 |
| 7. | Synthetic Transformations of Photochemical Chemodivergent Products | | S61 |
| 8. | Mechanistic Studies | | S68 |
| | 8.1. | Radical Inhibition Experiments | S68 |
| | 8.2. | Radical Probe Experiments | S72 |
| | 8.3. | Deuterium Labeling Experiment | S74 |
| | 8.4. | Cation Trapping Experiments | S79 |
| | 8.5. | Electrochemical Measurements | S82 |
| | 8.6. | UV-Vis Experiments | S86 |
| | 8.7. | Photoluminescence Experiments | S87 |
| | 8.8. | Light ON/OFF Experiments | S89 |
| | 8.9. | Impact of Light Intensity on Reaction Yield | S90 |
| | 8.10. | UV-Visible Study for EDA Complex | S91 |
| | 8.11. | UV-Visible Study for Direct Excitation of Compounds | S92 |
| | 8.12. | Stern-Volmer Fluorescence Quenching Experiments | S93 |
| | 8.13. | Investigation into Catalyst Activation Modes | S95 |
| | 8.14. | Determination of the Reaction Quantum Yield | S97 |
| | 8.15. | Additional Reaction Mechanism | S101 |
| 9. | Incompatible Substrates | | S103 |
| 10. | X-ray Crystal Structures and Data | | S105 |
| 11. | Computational Details | | S108 |
| 12. | NMR spectra | | S111 |
| 13. | Supplementary References | | S291 |

## Supplementary Methods:

### 1. General Considerations:

All commercially available chemicals and reagents were used without further purification unless otherwise stated. Solvents for extraction or column chromatography were of technical quality. All water used was purified via a Merck Millipore reverse osmosis purification system prior to use. All reactions were performed in oven-dried glassware under a positive pressure of argon with freshly distilled anhydrous solvents.[1] Solvents were transferred *via* syringe and were introduced into the reaction vessels through a rubber septum. Solvents were removed under reduced pressure using Büchi Rotavapor apparatus. All reactions conducted at rt refer to the temperature range of 30-35 ºC.

**Thin-layer chromatography (TLC):** The progress of the reaction was monitored by thin-layer chromatography (TLC) using $SiO_2$-60 UV254 coated aluminium sheets (Merck, TLC Silica gel 60 $F_{254}$). Visualization was achieved using UV light, iodine, and/or chemical staining with vanillin or basic potassium permanganate solutions as appropriate.

**Flash column chromatography (FC):** Purification of the reaction mixture was carried out with flash column chromatography on silica gel 230-400 mesh (Merck, 37-63 µm). Solvents for extraction and chromatography were of technical quality. Eluting solvent mixtures are individually reported in parenthesis.

**NMR spectra:** Proton, Carbon, and Fluorine nuclear magnetic resonance ($^{1}H$, $^{13}C$, and $^{19}F$ NMR) spectra were recorded on a Bruker Avance III HD (400, 101, and 377 MHz) spectrometer at 25 °C. Chemical shifts (δ) are given in ppm and reported as follows: multiplicity (s (singlet), d (doublet), t (triplet), q (quartet), dd (doublet of doublets), dt (doublet of triplets), and m (multiplet), coupling constants (*J*) in Hz, number of protons; suggested assignment. The residual deuterated solvent was used as internal standard ($CDCl_3$: $\delta_H$ = 7.26 ppm; $\delta_C$ = 77.16 ppm; DMSO-$d_6$: $\delta_H$ = 2.50 ppm; $\delta_C$ = 39.52 ppm).

**Melting point (Mp):** Melting points were measured using open glass capillaries in 'Tempstar' (model: KMP-207A) and Remco-Kolkata apparatus and are reported uncorrected.

**High-resolution mass spectrometry (HRMS):** HRMS were recorded using Waters XEVO G2-XS QTOF and Agilent Technologies 6530 Accurate-Mass QTOF by ESI technique.

**Photoreactions:** Photoreactions were carried out in borosilicate made VWR®, Culture tube (16×125 mm) using the Kessil LEDs (PR160L-390 nm, max 52W, https://kessil.com/products/science_PR160L.php) placed at a distance of approximately 5 cm. The maximum light intensity is centered at 390 nm. PAR38 12W blue LED bulb was used for the 450 nm light. Unless otherwise noted, all reactions were conducted with 100% light intensity using Kessil light.

**Electrochemical Measurements:** Cyclic Voltammetry was performed using CH Instruments (model: CHI1140C).

**UV-Vis Spectrophotometer:** UV-Vis absorption spectra were recorded using Shimadzu UV Spectrophotometer (model: UV-1800).

**Luminescence spectrometer:** Fluorescence studies were carried out using Hitachi Fluorescence Spectrophotometer (Model: F-2700) and PerkinElmer LS 55 Fluorescence Spectrometer.

## 2. General Procedure of Synthesis of *N*-Arylphenothiazines:[2]

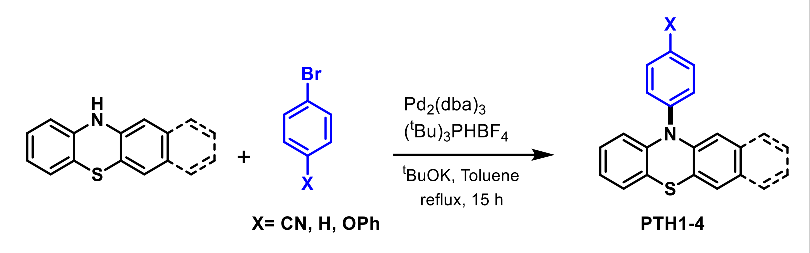


Phenothiazine (1 mmol) was dissolved in anhydrous toluene (3 mL). Arylbromide (1.2 mmol), potassium tert-butoxide (281 mg, 2.5 mmol), tri-*tert*-butylphosphonium tetrafluoroborate (17 mg, 0.06 mmol) was added, followed by tris(dibenzylideneacetone)dipalladium(0) (55 mg, 0.06 mmol). The reaction mixture was degassed using argon and refluxed for 15 h. After the completion of the reaction, 50 mL EtOAc and 25 mL water were added to the reaction mixture. After phase separation, the aqueous layer was extracted additionally with EtOAc (3×50 mL). The combined organic layers were washed with brine (50 mL), dried over $Na_2SO_4$, and concentrated under vacuo. Then, the crude was purified by silica gel column chromatography (230−400 mesh) using EtOAc/petroleum ether as eluent to afford the corresponding product **PTH1-4**.

**4-(12*H*-Benzo[*b*]phenothiazin-12-yl)benzonitrile (PTH1):**[3]

**Yield:** 58% (203 mg).

**Nature:** White solid.

**$R_f$ value** = 0.33 [EtOAc:Petroleum ether = 1:19 (v/v)].

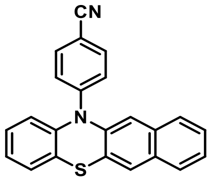


**PTH1**

**$^1$H NMR (400 MHz, $CDCl_3$) $\delta$ (ppm):** 7.81 (s, 1H), 7.72 – 7.66 (m, 2H), 7.61 (d, *J* = 8.8 Hz, 2H), 7.44 – 7.38 (m, 3H), 7.37 (s, 1H), 7.25 – 7.20 (m, 3H), 7.16 – 7.12 (m, 1H), 7.04 (dd, *J* = 8.0, 1.1 Hz, 1H).

**$^{13}$C{$^1$H} NMR (101 MHz, $CDCl_3$) $\delta$ (ppm):** 148.4, 141.5, 139.4, 134.1, 132.6, 131.4, 129.8, 129.6, 128.6, 127.5, 126.9, 126.8, 126.6, 126.3, 125.6, 123.8, 120.8, 120.7, 119.3, 106.3.

**4-(10*H*-Phenothiazin-10-yl)benzonitrile (PTH2):**

**Yield:** 93% (279 mg).

**Nature:** Yellow solid.

**Mp:** 153 – 155 °C.

**$R_f$ value** = 0.31 [EtOAc:Petroleum ether = 1:19 (v/v)].

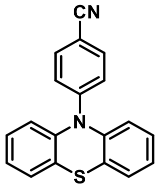


**PTH2**

**$^1$H NMR (400 MHz, DMSO-$d_6$) $\delta$ (ppm):** 7.72 (d, *J* = 9.0 Hz, 2H), 7.53 (dd, *J* = 7.7, 1.3 Hz, 2H), 7.40 – 7.36 (m, 2H), 7.31 (dd, *J* = 8.0, 1.3 Hz, 2H), 7.29 – 7.25 (m, 2H), 7.09 (d, *J* = 8.9 Hz, 2H).

**$^{13}$C{$^1$H} NMR (101 MHz, DMSO-$d_6$) $\delta$ (ppm):** 148.3, 140.6, 134.0, 131.2, 128.7, 127.9, 126.3, 125.5, 119.2, 117.5, 103.6.

**HRMS** (ESI) *m/z* calcd for $C_{19}H_{13}N_2S$ $[M+H]^+$: 301.0799; found: 301.0793.

## 10-Phenyl-10*H*-phenothiazine (PTH3):[2]

**Yield:** 89% (245 mg).

**Nature:** White solid.

**$R_f$ value** = 0.32 [EtOAc:Petroleum ether = 1:24 (v/v)].

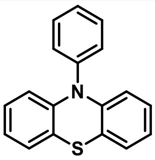

**PTH3**

**$^1$H NMR (400 MHz, DMSO-d$_6$) *δ* (ppm):** 7.69 – 7.65 (m, 2H), 7.56 – 7.52 (m, 1H), 7.42 (d, *J* = 7.3 Hz, 2H), 7.08 (dd, *J* = 7.5, 1.7 Hz, 2H), 6.93 (td, *J* = 7.9, 1.7 Hz, 2H), 6.86 (td, *J* = 7.5, 1.2 Hz, 2H), 6.16 (dd, *J* = 8.1, 1.2 Hz, 2H).

**$^{13}$C{$^1$H} NMR (101 MHz, DMSO-d$_6$) *δ* (ppm):** 143.6, 140.3, 131.1, 130.3, 128.5, 127.3, 126.7, 122.8, 119.4, 116.1.

## 10-(4-Phenoxyphenyl)-10*H*-phenothiazine (PTH4):

**Yield:** 92% (338 mg).

**Nature:** White solid.

**Mp:** 161 – 163 °C.

**$R_f$ value** = 0.30 [EtOAc:Petroleum ether = 1:24 (v/v)].

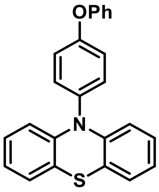


**PTH4**

**$^1$H NMR (400 MHz, DMSO-d$_6$) *δ* (ppm):** 7.49 – 7.45 (m, 2H), 7.42 (d, *J* = 8.7 Hz, 2H), 7.24 – 7.17 (m, 5H), 7.06 (dd, *J* = 7.4, 1.0 Hz, 2H), 6.97 – 6.92 (m, 2H), 6.85 (t, *J* = 7.4 Hz, 2H), 6.20 (d, *J* = 8.1 Hz, 2H).

**$^{13}$C{$^1$H} NMR (101 MHz, DMSO-d$_6$) *δ* (ppm):** 156.8, 155.9, 143.8, 134.9, 132.4, 130.3, 127.4, 126.6, 124.3, 122.7, 120.2, 119.5, 119.0, 115.8.

**HRMS** (ESI) *m/z* calcd for $C_{24}H_{17}NOS$ $[M]^+$: 367.1031; found: 367.1034.

## 3. Preparation of Starting Materials:

### 3.1. Preparation of Alkene Substrates:

Most of the alkenes were purchased from commercial sources and used as received. Some of the alkenes are prepared and characterized accordingly.

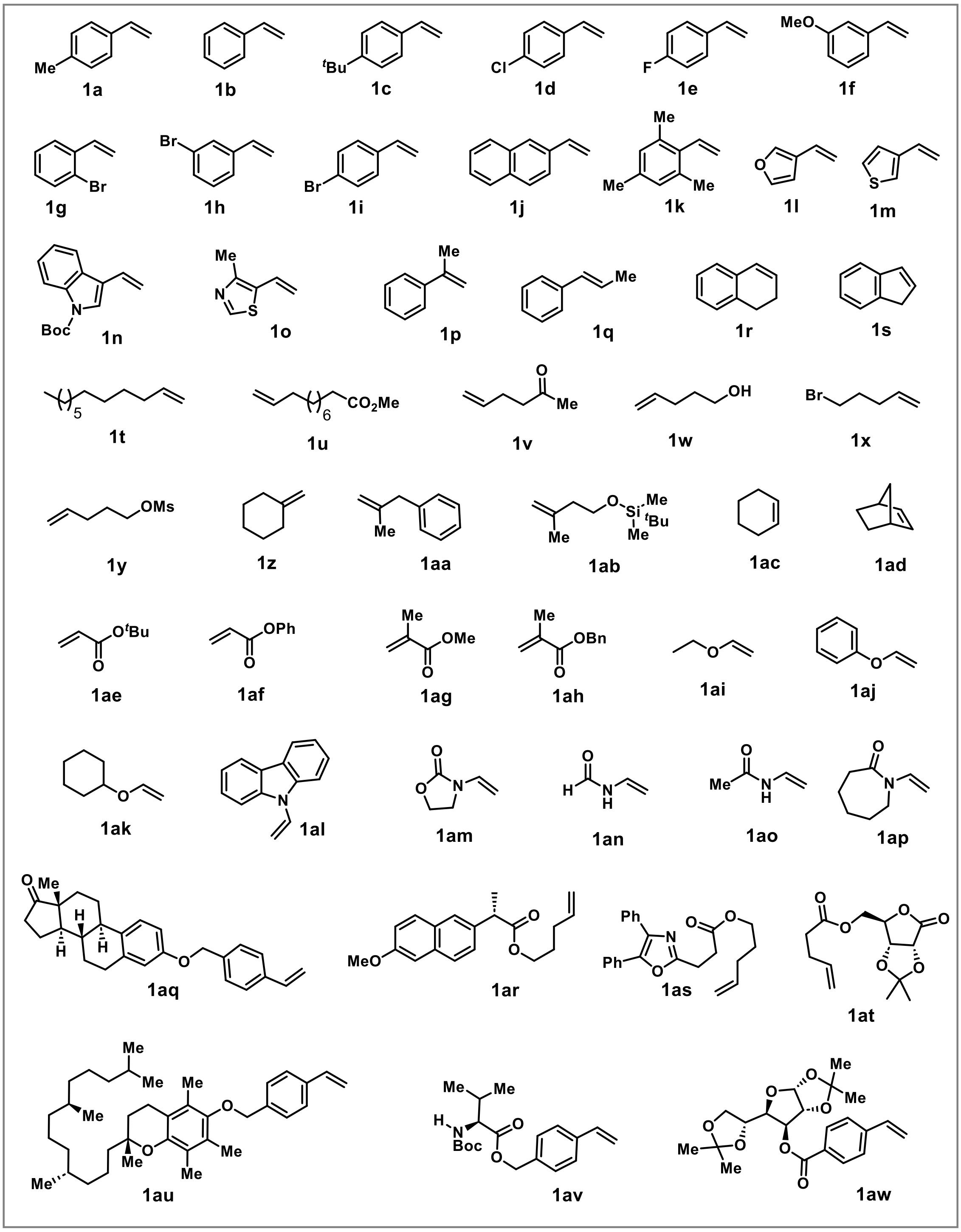


**Supplementary Fig. 1:** Alkenes included in the manuscript.

**Methyl undec-10-enoate (1u):**[4]

A solution of 10-undecenoic acid (1.84 g, 10 mmol) in 2% $H_2SO_4$/MeOH (20 mL) was refluxed for 4 h. After the completion of the reaction (monitored by TLC), the reaction mixture was cooled to rt and concentrated under vacuo. Then the reaction crude was diluted with ethyl acetate (20 mL) and saturated aqueous $NaHCO_3$ solution (10 mL). The organic layer was separated, and the aqueous layer was extracted with ethyl acetate (2×15 mL). The combined organic layers were washed with brine (30 mL) and dried over $Na_2SO_4$. The solution was concentrated and purified via silica gel column chromatography (230−400 mesh) using EtOAc/petroleum ether as eluent to afford the corresponding product **1u**.

**Methyl undec-10-enoate (1u):**

**Yield:** 91% (1.8 g).

**Nature:** Colourless oil.

**$R_f$ value** = 0.41 [EtOAc:Petroleum ether = 1:19 (v/v)].

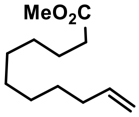


**1u**

**$^1$H NMR (400 MHz, $CDCl_3$) $\delta$ (ppm):** 5.84 – 5.73 (m, 1H), 4.99 – 4.89 (m, 2H), 3.65 (s, 3H), 2.28 (t, $J$ = 7.6 Hz, 2H), 2.01 (q, $J$ = 6.9 Hz, 2H), 1.61 – 1.56 (m, 2H), 1.38 – 1.32 (m, 2H), 1.31 – 1.23 (m, 8H).

**$^{13}$C{$^1$H} NMR (101 MHz, $CDCl_3$) $\delta$ (ppm):** 174.4, 139.3, 114.3, 51.6, 34.2, 33.9, 29.4, 29.3, 29.2, 29.1, 29.0, 25.0.

**Pent-4-en-1-yl methanesulfonate (1y):**[5]

A 100 mL RB flask fitted with a stir bar was charged with a solution of 4-penten-1-ol (1.03 mL, 10 mmol), triethylamine (1.53 mL, 11 mmol) in 40 mL dichloromethane. The mixture was cooled to 0 °C, and methanesulfonyl chloride (0.85 mL, 11 mmol) was added dropwise. The mixture was stirred for 2 h and quenched with 1(N) HCl (4 mL), and the solution was washed with saturated aqueous $NaHCO_3$ solution (2×30 mL) and brine (1×20 mL). The organic layer was dried over $Na_2SO_4$ and concentrated under vacuo. Then, the crude mass was purified via silica gel column chromatography (230−400 mesh) using EtOAc/petroleum ether as eluent to afford the corresponding product **1y**.

**Pent-4-en-1-yl methanesulfonate (1y):**

**Yield:** 88% (1.44 g).

**Nature:** Colourless oil.

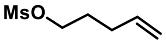


**1y**

**$R_f$ value** = 0.40 [EtOAc:Petroleum ether = 1:19 (v/v)].

**$^1$H NMR (400 MHz, $CDCl_3$) $\delta$ (ppm):** 5.81 – 5.71 (m, 1H), 5.07 – 4.99 (m, 2H), 4.21 (t, $J$ = 6.5 Hz, 2H), 2.98 (s, 3H), 2.16 (q, $J$ = 7.3 Hz, 2H), 1.83 (p, $J$ = 6.9 Hz, 2H).

**$^{13}$C{$^1$H} NMR (101 MHz, $CDCl_3$) $\delta$ (ppm):** 136.7, 116.1, 69.4, 37.3, 29.5, 28.2.

***tert*-Butyldimethyl((3-methylbut-3-en-1-yl)oxy)silane (1ab):**[6]

To a solution of 3-methylbut-3-en-1-ol (1 mL, 10 mmol) and imidazole (1.4 g, 20 mmol) in dry $CH_2Cl_2$ (20 mL) was added TBDMSCl (1.9 g, 12.5 mmol) and the resulting mixture was stirred at rt for 18 h. After the completion of the reaction, the reaction crude was diluted with $CH_2Cl_2$ (10 mL), and $H_2O$ (10

mL) was added. The mixture was extracted with $CH_2Cl_2$ (2×20 mL), and the combined organic layers were washed with brine and dried over $Na_2SO_4$. The solution was concentrated and purified via silica gel column chromatography (230−400 mesh) using EtOAc/petroleum ether as eluent to afford the corresponding product **1ab**.

***tert*-Butyldimethyl((3-methylbut-3-en-1-yl)oxy)silane (1ab):**

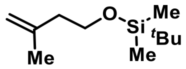

1ab

**Yield:** 90% (1.8 g).

**Nature:** Colourless oil.

**$R_f$ value** = 0.39 [EtOAc:Petroleum ether = 1:19 (v/v)].

**$^1$H NMR (400 MHz, $CDCl_3$) *δ* (ppm):** 4.75 – 4.69 (m, 2H), 3.71 (t, *J* = 7.1 Hz, 2H), 2.24 (t, *J* = 7.0 Hz, 2H), 1.74 (s, 3H), 0.89 (s, 9H), 0.05 (s, 6H).

**$^{13}$C{$^1$H} NMR (101 MHz, $CDCl_3$) *δ* (ppm):** 143.2, 111.6, 62.3, 41.3, 26.1, 23.1, 18.5, -5.2.

**3-Vinyloxazolidin-2-one (1am):**[7]

In a 25 mL round-bottomed flask equipped with magnetic stir-bar, bathophenanthroline (166 mg, 0.5 mmol), palladium(II) trifluoroacetate (166 mg, 0.5 mmol), and oxazolidinone (871 mg, 10 mmol) were dissolved in ethyl vinyl ether (10 mL). The flask was closed with a septum and then opened to the atmosphere by means of a needle. The yellow suspension was then stirred at 75 °C for 2 h. After completion of the reaction, the crude was concentrated and purified via silica gel column chromatography (230−400 mesh) using EtOAc/petroleum ether as eluent to afford the corresponding product **1am**.

**3-Vinyloxazolidin-2-one (1am):**

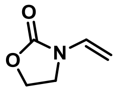

1am

**Yield:** 91% (1.03 g).

**Nature:** Colourless oil

**$R_f$ value** = 0.32 [EtOAc:Petroleum ether = 3:7 (v/v)].

**$^1$H NMR (400 MHz, $CDCl_3$) *δ* (ppm):** 6.87 (dd, *J* = 15.8, 8.9 Hz, 1H), 4.47 – 4.42 (m, 3H), 4.29 (dd, *J* = 15.8, 1.2 Hz, 1H), 3.71 (dd, *J* = 8.9, 7.3 Hz, 2H).

**$^{13}$C{$^1$H} NMR (101 MHz, $CDCl_3$) *δ* (ppm):** 155.5, 130.0, 93.6, 62.3, 42.0.

**(8*S*,9*R*,13*R*,14*R*)-13-Methyl-3-((4-vinylbenzyl)oxy)-6,7,8,9,11,12,13,14,15,16-decahydro-17*H*-cyclopenta[*a*]phenanthren-17-one (1aq):**[8]

In 10 mL of dry DMF, estrone (540 mg, 2.0 mmol), 4-vinylbenzyl chloride (0.34 mL, 2.4 mmol), and $K_2CO_3$ (414 mg, 3.0 mmol) were taken under argon atmosphere. Then, the resultant mixture was allowed to stirring for 24 h at rt. After completion of the reaction (as monitored by TLC), 20 mL of $Et_2O$ and 20 mL of distilled water were added to that. The reaction mixture was transferred to a separatory funnel, and the organic layer was extracted with $Et_2O$ (3×15 mL). The combined organic layers were washed with brine solution (10 mL), dried over $Na_2SO_4$, and concentrated under reduced pressure. Purification of the crude mixture with silica gel chromatography (EtOAc/petroleum ether) provided the title compound **1aq**.

**(8*S*,9*R*,13*R*,14*R*)-13-Methyl-3-((4-vinylbenzyl)oxy)-6,7,8,9,11,12,13,14,15,16-decahydro-17*H*-cyclopenta[*a*]phenanthren-17-one (1aq):**

**Yield:** 64% (495 mg).

**Nature:** White solid.

**$R_f$ value**= 0.48 [EtOAc/petroleum ether = 1:9 (v/v)]

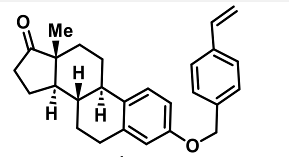


1aq

**$^1$H NMR (400 MHz, $CDCl_3$) *δ* (ppm):** 7.43 (d, *J* = 8.3 Hz, 2H), 7.38 (d, *J* = 8.3 Hz, 2H), 7.21 (d, *J* = 8.5 Hz, 1H), 6.79 (dd, *J* = 8.6, 2.8 Hz, 1H), 6.76 – 6.69 (m, 2H), 5.76 (dd, *J* = 17.6, 0.7 Hz, 1H), 5.26 (dd, *J* = 10.9, 0.7 Hz, 1H), 5.03 (s, 2H), 2.92 – 2.88 (m, 2H), 2.51 (dd, *J* = 18.8, 8.5 Hz, 1H), 2.42 – 2.38 (m, 1H), 2.28 – 2.23 (m, 1H), 2.19 – 2.10 (m, 1H), 2.09 – 1.94 (m, 3H), 1.68 – 1.53 (m, 3H), 1.52 – 1.38 (m, 3H), 0.91 (s, 3H).

**$^{13}$C{$^1$H} NMR (101 MHz, $CDCl_3$) *δ* (ppm):** 221.2, 156.9, 137.9, 137.4, 136.9, 136.6, 132.5, 127.8, 126.5, 115.0, 114.2, 112.5, 69.8, 50.5, 48.1, 44.1, 38.5, 36.0, 31.7, 29.8, 26.7, 26.0, 21.7, 14.0.

***(R)*-2,5,7,8-Tetramethyl-2-((4*R*,8*R*)-4,8,12-trimethyltridecyl)-6-((4-vinylbenzyl)oxy)chromane (1au):**

In 25 mL of dry DMF, DL-α-tocopherol (2.27 g, 5.0 mmol), 4-vinylbenzyl chloride (0.85 mL, 6.0 mmol), and $K_2CO_3$ (1.03 g, 7.5 mmol) were taken under argon atmosphere. Then, the resultant mixture was allowed to stirring for 24 h at rt. After completion of the reaction (as monitored by TLC), 50 mL of $Et_2O$ and 40 mL of distilled water were added to that. The reaction mixture was transferred to a separatory funnel, and the organic layer was extracted with $Et_2O$ (3×30 mL). The combined organic layers were washed with brine solution (30 mL), dried over $Na_2SO_4$, and concentrated under reduced pressure. Purification of the crude mixture with silica gel chromatography (EtOAc/petroleum ether) provided the title compound **1au**.

**(*R*)-2,5,7,8-Tetramethyl-2-((4*R*,8*R*)-4,8,12-trimethyltridecyl)-6-((4-vinylbenzyl)oxy)chromane (1au):**

**Yield:** 67% (1.83 g).

**Nature:** Viscous liquid.

**$R_f$ value** = 0.33 [EtOAc:Petroleum ether = 1:4 (v/v)].

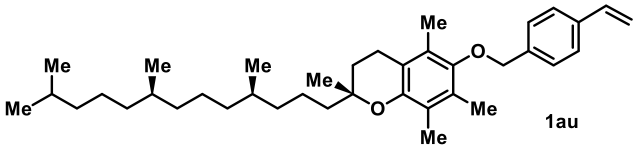


1au

**$^1$H NMR (400 MHz, $CDCl_3$) *δ* (ppm):** 7.51 (d, *J* = 8.5 Hz, 2H), 7.48 (d, *J* = 8.6 Hz, 2H), 6.79 (dd, *J* = 17.6, 10.9 Hz, 1H), 5.82 (d, *J* = 17.6 Hz, 1H), 5.30 (dd, *J* = 10.8, 0.6 Hz, 1H), 4.73 (s, 2H), 2.64 (t, *J* = 6.7 Hz, 2H), 2.27 (s, 3H), 2.22 (s, 3H), 2.16 (s, 3H), 1.92 – 1.78 (m, 2H), 1.66 – 1.53 (m, 4H), 1.50 – 1.39 (m, 4H), 1.34 – 1.30 (m, 9H), 1.22 – 1.11 (m, 7H), 0.93 – 0.90 (m, 12H).

**$^{13}$C{$^1$H} NMR (101 MHz, $CDCl_3$) *δ* (ppm):** 148.2, 148.0, 137.8, 137.2, 136.7, 128.0, 126.4, 126.1, 123.1, 117.7, 114.0, 74.9, 74.6, 40.1, 39.5, 37.7, 37.6, 37.5, 37.4, 32.9, 32.8, 31.4, 28.1, 25.0, 24.6, 24.0, 22.9, 22.8, 21.2, 20.8, 19.9, 19.8, 19.7, 13.0, 12.2, 12.0.

**HRMS** (ESI) *m/z* calcd for $C_{38}H_{59}O_2$ [M+H]$^+$: 547.4515; found: 547.4517.

**4-Vinylbenzyl (*tert*-butoxycarbonyl)-*L*-valinate (1av):**[9]

To a solution of Boc-*L*-valine (869 mg, 4.0 mmol) in 30 mL wet DMF (containing 1% water, 0.3 mL), 4-vinylbenzyl chloride (0.68 mL, 4.8 mmol) and $Na_2CO_3$ (508 mg, 4.8 mmol) were successively added. Then, the reaction mixture was stirred at 60 °C for 6 h. After completion of the reaction (as monitored by TLC) 30 mL of distilled water was added to that. The aqueous layer was extracted with ethyl acetate (3×30 mL), dried with dried $Na_2SO_4$, and concentrated under reduced pressure. Purification of the crude mixture with silica gel chromatography (EtOAc/petroleum ether) provided the title compound.

**4-Vinylbenzyl (*tert*-butoxycarbonyl)-*L*-valinate (1av):**

**Yield:** 61% (814 mg).

**Nature:** Colourless gel.

**$R_f$ value**= 0.41 [EtOAc/petroleum ether = 1:4 (v/v)]

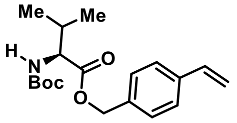


1av

**$^1$H NMR (400 MHz, $CDCl_3$) $\delta$ (ppm):** 7.40 (d, *J* = 8.2 Hz, 2H), 7.31 (d, *J* = 8.1 Hz, 2H), 6.71 (dd, *J* = 17.6, 10.9 Hz, 1H), 5.76 (dd, *J* = 17.6, 0.6 Hz, 1H), 5.27 (d, *J* = 11.0 Hz, 1H), 5.19 (d, *J* = 12.3 Hz, 1H), 5.10 (d, *J* = 12.3 Hz, 1H), 5.03 (d, *J* = 9.1 Hz, 1H), 4.27 (dd, *J* = 9.2, 4.6 Hz, 1H), 2.18 – 2.10 (m, 1H), 1.44 (s, 9H), 0.93 (d, *J* = 6.9 Hz, 3H), 0.84 (d, *J* = 6.9 Hz, 3H).

**$^{13}$C{$^1$H} NMR (101 MHz, $CDCl_3$) $\delta$ (ppm):** 172.5, 155.8, 137.8, 136.4, 135.0, 128.8, 126.5, 114.6, 79.9, 66.8, 58.6, 53.6, 31.4, 28.4, 19.2, 17.6.

**Synthesis of alkene substrates 1ar-1at, and 1aw:**

**General procedure for alkene esters synthesis (GP-DCC): DCC coupling of acids with alcohols**

To the ice-cooled solution of carboxylic acids (1.2 equiv.) in dry DCM (45 mL for 10 mmol scale), catalytic amount of DMAP (0.2 equiv.) and respective alcohols (1.0 equiv.) were added. Then, the mixture was allowed to stirred for 10-15 minutes to get a homogenous reaction mixture, and DCC (2.0 equiv.) was added at once under the same condition. Then cooling conditions were removed off, and the reaction mixture was allowed to stirring for 6-8 h at rt. Conversion into esters was checked with TLC and filtered through a short pad of celite to remove the solid particles. Then, the filtrate was concentrated under *vacuo* and purified by flash column chromatography on silica gel (EtOAc/ petroleum ether) to afford the corresponding alkenes.

**Pent-4-en-1-yl (*S*)-2-(6-methoxynaphthalen-2-yl)propanoate (1ar):** Following GP-DCC, the reaction between naproxen (552 mg, 2.4 mmol) and 4-penten-1-ol (0.207 mL, 2.0 mmol) provided the above title compound.

**Pent-4-en-1-yl (*S*)-2-(6-methoxynaphthalen-2-yl)propanoate (1ar):**

**Yield:** 70% (418 mg).

**Nature:** Colourless oil.

**$R_f$ value**= 0.40 [EtOAc/petroleum ether=1:9 (v/v)]

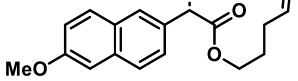


1ar

$^1$H NMR (400 MHz, CDCl$_3$) $\delta$ (ppm):** 7.71 (d, *J* = 8.5 Hz, 2H), 7.67 (d, *J* = 1.2 Hz, 1H), 7.41 (dd, *J* = 8.4, 1.8 Hz, 1H), 7.16 – 7.11 (m, 2H), 5.77 – 5.67 (m, 1H), 4.94 – 4.89 (m, 2H), 4.10 – 4.06 (m, 2H), 3.92 (s, 3H), 3.85 (q, *J* = 7.1 Hz, 1H), 2.04 – 1.98 (m, 2H), 1.71 – 1.64 (m, 2H), 1.58 (d, *J* = 7.1 Hz, 3H).

**$^{13}$C{$^1$H} NMR (101 MHz, CDCl$_3$) $\delta$ (ppm):** 174.8, 157.7, 137.5, 135.9, 133.8, 129.4, 129.0, 127.2, 126.4, 126.0, 119.1, 115.4, 105.6, 64.3, 55.4, 45.6, 30.1, 27.8, 18.6.

**HRMS** (ESI) *m/z* calcd for $C_{19}H_{22}O_3Na$ [M+Na]$^+$: 321.1467; found: 321.1453.

**Pent-4-en-1-yl 3-(4,5-diphenyloxazol-2-yl)propanoate (1as):** Following GP-DCC, the reaction between oxaprozin (1.76 g, 6.0 mmol) and 4-penten-1-ol (0.52 mL, 5.0 mmol) provided the above title compound.

**Pent-4-en-1-yl 3-(4,5-diphenyloxazol-2-yl)propanoate (1as):**

**Yield:** 45% (813 mg).

**Nature:** Colourless oil.

**R$_f$ value**= 0.31 [EtOAc/petroleum ether=3:7 (v/v)]

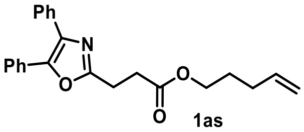


**1as**

**$^1$H NMR (400 MHz, CDCl$_3$) $\delta$ (ppm):** 7.64 – 7.62 (m, 2H), 7.58 – 7.56 (m, 2H), 7.39 – 7.30 (m, 6H), 5.83 – 5.72 (m, 1H), 5.04 – 4.95 (m, 2H), 4.14 (t, *J* = 6.6 Hz, 2H), 3.19 (t, *J* = 7.5 Hz, 2H), 2.92 (t, *J* = 7.5 Hz, 2H), 2.14 – 2.09 (m, 2H), 1.77 – 1.70 (m, 2H).

**$^{13}$C{$^1$H} NMR (101 MHz, CDCl$_3$) $\delta$ (ppm):** 172.2, 161.9, 145.5, 137.5, 135.2, 132.5, 129.1, 128.8, 128.7, 128.6, 128.2, 128.0, 126.6, 115.5, 64.4, 31.3, 30.1, 27.9, 23.7.

**HRMS** (ESI) *m/z* calcd for $C_{23}H_{24}NO_3$ [M+H]$^+$: 362.1756; found: 362.1752.

**((3a*R*,4*R*,6a*R*)-2,2-Dimethyl-6-oxotetrahydrofuro[3,4-*d*][1,3]dioxol-4-yl)methyl pent-4-enoate (1at):** Following GP-DCC, the reaction between 2,3-*O*-isopropylidene-D-ribono-1,4-lactone (1.88 g, 10 mmol) and 4-pentenoic acid (1.22 mL, 12 mmol) provided the above title compound.

**((3a*R*,4*R*,6a*R*)-2,2-Dimethyl-6-oxotetrahydrofuro[3,4-*d*][1,3]dioxol-4-yl)methyl pent-4-enoate (1at):**

**Yield:** 74% (2.0 g).

**Nature:** Viscous liquid.

**R$_f$ value**= 0.38 [EtOAc/petroleum ether=1:4 (v/v)]

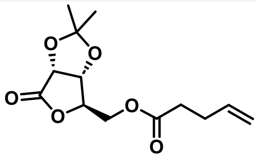


**1at**

**$^1$H NMR (400 MHz, CDCl$_3$) $\delta$ (ppm):** 5.83 – 5.74 (m, 1H), 5.08 – 5.01 (m, 2H), 4.78 – 4.74 (m, 2H), 4.68 (d, *J* = 5.7 Hz, 1H), 4.38 (dd, *J* = 12.4, 2.8 Hz, 1H), 4.25 (dd, *J* = 12.4, 2.4 Hz, 1H), 2.45 – 2.41 (m, 2H), 2.38 – 2.34 (m, 2H), 1.49 (s, 3H), 1.39 (s, 3H).

**$^{13}$C{$^1$H} NMR (101 MHz, CDCl$_3$) $\delta$ (ppm):** 173.6, 172.0, 136.1, 116.3, 114.0, 79.7, 77.9, 75.3, 63.6, 33.3, 28.7, 26.9, 25.7.

**HRMS** (ESI) *m/z* calcd for $C_{13}H_{18}O_6Na$ [M+Na]$^+$: 293.1001; found: 293.0995.

**(3a*R*,5*R*,6*S*,6a*R*)-5-((*R*)-2,2-Dimethyl-1,3-dioxolan-4-yl)-2,2-dimethyltetrahydrofuro[2,3-*d*][1,3]dioxol-6-yl 4-vinylbenzoate (1aw):** Following GP-DCC, the reaction between 1,2:5,6-Di-O-isopropylidene-*α*-D-glucofuranose (2.6 g, 10 mmol) and 4-Vinylbenzoic acid (1.78 g, 12 mmol) provided the above title compound.

**(3a*R*,5*R*,6*S*,6a*R*)-5-((*R*)-2,2-Dimethyl-1,3-dioxolan-4-yl)-2,2-dimethyltetrahydrofuro[2,3-*d*][1,3]dioxol-6-yl 4-vinylbenzoate (1aw):**[10]

**Yield:** 82% (3.2 g).

**Nature:** Viscous liquid.

**$R_f$ value**= 0.32 [EtOAc/petroleum ether=1:4 (v/v)]

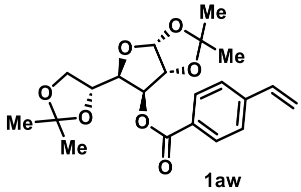


1aw

**$^1$H NMR (400 MHz, CDCl$_3$) *δ* (ppm):** 7.97 (d, *J* = 8.4 Hz, 2H), 7.46 (d, *J* = 8.3 Hz, 2H), 6.74 (dd, *J* = 17.6, 10.9 Hz, 1H), 5.95 (d, *J* = 3.7 Hz, 1H), 5.87 (d, *J* = 17.6 Hz, 1H), 5.49 (d, *J* = 2.7 Hz, 1H), 5.40 (d, *J* = 10.9 Hz, 1H), 4.63 (d, *J* = 3.7 Hz, 1H), 4.38 – 4.31 (m, 2H), 4.13 – 4.06 (m, 2H), 1.55 (s, 3H), 1.41 (s, 3H), 1.31 (s, 3H), 1.26 (s, 3H).

**$^{13}$C{$^1$H} NMR (101 MHz, CDCl$_3$) *δ* (ppm):** 165.1, 142.6, 135.9, 130.1, 128.6, 126.3, 117.1, 112.4, 109.5, 105.2, 83.5, 80.0, 76.7, 72.7, 67.3, 26.9, 26.8, 26.3, 25.3.

### 3.2. Preparation of Alkylthiocyanate/Alkylselenocyanate Substrates:

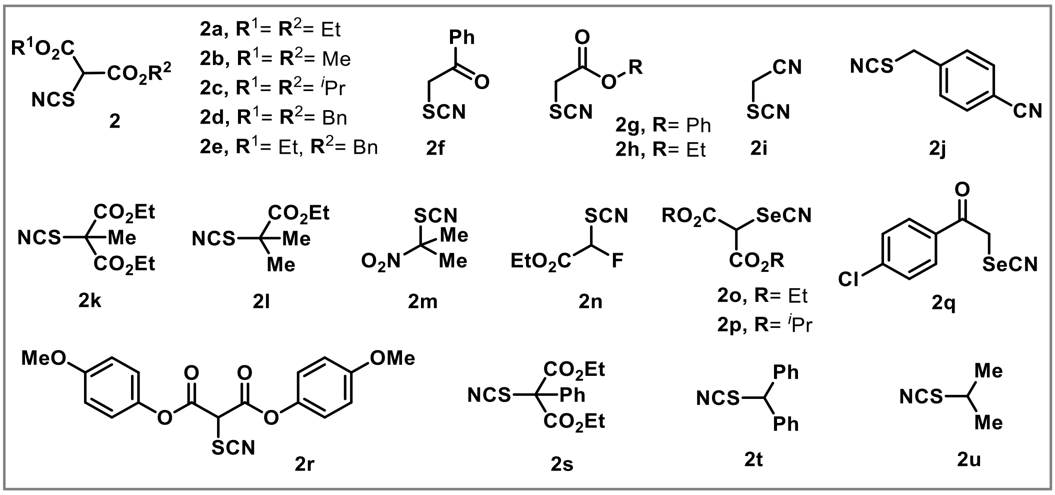


**Supplementary Fig. 2:** Alkylthiocyanate/Alkylselenocyanate included in the manuscript.

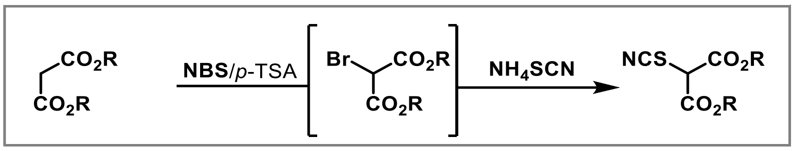


Thiocyanatomalonates were usually prepared from the corresponding malonates by one-pot process involving bromination, followed by thiocyanation.

**General Procedure A (GP-A):**[11,12] To a solution of the malonate compound (10.0 mmol) in acetonitrile (75 mL), *N*-bromosuccinimide (2.14 g, 12.0 mmol), and *p*-toluensulphonic acid (344 mg, 2.0 mmol) were added, and the mixture was refluxed for 4 h. Then, the reaction mixture was cooled to rt. Thereafter, KSCN (1.46 g, 15 mmol) was added, and the mixture was stirred at rt for 1 h. After the completion of the reaction (confirmed by TLC), the reaction mixture was quenched with water (40 mL) and extracted with EtOAc (3×60 mL). The combined organic layers were washed with brine (50 mL), dried over $Na_2SO_4$, and concentrated under vacuo. The residue was purified by silica gel column chromatography using EtOAc/petroleum ether as eluent to afford the corresponding thiocyanomalonate product.

**Supplementary Notes:** *The inorganic thiocyanate salt (Na/K/$NH_4SCN$) is hygroscopic, and care should be taken to ensure the reagent is anhydrous prior to use.*

**General Procedure B (GP-B):**[13] DBU (1.5 mL, 10.0 mmol) was slowly added to the malonate compound (10.0 mmol) in dry THF (75 mL) at 0 °C. The reaction mixture was stirred at this temperature for 30 min and was then cooled to -78 °C. $CBr_4$ (3.3 g, 10.0 mmol) was then added in one portion. The reaction mixture was stirred at -78 °C for 6 h and was quenched by the slow addition of an aqueous saturated solution of $NH_4Cl$ (30 mL) and water (10 mL). The mixture was warmed to rt, and the organic layers were separated and washed with brine (30 mL). The combined aqueous layers were extracted with dichloromethane (2×50 mL). The combined organic layers were dried over $Na_2SO_4$ and concentrated in

vacuo. The residue was purified by silica gel column chromatography using EtOAc/petroleum ether as eluent to afford the corresponding bromo product.

To a solution of the previously prepared bromo-malonate compound (1 equiv.) in acetonitrile (0.1 M), KSCN (1.5 equiv.) was added, and the mixture was stirred at rt for 1 h. After the reaction was completed (monitored by TLC), the reaction mixture was quenched with water (30 mL) and extracted with EtOAc (3×50 mL). The combined organic layers were washed with brine (50 mL), dried over $Na_2SO_4$, and concentrated under vacuo. The residue was purified by silica gel column chromatography using EtOAc/petroleum ether as eluent to afford the corresponding thiocyanomalonate product.

**General Procedure C (GP-C):** To a solution of the alkyl-bromide compound (5 mmol) in 50 mL acetonitrile, KSCN (729 mg, 7.5 mmol)/KSeCN (1.08 g, 7.5 mmol) was added and the mixture was stirred at rt for 3 h. After the reaction was completed (monitored by TLC), the reaction mixture was quenched with water (30 mL) and extracted with EtOAc (3×50 mL). The combined organic layers were washed with brine (50 mL), dried over $Na_2SO_4$, and concentrated under vacuo. The residue was purified by silica gel column chromatography using EtOAc/petroleum ether as eluent to afford the corresponding alkylthiocyanate/alkylselenocyanate product.

**General Procedure D (GP-D):**[14] 2-Substituted-malonate (5.0 mmol) and NaSCN (810 mg, 10.0 mmol) were mixed with EtOAc (25 mL), and then CAN (5.48 g, 10.0 mmol) was added with stirring. The reaction mixture was stirred for 3 h at rt. After the reaction was completed (monitored by TLC), EtOAc (25 mL) was added, and the mixture was poured into water (30 mL) and extracted with EtOAc (3×40 mL). Combined organic layers were washed with brine (50 mL), dried over $NaSO_4$, and concentrated under vacuo. The residue was purified by silica gel column chromatography using EtOAc/petroleum ether as eluent to afford the corresponding thiocyanate product.

**Diethyl 2-thiocyanatomalonate (2a):**[12]

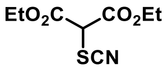

2a

Prepared according to **GP-A**.

**Yield:** 71% (1.54 g).

**Nature:** Colourless oil.

**$R_f$ value** = 0.34 [EtOAc:Petroleum ether = 1:9 (v/v)].

**$^1$H NMR (400 MHz, $CDCl_3$) *δ* (ppm):** 4.69 (s, 1H), 4.30 (q, *J* = 7.1 Hz, 4H), 1.31 (t, *J* = 7.2 Hz, 6H).

**$^{13}$C{$^1$H} NMR (101 MHz, $CDCl_3$) *δ* (ppm):** 163.8, 109.2, 63.9, 52.2, 13.9.

**Dimethyl 2-thiocyanatomalonate (2b):**

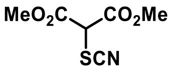

2b

Prepared according to **GP-A**.

**Yield:** 73% (1.38 g).

**Nature:** Colourless oil.

**$R_f$ value** = 0.32 [EtOAc:Petroleum ether = 1:9 (v/v)].

**$^1$H NMR (400 MHz, $CDCl_3$) *δ* (ppm):** 4.74 (s, 1H), 3.86 (s, 6H).

**$^{13}$C{$^1$H} NMR (101 MHz, $CDCl_3$) *δ* (ppm):** 164.2, 109.1, 54.4, 51.9.

**HRMS** (ESI) *m/z* calcd for $C_6H_7NO_4SNa$ $[M+Na]^+$: 211.9993; found: 211.9981.

**Diisopropyl 2-thiocyanatomalonate (2c):**

Prepared according to **GP-A**.

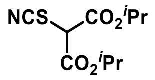

2c

**Yield:** 66% (1.62 g).

**Nature:** Colourless oil.

$R_f$ **value** = 0.42 [EtOAc:Petroleum ether = 1:9 (v/v)].

**$^1$H NMR (400 MHz, $CDCl_3$) $\delta$ (ppm):** 5.16 – 5.09 (m, 2H), 4.62 (s, 1H), 1.31 (d, $J$ = 3.0 Hz, 6H), 1.29 (d, $J$ = 3.0 Hz, 6H).

**$^{13}$C{$^1$H} NMR (101 MHz, $CDCl_3$) $\delta$ (ppm):** 163.4, 109.3, 72.2, 52.7, 21.6, 21.5.

**HRMS** (ESI) *m/z* calcd for $C_{10}H_{15}NO_4SNa$ $[M+Na]^+$: 268.0619; found: 268.0605.

**Dibenzyl 2-thiocyanatomalonate (2d):**

Prepared according to **GP-B**.

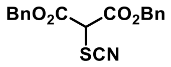

2d

**Yield:** 45% (1.54 g).

**Nature:** Colourless oil.

$R_f$ **value** = 0.33 [EtOAc:Petroleum ether = 1:9 (v/v)].

**$^1$H NMR (400 MHz, $CDCl_3$) $\delta$ (ppm):** 7.38 – 7.36 (m, 6H), 7.35 – 7.31 (m, 4H), 5.26 (s, 4H), 4.78 (s, 1H).

**$^{13}$C{$^1$H} NMR (101 MHz, $CDCl_3$) $\delta$ (ppm):** 163.5, 134.0, 129.0, 128.8, 128.6, 109.0, 69.4, 52.3.

**HRMS** (ESI) *m/z* calcd for $C_{18}H_{15}NO_4SNa$ $[M+Na]^+$: 364.0619; found: 364.0630.

**1-Benzyl 3-ethyl 2-thiocyanatomalonate (2e):**

Prepared according to **GP-B**.

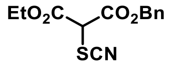

2e

**Yield:** 47% (1.31 g).

**Nature:** Colourless oil.

$R_f$ **value** = 0.32 [EtOAc:Petroleum ether = 1:9 (v/v)].

**$^1$H NMR (400 MHz, $CDCl_3$) $\delta$ (ppm):** 7.38 – 7.36 (m, 5H), 5.29 (d, $J$ = 1.0 Hz, 2H), 4.74 (s, 1H), 4.28 (q, $J$ = 7.1 Hz, 2H), 1.26 (t, $J$ = 7.1 Hz, 3H).

**$^{13}$C{$^1$H} NMR (101 MHz, $CDCl_3$) $\delta$ (ppm):** 163.7, 163.6, 134.1, 129.0, 128.8, 128.7, 109.1, 69.3, 64.0, 52.3, 13.9.

**HRMS** (ESI) *m/z* calcd for $C_{13}H_{17}N_2O_4S$ $[M+NH_4]^+$: 297.0909; found: 297.0897.

**1-Phenyl-2-thiocyanatoethan-1-one (2f):**[15]

Prepared according to **GP-C**.

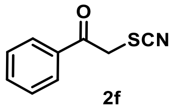

2f

**Yield:** 87% (771 mg).

**Nature:** White solid.

$R_f$ **value** = 0.39 [EtOAc:Petroleum ether = 1:19 (v/v)].

**$^1$H NMR (400 MHz, $CDCl_3$) $\delta$ (ppm):** 7.93 (dd, $J$ = 8.4, 1.2 Hz, 2H), 7.69 – 7.64 (m, 1H), 7.54 – 7.50 (m, 2H), 4.74 (s, 2H).

**$^{13}$C{$^1$H} NMR (101 MHz, $CDCl_3$) $\delta$ (ppm):** 191.0, 134.9, 134.0, 129.2, 128.5, 112.0, 43.1.

**Phenyl 2-thiocyanatoacetate (2g):**

Prepared according to **GP-C**.

**Yield:** 82% (792 mg).

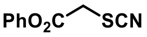


**2g**

**Nature:** Colourless oil.

**$R_f$ value** = 0.42 [EtOAc:Petroleum ether = 1:19 (v/v)].

**$^1$H NMR (400 MHz, $CDCl_3$) *δ* (ppm):** 7.44 – 7.39 (m, 2H), 7.31 – 7.27 (m, 1H), 7.17 – 7.14 (m, 2H), 3.97 (s, 2H).

**$^{13}$C{$^1$H} NMR (101 MHz, $CDCl_3$) *δ* (ppm):** 165.1, 150.2, 129.8, 126.8, 121.1, 110.6, 35.0.

**HRMS** (ESI) *m/z* calcd for $C_9H_8NO_2S$ $[M+H]^+$: 194.0276; found: 194.0261.

**Ethyl 2-thiocyanatoacetate (2h):**[16]

Prepared according to **GP-C**.

**Yield:** 83% (603 mg).

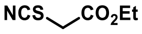


**2h**

**Nature:** Colourless oil.

**$R_f$ value** = 0.41 [EtOAc:Petroleum ether = 1:9 (v/v)].

**$^1$H NMR (400 MHz, $CDCl_3$) *δ* (ppm):** 4.24 (q, *J* = 7.1 Hz, 2H), 3.75 (s, 2H), 1.29 (t, *J* = 7.1 Hz, 3H).

**$^{13}$C{$^1$H} NMR (101 MHz, $CDCl_3$) *δ* (ppm):** 166.3, 110.9, 62.9, 35.0, 14.0.

**2-Thiocyanatoacetonitrile (2i):**

Prepared according to **GP-C**.

**Yield:** 85% (417 mg).

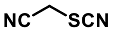


**2i**

**Nature:** Colourless oil.

**$R_f$ value** = 0.40 [EtOAc:Petroleum ether = 1:19 (v/v)].

**$^1$H NMR (400 MHz, $CDCl_3$) *δ* (ppm):** 3.79 (s, 2H).

**$^{13}$C{$^1$H} NMR (101 MHz, $CDCl_3$) *δ* (ppm):** 114.0, 109.3, 19.1.

**HRMS** (EI) *m/z* calcd for $C_3H_2N_2S$ $[M]^+$: 97.9939; found: 97.9937.

**4-(Thiocyanatomethyl)benzonitrile (2j):**[15]

Prepared according to **GP-C**.

**Yield:** 94% (819 mg).

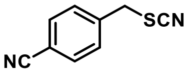


**2j**

**Nature:** White solid.

**$R_f$ value** = 0.42 [EtOAc:Petroleum ether = 1:19 (v/v)].

**$^1$H NMR (400 MHz, $CDCl_3$) *δ* (ppm):** 7.70 (d, *J* = 8.2 Hz, 2H), 7.49 (d, *J* = 8.2 Hz, 2H), 4.15 (s, 2H).

**$^{13}$C{$^1$H} NMR (101 MHz, $CDCl_3$) *δ* (ppm):** 139.8, 133.0, 129.8, 118.2, 113.0, 111.1, 37.5.

**Diethyl 2-methyl-2-thiocyanatomalonate (2k):**[14]

Prepared according to **GP-D**.

**Yield:** 93% (1.08 g).

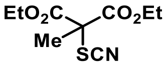


**2k**

**Nature:** Colourless oil.

**$R_f$ value** = 0.42 [EtOAc:Petroleum ether = 1:9 (v/v)].

**$^1$H NMR (400 MHz, $CDCl_3$) *δ* (ppm):** 4.29 (q, *J* = 7.1, Hz, 2H), 4.28 (q, *J* = 7.1, Hz, 2H), 1.94 (s, 3H), 1.29 (t, *J* = 7.1 Hz, 6H).

**$^{13}$C{$^1$H} NMR (101 MHz, $CDCl_3$) *δ* (ppm):** 166.6, 109.5, 63.7, 60.8, 22.5, 13.9.

**Preparation of ethyl 2-methyl-2-thiocyanatopropanoate (2l):** To a solution of the ethyl α-bromoisobutyrate (734 µL, 5 mmol) in 50 mL acetonitrile, KSCN (729 mg, 7.5 mmol) was added and the mixture was refluxed for 6 h. After the reaction was completed (monitored by TLC), the reaction mixture was quenched with water (30 mL) and extracted with EtOAc (3×50 mL). The combined organic layers were washed with brine (50 mL), dried over $Na_2SO_4$, and concentrated under vacuo. The residue was purified by silica gel column chromatography using EtOAc/petroleum ether as eluent to afford the corresponding alkylthiocyanate product.

**Ethyl 2-methyl-2-thiocyanatopropanoate (2l):**[17]

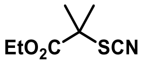


2l

**Yield:** 83% (719 mg).

**Nature:** Colourless oil.

**$R_f$ value** = 0.38 [EtOAc:Petroleum ether = 1:19 (v/v)].

**$^1$H NMR (400 MHz, $CDCl_3$) $\delta$ (ppm):** 4.27 (q, $J$ = 7.1 Hz, 2H), 1.76 (s, 6H), 1.32 (t, $J$ = 7.1 Hz, 3H).

**$^{13}$C{$^1$H} NMR (101 MHz, $CDCl_3$) $\delta$ (ppm):** 171.4, 110.9, 63.0, 55.3, 26.9, 14.1.

**Preparation of 2-nitro-2-thiocyanatopropane (2m):**[18] Freshly distilled 2-nitropropane (897 µL, 10 mmol) was dissolved in a solution of sodium hydroxide (440 mg, 11 mmol) in water (6 mL). The solution was added dropwise to a two-phase solution of sodium thiocyanate (1.62 g, 20 mmol) and potassium ferricyanide (6.59 g, 20 mmol) in water (6 mL) and dichloromethane (15 mL) at a rate such that the temperature remained between 20 °C and 25 °C. The reaction mixture was stirred for 20 min. The organic layer was separated, and the aqueous layer was extracted with diethyl ether. The combined organic extracts were washed with brine, dried over $Na_2SO_4$, and concentrated under vacuo. The crude was purified by silica gel flash column chromatography using EtOAc/petroleum ether as eluent to afford the corresponding product **2m**.

**2-Nitro-2-thiocyanatopropane (2m):**

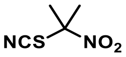


2m

**Yield:** 39% (570 mg).

**Nature:** Yellow oil.

**$R_f$ value** = 0.32 [EtOAc:Petroleum ether = 1:19 (v/v)].

**$^1$H NMR (400 MHz, $CDCl_3$) $\delta$ (ppm):** 2.10 (s, 6H).

**$^{13}$C{$^1$H} NMR (101 MHz, $CDCl_3$) $\delta$ (ppm):** 107.8, 93.7, 27.4.

**Ethyl 2-fluoro-2-thiocyanatoacetate (2n):**

Prepared according to **GP-C**.

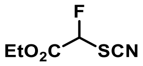


3n

**Yield:** 83% (677 mg).

**Nature:** Colourless oil.

**$R_f$ value** = 0.41 [EtOAc:Petroleum ether = 1:19 (v/v)].

**$^1$H NMR (400 MHz, $CDCl_3$) $\delta$ (ppm):** 6.18 (d, $J$ = 49.2 Hz, 1H), 4.40 (q, $J$ = 7.1 Hz, 2H), 1.38 (t, $J$ = 7.1 Hz, 3H).

**$^{13}C\{^1H\}$ NMR (101 MHz, $CDCl_3$) $\delta$ (ppm):** 163.0 (d, $J$ = 25.6 Hz), 106.6, 90.8 (d, $J$ = 249.0 Hz), 64.3, 14.1.

**$^{19}F$ NMR (377 MHz, $CDCl_3$) δ (ppm):** -160.9.

**HRMS** (ESI) *m/z* calcd for $C_5H_7FNO_2SNa$ $[M+H]^+$: 164.0182; found: 164.0166.

**Diethyl 2-selenocyanatomalonate (2o):**

Prepared according to **GP-C**.

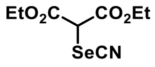

2o

**Yield:** 78% (1.03 g).

**Nature:** Pale yellow oil.

**$R_f$ value** = 0.31 [EtOAc:Petroleum ether = 1:19 (v/v)].

**$^1H$ NMR (400 MHz, $CDCl_3$) $\delta$ (ppm):** 4.93 (s, 1H), 4.33 (q, $J$ = 7.1, Hz, 2H), 4.32 (q, $J$ = 7.1, Hz, 2H), 1.34 (t, $J$ = 7.1 Hz, 6H).

**$^{13}C\{^1H\}$ NMR (101 MHz, $CDCl_3$) $\delta$ (ppm):** 165.1, 100.1, 63.9, 46.7, 14.1.

**HRMS** (ESI) *m/z* calcd for $C_8H_{11}NO_4SeNa$ $[M+Na]^+$: 287.9751; found: 287.9753.

**Diisopropyl 2-selenocyanatomalonate (2p):**

Prepared according to **GP-C**.

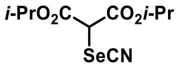

2p

**Yield:** 76% (1.11 g).

**Nature:** Colourless oil.

**$R_f$ value** = 0.31 [EtOAc:Petroleum ether = 1:19 (v/v)].

**$^1H$ NMR (400 MHz, $CDCl_3$) $\delta$ (ppm):** 5.16 – 5.07 (m, 2H), 4.86 (s, 1H), 1.31 (d, $J$ = 3.8 Hz, 6H), 1.29 (d, $J$ = 3.8 Hz, 6H).

**$^{13}C\{^1H\}$ NMR (101 MHz, $CDCl_3$) $\delta$ (ppm):** 164.6, 100.3, 72.2, 47.3, 21.6, 21.5.

**HRMS** (ESI) *m/z* calcd for $C_{10}H_{15}NO_4SeNa$ $[M+Na]^+$: 316.0064; found: 316.0069.

**1-(4-Chlorophenyl)-2-selenocyanatoethan-1-one (2q):**[19]

Prepared according to **GP-C**.

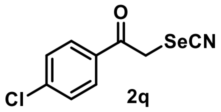

2q

**Yield:** 89% (1.15 g).

**Nature:** White solid.

**$R_f$ value** = 0.33 [EtOAc:Petroleum ether = 1:19 (v/v)].

**$^1H$ NMR (400 MHz, $CDCl_3$) $\delta$ (ppm):** 7.90 (d, $J$ = 8.7 Hz, 2H), 7.51 (d, $J$ = 8.7 Hz, 2H), 4.88 (s, 2H).

**$^{13}C\{^1H\}$ NMR (101 MHz, $CDCl_3$) $\delta$ (ppm):** 192.2, 141.7, 132.3, 130.2, 129.7, 101.7, 38.0.

**Bis(4-methoxyphenyl) 2-thiocyanatomalonate (2r):**

Prepared according to **GP-A**.

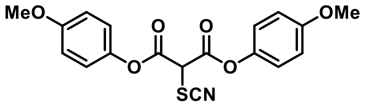

2r

**Yield:** 39% (1.46 g).

**Nature:** Colourless oil.

**$R_f$ value** = 0.41 [EtOAc:Petroleum ether = 2:3 (v/v)].

**$^1H$ NMR (400 MHz, $CDCl_3$) $\delta$ (ppm):** 7.12 (d, $J$ = 9.1 Hz, 4H), 6.92 (d, $J$ = 9.1 Hz, 4H), 5.12 (s, 1H), 3.81 (s, 6H).

**$^{13}C\{^1H\}$ NMR (101 MHz, $CDCl_3$) $\delta$ (ppm):** 162.8, 158.2, 143.7, 121.8, 114.9, 108.7, 55.8, 52.1.

**HRMS** (ESI) *m/z* calcd for $C_{18}H_{15}NO_6SNa$ $[M+Na]^+$: 396.0518; found: 396.0519.

**Diethyl 2-phenyl-2-thiocyanatomalonate (2s):**[14]

Prepared according to **GP-D**.

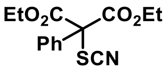


2s

**Yield:** 87% (1.28 g).

**Nature:** Colourless oil.

**$R_f$ value** = 0.35 [EtOAc:Petroleum ether = 1:9 (v/v)].

**$^1$H NMR (400 MHz, $CDCl_3$) $\delta$ (ppm):** 7.53 – 7.50 (m, 2H), 7.45 – 7.40 (m, 3H), 4.41 – 4.32 (m, 4H), 1.31 (t, $J$ = 7.1 Hz, 6H).

**$^{13}$C{$^1$H} NMR (101 MHz, $CDCl_3$) $\delta$ (ppm):** 165.9, 132.9, 129.7, 128.9, 128.2, 109.9, 69.5, 64.0, 13.8.

**(Thiocyanatomethylene)dibenzene (2t):**[20]

Prepared according to **GP-C**.

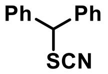


2t

**Yield:** 78% (879 mg).

**Nature:** Colourless oil.

**$R_f$ value** = 0.31 [EtOAc:Petroleum ether = 1:19 (v/v)].

**$^1$H NMR (400 MHz, $CDCl_3$) $\delta$ (ppm):** 7.48 – 7.33 (m, 10H), 5.85 (s, 1H).

**$^{13}$C{$^1$H} NMR (101 MHz, $CDCl_3$) $\delta$ (ppm):** 137.6, 129.2, 128.9, 128.3, 111.8, 57.5.

**2-Thiocyanatopropane (2u):**[21]

Prepared according to **GP-C** and heated at 60 °C.

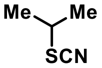


2u

**Yield:** 64% (324 mg).

**Nature:** Colourless oil.

**$R_f$ value** = 0.48 [EtOAc:Petroleum ether = 1:19 (v/v)].

**$^1$H NMR (400 MHz, $CDCl_3$) $\delta$ (ppm):** 3.51 – 3.44 (m 1H), 1.49 (d, $J$ = 6.7 Hz, 6H).

**$^{13}$C{$^1$H} NMR (101 MHz, $CDCl_3$) $\delta$ (ppm):** 111.6, 40.7, 23.7.

## 4. Reaction Optimization:

**General Procedure for Reaction Optimization:** An oven-dried culture tube equipped with a magnetic stir bar was charged with photo-catalyst (x mol%), diethyl 2-thiocyanatomalonate **2a** (44 mg, 0.2 mmol), and dry solvent (y mL). The tube was sealed with a Teflon screw cap before 4-methylstyrene **1a** (53 μL, 0.4 mmol) was added to it. Then, the reaction mixture was degassed by Freeze-Pump-Thaw cycles with argon and irradiated at rt with LEDs at a distance of approximately 5 cm for a specific time. A high-speed fan was used to maintain the temperature. After the completion of the ATRA reaction (confirmed by TLC), Aluminum chloride (27 mg, 0.2 mmol) was added to the ice-cold reaction mixture. After 1 h, 2 mL of ethyl acetate was added and quenched with saturated ammonium chloride solution (2 mL). The crude reaction mixture was extracted with ethyl acetate (2×2 mL), washed with brine (3 mL), and dried over anhydrous $Na_2SO_4$. The organic portion was concentrated, and $^1H$ NMR was recorded using 1,1,2,2-tetrachloroethane as internal standard.

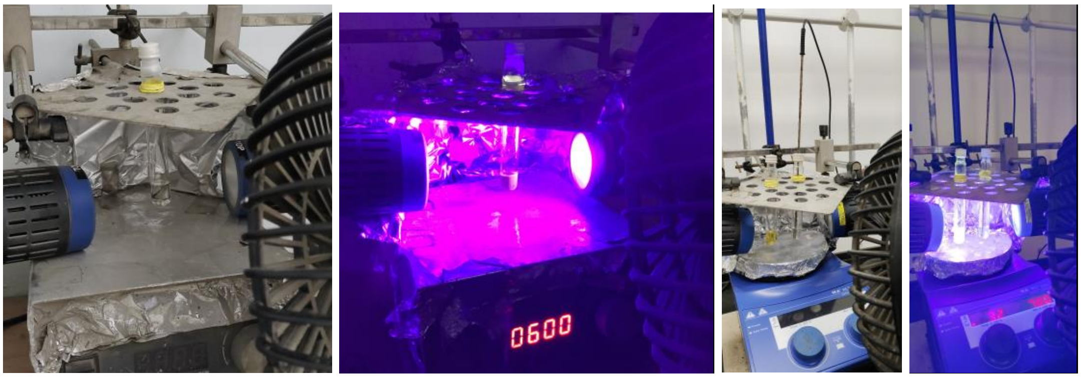


***Experimental set-up***

**Supplementary Table 1:** Optimization of the reaction condition:[a]

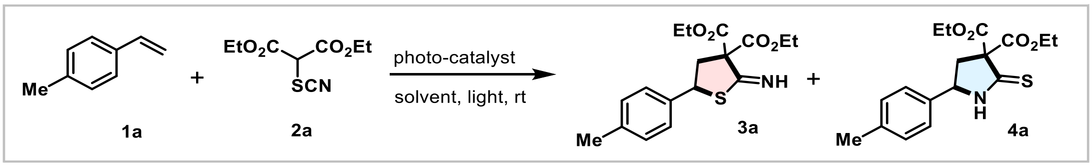


| Entry | Catalyst[a] | Solvent | Light (nm) | Time | 3a (%)[b] | 4a (%)[b] |
|---|---|---|---|---|---|---|
| 1 | PTH1 | Toluene | 390 | 1 h | 38 | 2 |
| 2 | PTH2 | Toluene | 390 | 30 min | 89 (86)[c] | 7 |
| 3 | PTH3 | Toluene | 390 | 20 min | 68 | 21 |
| 4 | PTH4 | Toluene | 390 | 20 min | 70 | 22 |
| 5 | *fac*-Ir(ppy)$_3$ (1 mol%) | Toluene | 450 | 6 h | 67 | 8 |
| 6 | PTH2 | $CH_3CN$ | 390 | 45 min | 73 | 6 |
| 7 | PTH2 | $CH_3NO_2$ | 390 | 45 min | 64 | 5 |
| 8 | PTH2 | EtOAc | 390 | 30 min | 84 | 6 |
| 9 | PTH2 | Acetone | 390 | 30 min | 61 | 5 |
| 10 | PTH2 | THF | 390 | 45 min | 56 | 2 |
| 11 | PTH2 | DMF | 390 | 1 h | 38 | 2 |

| | | | | | | |
|---|---|---|---|---|---|---|
| 12 | PTH2 | DMSO | 390 | 1 h | 35 | 2 |
| 13 | PTH2 | 1,2-DCE | 390 | 30 min | 76 | 5 |
| 14 | PTH2 | Ethanol | 390 | 4 h | 63 | 4 |
| 15[d] | PTH2 | Toluene | 390 | 30 min | 83 | 6 |
| 16[e] | PTH2 | Toluene | 390 | 30 min | 81 | 6 |
| 17[f] | PTH2 | Toluene | 390 | 30 min | 78 | 5 |
| 18 | - | Toluene | 390 | 30 min | 2 | 0 |
| 19 | - | Toluene | 390 | 24 h | 4 | 0 |
| 20 | PTH2 | Toluene | - | 30 min | 0 | 0 |
| 21[g] | PTH2 | Toluene | - | 24 h | 3 | 0 |
| 22[g] | - | Toluene | - | 24 h | 3 | 0 |
| 23 | PTH1 | Toluene | 390 | 24 h | 35 | 3 |
| 24 | PTH2 | Toluene | 390 | 24 h | 82 | 11 |
| 25 | PTH3 | Toluene | 390 | 24 h | 4 | 76 |
| 26 | PTH4 | Toluene | 390 | 24 h | 3 | 81 (79)[c] |
| 27 | *fac*-Ir(ppy)$_3$ (1 mol%) | Toluene | 450 | 24 h | 61 | 12 |

[a]Conditions: **1a** (0.4 mmol), **2a** (0.2 mmol), photo-catalyst (5 mol%), solvent (2 mL), degassed condition, irradiation with LEDs light in rt; after completion of photo-reaction, 1 equivalent $AlCl_3$ (0.2 mmol) was added at 0 °C and stirred for 1 h; [b]Crude $^1$H NMR yield (%) using 1,1,2,2-tetrachloroethane as internal standard; [c]Isolated yield; [d]Toluene (1.3 mL); [e]Toluene (4 mL); [f]Reactions performed in open air; [g]Reactions performed at 60 ºC.

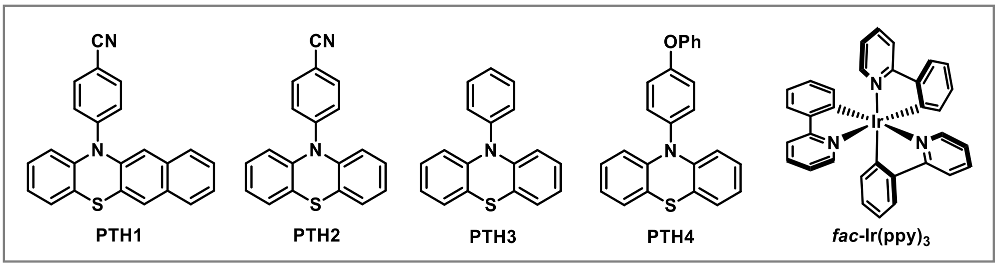


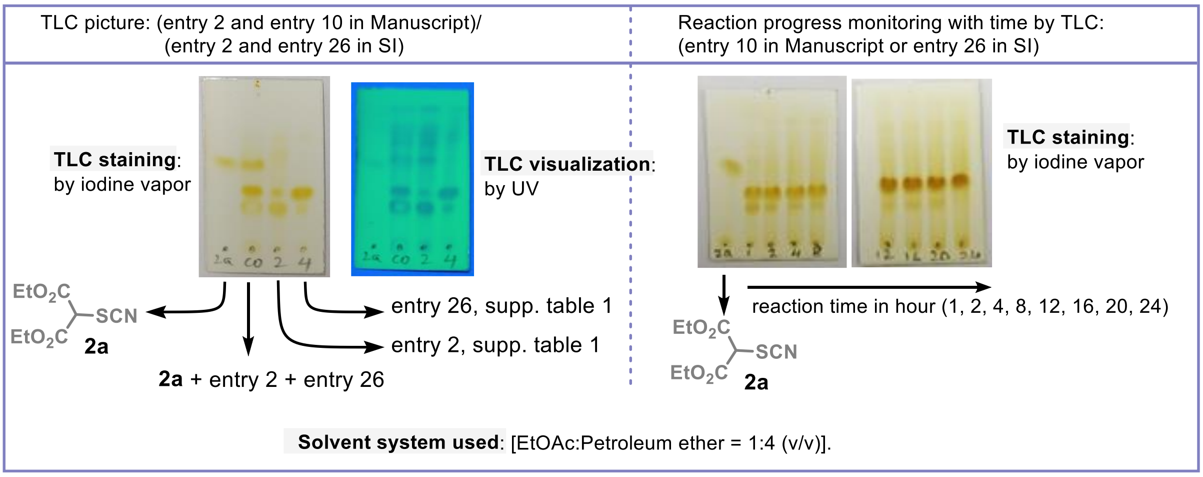

## 5. Reaction Generality:

### 5.1. Synthetic Procedures:

**General Procedure for Photocatalytic 2-Imino-Tetrahydrothiophenes Synthesis (GP-1):**

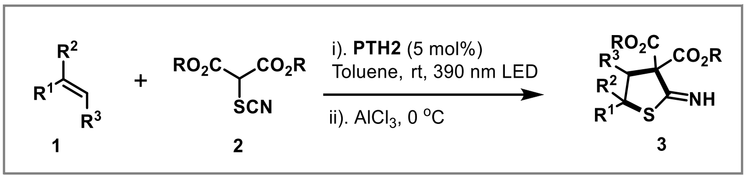


An oven-dried culture tube equipped with a magnetic stir bar was charged with **PTH2** (3 mg, 0.01 mmol, 5 mol%), thiocyanatomalonate **2** (0.2 mmol) and dry toluene (2 mL). The tube was sealed with a Teflon screw cap before olefin **1** (0.4 mmol aromatic olefin/ 0.8 mmol aliphatic olefin/ 0.2 mmol acrylate) was added to it. Then the reaction mixture was degassed by Freeze-Pump-Thaw cycles with argon and irradiated at rt with 390 nm LEDs at a distance of approximately 5 cm for 30 min (1 h for aliphatic olefin and acrylate). A high-speed fan was used to maintain the temperature. After the completion of the ATRA reaction (confirmed by TLC), Aluminum chloride (27 mg, 0.2 mmol) was added to the ice-cold reaction mixture. After 1 h, 2 mL of ethyl acetate was added and quenched with saturated ammonium chloride solution (2 mL). The crude reaction mixture was extracted with ethyl acetate (2×2 mL), washed with brine (3 mL), and dried over anhydrous $Na_2SO_4$. The organic portion was concentrated, and the residue was purified by silica gel column chromatography using EtOAc/petroleum ether as eluent to afford the corresponding 2-imino-tetrahydrothiophene product **3**.

**General Procedure for Photocatalytic Thiopyrrolidinones Synthesis (GP-2):**

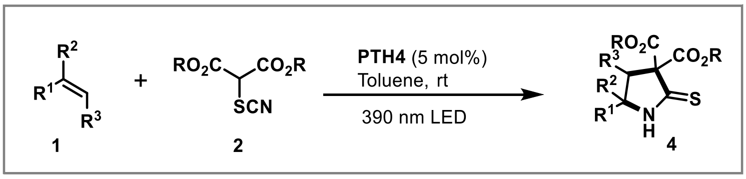


An oven-dried culture tube equipped with a magnetic stir bar was charged with **PTH4** (3.7 mg, 0.01 mmol, 5 mol%), thiocyanatomalonate **2** (0.2 mmol) and dry toluene (2 mL). The tube was sealed with a Teflon screw cap before olefin **1** (0.4 mmol) was added to it. Then, the reaction mixture was degassed by Freeze-Pump-Thaw cycles with argon and irradiated at rt with 390 nm LEDs at a distance of approximately 5 cm for 24 h (for products **4s-z**: reaction time 6 h). A high-speed fan was used to maintain the temperature. After the completion of the reaction (confirmed by TLC), reaction crude was concentrated and purified by silica gel column chromatography using EtOAc/petroleum ether as eluent to afford the corresponding thiopyrrolidinone product **4**.

**General Procedure for Photocatalytic Alkyl-thiocyanation of Alkenes (GP-3):**

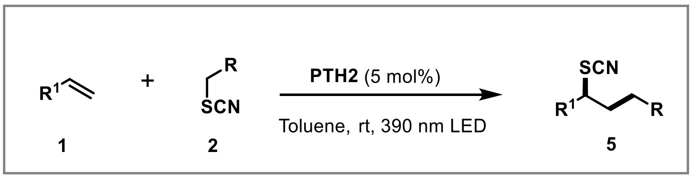

An oven-dried culture tube equipped with a magnetic stir bar was charged with **PTH2** (3 mg, 0.01 mmol, 5 mol%), alkylthiocyanate **2** (0.2 mmol) and dry toluene (2 mL). The tube was sealed with a Teflon screw cap before olefin **1** (0.4 mmol) was added to it. Then, the reaction mixture was degassed by Freeze-Pump-Thaw cycles with argon and irradiated at rt with 390 nm LEDs at a distance of approximately 5 cm for 30 min. A high-speed fan was used to maintain the temperature. After the completion of the reaction (confirmed by TLC), reaction crude was concentrated and purified by silica gel column chromatography using EtOAc/petroleum ether as eluent to afford the corresponding alkylthiocyanation product **5**.

**General Procedure for Photocatalytic Alkyl-isothiocyanation of Alkenes (GP-4):**

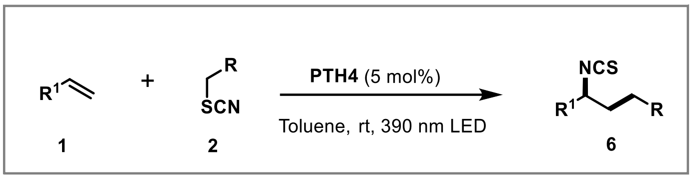


An oven-dried culture tube equipped with a magnetic stir bar was charged with **PTH4** (3.7 mg, 0.01 mmol, 5 mol%), alkylthiocyanate **2** (0.2 mmol) and dry toluene (2 mL). The tube was sealed with a Teflon screw cap before olefin **1** (0.4 mmol) was added to it. Then, the reaction mixture was degassed by Freeze-Pump-Thaw cycles with argon and irradiated at rt with 390 nm LEDs at a distance of approximately 5 cm for 24 h. A high-speed fan was used to maintain the temperature. After the completion of the reaction (confirmed by TLC), reaction crude was concentrated and purified by silica gel column chromatography using EtOAc/petroleum ether as eluent to afford the corresponding alkyl-isothiocyanation product **6**.

**General Procedure for Photocatalytic 2-Imino-Tetrahydroselenophenes Synthesis (GP-5):**

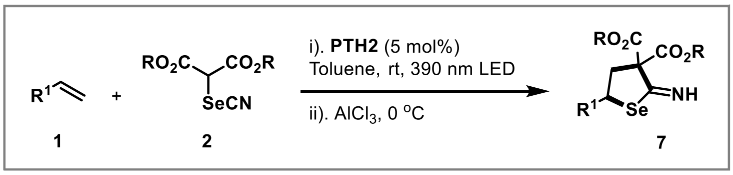


An oven-dried culture tube equipped with a magnetic stir bar was charged with **PTH2** (3 mg, 0.01 mmol, 5 mol%), selenocyanatomalonate **2** (0.2 mmol) and dry toluene (2 mL). The tube was sealed with a Teflon screw cap before olefin **1** (0.4 mmol) was added to it. Then, the reaction mixture was degassed by Freeze-Pump-Thaw cycles with argon and irradiated at rt with 390 nm LEDs at a distance of approximately 5 cm for 30 min. A high-speed fan was used to maintain the temperature. After the completion of the ATRA reaction (confirmed by TLC), Aluminum chloride (27 mg, 0.2 mmol) was added to the ice-cold reaction mixture. After 1 h, 2 mL of ethyl acetate was added and quenched with saturated ammonium chloride (2 mL). The crude reaction mixture was extracted with ethyl acetate (2×2 mL), washed with brine (3 mL), and dried over anhydrous $Na_2SO_4$. The organic portion was concentrated, and the residue was quickly purified by silica gel column chromatography using EtOAc/petroleum ether as eluent to afford the corresponding 2-imino-tetrahydroselenophene product **7**.

**Supplementary Notes:** *These products are relatively sensitive to air and moisture. Long-run purification on column chromatography leads to the degradation of the product, and a quick passing through the silica pad presents the current yield.*

**General Procedure for Photocatalytic Selenoxopyrrolidinones Synthesis (GP-6):**

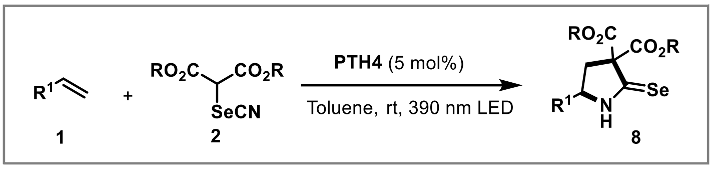


An oven-dried culture tube equipped with a magnetic stir bar was charged with **PTH4** (3.7 mg, 0.01 mmol, 5 mol%), selenocyanatomalonate **2** (0.2 mmol) and dry toluene (2 mL). The tube was sealed with a Teflon screw cap before olefin **1** (0.4 mmol) was added to it. Then, the reaction mixture was degassed by Freeze-Pump-Thaw cycles with argon and irradiated at rt with 390 nm LEDs at a distance of approximately 5 cm for 24 h. A high-speed fan was used to maintain the temperature. After the completion of the reaction (confirmed by TLC), reaction crude was concentrated and purified by silica gel column chromatography using EtOAc/petroleum ether as eluent to afford the corresponding selenoxopyrrolidine product **8**.

**Procedure of gram scale synthesis of 3a:**

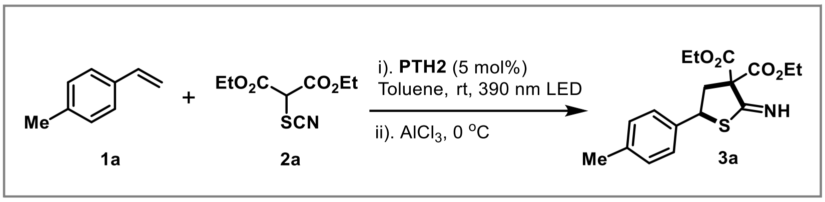


Following **GP-1**, a 250 mL round bottom flask equipped with a magnetic stir bar was charged with **PTH2** (90 mg, 0.3 mmol, 5 mol%), diethyl 2-thiocyanatomalonate **2a** (1.3 g, 6 mmol) and dry toluene (60 mL). The RB flask was sealed with a septum before 4-methylstyrene **1a** (1.58 mL, 12 mmol) was added to it. Then, the reaction mixture was degassed by Freeze-Pump-Thaw cycles with argon and irradiated at rt with 390 nm LEDs at a distance of approximately 5 cm for 1 h. A high-speed fan was used to maintain the temperature. After the completion of the ATRA reaction (confirmed by TLC), Aluminum chloride (800 mg, 6 mmol) was added to the ice-cold reaction mixture. After 1 h, 50 mL of ethyl acetate was added and quenched with saturated ammonium chloride (50 mL). The crude reaction mixture was extracted with ethyl acetate (2×50 mL), washed with brine (75 mL), and dried over anhydrous $Na_2SO_4$. The organic portion was concentrated, and the residue was purified by silica gel column chromatography using EtOAc/petroleum ether as eluent to afford the corresponding product **3a** with 81% (1.63 g) yield.

**Procedure of gram scale synthesis of 4a:**

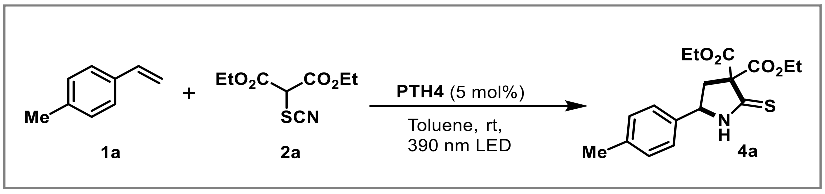


Following **GP-2**, a 250 mL round bottom flask equipped with a magnetic stir bar was charged with **PTH4** (110 mg, 0.3 mmol, 5 mol%), diethyl 2-thiocyanatomalonate **2a** (1.3 g, 6 mmol) and dry toluene (60 mL). The RB flask was sealed with a septum before 4-methylstyrene **1a** (1.58 mL, 12 mmol) was added to it. Then, the reaction mixture was degassed by Freeze-Pump-Thaw cycles with argon and irradiated at rt with 390 nm LEDs at a distance of approximately 5 cm for 30 h. A high-speed fan was used to maintain the temperature. After the completion of the reaction (confirmed by TLC), reaction crude was concentrated and purified by silica gel column chromatography using EtOAc/petroleum ether as eluent to afford the corresponding Thioxopyrrolidine product **4a** with 72% (1.45 g) yield.

## 5.2. Compound Characterization Data:

### Diethyl 2-(2-thiocyanato-2-(*p*-tolyl)ethyl)malonate (3a’):

*After the photo-ATRA reaction, the mixture was concentrated and was quickly (within 3-5 minutes) passed through the silica gel column to avoid cyclization.*

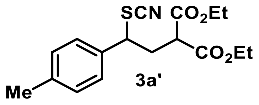


3a'

**Yield:** 85% (57 mg).

**Nature:** Colourless oil.

**$R_f$ value** = 0.36 [EtOAc:Petroleum ether = 1:9 (v/v)].

**$^1$H NMR (400 MHz, $CDCl_3$) *δ* (ppm):** 7.22 (d, *J* = 8.6 Hz, 2H), 7.19 (d, *J* = 8.7 Hz, 2H), 4.44 (dd, *J* = 9.5, 6.4 Hz, 1H), 4.25 – 4.10 (m, 4H), 3.29 (dd, *J* = 8.6, 6.1 Hz, 1H), 2.81 – 2.74 (m, 1H), 2.71 – 2.64 (m, 1H), 2.35 (s, 3H), 1.27 (t, *J* = 7.1 Hz, 3H), 1.24 (t, *J* = 7.1 Hz, 3H).

**$^{13}$C{$^1$H} NMR (101 MHz, $CDCl_3$) *δ* (ppm):** 168.3, 168.2, 139.6, 133.8, 130.2, 127.6, 111.1, 62.0, 50.9, 49.8, 34.6, 21.3, 14.2, 14.1.

**HRMS** (ESI) *m/z* calcd for $C_{17}H_{22}NO_4S$ $[M+H]^+$: 336.1270; found: 336.1275.

### Diethyl 2-imino-5-(*p*-tolyl)dihydrothiophene-3,3(2*H*)-dicarboxylate (3a):

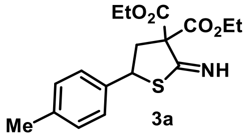


3a

**Yield:** 86% (58 mg).

**Nature:** Colourless oil.

**$R_f$ value** = 0.31 [EtOAc:Petroleum ether = 1:4 (v/v)].

**$^1$H NMR (400 MHz, $CDCl_3$) *δ* (ppm):** 7.32 (d, *J* = 8.1 Hz, 2H), 7.17 (d, *J* = 7.9 Hz, 2H), 4.80 (dd, *J* = 11.6, 4.9 Hz, 1H), 4.34 (q, *J* = 7.1 Hz, 2H), 4.31 – 4.25 (m, 2H), 3.09 (dd, *J* = 13.2, 5.0 Hz, 1H), 2.90 (t, *J* = 12.5 Hz, 1H), 2.34 (s, 3H), 1.34 (t, *J* = 7.1 Hz, 3H), 1.29 (t, *J* = 7.1 Hz, 3H).

**$^{13}$C{$^1$H} NMR (101 MHz, $CDCl_3$) *δ* (ppm):** 177.2, 166.9, 138.5, 134.9, 129.7, 127.7, 70.5, 63.0, 62.9, 50.6, 45.5, 21.3, 14.1.

**HRMS** (ESI) *m/z* calcd for $C_{17}H_{22}NO_4S$ $[M+H]^+$: 336.1270; found: 336.1243.

### Diethyl 2-imino-5-phenyldihydrothiophene-3,3(2*H*)-dicarboxylate (3b):

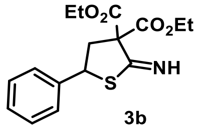


3b

**Yield:** 81% (52 mg).

**Nature:** Colourless oil.

**$R_f$ value** = 0.37 [EtOAc:Petroleum ether = 1:4 (v/v)].

**$^1$H NMR (400 MHz, $CDCl_3$) *δ* (ppm):** 7.45 – 7.42 (m, 2H), 7.39 – 7.29 (m, 3H), 4.84 (dd, *J* = 11.6, 5.0 Hz, 1H), 4.38 – 4.32 (m, 2H), 4.28 (q, *J* = 7.1 Hz, 2H), 3.14 (dd, *J* = 13.2, 5.0 Hz, 1H), 2.91 (dd, *J* = 13.2, 11.7 Hz, 1H), 1.34 (t, *J* = 7.1 Hz, 3H), 1.29 (t, *J* = 7.1 Hz, 3H).

**$^{13}$C{$^1$H} NMR (101 MHz, $CDCl_3$) *δ* (ppm):** 177.9, 166.8, 166.7, 137.8, 129.0, 128.6, 127.9, 70.3, 63.1, 63.0, 50.9, 45.4, 14.1, 14.0.

**HRMS** (ESI) *m/z* calcd for $C_{16}H_{20}NO_4S$ $[M+H]^+$: 322.1113; found: 322.1114.

### Diethyl 5-(4-(*tert*-butyl)phenyl)-2-iminodihydrothiophene-3,3(2*H*)-dicarboxylate (3c):

**Yield:** 87% (66 mg).

**Nature:** Colourless oil.

**$R_f$ value** = 0.38 [EtOAc:Petroleum ether = 1:4 (v/v)].

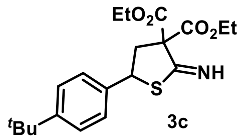

3c

**$^1$H NMR (400 MHz, $CDCl_3$) *δ* (ppm):** 9.83 (brs, 1H), 7.39 (d, *J* = 9.0 Hz, 1H), 7.36 (d, *J* = 8.2 Hz, 1H), 4.83 (dd, *J* = 11.6, 5.0 Hz, 1H), 4.38 – 4.32 (m, 2H), 4.27 (q, *J* = 7.1 Hz, 2H), 3.12 (dd, *J* = 13.2, 5.0 Hz, 1H), 2.91 (t, *J* = 12.4 Hz, 1H), 1.34 (t, *J* = 7.1 Hz, 3H), 1.31 (s, 9H), 1.29 (t, *J* = 7.1 Hz, 3H).

**$^{13}$C{$^1$H} NMR (101 MHz, $CDCl_3$) *δ* (ppm):** 166.9, 151.7, 134.8, 127.6, 126.0, 63.1, 62.9, 45.4, 34.8, 31.4, 14.1.

**HRMS** (ESI) *m/z* calcd for $C_{20}H_{28}NO_4S$ $[M+H]^+$: 378.1739; found: 378.1759.

**Diethyl 5-(4-chlorophenyl)-2-iminodihydrothiophene-3,3(2*H*)-dicarboxylate (3d):**

**Yield:** 86% (61 mg).

**Nature:** Colourless oil.

**$R_f$ value** = 0.31 [EtOAc:Petroleum ether = 1:4 (v/v)].

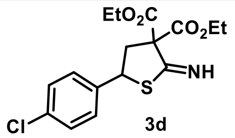

3d

**$^1$H NMR (400 MHz, DMSO-$d_6$) *δ* (ppm):** 10.96 (brs, 1H), 7.50 (d, *J* = 8.5 Hz, 2H), 7.44 (d, *J* = 8.5 Hz, 2H), 4.88 (dd, *J* = 11.0, 4.9 Hz, 1H), 4.28 – 4.23 (m, 2H), 4.16 – 4.11 (m, 2H), 2.97 (dd, *J* = 13.0, 5.1 Hz, 1H), 2.79 (dd, *J* = 12.9, 11.3 Hz, 1H), 1.23 (t, *J* = 7.1 Hz, 3H), 1.16 (t, *J* = 7.1 Hz, 3H).

**$^{13}$C{$^1$H} NMR (101 MHz, DMSO-$d_6$) *δ* (ppm):** 171.3, 166.9, 166.8, 137.4, 132.7, 129.7, 128.8, 70.5, 62.2, 62.0, 48.9, 44.1, 13.9, 13.8.

**HRMS** (ESI) *m/z* calcd for $C_{16}H_{19}ClNO_4S$ $[M+H]^+$: 356.0723; found: 356.0716.

**Diethyl 5-(4-fluorophenyl)-2-iminodihydrothiophene-3,3(2*H*)-dicarboxylate (3e):**

**Yield:** 88% (60 mg).

**Nature:** Colourless oil.

**$R_f$ value** = 0.49 [EtOAc:Petroleum ether = 2:3 (v/v)].

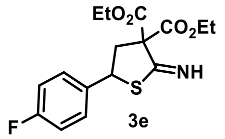

3e

**$^1$H NMR (400 MHz, DMSO-$d_6$) *δ* (ppm):** 10.93 (brs, 1H), 7.54 – 7.50 (m, 2H), 7.23 – 7.18 (m, 2H), 4.87 (dd, *J* = 10.7, 4.3 Hz, 1H), 4.26 (q, *J* = 7.1 Hz, 1H), 4.19 – 4.08 (m, 2H), 2.96 (dd, *J* = 13.0, 5.0 Hz, 1H), 2.80 (t, *J* = 12.9 Hz, 1H), 1.23 (t, *J* = 7.1 Hz, 3H), 1.17 (t, *J* = 7.1 Hz, 3H).

**$^{13}$C{$^1$H} NMR (101 MHz, DMSO-$d_6$) *δ* (ppm):** 171.5, 166.9, 166.8, 161.8 (d, *J* = 244.6 Hz), 134.5 (d, *J* = 2.8 Hz), 129.8 (d, *J* = 8.4 Hz), 115.7 (d, *J* = 21.6 Hz), 70.5, 62.2, 62.0, 48.9, 44.4, 13.9, 13.8.

**$^{19}$F NMR (377 MHz, DMSO-$d_6$) δ (ppm):** -113.2.

**HRMS** (ESI) *m/z* calcd for $C_{16}H_{19}FNO_4S$ $[M+H]^+$: 340.1019; found: 340.1004.

**Diethyl 2-imino-5-(3-methoxyphenyl)dihydrothiophene-3,3(2*H*)-dicarboxylate (3f):**

**Yield:** 88% (62 mg).

**Nature:** Colourless oil.

**$R_f$ value** = 0.35 [EtOAc:Petroleum ether = 1:4 (v/v)].

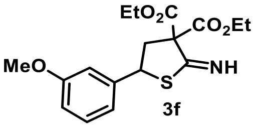

3f

**$^1$H NMR (400 MHz, CDCl$_3$) $\delta$ (ppm):** 7.26 (t, $J$ = 7.9 Hz, 1H), 7.00 (d, $J$ = 7.9 Hz, 1H), 6.98 – 6.96 (m, 1H), 6.85 – 6.82 (m, 1H), 4.80 (dd, $J$ = 11.5, 5.0 Hz, 1H), 4.37 – 4.31 (m, 2H), 4.27 (q, $J$ = 7.1 Hz, 2H), 3.80 (s, 3H), 3.12 (dd, $J$ = 13.2, 5.0 Hz, 1H), 2.89 (dd, $J$ = 13.2, 11.6 Hz, 1H), 1.33 (t, $J$ = 7.1 Hz, 3H), 1.28 (t, $J$ = 7.1 Hz, 3H).

**$^{13}$C{$^1$H} NMR (101 MHz, CDCl$_3$) $\delta$ (ppm):** 177.3, 166.8, 166.7, 160.0, 139.5, 130.0, 120.0, 114.0, 113.3, 70.3, 63.0, 62.9, 55.4, 50.7, 45.3, 14.1, 14.0.

**HRMS** (ESI) *m/z* calcd for $C_{17}H_{22}NO_5S$ [M+H]$^+$: 352.1219; found: 352.1202.

**Diethyl 5-(2-bromophenyl)-2-iminodihydrothiophene-3,3(2*H*)-dicarboxylate (3g):**

**Yield:** 76% (61 mg).

**Nature:** Colourless oil.

**R$_f$ value** = 0.30 [EtOAc:Petroleum ether = 1:4 (v/v)].

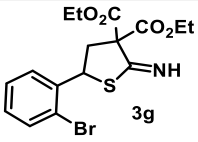


**$^1$H NMR (400 MHz, CDCl$_3$) $\delta$ (ppm):** 7.70 (dd, $J$ = 7.9, 1.6 Hz, 1H), 7.57 (dd, $J$ = 8.0, 1.2 Hz, 1H), 7.35 (td, $J$ = 7.6, 1.2 Hz, 1H), 7.19 – 7.15 (m, 1H), 5.34 (dd, $J$ = 10.9, 5.1 Hz, 1H), 4.40 – 4.32 (m, 2H), 4.25 (q, $J$ = 7.1 Hz, 2H), 3.23 (dd, $J$ = 13.2, 5.1 Hz, 1H), 2.79 (dd, $J$ = 12.9, 11.2 Hz, 1H), 1.35 (t, $J$ = 7.1 Hz, 3H), 1.27 (t, $J$ = 7.1 Hz, 3H).

**$^{13}$C{$^1$H} NMR (101 MHz, CDCl$_3$) $\delta$ (ppm):** 176.9, 166.8, 166.6, 137.2, 133.6, 129.8, 128.8, 128.3, 124.4, 70.0, 63.1, 62.9, 49.5, 44.0, 14.2, 14.1.

**HRMS** (ESI) *m/z* calcd for $C_{16}H_{19}BrNO_4S$ [M+H]$^+$: 400.0218; found: 400.0217.

**Diethyl 5-(3-bromophenyl)-2-iminodihydrothiophene-3,3(2*H*)-dicarboxylate (3h):**

**Yield:** 81% (65 mg).

**Nature:** Colourless oil.

**R$_f$ value** = 0.31 [EtOAc:Petroleum ether = 1:4 (v/v)].

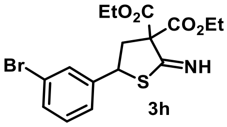


**$^1$H NMR (400 MHz, CDCl$_3$) $\delta$ (ppm):** 7.60 (t, $J$ = 1.7 Hz, 1H), 7.46 – 7.44 (m, 1H), 7.37 (d, $J$ = 7.8 Hz, 1H), 7.24 (t, $J$ = 7.8 Hz, 1H), 4.80 (dd, $J$ = 11.4, 5.1 Hz, 1H), 4.37 – 4.32 (m, 2H), 4.29 (q, $J$ = 7.1 Hz, 2H), 3.15 (dd, $J$ = 13.3, 5.1 Hz, 1H), 2.85 (dd, $J$ = 13.0, 11.7 Hz, 1H), 1.34 (t, $J$ = 7.1 Hz, 3H), 1.30 (t, $J$ = 7.1 Hz, 3H).

**$^{13}$C{$^1$H} NMR (101 MHz, CDCl$_3$) $\delta$ (ppm):** 178.2, 166.5, 166.2, 140.0, 131.8, 130.9, 130.6, 126.6, 123.1, 70.1, 63.4, 63.2, 50.3, 45.1, 14.1.

**HRMS** (ESI) *m/z* calcd for $C_{16}H_{19}BrNO_4S$ [M+H]$^+$: 400.0218; found: 422.0211.

**Diethyl 5-(4-bromophenyl)-2-iminodihydrothiophene-3,3(2*H*)-dicarboxylate (3i):**

**Yield:** 79% (63 mg).

**Nature:** Colourless oil.

**R$_f$ value** = 0.30 [EtOAc:Petroleum ether = 1:4 (v/v)].

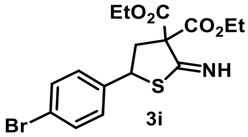


**$^1$H NMR (400 MHz, CDCl$_3$) $\delta$ (ppm):** 7.49 (d, $J$ = 8.5 Hz, 2H), 7.32 (d, $J$ = 8.5 Hz, 2H), 4.79 (dd, $J$ = 11.4, 5.0 Hz, 1H), 4.37 – 4.32 (m, 2H), 4.28 (q, $J$ = 7.1 Hz, 2H), 3.12 (dd, $J$ = 13.2, 5.1 Hz, 1H), 2.84 (t, $J$ = 12.3 Hz, 1H), 1.34 (t, $J$ = 7.1 Hz, 3H), 1.29 (t, $J$ = 7.1 Hz, 3H).

**$^{13}$C{$^1$H} NMR (101 MHz, CDCl$_3$) $\delta$ (ppm):** 166.7, 166.6, 137.0, 132.2, 129.6, 122.5, 70.2, 63.2, 63.0, 50.1, 45.3, 14.1.

**HRMS** (ESI) *m/z* calcd for $C_{16}H_{19}BrNO_4S$ $[M+H]^+$: 400.0218; found: 400.0204.

**Diethyl 2-imino-5-(naphthalen-2-yl)dihydrothiophene-3,3(2*H*)-dicarboxylate (3j):**

**Yield:** 58% (43 mg).

**Nature:** Colourless oil.

**$R_f$ value** = 0.33 [EtOAc:Petroleum ether = 1:4 (v/v)].

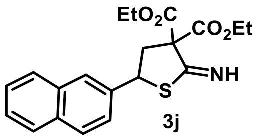

3j

**$^1$H NMR (400 MHz, $CDCl_3$) *δ* (ppm):** 7.88 (d, *J* = 1.2 Hz, 1H), 7.84 – 7.80 (m, 3H), 7.57 (dd, *J* = 8.5, 1.9 Hz, 1H), 7.51 – 7.49 (m, 2H), 5.04 (dd, *J* = 11.4, 5.0 Hz, 1H), 4.37 (q, *J* = 7.1 Hz, 2H), 4.28 (q, *J* = 7.1 Hz, 2H), 3.21 (dd, *J* = 13.3, 5.1 Hz, 1H), 3.03 (dd, *J* = 13.2, 11.5 Hz, 1H), 1.35 (t, *J* = 7.1 Hz, 3H), 1.29 (t, *J* = 7.1 Hz, 3H).

**$^{13}$C{$^1$H} NMR (101 MHz, $CDCl_3$) *δ* (ppm):** 178.4, 166.7, 166.5, 134.9, 133.3, 133.2, 129.0, 127.9, 127.8, 127.1, 126.7, 126.6, 125.1, 70.3, 63.2, 63.0, 51.2, 45.1, 14.1, 14.0.

**HRMS** (ESI) *m/z* calcd for $C_{20}H_{22}NO_4S$ $[M+H]^+$: 372.1270; found: 372.1274.

**Diethyl 2-imino-5-mesityldihydrothiophene-3,3(2*H*)-dicarboxylate (3k):**

**Yield:** 84% (61 mg).

**Nature:** Colourless oil.

**$R_f$ value** = 0.35 [EtOAc:Petroleum ether = 1:4 (v/v)].

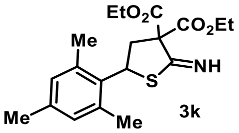

3k

**$^1$H NMR (400 MHz, DMSO-$d_6$) *δ* (ppm):** 10.97 (brs, 1H), 6.86 (s, 2H), 5.21 (d, *J* = 7.3 Hz, 1H), 4.32 – 4.24 (m, 2H), 4.21 – 4.16 (m, 2H), 3.18 (t, *J* = 12.5 Hz, 1H), 2.79 (dd, *J* = 13.3, 5.2 Hz, 1H), 2.39 (s, 6H), 2.19 (s, 3H), 1.25 (t, *J* = 7.1 Hz, 3H), 1.19 (t, *J* = 7.1 Hz, 3H).

**$^{13}$C{$^1$H} NMR (101 MHz, DMSO-$d_6$) *δ* (ppm):** 172.6, 167.0, 137.2, 137.1, 130.5, 128.9, 70.5, 62.1, 62.0, 45.4, 40.1, 20.6, 20.4, 13.9, 13.8.

**HRMS** (ESI) *m/z* calcd for $C_{19}H_{25}NO_4SK$ $[M+K]^+$: 402.1141; found: 402.1126.

**Diethyl 5-(furan-3-yl)-2-iminodihydrothiophene-3,3(2*H*)-dicarboxylate (3l):**

**Yield:** 80% (50 mg).

**Nature:** Colourless oil.

**$R_f$ value** = 0.34 [EtOAc:Petroleum ether = 1:4 (v/v)].

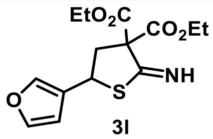

3l

**$^1$H NMR (400 MHz, $CDCl_3$) *δ* (ppm):** 9.64 (brs, 1H), 7.42 – 7.40 (m, 2H), 6.45 (s, 1H), 4.78 (dd, *J* = 10.9, 5.1 Hz, 1H), 4.31 (q, *J* = 7.1 Hz, 2H), 4.25 (q, *J* = 7.1 Hz, 2H), 3.09 (dd, *J* = 13.2, 5.1 Hz, 1H), 2.90 – 2.81 (m, 1H), 1.31 (t, *J* = 7.1 Hz, 3H), 1.28 (t, *J* = 7.1 Hz, 3H).

**$^{13}$C{$^1$H} NMR (101 MHz, $CDCl_3$) *δ* (ppm):** 177.0, 166.8, 144.1, 140.3, 123.4, 109.3, 70.0, 63.0, 62.9, 44.3, 41.1, 14.1, 14.0.

**HRMS** (ESI) *m/z* calcd for $C_{14}H_{18}NO_5S$ $[M+H]^+$: 312.0906; found: 312.0914.

**Diethyl 2-imino-5-(thiophen-3-yl)dihydrothiophene-3,3(2*H*)-dicarboxylate (3m):**

**Yield:** 81% (53 mg).

**Nature:** Colourless oil.

**$R_f$ value** = 0.34 [EtOAc:Petroleum ether = 1:4 (v/v)].

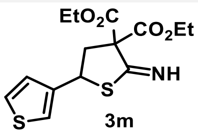

3m

**$^{1}$H NMR (400 MHz, DMSO-d$_6$) *δ* (ppm):** 10.88 (brs, 1H), 7.56 (d, *J* = 3.6 Hz, 2H), 7.18 (s, 1H), 4.94 (dd, *J* = 10.4, 4.4 Hz, 1H), 4.25 (q, *J* = 7.1 Hz, 2H), 4.14 – 4.12 (m, 2H), 2.98 (dd, *J* = 13.0, 5.0 Hz, 1H), 2.86 (dd, *J* = 12.9, 11.0 Hz, 1H), 1.23 (t, *J* = 7.1 Hz, 3H), 1.17 (t, *J* = 7.1 Hz, 3H).

**$^{13}$C{$^{1}$H} NMR (101 MHz, DMSO-d$_6$) *δ* (ppm):** 171.5, 167.1, 166.9, 139.2, 127.4, 126.8, 123.5, 70.4, 62.0, 61.9, 45.2, 43.6, 13.9, 13.8.

**HRMS** (ESI) *m/z* calcd for $C_{14}H_{18}NO_4S_2$ [M+H]$^+$: 328.0677; found: 328.0663.

**Diethyl 5-(1-(*tert*-butoxycarbonyl)-1*H*-indol-3-yl)-2-iminodihydrothiophene-3,3(2*H*)-dicarboxylate (3n):**

**Yield:** 38% (35 mg).

**Nature:** Colourless oil.

**R$_f$ value** = 0.38 [EtOAc:Petroleum ether = 3:7 (v/v)].

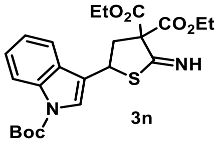

3n

**$^{1}$H NMR (400 MHz, CDCl$_3$) *δ* (ppm):** 8.15 (d, *J* = 7.9 Hz, 1H), 7.64 – 7.63 (m, 2H), 7.37 – 7.33 (m, 1H), 7.28 – 7.25 (m, 1H), 5.09 (dd, *J* = 10.8, 5.1 Hz, 1H), 4.39 – 4.33 (m, 2H), 4.27 (q, *J* = 7.1 Hz, 2H), 3.24 (dd, *J* = 13.1, 5.2 Hz, 1H), 3.15 (t, *J* = 12.0 Hz, 1H), 1.67 (s, 9H), 1.35 (t, *J* = 7.1 Hz, 3H), 1.28 (t, *J* = 7.1 Hz, 3H).

**$^{13}$C{$^{1}$H} NMR (101 MHz, CDCl$_3$) *δ* (ppm):** 176.9, 167.0, 166.8, 149.5, 136.0, 128.7, 125.2, 123.8, 122.9, 119.4, 117.9, 115.7, 84.3, 70.1, 63.1, 62.9, 42.9, 28.3, 14.1, 14.0.

**HRMS** (ESI) *m/z* calcd for $C_{23}H_{29}N_2O_6S$ [M+H]$^+$: 461.1746; found: 461.1739.

**Diethyl 2-imino-5-(4-methylthiazol-5-yl)dihydrothiophene-3,3(2*H*)-dicarboxylate (3o):**

**Yield:** 35% (24 mg).

**Nature:** Colourless oil.

**R$_f$ value** = 0.31 [EtOAc:Petroleum ether = 3:7 (v/v)].

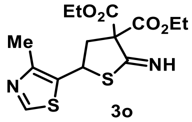

3o

**$^{1}$H NMR (400 MHz, CDCl$_3$) *δ* (ppm):** 8.68 (s, 1H), 5.14 (dd, *J* = 11.5, 5.0 Hz, 1H), 4.38 – 4.31 (m, 2H), 4.29 (q, *J* = 7.1 Hz, 2H), 3.21 (dd, *J* = 13.1, 5.0 Hz, 1H), 2.82 (t, *J* = 12.2 Hz, 1H), 2.44 (s, 3H), 1.34 (t, *J* = 7.1 Hz, 3H), 1.30 (t, *J* = 7.1 Hz, 3H).

**$^{13}$C{$^{1}$H} NMR (101 MHz, CDCl$_3$) *δ* (ppm):** 165.4, 164.9, 151.8, 151.5, 130.0, 73.7, 63.5, 63.3, 44.0, 42.6, 15.5, 14.1.

**HRMS** (ESI) *m/z* calcd for $C_{14}H_{19}N_2O_4S_2$ [M+H]$^+$: 343.0786; found: 343.0771.

**Diethyl 2-imino-5-methyl-5-phenyldihydrothiophene-3,3(2*H*)-dicarboxylate (3p):**

**Yield:** 64% (43 mg).

**Nature:** Colourless oil.

**R$_f$ value** = 0.38 [EtOAc:Petroleum ether = 1:4 (v/v)].

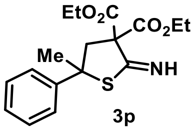

3p

**$^{1}$H NMR (400 MHz, CDCl$_3$) *δ* (ppm):** 7.54 – 7.51 (m, 2H), 7.35 – 7.31 (m, 2H), 7.27 – 7.23 (m, 1H), 4.36 – 4.26 (m, 2H), 3.82 – 3.76 (m, 2H), 3.42 (d, *J* = 13.6 Hz, 1H), 3.21 (d, *J* = 13.6 Hz, 1H), 1.84 (s, 3H), 1.31 (t, *J* = 7.1 Hz, 3H), 1.08 (t, *J* = 7.1 Hz, 3H).

**$^{13}$C{$^{1}$H} NMR (101 MHz, CDCl$_3$) *δ* (ppm):** 177.9, 167.2, 166.5, 143.9, 128.5, 127.6, 126.5, 70.5, 63.2, 62.8, 60.3, 50.3, 33.0, 14.1, 13.8.

**HRMS** (ESI) *m/z* calcd for $C_{17}H_{22}NO_4S$ [M+H]$^+$: 336.1264; found: 336.1262.

**Diethyl 2-imino-4-methyl-5-phenyldihydrothiophene-3,3(2*H*)-dicarboxylate (3q):**

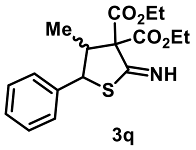

3q

**Yield:** 58% (39 mg, combined yield as a 1.4:1 mixture of diastereomers).

**Nature:** Colourless oil.

**$R_f$ value** = 0.32 [EtOAc:Petroleum ether = 1:4 (v/v)].

**$^1$H NMR (400 MHz, $CDCl_3$) *δ* (ppm):** (for the major isomer) 9.91 (brs, 1H), 7.43 – 7.30 (m, 5H), 4.45 (d, *J* = 11.2 Hz, 1H), 4.40 – 4.18 (m, 4H), 3.31 – 3.23 (m, 1H), 1.35 (t, *J* = 7.1 Hz, 6H), 1.01 (d, *J* = 6.8 Hz, 3H); (for the minor isomer) 9.91 (brs, 1H), 7.43 – 7.30 (m, 5H), 5.47 (d, *J* = 5.2 Hz, 1H), 4.40 – 4.18 (m, 4H), 3.41 – 3.35 (m, 1H), 1.30 (t, *J* = 7.1 Hz, 3H), 1.26 (t, *J* = 7.1 Hz, 3H), 0.74 (d, *J* = 7.1 Hz, 3H).

**$^{13}$C{$^1$H} NMR (101 MHz, $CDCl_3$) *δ* (ppm):** (for the mixture) 181.2, 166.7, 166.6, 166.4, 166.3, 136.7, 135.9, 129.0, 128.7, 128.6, 128.5, 128.1, 72.9, 63.0, 62.8, 62.5, 62.3, 51.3, 46.5, 14.3, 14.2, 14.1, 14.0, 13.3, 11.0.

**HRMS** (ESI) *m/z* calcd for $C_{17}H_{22}NO_4S$ $[M+H]^+$: 336.1270; found: 336.1257.

**Diethyl (3a*R*,8b*S*)-2-imino-4,8b-dihydro-2*H*-indeno[1,2-*b*]thiophene-3,3(3a*H*)-dicarboxylate (3r):**

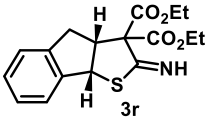

3r

**Yield:** 84% (56 mg).

**Nature:** Colourless oil.

**$R_f$ value** = 0.31 [EtOAc:Petroleum ether = 1:4 (v/v)].

**$^1$H NMR (400 MHz, $CDCl_3$) *δ* (ppm):** 10.04 (brs, 1H), 7.26 – 7.17 (m, 4H), 5.25 (d, *J* = 6.3 Hz, 1H), 4.35 – 4.23 (m, 4H), 3.91 – 3.85 (m, 1H), 3.00 (d, *J* = 7.9 Hz, 2H), 1.32 (t, *J* = 7.1 Hz, 3H), 1.29 (t, *J* = 7.1 Hz, 3H).

**$^{13}$C{$^1$H} NMR (101 MHz, $CDCl_3$) *δ* (ppm):** 166.5, 142.0, 141.5, 128.5, 127.3, 125.1, 124.6, 70.7, 63.0, 62.4, 52.4, 49.5, 33.6, 14.1, 13.9.

**HRMS** (ESI) *m/z* calcd for $C_{17}H_{20}NO_4S$ $[M+H]^+$: 334.1113; found: 334.1106.

**Diethyl 5-decyl-2-iminodihydrothiophene-3,3(2*H*)-dicarboxylate (3s):**

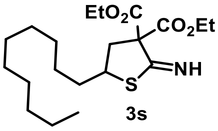

3s

**Yield:** 83% (64 mg).

**Nature:** Colourless oil.

**$R_f$ value** = 0.37 [EtOAc:Petroleum ether = 1:4 (v/v)].

**$^1$H NMR (400 MHz, $CDCl_3$) *δ* (ppm):** 9.87 (brs, 1H), 4.31 – 4.24 (m, 4H), 3.72 – 3.63 (m, 1H), 2.96 – 2.85 (m, 1H), 2.47 (dd, *J* = 13.0, 10.8 Hz, 1H), 1.83 – 1.70 (m, 1H), 1.68 – 1.60 (m, 1H), 1.31 – 1.22 (m, 16H), 1.30 (t, *J* = 7.1 Hz, 3H), 1.29 (t, *J* = 7.1 Hz, 3H), 0.87 (t, *J* = 6.8 Hz, 3H).

**$^{13}$C{$^1$H} NMR (101 MHz, $CDCl_3$) *δ* (ppm):** 177.6, 167.3, 167.1, 70.1, 62.8, 62.7, 47.8, 43.2, 36.0, 32.0, 29.7, 29.6, 29.5, 29.4, 29.3, 28.6, 22.8, 14.3, 14.1, 14.0.

**HRMS** (ESI) *m/z* calcd for $C_{20}H_{36}NO_4S$ $[M+H]^+$: 386.2365; found: 386.2369.

**Diethyl 2-imino-5-(9-methoxy-9-oxononyl)dihydrothiophene-3,3(2*H*)-dicarboxylate (3t):**

**Yield:** 82% (68 mg).

**Nature:** Colourless oil.

**$R_f$ value** = 0.32 [EtOAc:Petroleum ether = 1:4 (v/v)].

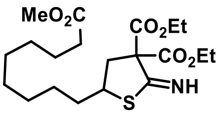


3t

**$^1$H NMR (400 MHz, $CDCl_3$) *δ* (ppm):** 9.52 (brs, 1H), 4.34 – 4.24 (m, 4H), 3.68 – 3.62 (m, 1H), 3.65 (s, 3H), 2.92 (dd, *J* = 13.0, 4.9 Hz, 1H), 2.46 (t, *J* = 11.8 Hz, 1H), 2.29 (t, *J* = 7.5 Hz, 2H), 1.76 – 1.71 (m, 1H), 1.61 (dd, *J* = 16.4, 10.4 Hz, 3H), 1.31 – 1.27 (m, 16H).

**$^{13}$C{$^1$H} NMR (101 MHz, $CDCl_3$) *δ* (ppm):** 177.5, 174.4, 167.2, 62.8, 62.7, 51.6, 43.1, 35.9, 34.2, 29.4, 29.3, 29.2, 29.1, 28.6, 25.0, 14.1, 14.0.

**HRMS** (ESI) *m/z* calcd for $C_{20}H_{34}NO_6S$ $[M+H]^+$: 416.2107; found: 416.2098.

**Diethyl 2-imino-5-(3-oxobutyl)dihydrothiophene-3,3(2*H*)-dicarboxylate (3u):**

**Yield:** 79% (50 mg).

**Nature:** Colourless oil.

**$R_f$ value** = 0.36 [EtOAc:Petroleum ether = 2:3 (v/v)].

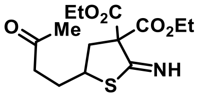


3u

**$^1$H NMR (400 MHz, $CDCl_3$) *δ* (ppm):** 4.26 (q, *J* = 7.1 Hz, 2H), 4.25 (q, *J* = 7.1 Hz, 2H), 3.73 – 3.65 (m, 1H), 2.92 (dd, *J* = 13.1, 5.1 Hz, 1H), 2.52 (t, *J* = 7.4 Hz, 2H), 2.50 – 2.44 (m, 1H), 2.16 – 2.05 (m, 1H), 2.14 (s, 3H), 1.91 – 1.82 (m, 1H), 1.27 (t, *J* = 7.1 Hz, 6H).

**$^{13}$C{$^1$H} NMR (101 MHz, $CDCl_3$) *δ* (ppm):** 206.9, 176.9, 167.0, 166.8, 69.9, 62.9, 62.8, 46.6, 42.8, 41.6, 30.1, 29.3, 14.1, 14.0.

**HRMS** (ESI) *m/z* calcd for $C_{14}H_{21}NO_5SNa$ $[M+Na]^+$: 338.1038; found: 338.1014.

**Diethyl 5-(3-hydroxypropyl)-2-iminodihydrothiophene-3,3(2*H*)-dicarboxylate (3v):**

**Yield:** 84% (51 mg).

**Nature:** Colourless oil.

**$R_f$ value** = 0.33 [EtOAc:Petroleum ether = 3:2 (v/v)].

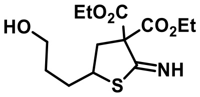


3v

**$^1$H NMR (400 MHz, $CDCl_3$) *δ* (ppm):** 4.31 – 4.25 (m, 4H), 3.76 – 3.69 (m, 1H), 3.66 (t, *J* = 6.1 Hz, 2H), 2.97 (dd, *J* = 13.1, 5.0 Hz, 1H), 2.50 (dd, *J* = 12.7, 11.1 Hz, 1H), 1.96 – 1.87 (m, 1H), 1.80 – 1.71 (m, 1H), 1.70 – 1.60 (m, 2H), 1.30 (t, *J* = 7.1 Hz, 3H), 1.29 (t, *J* = 7.1 Hz, 3H).

**$^{13}$C{$^1$H} NMR (101 MHz, $CDCl_3$) *δ* (ppm):** 167.1, 166.8, 70.0, 63.0, 62.9, 62.2, 47.6, 43.0, 32.3, 31.4, 14.1, 14.0.

**HRMS** (ESI) *m/z* calcd for $C_{13}H_{22}NO_5S$ $[M+H]^+$: 304.1219; found: 304.1211.

**Diethyl 5-(3-bromopropyl)-2-iminodihydrothiophene-3,3(2*H*)-dicarboxylate (3w):**

**Yield:** 63% (46 mg).

**Nature:** Colourless oil.

**$R_f$ value** = 0.31 [EtOAc:Petroleum ether = 2:3 (v/v)].

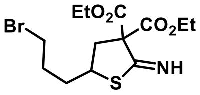


3w

**$^1$H NMR (400 MHz, $CDCl_3$) *δ* (ppm):** 4.31 – 4.24 (m, 4H), 3.73 – 3.66 (m, 1H), 3.40 (t, *J* = 5.8 Hz, 2H), 2.96 (dd, *J* = 13.1, 5.0 Hz, 1H), 2.49 (dd, *J* = 13.1, 10.6 Hz, 1H), 2.01 – 1.89 (m, 3H), 1.84 – 1.75 (m, 1H), 1.29 (t, *J* = 7.1 Hz, 3H), 1.28 (t, *J* = 7.1 Hz, 3H).

**$^{13}C\{^1H\}$ NMR (101 MHz, $CDCl_3$) $\delta$ (ppm):** 177.3, 167.0, 166.8, 69.9, 63.0, 62.9, 46.8, 42.9, 34.4, 32.8, 31.3,14.1, 14.0.

**HRMS** (ESI) *m/z* calcd for $C_{13}H_{21}BrNO_4S$ $[M+H]^+$: 366.0375; found: 366.0372.

**Diethyl 2-imino-5-(3-((methylsulfonyl)oxy)propyl)dihydrothiophene-3,3(2*H*)-dicarboxylate (3x):**

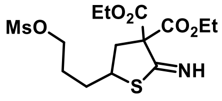

3x

**Yield:** 80% (61 mg).

**Nature:** Colourless oil.

**$R_f$ value** = 0.34 [EtOAc:Petroleum ether = 2:3 (v/v)].

**$^1H$ NMR (400 MHz, $CDCl_3$) $\delta$ (ppm):** 4.31 – 4.25 (m, 2H), 4.28 (q, *J* = 7.1 Hz, 2H), 4.23 (t, *J* = 6.1 Hz, 2H), 3.75 – 3.67 (m, 1H), 3.04 – 2.94 (m, 1H), 3.02 (s, 3H), 2.49 (dd, *J* = 13.1, 10.6 Hz, 1H), 1.96 – 1.91 (m, 1H), 1.87 – 1.74 (m, 3H), 1.29 (t, *J* = 7.1 Hz, 3H), 1.28 (t, *J* = 7.1 Hz, 3H).

**$^{13}C\{^1H\}$ NMR (101 MHz, $CDCl_3$) $\delta$ (ppm):** 176.9, 166.9, 166.7, 69.9, 68.9, 63.0, 62.9, 46.7, 42.8, 37.6, 31.9, 28.1, 14.0.

**HRMS** (ESI) *m/z* calcd for $C_{14}H_{24}NO_7S_2$ $[M+H]^+$: 382.0994; found: 382.0984.

**Diethyl 2-imino-1-thiaspiro[4.5]decane-3,3-dicarboxylate (3y):**

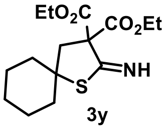

3y

**Yield:** 62% (39 mg).

**Nature:** Colourless oil.

**$R_f$ value** = 0.34 [EtOAc:Petroleum ether = 1:4 (v/v)].

**$^1H$ NMR (400 MHz, $CDCl_3$) $\delta$ (ppm):** 4.32 – 4.23 (m, 4H), 2.85 (s, 2H), 1.84 (dd, *J* = 15.2, 6.2 Hz, 2H), 1.69 – 1.56 (m, 6H), 1.46 – 1.37 (m, 2H), 1.29 (t, *J* = 7.1 Hz, 6H).

**$^{13}C\{^1H\}$ NMR (101 MHz, $CDCl_3$) $\delta$ (ppm):** 177.5, 167.9, 69.8, 62.9, 59.7, 48.7, 40.2, 25.2, 24.5, 14.1.

**HRMS** (ESI) *m/z* calcd for $C_{15}H_{24}NO_4S$ $[M+H]^+$: 314.1426; found: 314.1420.

**Diethyl 5-benzyl-2-imino-5-methyldihydrothiophene-3,3(2*H*)-dicarboxylate (3z):**

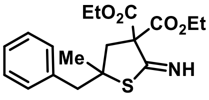

3z

**Yield:** 72% (50 mg).

**Nature:** Colourless oil.

**$R_f$ value** = 0.32 [EtOAc:Petroleum ether = 1:4 (v/v)].

**$^1H$ NMR (400 MHz, $CDCl_3$) $\delta$ (ppm):** 7.32 – 7.26 (m, 3H), 7.23 – 7.21 (m, 2H), 4.34 – 4.28 (m, 2H), 4.28 (q, *J* = 7.1 Hz, 2H) 3.06 (d, *J* = 13.6, 1H), 3.03 (d, *J* = 13.7, 1H), 2.96 (d, *J* = 13.5 Hz, 1H), 2.82 (d, *J* = 13.7 Hz, 1H), 1.38 (s, 3H), 1.31 (t, *J* = 7.1 Hz, 3H), 1.29 (t, *J* = 7.1 Hz, 3H).

**$^{13}C\{^1H\}$ NMR (101 MHz, $CDCl_3$) $\delta$ (ppm):** 177.3, 167.9, 167.7, 136.8, 130.4, 128.3, 127.1 70.5, 63.0, 62.9, 57.4, 49.3, 48.7, 28.6, 14.0, 13.9.

**HRMS** (ESI) *m/z* calcd for $C_{18}H_{24}NO_4S$ $[M+H]^+$: 350.1426; found: 350.1409.

**Diethyl 5-(2-((*tert*-butyldimethylsilyl)oxy)ethyl)-2-imino-5-methyldihydrothiophene-3,3(2*H*)-dicarboxylate (3aa):**

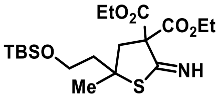

3aa

**Yield:** 67% (56 mg).

**Nature:** Colourless oil.

**$R_f$ value** = 0.32 [EtOAc:Petroleum ether = 3:7 (v/v)].

$^{1}$**H NMR (400 MHz, $CDCl_3$) δ (ppm):** 9.49 (brs, 1H), 4.35 – 4.26 (m, 4H), 3.76 – 3.72 (m, 2H), 2.97 (d, *J* = 13.7 Hz, 1H), 2.82 (d, *J* = 13.7 Hz, 1H), 2.04 – 1.91 (m, 2H), 1.47 (s, 3H), 1.31 (t, *J* = 7.1 Hz, 3H), 1.29 (t, *J* = 7.1 Hz, 3H), 0.89 (s, 9H), 0.05 (s, 6H).

$^{13}$**C{$^{1}$H} NMR (101 MHz, $CDCl_3$) δ (ppm):** 168.1, 167.7, 69.9, 64.0, 63.0, 62.9, 60.0, 49.7, 45.8, 28.4, 26.0, 18.3, 14.1, -5.3.

**HRMS** (ESI) *m/z* calcd for $C_{19}H_{36}NO_5SSi$ $[M+H]^+$: 418.2083; found: 418.2081.

**Diethyl 2-iminohexahydrobenzo[*b*]thiophene-3,3(2*H*)-dicarboxylate (3ab):**

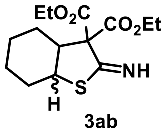

**3ab**

**Yield:** 65% (39 mg, combined yield as a 4:1 mixture of diastereomers).

**Nature:** Colourless oil.

**$R_f$ value** = 0.40 [EtOAc:Petroleum ether = 1:4 (v/v)].

$^{1}$**H NMR (400 MHz, $CDCl_3$) δ (ppm):** (for the mixture) 9.64 (brs, 1H), 4.40 – 4.16 (m, 4H), 3.71 – 3.32 (m, 1H), 3.02 – 2.66 (m, 1H), 2.28 – 2.14 (m, 1H), 2.03 – 1.99 (m, 1H), 1.92 – 1.76 (m, 2H), 1.64 – 1.39 (m, 4H), 1.35 – 1.23 (m, 6H).

$^{13}$**C{$^{1}$H} NMR (101 MHz, $CDCl_3$) δ (ppm):** (for the mixture) 178.2, 166.9, 166.7, 166.4, 166.3, 71.5, 62.8, 62.7, 62.3, 62.2, 55.2, 49.3, 46.6, 31.8, 29.8, 27.9, 26.6, 25.8, 25.7, 24.6, 22.6, 19.2, 14.3, 14.2, 14.0.

**HRMS** (ESI) *m/z* calcd for $C_{14}H_{22}NO_4S$ $[M+H]^+$: 300.1270; found: 300.1261.

**Diethyl (3a*R*,4*S*,7*R*,7a*S*)-2-iminohexahydro-4,7-methanobenzo[b]thiophene-3,3(2*H*)-dicarboxylate (3ac):**

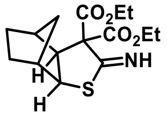

**3ac**

**Yield:** 53% (33 mg).

**Nature:** Colourless oil.

**$R_f$ value** = 0.39 [EtOAc:Petroleum ether = 1:4 (v/v)].

$^{1}$**H NMR (400 MHz, $CDCl_3$) δ (ppm):** 4.33 – 4.12 (m, 4H), 3.69 (dd, *J* = 7.4, 0.9 Hz, 1H), 3.20 (dd, *J* = 7.5, 1.0 Hz, 1H), 2.34 (d, *J* = 4.3 Hz, 1H), 1.91 (d, *J* = 3.5 Hz, 1H), 1.80 (d, *J* = 11.0 Hz, 1H), 1.68 – 1.60 (m, 1H), 1.59 – 1.51 (m, 1H), 1.30 – 1.20 (m, 1H), 1.27 (t, *J* = 7.1 Hz, 3H), 1.22 (t, *J* = 7.1 Hz, 3H), 1.17 – 1.13 (m, 2H).

$^{13}$**C{$^{1}$H} NMR (101 MHz, $CDCl_3$) δ (ppm):** 179.2, 166.8, 166.6, 72.3, 63.1, 62.1, 54.0, 53.1, 44.5, 39.5, 33.3, 30.2, 27.2, 14.1, 13.8.

**HRMS** (ESI) *m/z* calcd for $C_{15}H_{22}NO_4S$ $[M+H]^+$: 312.1270; found: 312.1255.

**2-(*tert*-Butyl) 4,4-diethyl 5-iminodihydrothiophene-2,4,4(5*H*)-tricarboxylate (3ad):**

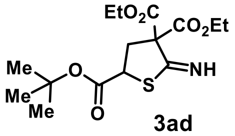

**3ad**

**Yield:** 39% (27 mg).

**Nature:** Colourless oil.

**$R_f$ value** = 0.42 [EtOAc:Petroleum ether = 3:7 (v/v)].

$^{1}$**H NMR (400 MHz, $CDCl_3$) δ (ppm):** 4.31 – 4.24 (m, 3H), 4.27 (q, *J* = 7.1 Hz, 2H), 3.05 (dd, *J* = 13.5, 6.3 Hz, 1H), 2.97 (dd, *J* = 13.5, 8.8 Hz, 1H), 1.45 (s, 9H), 1.29 (t, *J* = 7.1 Hz, 3H), 1.28 (t, *J* = 7.1 Hz, 3H).

**$^{13}C\{^1H\}$ NMR (101 MHz, $CDCl_3$) $\delta$ (ppm):** 176.1, 168.7, 166.6, 166.5, 83.2, 69.0, 63.1, 62.9, 47.7, 38.3, 28.0, 14.0.

**HRMS** (ESI) *m/z* calcd for $C_{15}H_{24}NO_6S$ $[M+H]^+$: 346.1324; found: 346.1335.

**4,4-Diethyl 2-phenyl 5-iminodihydrothiophene-2,4,4(5*H*)-tricarboxylate (3ae):**

**Yield:** 41% (30 mg).

**Nature:** Colourless oil.

**$R_f$ value** = 0.40 [EtOAc:Petroleum ether = 3:7 (v/v)].

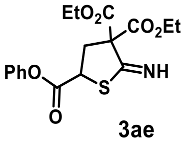


**3ae**

**$^1H$ NMR (400 MHz, $CDCl_3$) $\delta$ (ppm):** 7.41 – 7.37 (m, 2H), 7.26 (t, *J* = 7.6 Hz, 1H), 7.11 (d, *J* = 7.6 Hz, 2H), 4.61 (t, *J* = 7.0 Hz, 1H), 4.36 – 4.29 (m, 2H), 4.27 (q, *J* = 7.1 Hz, 2H), 3.29 – 3.17 (m, 2H), 1.32 (t, *J* = 7.1 Hz, 3H), 1.27 (d, *J* = 7.1 Hz, 3H).

**$^{13}C\{^1H\}$ NMR (101 MHz, $CDCl_3$) $\delta$ (ppm):** 168.6, 165.8, 165.7, 150.5, 129.7, 126.5, 121.3, 63.3, 63.1, 55.6, 48.4, 38.3, 14.1, 14.0.

**HRMS** (ESI) *m/z* calcd for $C_{17}H_{20}NO_6S$ $[M+H]^+$: 366.1011; found: 366.1014.

**4,4-Diethyl 2-methyl 5-imino-2-methyldihydrothiophene-2,4,4(5*H*)-tricarboxylate (3af):**

**Yield:** 47% (30 mg).

**Nature:** Colourless oil.

**$R_f$ value** = 0.43 [EtOAc:Petroleum ether = 3:7 (v/v)].

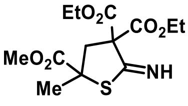


**3af**

**$^1H$ NMR (400 MHz, $CDCl_3$) $\delta$ (ppm):** 4.35 – 4.29 (m, 2H), 4.27 (q, *J* = 7.1 Hz, 2H), 3.73 (s, 3H), 3.41 (d, *J* = 14.1 Hz, 1H), 2.94 (d, *J* = 14.1 Hz, 1H), 1.74 (s, 3H), 1.31 (t, *J* = 7.1 Hz, 3H), 1.30 (d, *J* = 7.1 Hz, 3H).

**$^{13}C\{^1H\}$ NMR (101 MHz, $CDCl_3$) $\delta$ (ppm):** 173.4, 167.0, 70.1, 63.2, 62.9, 56.7, 53.3, 45.8, 26.8, 14.1, 14.0.

**HRMS** (ESI) *m/z* calcd for $C_{13}H_{20}NO_6S$ $[M+H]^+$: 318.1011; found: 318.1014.

**2-Benzyl 4,4-diethyl 5-imino-2-methyldihydrothiophene-2,4,4(5*H*)-tricarboxylate (3ag):**

**Yield:** 42% (33 mg).

**Nature:** Colourless oil.

**$R_f$ value** = 0.38 [EtOAc:Petroleum ether = 3:7 (v/v)].

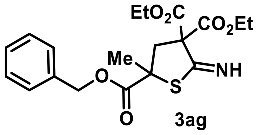


**3ag**

**$^1H$ NMR (400 MHz, $CDCl_3$) $\delta$ (ppm):** 7.39 – 7.31 (m, 5H), 5.14 (s, 2H), 4.36 – 4.26 (m, 2H), 4.19 (q, *J* = 7.1 Hz, 2H), 3.44 (d, *J* = 14.0 Hz, 1H), 2.94 (d, *J* = 14.1 Hz, 1H), 1.74 (s, 3H), 1.30 (t, *J* = 7.1 Hz, 3H), 1.26 (t, *J* = 7.1 Hz, 3H).

**$^{13}C\{^1H\}$ NMR (101 MHz, $CDCl_3$) $\delta$ (ppm):** 172.7, 167.0, 166.9, 135.3, 128.8, 128.6, 128.1, 70.1, 67.9, 63.2, 62.9, 57.0, 45.7, 26.8, 14.1, 14.0.

**HRMS** (ESI) *m/z* calcd for $C_{19}H_{24}NO_6S$ $[M+H]^+$: 394.1324; found: 394.1316.

**Dimethyl 2-imino-5-phenyldihydrothiophene-3,3(2*H*)-dicarboxylate (3ah):**

**Yield:** 82% (48 mg).

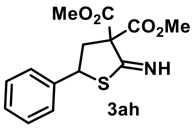

3ah

**Nature:** Colourless oil.

**$R_f$ value** = 0.30 [EtOAc:Petroleum ether = 1:4 (v/v)].

**$^1$H NMR (400 MHz, DMSO-d$_6$) *δ* (ppm):** 10.95 (brs, 1H), 7.46 (d, *J* = 7.3 Hz, 2H), 7.40 – 7.36 (m, 2H), 7.32 (t, *J* = 7.2 Hz, 1H), 4.88 (dd, *J* = 11.3, 4.9 Hz, 1H), 3.80 (s, 3H), 3.69 (s, 3H), 2.98 (dd, *J* = 13.0, 5.0 Hz, 1H), 2.84 (t, *J* = 12.8 Hz, 1H).

**$^{13}$C{$^1$H} NMR (101 MHz, DMSO-d$_6$) *δ* (ppm):** 171.5, 167.5, 167.4, 138.2, 128.8, 128.2, 127.6, 70.7, 53.5, 53.2, 49.8, 44.4.

**HRMS** (ESI) *m/z* calcd for $C_{14}H_{16}NO_4S$ [M+H]$^+$: 294.0800; found: 294.0801.

**Diisopropyl 2-imino-5-phenyldihydrothiophene-3,3(2*H*)-dicarboxylate (3ai):**

**Yield:** 77% (54 mg).

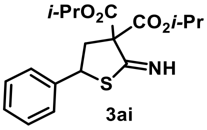

3ai

**Nature:** Colourless oil.

**$R_f$ value** = 0.32 [EtOAc:Petroleum ether = 1:4 (v/v)].

**$^1$H NMR (400 MHz, DMSO-d$_6$) *δ* (ppm):** 10.89 (brs, 1H), 7.46 (d, *J* = 7.3 Hz, 2H), 7.39 – 7.36 (m, 2H), 7.31 (t, *J* = 7.2 Hz, 1H), 5.09 – 5.03 (m, 1H), 4.93 – 4.90 (m, 1H), 4.82 (d, *J* = 6.0 Hz, 1H), 2.94 (dd, *J* = 13.0, 5.0 Hz, 1H), 2.94 (t, *J* = 12.8, 1H), 1.25 (d, *J* = 6.3 Hz, 3H), 1.23 (d, *J* = 6.3 Hz, 3H), 1.19 (d, *J* = 6.2 Hz, 3H), 1.13 (d, *J* = 6.1 Hz, 3H).

**$^{13}$C{$^1$H} NMR (101 MHz, DMSO-d$_6$) *δ* (ppm):** 171.6, 166.5, 166.4, 138.3, 128.8, 128.2, 127.6, 70.4, 69.6, 49.7, 44.3, 21.4, 21.3, 21.2, 21.1.

**HRMS** (ESI) *m/z* calcd for $C_{18}H_{24}NO_4S$ [M+H]$^+$: 350.1426; found: 350.1422.

**Dibenzyl 2-imino-5-phenyldihydrothiophene-3,3(2*H*)-dicarboxylate (3aj):**

**Yield:** 65% (58 mg).

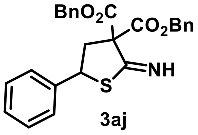

3aj

**Nature:** Colourless oil.

**$R_f$ value** = 0.31 [EtOAc:Petroleum ether = 1:4 (v/v)].

**$^1$H NMR (400 MHz, CDCl$_3$) *δ* (ppm):** 7.38 – 7.27 (m, 15H), 5.27 (d, *J* = 3.3 Hz, 2H), 5.20 (s, 2H), 4.74 (dd, *J* = 11.5, 5.0 Hz, 1H), 3.12 (dd, *J* = 13.2, 5.0 Hz, 1H), 2.93 (t, *J* = 12.4 Hz, 1H).

**$^{13}$C{$^1$H} NMR (101 MHz, CDCl$_3$) *δ* (ppm):** 166.6, 137.8, 135.1, 134.9, 129.0, 128.9, 128.8, 128.7, 128.6, 128.5, 128.3, 128.2, 127.8, 70.5, 68.5, 68.4, 50.8, 45.5.

**HRMS** (ESI) *m/z* calcd for $C_{26}H_{23}NO_4SNa$ [M+Na]$^+$: 468.1245; found: 468.1224.

**3-Benzyl 3-ethyl 2-imino-5-phenyldihydrothiophene-3,3(2*H*)-dicarboxylate (3ak):**

**Yield:** 76% (58 mg, combined yield as a 1:1 mixture of diastereomers with rotamers).

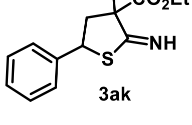

3ak

**Nature:** Colourless oil.

**$R_f$ value** = 0.30 [EtOAc:Petroleum ether = 1:4 (v/v)].

**$^1$H NMR (400 MHz, CDCl$_3$) *δ* (ppm):** (for the diastereomeric and rotameric mixture) 7.45 – 7.37 (m, 10H), 7.36 – 7.28 (m, 10H), 5.32 (d, *J* = 1.8 Hz, 2H), 5.24 (s, 2H), 4.85 (dd, *J* = 11.5, 5.0 Hz, 1H), 4.72 (dd, *J* = 11.6, 5.0 Hz, 1H), 4.36 – 4.26 (m, 2H), 4.22 (q, *J* = 7.1 Hz, 2H), 3.15 (dd, *J* = 13.2, 5.0 Hz, 1H), 3.10 (dd, *J* = 13.2, 4.9 Hz, 1H), 2.92 (t, *J* = 12.4 Hz, 1H), ), 2.91 (t, *J* = 12.3 Hz, 1H), (1.34

(t, $J$ = 7.1 Hz) rotameric peaks + 1.23 (t, $J$ = 6.8 Hz), 3H), (1.29 (t, $J$ = 7.1 Hz) rotameric peaks + 1.20 (t, $J$ = 6.8 Hz), 3H).

**$^{13}C\{^1H\}$ NMR (101 MHz, $CDCl_3$) $\delta$ (ppm):** (for the diastereomeric and rotameric mixture) 177.3, 177.2, 166.9, 166.8, 166.7, 166.6, 137.9+137.8 (*rotameric peaks*), 137.7, 135.2, 135.0, 129.0, 128.8, 128.7, 128.7, 128.6, 128.3, 127.9, 127.8, 70.5, 70.4, 68.4, 68.3, 63.2+63.1 (*rotameric peaks*), 63.0+62.9 (*rotameric peaks*), 50.8, 45.6, 45.4, 14.2+14.1 (*rotameric peaks*), 14.0+13.9 (*rotameric peaks*).

**HRMS** (ESI) *m/z* calcd for $C_{21}H_{21}NO_4SNa$ $[M+Na]^+$: 406.1089; found: 406.1075.

**Diethyl 2-imino-5-(4-((((8*S*,9*R*,13*R*,14*R*)-13-methyl-17-oxo-7,8,9,11,12,13,14,15,16,17-decahydro-6*H*-cyclopenta[*a*]phenanthren-3-yl)oxy)methyl)phenyl)dihydrothiophene-3,3(2*H*)-dicarboxylate (3al):**

**Yield:** 78% (94 mg, combined yield as a 2:1 mixture of diastereomers).

**Nature:** Colourless oil.

**$R_f$ value** = 0.30 [EtOAc:Petroleum ether = 2:3 (v/v)].

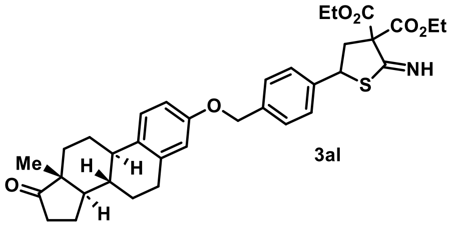


3al

**$^1H$ NMR (400 MHz, $CDCl_3$) $\delta$ (ppm):** (for the mixture) 7.46 – 7.39 (m, 3H), 7.24 – 7.17 (m, 2H), 6.85 – 6.78 (m, 1H), 6.76 – 6.69 (m, 1H), 5.02 (s, 2H), 4.91 – 4.82 (m, 1H), 4.35 (q, $J$ = 7.1 Hz, 1H), 4.28 (q, $J$ = 7.1 Hz, 1H), 4.23 – 3.94 (m, 2H), 3.16 – 2.99 (m, 1H), 2.90 – 2.81 (m, 3H), 2.54 – 2.47 (m, 1H), 2.40 – 2.37 (m, 1H), 2.27 – 2.22 (m, 1H), 2.12 – 2.02 (m, 2H), 1.99 – 1.94 (m, 2H), 1.65 – 1.53 (m, 3H), 1.51 – 1.41 (m, 3H), 1.36 – 1.09 (m, 6H), 0.90 (s, 3H).

**$^{13}C\{^1H\}$ NMR (101 MHz, $CDCl_3$) $\delta$ (ppm):** (for the mixture) 221.1, 169.6, 169.5, 169.3, 169.1, 156.8, 138.1, 138.0, 137.9, 137.8, 137.7, 137.6, 132.6, 132.5, 128.2, 128.1, 128.0, 126.5, 126.4, 115.0, 112.5, 112.4, 69.9, 69.5, 63.0, 62.9, 61.4, 61.2, 50.5, 48.1, 45.4, 44.1, 38.5, 36.0, 31.7, 29.8, 26.6, 26.0, 21.7, 14.2, 14.1, 14.0, 13.9.

**HRMS** (ESI) *m/z* calcd for $C_{35}H_{42}NO_6S$ $[M+H]^+$: 604.2733; found: 604.2735.

**Diethyl 2-imino-5-(3-(((*S*)-2-(6-methoxynaphthalen-2-yl)propanoyl)oxy)propyl)dihydrothiophene-3,3(2*H*)-dicarboxylate (3am):**

**Yield:** 71% (73 mg).

**Nature:** Colourless oil.

**$R_f$ value** = 0.32 [EtOAc:Petroleum ether = 3:7 (v/v)].

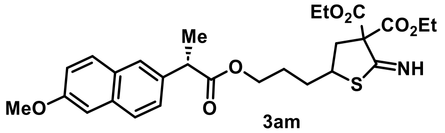


3am

**$^1H$ NMR (400 MHz, $CDCl_3$) $\delta$ (ppm):** 7.71 (d, $J$ = 8.4, Hz, 1H), 7.39 (d, $J$ = 8.5, Hz, 1H), 7.66 (s, 1H), 7.39 (d, $J$ = 8.4 Hz, 1H), 7.13 (d, $J$ = 8.8 Hz, 1H), 7.12 (s, 1H), 4.34 – 4.20 (m, 2H), 4.27 (q, $J$ = 7.1 Hz, 2H), 4.11 – 4.03 (m, 2H), 3.93 – 3.82 (m, 1H), 3.91 (s, 3H), 3.59 – 3.55 (m, 1H), 2.80 (dd, $J$ = 13.1, 5.0 Hz, 1H), 2.31 – 2.25 (m, 1H), 1.67 – 1.52 (m, 4H) 1.57 (d, $J$ = 7.1 Hz, 3H), 1.30 (t, $J$ = 7.1 Hz, 3H), 1.27 (t, $J$ = 7.1 Hz, 3H).

**$^{13}C\{^1H\}$ NMR (101 MHz, $CDCl_3$) $\delta$ (ppm):** 174.7, 157.8, 135.7, 133.8, 129.4, 129.0, 127.4, 126.3, 126.2, 126.1, 126.0, 119.2, 105.7, 63.9, 63.2, 63.1, 55.4, 45.5, 42.6, 32.1, 29.8, 27.6, 18.4, 14.1, 14.0.

**HRMS** (ESI) *m/z* calcd for $C_{27}H_{34}NO_7S$ $[M+H]^+$: 561.2056; found: 561.2052.

**Diethyl 5-(3-((3-(4,5-diphenyloxazol-2-yl)propanoyl)oxy)propyl)-2-iminodihydrothiophene-3,3(2*H*)-dicarboxylate (3an):**

**Yield:** 75% (87 mg).

**Nature:** Colourless oil.

**$R_f$ value** = 0.38 [EtOAc:Petroleum ether = 2:3 (v/v)].

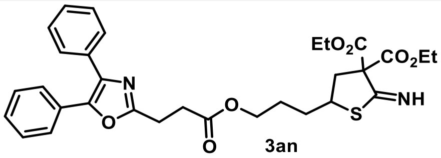


3an

**$^1$H NMR (400 MHz, $CDCl_3$) *δ* (ppm):** 7.63 – 7.61 (m, 2H), 7.58 – 7.55 (m, 2H), 7.38 – 7.29 (m, 6H), 4.31 – 4.10 (m, 2H), 4.28 (q, *J* = 7.1 Hz, 2H), 4.13 (t, *J* = 5.9 Hz, 2H), 3.72 – 3.57 (m, 1H), 3.18 (t, *J* = 7.4 Hz, 2H), 2.95 – 2.90 (m, 3H), 2.45 (dd, *J* = 13.0, 10.7 Hz, 1H), 1.86 – 1.69 (m, 4H), 1.29 (t, *J* = 7.1 Hz, 3H), 1.28 – 1.22 (m, 3H).

**$^{13}C\{^1H\}$ NMR (101 MHz, $CDCl_3$) *δ* (ppm):** 177.5, 172.1, 167.0, 166.7, 161.8, 145.6, 135.2, 132.5, 129.1, 128.8, 128.7, 128.6, 128.2, 128.0, 126.6, 70.0, 64.1, 63.0, 62.9, 47.2, 42.8, 32.4, 31.2, 27.6, 23.6, 14.1, 14.0.

**HRMS** (ESI) *m/z* calcd for $C_{31}H_{35}N_2O_7S$ $[M+H]^+$: 579.2165; found: 579.2147.

**Diethyl 5-(3-(((3a*S*,4*S*,6a*S*)-2,2-dimethyl-6-oxotetrahydrofuro[3,4-*d*][1,3]dioxol-4-yl)methoxy)-3-oxopropyl)-2-iminodihydrothiophene-3,3(2*H*)-dicarboxylate (3ao):**

**Yield:** 66% (64 mg).

**Nature:** Colourless oil.

**$R_f$ value** = 0.37 [EtOAc:Petroleum ether = 1:1 (v/v)].

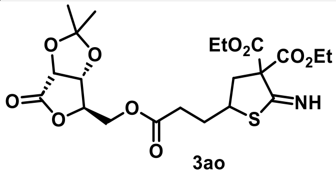


3ao

**$^1$H NMR (400 MHz, $CDCl_3$) *δ* (ppm):** 4.77 – 4.76 (m, 2H), 4.70 (t, *J* = 5.5 Hz, 1H), 4.41 – 4.37 (m, 1H), 4.29 – 4.20 (m, 5H), 3.74 – 3.67 (m, 1H), 2.96 – 2.91 (m, 1H), 2.51 – 2.39 (m, 3H), 2.17 – 2.04 (m, 1H), 2.01 – 1.89 (m, 1H), 1.47 (s, 3H), 1.38 (s, 3H), 1.29 – 1.23 (m, 3H), 1.28 (t, *J* = 7.1 Hz, 3H).

**$^{13}C\{^1H\}$ NMR (101 MHz, $CDCl_3$) *δ* (ppm):** 176.5, 173.5, 171.3, 166.9, 166.7, 114.0, 79.6, 77.8, 75.2, 69.8, 63.9, 63.0, 62.9, 46.1, 42.5, 32.2, 30.4, 26.8, 25.6, 14.1, 14.0.

**HRMS** (ESI) *m/z* calcd for $C_{21}H_{33}N_2O_{10}S$ $[M+NH_4]^+$: 505.1856; found: 505.1840.

**Diethyl 2-thioxo-5-(*p*-tolyl)pyrrolidine-3,3-dicarboxylate (4a):**

**Yield:** 79% (53 mg).

**Nature:** White solid.

**Mp:** 105 – 107 °C

**$R_f$ value** = 0.39 [EtOAc:Petroleum ether = 1:4 (v/v)].

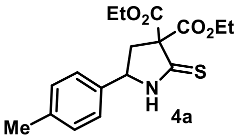


4a

**$^1$H NMR (400 MHz, $CDCl_3$) *δ* (ppm):** 8.28 (brs, 1H), 7.21 (d, *J* = 8.1 Hz, 2H), 7.18 (d, *J* = 8.3 Hz, 2H), 4.92 (t, *J* = 7.7 Hz, 1H), 4.34 – 4.29 (m, 2H), 4.27 (q, *J* = 7.1 Hz, 2H), 3.18 (dd, *J* = 13.4, 6.7 Hz, 1H), 2.84 (dd, *J* = 13.4, 8.7 Hz, 1H), 2.34 (s, 3H), 1.32 (t, *J* = 7.1 Hz, 3H), 1.29 (t, *J* = 7.1 Hz, 3H).

**$^{13}C\{^1H\}$ NMR (101 MHz, $CDCl_3$) *δ* (ppm):** 197.7, 167.3, 167.1, 138.9, 135.4, 129.9, 126.5, 71.8, 62.9, 62.7, 62.6, 42.7, 21.3, 14.1, 14.0.

**HRMS** (ESI) *m/z* calcd for $C_{17}H_{22}NO_4S$ $[M+H]^+$: 336.1270; found: 336.1261.

**Diethyl 5-phenyl-2-thioxopyrrolidine-3,3-dicarboxylate (4b):**

**Yield:** 72% (46 mg).

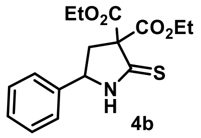


4b

**Nature:** White solid.

**Mp:** 83 – 85 °C

**$R_f$ value** = 0.42 [EtOAc:Petroleum ether = 1:4 (v/v)].

**$^1$H NMR (400 MHz, $CDCl_3$) *δ* (ppm):** 8.16 (brs, 1H), 7.41 – 7.31 (m, 5H), 4.96 (dd, *J* = 8.1, 7.3 Hz, 1H), 4.35 – 4.29 (m, 2H), 4.27 (q, *J* = 7.1 Hz, 2H), 3.23 (dd, *J* = 13.4, 6.8 Hz, 1H), 2.87 (dd, *J* = 13.4, 8.6 Hz, 1H), 1.33 (t, *J* = 7.1 Hz, 3H), 1.28 (t, *J* = 7.1 Hz, 3H).

**$^{13}$C{$^1$H} NMR (101 MHz, $CDCl_3$) *δ* (ppm):** 197.9, 167.2, 167.1, 138.6, 129.3, 129.1, 126.6, 71.7, 62.9, 62.8, 62.7, 42.7, 14.1, 14.0.

**HRMS** (ESI) *m/z* calcd for $C_{16}H_{19}NO_4SNa$ [M+Na]$^+$: 344.0932; found: 344.0938.

**Diethyl 5-(4-(*tert*-butyl)phenyl)-2-thioxopyrrolidine-3,3-dicarboxylate (4c):**

**Yield:** 79% (60 mg).

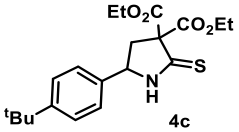


4c

**Nature:** White solid.

**Mp:** 134 – 136 °C

**$R_f$ value** = 0.39 [EtOAc:Petroleum ether = 1:4 (v/v)].

**$^1$H NMR (400 MHz, $CDCl_3$) *δ* (ppm):** 8.19 (brs, 1H), 7.40 (d, *J* = 8.4 Hz, 2H), 7.26 (d, *J* = 8.4 Hz, 2H), 4.94 (dd, *J* = 8.4, 7.0 Hz, 1H), 4.35 – 4.29 (m, 2H), 4.27 (q, *J* = 7.1 Hz, 2H), 3.19 (dd, *J* = 13.3, 6.7 Hz, 1H), 2.87 (dd, *J* = 13.3, 8.7 Hz, 1H), 1.32 (t, *J* = 7.1 Hz, 3H), 1.31 (s, 9H), 1.28 (t, *J* = 7.1 Hz, 3H).

**$^{13}$C{$^1$H} NMR (101 MHz, $CDCl_3$) *δ* (ppm):** 197.7, 167.3, 167.1, 152.2, 135.4, 126.3, 126.2, 71.7, 62.9, 62.7, 62.6, 42.7, 34.8, 31.4, 14.1.

**HRMS** (ESI) *m/z* calcd for $C_{20}H_{27}NO_4SNa$ [M+Na]$^+$: 400.1558; found: 400.1585.

**Diethyl 5-(4-chlorophenyl)-2-thioxopyrrolidine-3,3-dicarboxylate (4d):**

**Yield:** 73% (52 mg).

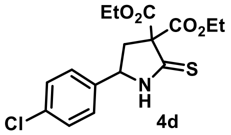


4d

**Nature:** White solid.

**Mp:** 117 – 119 °C

**$R_f$ value** = 0.35 [EtOAc:Petroleum ether = 1:4 (v/v)].

**$^1$H NMR (400 MHz, $CDCl_3$) *δ* (ppm):** 8.73 (brs, 1H), 7.34 (d, *J* = 8.4 Hz, 2H), 7.26 (d, *J* = 8.4 Hz, 2H), 4.95 (t, *J* = 7.6 Hz, 1H), 4.32 – 4.26 (m, 2H), 4.25 (q, *J* = 7.1 Hz, 2H), 3.21 (dd, *J* = 13.4, 6.9 Hz, 1H), 2.79 (dd, *J* = 13.5, 8.4 Hz, 1H), 1.30 (t, *J* = 7.1 Hz, 3H), 1.26 (t, *J* = 7.1 Hz, 3H).

**$^{13}$C{$^1$H} NMR (101 MHz, $CDCl_3$) *δ* (ppm):** 197.9, 167.2, 167.0, 137.1, 134.8, 129.4, 128.0, 71.6, 63.0, 62.8, 62.2, 42.5, 14.1, 14.0.

**HRMS** (ESI) *m/z* calcd for $C_{16}H_{19}ClNO_4S$ [M+H]$^+$: 356.0723; found: 356.0728.

**Diethyl 5-(4-fluorophenyl)-2-thioxopyrrolidine-3,3-dicarboxylate (4e):**

**Yield:** 75% (51 mg).

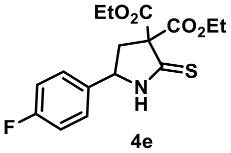


4e

**Nature:** White solid.

**Mp:** 122 – 124 °C

**$R_f$ value** = 0.34 [EtOAc:Petroleum ether = 1:4 (v/v)].

**$^{1}$H NMR (400 MHz, CDCl$_3$) *δ* (ppm):** 8.72 (brs, 1H), 7.30 (dd, *J* = 8.6, 1.3 Hz, 2H), 7.08 – 7.03 (m, 2H), 4.96 (t, *J* = 7.6 Hz, 1H), 4.32 – 4.26 (m, 2H), 4.25 (q, *J* = 7.1 Hz, 2H), 3.19 (dd, *J* = 13.4, 6.8 Hz, 1H), 2.81 (dd, *J* = 13.4, 8.4 Hz, 1H), 1.30 (t, *J* = 7.1 Hz, 3H), 1.26 (t, *J* = 7.1 Hz, 3H).

**$^{13}$C{$^{1}$H} NMR (101 MHz, CDCl$_3$) *δ* (ppm):** 197.7, 167.2, 167.1, 162.9 (d, *J* = 248.0 Hz), 134.3 (d, *J* = 3.1 Hz), 128.4 (d, *J* = 8.4 Hz), 116.2 (d, *J* = 21.7 Hz), 71.7, 62.9, 62.8, 62.2, 42.6, 14.1, 14.0.

**$^{19}$F NMR (377 MHz, CDCl$_3$) δ (ppm):** -112.7.

**HRMS** (ESI) *m/z* calcd for $C_{16}H_{18}FNO_4SNa$ [M+Na]$^+$: 362.0838; found: 362.0830.

**Diethyl 5-(3-methoxyphenyl)-2-thioxopyrrolidine-3,3-dicarboxylate (4f):**

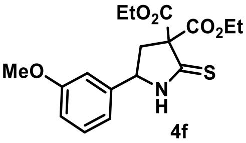

4f

**Yield:** 78% (55 mg).

**Nature:** White solid.

**Mp:** 161 – 163 °C

**R$_f$ value** = 0.37 [EtOAc:Petroleum ether = 1:4 (v/v)].

**$^{1}$H NMR (400 MHz, CDCl$_3$) *δ* (ppm):** 8.24 (brs, 1H), 7.29 (t, *J* = 7.9 Hz, 1H), 6.91 – 6.86 (m, 2H), 6.84 – 6.83 (m, 1H), 4.93 (dd, *J* = 8.2, 7.1 Hz, 1H), 4.35 – 4.29 (m, 2H), 4.27 (q, *J* = 7.1 Hz, 2H), 3.81 (s, 3H), 3.22 (dd, *J* = 13.3, 6.8 Hz, 1H), 2.85 (dd, *J* = 13.4, 8.6 Hz, 1H), 1.32 (t, *J* = 7.1 Hz, 3H), 1.28 (t, *J* = 7.1 Hz, 3H).

**$^{13}$C{$^{1}$H} NMR (101 MHz, CDCl$_3$) *δ* (ppm):** 197.9, 167.2, 167.1, 160.3, 140.2, 130.4, 118.6, 114.4, 112.0, 71.6, 62.9, 62.7, 55.5, 42.6, 14.1, 14.0.

**HRMS** (ESI) *m/z* calcd for $C_{17}H_{21}NO_5SNa$ [M+Na]$^+$: 374.1038; found: 374.1020.

**Diethyl 5-(2-bromophenyl)-2-thioxopyrrolidine-3,3-dicarboxylate (4g):**

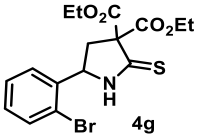

4g

**Yield:** 61% (49 mg).

**Nature:** White solid.

**Mp:** 171 – 173 °C

**R$_f$ value** = 0.34 [EtOAc:Petroleum ether = 1:4 (v/v)].

**$^{1}$H NMR (400 MHz, CDCl$_3$) *δ* (ppm):** 8.57 (brs, 1H), 7.57 (dd, *J* = 7.9, 0.9 Hz, 1H), 7.40 – 7.33 (m, 2H), 7.22 – 7.17 (m, 1H), 5.37 (t, *J* = 7.1 Hz, 1H), 4.32 (q, *J* = 7.1 Hz, 2H), 4.19 (q, *J* = 7.0 Hz, 2H), 3.43 (dd, *J* = 13.5, 7.5 Hz, 1H), 2.76 (dd, *J* = 13.5, 6.7 Hz, 1H), 1.32 (t, *J* = 7.1 Hz, 3H), 1.21 (t, *J* = 7.1 Hz, 3H).

**$^{13}$C{$^{1}$H} NMR (101 MHz, CDCl$_3$) *δ* (ppm):** 198.7, 167.1, 167.0, 138.1, 133.4, 130.1, 128.3, 126.9, 122.4, 71.2, 63.0, 62.7, 61.9, 40.7, 14.1, 14.0.

**HRMS** (ESI) *m/z* calcd for $C_{16}H_{18}BrNO_4SNa$ [M+Na]$^+$: 422.0038; found: 422.0033.

**Diethyl 5-(3-bromophenyl)-2-thioxopyrrolidine-3,3-dicarboxylate (4h):**

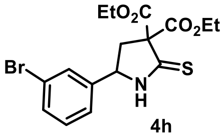

4h

**Yield:** 69% (55 mg).

**Nature:** White solid.

**Mp:** 132 – 134 °C

**R$_f$ value** = 0.35 [EtOAc:Petroleum ether = 1:4 (v/v)].

**$^1$H NMR (400 MHz, CDCl$_3$) *δ* (ppm):** 8.19 (brs, 1H), 7.50 – 7.46 (m, 1H), 7.47 (s, 1H), 7.27 – 7.26 (m, 2H), 4.94 (t, *J* = 7.6 Hz, 1H), 4.33 – 4.30 (m, 2H), 4.27 (q, *J* = 7.1 Hz, 2H), 3.24 (dd, *J* = 13.4, 6.9 Hz, 1H), 2.82 (dd, *J* = 13.4, 8.3 Hz, 1H), 1.32 (t, *J* = 7.1 Hz, 3H), 1.29 (t, *J* = 7.1 Hz, 3H).

**$^{13}$C{$^1$H} NMR (101 MHz, CDCl$_3$) *δ* (ppm):** 198.2, 167.1, 167.0, 141.0, 132.2, 130.9, 129.6, 125.2, 123.3, 71.5, 63.1, 62.9, 62.1, 42.5, 14.1.

**HRMS** (ESI) *m/z* calcd for $C_{16}H_{18}BrNO_4SNa$ [M+Na]$^+$: 422.0038; found: 422.0032.

**Diethyl 5-(4-bromophenyl)-2-thioxopyrrolidine-3,3-dicarboxylate (4i):**

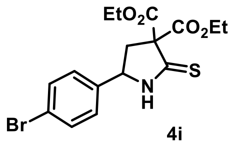


4i

**Yield:** 66% (53 mg).

**Nature:** White solid.

**Mp:** 119 – 121 °C

**$R_f$ value** = 0.34 [EtOAc:Petroleum ether = 1:4 (v/v)].

**$^1$H NMR (400 MHz, CDCl$_3$) *δ* (ppm):** 8.55 (brs, 1H), 7.51 (d, *J* = 8.4 Hz, 2H), 7.20 (d, *J* = 8.4 Hz, 2H), 4.94 (t, *J* = 7.6 Hz, 1H), 4.33 – 4.27 (m, 2H), 4.26 (q, *J* = 7.1 Hz, 2H), 3.21 (dd, *J* = 13.4, 6.9 Hz, 1H), 2.80 (dd, *J* = 13.4, 8.4 Hz, 1H), 1.31 (t, *J* = 7.1 Hz, 3H), 1.27 (t, *J* = 7.1 Hz, 3H).

**$^{13}$C{$^1$H} NMR (101 MHz, CDCl$_3$) *δ* (ppm):** 198.0, 167.1, 167.0, 137.7, 132.4, 128.3, 123.0, 71.6, 63.0, 62.8, 62.2, 42.5, 14.1.

**HRMS** (ESI) *m/z* calcd for $C_{16}H_{18}BrNO_4SNa$ [M+Na]$^+$: 422.0038; found: 422.0038.

**Diethyl 5-(naphthalen-2-yl)-2-thioxopyrrolidine-3,3-dicarboxylate (4j):**

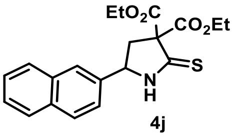


4j

**Yield:** 51% (38 mg).

**Nature:** White solid.

**Mp:** 184 – 186 °C

**$R_f$ value** = 0.41 [EtOAc:Petroleum ether = 1:4 (v/v)].

**$^1$H NMR (400 MHz, CDCl$_3$) *δ* (ppm):** 8.54 (brs, 1H), 7.87 (d, *J* = 8.6 Hz, 1H), 7.84 – 7.82 (m, 2H), 7.76 (s, 1H), 7.52 – 7.50 (m, 2H), 7.42 (dd, *J* = 8.5, 1.8 Hz, 1H), 5.13 (t, *J* = 7.6 Hz, 1H), 4.35 – 4.29 (m, 2H), 4.25 (q, *J* = 7.2 Hz, 2H), 3.28 (dd, *J* = 13.4, 6.9 Hz, 1H), 2.95 (dd, *J* = 13.4, 8.5 Hz, 1H), 1.32 (t, *J* = 7.1 Hz, 3H), 1.26 (t, *J* = 7.1 Hz, 3H).

**$^{13}$C{$^1$H} NMR (101 MHz, CDCl$_3$) *δ* (ppm):** 197.9, 167.3, 167.1, 135.8, 133.4, 133.2, 129.4, 128.1, 127.9, 126.9, 126.7, 125.9, 123.7, 71.7, 63.0, 62.9, 62.7, 42.5, 14.1, 14.0.

**HRMS** (ESI) *m/z* calcd for $C_{20}H_{21}NO_4SNa$ [M+Na]$^+$: 394.1089; found: 394.1090.

**Diethyl 5-mesityl-2-thioxopyrrolidine-3,3-dicarboxylate (4k):**

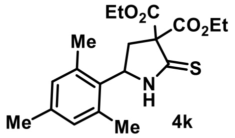


4k

**Yield:** 77% (56 mg).

**Nature:** White solid.

**Mp:** 153 – 155 °C

**$R_f$ value** = 0.38 [EtOAc:Petroleum ether = 1:4 (v/v)].

**$^1$H NMR (400 MHz, CDCl$_3$) *δ* (ppm):** 8.35 (brs, 1H), 6.84 (s, 2H), 5.40 (t, *J* = 8.6 Hz, 1H), 4.36 – 4.31 (m, 2H), 4.30 (q, *J* = 7.1 Hz, 2H), 3.04 (d, *J* = 8.4 Hz, 2H), 2.34 (s, 6H), 2.23 (s, 3H), 1.33 (t, *J* = 7.1 Hz, 3H), 1.30 (t, *J* = 7.1 Hz, 3H).

**$^{13}C\{^{1}H\}$ NMR (101 MHz, $CDCl_3$) δ (ppm):** 196.4, 167.3, 167.1, 138.3, 136.6, 131.0, 129.8, 71.8, 62.8, 62.7, 59.0, 39.0, 20.8, 14.1.

**HRMS** (ESI) *m/z* calcd for $C_{19}H_{25}NO_4SNa$ $[M+Na]^+$: 386.1402; found: 386.1393.

**Diethyl 5-(thiophen-3-yl)-2-thioxopyrrolidine-3,3-dicarboxylate (4l):**

**Yield:** 73% (48 mg).

**Nature:** Low melting solid.

**$R_f$ value** = 0.36 [EtOAc:Petroleum ether = 1:4 (v/v)].

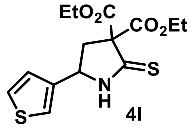


**$^1H$ NMR (400 MHz, $CDCl_3$) δ (ppm):** 8.48 (brs, 1H), 7.36 (dd, $J$ = 5.1, 3.0 Hz, 1H), 7.26 (s, 1H), 7.06 (dd, $J$ = 5.1, 1.2 Hz, 1H), 5.08 (t, $J$ = 7.5 Hz, 1H), 4.34 – 4.28 (m, 2H), 4.25 (q, $J$ = 7.1 Hz, 2H), 3.20 (dd, $J$ = 13.4, 6.8 Hz, 1H), 2.91 (dd, $J$ = 13.3, 8.0 Hz, 1H), 1.31 (t, $J$ = 7.1 Hz, 3H), 1.27 (t, $J$ = 7.1 Hz, 3H).

**$^{13}C\{^{1}H\}$ NMR (101 MHz, $CDCl_3$) δ (ppm):** 197.4, 167.2, 167.1, 139.4, 127.7, 125.6, 123.0, 71.6, 63.0, 62.8, 58.5, 41.6, 14.1, 14.0.

**HRMS** (ESI) *m/z* calcd for $C_{14}H_{17}NO_4S_2Na$ $[M+Na]^+$: 350.0497; found: 350.0508.

**Diethyl 5-(1-(*tert*-butoxycarbonyl)-1*H*-indol-3-yl)-2-mercapto-4,5-dihydro-3*H*-pyrrole-3,3-dicarboxylate (4m):**

**Yield:** 36% (33 mg).

**Nature:** Transparent gummy liquid.

**$R_f$ value** = 0.32 [EtOAc:Petroleum ether = 1:4 (v/v)].

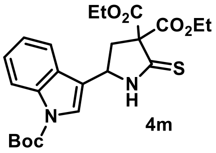


**$^1H$ NMR (400 MHz, $CDCl_3$) δ (ppm):** 8.17 (d, $J$ = 8.2 Hz, 1H), 8.11 (brs, 1H), 7.60 (d, $J$ = 7.5 Hz, 2H), 7.36 (t, $J$ = 7.8 Hz, 1H), 7.26 (t, $J$ = 7.8 Hz, 1H), 5.22 (t, $J$ = 7.8 Hz, 1H), 4.37 – 4.33 (m, 2H), 4.31 (q, $J$ = 7.1 Hz, 2H), 3.20 (dd, $J$ = 7.8, 4.3 Hz, 2H), 1.67 (s, 9H), 1.34 (t, $J$ = 7.1 Hz, 3H), 1.31 (t, $J$ = 7.1 Hz, 3H).

**$^{13}C\{^{1}H\}$ NMR (101 MHz, $CDCl_3$) δ (ppm):** 197.7, 167.2, 167.1, 149.4, 136.2, 127.5, 125.4, 124.1, 123.3, 119.3, 117.3, 115.9, 84.6, 71.8, 63.0, 62.8, 56.0, 40.2, 28.3, 14.1, 14.0.

**HRMS** (ESI) *m/z* calcd for $C_{23}H_{29}N_2O_6S$ $[M+H]^+$: 461.1746; found: 461.1742.

**Diethyl 5-(4-methylthiazol-5-yl)-2-thioxopyrrolidine-3,3-dicarboxylate (4n):**

**Yield:** 32% (22 mg).

**Nature:** Low melting solid.

**$R_f$ value** = 0.34 [EtOAc:Petroleum ether = 3:7 (v/v)].

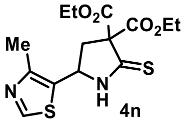


**$^1H$ NMR (400 MHz, $CDCl_3$) δ (ppm):** 8.72 (s, 1H), 8.34 (brs, 1H), 5.29 (dd, $J$ = 8.6, 6.9 Hz, 1H), 4.36 – 4.32 (m, 2H), 4.31 (q, $J$ = 7.1 Hz, 2H), 3.27 (dd, $J$ = 13.4, 6.7 Hz, 1H), 2.89 (dd, $J$ = 13.4, 8.9 Hz, 1H), 2.45 (s, 3H), 1.33 (t, $J$ = 7.1 Hz, 3H), 1.32 (t, $J$ = 7.2 Hz, 3H).

**$^{13}C\{^{1}H\}$ NMR (101 MHz, $CDCl_3$) δ (ppm):** 197.1, 166.9, 166.7, 152.2, 151.5, 129.6, 71.5, 63.1, 63.0, 55.4, 42.7, 15.6, 14.1.

**HRMS** (ESI) *m/z* calcd for $C_{14}H_{18}N_2O_4S_2Na$ $[M+Na]^+$: 365.0606; found: 365.0594.

**Diethyl 5-methyl-5-phenyl-2-thioxopyrrolidine-3,3-dicarboxylate (4o):**

**Yield:** 57% (38 mg).

**Nature:** White solid.

**Mp:** 160 – 162 °C

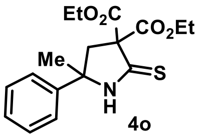


4o

$R_f$ **value** = 0.42 [EtOAc:Petroleum ether = 1:4 (v/v)].

**$^1$H NMR (400 MHz, CDCl$_3$) $\delta$ (ppm):** 9.50 (brs, 1H), 7.36 – 7.31 (m, 4H), 7.29 – 7.24 (m, 1H), 4.30 (q, $J$ = 7.4 Hz, 2H), 3.95 – 3.85 (m, 2H), 3.22 (d, $J$ = 13.3 Hz, 1H), 3.14 (d, $J$ = 13.3 Hz, 1H), 1.73 (s, 3H), 1.30 (t, $J$ = 7.1 Hz, 3H), 1.03 (t, $J$ = 7.1 Hz, 3H).

**$^{13}$C{$^1$H} NMR (101 MHz, CDCl$_3$) $\delta$ (ppm):** 197.1, 167.6, 167.0, 143.5, 128.8, 127.8, 125.0, 71.9, 68.1, 63.0, 62.5, 48.0, 29.3, 14.1, 13.7.

**HRMS** (ESI) *m/z* calcd for $C_{17}H_{21}NO_4SNa$ [M+Na]$^+$: 358.1089; found: 358.1071.

**Diethyl 4-methyl-5-phenyl-2-thioxopyrrolidine-3,3-dicarboxylate (4p):**

**Yield:** 51% (34 mg, combined yield as a 5:1 mixture of diastereomers).

**Nature:** Low melting solid.

$R_f$ **value** = 0.43 [EtOAc:Petroleum ether = 1:4 (v/v)].

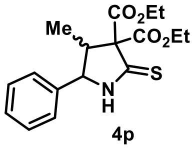


4p

**$^1$H NMR (400 MHz, CDCl$_3$) $\delta$ (ppm):** (for the major isomer) 7.83 (brs, 1H), 7.43 – 7.37 (m, 3H), 7.34 – 7.32 (m, 2H), 4.48 (d, $J$ = 9.8 Hz, 1H), 4.42 – 4.18 (m, 4H), 3.31 – 3.23 (m, 1H), 1.37 (t, $J$ = 7.1 Hz, 3H), 1.35 (t, $J$ = 7.1 Hz, 3H), 1.05 (d, $J$ = 6.9 Hz, 1H); (for the minor isomer) 7.83 (brs, 1H), 7.43 – 7.37 (m, 3H), 7.34 – 7.32 (m, 2H), 5.25 (d, $J$ = 8.1 Hz, 1H), 4.42 – 4.18 (m, 4H), 3.67 – 3.64 (m, 1H), 1.25 (t, $J$ = 7.1 Hz, 6H), 0.69 (d, $J$ = 7.5 Hz, 1H).

**$^{13}$C{$^1$H} NMR (101 MHz, CDCl$_3$) $\delta$ (ppm):** (for the major isomer) 198.1, 166.3, 165.8, 137.1, 129.4, 129.3, 127.2, 75.1, 68.7, 62.7, 62.4, 49.3, 14.3, 14.2, 12.0; (for the minor isomer) 198.2, 166.4, 166.3, 136.0, 129.5, 129.4, 127.3, 74.4, 65.5, 63.2, 62.9, 54.8, 14.1, 13.9, 11.7.

**HRMS** (ESI) *m/z* calcd for $C_{17}H_{22}NO_4S$ [M+H]$^+$: 336.1270; found: 336.1266.

**Diethyl (3a*R*,9b*S*)-2-thioxo-1,2,3a,4,5,9b-hexahydro-3*H*-benzo[*g*]indole-3,3-dicarboxylate (4q):**

**Yield:** 72% (50 mg, combined yield as a 2:1 mixture of diastereomers).

Characterization data was obtained for the major diastereomer by flash-column chromatography.

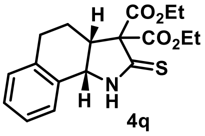


4q

**Nature:** White solid.

**Mp:** 194 – 196 °C

$R_f$ **value** = 0.33 [EtOAc:Petroleum ether = 1:4 (v/v)].

**$^1$H NMR (400 MHz, CDCl$_3$) $\delta$ (ppm):** 9.12 (brs, 1H), 7.25 – 7.14 (m, 4H), 4.68 (d, $J$ = 11.1 Hz, 1H), 4.41 – 4.24 (m, 4H), 3.18 – 3.00 (m, 3H), 2.22 – 2.16 (m, 1H), 1.84 – 1.73 (m, 1H), 1.34 (t, $J$ = 7.1 Hz, 3H), 1.32 (t, $J$ = 7.1 Hz, 3H).

**$^{13}$C{$^1$H} NMR (101 MHz, CDCl$_3$) $\delta$ (ppm):** 199.9, 166.2, 165.6, 135.0, 134.4, 129.6, 127.9, 126.2, 123.3, 73.9, 62.7, 62.5, 62.2, 49.4, 28.8, 22.0, 14.4, 14.2.

**HRMS** (ESI) *m/z* calcd for $C_{18}H_{21}NO_4SNa$ [M+Na]$^+$: 370.1089; found: 370.1115.

**Diethyl (3a*R*,8b*S*)-2-thioxo-1,3a,4,8b-tetrahydroindeno[1,2-*b*]pyrrole-3,3(2*H*)-dicarboxylate (4r):**

**Yield:** 75% (50 mg).

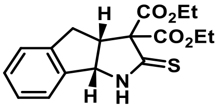

4r

**Nature:** White solid.

**Mp:** 136 – 138 °C

**$R_f$ value** = 0.34 [EtOAc:Petroleum ether = 1:4 (v/v)].

**$^1$H NMR (400 MHz, $CDCl_3$) *δ* (ppm):** 8.77 (brs, 1H), 7.34 – 7.27 (m, 2H), 7.24 – 7.20 (m, 2H), 5.33 (d, *J* = 6.5 Hz, 1H), 4.36 – 4.25 (m, 2H), 4.23 (q, *J* = 7.2 Hz, 2H), 4.07 – 4.02 (m, 1H), 3.16 – 3.01 (m, 2H), 1.32 (t, *J* = 7.1 Hz, 3H), 1.25 (t, *J* = 7.1 Hz, 3H).

**$^{13}$C{$^1$H} NMR (101 MHz, $CDCl_3$) *δ* (ppm):** 195.3, 166.7, 166.2, 142.7, 138.3, 129.6, 127.6, 125.2, 125.0, 73.8, 68.5, 63.2, 62.1, 48.8, 34.0, 14.1, 14.0.

**HRMS** (ESI) *m/z* calcd for $C_{17}H_{19}NO_4SNa$ $[M+Na]^+$: 356.0932; found: 356.0907.

**Diethyl 5-ethoxy-2-thioxopyrrolidine-3,3-dicarboxylate (4s):**

**Yield:** 66% (38 mg).

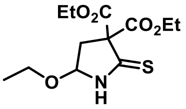

4s

**Nature:** Pale yellow oil.

**$R_f$ value** = 0.42 [EtOAc:Petroleum ether = 3:7 (v/v)].

**$^1$H NMR (400 MHz, $CDCl_3$) *δ* (ppm):** 8.84 (brs, 1H), 5.09 (d, *J* = 5.7 Hz, 1H), 4.32 – 4.26 (m, 4H), 3.62 – 3.55 (m, 1H), 349 – 3.41 (m, 1H), 3.13 (dd, *J* = 13.7, 5.8 Hz, 1H), 2.81 (dd, *J* = 13.7, 1.5 Hz, 1H), 1.31 (t, *J* = 7.1, 3H), 1.30 (t, *J* = 7.1, 3H), 1.18 (t, *J* = 7.0 Hz, 3H).

**$^{13}$C{$^1$H} NMR (101 MHz, $CDCl_3$) *δ* (ppm):** 200.2, 167.2, 88.4, 70.2, 64.1, 63.1, 62.6, 40.5, 15.2, 14.1, 14.0.

**HRMS** (ESI) *m/z* calcd for $C_{12}H_{19}NO_5SNa$ $[M+Na]^+$: 312.0882; found: 312.0884.

**Diethyl 5-phenoxy-2-thioxopyrrolidine-3,3-dicarboxylate (4t):**

**Yield:** 73% (49 mg).

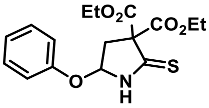

4t

**Nature:** Pale yellow oil.

**$R_f$ value** = 0.39 [EtOAc:Petroleum ether = 3:7 (v/v)].

**$^1$H NMR (400 MHz, DMSO-$d_6$) *δ* (ppm):** 11.54 (brs, 1H), 7.34 – 7.30 (m, 2H), 7.02 (t, *J* = 7.3 Hz, 1H), 6.93 (dd, *J* = 8.7, 0.8 Hz, 2H), 6.03 (dd, *J* = 5.0, 1.7 Hz, 1H), 4.23 – 4.17 (m, 2H), 4.19 (q, *J* = 7.2 Hz, 2H), 3.16 (dd, *J* = 13.8, 5.4 Hz, 1H), 2.79 (d, *J* = 13.9 Hz, 1H), 1.21 (t, *J* = 7.1 Hz, 3H), 1.17 (t, *J* = 7.1 Hz, 3H).

**$^{13}$C{$^1$H} NMR (101 MHz, DMSO-$d_6$) *δ* (ppm):** 199.9, 166.9, 166.8, 155.5, 129.7, 122.0, 116.1, 86.4, 70.3, 62.2, 61.7, 39.7, 13.8, 13.7.

**HRMS** (ESI) *m/z* calcd for $C_{16}H_{19}NO_5SNa$ $[M+Na]^+$: 360.0882; found: 360.0885.

**Diethyl 5-(cyclohexyloxy)-2-thioxopyrrolidine-3,3-dicarboxylate (4u):**

**Yield:** 64% (44 mg).

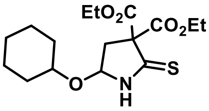

4u

**Nature:** Pale yellow oil.

**$R_f$ value** = 0.44 [EtOAc:Petroleum ether = 3:7 (v/v)].

**$^1$H NMR (400 MHz, $CDCl_3$) *δ* (ppm):** 9.37 (brs, 1H), 5.22 (dd, *J* = 5.7, 2.1 Hz, 1H), 4.29 – 4.17 (m, 2H), 4.27 (q, *J* = 7.2 Hz, 2H), 3.43 – 3.38 (m, 1H), 3.07 (dd, *J* = 13.5, 5.7 Hz, 1H), 2.72 (dd, *J* = 13.5,

2.1 Hz, 1H), 2.04 – 1.60 (m, 6H), 1.49 – 1.44 (m, 2H), 1.30 – 1.25 (m, 2H), 1.28 (t, *J* = 7.1 Hz, 3H), 1.27 (t, *J* = 7.1 Hz, 3H).

**$^{13}$C{$^1$H} NMR (101 MHz, CDCl$_3$) *δ* (ppm):** 199.5, 167.3, 167.2, 86.3, 76.1, 70.4, 62.9, 62.4, 41.1, 33.0, 31.7, 25.5, 23.8, 23.7, 14.0.

**HRMS** (ESI) *m/z* calcd for $C_{16}H_{25}NO_5SNa$ [M+Na]$^+$: 366.1351; found: 366.1353.

**Diethyl 5-(9*H*-carbazol-9-yl)-2-thioxopyrrolidine-3,3-dicarboxylate (4v):**

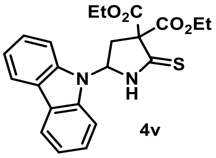

4v

**Yield:** 78% (64 mg).

**Nature:** White solid.

**Mp:** 199 – 201 °C

**R$_f$ value** = 0.34 [EtOAc:Petroleum ether = 2:3 (v/v)].

**$^1$H NMR (400 MHz, CDCl$_3$) *δ* (ppm):** 8.08 (d, *J* = 7.7 Hz, 2H), 7.95 (brs, 1H), 7.52 (d, *J* = 8.3 Hz, 2H), 7.48 – 7.44 (m, 2H), 7.31 – 7.27 (m, 2H), 6.71 (t, *J* = 7.7 Hz, 1H), 4.37 (q, *J* = 7.1 Hz, 4H), 3.55 (dd, *J* = 14.0, 8.3 Hz, 1H), 3.30 (dd, *J* = 14.0, 7.1 Hz, 1H), 1.37 (t, *J* = 7.1 Hz, 3H), 1.36 (t, *J* = 7.1 Hz, 3H).

**$^{13}$C{$^1$H} NMR (101 MHz, CDCl$_3$) *δ* (ppm):** 196.6, 167.0, 166.7, 138.5, 126.5, 124.3, 120.8, 110.3, 71.2, 69.2, 63.4, 63.0, 37.0, 14.1, 14.0.

**HRMS** (ESI) *m/z* calcd for $C_{22}H_{23}N_2O_4S$ [M+H]$^+$: 411.1379; found: 411.1376.

**Diethyl 5-(2-oxooxazolidin-3-yl)-2-thioxopyrrolidine-3,3-dicarboxylate (4w):**

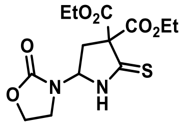

4w

**Yield:** 47% (31 mg).

**Nature:** Pale yellow oil.

**R$_f$ value** = 0.38 [EtOAc:Petroleum ether = 1:1 (v/v)].

**$^1$H NMR (400 MHz, CDCl$_3$) *δ* (ppm):** 8.61 (brs, 1H), 5.89 (dd, *J* = 7.8, 5.9 Hz, 1H), 4.40 (dd, *J* = 15.8, 7.3 Hz, 2H), 4.35 – 4.27 (m, 4H), 3.63 – 3.59 (m, 2H), 3.17 (dd, *J* = 14.4, 7.9 Hz, 1H), 2.85 (dd, *J* = 14.4, 5.8 Hz, 1H), 1.32 (t, *J* = 7.1 Hz, 3H), 1.30 (t, *J* = 7.1 Hz, 3H).

**$^{13}$C{$^1$H} NMR (101 MHz, CDCl$_3$) *δ* (ppm):** 197.2, 167.3, 166.8, 157.8, 70.4, 68.0, 63.2, 63.1, 62.8, 39.4, 35.5, 14.1, 14.0.

**HRMS** (ESI) *m/z* calcd for $C_{13}H_{19}N_2O_6S$ [M+H]$^+$: 331.0964; found: 331.0968.

**Diethyl 5-formamido-2-thioxopyrrolidine-3,3-dicarboxylate (4x):**

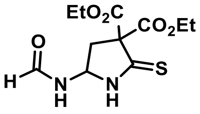

4x

**Yield:** 40% (23 mg).

**Nature:** Pale yellow oil.

**R$_f$ value** = 0.37 [EtOAc:Petroleum ether = 1:1 (v/v)].

**$^1$H NMR (400 MHz, CDCl$_3$) *δ* (ppm):** 8.85 (brs, 1H), 8.18 (brs, 1H), 7.28 (brs, 1H), 5.87 (td, *J* = 8.3, 2.5 Hz, 1H), 4.35 – 4.24 (m, 4H), 3.04 (dd, *J* = 14.1, 7.8 Hz, 1H), 2.76 (dd, *J* = 14.1, 2.8 Hz, 1H), 1.32 (t, *J* = 7.1 Hz, 3H), 1.29 (t, *J* = 7.1 Hz, 3H).

**$^{13}$C{$^1$H} NMR (101 MHz, CDCl$_3$) *δ* (ppm):** 196.7, 168.3, 166.7, 161.7, 70.2, 63.7, 62.9, 62.4, 38.5, 14.1, 14.0.

**HRMS** (ESI) *m/z* calcd for $C_{11}H_{16}N_2O_5SNa$ [M+Na]$^+$: 311.0678; found: 311.0679.

**Diethyl 5-acetamido-2-thioxopyrrolidine-3,3-dicarboxylate (4y):**

**Yield:** 43% (26 mg).

**Nature:** Pale yellow oil.

**R$_f$ value** = 0.43 [EtOAc:Petroleum ether = 1:1 (v/v)].

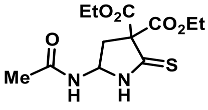

4y

**$^1$H NMR (400 MHz, CDCl$_3$) *δ* (ppm):** 8.38 (brs, 1H), 6.97 (d, *J* = 8.0 Hz, 1H), 5.75 (t, *J* = 7.5 Hz, 1H), 4.37 – 4.22 (m, 4H), 2.99 (dd, *J* = 14.1, 7.9 Hz, 1H), 2.75 (dd, *J* = 14.1, 2.3 Hz, 1H), 2.02 (s, 3H), 1.34 (t, *J* = 7.1 Hz, 3H), 1.30 (t, *J* = 7.1 Hz, 3H).

**$^{13}$C{$^1$H} NMR (101 MHz, CDCl$_3$) *δ* (ppm):** 196.4, 171.1, 168.2, 166.8, 70.2, 64.0, 63.6, 62.8, 38.4, 23.3, 14.1, 14.0.

**HRMS** (ESI) *m/z* calcd for $C_{12}H_{19}N_2O_5S$ $[M+H]^+$: 303.1015; found: 303.1017.

**Diethyl 5-(2-oxoazepan-1-yl)-2-thioxopyrrolidine-3,3-dicarboxylate (4z):**

**Yield:** 63% (45 mg).

**Nature:** White solid.

**Mp:** 180 – 182 °C

**R$_f$ value** = 0.42 [EtOAc:Petroleum ether = 1:1 (v/v)].

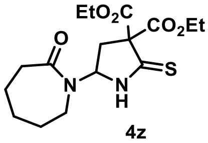

4z

**$^1$H NMR (400 MHz, CDCl$_3$) *δ* (ppm):** 7.82 (brs, 1H), 6.58 (t, *J* = 7.3 Hz, 1H), 4.35 – 4.26 (m, 4H), 3.32 (t, *J* = 6.4 Hz, 2H), 3.06 (dd, *J* = 14.3, 7.6 Hz, 1H), 2.75 (dd, *J* = 14.3, 6.9 Hz, 1H), 2.57 (t, *J* = 4.9 Hz, 2H), 1.78 – 1.63 (m, 6H), 1.33 (t, *J* = 7.1 Hz, 3H), 1.30 (t, *J* = 7.1 Hz, 3H).

**$^{13}$C{$^1$H} NMR (101 MHz, CDCl$_3$) *δ* (ppm):** 197.4, 177.1, 167.3, 166.9, 70.5, 67.4, 63.1, 42.4, 37.7, 36.0, 29.9, 29.7, 23.6, 14.1, 14.0.

**HRMS** (ESI) *m/z* calcd for $C_{16}H_{25}N_2O_5S$ $[M+H]^+$: 357.1484; found: 357.1487.

**Dimethyl 5-phenyl-2-thioxopyrrolidine-3,3-dicarboxylate (4aa):**

**Yield:** 73% (43 mg).

**Nature:** White solid.

**Mp:** 147 – 149 °C

**R$_f$ value** = 0.34 [EtOAc:Petroleum ether = 1:4 (v/v)].

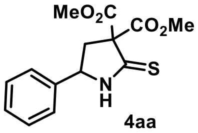

4aa

**$^1$H NMR (400 MHz, CDCl$_3$) *δ* (ppm):** 8.04 (brs, 1H), 7.43 – 7.36 (m, 3H), 7.35 – 7.32 (m, 2H), 4.96 (dd, *J* = 8.5, 6.8 Hz, 1H), 3.88 (s, 3H), 3.83 (s, 3H), 3.25 (dd, *J* = 13.3, 6.7 Hz, 1H), 2.90 (dd, *J* = 13.5, 8.7 Hz, 1H).

**$^{13}$C{$^1$H} NMR (101 MHz, CDCl$_3$) *δ* (ppm):** 197.8, 167.7, 138.3, 129.4, 129.2, 126.6, 71.9, 62.9, 54.0, 53.8, 42.9.

**HRMS** (ESI) *m/z* calcd for $C_{14}H_{15}NO_4SNa$ $[M+Na]^+$: 316.0619; found: 316.0619.

**Diisopropyl 5-phenyl-2-thioxopyrrolidine-3,3-dicarboxylate (4ab):**

**Yield:** 69% (48 mg).

**Nature:** Low melting solid.

**R$_f$ value** = 0.38 [EtOAc:Petroleum ether = 1:4 (v/v)].

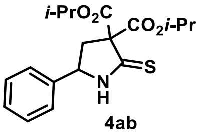

4ab

**$^1$H NMR (400 MHz, CDCl$_3$) $\delta$ (ppm):** 8.58 (brs, 1H), 7.38 – 7.29 (m, 5H), 5.25 – 5.04 (m, 2H), 4.95 (t, $J$ = 7.6 Hz, 1H), 3.19 (dd, $J$ = 13.4, 7.0 Hz, 1H), 2.81 (dd, $J$ = 13.3, 8.2 Hz, 1H), 1.29 (d, $J$ = 5.6 Hz, 6H), 1.27 (d, $J$ = 5.8 Hz, 3H), 1.20 (d, $J$ = 6.3 Hz, 3H).

**$^{13}$C{$^1$H} NMR (101 MHz, CDCl$_3$) $\delta$ (ppm):** 198.0, 166.8, 166.6, 138.8, 129.0, 128.7, 126.5, 71.4, 70.5, 70.4, 62.6, 42.4, 21.7, 21.6, 21.5, 21.4.

**HRMS** (ESI) *m/z* calcd for $C_{18}H_{24}NO_4S$ [M+H]$^+$: 350.1426; found: 350.1430.

**Dibenzyl 5-phenyl-2-thioxopyrrolidine-3,3-dicarboxylate (4ac):**

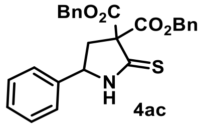


4ac

**Yield:** 56% (50 mg).

**Nature:** White solid.

**Mp:** 139 – 141 °C

**R$_f$ value** = 0.34 [EtOAc:Petroleum ether = 1:4 (v/v)].

**$^1$H NMR (400 MHz, CDCl$_3$) $\delta$ (ppm):** 7.83 (brs, 1H), 7.38 – 7.26 (m, 15H), 5.26 (s, 2H), 5.21 (s, 2H), 4.90 (dd, $J$ = 8.1, 7.3 Hz, 1H), 3.22 (dd, $J$ = 13.5, 6.8 Hz, 1H), 2.88 (dd, $J$ = 13.5, 8.6 Hz, 1H).

**$^{13}$C{$^1$H} NMR (101 MHz, CDCl$_3$) $\delta$ (ppm):** 197.5, 167.0, 166.9, 138.4, 135.1, 129.3, 129.2, 128.8, 128.7, 128.6, 128.5, 128.4, 128.3, 126.6, 71.9, 68.5, 68.4, 62.8, 42.8.

**HRMS** (ESI) *m/z* calcd for $C_{26}H_{24}NO_4S$ [M+H]$^+$: 446.1426; found: 446.1429.

**3-Benzyl 3-ethyl 5-phenyl-2-thioxopyrrolidine-3,3-dicarboxylate (4ad):**

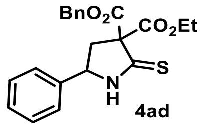


4ad

**Yield:** 65% (50 mg, combined yield as a 1.3:1 mixture of diastereomers).

**Nature:** Low melting solid.

**R$_f$ value** = 0.33 [EtOAc:Petroleum ether = 1:4 (v/v)].

**$^1$H NMR (400 MHz, CDCl$_3$) $\delta$ (ppm):** (for the major isomer) 7.97 (brs, 1H), 7.42 – 7.33 (m, 8H), 7.31 – 7.28 (m, 2H), 5.24 (q, $J$ = 12.3 Hz, 2H), 4.97 (t, $J$ = 7.6 Hz, 1H), 4.28 (q, $J$ = 7.1 Hz, 2H), 3.25 (dd, $J$ = 13.6, 7.0 Hz, 1H), 2.86 (dd, $J$ = 13.4, 8.4 Hz, 1H), 1.24 (t, $J$ = 7.2 Hz, 3H); (for the minor isomer) 7.96 (brs, 1H), 7.42 – 7.33 (m, 8H), 7.31 – 7.28 (m, 2H), 5.30 (s, 2H), 4.88 (dd, $J$ = 8.5, 7.0 Hz, 1H), 4.27 – 4.21 (m, 2H), 3.20 (dd, $J$ = 14.0, 7.2 Hz, 1H), 2.89 (dd, $J$ = 13.3, 8.9 Hz, 1H), 1.22 (t, $J$ = 7.3 Hz, 3H).

**$^{13}$C{$^1$H} NMR (101 MHz, CDCl$_3$) $\delta$ (ppm):** (for the mixture) 197.6, 197.5, 167.2, 167.1, 167.0, 166.9, 138.6, 138.5,135.2, 135.1, 129.2, 129.0, 128.9, 128.7, 128.6, 128.5, 128.4, 128.3, 128.2, 126.5, 71.8, 71.7, 68.3, 68.2, 63.0, 62.9, 62.8, 42.7, 42.6, 13.9.

**HRMS** (ESI) *m/z* calcd for $C_{21}H_{22}NO_4S$ [M+H]$^+$: 384.1270; found: 384.1272.

**Diethyl 5-(4-((((8*S*,9*R*,13*R*,14*R*)-13-methyl-17-oxo-7,8,9,11,12,13,14,15,16,17-decahydro-6*H*-cyclopenta[*a*]phenanthren-3-yl)oxy)methyl)phenyl)-2-thioxopyrrolidine-3,3-dicarboxylate (4ae):**

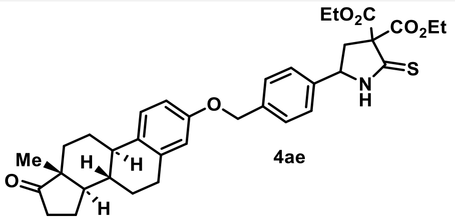


4ae

**Yield:** 67% (81 mg).

**Nature:** Low melting solid.

**R$_f$ value** = 0.34 [EtOAc:Petroleum ether = 2:3 (v/v)].

**$^1$H NMR (400 MHz, CDCl$_3$) *δ* (ppm):** 8.05 (brs, 1H), 7.45 (d, *J* = 8.1 Hz, 2H), 7.34 (d, *J* = 8.1 Hz, 2H), 7.20 (d, *J* = 8.6 Hz, 1H), 6.76 (dd, *J* = 8.5, 2.8 Hz, 1H), 6.72 – 6.68 (m, 1H), 5.03 (s, 2H), 4.96 (t, *J* = 7.7 Hz, 1H), 4.39 – 4.30 (m, 2H), 4.28 (q, *J* = 7.1 Hz, 2H), 3.23 (dd, *J* = 13.4, 6.8 Hz, 1H), 2.91 – 2.84 (m, 3H), 2.50 (dd, *J* = 18.8, 8.6 Hz, 1H), 2.44 – 2.34 (m, 1H), 2.27 – 2.22 (m, 1H), 2.19 – 2.05 (m, 2H), 2.03 – 1.94 (m, 2H), 1.65 – 1.41 (m, 6H), 1.33 (t, *J* = 7.1 Hz, 3H), 1.29 (t, *J* = 7.1 Hz, 3H), 0.90 (s, 3H).

**$^{13}$C{$^1$H} NMR (101 MHz, CDCl$_3$) *δ* (ppm):** 221.1, 198.0, 167.2, 167.1, 156.7, 138.3, 138.2, 138.0, 132.7, 128.3, 126.8, 126.5, 115.0, 112.4, 71.7, 69.5, 62.9, 62.8, 62.5, 50.5, 48.1, 44.1, 42.7, 38.5, 36.0, 31.7, 29.8, 26.6, 26.0, 21.7, 14.1, 14.0.

**HRMS** (ESI) *m/z* calcd for $C_{35}H_{41}NO_6SNa$ [M+Na]$^+$: 626.2552; found: 626.2551.

**Diethyl 5-(4-((((*R*)-2,5,7,8-tetramethyl-2-((4*R*,8*R*)-4,8,12-trimethyltridecyl)chroman-6-yl)oxy)methyl)phenyl)-2-thioxopyrrolidine-3,3-dicarboxylate (4af):**

**Yield:** 63% (96 mg).

**Nature:** Low melting solid.

**R$_f$ value** = 0.33 [EtOAc:Petroleum ether = 2:3 (v/v)].

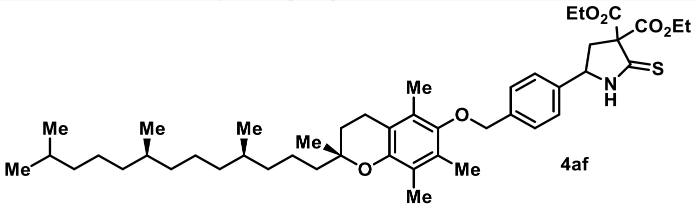


**4af**

**$^1$H NMR (400 MHz, CDCl$_3$) *δ* (ppm):** 7.83 (brs, 1H), 7.54 (d, *J* = 8.1 Hz, 2H), 7.37 (d, *J* = 8.1 Hz, 2H), 4.98 (dd, *J* = 8.5, 6.9 Hz, 1H), 4.69 (s, 2H), 4.39 – 4.32 (m, 2H), 4.30 (q, *J* = 7.1 Hz, 2H), 3.25 (dd, *J* = 13.4, 6.7 Hz, 1H), 2.89 (dd, *J* = 13.5, 8.7 Hz, 1H), 2.59 (t, *J* = 6.7 Hz, 2H), 2.20 (s, 3H), 2.15 (s, 3H), 2.10 (s, 3H), 1.86 – 1.73 (m, 2H), 1.55 – 1.48 (m, 4H), 1.39 – 1.27 (m, 4H), 1.35 (t, *J* = 7.1 Hz, 3H), 1.31 (t, *J* = 7.1 Hz, 3H), 1.24 (brs, 9H), 1.15 – 1.05 (m, 7H), 0.87 – 0.83 (m, 12H).

**$^{13}$C{$^1$H} NMR (101 MHz, CDCl$_3$) *δ* (ppm):** 198.0, 167.2, 167.1, 148.2, 148.0, 139.2, 138.0, 128.5, 127.9, 126.7, 126.0, 123.2, 117.8, 75.0, 74.1, 71.7, 63.0, 62.8, 62.6, 42.9, 40.2, 39.5, 37.7, 37.6, 37.5, 37.4, 32.9, 32.8, 31.4, 28.1, 25.0, 24.6, 24.0, 22.9, 22.8, 21.2, 20.8, 19.9, 19.8, 19.7, 14.1, 13.0, 12.1, 12.0.

**HRMS** (ESI) *m/z* calcd for $C_{46}H_{69}NO_6SNa$ [M+Na]$^+$: 786.4743; found: 786.4773.

**Diethyl 5-(4-((((*tert*-butoxycarbonyl)-*L*-valyl)oxy)methyl)phenyl)-2-thioxopyrrolidine-3,3-dicarboxylate (4ag):**

**Yield:** 51% (56 mg).

**Nature:** Colourless oil.

**R$_f$ value** = 0.31 [EtOAc:Petroleum ether = 3:7 (v/v)].

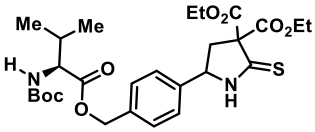


**4ag**

**$^1$H NMR (400 MHz, CDCl$_3$) *δ* (ppm):** 8.00 (brs, 1H), 7.38 (d, *J* = 8.1 Hz, 2H), 7.33 (d, *J* = 8.2 Hz, 2H), 5.15 (q, *J* = 12.6 Hz, 2H), 5.01 (d, *J* = 9.1 Hz, 1H), 4.96 (dd, *J* = 8.0, 7.3 Hz, 1H), 4.38 – 4.24 (m, 2H), 4.28 (q, *J* = 7.1 Hz, 2H), 3.23 (dd, *J* = 13.4, 6.8 Hz, 1H), 2.85 (dd, *J* = 13.4, 8.6 Hz, 1H), 2.17 – 2.10 (m, 1H), 1.63 (s, 1H), 1.43 (s, 9H), 1.33 (t, *J* = 7.1 Hz, 3H), 1.29 (t, *J* = 7.1 Hz, 3H), 0.94 (d, *J* = 6.8 Hz, 3H), 0.85 (d, *J* = 6.9 Hz, 3H).

**$^{13}$C{$^1$H} NMR (101 MHz, CDCl$_3$) *δ* (ppm):** 198.0, 172.4, 167.2, 167.0, 155.8, 138.8, 136.4, 129.2, 126.8, 80.0, 71.6, 66.4, 63.0, 62.8, 62.5, 58.7, 42.7, 31.4, 28.4, 19.2, 17.6, 14.1.

**HRMS** (ESI) *m/z* calcd for $C_{27}H_{38}N_2O_8SNa$ [M+Na]$^+$: 573.2247; found: 573.2241.

**Diethyl 5-(4-((((3a*R*,5*R*,6*S*,6a*R*)-5-((*R*)-2,2-dimethyl-1,3-dioxolan-4-yl)-2,2-dimethyltetrahydrofuro[2,3-*d*][1,3]dioxol-6-yl)oxy)carbonyl)phenyl)-2-thioxopyrrolidine-3,3-dicarboxylate (4ah):**

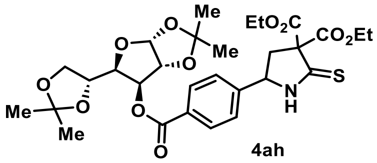

4ah

**Yield:** 61% (74 mg).

**Nature:** Colourless oil.

$R_f$ **value** = 0.43 [EtOAc:Petroleum ether = 1:1 (v/v)].

**$^1$H NMR (400 MHz, CDCl$_3$) *δ* (ppm):** 8.52 (d, *J* = 9.0 Hz, 1H), 8.03 (d, *J* = 7.0 Hz, 2H), 7.42 (d, *J* = 7.0 Hz, 2H), 5.94 (s, 1H), 5.48 (s, 1H), 5.03 (t, *J* = 7.4 Hz, 1H), 4.62 (d, *J* = 1.1 Hz, 1H), 4.31 – 4.22 (m, 4H), 4.25 (q, *J* = 7.1 Hz, 2H), 4.09 (d, *J* = 6.8 Hz, 2H), 3.26 (dd, *J* = 13.4, 7.0 Hz, 1H), 2.82 (dd, *J* = 13.4, 8.2 Hz, 1H), 1.54 (s, 3H), 1.40 (s, 3H), 1.33 – 1.28 (m, 3H), 1.31 (s, 3H), 1.27 – 1.25 (m, 3H), 1.25 (s, 3H).

**$^{13}$C{$^1$H} NMR (101 MHz, CDCl$_3$) *δ* (ppm):** 198.2, 167.1, 166.9, 164.7, 144.4, 130.7, 130.1, 126.7, 112.5, 109.6, 105.2, 83.5, 80.0, 77.0, 72.7, 71.5, 67.4, 63.0, 62.7, 62.3, 42.4, 27.0, 26.8, 26.3, 25.3, 14.1, 14.0.

**HRMS** (ESI) *m/z* calcd for $C_{29}H_{37}NO_{11}SNa$ [M+Na]$^+$: 630.1985; found: 630.1990.

**1,4-Diphenyl-4-thiocyanatobutan-1-one (5a):**

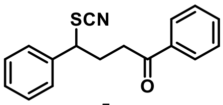

5a

**Yield:** 84% (47 mg).

**Nature:** White solid.

**Mp:** 92 – 94 °C

$R_f$ **value** = 0.34 [EtOAc:Petroleum ether = 1:9 (v/v)].

**$^1$H NMR (400 MHz, CDCl$_3$) *δ* (ppm):** 7.89 (d, *J* = 7.4 Hz, 2H), 7.58 – 7.55 (m, 1H), 7.47 – 7.43 (m, 2H), 7.40 – 7.34 (m, 5H), 4.55 (dd, *J* = 8.5, 7.2 Hz, 1H), 3.07 (t, *J* = 6.9 Hz, 2H), 2.70 – 2.54 (m, 2H).

**$^{13}$C{$^1$H} NMR (101 MHz, CDCl$_3$) *δ* (ppm):** 198.2, 138.1, 136.4, 133.6, 129.4, 129.2, 128.8, 128.1, 127.6, 111.5, 52.7, 35.7, 30.0.

**HRMS** (ESI) *m/z* calcd for $C_{17}H_{15}NOSNa$ [M+Na]$^+$: 304.0772; found: 304.0753.

**Phenyl 4-phenyl-4-thiocyanatobutanoate (5b):**

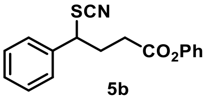

5b

**Yield:** 81% (48 mg).

**Nature:** Colourless oil.

$R_f$ **value** = 0.32 [EtOAc:Petroleum ether = 1:9 (v/v)].

**$^1$H NMR (400 MHz, CDCl$_3$) *δ* (ppm):** 7.45 – 7.36 (m, 7H), 7.26 – 7.22 (m, 1H), 7.06 – 7.03 (m, 2H), 4.54 (dd, *J* = 9.1, 5.6 Hz, 1H), 2.69 – 2.56 (m, 4H).

**$^{13}$C{$^1$H} NMR (101 MHz, CDCl$_3$) *δ* (ppm):** 170.7, 150.5, 137.4, 129.6, 129.5, 129.5, 127.6, 126.2, 121.5, 111.2, 52.6, 31.8, 30.9.

**HRMS** (ESI) *m/z* calcd for $C_{17}H_{15}NO_2SNa$ [M+Na]$^+$: 320.0721; found: 320.0723.

**Ethyl 4-thiocyanato-4-(*p*-tolyl)butanoate (5c):**

**Yield:** 80% (58 mg).

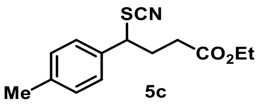

5c

**Nature:** Colourless oil.

**R$_f$ value** = 0.30 [EtOAc:Petroleum ether = 1:9 (v/v)].

**$^1$H NMR (400 MHz, CDCl$_3$) *δ* (ppm):** 7.23 (d, *J* = 8.3 Hz, 2H), 7.19 (d, *J* = 8.2 Hz, 2H), 4.43 (dd, *J* = 8.9, 6.6 Hz, 1H), 4.11 (q, *J* = 7.1 Hz, 2H), 2.54 – 2.40 (m, 2H), 2.39 – 2.30 (m, 2H), 2.35 (s, 3H), 1.24 (t, *J* = 7.2 Hz, 3H).

**$^{13}$C{$^1$H} NMR (101 MHz, CDCl$_3$) *δ* (ppm):** 172.1, 139.3, 134.4, 130.0, 127.5, 111.5, 60.9, 52.4, 31.8, 30.9, 21.3, 14.3.

**HRMS** (ESI) *m/z* calcd for $C_{14}H_{17}NO_2SNa$ [M+Na]$^+$: 286.0878; found: 286.0883.

**4-Phenyl-4-thiocyanatobutanenitrile (5d):**

**Yield:** 82% (33 mg).

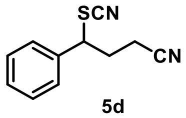

5d

**Nature:** Colourless oil.

**R$_f$ value** = 0.46 [EtOAc:Petroleum ether = 1:4 (v/v)].

**$^1$H NMR (400 MHz, CDCl$_3$) *δ* (ppm):** 7.46 – 7.40 (m, 3H), 7.39 – 7.34 (m, 2H), 4.45 (dd, *J* = 9.0, 6.6 Hz, 1H), 2.63 – 2.55 (m, 1H), 2.53 – 2.44 (m, 2H), 2.38 – 2.29 (m, 1H).

**$^{13}$C{$^1$H} NMR (101 MHz, CDCl$_3$) *δ* (ppm):** 136.0, 129.9, 129.7, 127.4, 117.9, 110.5, 51.1, 31.2, 15.5.

**HRMS** (ESI) *m/z* calcd for $C_{11}H_{10}N_2SNa$ [M+Na]$^+$: 225.0462; found: 225.0457.

**4-(3-Phenyl-3-thiocyanatopropyl)benzonitrile (5e):**

**Yield:** 56% (31 mg).

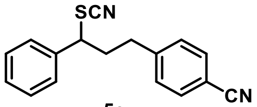

5e

**Nature:** Colourless oil.

**R$_f$ value** = 0.33 [EtOAc:Petroleum ether = 1:9 (v/v)].

**$^1$H NMR (400 MHz, CDCl$_3$) *δ* (ppm):** 7.59 (d, *J* = 8.2 Hz, 2H), 7.44 – 7.37 (m, 3H), 7.34 – 7.32 (m, 2H), 7.24 (d, *J* = 8.2 Hz, 2H), 4.26 (dd, *J* = 8.7, 6.6 Hz, 1H), 2.73 (t, *J* = 7.7 Hz, 2H), 2.59 – 2.44 (m, 2H).

**$^{13}$C{$^1$H} NMR (101 MHz, CDCl$_3$) *δ* (ppm):** 145.5, 137.5, 132.6, 129.5, 129.4 129.3, 127.5, 118.7, 111.3, 110.6, 52.4, 36.7, 33.5.

**HRMS** (ESI) *m/z* calcd for $C_{17}H_{14}NO_2SNa$ [M+Na]$^+$: 301.0775; found: 301.0778.

**Diethyl 2-methyl-2-(2-thiocyanato-2-(*p*-tolyl)ethyl)malonate (5f):**

**Yield:** 84% (59 mg).

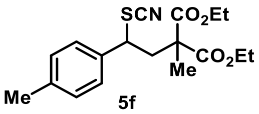

5f

**Nature:** Colourless oil.

**Rf value** = 0.32 [EtOAc:Petroleum ether = 1:9 (v/v)].

**$^1$H NMR (400 MHz, CDCl$_3$) *δ* (ppm):** 7.22 (d, *J* = 8.2 Hz, 2H), 7.16 (d, *J* = 8.0 Hz, 2H), 4.55 (dd, *J* = 9.2, 4.5 Hz, 1H), 4.09 – 4.01 (m, 2H), 3.99 – 3.93 (m, 1H), 3.88 – 3.81 (m, 1H), 2.84 (dd, *J* = 14.7, 9.2 Hz, 1H), 2.72 (dd, *J* = 14.7, 4.5 Hz, 1H), 2.33 (s, 3H), 1.37 (s, 3H), 1.21 (t, *J* = 7.1 Hz, 3H), 1.15 (t, *J* = 7.1 Hz, 3H).

**$^{13}$C{$^1$H} NMR (101 MHz, CDCl$_3$) *δ* (ppm):** 171.2, 171.0, 139.3, 135.0, 129.8, 127.9, 111.7, 61.9, 61.7, 53.1, 50.0, 41.0, 21.3, 20.5, 14.0, 13.9.

**HRMS** (ESI) *m/z* calcd for $C_{18}H_{23}NO_4SNa$ [M+Na]$^+$: 372.1245; found: 372.1250.

**Ethyl 2,2-dimethyl-4-thiocyanato-4-(*p*-tolyl)butanoate (5g):**

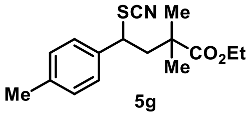

5g

**Yield:** 84% (49 mg).

**Nature:** Colourless oil.

**Rf value** = 0.33 [EtOAc:Petroleum ether = 1:9 (v/v)].

**$^1$H NMR (400 MHz, CDCl$_3$) *δ* (ppm):** 7.22 (d, *J* = 8.1 Hz, 2H), 7.16 (d, *J* = 8.0 Hz, 2H), 4.47 (dd, *J* = 9.4, 4.7 Hz, 1H), 3.89 – 3.89 (m, 1H), 3.79 – 3.71 (m, 1H), 2.55 (dd, *J* = 14.3, 9.4 Hz, 1H), 2.36 (dd, *J* = 14.3, 9.4 Hz, 1H), 2.33 (s, 3H), 1.21 (s, 3H), 1.15 (t, *J* = 7.1 Hz, 3H), 1.11 (s, 3H).

**$^{13}$C{$^1$H} NMR (101 MHz, CDCl$_3$) *δ* (ppm):** 176.3, 139.2, 135.3, 129.8, 127.9, 112.0, 60.9, 50.9, 45.8, 42.2, 26.0, 25.5, 21.3, 14.0.

**HRMS** (ESI) *m/z* calcd for $C_{16}H_{21}NO_2S$ [M]$^+$: 291.1293; found: 291.1290.

**1-Methyl-4-(3-methyl-3-nitro-1-thiocyanatobutyl)benzene (5h):**

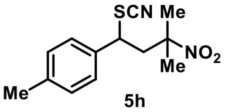

5h

**Yield:** 64% (34 mg).

**Nature:** Colourless oil.

**R$_f$ value** = 0.30 [EtOAc:Petroleum ether = 1:19 (v/v)].

**$^1$H NMR (400 MHz, CDCl$_3$) *δ* (ppm):** 7.22 (d, *J* = 8.4 Hz, 2H), 7.18 (d, *J* = 8.3 Hz, 2H), 4.44 (dd, *J* = 9.8, 4.3 Hz, 1H), 2.94 (dd, *J* = 15.1, 9.8 Hz, 1H), 2.77 (dd, *J* = 15.0, 4.2 Hz, 1H), 2.35 (s, 3H), 1.58 (s, 3H), 1.46 (s, 3H).

**$^{13}$C{$^1$H} NMR (101 MHz, CDCl$_3$) *δ* (ppm):** 139.9, 133.9, 130.2, 127.6, 111.3, 87.1, 49.5, 45.3, 26.6, 26.5, 21.4.

**HRMS** (ESI) *m/z* calcd for $C_{13}H_{16}N_2O_2SNa$ [M+Na]$^+$: 287.0830; found: 287.0832.

**Ethyl 2-fluoro-4-thiocyanato-4-(*p*-tolyl)butanoate (5i):**

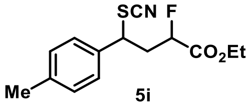

5i

**Yield:** 78% (44 mg, combined yield as a 1.2:1 mixture of diastereomers).

**Nature:** Colourless oil.

**R$_f$ value** = 0.31 [EtOAc:Petroleum ether = 1:19 (v/v)].

**$^1$H NMR (400 MHz, CDCl$_3$) *δ* (ppm):** (for the major isomer) 7.29 – 7.21 (m, 4H), 4.67 – 4.51 (m, 2H), 4.26 (q, *J* = 7.1 Hz, 2H), 2.87 – 2.62 (m, 2H), 2.37 (s, 3H), 1.31 (t, *J* = 7.2 Hz, 3H); (for the minor isomer) 7.29 – 7.21 (m, 4H), 5.17 – 5.02 (m, 1H), 4.60 – 4.51 (m, 1H), 4.18 – 4.09 (m, 2H), 2.87 – 2.62 (m, 2H), 2.36 (s, 3H), 1.28 (t, *J* = 7.2 Hz, 3H).

**$^{13}$C{$^1$H} NMR (101 MHz, CDCl$_3$) *δ* (ppm):** (for the major isomer) 168.5 (d, *J* = 23.4 Hz), 139.6, 134.4, 130.0, 127.4, 110.7, 86.1 (d, *J* = 187.9 Hz), 62.1, 48.6 (d, *J* = 3.8 Hz), 37.9 (d, *J* = 21.3 Hz), 21.3, 14.1; (for the minor isomer) 168.6 (d, *J* = 23.0 Hz), 139.9, 132.7, 130.3, 127.7, 111.0, 86.0 (d, *J* = 187.3 Hz), 62.2, 48.2 (d, *J* = 2.2 Hz), 38.6 (d, *J* = 20.9 Hz), 21.2, 14.2.

**$^{19}$F NMR (377 MHz, CDCl$_3$) δ (ppm):** (for the major isomer) -194.2; (for the minor isomer) -192.9.

**HRMS** (ESI) *m/z* calcd for $C_{14}H_{16}FNO_2SNa$ [M+Na]$^+$: 304.0783; found: 304.0784.

**4-Isothiocyanato-1,4-diphenylbutan-1-one (6a):**

**Yield:** 68% (38 mg).

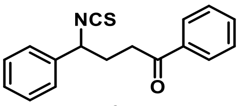


6a

**Nature:** Colourless oil.

**$R_f$ value** = 0.34 [EtOAc:Petroleum ether = 1:19 (v/v)].

**$^1$H NMR (400 MHz, $CDCl_3$) *δ* (ppm):** 7.96 – 7.94 (m, 2H), 7.61 – 7.56 (m, 1H), 7.50 – 7.46 (m, 2H), 7.43 – 7.32 (m, 5H), 4.99 (dd, *J* = 8.4, 5.3 Hz, 1H), 3.23 – 3.05 (m, 2H), 2.44 – 2.28 (m, 2H).

**$^{13}$C{$^1$H} NMR (101 MHz, $CDCl_3$) *δ* (ppm):** 198.6, 138.9, 136.7, 133.6, 133.1, 129.1, 128.7, 128.6, 128.2, 126.0, 61.2, 34.6, 33.3.

**HRMS** (ESI) *m/z* calcd for $C_{17}H_{15}NOSNa$ [M+Na]$^+$: 304.0772; found: 304.0774.

**Phenyl 4-isothiocyanato-4-phenylbutanoate (6b):**

**Yield:** 64% (38 mg).

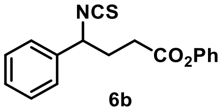


6b

**Nature:** Colourless oil.

**$R_f$ value** = 0.31 [EtOAc:Petroleum ether = 1:19 (v/v)].

**$^1$H NMR (400 MHz, $CDCl_3$) *δ* (ppm):** 7.44 – 7.34 (m, 7H), 7.27 – 7.23 (m, 1H), 7.10 – 7.08 (m, 2H), 4.99 (t, *J* = 6.9 Hz, 1H), 2.81 – 2.66 (m, 2H), 2.33 (dd, *J* = 14.2, 7.2 Hz, 2H).

**$^{13}$C{$^1$H} NMR (101 MHz, $CDCl_3$) *δ* (ppm):** 171.0, 150.6, 138.5, 133.6, 129.6, 129.2, 128.7, 126.1, 126.0, 121.6, 60.9, 33.8, 30.7.

**HRMS** (ESI) *m/z* calcd for $C_{17}H_{15}NO_2SNa$ [M+Na]$^+$: 320.0721; found: 320.0748.

**Ethyl 4-isothiocyanato-4-(*p*-tolyl)butanoate (6c):**

**Yield:** 66% (48 mg).

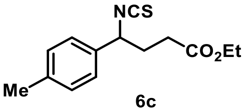


6c

**Nature:** Colourless oil.

**$R_f$ value** = 0.30 [EtOAc:Petroleum ether = 1:19 (v/v)].

**$^1$H NMR (400 MHz, $CDCl_3$) *δ* (ppm):** 7.20 (d, *J* = 8.1 Hz, 2H), 7.18 (d, *J* = 7.4 Hz, 2H), 4.85 (d, *J* = 1.4 Hz, 1H), 4.14 (q, *J* = 7.1 Hz, 2H), 2.48 – 2.41 (m, 2H), 2.35 (s, 3H), 2.22 – 2.16 (m, 2H), 1.26 (t, *J* = 7.1 Hz, 3H).

**$^{13}$C{$^1$H} NMR (101 MHz, $CDCl_3$) *δ* (ppm):** 172.5, 138.5, 135.6, 132.8, 129.8, 126.0, 60.9, 60.8, 34.0, 30.7, 21.3, 14.3.

**HRMS** (ESI) *m/z* calcd for $C_{14}H_{17}N_2OSK$ [M+K]$^+$: 302.0617; found: 302.0614.

**4-Isothiocyanato-4-phenylbutanenitrile (6d):**

**Yield:** 64% (26 mg).

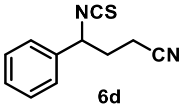


6d

**Nature:** Colourless oil.

**$R_f$ value** = 0.42 [EtOAc:Petroleum ether = 1:9 (v/v)].

**$^1$H NMR (400 MHz, $CDCl_3$) *δ* (ppm):** 7.45 – 7.36 (m, 3H), 7.32 (d, *J* = 6.9 Hz, 2H), 4.94 (dd, *J* = 8.2, 5.6 Hz, 1H), 2.57 – 2.49 (m, 1H), 2.45 – 2.38 (m, 1H), 2.32 – 2.15 (m, 2H).

**$^{13}$C{$^1$H} NMR (101 MHz, $CDCl_3$) *δ* (ppm):** 137.4, 130.2, 129.4, 129.2, 126.0, 118.2, 60.3, 34.4, 14.5.

**HRMS** (ESI) *m/z* calcd for $C_{11}H_{11}NO_2S$ [M+H]$^+$: 203.0643; found: 203.0640.

**4-(3-Isothiocyanato-3-phenylpropyl)benzonitrile (6e):**

**Yield:** 43% (24 mg).

**Nature:** Colourless oil.

**$R_f$ value** = 0.33 [EtOAc:Petroleum ether = 1:19 (v/v)].

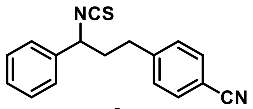

**6e**

**$^1$H NMR (400 MHz, $CDCl_3$) *δ* (ppm):** 7.60 (d, *J* = 8.2 Hz, 2H), 7.42 – 7.34 (m, 3H), 7.33 – 7.27 (m, 4H), 4.73 (dd, *J* = 8.6, 5.1 Hz, 1H), 2.90 – 2.76 (m, 2H), 2.32 – 2.13 (m, 2H).

**$^{13}$C{$^1$H} NMR (101 MHz, $CDCl_3$) *δ* (ppm):** 145.9, 138.6, 133.6, 132.6, 129.4, 129.2, 128.7, 126.0, 119.0, 110.5, 61.0, 40.0, 32.5.

**HRMS** (ESI) *m/z* calcd for $C_{17}H_{14}N_2SNa$ $[M+Na]^+$: 301.0775; found: 301.0772.

**Diethyl 2-(2-isothiocyanato-2-(*p*-tolyl)ethyl)-2-methylmalonate (6f):**

**Yield:** 77% (54 mg).

**Nature:** Colourless oil.

**$R_f$ value** = 0.34 [EtOAc:Petroleum ether = 1:19 (v/v)].

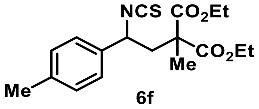

**6f**

**$^1$H NMR (400 MHz, $CDCl_3$) *δ* (ppm):** 7.23 (d, *J* = 8.4 Hz, 2H), 7.19 (d, *J* = 8.2 Hz, 2H), 4.90 (dd, *J* = 10.1, 3.3 Hz, 1H), 4.25 (q, *J* = 7.1 Hz, 2H), 4.22 – 4.16 (m, 2H), 2.50 (dd, *J* = 14.7, 10.1 Hz, 1H), 2.35 (s, 3H), 2.35 – 2.30 (m, 1H), 1.54 (s, 3H), 1.29 (t, *J* = 7.1 Hz, 3H), 1.29 (t, *J* = 7.1 Hz, 3H).

**$^{13}$C{$^1$H} NMR (101 MHz, $CDCl_3$) *δ* (ppm):** 171.6, 171.5, 138.5, 136.9, 132.6, 129.8, 125.9, 62.1, 62.0, 57.9, 52.6, 44.3, 21.3, 20.4, 14.2, 14.1.

**HRMS** (ESI) *m/z* calcd for $C_{18}H_{23}NO_4SNa$ $[M+Na]^+$: 372.1245; found: 372.1224.

**Ethyl 4-isothiocyanato-2,2-dimethyl-4-(*p*-tolyl)butanoate (6g):**

**Yield:** 72% (42 mg).

**Nature:** Colourless oil.

**$R_f$ value** = 0.33 [EtOAc:Petroleum ether = 1:19 (v/v)].

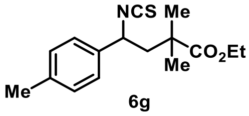

**6g**

**$^1$H NMR (400 MHz, $CDCl_3$) *δ* (ppm):** 7.20 (d, *J* = 8.9 Hz, 2H), 7.18 (d, *J* = 9.1 Hz, 2H), 4.77 (dd, *J* = 9.9, 3.7 Hz, 1H), 4.18 – 4.10 (m, 2H), 2.35 (s, 3H), 2.32 – 2.25 (m, 1H), 1.94 (dd, *J* = 14.4, 3.7 Hz, 1H), 1.30 (s, 3H), 1.28 (t, *J* = 7.2, 3H), 1.26 (s, 3H).

**$^{13}$C{$^1$H} NMR (101 MHz, $CDCl_3$) *δ* (ppm):** 176.8, 138.3, 137.2, 132.2, 129.7, 125.9, 61.2, 58.7, 48.8, 41.5, 26.2, 25.3, 21.2, 18.6, 14.3.

**HRMS** (ESI) *m/z* calcd for $C_{16}H_{21}NO_2S$ $[M]^+$: 291.1293; found: 291.1295.

**1-(1-Isothiocyanato-3-methyl-3-nitrobutyl)-4-methylbenzene (6h):**

**Yield:** 49% (26 mg).

**Nature:** Colourless oil.

**$R_f$ value** = 0.47 [EtOAc:Petroleum ether = 1:19 (v/v)].

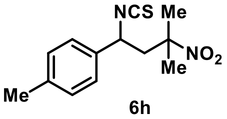

**6h**

**$^1$H NMR (400 MHz, $CDCl_3$) *δ* (ppm):** 7.21 (d, *J* = 9.2 Hz, 2H), 7.19 (d, *J* = 8.9 Hz, 2H), 4.76 (dd, *J* = 9.7, 3.4 Hz, 1H), 2.54 (dd, *J* = 15.2, 9.7 Hz, 1H), 2.42 (dd, *J* = 15.2, 3.4 Hz, 1H), 2.36 (s, 3H), 1.75 (s, 3H), 1.66 (s, 3H).

**$^{13}$C{$^1$H} NMR (101 MHz, $CDCl_3$) *δ* (ppm):** 138.8, 136.2, 134.3, 130.0, 125.8, 86.9, 57.7, 48.5, 27.1, 26.0, 21.2.

**HRMS** (ESI) *m/z* calcd for $C_{13}H_{16}N_2O_2SNa$ $[M+Na]^+$: 287.0830; found: 287.0834.

**Ethyl 2-fluoro-4-isothiocyanato-4-(*p*-tolyl)butanoate (6i):**

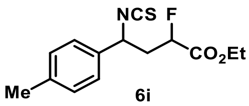

6i

**Yield:** 69% (39 mg, combined yield as a 1.8:1 mixture of diastereomers).

**Nature:** Colourless oil.

**$R_f$ value** = 0.49 [EtOAc:Petroleum ether = 1:19 (v/v)].

**$^1$H NMR (400 MHz, $CDCl_3$) *δ* (ppm):** (for the major isomer) 7.24 (d, *J* = 8.6 Hz, 2H), 7.20 (d, *J* = 9.5 Hz, 2H), 4.93 (t, *J* = 7.4 Hz, 1H), 4.80 – 4.65 (m, 1H), 4.29 (q, *J* = 7.2 Hz, 2H), 2.66 – 2.43 (m, 1H), 2.37 (s, 3H), 2.42 – 2.21 (m, 1H), 1.32 (t, *J* = 7.2 Hz, 1H); (for the minor isomer) 7.24 (d, *J* = 8.6 Hz, 2H), 7.20 (d, *J* = 9.5 Hz, 2H), 5.23 – 5.07 (m, 1H), 5.02 (dd, *J* = 10.9, 3.6 Hz, 1H), 4.26 – 4.20 (m, 1H), 2.66 – 2.43 (m, 1H), 2.36 (s, 1H), 2.42 – 2.21 (m, 1H), 1.30 (t, *J* = 7.1 Hz, 1H).

**$^{13}$C{$^1$H} NMR (101 MHz, $CDCl_3$) *δ* (ppm):** (for the major isomer) 168.8 (d, *J* = 23.3 Hz), 139.1, 134.6, 134.3, 130.0 126.3, 85.8 (d, *J* = 186.9 Hz), 62.3, 57.2 (d, *J* = 3.7 Hz), 40.8 (d, *J* = 20.5 Hz), 21.3, 14.3; (for the minor isomer) 168.9 (d, *J* = 22.9 Hz), 138.9, 135.2, 134.3, 129.9, 125.9, 85.9 (d, *J* = 186.4 Hz), 62.2, 57.4 (d, *J* = 2.6 Hz), 41.5 (d, *J* = 20.6 Hz), 21.2, 14.2.

**$^{19}$F NMR (377 MHz, $CDCl_3$) δ (ppm):** (for the major isomer) -193.9; (for the minor isomer) -194.0.

**HRMS** (ESI) *m/z* calcd for $C_{14}H_{16}FNO_2SNa$ [M+Na]$^+$: 304.0783; found: 304.0777.

**Diethyl 2-imino-5-phenyldihydroselenophene-3,3(2*H*)-dicarboxylate (7a):**

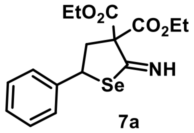

7a

**Yield:** 69% (51 mg).

**Nature:** Colourless oil.

**$R_f$ value** = 0.30 [EtOAc:Petroleum ether = 1:4 (v/v)].

**$^1$H NMR (400 MHz, DMSO-$d_6$) *δ* (ppm):** 11.46 (brs, 1H), 7.47 (d, *J* = 7.5 Hz, 2H), 7.38 – 7.34 (m, 2H), 7.31 – 7.27 (m, 1H), 4.93 (dd, *J* = 11.0, 5.3 Hz, 1H), 4.27 (q, *J* = 7.1 Hz, 2H), 4.19 – 4.12 (m, 2H), 2.94 – 2.85 (m, 2H), 1.24 (t, *J* = 7.1 Hz, 3H), 1.18 (t, *J* = 7.1 Hz, 3H).

**$^{13}$C{$^1$H} NMR (101 MHz, DMSO-$d_6$) *δ* (ppm):** 170.1, 167.2, 166.8, 139.3, 128.9, 127.9, 127.8, 73.9, 62.0, 61.9, 46.0, 44.4, 13.9.

**HRMS** (ESI) *m/z* calcd for $C_{16}H_{20}NO_4Se$ [M+H]$^+$: 370.0558; found: 370.0568.

**Diethyl 2-imino-5-(*p*-tolyl)dihydroselenophene-3,3(2*H*)-dicarboxylate (7b):**

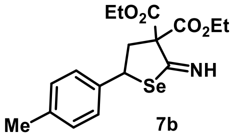

7b

**Yield:** 72% (55 mg).

**Nature:** Pale yellow oil.

**$R_f$ value** = 0.31 [EtOAc:Petroleum ether = 1:4 (v/v)].

**$^1$H NMR (400 MHz, DMSO-$d_6$) *δ* (ppm):** 11.43 (brs, 1H), 7.35 (d, *J* = 8.0 Hz, 2H), 7.16 (d, *J* = 8.0 Hz, 2H), 4.89 (dd, *J* = 9.9, 6.5 Hz, 1H), 4.26 (q, *J* = 7.1 Hz, 2H), 4.20 – 4.12 (m, 2H), 2.91 – 2.83 (m, 2H), 2.28 (s, 3H), 1.23 (t, *J* = 7.1 Hz, 3H), 1.18 (t, *J* = 7.1 Hz, 3H).

**$^{13}$C{$^1$H} NMR (101 MHz, DMSO-$d_6$) *δ* (ppm):** 170.2, 167.1, 166.7, 137.2, 136.2, 129.4, 127.6, 73.9, 61.9, 61.8, 45.9, 44.5, 20.7, 13.8.

**HRMS** (ESI) *m/z* calcd for $C_{17}H_{22}NO_4Se$ [M+H]$^+$: 384.0714; found: 384.0707.

**Diethyl 5-(4-chlorophenyl)-2-iminodihydroselenophene-3,3(2*H*)-dicarboxylate (7c):**

**Yield:** 67% (57 mg).

**Nature:** Pale yellow oil.

**R$_f$ value** = 0.30 [EtOAc:Petroleum ether = 1:4 (v/v)].

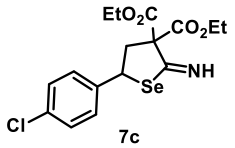


7c

**$^1$H NMR (400 MHz, DMSO-d$_6$) $\delta$ (ppm):** 11.48 (brs, 1H), 7.51 (d, $J$ = 8.5 Hz, 2H), 7.41 (d, $J$ = 8.5 Hz, 2H), 4.96 (dd, $J$ = 11.8, 4.8 Hz, 1H), 4.26 (q, $J$ = 7.1 Hz, 2H), 4.19 – 4.11 (m, 2H), 2.95 – 2.81 (m, 2H), 1.23 (t, $J$ = 7.1 Hz, 3H), 1.18 (t, $J$ = 7.1 Hz, 3H).

**$^{13}$C{$^1$H} NMR (101 MHz, DMSO-d$_6$) $\delta$ (ppm):** 170.4, 167.0, 166.6, 138.5, 132.3, 129.8, 128.8, 73.8, 62.0, 61.9, 45.0, 44.3, 13.8.

**HRMS** (ESI) *m/z* calcd for $C_{16}H_{19}ClNO_4Se$ [M+H]$^+$: 404.0168; found: 404.0161.

**Diethyl 2-imino-5-(naphthalen-2-yl)dihydroselenophene-3,3(2*H*)-dicarboxylate (7d):**

**Yield:** 49% (41 mg).

**Nature:** Pale yellow oil.

**R$_f$ value** = 0.31 [EtOAc:Petroleum ether = 1:4 (v/v)].

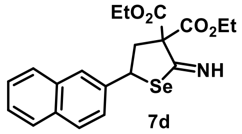


7d

**$^1$H NMR (400 MHz, DMSO-d$_6$) $\delta$ (ppm):** 11.49 (brs, 1H), 8.01 (s, 1H), 7.93 – 7.89 (m, 3H), 7.61 (d, $J$ = 8.5 Hz, 1H), 7.54 – 7.49 (m, 2H), 5.12 (dd, $J$ = 10.6, 5.9 Hz, 1H), 4.33 – 4.26 (m, 2H), 4.20 – 4.12 (m, 2H), 3.07 – 2.99 (m, 2H), 1.26 (t, $J$ = 7.1 Hz, 3H), 1.19 (t, $J$ = 7.1 Hz, 3H).

**$^{13}$C{$^1$H} NMR (101 MHz, CDCl$_3$) $\delta$ (ppm):** 170.3, 167.1, 166.7, 136.7, 132.9, 132.5, 128.6, 127.8, 127.6, 126.5, 126.4, 126.3, 125.7, 73.9, 62.1, 61.9, 46.3, 44.2, 13.9, 13.8.

**HRMS** (ESI) *m/z* calcd for $C_{20}H_{22}NO_4Se$ [M+H]$^+$: 420.0714; found: 420.0708.

**Diethyl 2-imino-5-(thiophen-3-yl)dihydroselenophene-3,3(2*H*)-dicarboxylate (7e):**

**Yield:** 64% (48 mg).

**Nature:** Pale yellow oil.

**R$_f$ value** = 0.33 [EtOAc:Petroleum ether = 1:4 (v/v)].

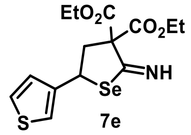


7e

**$^1$H NMR (400 MHz, DMSO-d$_6$) $\delta$ (ppm):** 11.43 (brs, 1H), 7.55 (s, 1H), 7.55 – 7.54 (m, 1H), 7.20 – 7.18 (m, 1H), 5.04 (dd, $J$ = 11.3, 4.7 Hz, 1H), 4.25 (q, $J$ = 7.1 Hz, 2H), 4.19 – 4.11 (m, 2H), 2.98 – 2.86 (m, 2H), 1.23 (t, $J$ = 7.1 Hz, 3H), 1.18 (t, $J$ = 7.1 Hz, 3H).

**$^{13}$C{$^1$H} NMR (101 MHz, DMSO-d$_6$) $\delta$ (ppm):** 170.1, 167.1, 166.8, 140.3, 127.2, 127.1, 122.9, 73.7, 62.0, 61.8, 44.0, 40.9, 13.8.

**HRMS** (ESI) *m/z* calcd for $C_{14}H_{18}NO_4SSe$ [M+H]$^+$: 376.0122; found: 376.0119.

**Diisopropyl 2-imino-5-phenyldihydroselenophene-3,3(2*H*)-dicarboxylate (7f):**

**Yield:** 63% (50 mg).

**Nature:** Pale yellow oil.

**R$_f$ value** = 0.33 [EtOAc:Petroleum ether = 1:4 (v/v)].

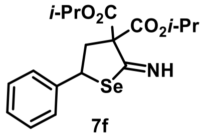


7f

**$^1$H NMR (400 MHz, DMSO-d$_6$) *δ* (ppm):** 11.42 (brs, 1H), 7.46 (d, *J* = 7.4 Hz, 2H), 7.38 – 7.34 (m, 2H), 7.31 – 7.27 (m, 1H), 5.10 – 5.03 (m, 1H), 4.98 – 4.88 (m, 2H), 2.92 – 2.82 (m, 2H), 1.26 (d, *J* = 6.3 Hz, 3H), 1.23 (d, *J* = 6.2 Hz, 3H), 1.20 (d, *J* = 6.2 Hz, 3H), 1.16 (d, *J* = 6.2 Hz, 3H).

**$^{13}$C{$^1$H} NMR (101 MHz, DMSO-d$_6$) *δ* (ppm):** 170.1, 166.6, 166.5, 139.4, 128.9, 127.9, 127.8, 73.6, 69.6, 69.5, 45.9, 44.4, 21.4, 21.3, 21.2.

**HRMS** (ESI) *m/z* calcd for $C_{18}H_{23}NO_4SeNa$ [M+Na]$^+$: 420.0690; found: 420.0688.

**Synthesis of 1-(4-Chlorophenyl)-4-phenyl-4-selenocyanatobutan-1-one (7g):**

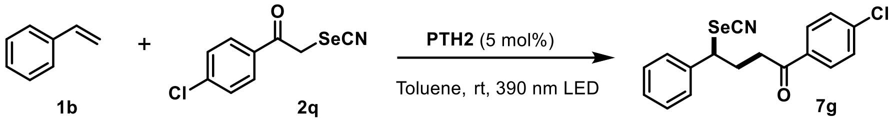


An oven-dried culture tube equipped with a magnetic stir bar was charged with **PTH2** (3 mg, 0.01 mmol, 5 mol%), 1-(4-chlorophenyl)-2-selenocyanatoethan-1-one **2q** (52 mg, 0.2 mmol) and dry toluene (2 mL). The tube was sealed with a Teflon screw cap before styrene **1b** (46 µL, 0.4 mmol) was added to it. Then, the reaction mixture was degassed by Freeze-Pump-Thaw cycles with argon and irradiated at rt with 390 nm LEDs at a distance of approximately 5 cm for 30 min. A high-speed fan was used to maintain the temperature. After the completion of the reaction (confirmed by TLC), reaction crude was concentrated and purified by silica gel column chromatography using EtOAc/petroleum ether as eluent to afford the corresponding alkylselenocyanation product **7g**.

**1-(4-Chlorophenyl)-4-phenyl-4-selenocyanatobutan-1-one (7g):**

**Yield:** 83% (60 mg).

**Nature:** Low melting solid

**R$_f$ value** = 0.33 [EtOAc:Petroleum ether = 1:9 (v/v)].

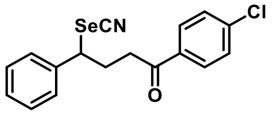


7g

**$^1$H NMR (400 MHz, CDCl$_3$) *δ* (ppm):** 7.82 (d, *J* = 8.7 Hz, 2H), 7.43 – 7.33 (m, 5H), 7.41 (d, *J* = 8.7 Hz, 2H), 4.79 (dd, *J* = 8.9, 7.0 Hz, 1H), 3.05 – 3.02 (m, 2H), 2.80 – 2.70 (m, 2H).

**$^{13}$C{$^1$H} NMR (101 MHz, CDCl$_3$) *δ* (ppm):** 197.1, 140.1, 138.5, 134.8, 129.5, 129.5, 129.3, 129.2, 127.6, 102.2, 50.2, 36.7, 30.8.

**HRMS** (ESI) *m/z* calcd for $C_{17}H_{18}ClN_2OSe$ [M+NH$_4$]$^+$: 381.0273; found: 381.0250.

**Diethyl 5-phenyl-2-selenoxopyrrolidine-3,3-dicarboxylate (8a):**

**Yield:** 61% (45 mg).

**Nature:** Low melting solid.

**R$_f$ value** = 0.34 [EtOAc:Petroleum ether = 1:4 (v/v)].

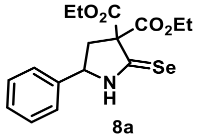


8a

**$^1$H NMR (400 MHz, CDCl$_3$) *δ* (ppm):** 9.55 (brs, 1H), 7.40 – 7.30 (m, 5H), 4.80 (t, *J* = 7.4 Hz, 1H), 4.33 – 4.27 (m, 2H), 4.26 (q, *J* = 7.2 Hz, 2H), 3.22 (dd, *J* = 13.4, 6.9 Hz, 1H), 2.86 (dd, *J* = 13.4, 8.5 Hz, 1H), 1.31 (t, *J* = 7.2 Hz, 3H), 1.28 (t, *J* = 7.2 Hz, 3H).

**$^{13}$C{$^1$H} NMR (101 MHz, CDCl$_3$) *δ* (ppm):** 200.3, 166.7, 166.5, 137.7, 129.2, 129.0, 126.6, 74.9, 65.6, 62.9, 62.8, 42.4, 14.0.

**HRMS** (ESI) *m/z* calcd for $C_{16}H_{20}NO_4Se$ [M+H]$^+$: 370.0558; found: 370.0555.

**Diethyl 2-selenoxo-5-(*p*-tolyl)pyrrolidine-3,3-dicarboxylate (8b):**

**Yield:** 64% (49 mg).

**Nature:** Low melting solid.

**$R_f$ value** = 0.35 [EtOAc:Petroleum ether = 1:4 (v/v)].

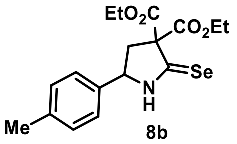

8b

**$^1$H NMR (400 MHz, DMSO-d$_6$) *δ* (ppm):** 12.01 (brs, 1H), 7.21 (d, *J* = 8.2 Hz, 2H), 7.17 (d, *J* = 8.1 Hz, 2H), 4.82 (t, *J* = 7.4 Hz, 1H), 4.25 – 4.16 (m, 2H), 4.15 – 4.08 (m, 2H), 3.12 (dd, *J* = 13.3, 7.2 Hz, 1H), 2.61 (dd, *J* = 13.3, 7.6 Hz, 1H), 2.30 (s, 3H), 1.23 (t, *J* = 7.1 Hz, 3H), 1.16 (t, *J* = 7.1 Hz, 3H).

**$^{13}$C{$^1$H} NMR (101 MHz, DMSO-d$_6$) *δ* (ppm):** 197.6, 166.7, 166.4, 137.6, 135.9, 129.3, 126.3, 74.8, 64.5, 62.0, 61.8, 41.8, 20.7, 13.8, 13.7.

**HRMS** (ESI) *m/z* calcd for $C_{17}H_{22}NO_4Se$ [M+H]$^+$: 384.0714; found: 384.0699.

**Diethyl 5-(4-chlorophenyl)-2-selenoxopyrrolidine-3,3-dicarboxylate (8c):**

**Yield:** 62% (50 mg).

**Nature:** Low melting solid**.**

**$R_f$ value** = 0.38 [EtOAc:Petroleum ether = 1:4 (v/v)].

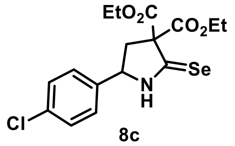

8c

**$^1$H NMR (400 MHz, DMSO-d$_6$) *δ* (ppm):** 12.06 (brs, 1H), 7.48 (d, *J* = 8.5 Hz, 2H), 7.32 (d, *J* = 8.5 Hz, 2H), 4.91 (t, *J* = 7.3 Hz, 1H), 4.24 – 4.17 (m, 2H), 4.15 – 4.07 (m, 2H), 3.16 (dd, *J* = 13.4, 7.4 Hz, 1H), 2.61 (dd, *J* = 13.4, 7.2 Hz, 1H), 1.23 (t, *J* = 7.1 Hz, 3H), 1.14 (t, *J* = 7.1 Hz, 3H).

**$^{13}$C{$^1$H} NMR (101 MHz, DMSO-d$_6$) *δ* (ppm):** 198.1, 166.6, 166.3, 137.9, 132.8, 128.7, 128.3, 74.8, 63.9, 62.0, 61.9, 41.6, 13.8, 13.7.

**HRMS** (ESI) *m/z* calcd for $C_{16}H_{18}ClNO_4SeNa$ [M+Na]$^+$: 425.9987; found: 425.9979.

**Diethyl 5-(naphthalen-2-yl)-2-selenoxopyrrolidine-3,3-dicarboxylate (8d):**

**Yield:** 44% (37 mg).

**Nature:** Low melting solid.

**$R_f$ value** = 0.38 [EtOAc:Petroleum ether = 1:4 (v/v)].

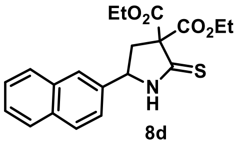

8d

**$^1$H NMR (400 MHz, DMSO-d$_6$) *δ* (ppm):** 12.16 (brs, 1H), 7.98 (d, *J* = 8.6 Hz, 1H), 7.94 – 7.91 (m, 2H), 7.83 (s, 1H), 7.57 – 7.52 (m, 2H), 7.43 (dd, *J* = 8.5, 1.4 Hz, 1H), 5.06 (t, *J* = 7.3 Hz, 1H), 4.28 – 4.20 (m, 2H), 4.14 – 4.05 (m, 2H), 3.23 (dd, *J* = 13.4, 7.3 Hz, 1H), 2.75 (dd, *J* = 13.4, 7.5 Hz, 1H), 1.25 (t, *J* = 7.1 Hz, 3H), 1.13 (t, *J* = 7.1 Hz, 3H).

**$^{13}$C{$^1$H} NMR (101 MHz, DMSO-d$_6$) *δ* (ppm):** 198.0, 166.7, 166.4, 136.3, 132.6, 128.7, 127.8, 127.7, 126.7, 126.4, 125.3, 124.1, 74.9, 64.8, 62.0, 61.9, 41.6, 13.8, 13.7.

**HRMS** (ESI) *m/z* calcd for $C_{20}H_{22}NO_4Se$ [M+H]$^+$: 420.0714; found: 420.0700.

**Diethyl 2-selenoxo-5-(thiophen-3-yl)pyrrolidine-3,3-dicarboxylate (8e):**

**Yield:** 56% (42 mg).

**Nature:** Low melting solid.

**$R_f$ value** = 0.39 [EtOAc:Petroleum ether = 1:4 (v/v)].

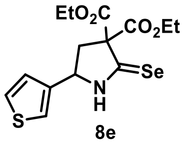

8e

**$^1$H NMR (400 MHz, CDCl$_3$) $\delta$ (ppm):** 9.38 (brs, 1H), 7.37 (dd, $J$ = 4.9, 3.0 Hz, 1H), 7.28 (s, 1H), 7.07 (d, $J$ = 5.0 Hz, 1H), 4.92 (t, $J$ = 7.5 Hz, 1H), 4.34 – 4.25 (m, 2H), 4.27 (q, $J$ = 7.1 Hz, 2H), 3.22 (dd, $J$ = 13.3, 6.9 Hz, 1H), 2.92 (dd, $J$ = 13.3, 8.2 Hz, 1H), 1.32 (t, $J$ = 7.1 Hz, 3H), 1.29 (t, $J$ = 7.1 Hz, 3H).

**$^{13}$C{$^1$H} NMR (101 MHz, CDCl$_3$) $\delta$ (ppm):** 200.0, 166.8, 166.5, 138.4, 127.8, 125.6, 123.4, 74.8, 63.0, 62.9, 61.3, 41.5, 14.1.

**HRMS** (ESI) *m/z* calcd for $C_{14}H_{18}NO_4SSe$ [M+H]$^+$: 376.0122; found: 376.0107.

**Diisopropyl 5-phenyl-2-selenoxopyrrolidine-3,3-dicarboxylate (8f):**

**Yield:** 58% (46 mg).

**Nature:** Low melting solid.

**R$_f$ value** = 0.42 [EtOAc:Petroleum ether = 1:4 (v/v)].

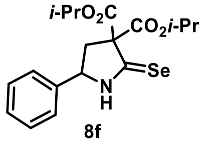

8f

**$^1$H NMR (400 MHz, DMSO-d$_6$) $\delta$ (ppm):** 12.00 (brs, 1H), 7.42 – 7.38 (m, 2H), 7.35 – 7.31 (m, 1H), 7.29 (d, $J$ = 7.1 Hz, 2H), 4.05 – 4.98 (m, 1H), 4.91 – 4.85 (m, 2H), 3.14 (dd, $J$ = 13.3, 7.5 Hz, 1H), 2.62 (dd, $J$ = 13.3, 6.9 Hz, 1H), 1.25 (d, $J$ = 6.3 Hz, 3H), 1.23 (d, $J$ = 6.3 Hz, 3H), 1.20 (d, $J$ = 6.2 Hz, 3H), 1.09 (d, $J$ = 6.2 Hz, 3H).

**$^{13}$C{$^1$H} NMR (101 MHz, DMSO-d$_6$) $\delta$ (ppm):** 198.1, 166.1, 165.9, 139.1, 128.7, 128.1, 126.3, 74.4, 69.6, 69.5, 64.4, 41.6, 21.4, 21.3, 21.2, 21.0.

**HRMS** (ESI) *m/z* calcd for $C_{18}H_{23}NO_4SeNa$ [M+Na]$^+$: 420.0690; found: 420.0685.

**Synthesis 1-(4-Chlorophenyl)-4-isoselenocyanato-4-phenylbutan-1-one (8g):**

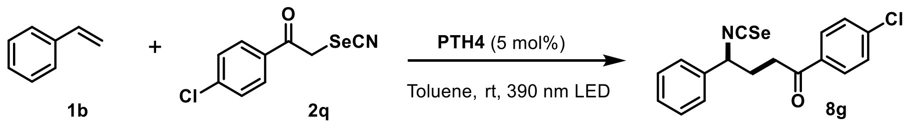


An oven-dried culture tube equipped with a magnetic stir bar was charged with **PTH4** (3.7 mg, 0.01 mmol, 5 mol%), 1-(4-chlorophenyl)-2-selenocyanatoethan-1-one **2q** (52 mg 0.2 mmol) and dry toluene (2 mL). The tube was sealed with a Teflon screw cap before styrene **1b** (46 µL, 0.4 mmol) was added to it. Then, the reaction mixture was degassed by Freeze-Pump-Thaw cycles with argon and irradiated at rt with 390 nm LEDs at a distance of approximately 5 cm for 24 h. A high-speed fan was used to maintain the temperature. After the completion of the reaction (confirmed by TLC), reaction crude was concentrated and purified by silica gel column chromatography using EtOAc/petroleum ether as eluent to afford the corresponding alkylisoselenocyanation product **8g**.

**1-(4-Chlorophenyl)-4-isoselenocyanato-4-phenylbutan-1-one (8g):**

**Yield:** 68% (49 mg).

**Nature:** Pale yellow oil.

**R$_f$ value** = 0.31 [EtOAc:Petroleum ether = 1:19 (v/v)].

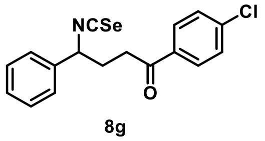

8g

**$^1$H NMR (400 MHz, CDCl$_3$) $\delta$ (ppm):** 7.88 (d, $J$ = 8.6 Hz, 2H), 7.45 (d, $J$ = 8.6 Hz, 2H), 7.40 (d, $J$ = 6.8 Hz, 2H), 7.37 – 7.34 (m, 3H), 5.06 (dd, $J$ = 8.4, 5.4 Hz, 1H), 3.21 – 3.02 (m, 2H), 2.46 – 2.31 (m, 2H).

**$^{13}C\{^1H\}$ NMR (101 MHz, $CDCl_3$) $\delta$ (ppm):** 197.1, 140.1, 137.6, 134.9, 129.6, 129.3, 129.2, 128.9, 128.2, 126.0, 61.6, 34.4, 33.0.

**HRMS** (ESI) *m/z* calcd for $C_{17}H_{15}ClNOSe$ $[M+H]^+$: 364.0007; found: 364.0008.

## 6. Photocatalytic Three-Component Chemodivergent Reaction:

### Procedure of Photocatalytic 2-Imino-Tetrahydrothiophene Synthesis by Three-Component Reaction:

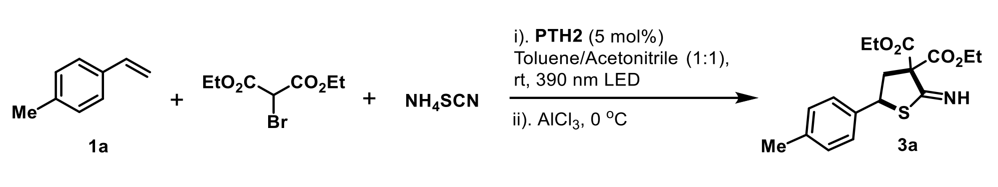


An oven-dried culture tube equipped with a magnetic stir bar was charged with **PTH2** (3 mg, 0.01 mmol, 5 mol%), diethyl 2-bromomalonate (34 µL, 0.2 mmol), ammonium thiocyanate (30 mg, 0.4 mmol), and dry toluene/acetonitrile (1:1) (2 mL). The tube was sealed with a Teflon screw cap before 4-methylstyrene **1a** (53 µL, 0.4 mmol) was added to it. Then, the reaction mixture was degassed by Freeze-Pump-Thaw cycles with argon and irradiated at rt with 390 nm LEDs at a distance of approximately 5 cm for 1 h. A high-speed fan was used to maintain the temperature. After the consumption of bromomalonate (confirmed by TLC), Aluminum chloride (27 mg, 0.2 mmol) was added to the ice-cold reaction mixture. After 1 h, 2 mL of ethyl acetate was added and quenched with saturated ammonium chloride solution (2 mL). The crude reaction mixture was extracted with ethyl acetate (2×2 mL), washed with brine (3 mL), and dried over anhydrous $Na_2SO_4$. The organic portion was concentrated, and the residue was purified by silica gel column chromatography using EtOAc/petroleum ether as eluent to afford the corresponding 2-imino-tetrahydrothiophene product **3a** with 63% (42 mg) yield.

### Procedure of Photocatalytic Thiopyrrolidinones Synthesis by Three-Component Reaction:

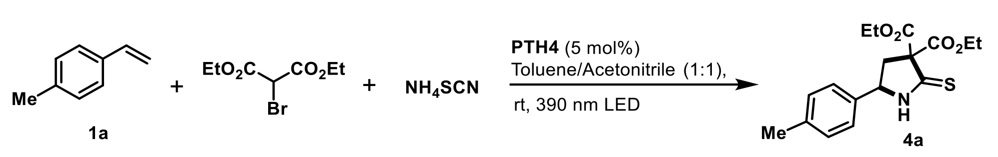


An oven-dried culture tube equipped with a magnetic stir bar was charged with **PTH4** (3.7 mg, 0.01 mmol, 5 mol%), diethyl 2-bromomalonate (34 µL, 0.2 mmol), ammonium thiocyanate (30 mg, 0.4 mmol), and dry toluene/acetonitrile (1:1) (2 mL). The tube was sealed with a Teflon screw cap before 4-methylstyrene **1a** (53 µL, 0.4 mmol) was added to it. Then, the reaction mixture was degassed by Freeze-Pump-Thaw cycles with argon and irradiated at rt with 390 nm LEDs at a distance of approximately 5 cm 36 h. A high-speed fan was used to maintain the temperature. After the completion of the reaction (confirmed by TLC), reaction crude was concentrated and purified by silica gel column chromatography using EtOAc/petroleum ether as eluent to afford the corresponding thiopyrrolidinone product **4a** with 57% (38 mg) yield.

## 7. Synthetic Transformations of Photochemical Chemodivergent Products:

### Hydrolysis to Thiolactone 9:

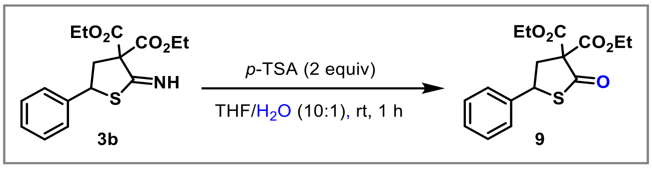


Compound **3b** (64 mg, 0.2 mmol) was added to a mixture of THF–$H_2O$ (2 mL, 10:1) and *p*-toluenesulfonic acid (69 mg, 0.4 mmol) and the reaction mixture was vigorously stirred at rt for 1 h. After completion of the reaction, the crude was concentrated under vacuo, and the residue was purified by silica gel column chromatography using EtOAc/petroleum ether as eluent to afford the corresponding product **9**.

**Diethyl 2-oxo-5-phenyldihydrothiophene-3,3(2*H*)-dicarboxylate (9):**

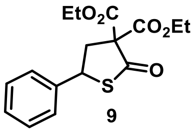


**Yield:** 87% (56 mg).

**Nature:** Colourless oil.

**$R_f$ value** = 0.33 [EtOAc:Petroleum ether = 1:9 (v/v)].

**$^1$H NMR (400 MHz, $CDCl_3$) $\delta$ (ppm):** 7.44 (d, *J* = 7.2 Hz, 2H), 7.40 – 7.37 (m, 2H), 7.35 – 7.32 (m, 1H), 4.90 (dd, *J* = 11.4, 5.1 Hz, 1H), 4.41 – 4.32 (m, 2H), 4.27 (q, *J* = 7.1 Hz, 2H), 3.19 (dd, *J* = 13.3, 5.1 Hz, 1H), 2.93 (dd, *J* = 13.3, 11.4 Hz, 1H), 1.35 (t, *J* = 7.1 Hz, 3H), 1.29 (t, *J* = 7.1 Hz, 3H).

**$^{13}C\{^1H\}$ NMR (101 MHz, $CDCl_3$) $\delta$ (ppm):** 197.5, 165.8, 165.4, 137.6, 129.2, 128.8, 127.8, 73.8, 63.3, 63.1, 50.4, 43.5, 14.1.

**HRMS** (ESI) *m/z* calcd for $C_{16}H_{18}O_5SNa$ $[M+Na]^+$: 345.0773; found: 345.0765.

### Decarboalkoxylation-isomerization to 2-Amino-dihydrothiophene 10:[22]

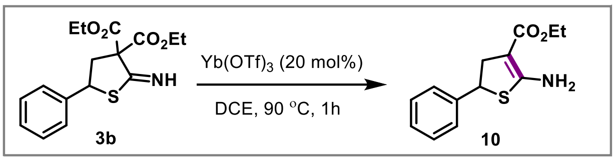


In a culture tube, compound **3b** (64.3 mg, 0.2 mmol) and ytterbium(III) trifluoromethanesulfonate (25 mg, 0.04 mmol) were added. Then, 3 mL of DCE was added and heated at 90 °C for 1 h. After completion of the reaction, the crude was concentrated under vacuo, and the residue was purified by silica gel column chromatography using EtOAc/petroleum ether as eluent to afford the corresponding product **10**.

**Ethyl 2-amino-5-phenyl-4,5-dihydrothiophene-3-carboxylate (10):**

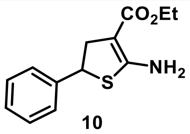


**Yield:** 84% (42 mg).

**Nature:** White solid.

**$R_f$ value** = 0.30 [EtOAc:Petroleum ether = 1:9 (v/v)].

**$^{1}H$ NMR (400 MHz, $CDCl_3$) *δ* (ppm):** 7.44 – 7.41 (m, 2H), 7.35 – 7.31 (m, 2H), 7.29 – 7.27 (m, 1H), 6.06 (brs, 2H), 4.86 (t, *J* = 8.1 Hz, 1H), 4.18 – 4.12 (m, 2H), 3.40 (dd, *J* = 14.3, 8.5 Hz, 1H), 3.16 (dd, *J* = 14.3, 7.7 Hz, 1H), 1.26 (t, *J* = 7.1 Hz, 3H).

**$^{13}C\{^{1}H\}$ NMR (101 MHz, $CDCl_3$) *δ* (ppm):** 166.5, 162.1, 141.6, 128.8, 127.9, 127.4, 91.2, 59.2, 51.5, 41.8, 14.8.

### Cyclization to Spiro Thia-oxazete 11:

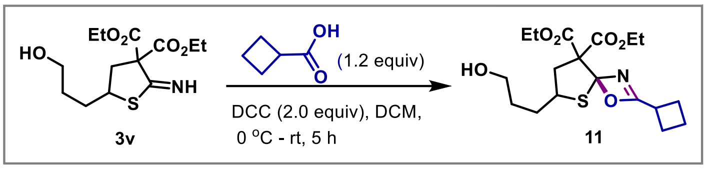


Cyclobutanecarboxylic acid (12 µL, 0.12 mmol) and *N*,*N′*-Dicyclohexylcarbodiimide (DCC) (42 mg, 0.2 mmol) were sequentially added to a solution of **3v** (34 mg, 0.1 mmol) in $CH_2Cl_2$ (2 mL) at 0 °C and the reaction was stirred for 5 h with warming to rt. After the completion of the reaction, the crude product was filtered and washed with $CH_2Cl_2$ (5×2 mL). The filtrate was washed sequentially with saturated $NaHCO_3$ solution and brine (2 mL each). The combined organic extracts were dried over anhydrous $Na_2SO_4$, filtered, and concentrated under vacuo. Then, the residue was purified by silica gel column chromatography (230−400 mesh) using EtOAc/petroleum ether as eluent to afford the corresponding spiro thia-oxazete **11**.

**Diethyl 2-cyclobutyl-6-(3-hydroxypropyl)-1-oxa-5-thia-3-azaspiro[3.4]oct-2-ene-8,8-dicarboxylate (11):**

**Yield:** 78% (30 mg, combined yield as a 5:1 mixture of diastereomers).

**Nature:** Colourless oil.

**$R_f$ value** = 0.42 [EtOAc:Petroleum ether = 3:7 (v/v)].

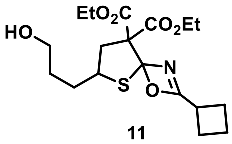


**$^{1}H$ NMR (400 MHz, $CDCl_3$) *δ* (ppm):** (for the mixture) 4.27 – 4.18 (m, 4H), 4.11 (t, *J* = 5.4 Hz, 2H), 3.70 – 3.58 (m, 1H), 3.18 – 3.06 (m, 2H), 2.46 – 2.39 (m, 1H), 2.30 – 2.15 (m, 5H), 2.03 – 1.83 (m, 4H), 1.82 – 1.69 (m, 1H), 1.29 (t, *J* = 7.1 Hz, 3H), 1.28 (t, *J* = 7.1 Hz, 3H).

**$^{13}C\{^{1}H\}$ NMR (101 MHz, $CDCl_3$) *δ* (ppm):** (for the mixture) 175.6, 175.5, 168.6, 168.5, 168.4, 168.4, 109.8, 109.7, 63.5, 63.3, 62.2, 62.1, 62.0, 61.9, 49.7, 49.5, 48.7, 48.2, 38.1, 34.4, 32.6, 31.3, 29.6, 29.3, 26.4, 26.3, 25.4, 18.5, 14.2, 14.1.

**HRMS** (ESI) *m/z* calcd for $C_{18}H_{27}NO_6SNa$ $[M+Na]^+$: 408.1457; found: 408.1436.

### Cyclization to Bridged Thiabicycle 12:

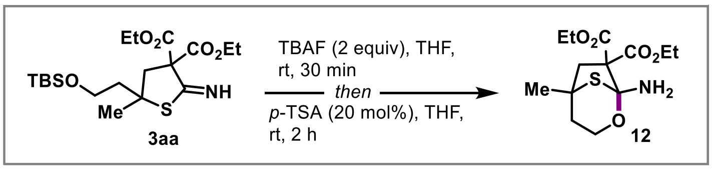


Tetra-*n*-butylammonium fluoride solution (1M in THF) (0.2 mL, 0.2 mmol) was added to ice-cold solution of 2-iminotetrahydrothiophene **3aa** (42 mg, 0.1 mmol) in dry THF (2 mL) and the reaction stirred in rt for 30 min. Then *p*-toluenesulfonic acid (4 mg, 0.02 mmol) was added to the mixture. The progress of the

reaction was monitored by TLC. After the completion of the reaction, the reaction was concentrated in vacuo and quenched with water (1 mL). The aqueous layer was extracted with ethyl acetate (3×3 mL each), and the combined organic layers were washed with brine (5 mL), dried with anhydrous $Na_2SO_4$, filtered, and concentrated under vacuo. Then, the residue was purified by silica gel column chromatography (230−400 mesh) using EtOAc/petroleum ether as eluent to afford the corresponding product **12**.

**Diethyl 1-amino-5-methyl-2-oxa-8-thiabicyclo[3.2.1]octane-7,7-dicarboxylate (12):**

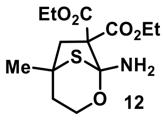

12

**Yield:** 66% (20 mg).

**Nature:** Colourless oil.

**$R_f$ value** = 0.31 [EtOAc:Petroleum ether = 1:4 (v/v)].

**$^1$H NMR (400 MHz, $CDCl_3$) $\delta$ (ppm):** 5.97 (brs, 2H), 4.33 – 4.23 (m, 2H), 4.19 (q, $J$ = 7.1 Hz, 2H), 4.12 (q, $J$ = 7.1 Hz, 2H), 2.91 (d, $J$ = 14.1 Hz, 1H), 2.81 (d, $J$ = 14.1 Hz, 1H), 2.25 – 2.17 (m, 1H), 2.09 – 2.02 (m, 1H), 1.53 (s, 3H), 1.30 (t, $J$ = 7.1 Hz, 3H), 1.26 (t, $J$ = 7.1 Hz, 3H).

**$^{13}$C{$^1$H} NMR (101 MHz, $CDCl_3$) $\delta$ (ppm):** 166.7, 162.0, 155.2, 90.9, 65.2, 64.2, 59.2, 56.5, 46.9, 40.8, 28.5, 14.8, 14.4.

**HRMS** (ESI) *m/z* calcd for $C_{13}H_{22}NO_5S$ $[M+H]^+$: 304.1219; found: 304.1211.

**Thioxo-pyrrolidine to Oxo-pyrrolidine 13:**

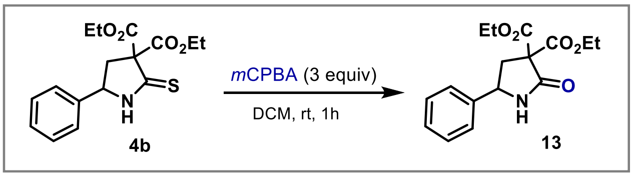


70% 3-Chloroperbenzoic acid (148 mg, 0.6 mmol) was added (slow addition over 15-20 min) to ice-cold solution of thioxopyrrolidine **4b** (64 mg, 0.2 mmol) in DCM (2 mL). Then, the reaction was allowed to warm up to rt and stirred for 1 h. The progress of the reaction was monitored by TLC. After the completion of the reaction, saturated NaHCO3 solution was added until the pH was neutral. The organic layer was separated, and the aqueous phase was extracted with DCM (2×3 mL). The combined organic layers were washed with brine (3 mL), dried with anhydrous $Na_2SO_4$, and concentrated under vacuo. Then, the residue was purified by silica gel column chromatography (230−400 mesh) using EtOAc/petroleum ether as eluent to afford the corresponding product **13**.

**Diethyl 2-oxo-5-phenylpyrrolidine-3,3-dicarboxylate (13):**

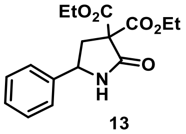

13

**Yield:** 85% (52 mg).

**Nature:** Colourless oil.

**$R_f$ value** = 0.30 [EtOAc:Petroleum ether = 1:4 (v/v)].

**$^1$H NMR (400 MHz, $CDCl_3$) $\delta$ (ppm):** 7.35 – 7.27 (m, 5H), 7.16 (brs, 1H), 4.74 (t, $J$ = 7.3 Hz, 1H), 4.34 – 4.24 (m, 2H), 4.19 (q, $J$ = 7.0 Hz, 2H), 3.15 (dd, $J$ = 13.5, 7.1 Hz, 1H), 2.59 (dd, $J$ = 13.5, 7.6 Hz, 1H), 1.28 (t, $J$ = 7.1 Hz, 3H), 1.21 (t, $J$ = 7.2 Hz, 3H).

**$^{13}C\{^1H\}$ NMR (101 MHz, CDCl$_3$) $\delta$ (ppm):** 169.9, 167.1, 167.0, 140.7, 129.0, 128.3, 126.0, 63.4, 62.7, 62.5, 55.4, 40.3, 14.0, 13.9.

**HRMS** (ESI) *m/z* calcd for $C_{16}H_{19}NO_5Na$ $[M+Na]^+$: 328.1161; found: 328.1146.

**Thioxopyrrolidine to Thioimidate 14:**

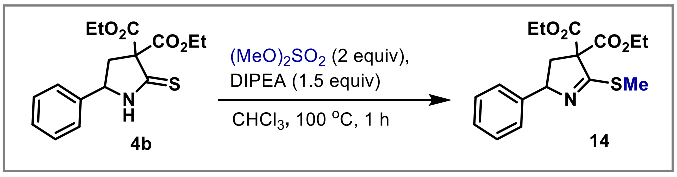


DIPEA (52 µL, 0.3 mmol) and dimethyl sulfate (38 µL, 0.4 mmol) was added to the solution of thioxopyrrolidine **4b** (64 mg, 0.2 mmol) in $CHCl_3$ (1 mL) in a sealed tube. Then, the reaction mixture was heated at 100 °C for 1 h. The progress of the reaction was monitored by TLC. After the completion of the reaction, DCM (5 mL) was added and washed with water (3 mL). The organic layer was dried with anhydrous $Na_2SO_4$ and concentrated under vacuo. Then, the residue was purified by silica gel column chromatography (230−400 mesh) using EtOAc/petroleum ether as eluent to afford the corresponding product **14**.

**Diethyl 2-(methylthio)-5-phenyl-4,5-dihydro-3*H*-pyrrole-3,3-dicarboxylate (14):**

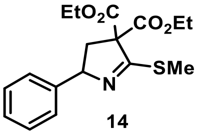


**Yield:** 73% (49 mg).

**Nature:** Colourless oil.

**$R_f$ value** = 0.34 [EtOAc:Petroleum ether = 1:4 (v/v)].

**$^1H$ NMR (400 MHz, CDCl$_3$) $\delta$ (ppm):** 7.35 – 7.23 (m, 5H), 5.15 (t, *J* = 7.7 Hz, 1H), 4.29 (q, *J* = 7.0 Hz, 2H), 4.27 – 4.20 (m, 2H), 3.16 (dd, *J* = 13.5, 7.2 Hz, 1H), 2.54 (dd, *J* = 13.5, 7.2 Hz, 1H) 2.53 (s, 3H), 1.31 (t, *J* = 7.1 Hz, 3H), 1.27 (t, *J* = 7.1 Hz, 3H).

**$^{13}C\{^1H\}$ NMR (101 MHz, CDCl$_3$) $\delta$ (ppm):** 168.9, 167.7, 142.7, 128.6, 127.4, 126.6, 73.5, 72.8, 62.5, 62.4, 43.6, 15.0, 14.0, 13.9.

**HRMS** (ESI) *m/z* calcd for $C_{17}H_{22}NO_4S$ $[M+H]^+$: 336.1270; found: 336.1275.

**Thiocyanate to Phosphonothioate 15:**

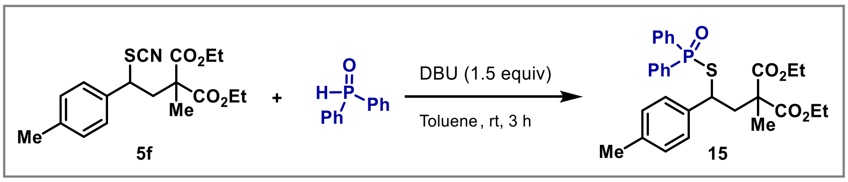


To a culture tube with a magnetic stir bar were added **5f** (70 mg, 0.2 mmol), diphenylphosphine oxide (61 mg, 0.3 mmol), 1,8-diazabicyclo[5.4.0]undec-7-ene (DBU, 45 mg, 0.3 mmol) and toluene (3 mL). The resulting mixture was stirred for 3 h at rt. After completion of the reaction (monitored by TLC), the solvent was removed under vacuo. The residue was purified by silica gel column chromatography (230-400 mesh) using EtOAc/petroleum ether as eluent to afford the corresponding **15**.

**Diethyl 2-(2-((diphenylphosphoryl)thio)-2-(*p*-tolyl)ethyl)-2-methylmalonate (15):**

**Yield:** 71% (75 mg).

**Nature:** Low melting solid.

**R$_f$ value**= 0.33 [EtOAc/petroleum ether=1:1(v/v)]

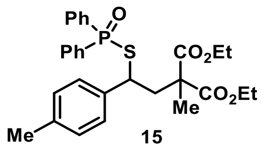

15

**$^1$H NMR (400 MHz, CDCl$_3$) *δ* (ppm):** 7.78 – 7.72 (m, 2H), 7.70 – 7.64 (m, 2H), 7.50 – 7.45 (m, 1H), 7.44 – 7.38 (m, 3H), 7.32 – 7.27 (m, 2H), 6.96 (d, *J* = 8.1 Hz, 2H), 6.86 (d, *J* = 8.0 Hz, 2H), 4.46 – 4.40 (m, 1H), 4.06 (q, *J* = 7.0 Hz, 2H), 3.74 – 3.66 (m, 1H), 3.50 – 3.42 (m, 1H), 2.84 (dd, *J* = 14.5, 10.4 Hz, 1H), 2.74 (dd, *J* = 14.5, 4.7 Hz, 1H), 2.20 (s, 3H), 1.30 (s, 3H), 1.17 (t, *J* = 7.1 Hz, 3H), 1.01 (t, *J* = 7.2 Hz, 3H).

**$^{13}$C{$^1$H} NMR (101 MHz, CDCl$_3$) *δ* (ppm):** 171.8, 171.0, 137.7 (d, *J* = 2.9 Hz), 137.2, 134.6, 133.6, 133.2, 132.3 (d, *J* = 2.7 Hz), 132.0 (d, *J* = 10.5 Hz), 131.8 (d, *J* = 2.5 Hz), 131.2 (d, *J* = 10.8 Hz), 128.9, 128.6 (d, *J* = 13.1 Hz), 128.4 (d, *J* = 13.4 Hz), 128.1, 61.5, 61.1, 53.2, 45.6 (d, *J* = 1.6 Hz), 42.8 (d, *J* = 4.2 Hz), 21.1, 19.5, 14.0, 13.7.

**$^{31}$P NMR (162 MHz, CDCl$_3$) *δ* (ppm):** 41.1.

**HRMS** (ESI) *m/z* calcd for $C_{29}H_{33}O_5PSNa$ [M+Na]$^+$: 547.1684; found: 547.1690.

## Thiocyanate to Trifluoromethyl Thioethers 16:

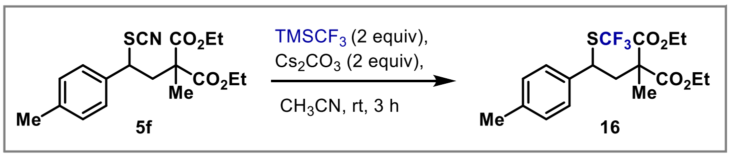


In a culture tube with a magnetic stir bar were charged **5f** (70 mg, 0.2 mmol), $CH_3CN$ (2 mL), and $Cs_2CO_3$ (130 mg, 0.4 mmol). Then $TMSCF_3$ (59 µL, 0.4 mmol) was added to the ice-cold reaction mixture, and the resulting mixture was stirred at ambient temperature for 3 h. After completion of the reaction (monitored by TLC), water (2 mL) was added to quench the reaction, and the mixture was extracted with DCM (5 mL). The resulting organic phase was washed with brine (3 mL). The organic layer was dried over anhydrous $Na_2SO_4$ and concentrated in vacuo. The residue was purified by silica gel column chromatography (230-400 mesh) using EtOAc/petroleum ether as eluent to afford the corresponding **16**.

### Diethyl 2-methyl-2-(2-(*p*-tolyl)-2-((trifluoromethyl)thio)ethyl)malonate (16):

**Yield:** 61% (48 mg).

**Nature:** Transparent gum.

**R$_f$ value**= 0.30 [EtOAc/petroleum ether=1:19(v/v)]

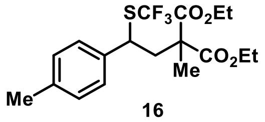

16

**$^1$H NMR (400 MHz, CDCl$_3$) *δ* (ppm):** 7.18 (d, *J* = 8.2 Hz, 2H), 7.11 (d, *J* = 8.0 Hz, 2H), 4.43 (dd, *J* = 9.0, 5.1 Hz, 1H), 4.10 (q, *J* = 7.1 Hz, 2H), 3.96 – 3.88 (m, 1H), 3.79 – 3.71 (m, 1H), 2.69 (dd, *J* = 14.7, 9.0 Hz, 1H), 2.62 (dd, *J* = 14.7, 5.1 Hz, 1H), 2.31 (s, 3H), 1.37 (s, 3H), 1.22 (t, *J* = 7.1 Hz, 3H), 1.11 (t, *J* = 7.1 Hz, 3H).

**$^{13}$C{$^1$H} NMR (101 MHz, CDCl$_3$) *δ* (ppm):** 171.5, 171.2, 138.1, 137.0, 130.3 (q, *J* = 308.8 Hz), 129.5, 127.8, 61.7, 61.5, 53.1, 45.8, 41.6, 21.2, 20.1, 14.0, 13.8.

**$^{19}$F NMR (377 MHz, CDCl$_3$) δ (ppm):** - 40.3.

**HRMS** (ESI) *m/z* calcd for $C_{18}H_{23}F_3O_4SNa$ [M+Na]$^+$: 415.1167; found: 415.1169.

### Isothiocyanate to 1,2,4-thiadiazole 17:[23]

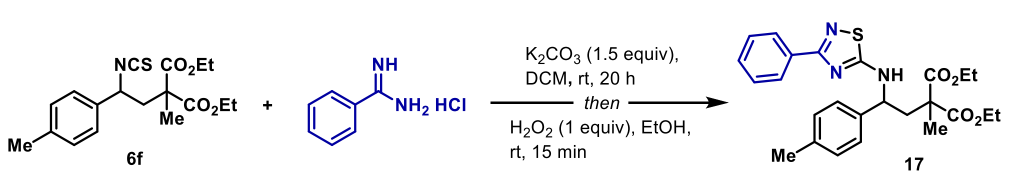


To a round bottom flask with a magnetic stir bar were charged with **6f** (70 mg, 0.2 mmol), benzamidine hydrochloride (31 mg, 0.2 mmol), potassium carbonate (41 mg, 0.3 mmol), and DCM (2 mL). The reaction mixture was stirred for 20 h at rt. Upon completion of the reaction, the mixture was quenched with water (2 mL) and extracted with ethyl acetate (3×2 mL). The combined organic layers were dried over anhydrous $Na_2SO_4$, and concentrated under vacuo. Then, the ethanol (2 mL) and hydrogen peroxide (23 µL, 0.2 mmol, 30% w/v in water) were added to the crude product. The mixture was then stirred for 15 min at rt. After completion of the reaction (monitored by TLC), the mixture was evaporated under vacuo, and the residue was purified by silica gel column chromatography (230-400 mesh) using EtOAc/petroleum ether as eluent to afford the corresponding **17**.

**Diethyl 2-methyl-2-(2-((3-phenyl-1,2,4-thiadiazol-5-yl)amino)-2-(*p*-tolyl)ethyl)malonate (17):**

**Yield:** 67% (63 mg).

**Nature:** Colourless oil.

**$R_f$ value**= 0.32 [EtOAc/petroleum ether=1:4 (v/v)]

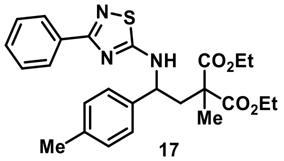

17

**$^1$H NMR (400 MHz, CDCl$_3$) $\delta$ (ppm):** 8.13 – 8.10 (m, 2H), 7.40 – 7.38 (m, 3H), 7.27 (d, *J* = 8.0 Hz, 2H), 7.16 (d, *J* = 8.0 Hz, 2H), 6.81 (d, *J* = 6.7 Hz, 1H), 4.53 (t, *J* = 6.3 Hz, 1H), 4.22 (q, *J* = 7.1 Hz, 2H). 4.17 – 4.04 (m, 2H), 2.66 (dd, *J* = 15.0, 10.6 Hz, 1H), 2.33 (s, 3H), 2.19 (dd, *J* = 15.0, 3.7 Hz, 1H), 1.55 (s, 3H), 1.22 (t, *J* = 7.2 Hz, 3H), 1.20 (t, *J* = 7.2 Hz, 3H).

**$^{13}$C{$^1$H} NMR (101 MHz, CDCl$_3$) $\delta$ (ppm):** 183.3, 172.6, 172.5, 169.8, 138.1, 137.8, 133.4, 129.9, 129.7, 128.5, 128.1, 126.6, 62.2, 62.1, 57.7, 52.9, 43.2, 21.2, 20.6, 14.1, 14.0.

**HRMS** (ESI) *m/z* calcd for $C_{25}H_{30}N_3O_4S$ $[M+H]^+$: 468.1957; found: 468.1958.

### Isothiocyanate to Thiourea Derivative 18:

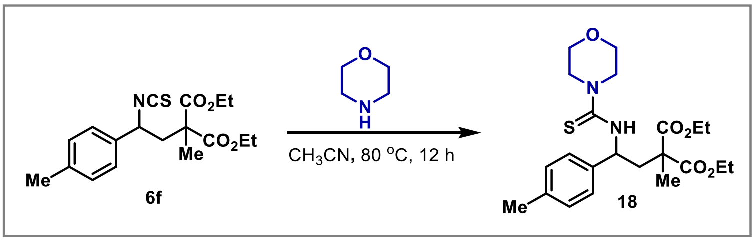


To a round bottom flask with a magnetic stir bar were charged with **6f** (70 mg, 0.2 mmol), morpholine (21 µL, 0.24 mmol), and $CH_3CN$ (2 mL). The reaction mixture was heated at 80 ºC for 12 h. After completion of the reaction (monitored by TLC), saturated $NH_4Cl$ was added, and the mixture was extracted with ethyl acetate (2×5 mL). The organic layer was washed with brine (3 mL), dried over

anhydrous $Na_2SO_4$, and evaporated under vacuo. The residue was purified by silica gel column chromatography (230-400 mesh) using EtOAc/petroleum ether as eluent to afford the corresponding **18**.

**Diethyl 2-methyl-2-(2-(morpholine-4-carbothioamido)-2-(*p*-tolyl)ethyl)malonate (18):**

**Yield:** 68% (59 mg).

**Nature:** Colourless oil.

**$R_f$ value**= 0.34 [EtOAc/petroleum ether=3:7(v/v)]

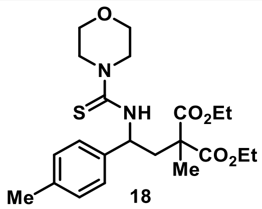

18

**$^1$H NMR (400 MHz, $CDCl_3$) $\delta$ (ppm):** 7.20 (d, $J$ = 8.1 Hz, 2H), 7.13 (d, $J$ = 8.0 Hz, 2H), 7.08 (d, $J$ = 6.5 Hz, 1H), 5.58 – 5.52 (m, 1H), 4.30 – 4.18 (m, 2H), 4.17 – 4.06 (m, 2H), 3.781 – 3.75 (m, 4H), 3.74 – 3.69 (m, 4H), 2.70 (dd, $J$ = 15.0, 11.9 Hz, 1H), 2.30 (s, 3H), 1.97 (dd, $J$ = 15.0, 3.9 Hz, 1H), 1.56 (s, 3H), 1.27 (t, $J$ = 7.1 Hz, 3H), 1.21 (t, $J$ = 7.1 Hz, 3H).

**$^{13}$C{$^1$H} NMR (101 MHz, $CDCl_3$) $\delta$ (ppm):** 181.7, 173.7, 173.0, 140.1, 136.9, 129.4, 126.5, 66.4, 62.9, 62.0, 56.2, 53.3, 47.5, 42.0, 21.2, 20.0, 14.1, 14.0.

**HRMS** (ESI) *m/z* calcd for $C_{22}H_{32}N_2O_5SNa$ $[M+Na]^+$: 459.1930; found: 459.1914.

## 8. Mechanistic Studies:

### 8.1. Radical Inhibition Experiments:

#### 8.1.1. Trapping with TEMPO:

To explore the reaction mechanism, a radical trapping experiment was performed under the standard reaction condition. In the presence of TEMPO (3 equiv.) free radical, the reaction of 4-methylstyrene **1a** (2 equiv.) and diethyl 2-methyl-2-thiocyanatomalonate **2k** (1 equiv.) were fully suppressed, and no desired product **5f** was formed, but 33% of TEMPO adduct **19** was isolated. Very importantly, a trace amount of TEMPO adducts **20** and **21** were detected in HRMS analysis from the crude reaction mixture, implying that the mechanism of this photo-reaction involves the generation of malonyl and thiocyanate radical from **2k**. This newly generated malonyl and thiocyanate radical coupled with TEMPO to provide **19** and **21**, respectively. Additionally, the existence of **20** suggests that the benzyl radical species is the intermediate of this reaction.

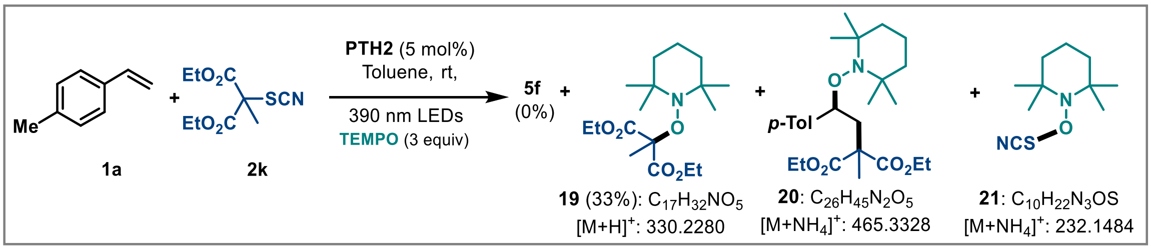


A culture tube equipped with a magnetic stir bar was charged with **PTH2** (3 mg, 0.01 mmol, 5 mol%), diethyl 2-methyl-2-thiocyanatomalonate **2k** (46 mg, 0.2 mmol) and dry toluene (2 mL). The tube was sealed with a Teflon screw cap, evacuated, and backfilled with argon, before 4-methylstyrene **1a** (53 µL, 0.4 mmol) and TEMPO free radical (94 mg, 0.6 mmol) were added to it. Then, the reaction mixture was degassed by Freeze-Pump-Thaw cycles with argon and irradiated at rt with 390 nm LEDs at a distance of approximately 5 cm. A high-speed fan was used to maintain the temperature. After 30 min no desired carbo-thiocyanation product **5f** was formed. A trace amount of the TEMPO adducts **20** and **21** were detected in HRMS analysis from the crude reaction mixture, and 33% of **19** was isolated using silica gel column chromatography. These results suggested that the reaction passes through the radical pathway.

**Diethyl 2-methyl-2-((2,2,6,6-tetramethylpiperidin-1-yl)oxy)malonate (19):**[24]

**Yield:** 33% (22 mg).

**Nature:** White solid.

**$R_f$ value** = 0.33 [EtOAc:Petroleum ether = 1:19 (v/v)].

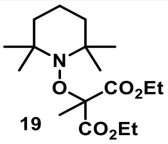


19

**$^1$H NMR (400 MHz, $CDCl_3$) $\delta$ (ppm):** 4.28 – 4.16 (m, 4H), 1.73 (s, 3H), 1.56 – 1.39 (m, 6H), 1.27 (t, $J$ = 7.1 Hz, 6H), 1.22 (s, 6H), 1.02 (s, 6H).

**$^{13}$C{$^1$H} NMR (101 MHz, $CDCl_3$) $\delta$ (ppm):** 170.6, 85.7, 61.7, 60.5, 40.9, 33.2, 20.8, 18.1, 17.1, 14.2.

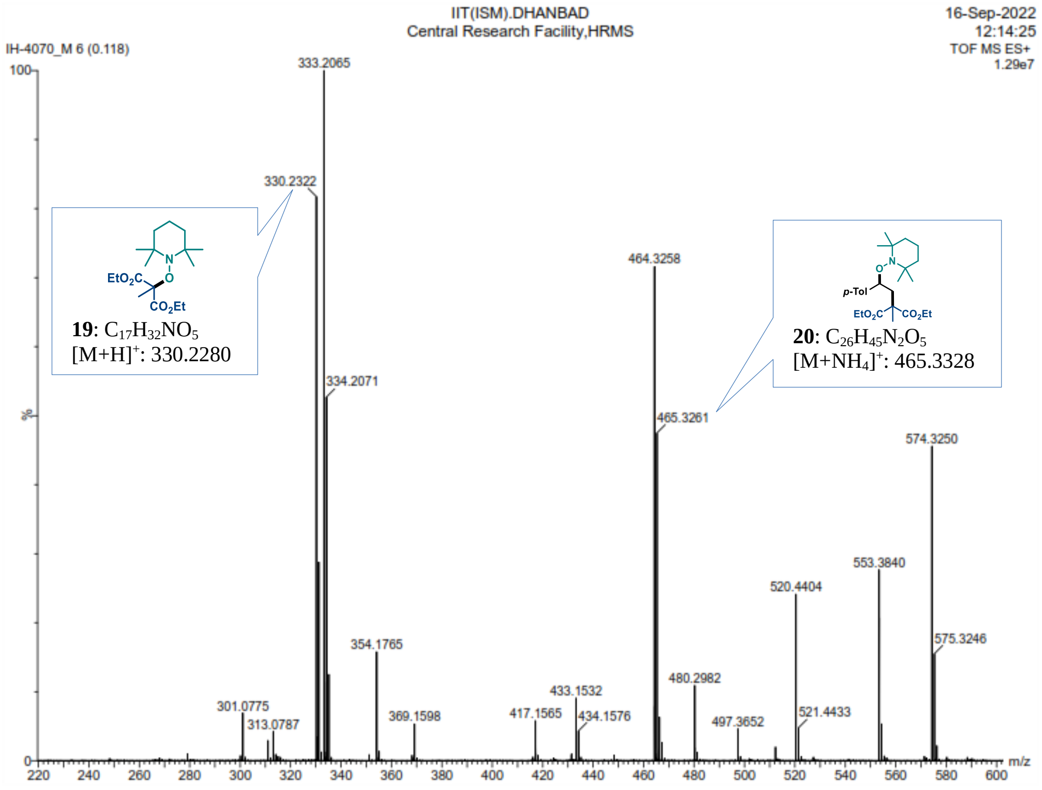


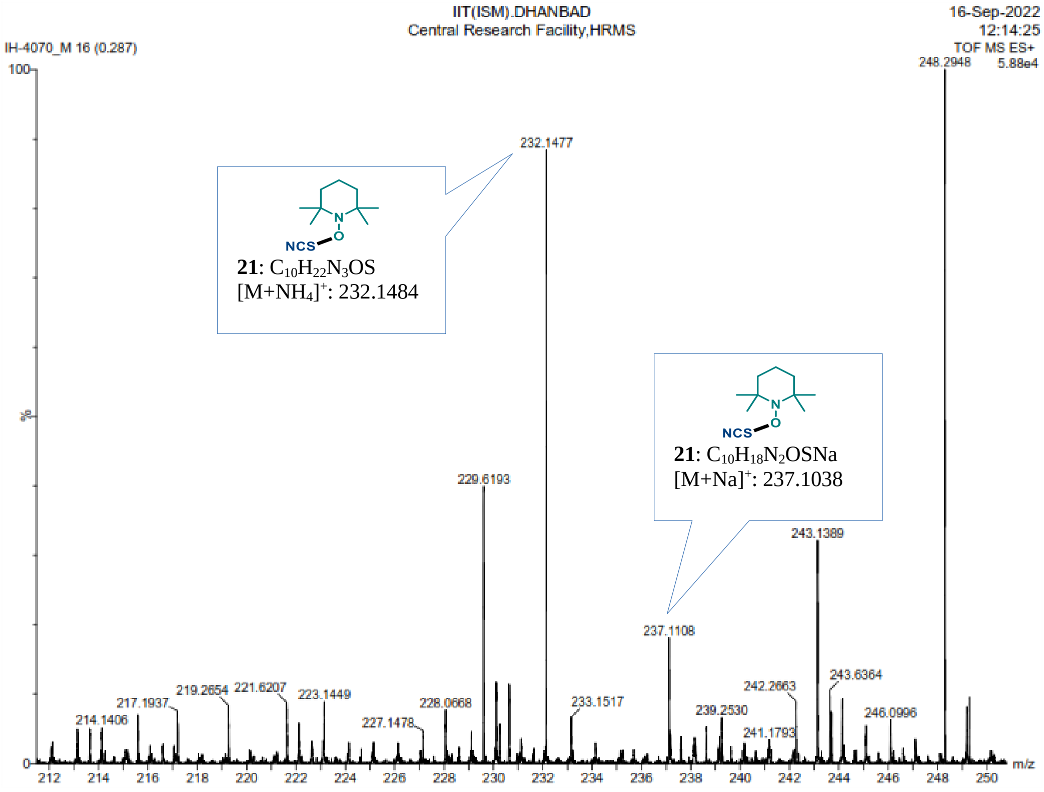


**Supplementary Fig. 3:** HRMS of the crude reaction mixture (compounds **19**, **20**, and **21**)

## 8.1.2. Trapping with 2-phenylimidazo[1,2-*a*]pyridine:

To explore the reaction mechanism, another radical trapping experiment was performed under the standard reaction condition. In the presence of 2-phenyl imidazo[1,2-*a*]pyridine (3 equiv.), the reaction of 4-methylstyrene **1a** (2 equiv.) and diethyl 2-methyl-2-thiocyanatomalonate **2k** (1 equiv.) were fully suppressed and no desired product **5f** was formed but 26% and 18% of adduct **19'** and **21'** ware isolated. Very importantly, a trace amount of imidazopyridine adducts **20'** was detected in HRMS analysis from the crude reaction mixture, and this also implies that the mechanism of this photo-reaction involves the generation of malonyl and thiocyanate radical from **2k**.[25] This newly generated malonyl and thiocyanate radical coupled with imidazopyridine to provide **19'** and **21'**, respectively. Additionally, the existence of **20'** suggests that the benzyl radical species is the intermediate of this reaction.

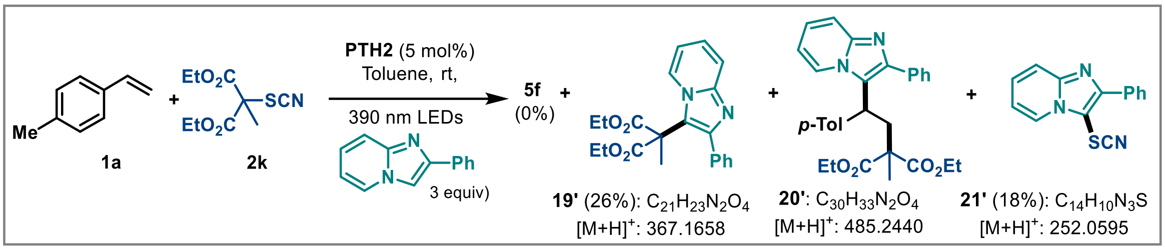


A culture tube equipped with a magnetic stir bar was charged with **PTH2** (3 mg, 0.01 mmol, 5 mol%), diethyl 2-methyl-2-thiocyanatomalonate **2k** (46 mg, 0.2 mmol) and dry toluene (2 mL). The tube was sealed with a Teflon screw cap, evacuated, and backfilled with argon, before 4-methylstyrene **1a** (53 µL, 0.4 mmol) and 2-phenylimidazo[1,2-*a*]pyridine (116 mg, 0.6 mmol) were added to it. Then, the reaction mixture was degassed by Freeze-Pump-Thaw cycles with argon and irradiated at rt with 390 nm LEDs at a distance of approximately 5 cm. A high-speed fan was used to maintain the temperature. After 30 min no desired carbo-thiocyanation product **5f** was formed. A trace amount of the imidazopyridine adduct **20'** was detected in HRMS analysis from the crude reaction mixture, and 26% of **19'** and 18% of **21'** were isolated using silica gel column chromatography. These results also suggested that the reaction passes through the radical pathway.

**Diethyl 2-methyl-2-(2-phenylimidazo[1,2-*a*]pyridin-3-yl)malonate (19'):**

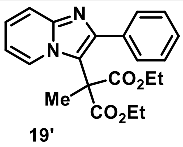


**Yield:** 26% (19 mg).

**Nature:** Colourless oil

**$R_f$ value** = 0.30 [EtOAc:Petroleum ether = 3:7 (v/v)].

**$^1$H NMR (400 MHz, $CDCl_3$) $\delta$ (ppm):** 7.97 (d, $J$ = 7.0 Hz, 1H), 7.76 (d, $J$ = 9.0 Hz, 1H), 7.48 – 7.45 (m, 2H), 7.43 – 7.38 (m, 3H), 7.32 – 7.28 (m, 1H), 6.89 – 6.85 (m, 1H), 4.13 – 3.95 (m, 4H), 1.78 (s, 3H), 1.14 (t, $J$ = 7.1 Hz, 6H).

**$^{13}$C{$^1$H} NMR (101 MHz, $CDCl_3$) $\delta$ (ppm):** 169.6, 144.4, 144.3, 135.0, 130.2, 128.6, 128.1, 125.8, 125.7, 117.7, 117.5, 112.5, 62.6, 54.4, 21.8, 13.9.

**HRMS** (ESI) *m/z* calcd for $C_{21}H_{23}N_2O_4$ [M+H]$^+$: 367.1658; found: 367.1667.

**2-Phenyl-3-thiocyanatoimidazo[1,2-*a*]pyridine (21'):**[25]

**Yield:** 18% (9 mg).

**Nature:** White solid

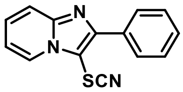


21'

$\mathbf{R_f}$ **value** = 0.42 [EtOAc:Petroleum ether = 3:7 (v/v)].

**$^1$H NMR (400 MHz, $CDCl_3$) $\delta$ (ppm):** 8.48 (d, $J$ = 6.8 Hz, 1H), 8.08 – 8.05 (m, 2H), 7.81 (d, $J$ = 8.9 Hz, 1H), 7.57 – 7.47 (m, 4H), 7.18 – 7.15 (m, 1H).

**$^{13}C\{^1H\}$ NMR (101 MHz, $CDCl_3$) $\delta$ (ppm):** 153.0, 147.9, 131.8, 129.7, 129.0, 128.9, 128.4, 124.6, 118.3, 114.8, 108.1, 95.1.

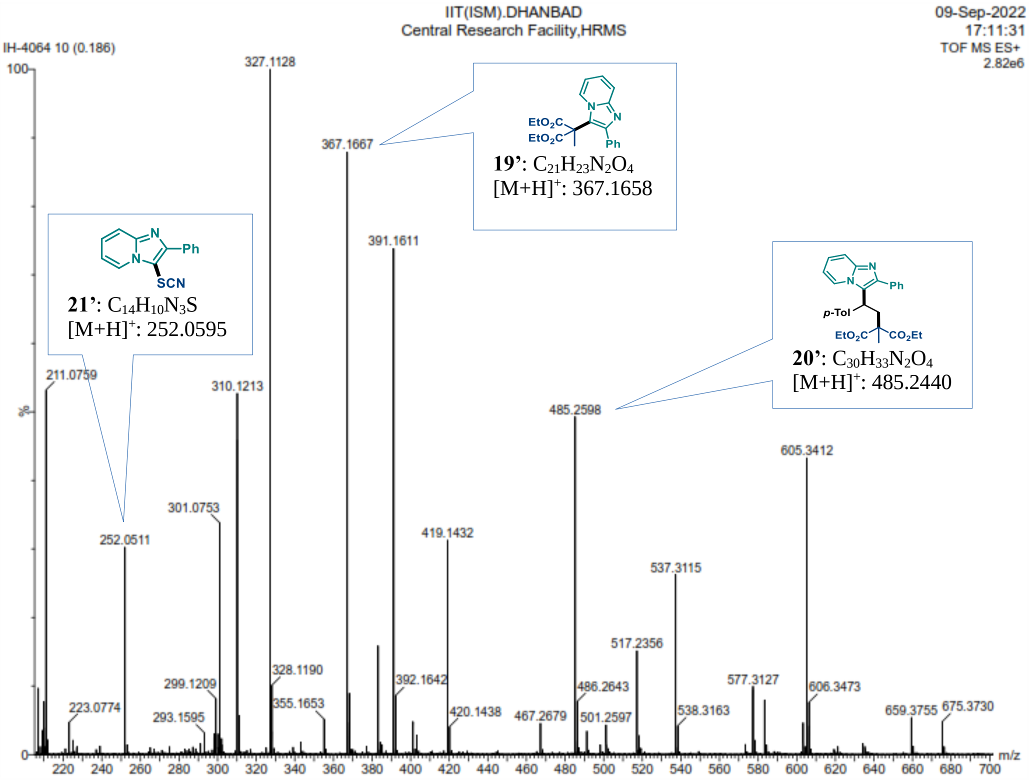


**Supplementary Fig. 4:** HRMS of the crude reaction mixture (compounds **19'**, **20'**, and **21'**)

## 8.2. Radical Probe Experiments:

### 8.2.1. Radical Clock Experiment:

Next, a radical clock experiment was conducted to gain a better insight into the reaction mechanism. Here *α*-cyclopropyl-4-chlorostyrene **22** reacts with diethyl 2-thiocyanatomalonate **2a** under standard reaction conditions, producing ring-opening product **23** in good yield. The rearrangement product **23** has resulted from oxidative addition with thiocyanate, which is generated from the rapid ring opening process of cyclopropyl methyl radical **I**, itself generated upon the addition of the malonyl radical to the alkene. This experiment discloses strong support for the participation of malonyl radicals in this protocol via SET-type mechanism.

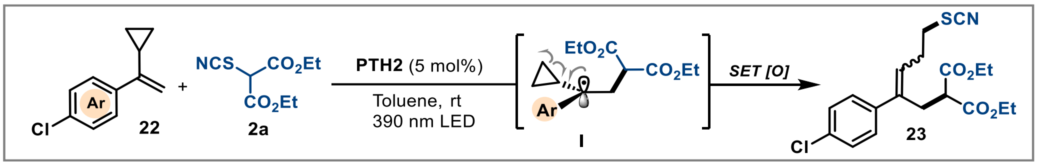


A culture tube equipped with a magnetic stir bar was charged with **PTH2** (3 mg, 0.01 mmol, 5 mol%), diethyl 2-thiocyanatomalonate **2a** (44 mg, 0.2 mmol), and dry toluene (2 mL). The tube was sealed with a Teflon screw cap, evacuated, and backfilled with argon, before *α*-cyclopropyl-4-chlorostyrene **22** (62 μL, 0.4 mmol) was added to it. Then, the yellow reaction mixture was degassed by Freeze-Pump-Thaw cycles with argon and irradiated at rt with 390 nm LEDs at a distance of approximately 5 cm for 30 min. After completion of the reaction (confirmed by TLC), the crude was concentrated under vacuo, and purified by silica gel column chromatography (230−400 mesh) using EtOAc/petroleum ether as eluent to afford the corresponding product **23**.

**Diethyl 2-(2-(4-chlorophenyl)-5-thiocyanatopent-2-en-1-yl)malonate (23):**

**Yield:** 58% (46 mg, combined yield as a 1:1 mixture of *E*/*Z* isomers)

**Nature:** Colourless oil.

**$R_f$ value** = 0.39 [EtOAc:Petroleum ether = 1:4 (v/v)].

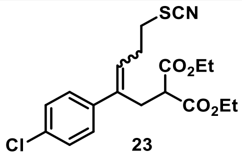


**$^1$H NMR (400 MHz, $CDCl_3$) *δ* (ppm):** (for the mixture) 7.35 (d, *J* = 8.4 Hz, 2H), 7.30 (d, *J* = 8.5 Hz, 2H), 7.22 (d, *J* = 8.5 Hz, 2H), 7.07 (d, *J* = 8.4 Hz, 2H), 5.66 (t, *J* = 7.3 Hz, 1H), 5.54 (t, *J* = 7.2 Hz, 1H), 4.18 – 4.12 (m, 4H), 4.12 (q, *J* = 7.1 Hz, 4H), 3.29 (t, *J* = 7.8 Hz, 1H), 3.28 (t, *J* = 7.8 Hz, 1H), 3.12 (d, *J* = 7.7 Hz, 2H), 3.07 (t, *J* = 7.1 Hz, 2H), 2.93 (d, *J* = 7.7 Hz, 2H), 2.88 (t, *J* = 7.1 Hz, 2H), 2.78 (q, *J* = 7.2 Hz, 2H), 2.40 (q, *J* = 7.1 Hz, 2H), 1.24 (t, *J* = 7.1 Hz, 6H), 1.21 (t, *J* = 7.1 Hz, 6H).

**$^{13}C\{^1H\}$ NMR (101 MHz, $CDCl_3$) *δ* (ppm):** (for the mixture) 168.9, 168.8, 140.5, 139.4, 139.2, 136.9, 133.8, 133.7, 129.9, 129.0, 128.9, 128.1, 127.4, 125.8, 112.2, 112.0, 61.8, 61.7, 50.6, 50.4, 38.4, 33.8, 29.2, 29.1, 29.0, 14.2, 14.1.

**HRMS** (ESI) *m/z* calcd for $C_{19}H_{23}ClNO_4S$ $[M+H]^+$: 396.1036; found: 396.1007.

## 8.2.2. Radical 5-Exo-trig Cyclization:

Next, a radical 5-exo-trig cyclization experiment was conducted to gain a better insight into the reaction mechanism. Here dimethyl 2,2-diallylmalonate **24** reacts with 2-methyl-2-thiocyanatomalonate **2k** under standard reaction conditions, producing cyclized product **25** in good yield. The rearrangement product **25** has resulted from oxidative addition with thiocyanate, which is generated from the rapid rearrangement process of radical **II**, itself generated upon the addition of the malonyl radical to the alkene. This experiment also discloses strong support for the participation of malonyl radical in this protocol via SET-type mechanism.

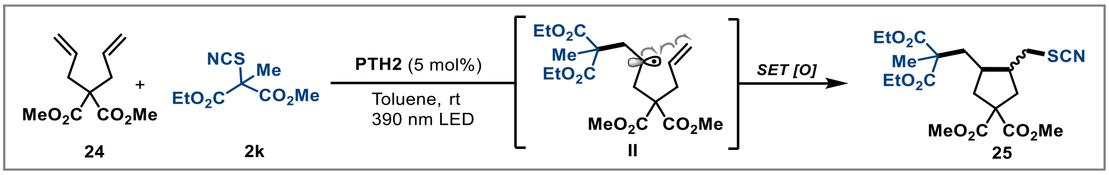


A culture tube equipped with a magnetic stir bar was charged with **PTH2** (3 mg, 0.01 mmol, 5 mol%), 2-methyl-2-thiocyanatomalonate **2k** (46 mg, 0.2 mmol), and dry toluene (2 mL). The tube was sealed with a Teflon screw cap, evacuated, and backfilled with argon,  before dimethyl 2,2-diallylmalonate **24** (85 mg, 0.4 mmol) was added to it. Then, the yellow reaction mixture was degassed by Freeze-Pump-Thaw cycles with argon and irradiated at rt with 390 nm LEDs at a distance of approximately 5 cm for 30 min. After completion of the reaction (confirmed by TLC), the crude was concentrated under vacuo, and purified by silica gel column chromatography (230−400 mesh) using EtOAc/petroleum ether as eluent to afford the corresponding product **25**.

**Dimethyl 3-(3-ethoxy-2-(ethoxycarbonyl)-2-methyl-3-oxopropyl)-4-(thiocyanatomethyl)cyclopentane-1,1-dicarboxylate (25):**

**Yield:** 63% (56 mg, combined yield as a 9:1 mixture of diastereomers).

**Nature:** Colourless oil.

**$R_f$ value** = 0.43 [EtOAc:Petroleum ether = 1:4 (v/v)].

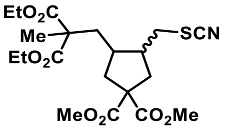


25

**$^1$H NMR (400 MHz, CDCl$_3$) *δ* (ppm):** (for the mixture) 4.22 – 4.10 (m, 4H), 3.70 (s, 3H), 3.69 (s, 3H), 3.38 – 3.21 (m, 1H), 2.79 – 2.56 (m, 1H), 2.50 – 2.27 (m, 4H), 2.21 – 2.12 (m, 1H), 2.07 – 2.01 (m, 1H), 1.86 – 1.76 (m, 2H), 1.41 (s, 3H), 1.29 – 1.16 (m, 6H).

**$^{13}$C{$^1$H} NMR (101 MHz, CDCl$_3$) *δ* (ppm):** (for the mixture) 173.2, 173.1, 172.5, 172.4, 172.3, 172.2, 172.1, 172.0, 112.2, 112.1, 61.8, 61.7, 61.6, 58.7, 58.2, 53.4, 53.3, 53.2, 53.1, 53.0, 46.3, 43.6, 41.4, 40.6, 39.6, 39.1, 39.0, 38.1, 37.5, 37.4, 34.9, 34.4, 20.7, 20.4, 14.1, 14.0.

**HRMS** (ESI) *m/z* calcd for $C_{20}H_{29}NO_8SNa$ [M+Na]$^+$: 466.1512; found: 466.1516.

## 8.3. Deuterium Labeling Experiment:

### Preparation of Diethyl 2-thiocyanatomalonate-*d* (2a-*d*):

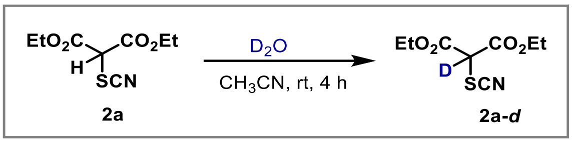


A 50 mL round bottom flask equipped with a magnetic stir bar was charged with diethyl 2-thiocyanatomalonate **2a** (652 mg, 3 mmol) and dry acetonitrile (15 mL). The RB flask was sealed with a septum before $D_2O$ (271 µL, 15 mmol) was added and stirred at rt for 4 h. After 4 h, the crude reaction mixture was concentrated, and thereafter immediately recorded the NMR and HRMS data. The compound was instantly used for photochemical reactions without further purification.

**Diethyl 2-thiocyanatomalonate-*d* (2a-*d*):**

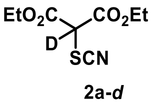


Contains 27% diethyl 2-thiocyanatomalonate (**2a**).

**$^1$H NMR (400 MHz, $CDCl_3$) *δ* (ppm):** 4.30 (q, *J* = 7.1 Hz, 4H), 1.30 (t, *J* = 7.1 Hz, 6H).

**$^{13}$C{$^1$H} NMR (101 MHz, $CDCl_3$) *δ* (ppm):** 163.7, 109.1, 63.9, 51.9 (t, *J* = 22.9 Hz), 13.9.

**HRMS** (ESI) *m/z* calcd for $C_8H_{10}DNO_4SNa$ [M+Na]$^+$: 241.0369; found: 241.0370.

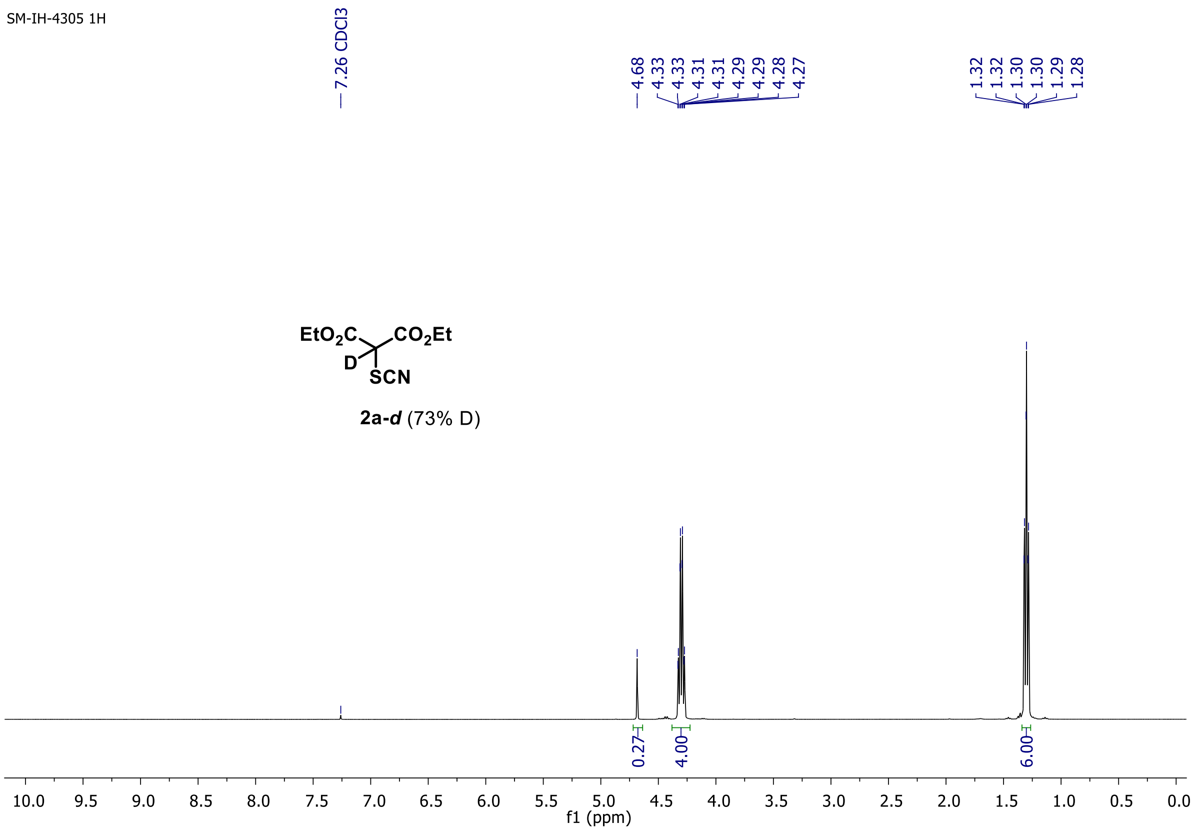


**Supplementary Fig. 5:** Crude $^1$H NMR of the reaction mixture of compound **2a-*d*** (400 MHz, $CDCl_3$)

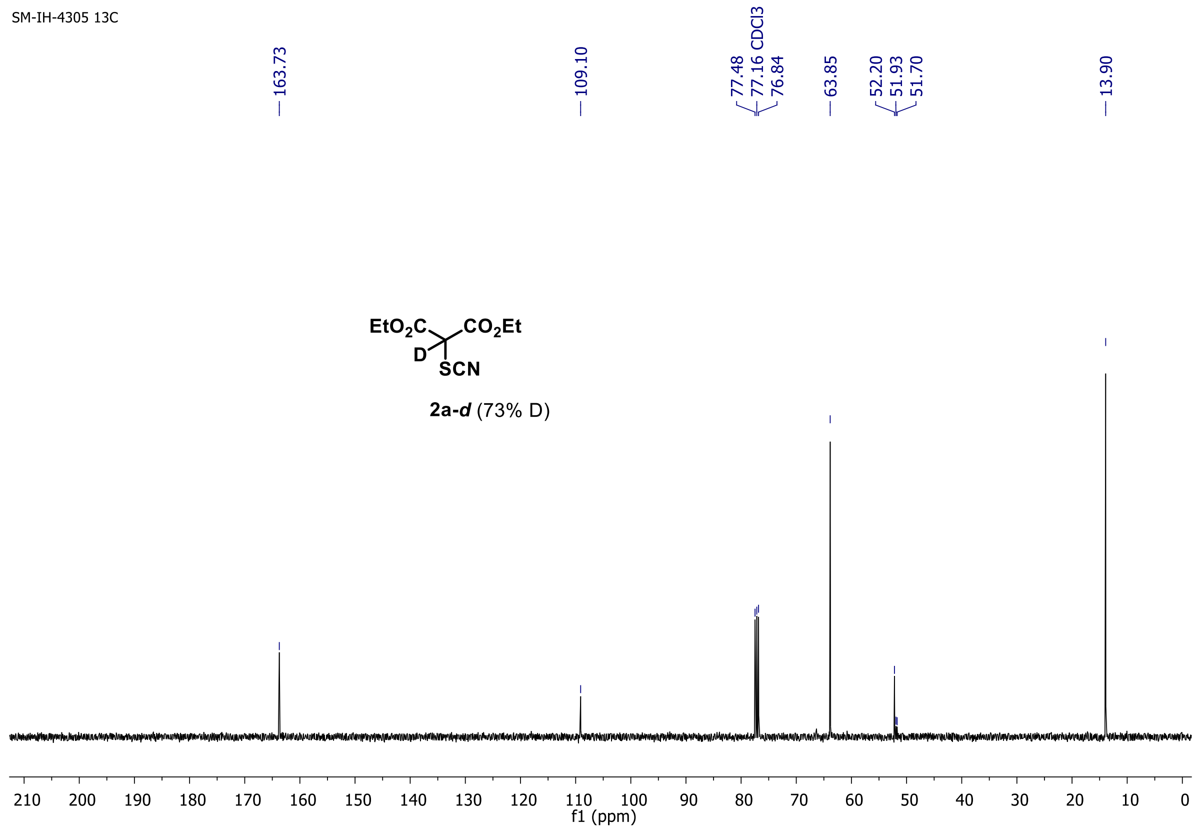


**Supplementary Fig. 6:** Crude $^{13}C\{^{1}H\}$ NMR of reaction mixture of compound ***2a-d*** (101 MHz, $CDCl_3$):

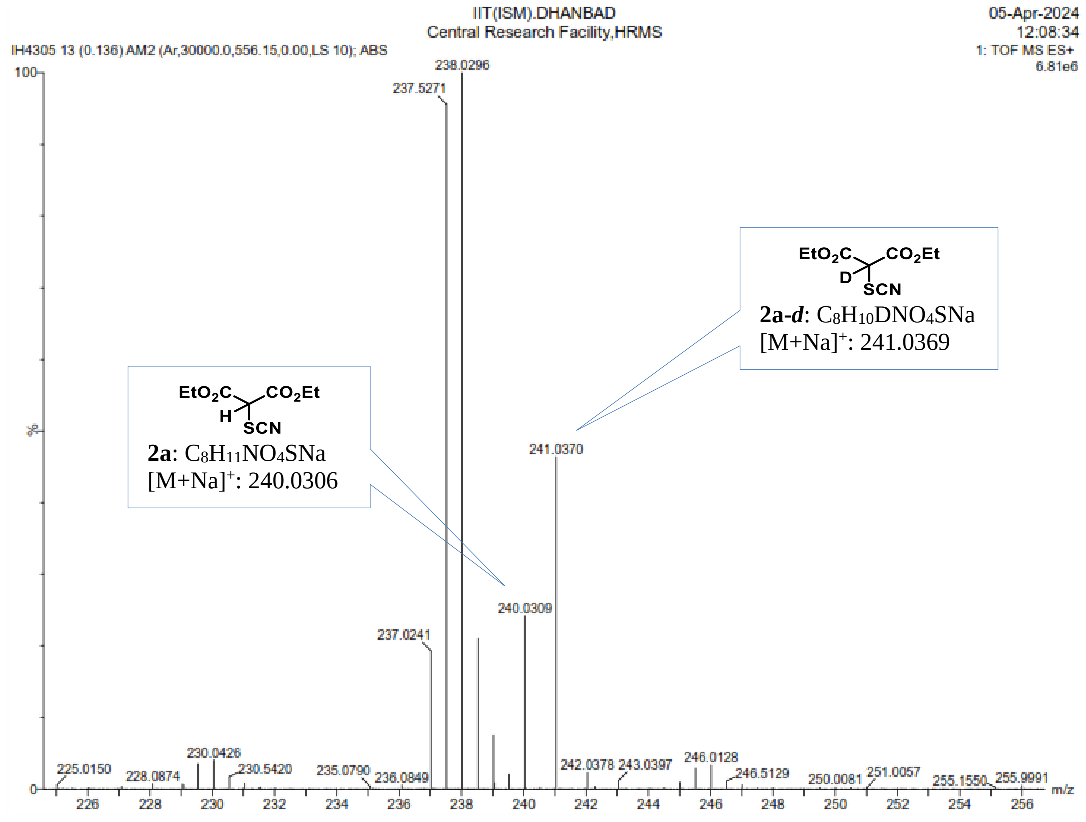


**Supplementary Fig. 7:** HRMS of the crude reaction mixture of compound ***2a-d***

## Photocascade Reaction with Deuterium Labeled Thiocyanatomalonate:

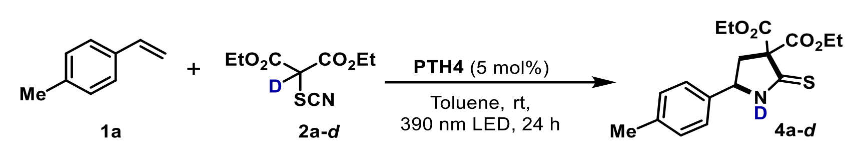


An oven-dried culture tube equipped with a magnetic stir bar was charged with **PTH4** (3.7 mg, 0.01 mmol, 5 mol%), freshly prepared dry diethyl 2-thiocyanatomalonate-*d* (**2a-*d***) (44 mg, 0.2 mmol) and dry toluene (2 mL). The tube was sealed with a Teflon screw cap before 4-methylstyrene **1a** (53 µL, 0.4 mmol) was added to it. Then, the reaction mixture was degassed by Freeze-Pump-Thaw cycles with argon and irradiated at rt with 390 nm LEDs at a distance of approximately 5 cm for 24 h. A high-speed fan was used to maintain the temperature. After the completion of the reaction (confirmed by TLC), the reaction crude was concentrated, and the NMR was immediately recorded with the collection of the HRMS data.

**Supplementary Notes:** *The deuterium-labeled starting material **2a-d** and product **4a-d** are highly susceptible to the exchange of proton with water (even moisture). Attempts to purify these by silica gel column chromatography lead to the corresponding non-deuterated (protonated) products. So, extra care should be taken to ensure the reaction medium and other reagents are extremely anhydrous prior to use.*

### Diethyl 2-thioxo-5-(*p*-tolyl)pyrrolidine-3,3-dicarboxylate-1-*d* (4a-*d*):

Contains 42% diethyl 2-thioxo-5-(*p*-tolyl)pyrrolidine-3,3-dicarboxylate (**4a**).

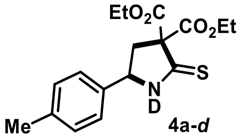


**$^{1}$H NMR (400 MHz, CDCl$_3$) *δ* (ppm):** 7.20 (d, *J* = 8.3 Hz, 2H), 7.17 (d, *J* = 8.4 Hz, 2H), 4.92 (t, *J* = 7.7 Hz, 1H), 4.34 – 4.23 (m, 2H), 4.26 (q, *J* = 7.1 Hz, 2H), 3.18 (dd, *J* = 13.4, 6.8 Hz, 1H), 2.84 (dd, *J* = 13.4, 8.6 Hz, 1H), 2.34 (s, 3H), 1.32 (t, *J* = 7.1 Hz, 3H), 1.28 (t, *J* = 7.1 Hz, 3H).

**$^{13}$C{$^{1}$H} NMR (101 MHz, CDCl$_3$) *δ* (ppm):** 197.6, 167.3, 167.1, 138.8, 135.5, 129.8, 126.5, 71.8, 62.8, 62.6, 62.5, 42.6, 21.2, 14.1, 14.0.

**HRMS** (ESI) *m/z* calcd for $C_{17}H_{21}DNO_4S$ $[M+H]^+$: 337.1332; found: 337.1330.

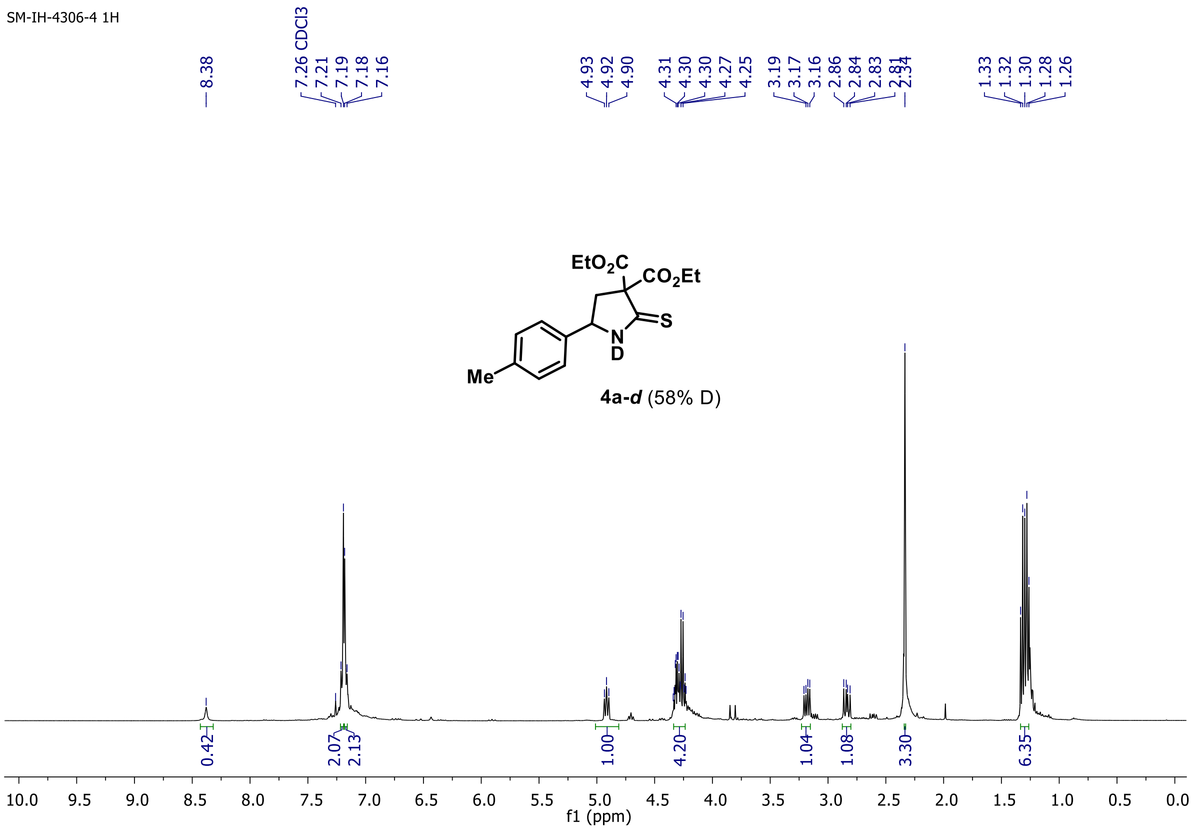


**Supplementary Fig. 8:** Crude $^1$H NMR of the reaction mixture of compound **4a-*d*** (400 MHz, $CDCl_3$)

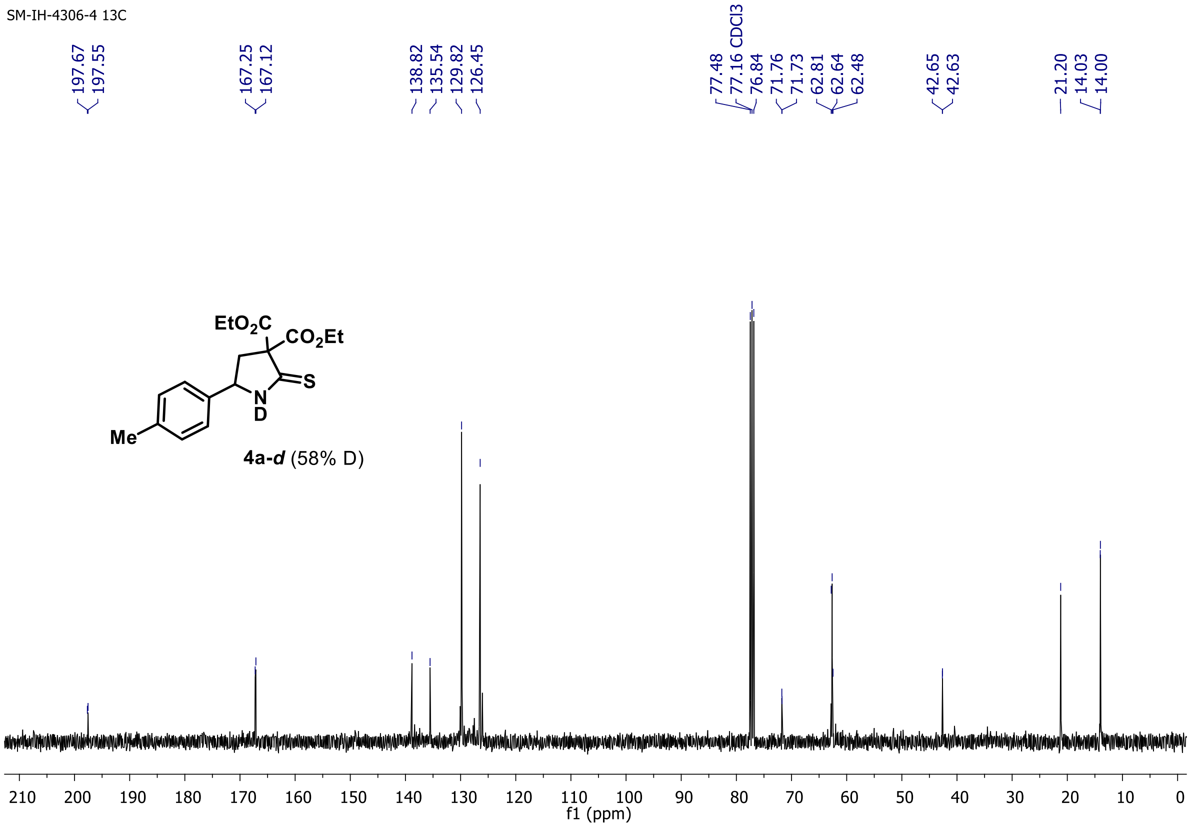


**Supplementary Fig. 9:** Crude $^{13}C\{^1H\}$ NMR of reaction mixture of compound **4a-*d*** (101 MHz, $CDCl_3$):

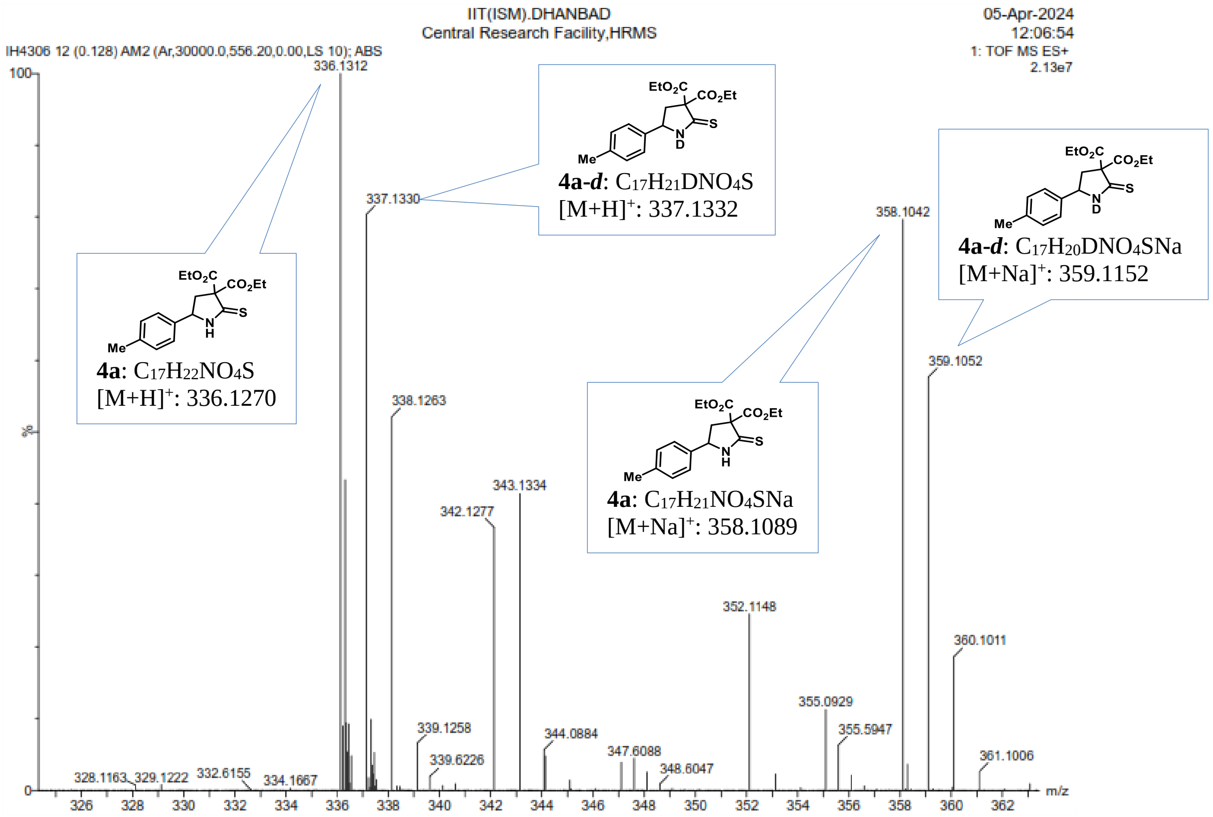


**Supplementary Fig. 10:** HRMS of the crude reaction mixture of compound **4a-*d***

## 8.4. Cation Trapping Experiments:

When we carried out the reaction between ATRA thiocyanation product **5f** and ethanol/1,2,4-trimethoxybenzene under the standard condition, we found to formation of trapping product **26a/26b** in good yield. The trapping product **26a/26b** was resulted from ethoxy/trimethoxybenzene trapping from ethanol/1,2,4-trimethoxybenzene to the cationic intermediate **III**, which is generated from the oxidative transformation of radical to cation, itself generated upon photo-generation of benzylic radical from **5f**. This reveals strong support for the presence of a cationic intermediate in this reaction.

### 8.4.1. Cation Trapping Experiment with Ethanol:

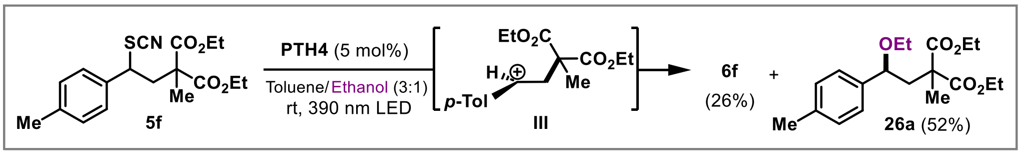


A culture tube equipped with a magnetic stir bar was charged with **PTH4** (3.7 mg, 0.01 mmol, 5 mol%), diethyl 2-methyl-2-(2-thiocyanato-2-(*p*-tolyl)ethyl)malonate **5f** (70 mg, 0.2 mmol) and dry toluene-ethanol (2 mL, 3:1). The tube was sealed with a Teflon screw cap, evacuated and backfilled with argon. Then, the reaction mixture was degassed by Freeze-Pump-Thaw cycles with argon and irradiated at rt with 390 nm LEDs at a distance of approximately 5 cm for 24 h. After completion of the reaction (confirmed by TLC), the crude was concentrated under vacuo, and purified by silica gel column chromatography (230−400 mesh) using EtOAc/petroleum ether as eluent to afford the product **26a** (52%) along with **6f** (26%).

**Diethyl 2-(2-ethoxy-2-(*p*-tolyl)ethyl)-2-methylmalonate (26a):**

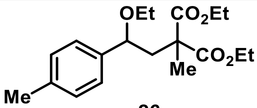


**26a**

**Yield:** 52% (35 mg).

**Nature:** Colourless oil

**$R_f$ value** = 0.30 [EtOAc:Petroleum ether = 1:19 (v/v)].

**$^1$H NMR (400 MHz, $CDCl_3$) $\delta$ (ppm):** 7.19 (d, $J$ = 8.0 Hz, 2H), 7.14 (d, $J$ = 8.0 Hz, 2H), 4.30 (dd, $J$ = 10.4, 2.6 Hz, 1H), 4.21 – 4.15 (m, 2H), 4.14 (q, $J$ = 7.2 Hz, 2H), 3.27 – 3.15 (m, 2H), 2.43 (dd, $J$ = 14.7, 10.5 Hz, 1H), 2.33 (s, 3H), 2.07 (dd, $J$ = 14.7, 2.8 Hz, 1H), 1.52 (s, 3H), 1.28 (t, $J$ = 7.2 Hz, 3H), 1.23 (t, $J$ = 7.2 Hz, 3H), 1.08 (t, $J$ = 7.0 Hz, 3H).

**$^{13}$C{$^1$H} NMR (101 MHz, $CDCl_3$) $\delta$ (ppm):** 172.7, 172.3, 139.9, 137.3, 129.2, 126.5, 78.2, 64.2, 61.3, 61.0, 52.5, 44.1, 21.2, 20.2, 15.2, 14.2, 14.1.

**HRMS** (ESI) *m/z* calcd for $C_{19}H_{28}O_5Na$ $[M+Na]^+$: 359.1834; found: 359.1837.

### 8.4.2. Cation Trapping Experiment with 1,2,4-Trimethoxybenzene:

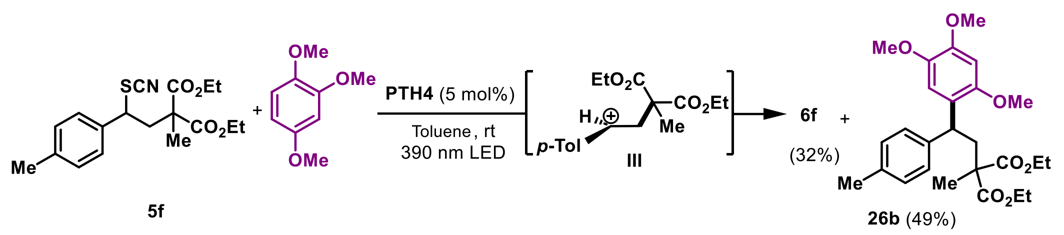


A culture tube equipped with a magnetic stir bar was charged with **PTH4** (3.7 mg, 0.01 mmol, 5 mol%), diethyl 2-methyl-2-(2-thiocyanato-2-(*p*-tolyl)ethyl)malonate **5f** (70 mg, 0.2 mmol), 1,2,4-trimethoxybenzene (149 µL, 1.0 mmol) and dry toluene (2 mL). The tube was sealed with a Teflon screw cap, evacuated, and backfilled with argon. Then, the reaction mixture was degassed by Freeze-Pump-Thaw cycles with argon and irradiated at rt with 390 nm LEDs at a distance of approximately 5 cm for 24 h. After completion of the reaction (confirmed by TLC), the crude was concentrated under vacuo, and purified by silica gel column chromatography (230−400 mesh) using EtOAc/petroleum ether as eluent to afford the product **26b** (49%) along with **6f** (32%).

**Diethyl 2-methyl-2-(2-(*p*-tolyl)-2-(2,4,5-trimethoxyphenyl)ethyl)malonate (26b):**

**Yield:** 49% (45 mg).

**Nature:** White solid.

**Mp:** 142 – 144 °C

**$R_f$ value** = 0.42 [EtOAc:Petroleum ether = 1:4 (v/v)].

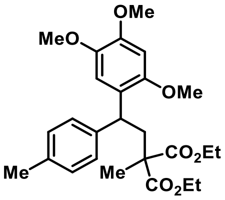


26b

**$^1$H NMR (400 MHz, $CDCl_3$) δ (ppm):** 7.18 (d, *J* = 8.1 Hz, 2H), 7.04 (d, *J* = 8.0 Hz, 2H), 6.80 (s, 1H), 6.45 (s, 1H), 4.48 (t, *J* = 7.0 Hz, 1H), 3.98 – 3.90 (m, 2H), 3.87 – 3.79 (m, 2H), 3.82 (s, 3H), 3.80 (s, 3H), 3.77 (s, 3H), 2.76 – 2.64 (m, 2H), 2.26 (s, 3H), 1.41 (s, 3H), 1.13 (t, *J* = 7.1 Hz, 3H), 1.12 (t, *J* = 7.1 Hz, 3H).

**$^{13}$C{$^1$H} NMR (101 MHz, $CDCl_3$) δ (ppm):** 172.2, 151.0, 148.0, 143.0, 142.0, 135.4, 128.9, 127.9, 124.9, 112.7, 98.0, 61.0, 56.8, 56.5, 56.2, 53.4, 40.0, 37.8, 21.0, 20.1, 13.9.

**HRMS** (ESI) *m/z* calcd for $C_{26}H_{34}O_7Na$ $[M+Na]^+$: 481.2202; found: 481.2204.

## Reaction with Aliphatic Alkenes:

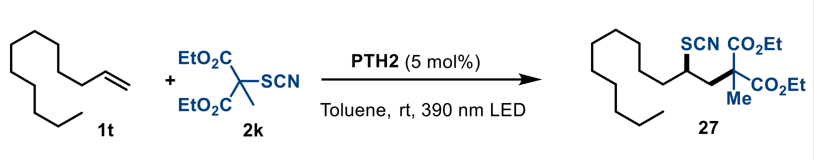


### Diethyl 2-methyl-2-(2-thiocyanatododecyl)malonate (27):

*By following the general procedure* ***GP-3****, the reaction between 1-dodecene* ***1t*** *(178 µL, 0.8 mmol) and 2-methyl-2-thiocyanatomalonate* ***2k*** *(46 mg, 0.2 mmol) provided the desired compound* ***27*** *after irradiation for 1 h with 390 nm LEDs.*

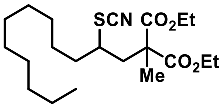

**27**

**Yield:** 85% (68 mg).

**Nature:** Colourless oil.

**$R_f$ value** = 0.30 [EtOAc:Petroleum ether = 1:9 (v/v)].

**$^1$H NMR (400 MHz, $CDCl_3$) $\delta$ (ppm):** 4.23 – 4.13 (m, 2H), 4.18 (q, $J$ = 7.1 Hz, 2H), 3.14 – 3.07 (m, 1H), 2.31 (d, $J$ = 6.1 Hz, 2H), 1.81 – 1.72 (m, 2H), 1.45 (s, 3H), 1.37 – 1.23 (m, 16H), 1.25 (t, $J$ = 7.1 Hz, 3H), 1.24 (t, $J$ = 7.1 Hz, 3H). 0.86 (t, $J$ = 6.8 Hz, 3H).

**$^{13}$C{$^1$H} NMR (101 MHz, $CDCl_3$) $\delta$ (ppm):** 171.5, 110.8, 61.9, 61.8, 52.7, 46.9, 41.3, 37.1, 32.0, 29.6, 29.5, 29.4, 29.3, 29.1, 26.9, 22.7, 20.7, 14.2, 14.0, 13.9.

**HRMS** (ESI) *m/z* calcd for $C_{21}H_{37}NO_4SNa$ [M+Na]$^+$: 422.2341; found: 422.2346.

## Isolation of By-product for Electron-Rich Aliphatic Alkenes:

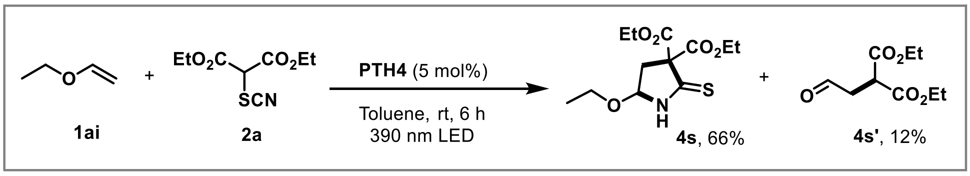


### Diethyl 2-(2-oxoethyl)malonate (4s'):[26]

**Yield:** 12% (5 mg) with 66% (38 mg) of **4s** following **GP-2**.

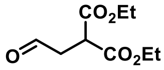

**4s'**

**Nature:** Colourless oil.

**$R_f$ value** = 0.44 [EtOAc:Petroleum ether = 1:4 (v/v)].

**$^1$H NMR (400 MHz, $CDCl_3$) $\delta$ (ppm):** 9.78 (s, 1H), 4.22 (q, $J$ = 7.1 Hz, 2H), 4.21 (q, $J$ = 7.1 Hz, 2H), 3.87 (t, $J$ = 7.0 Hz, 1H), 3.10 (d, $J$ = 7.0 Hz, 2H), 1.28 (t, $J$ = 7.2 Hz, 6H).

**$^{13}$C{$^1$H} NMR (101 MHz, $CDCl_3$) $\delta$ (ppm):** 198.3, 168.6, 62.1, 45.8, 42.5, 14.1.

## 8.5. Electrochemical Measurements:

Cyclic Voltammetry was performed using CH Instruments (model: CHI1140C) using a glassy carbon working electrode, saturated calomel reference electrode, and a platinum wire counter electrode. Samples were prepared with 2.0 mmol of substrate in 5 mL of 0.1 M tetrabutylammonium hexafluorophosphate ($TBAPF_6$) in dry and degassed acetonitrile. The potential was scanned at a scan rate 100 mV/s. A background of the electrolyte solution was subtracted from the voltammogram. Reduction was measured by scanning potential in the negative direction and oxidation in the positive direction. The glassy carbon electrode was polished between each scan. $E_{p/2}$ is given as the half-wave potential for irreversible reduction where the current is equal to one-half the peak current of the reduction event.

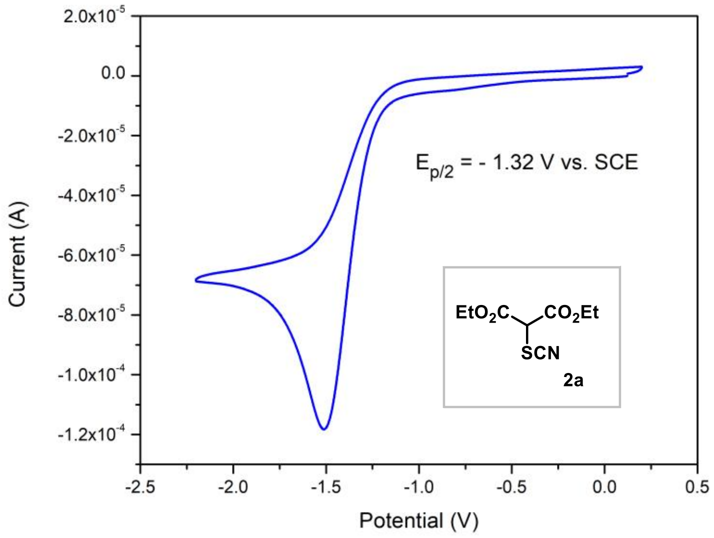


**Supplementary Fig. 11:** Cyclic voltammogram of **2a**

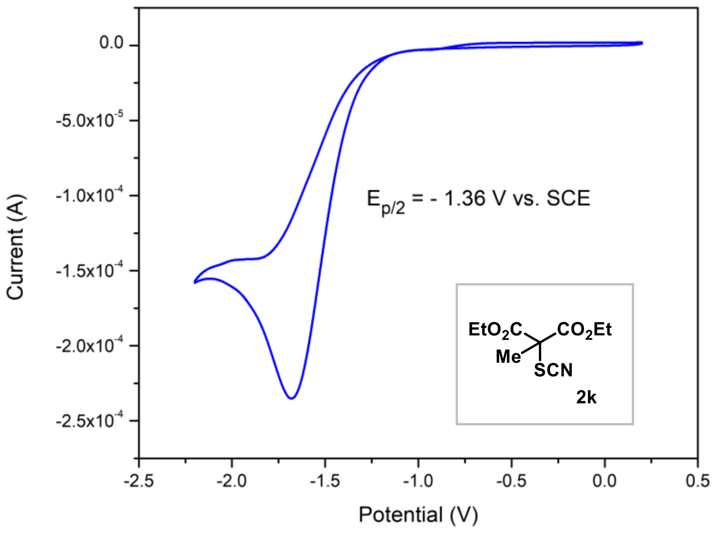


**Supplementary Fig. 12:** Cyclic voltammogram of **2k**

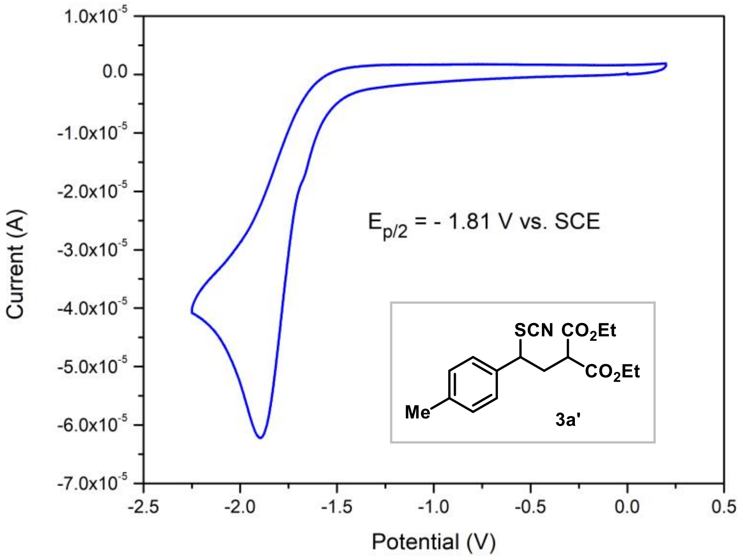


**Supplementary Fig. 13:** Cyclic voltammogram of **3a’**

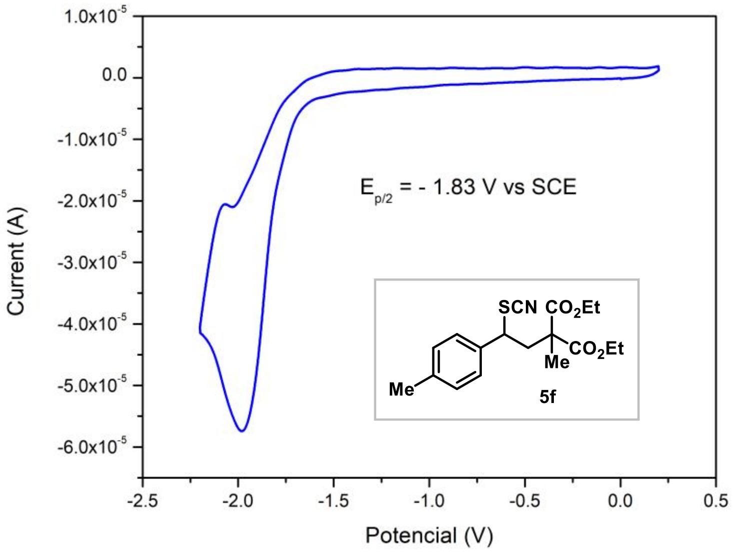


**Supplementary Fig. 14:** Cyclic voltammogram of **5f**

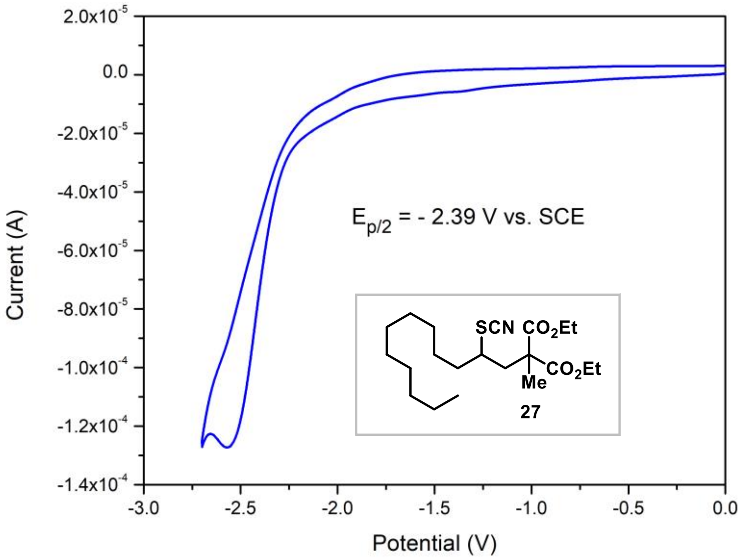


**Supplementary Fig. 15:** Cyclic voltammogram of **27**

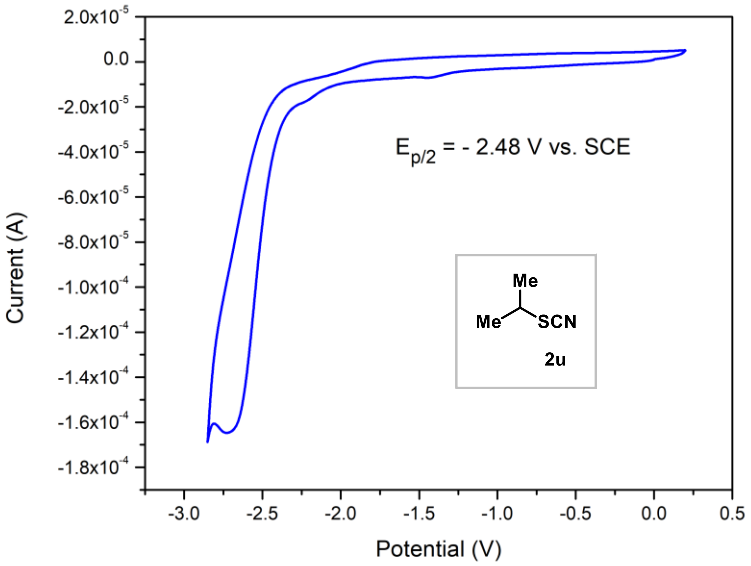


**Supplementary Fig. 16:** Cyclic voltammogram of **2u**

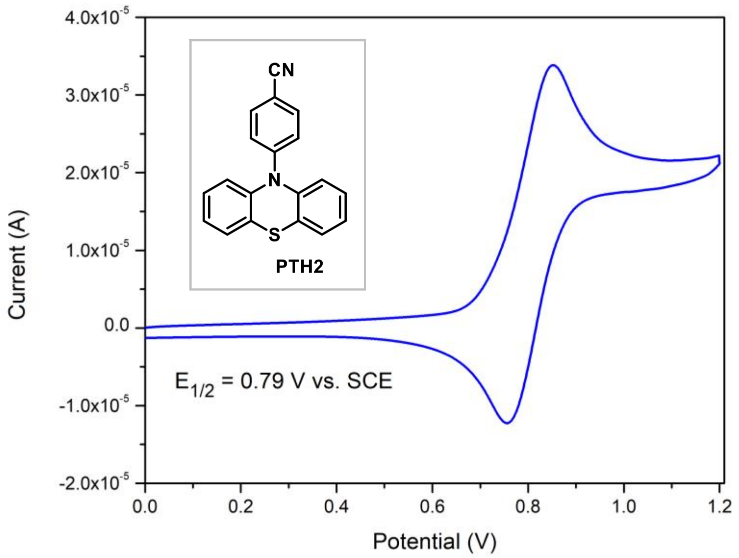


**Supplementary Fig. 17:** Cyclic voltammogram of **PTH2**

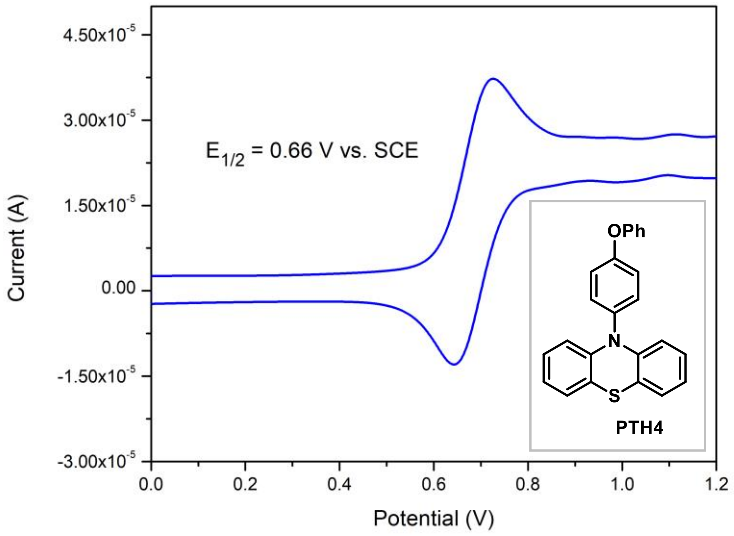


**Supplementary Fig. 18:** Cyclic voltammogram of **PTH4**

## 8.6. UV-Vis Experiments:

UV-Vis absorption spectra of organophotocatalysts were measured with 40 µM acetonitrile/dichloromethane solution.

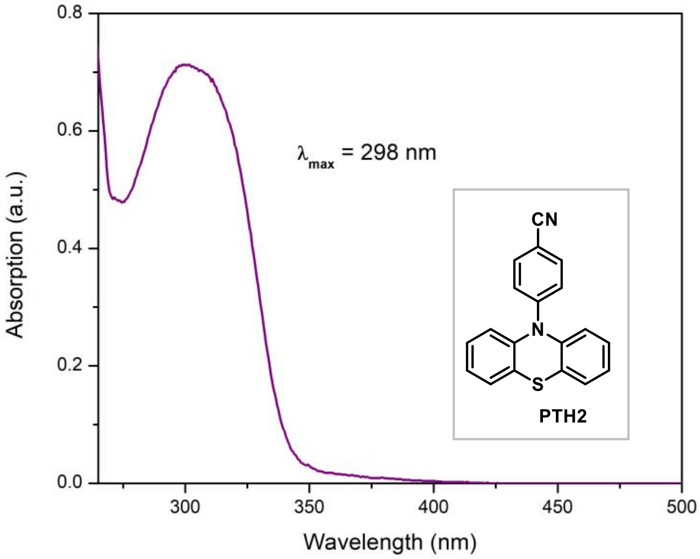


**Supplementary Fig. 19:** UV-Vis spectrum of **PTH2**

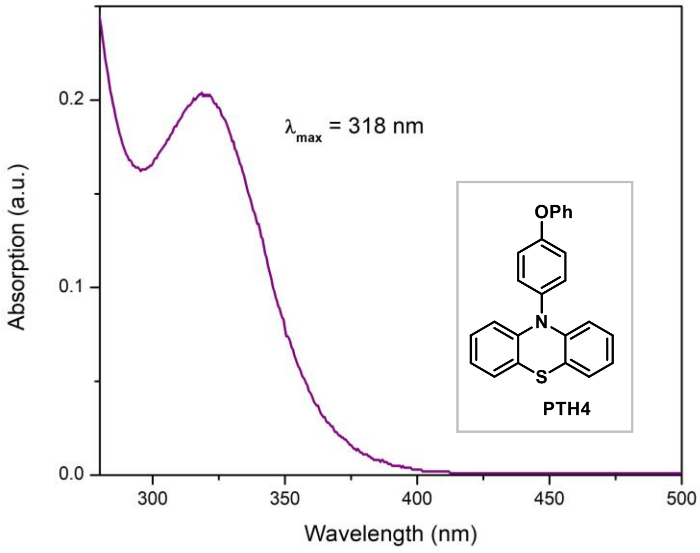


**Supplementary Fig. 20:** UV-Vis spectrum of **PTH4**

## 8.7. Photoluminescence Experiments:

Fluorescence spectra of organo-photocatalysts were measured with 40µM acetonitrile/dichloromethane solution.

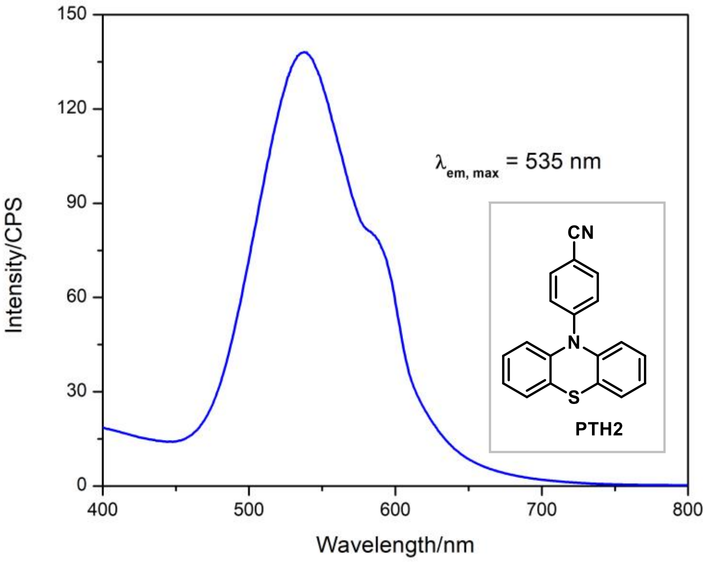


**Supplementary Fig. 21:** Fluorescence spectrum of **PTH2**

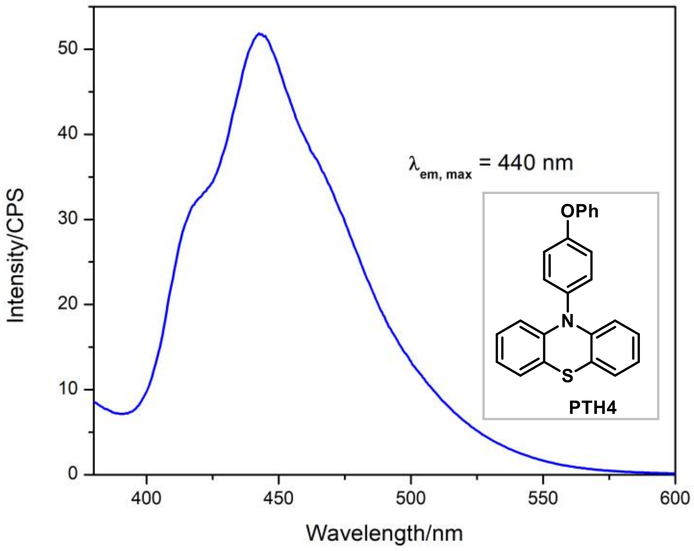


**Supplementary Fig. 22:** Fluorescence spectrum of **PTH4**

## Summary of Redox Properties of Organo-photocatalysts:[27,28]

| | **PTH1**[3] | **PTH2** | **PTH3**[28] | **PTH4** |
|---|---|---|---|---|
| $E_{1/2}$ ($PC^{\bullet+}/PC$) (V vs SCE)[a] | 0.84 | 0.79 | 0.68 | 0.66 |
| $\lambda_{max,\ em}$ (nm)[b] | 571 | 535 | 445 | 440 |
| $E_{S1,\ exp}$ (eV)[c] | 2.17 | 2.32 | 2.78 | 2.82 |
| $E^{0*}_{S1,\ exp}$ ($PC^{\bullet+}/PC^*$) (V vs SCE)[d] | -1.33 | -1.53 | -2.10 | -2.16 |

[a]Determined by CV experiments; [b]Determined by photoluminescence Experiments; [c]Singlet energies were calculated using the maximum wavelength of emission; [d]Singlet excited state reduction potentials were calculated using the singlet energies and the $E_{1/2}$.

## 8.8. Light ON/OFF Experiments:

Six reactions were set up in 0.2 mmol scale in reaction tube with diethyl 2-methyl-2-thiocyanatomalonate **2k**, 4-methylstyrene **1a**, and **PTH2** as mentioned in the standard reaction condition (**GP-3**). The resulting mixture was irritated with 390 nm LED light for 5 min. After 5 min, the LED irradiator was put off, and one reaction tube was removed from the irradiation setup. The remaining five tubes were stirred without light for an additional 5 min. Then, one tube was removed for analysis, and the irradiation source was turned on to irradiate the remaining four reaction mixtures. In this way, the alternately switch on and switch off of the irradiator was continued for 30 min. From the crude reaction mixture, the $^{1}H$ NMR yields were measured using 1,1,2,2-tetrachloroethane as internal standard.

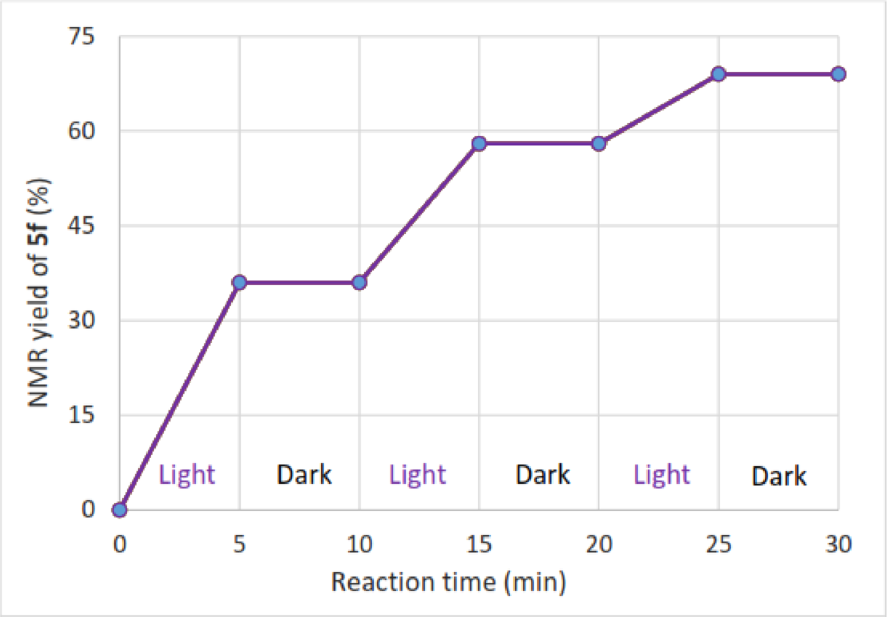


**Supplementary Fig. 23:** Light ON/OFF Experiments

**Conclusion:** The light on-off experiment shows that light irradiation was essential for the photo-catalytic alkylthiocyanation reaction of alkenes.

## 8.9. Impact of Light Intensity on Reaction Yield:

Four reactions were set up in 0.2 mmol scale in the reaction tube with diethyl 2-methyl-2-thiocyanatomalonate **2k**, 4-methylstyrene **1a**, and **PTH2** as mentioned in the standard reaction condition (**GP-3**). The resulting mixture was irritated with 390 nm LED light for 30 min with different intensities. PR160L-390 nm Kessil LEDs (max 52W intensity) were used for light sources with the controlling intensity. The $^1H$ NMR yields were measured using 1,1,2,2-tetrachloroethane as internal standard from the crude reaction mixture.

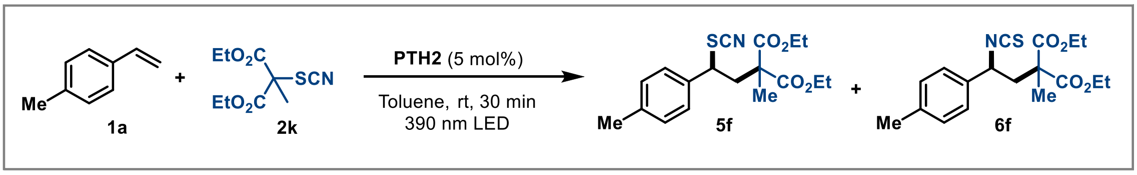


| Entry | Light intensity (%) | $^1H$ NMR yield of 5f/6f (%) |
|---|---|---|
| 1 | 25 | 17/trace |
| 2 | 50 | 46/trace |
| 3 | 75 | 68/3 |
| 4 | 100 | 87/5 |

**Supplementary Table 2:** Reaction yield with the changing the light intensity.

**Supplementary Discussion:** These results show that product yield increases with the higher light intensity. So, this chemo-divergent photocatalytic reaction depends on light intensity, i.e., photon flux.

## 8.10. UV-Visible Study for EDA Complex:

The UV−Vis absorption spectroscopic experiments were recorded using a quartz cuvette of 1.0 cm path length by Shimadzu UV-1800 Spectrophotometer. Initially, 0.1 mM solution of **PTH2** and **PTH4** photocatalyst, and 2 mM solution of **1a**, 1 mM solution of **2k**, and 1 mM solution of **5f** in acetonitrile were prepared and then absorbance of individual reacting components and their combinations were measured with the wavelength range from 280 to 530 nm.

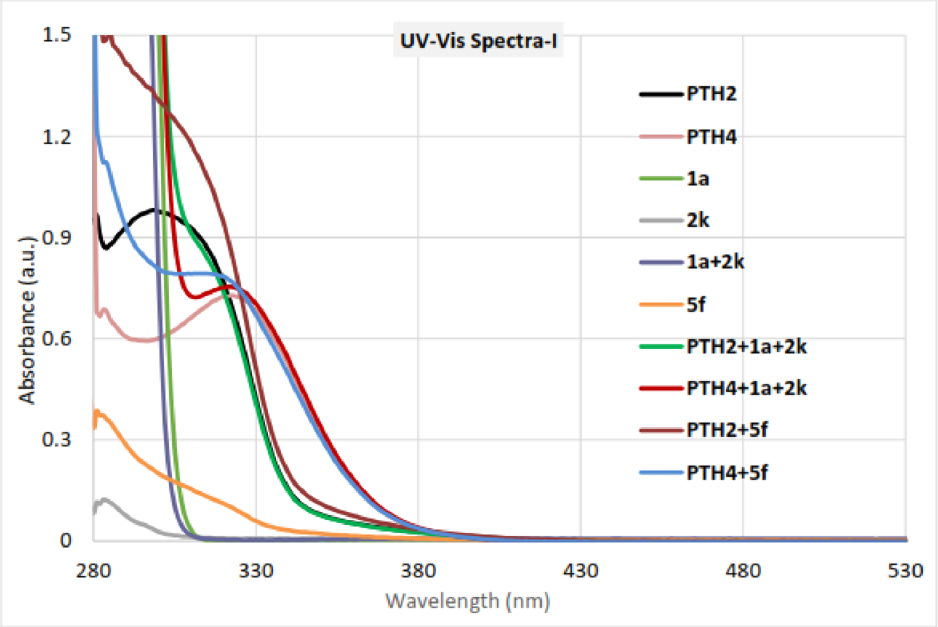


**Supplementary Fig. 24:** UV-Vis Spectra of **PTH2**, **PTH4**, individual reactants, **1a**, **2k**, **5f** and their combined mixtures.

**Supplementary Discussion:** These spectra clearly indicate that the photocatalyst (**PTH2** and **PTH4**) shows absorption exclusively at $\lambda_{max}$= 298 nm and 318 nm, and there is no new peak in the absorption spectra of the mixture of starting materials and also with/without photocatalyst. Hence, the formation of any electron donor-acceptor (EDA) complex is being ruled out.

## 8.11. UV-Visible Study for Direct Excitation of Compounds:

The emission spectrum of Kessil 390 nm LEDs was recorded using an Ocean Optics HR4000 High-Resolution Fiber Optic Spectrometer. Here, the $\lambda_{max}$ value of the Kessil LEDs (PR160L-390 nm) is 390 nm and presented in the below graph with the normalized value. Then, the UV−Vis absorption spectroscopic experiments were recorded using a quartz cuvette of 1.0 cm path length by Shimadzu UV-1800 Spectrophotometer. 2 mM solution of **1a**, 1 mM solution of **2k**, and 1 mM solution of **5f** in acetonitrile were prepared, and then absorbance of individual components were measured with the wavelength range from 280 to 515 nm.

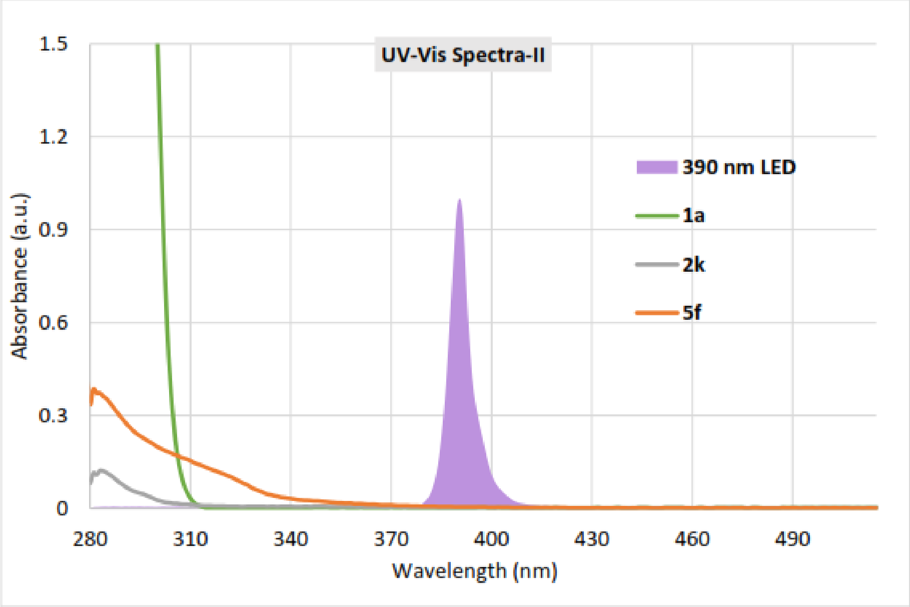


**Supplementary Fig. 25:** UV-Vis spectra of **1a**, **2k**, and **5f** with normalized emission spectrum of Kessil (PR160L-390 nm) LEDs.

**Supplementary Discussion:** These absorption spectra clearly show that reacting components didn't absorb light in the visible light region, especially the Kessil 390 nm LEDs emission region. Hence, the product formation from the reacting components under the direct irradiation of light (390 nm LEDs) without photocatalyst complex is being ruled out.

## 8.12. Stern-Volmer Fluorescence Quenching Experiments:

Fluorescence quenching studies were carried out using a Hitachi Fluorescence Spectrophotometer F-2700. Photocatalyst **PTH2** and varying concentrations of quencher were combined in dry and degassed dichloromethane in quartz cuvettes. For the quenching experiments, the concentration of the **PTH2** was 4.0 x $10^{-5}$ M. The solutions were irradiated at 298 nm, and the intensity of emission maxima was observed at 535 nm for **PTH2**. However, photocatalyst **PTH4** and varying concentrations of quencher were combined in dry and degassed acetonitrile in quartz cuvettes. For the quenching experiments, the concentration of the **PTH4** was also 4.0 x $10^{-5}$ M. The solutions were irradiated at 318 nm, and luminescence maxima was measured at 440 nm for PTH4. Plots were derived according to the Stern-Volmer equation, and $K_{sv}$ was calculated.

Stern-Volmer equation: $I_0/I = 1 + K_{sv}[Q]$

Where $I_0$ is the luminescence intensity without the quencher, I is the intensity with the quencher, [Q] is the concentration of added quencher, and $K_{sv}$ is the Stern-Volmer quenching constant. All the emission spectra were recorded after each addition of the quencher.

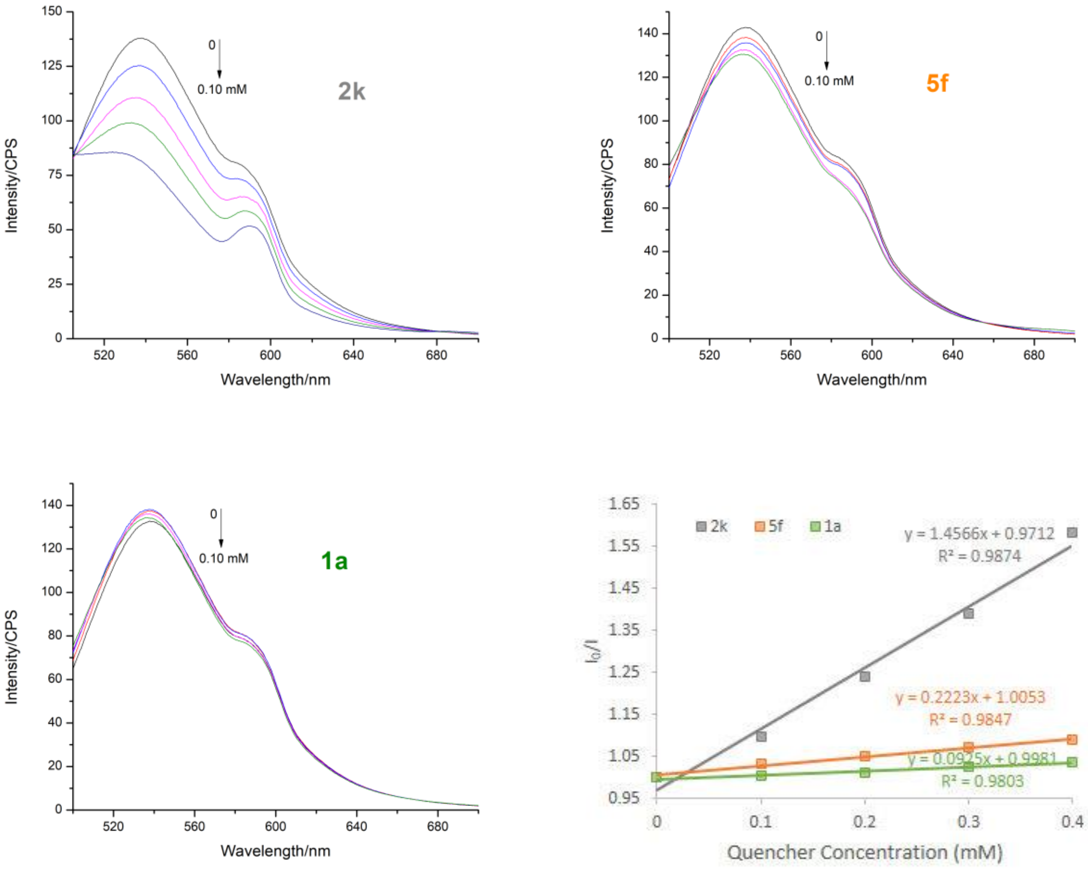


**Supplementary Fig. 26:** Emission spectra and Stern-Volmer plots of **PTH2**, quenching with varying concentrations of **2k**, **5f**, and **1a**.

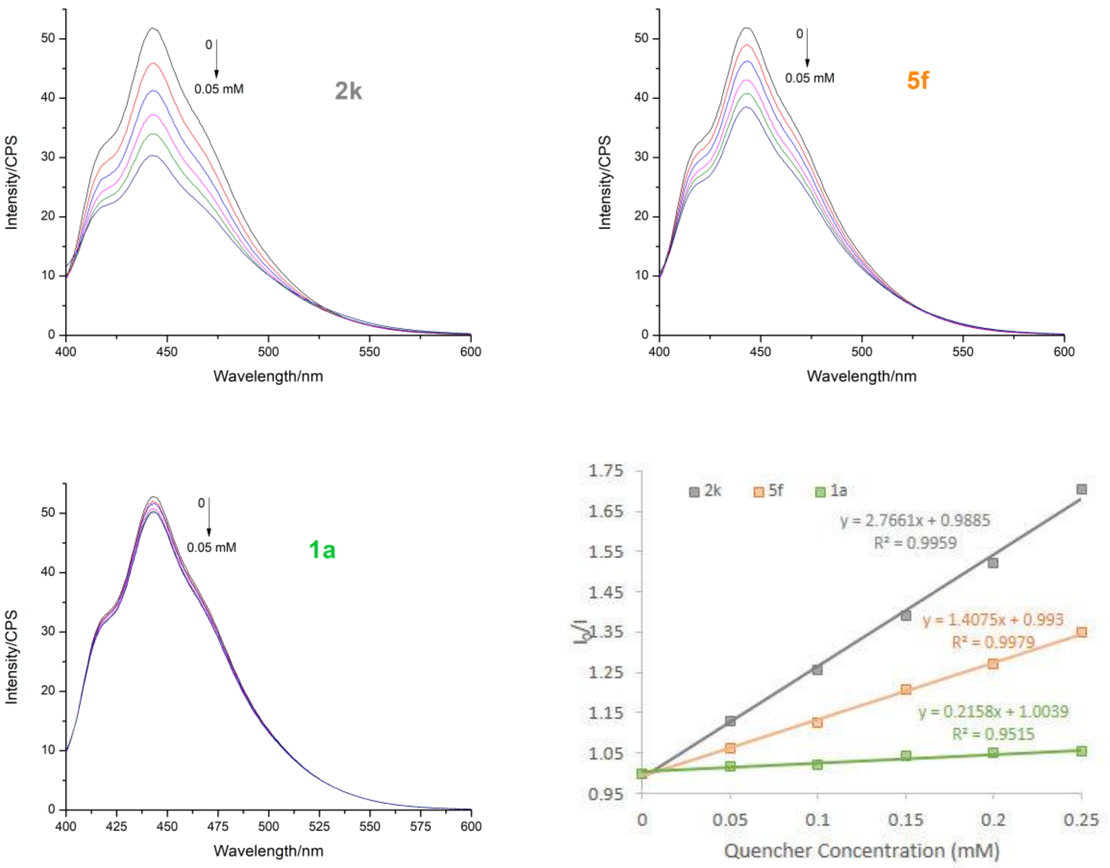


**Supplementary Fig. 27:** Emission spectra and Stern-Volmer plots of **PTH4**, quenching with varying concentrations of **2k**, **5f**, and **1a**.

**Conclusion:** These results show that while both photocatalysts are readily quenched by **2k**, but only **PTH4** is efficiently quenched by **5f**, which accounts for the chemo-divergency in the product formation.

## 8.13. Investigation into Catalyst Activation Modes:

### For Atom Transfer Radical Addition (ATRA) Event:

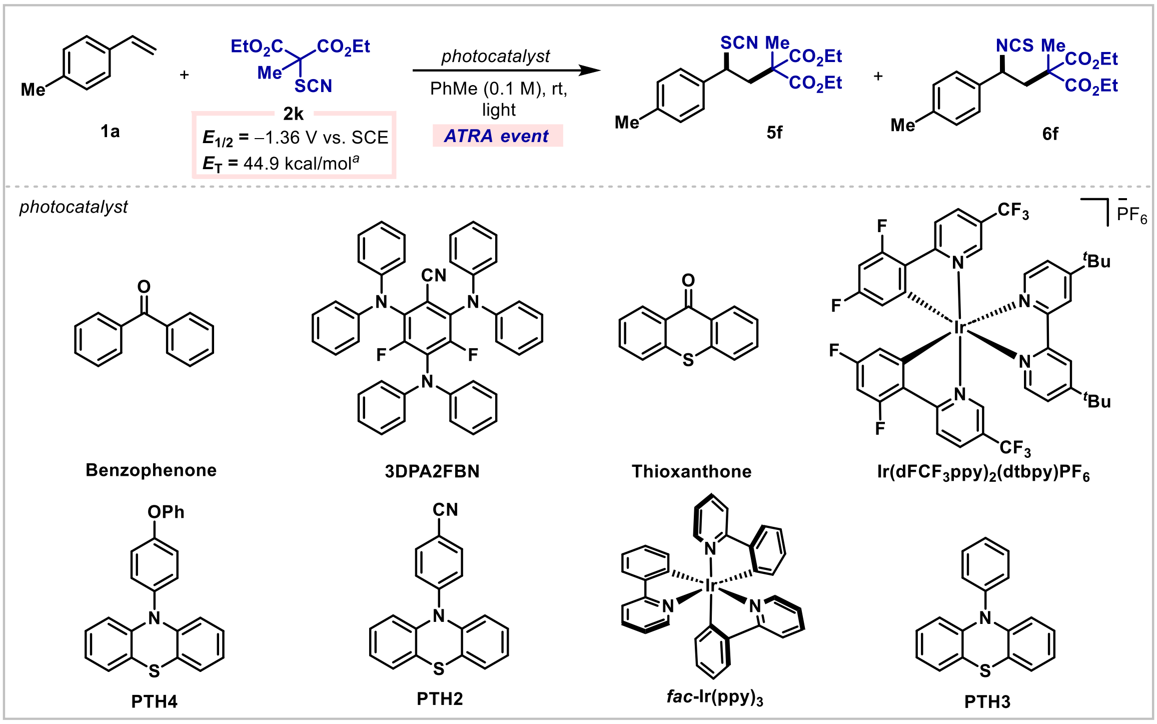


| entry | photocatalyst (x mol%) | $E_T$ (kcal/mol) | $E_{1/2}$ ($M^{\bullet+}/M^*$) V vs. SCE | Light (nm) | ATRA output | |
|---|---|---|---|---|---|---|
| | | | | | t (h) | **5f/6f** (%)[b] |
| 1 | Benzophenone (5) | 69.1[29] | – 0.61 | 370 | 6 | 4/0 |
| 2 | 3DPA2FBN (5) | 65.5[30] | – 1.60 | 390 | 6 | 32/3 |
| 3 | Thioxanthone (5) | 63.4[29] | – 1.11 | 390 | 6 | 3/0 |
| 4 | $Ir(dFCF_3ppy)_2(dtbpy)PF_6$ (1) | 61.8[29] | – 0.89 | 450 | 6 | 6/0 |
| 5 | PTH4 (5) | 58.4[a] | – 2.16 | 390 | 0.5 | 57/32 |
| 6 | PTH2 (5) | 58.1[a] | – 1.53 | 390 | 0.5 | 87/5 |
| 7 | *fac*-$Ir(ppy)_3$ (1) | 58.1[29](57.9)[a] | – 1.73 | 450 | 6 | 68/5 |
| 8 | PTH3 (5) | 55.3[31] | – 2.10 | 390 | 0.5 | 51/30 |

[a]computationally calculated value; [b]crude $^1H$ NMR yield using 1,1,2,2-tetrachloroethane as internal standard.

**Supplementary Table 3:** Photocatalytic reaction output of ATRA event.

**For Isomerization Event:**

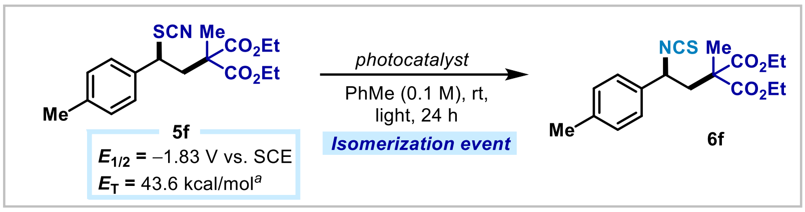


| entry | photocatalyst (x mol%) | $E_T$ (kcal/mol) | $E_{1/2}$ ($M^{\bullet+}/M^*$) V vs. SCE | Light (nm) | Isomerization output **6f** (%)[b] |
|---|---|---|---|---|---|
| 1 | Benzophenone (5) | 69.1[29] | – 0.61 | 370 | 3 |
| 2 | 3DPA2FBN (5) | 65.5[30] | – 1.60 | 390 | 2 |
| 3 | Thioxanthone (5) | 63.4[29] | – 1.11 | 390 | 3 |
| 4 | $Ir(dFCF_3ppy)_2(dtbpy)PF_6$ (1) | 61.8[29] | – 0.89 | 450 | trace |
| 5 | PTH4 (5) | 58.4[a] | – 2.16 | 390 | 85 |
| 6 | PTH2 (5) | 58.1[a] | – 1.53 | 390 | 5 |
| 7 | *fac*-$Ir(ppy)_3$ (1) | 58.1[29](57.9)[a] | – 1.73 | 450 | 6 |
| 8 | PTH3 (5) | 55.3[31] | – 2.10 | 390 | 81 |

[a]computationally calculated value; [b]crude $^1H$ NMR yield using 1,1,2,2-tetrachloroethane as internal standard.

**Supplementary Table 4:** Photocatalytic reaction output of isomerization event.

**Supplementary Discussion**: These two tables clearly indicate that the SET process is only responsible for the ATRA and isomerization event. However, the triplet energy of the photocatalyst is higher than the reactive materials, and a productive event is only possible when the reduction potential of the photocatalyst exceeds that of the starting materials.

## 8.14. Determination of the Reaction Quantum Yield:

The emission spectrum of Kessil 390 nm LEDs for quantum yield experiment was recorded using an Ocean Optics HR4000 High-Resolution Fiber Optic Spectrometer. Here, the $\lambda_{max}$ value of the Kessil LEDs (PR160L-390 nm)

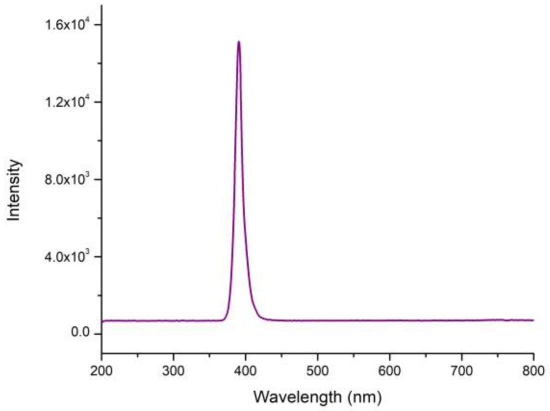


**Supplementary Fig. 28:** Emission spectrum of the Kessil LEDs (PR160L-390 nm)

**Determination of the light intensity at 390 nm:**

According to the procedure of T. P. Yoon,[32] the photon flux of the LED ($\lambda_{max}$ = 390 nm) was determined by the standard ferrioxalate actinometry. 0.15 M solution of ferrioxalate was prepared by dissolving 2.21 g of potassium ferrioxalate hydrate in 30 mL of 0.05 M $H_2SO_4$. A buffered solution of phenanthroline was prepared by dissolving 50 mg of phenanthroline and 11.25 g of sodium acetate in 50 mL of 0.5 M $H_2SO_4$. Both solutions were stored in the dark. To determine the photon flux of the spectrophotometer, 2.0 mL of the ferrioxalate solution was placed in a cuvette and irradiated for 45.0 seconds at λ = 390 nm. After irradiation, 0.35 mL of the phenanthroline solution was added to the cuvette. The solution was then allowed to rest for 1 h to allow the ferrous ions to completely coordinate with the phenanthroline. The absorbance of the solution was measured at 510 nm. A non-irradiated sample was also prepared, and the absorbance at 510 nm was measured. Conversion was calculated using eq 1.

$$\text{mol Fe}^{2+} = \frac{\text{V} \bullet \Delta\text{A (510 nm)}}{\text{l} \bullet \varepsilon \text{ (510 nm)}} \qquad (1)$$

Where V is the total volume (0.00235 L) of the solution after the addition of phenanthroline, ΔA is the difference in absorbance at 510 nm between the irradiated and non-irradiated solutions, l is the path length (1 cm), and ε is the molar absorptivity at 510 nm (11,100 L $mol^{-1}cm^{-1}$).[33] The photon flux can be calculated using eq 2.

$$\text{Photon flux} = \frac{\text{mol Fe}^{2+}}{\Phi \bullet \text{t} \bullet \text{f}} \qquad (2)$$

Where Φ is the quantum yield for the ferrioxalate actinometer (1.17 for a 0.15 M solution),[33] t is the time (45 s), and f is the fraction of light absorbed at λ = 390 nm by the ferrioxalate actinometer. The value is calculated using eq 3, where $A_{390\ nm}$ is the absorbance of the ferrioxalate solution at 390 nm. An absorption spectrum gave an $A_{390\ nm}$ value of > 3, indicating that the fraction of absorbed light (f) is > 0.999.

$$f = 1 - 10^{-A_{390\ nm}} \qquad (3)$$

Sample calculation:

$$\text{mol } Fe^{2+} = \frac{0.00235 \text{ L} \bullet 1.008}{1.00 \text{ cm} \bullet 11{,}100 \text{ L mol}^{-1} \text{ cm}^{-1}} = 2.13 \text{ X } 10^{-7} \text{ mol}$$

$$\text{Photon flux} = \frac{2.13 \text{ X } 10^{-7} \text{ mol}}{1.17 \bullet 45\ s \bullet 0.999} = 4.05 \text{ X } 10^{-9} \text{ einstein } s^{-1}$$

The photon flux was calculated (average of three experiments) to be 4.05 × $10^{-9}$ einstein $s^{-1}$.

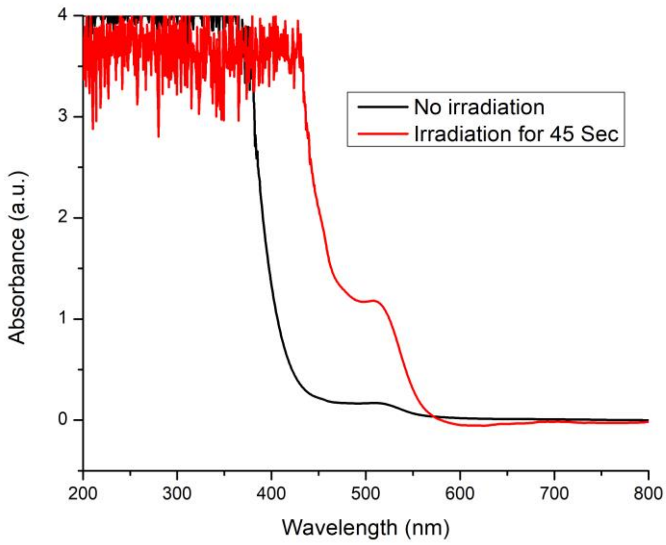


**Supplementary Fig. 29:** UV-Vis spectra of irradiated and non-irradiated ferrioxalate actinometer solutions.

**Determination of quantum yield for aromatic alkenes:**

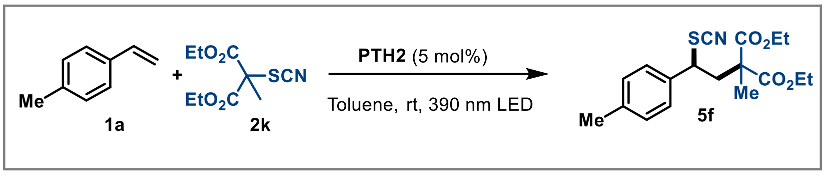


A quartz cuvette with two sides taped over with electrical tape equipped with a magnetic stir bar was charged with **PTH2** (3 mg, 0.01 mmol, 5 mol%), diethyl 2-methyl-2-thiocyanatomalonate **2k** (46 mg, 0.2 mmol) and dry toluene (2 mL). The quartz cuvette was capped before 4-methylstyrene **1a** (53 µL, 0.4 mmol) was added to it. Then, the reaction mixture was degassed with argon and irradiated at rt with 390 nm LEDs at a distance of approximately 5 cm for 20 min. A high-speed fan was used to maintain the temperature. After 20 min, reaction crude was concentrated, and the $^{1}H$ NMR yield was measured using 1,1,2,2-tetrachloroethane as internal standard for the corresponding alkylthiocyanation product **5f**.

Yield of the product **5f** is 79% ($15.8\times10^{-5}$ mol), where the photon flux is $4.05\times10^{-9}$ einstein $s^{-1}$ (described above), t is the reaction time (1200 s) and f is the fraction of incident light absorbed by the reaction mixture. The absorbance of the reaction mixture at 390 nm was measured to be 0.925.

$$\Phi = \frac{\text{mol product}}{\text{flux} \bullet \text{t} \bullet \text{f}} \qquad (4)$$

$$\Phi = \frac{15.8 \text{ X } 10^{-5} \text{ mol}}{4.05 \text{ X } 10^{-9}\text{einstein } s^{-1} \bullet 1200\ s \bullet 0.881} = 36.9$$

The reaction quantum yield (Φ) of aromatic alkene was thus determined to be 36.9.

**Determination of quantum yield for aliphatic alkenes:**

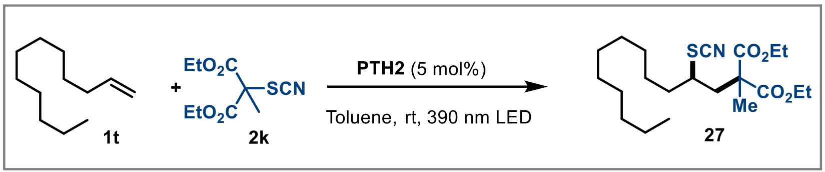


A quartz cuvette with two sides taped over with electrical tape equipped with a magnetic stir bar was charged with **PTH2** (3 mg, 0.01 mmol, 5 mol%), diethyl 2-methyl-2-thiocyanatomalonate **2k** (46 mg, 0.2 mmol) and dry toluene (2 mL). The quartz cuvette was capped before 1-dodecene **1t** (178 µL, 0.8 mmol) was added to it. Then, the reaction mixture was degassed with argon and irradiated at rt with 390 nm LEDs at a distance of approximately 5 cm for 20 min. A high-speed fan was used to maintain the temperature. After 20 min, reaction crude was concentrated, and the $^{1}$H NMR yield was measured using 1,1,2,2-tetrachloroethane as internal standard for the corresponding alkylthiocyanation product **27**.

Yield of the product **27** is 54% ($10.8\times10^{-5}$ mol), where the photon flux is $4.05\times10^{-9}$ einstein $s^{-1}$ (described above), t is the reaction time (1200 s), and f is the fraction of incident light absorbed by the reaction mixture. An absorbance of the reaction mixture at 390 nm was measured to be 0.925

$$\Phi = \frac{\text{mol product}}{\text{flux} \bullet \text{t} \bullet \text{f}} \qquad (4)$$

$$\Phi = \frac{10.8 \text{ X } 10^{-5} \text{ mol}}{4.05 \text{ X } 10^{-9}\text{einstein } s^{-1} \bullet 1200\ s \bullet 0.881} = 25.2$$

The reaction quantum yield (Φ) for aliphatic alkene was thus determined to be 25.2.

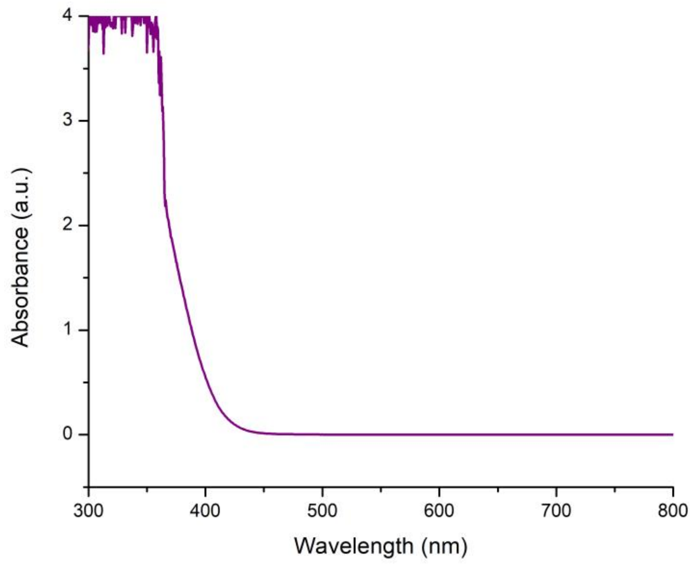


**Supplementary Fig. 30:** UV-Vis spectrum of 5×10$^{-3}$ M solution of **PTH2** in toluene.

## 8.15. Additional Reaction Mechanism:

| ***Alkene substrate*** | ***Condition A (with PTH2)*** | ***Condition B (with PTH4)*** | ***Comments*** |
|---|---|---|---|
| 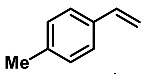 **1a** | 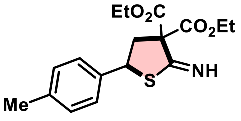 **3a** (89%) + **4a** (7%) | 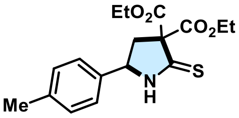 **4a** (81%) with trace amount of *S*-cyclization | *S*- and *N*-heterocycles were formed predominantly by single or double catalytic cycle due to redox potential matching with **PTH2** and **PTH4**. |
| 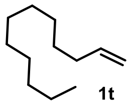 **1t** | 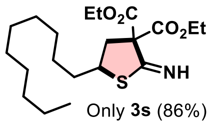 Only **3s** (86%) | *Only S-cyclization product observed* | The reduction potential of the catalyst (**PTH4**) was insufficient to reductively split 1st cycle carbothiocyantion product to initiate the 2nd cycle of isomerization. |
| 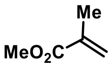 **1ag** | 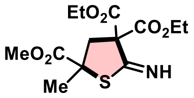 Only **3af** (52%) | *Only S-cyclization product **3af** observed* | The non-isomerization of acrylate derived-thiocyanate in 2nd photoredox is presumably because radical oxidation (to carbocation) adjacent to the ester is relatively difficult.[34] |
| 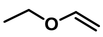 **1ai** | *Trace amount of S-cyclization with major amount of **4r*** | 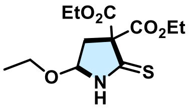 **4s** (68%) with trace amount of *S*-cyclization | Due to the formation of stable α-alkoxyalkyl carbocation after the addition of malonyl radical to activated aliphatic alkenes, isothiocyanate was produced as a thermodynamically control product. |

**Supplementary Table 5:** Summary of substrates with chemodivergent products.

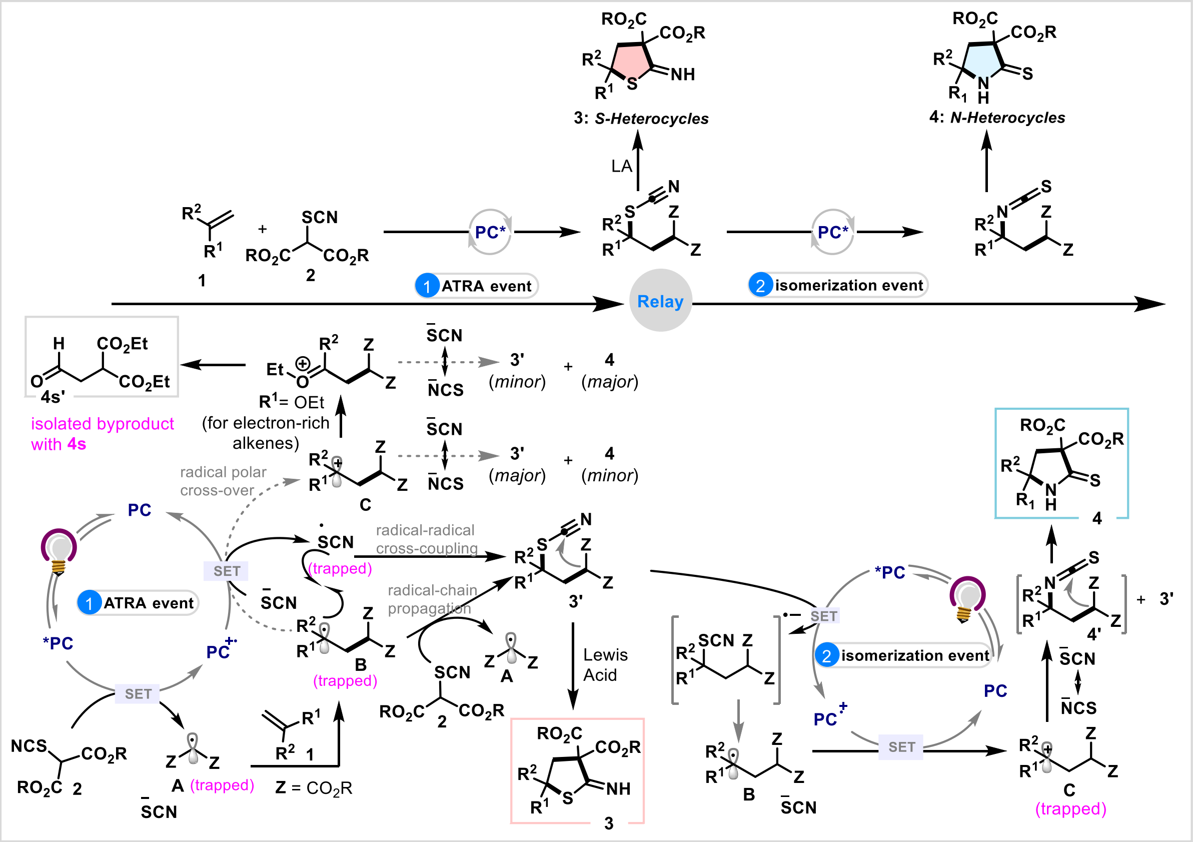

**Supplementary Discussion:** From the above substrate-dependent outcome, a possible reaction mechanism for the oxidative quenching of **2** and olefin involves visible light-mediated initiation of a radical-chain propagation to provide ATRA product **3'**. Termination of the propagation *via* oxidation of thiocyanate anion to thiocyanate radical ($E_{SCN•/SCN^-}$ = 0.62 V *vs*. SCE) by the catalyst ($E_{PC•+/PC}$ = 0.79 V *vs*. SCE for **PTH2**) additionally provides the carbothiocyanation product **3'** by radical-radical coupling and simultaneously regenerates the ground state photocatalyst. Alternatively, a radical polar cross-over termination through carbocation **C** is also probable. This radical-polar termination is reasonable when the alkene is styrene-derived; it is not suitable for aliphatic alkenes and acrylate. Oxidation to the cation requires for radical **B** to encounter $PC^{•+}$ in solution, both of which will be present in extremely low concentrations. Oxidation of **B** is still certainly feasible if it is a benzylic radical, as it will be sufficiently stabilized and long enough lived to encounter $PC^{•+}$. However, a non-stabilized, aliphatic secondary radical will not. For the aliphatic cases, it is much more likely to react with **2**, which will be present in a much higher concentration. Specifically, the reaction likely proceeds through chain propagation. The measured quantum yield for both aliphatic ($\Phi$ =25.2) and aromatic alkenes ($\Phi$ = 36.9) are in line with the radical chain process. For aromatic alkenes, the formation of a trace amount of *N*-heterocycle (7%) indeed indicates a certain extent of radical-polar mechanism also operating parallel in the ATRA step, but we did not identify any *N*-product for aliphatic alkenes and acrylate.

In the isomerization cycle with catalyst **PTH4**, oxidative radical polar cross-over converts thiocyanate **3'** to carbocation **C** through the radical **B**. Now, nucleophilic trapping of carbocation **C** with isothiocyanate anion transforms **3'** slowly to thermodynamically more stable thiocyanate **4'**, which spontaneously cyclized to *N*-heterocycles **4** in the reaction medium. The reductive cleavage of **3'** to **B** is the key for the initiation of 2$^{nd}$ photoredox cycle. Although this initiation of 2$^{nd}$ cycle is easy for benzylic thiocyanates ($E_{1/2}^{red}$ = -1.81 V vs. SCE), the corresponding photoredox reduction for aliphatic thiocyanate ($E_{1/2}^{red}$= -2.39 V *vs*. SCE) is relatively difficult with the current photocatalyst ($E_{1/2}^{*}$ = −2.16 V *vs.* SCE for **PTH4**), which probably explains the non-isomerization of aliphatic thiocyanates to isothiocyanates and thereby their limitation in *N*-heterocycles formation. But in case of electron-rich aliphatic alkenes, single catalytic cycle provide *N*-heterocycles because oxidation of α-alkoxyalkyl radical after malonyl radical addition to alkene is much easier for the formation of stable carbocation,[35] which was trapped by the *N*-site of SCN leading to thermodynamically stable *N*-heterocycles.

Therefore, in this photoredox cascade catalysis, the carbothiocyanation with ATRA event involves in radical propagation and/or radical polar cross-over where isomerization to thiocyanates involves only in radical polar cross-over cycle, but the overall yield depends on the nature of the substrates.

## 9. Incompatible Substrates:

The following substrates failed to undergo only one or neither of the desired reactions.

| *Substrate* | *Condition A* (with PTH2) | *Condition B* (with PTH4) | *Comments* |
|---|---|---|---|
| 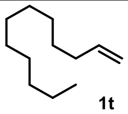 1t | 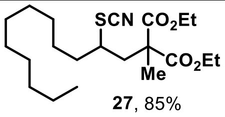 27, 85% | *only ATRA product observed* | ATRA product was observed with excellent yield in both conditions, but isomerization didn't take place because of the higher reduction potential of alkylthiocyanate **27** ($E_{1/2}$ = -2.39 V *vs.* SCE) than the working photocatalyst **PTH4** ($E_{1/2}$* = -2.16 V *vs.* SCE). |
| 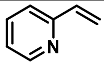 | *complex mixture* | *complex mixture* | A complex mixture was observed after the reaction with malonate **2a** in standard conditions, which is difficult to isolate. |
| 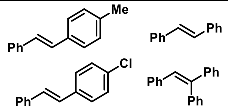 | *no reaction* | *no reaction* | No reaction takes place with diethyl 2-thiocyanatomalonate (**2a**) under optimized condition. It may be due to the steric repulsion by bulky aryl groups of alkenes to the malonyl radical addition. |
| 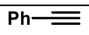 or 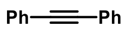 | *complex mixture* | *complex mixture* | A complex mixture was observed after the reaction with malonate **2a** in standard conditions, which is difficult to isolate. |
| 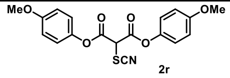 2r | *complex mixture* | *complex mixture* | A complex mixture was observed after the reaction with styrene **1b** in standard conditions, which is difficult to isolate. |
| 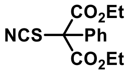 2s | 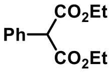 78% | *only diethyl 2-phenylmalonate observed* | Diethyl 2-phenylmalonate was afforded instead of the desired product after the reaction with 4-methylstyrene **1a** under standard conditions. After photo-irradiation, destabilized diethyl 2-phenylmalonate radical/ doubly destabilized carbocation was formed, and quenching by the solvent afforded the mentioned product. |
| 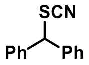 2t | *no reaction* | 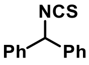 57% | After the reaction with styrene **1b**, we isolated the product (isothiocyanatomethylene)dibenzene with a moderate yield. Such benzhydryl radical can easily be oxidized to highly stabilized carbocations which was trapped by the isothiocyanate anion and leading the isomerized isothiocyanate product. |
| 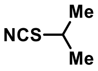 2u | *no reaction* | *no reaction* | No reaction occurred with styrene **1b**, due to the higher reduction potential of **2u** ($E_{1/2}$ = -2.48 V *vs.* SCE) than the existing photo-catalysts (**PTH2**, $E_{1/2}$* = -1.53 V and **PTH4**, $E_{1/2}$* = -2.16 V *vs.* SCE). |

**Supplementary Table 6:** Summary of incompatible substrates.

## Diethyl 2-phenylmalonate:[36]

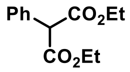

**Yield:** 78% (37 mg).

**Nature:** Colourless oil.

**$R_f$ value** = 0.42 [EtOAc:Petroleum ether = 1:9 (v/v)].

**$^1$H NMR (400 MHz, $CDCl_3$) $\delta$ (ppm):** 7.42 – 7.31 (m, 5H), 4.62 (s, 1H), 4.26 – 4.17 (m, 4H), 1.26 (t, $J$ = 7.1 Hz, 6H).

**$^{13}C\{^1H\}$ NMR (101 MHz, $CDCl_3$) $\delta$ (ppm):** 168.3, 132.9, 129.4, 128.7, 128.3, 61.9, 58.1, 14.1.

## (Isothiocyanatomethylene)dibenzene:[37]

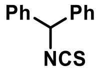

**Yield:** 57% (26 mg).

**Nature:** White solid.

**$R_f$ value** = 0.34 [EtOAc:Petroleum ether = 1:19 (v/v)].

**$^1$H NMR (400 MHz, $CDCl_3$) $\delta$ (ppm):** 7.42 – 7.37 (m, 4H), 7.35 – 7.29 (m, 6H), 6.00 (s, 1H).

**$^{13}C\{^1H\}$ NMR (101 MHz, $CDCl_3$) $\delta$ (ppm):** 139.3, 134.5, 129.1, 128.4, 126.7, 64.7.

## 10. X-ray Crystal Structures and Data:

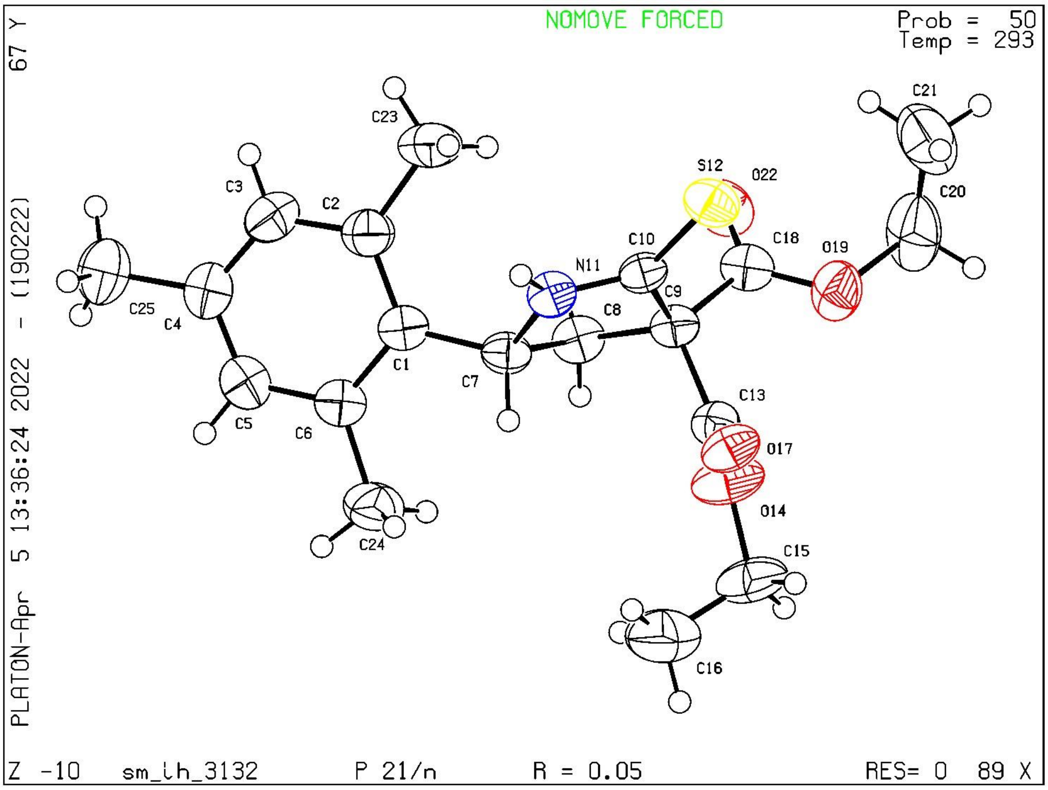


**Supplementary Fig. 31:** ORTEP plot of compound **4k** with 50% ellipsoid probability.

**Crystal data for 4k**: X-ray single crystal data were collected using MoKα (λ = 0.71073 Å) radiation on a Rigaku SuperNova diffractometer equipped with an Eos S2 detector. Structure solution/refinement was carried out using Shelx-2013. The structure was solved by direct method and refined in a routine manner. Non-hydrogen atoms were treated anisotropically. All hydrogen atoms were geometrically fixed. CCDC (**CCDC No: 2164590**) contains the supplementary crystallographic data of **4k**. These data can be obtained free of charge via www.ccdc.cam.ac.uk/conts/retrieving.html (or from the Cambridge Crystallographic Data Centre, 12 Union Road, Cambridge CB21EZ, UK; fax: (+44) 1223-336-033; or deposit@ccdc.cam.ac.uk).

**Supplementary Table 7: Crystal data and structure refinement for compound 4k**

| | |
|---|---|
| Identification code | CCDC 2164590 |
| Empirical formula | $C_{19}H_{25}NO_4S$ |
| Formula weight | 363.46 |
| Temperature/K | 293(2) |
| Crystal system | monoclinic |
| Space group | P 21/n |
| a/Å | 11.1821(7) |

| | |
|---|---|
| b/Å | 10.7510(8) |
| c/Å | 16.5882(9) |
| α/° | 90 |
| β/° | 96.802(5) |
| γ/° | 90 |
| Volume/Å$^3$ | 1980.2(2) |
| Z | 4 |
| $\rho_{calc}$g/cm$^3$ | 1.219 |
| μ/mm$^{-1}$ | 0.185 |
| F(000) | 776 |
| Radiation | MoKα (λ = 0.71073) |
| Theta (mix) | 2.087 |
| Theta (max) | 29.517 |
| h, k, lmax | 14, 13, 22 |
| R (reflections) | 0.0504 (2948) |
| wR2 (reflections) | 0.1337 (4603) |

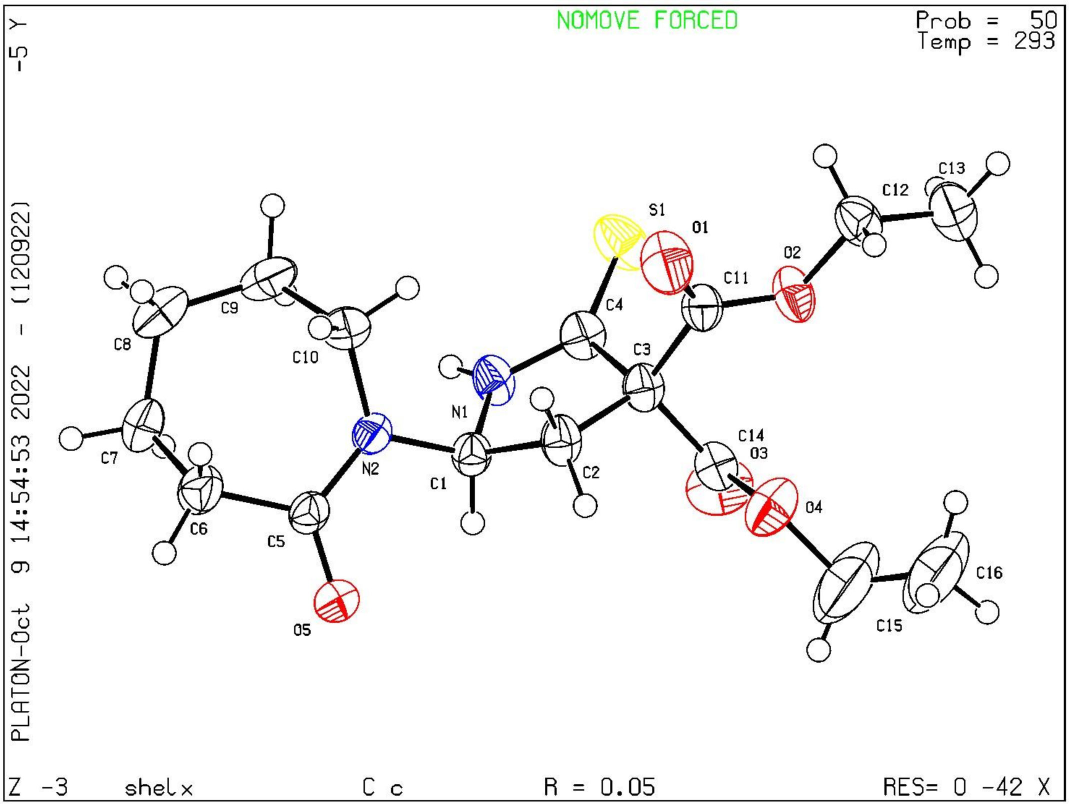


**Supplementary Fig. 32:** ORTEP plot of compound **4z** with 50% ellipsoid probability.

**Crystal data for 4z**: X-ray single crystal data were collected using MoKα (λ = 0.71073 Å) radiation on a Rigaku SuperNova diffractometer equipped with an Eos S2 detector. Structure solution/refinement was carried out using Shelx-2013. The structure was solved by direct method and refined in a routine manner. Non-hydrogen atoms were treated anisotropically. All hydrogen atoms were geometrically fixed. CCDC (**CCDC No: 2182605**) contains the supplementary crystallographic data of **4z**. These data can be obtained free of charge via www.ccdc.cam.ac.uk/conts/retrieving.html (or from the Cambridge Crystallographic Data Centre, 12 Union Road, Cambridge CB21EZ, UK; fax: (+44) 1223-336-033; or deposit@ccdc.cam.ac.uk).

**Supplementary Table 8: Crystal data and structure refinement for compound 4z**

| | |
|---|---|
| Identification code | CCDC 2182605 |
| Empirical formula | $C_{16}H_{22}N_2O_5S$ |
| Formula weight | 356.43 |
| Temperature/K | 293(2) |
| Crystal system | monoclinic |
| Space group | P 21/n |
| a/Å | 13.8149(6) |
| b/Å | 13.0683(5) |
| c/Å | 10.2557(4) |
| α/° | 90 |
| β/° | 98.044(4) |
| γ/° | 90 |
| Volume/Å$^3$ | 1833.32(13) |
| Z | 4 |
| $\rho_{calc}$g/cm$^3$ | 1.291 |
| μ/mm$^{-1}$ | 0.203 |
| F(000) | 760.0 |
| Radiation | MoKα (λ = 0.71073) |
| Theta (mix) | 2.7860 |
| Theta (max) | 28.8920 |
| h, k, lmax | 17, 16, 23 |
| R (reflections) | 0.0466 (3147) |
| wR2 (reflections) | 0.1327 3566) |

## 11. Computational Details:

All the geometries under investigation have been fully optimized using the dispersion-corrected B3LYP-D3[38-43] functional, coupled with the def2-TZVPP[44,45] basis set. To account for the solvent effects, we employed the SMD solvation model[46] with toluene solvent medium. Subsequently, we determined the vibrational frequencies of each stationary point using the same level of theory. This allowed us to classify the stationary points as either real minima (with no imaginary frequencies) or transition states (with only one imaginary frequency). The calculated triplet-singlet energy gap represents the energy difference between the optimized triplet and singlet states, respectively. All the calculations were executed using Gaussian 16.[47]

| Compound | Structure | Singlet State $E_S$ (Ha) | Triplet State $E_T$ (Ha) | $\Delta E_T$ ($E_T$-$E_S$) (kcal/mol) |
|---|---|---|---|---|
| **2k** | 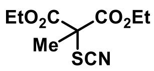 | -1104.98296 | -1104.91149 | 44.9 |
| **5f** | 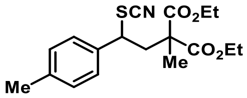 | -1454.14445 | -1454.07498 | 43.6 |
| ***fac*-Ir(ppy)$_3$** | 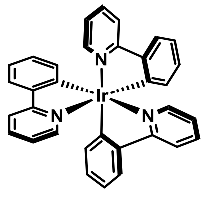 | -1541.37696 | -1541.28472 | 57.9 (58.1)[a] |
| **PTH2** | 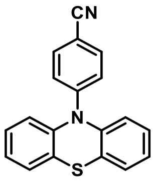 | -1239.32088 | -1239.22831 | 58.1 |
| **PTH4** | 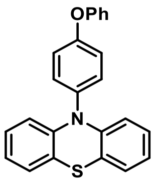 | -1453.43940 | -1453.34631 | 58.4 |

**Supplementary Table 9:** The calculated singlet, triplet state energies (Hartree), and singlet-triplet energy gap in kcal/mol.

[a]This is the reported energy value of *fac*-Ir(ppy)$_3$, which is matched with our computationally calculated value. For reference, see: Bellotti, P. *et al.* Visible-Light Photocatalyzed peri-(3+2) Cycloadditions of Quinolines. *J. Am. Chem. Soc.* **144**, 15662-15671 (2022)

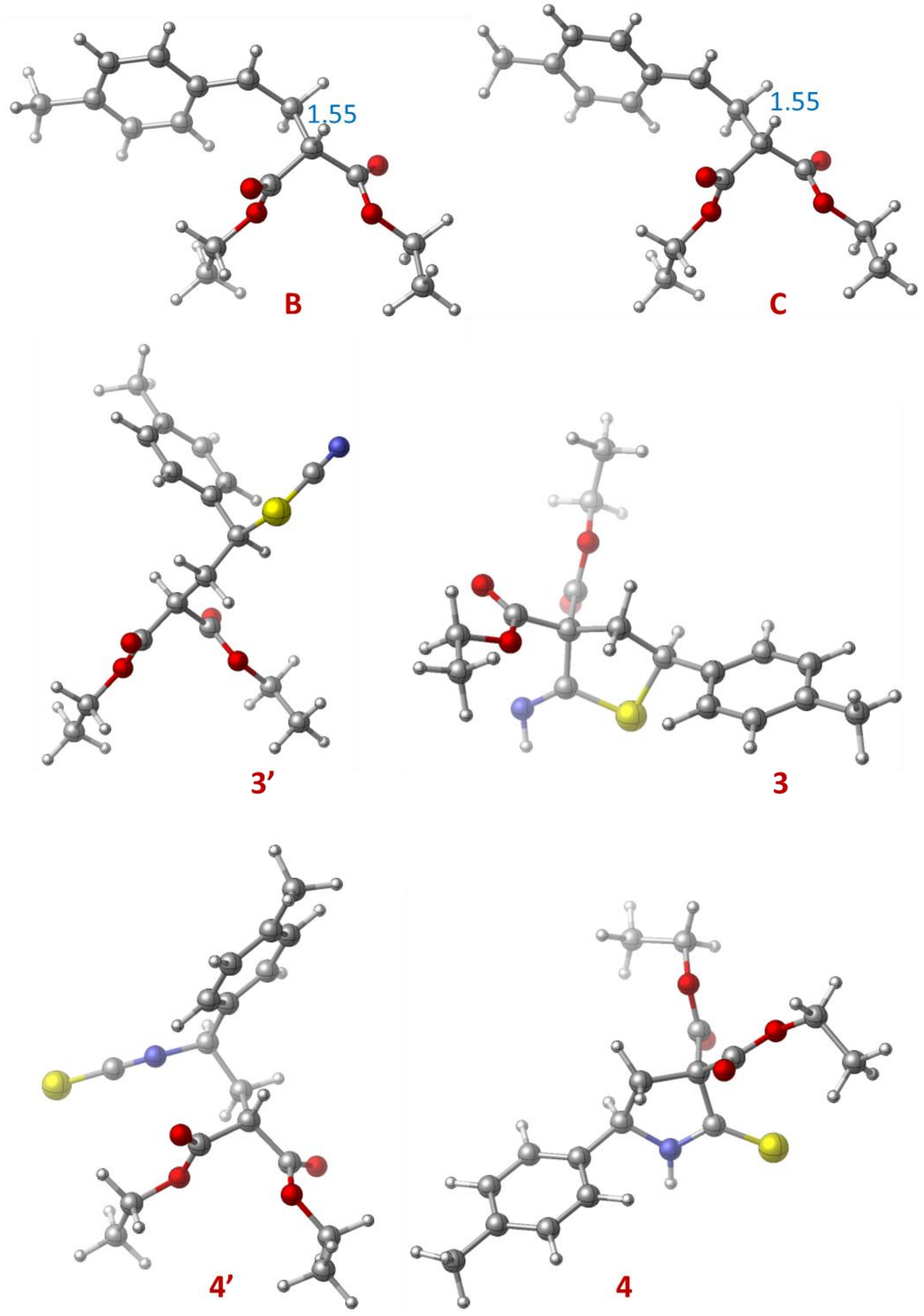


**Supplementary Fig. 33:** The optimized geometries of various intermediates and products (starting from 4-methylstyrene) mentioned in Fig. 8. The bond distances are in Å. [C: grey, H: white, S: yellow, N: blue, O: red]

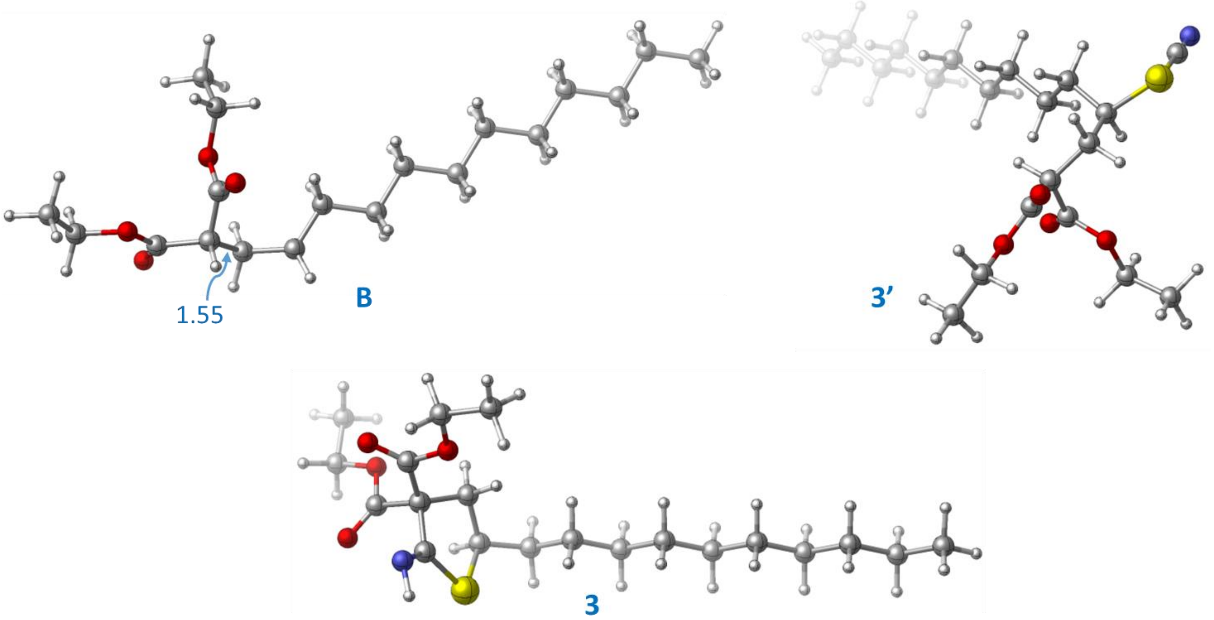


**Supplementary Fig. 34:** The optimized geometries of various intermediates and products (starting from 1-dodecene) mentioned in Fig. 8. The bond distances are in Å. [C: grey, H: white, S: yellow, N: blue, O: red]

## 12. NMR Spectra:

$^1$H NMR of **PTH1** (400 MHz, $CDCl_3$):

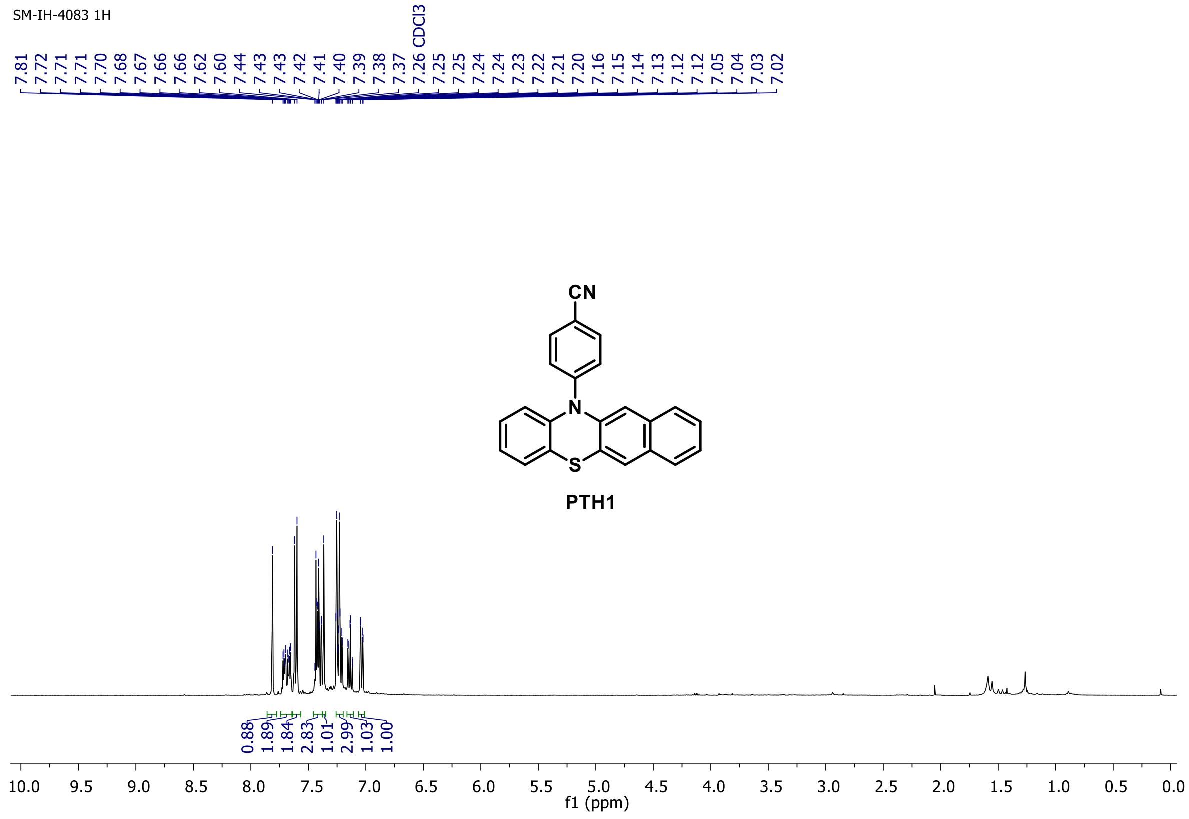


$^{13}C\{^1H\}$ NMR of **PTH1** (101 MHz, $CDCl_3$):

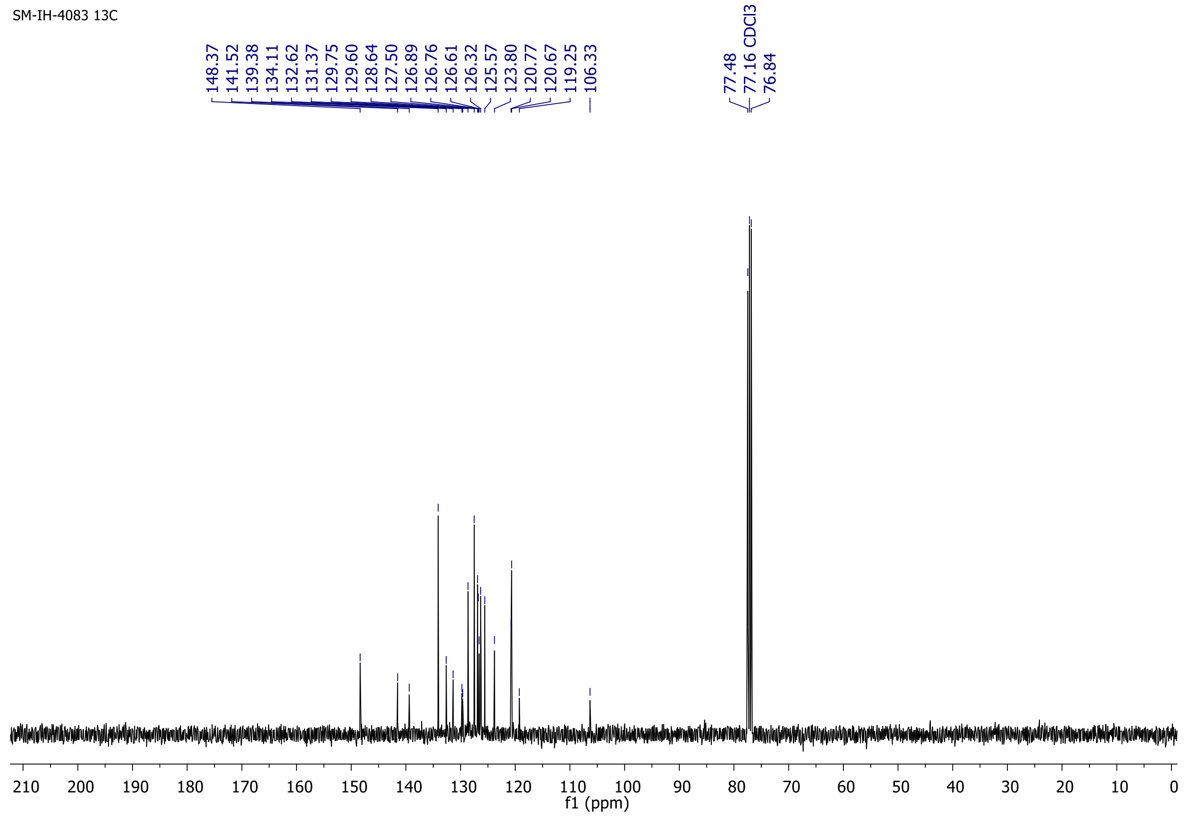

$^{1}H$ NMR of **PTH2** (400 MHz, DMSO-d$_6$):

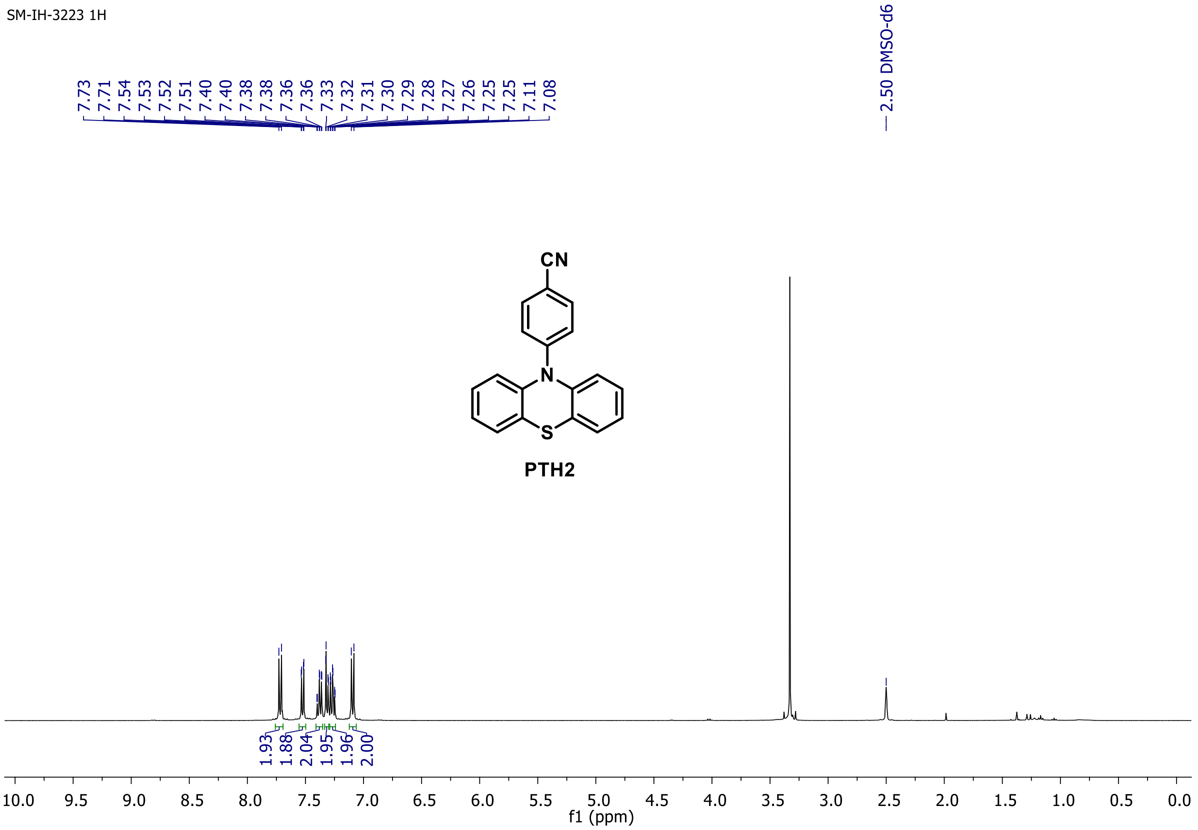


$^{13}C\{^{1}H\}$ NMR of **PTH2** (101 MHz, DMSO-d$_6$):

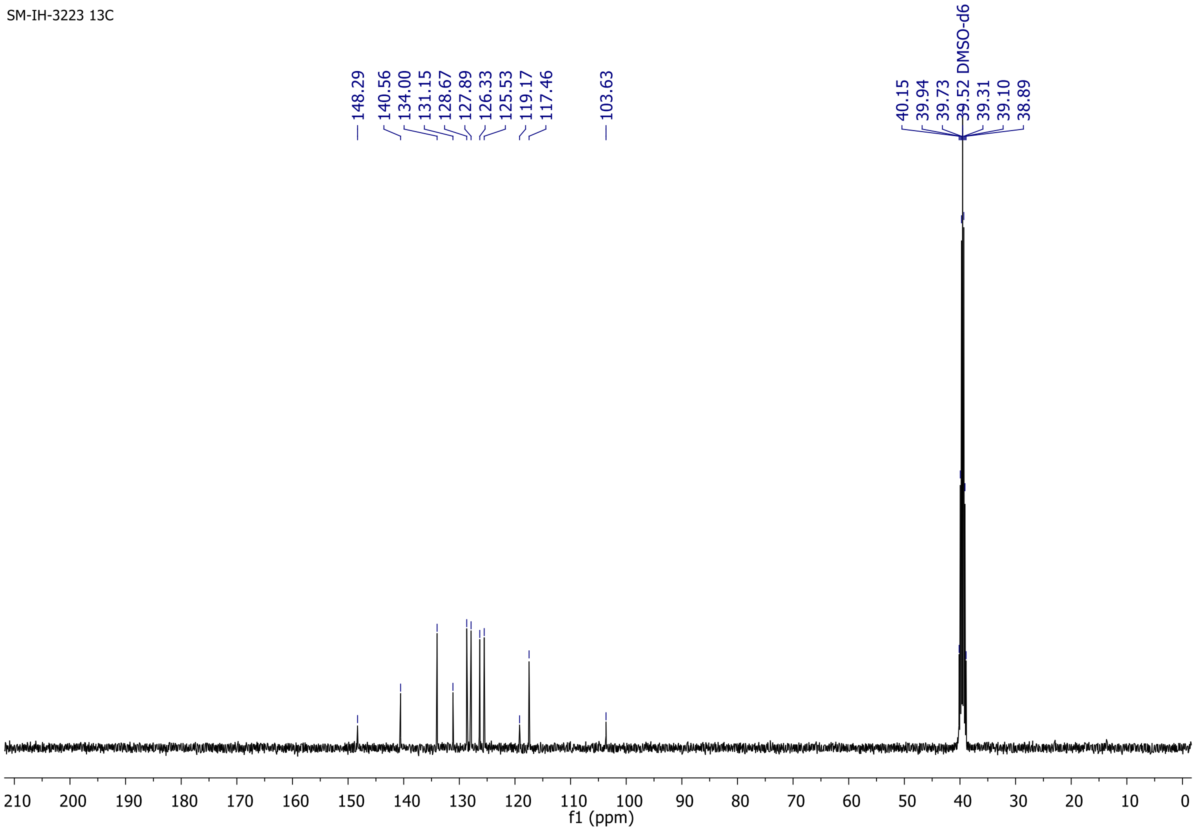

$^{1}H$ NMR of **PTH3** (400 MHz, DMSO-d$_6$):

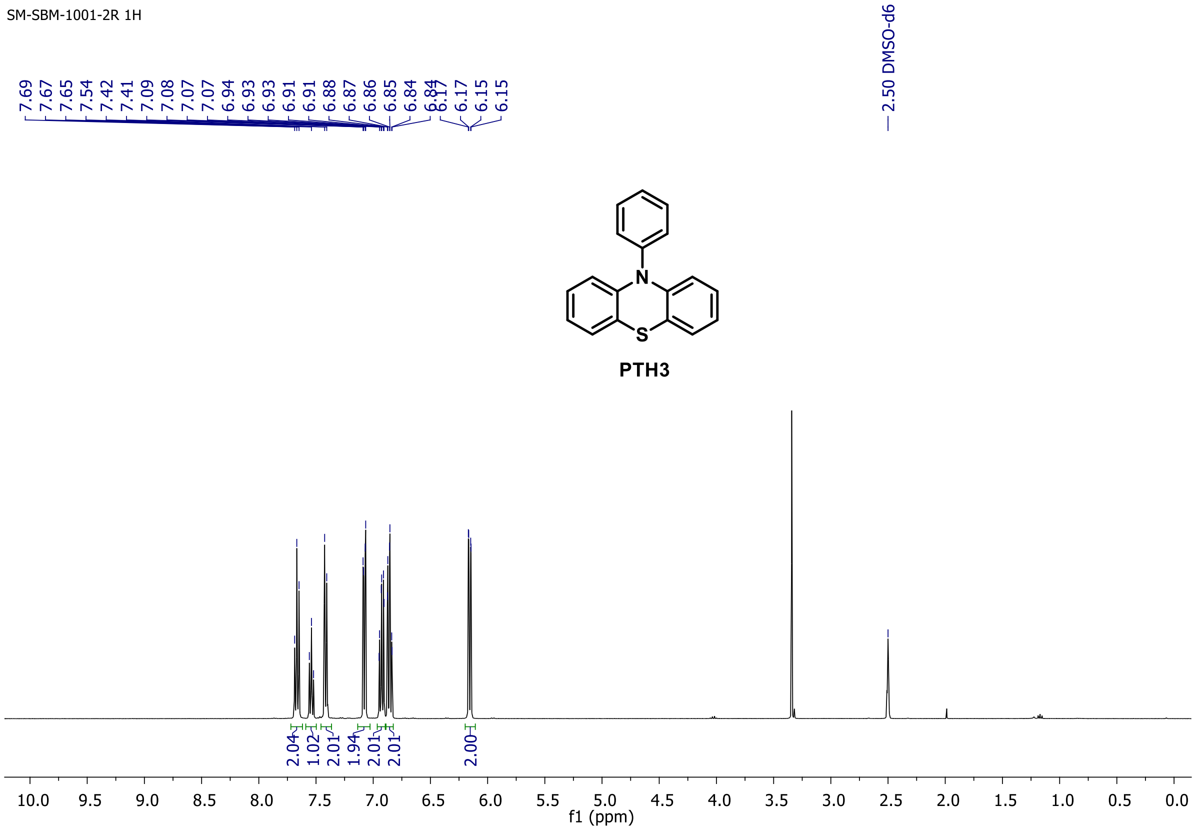


$^{13}C\{^{1}H\}$ NMR of **PTH3** (101 MHz, DMSO-d$_6$):

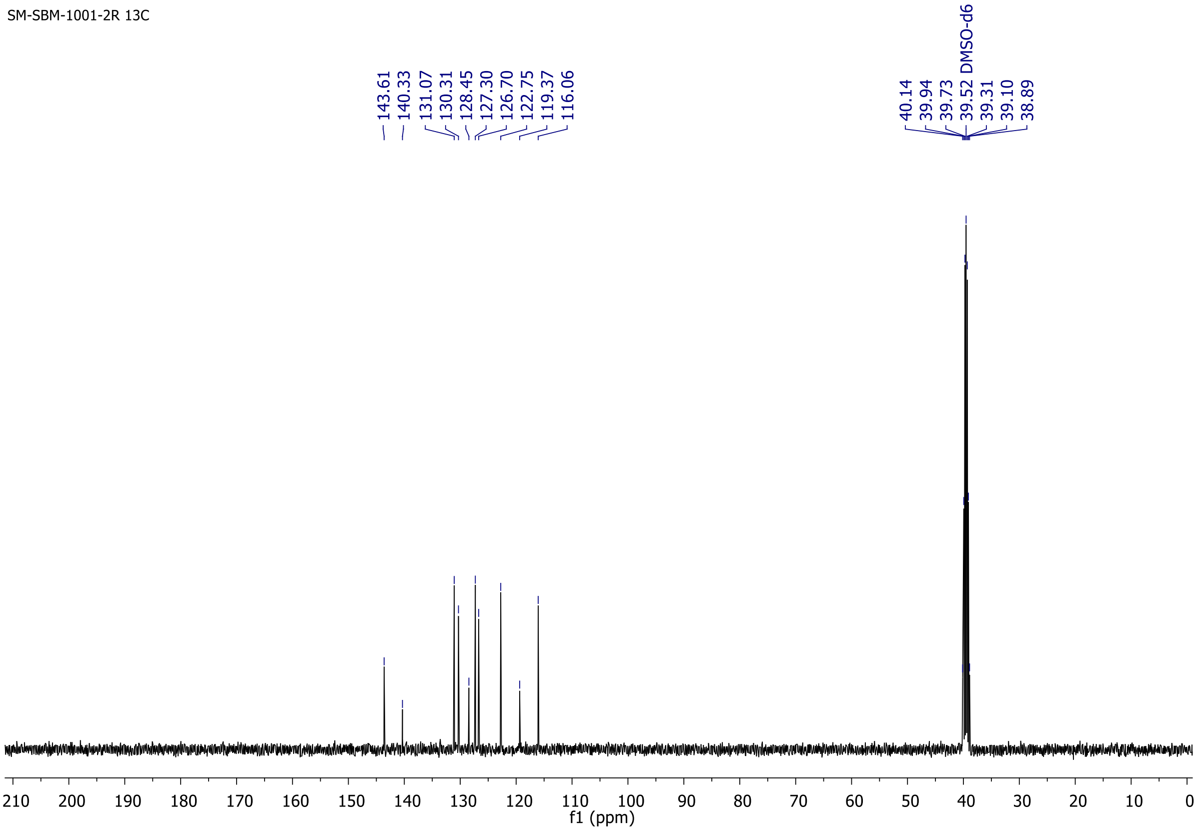

$^{1}H$ NMR of **PTH4** (400 MHz, DMSO-d$_6$):

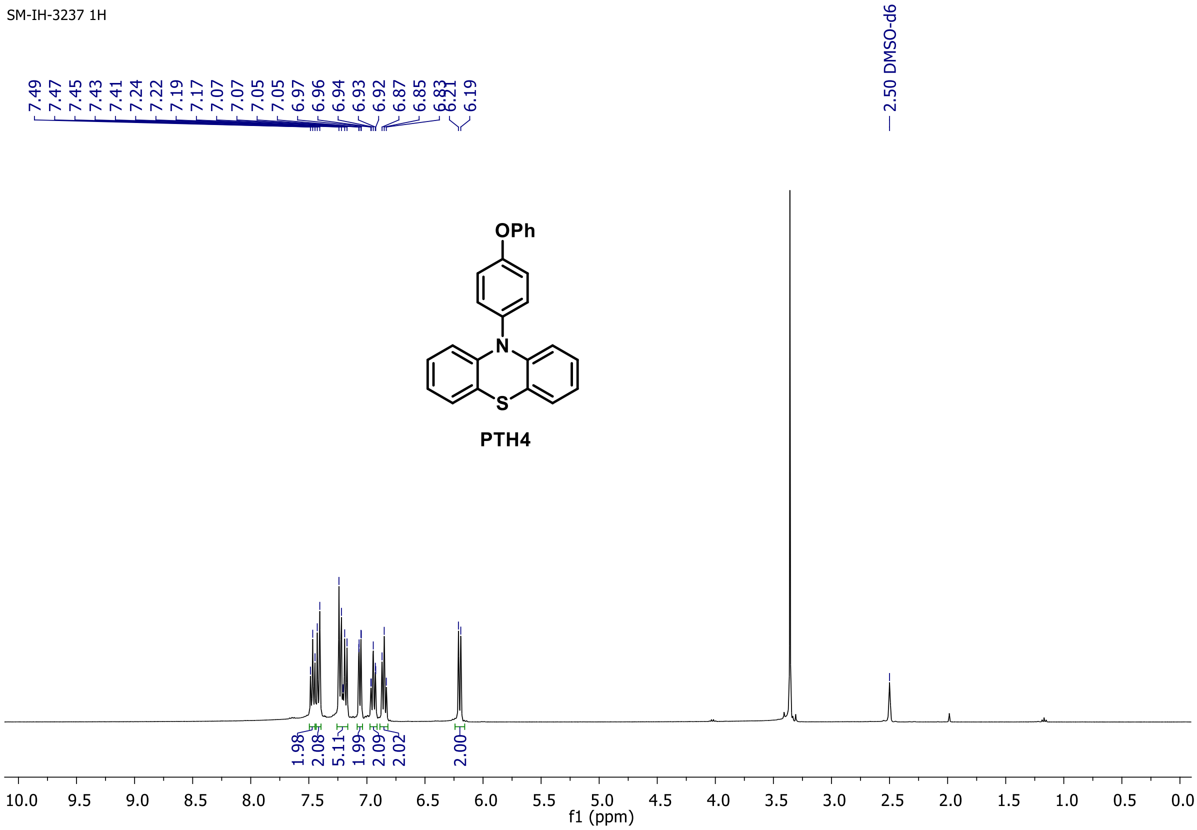


$^{13}C\{^{1}H\}$ NMR of **PTH4** (101 MHz, DMSO-d$_6$):

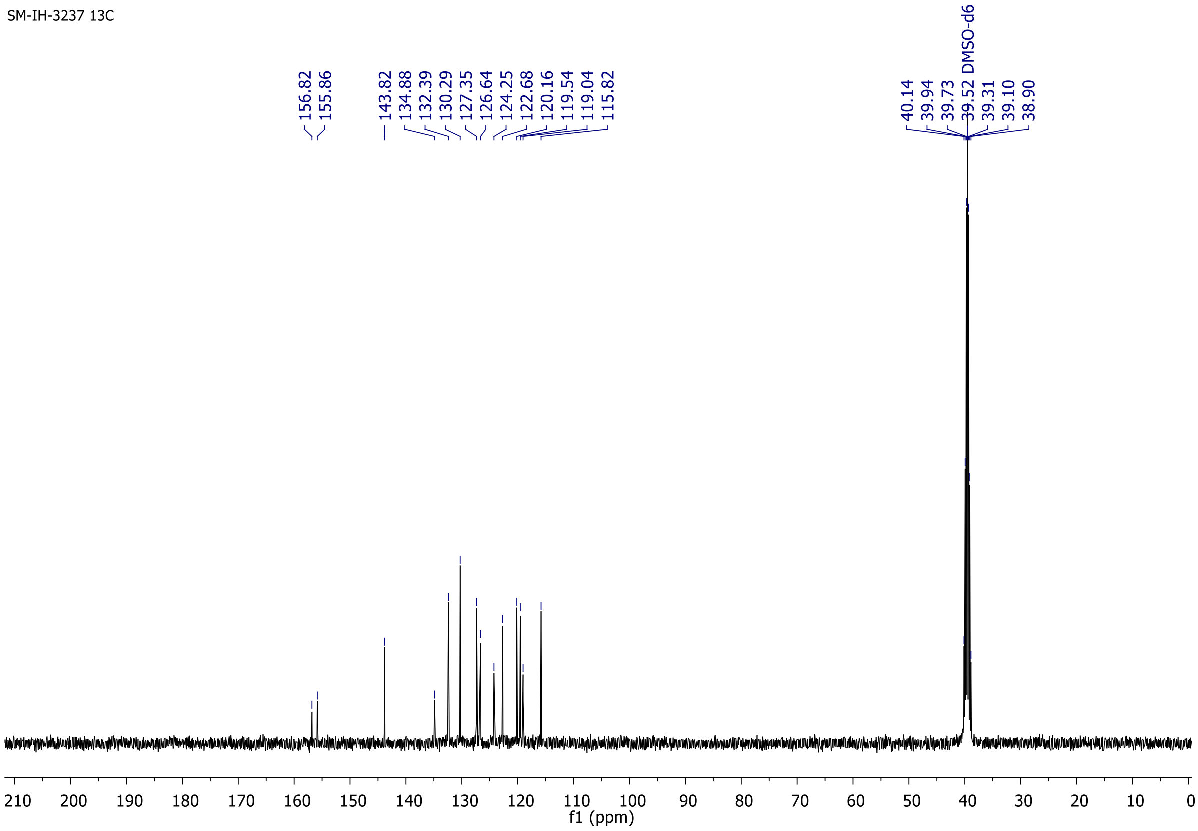

$^{1}H$ NMR of **1u** (400 MHz, $CDCl_3$):

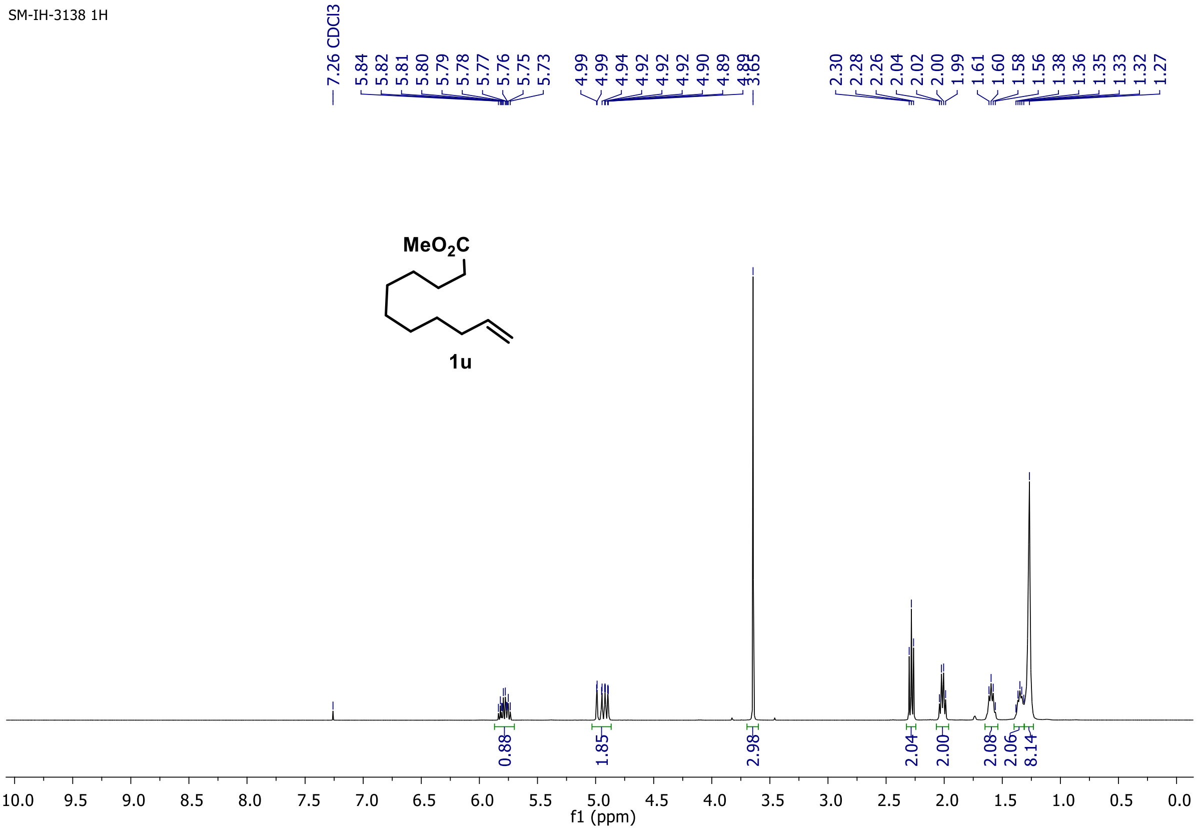


$^{13}C\{^{1}H\}$ NMR of **1u** (101 MHz, $CDCl_3$):

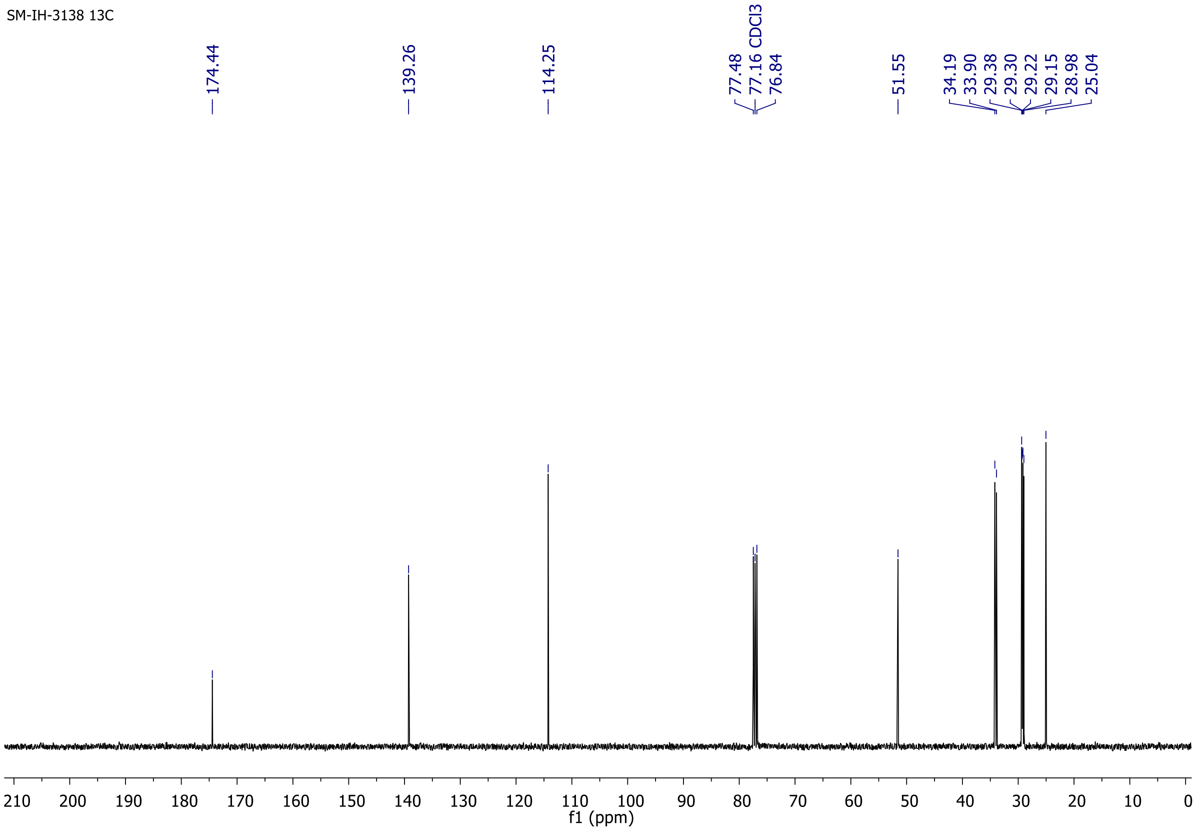

$^{1}$H NMR of **1y** (400 MHz, $CDCl_3$):

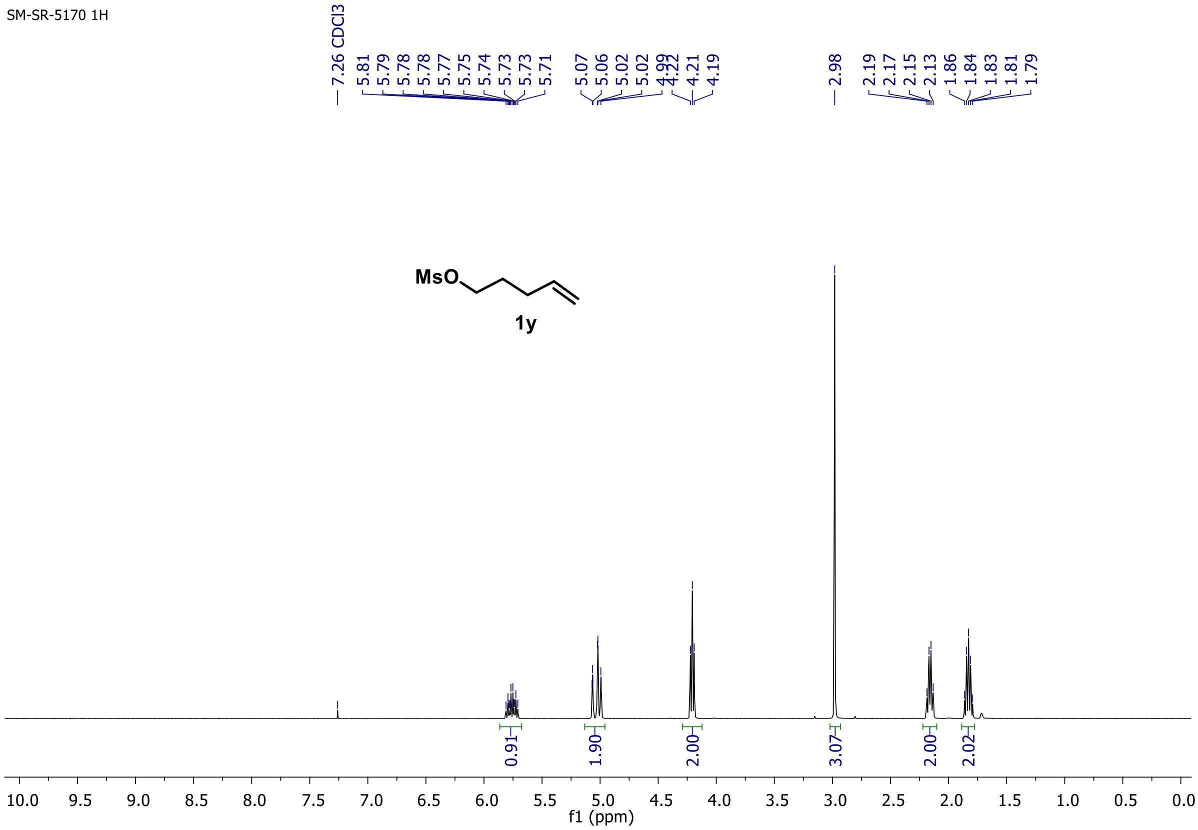


$^{13}$C{$^{1}$H} NMR of **1y** (101 MHz, $CDCl_3$):

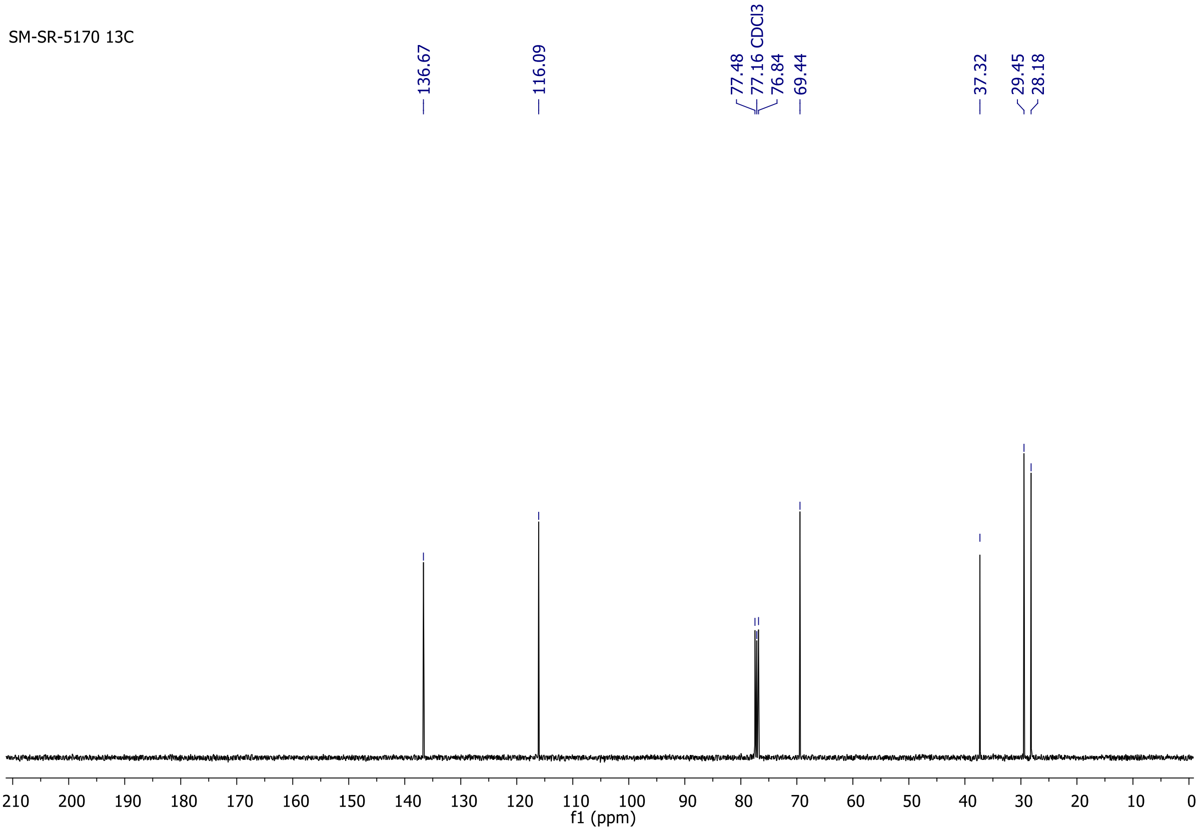

$^{1}$H NMR of **1ab** (400 MHz, $CDCl_3$):

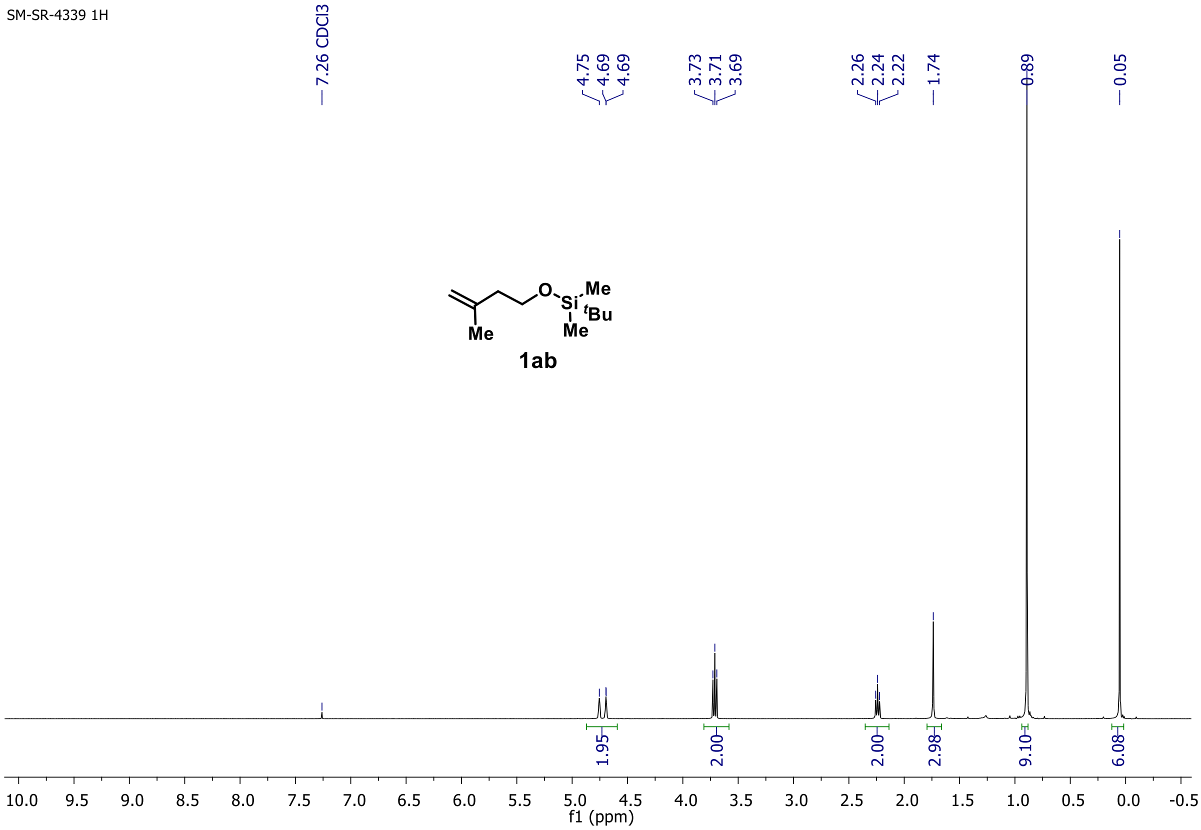


$^{13}$C{$^{1}$H} NMR of **1ab** (101 MHz, $CDCl_3$):

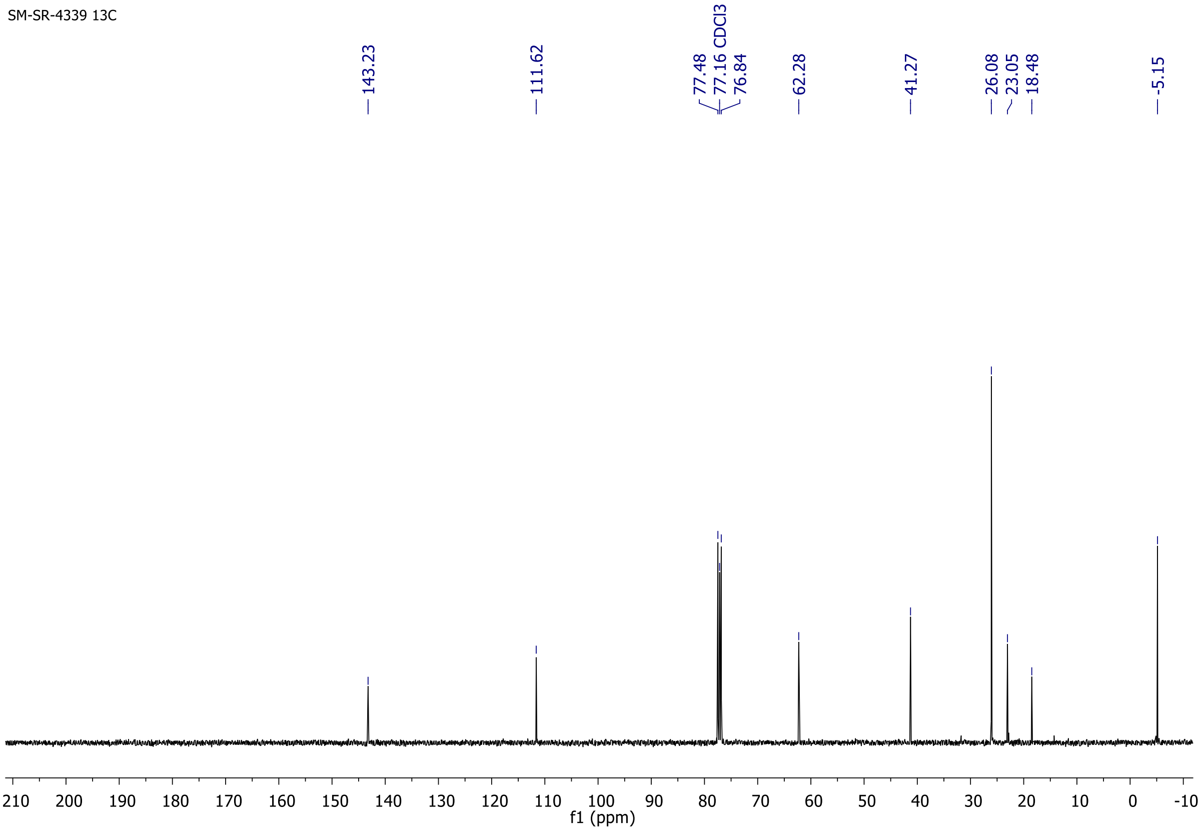

<sup></sup>
$^{1}$H NMR of **1am** (400 MHz, $CDCl_3$):

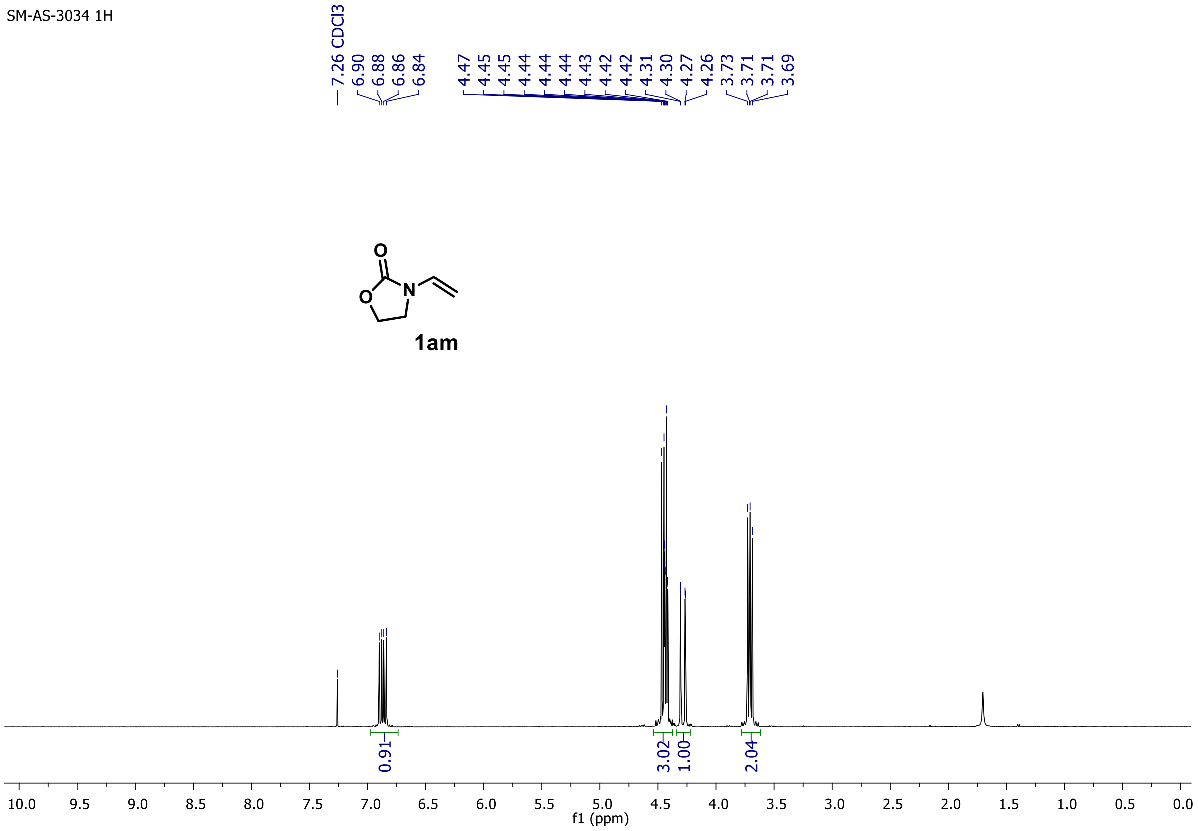


$^{13}$C{$^{1}$H} NMR of **1am** (101 MHz, $CDCl_3$):

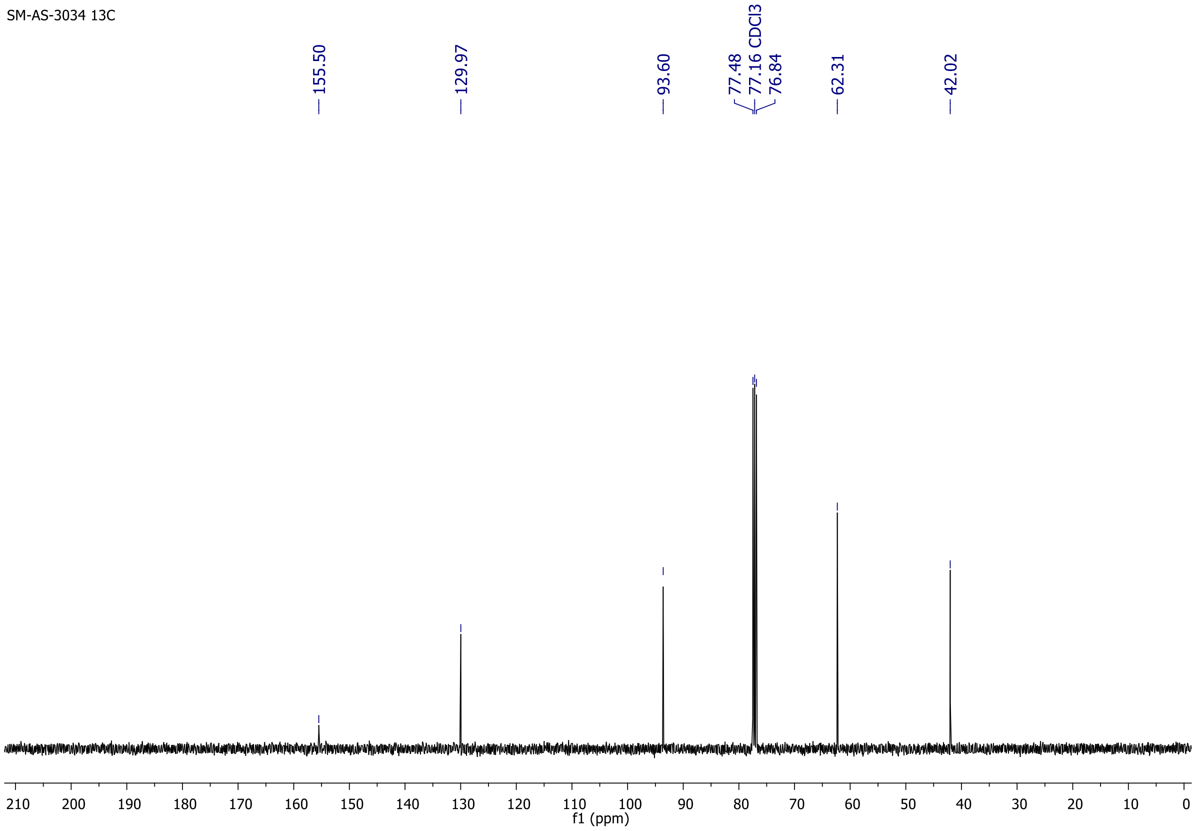

$^{1}H$ NMR of **1aq** (400 MHz, $CDCl_3$):

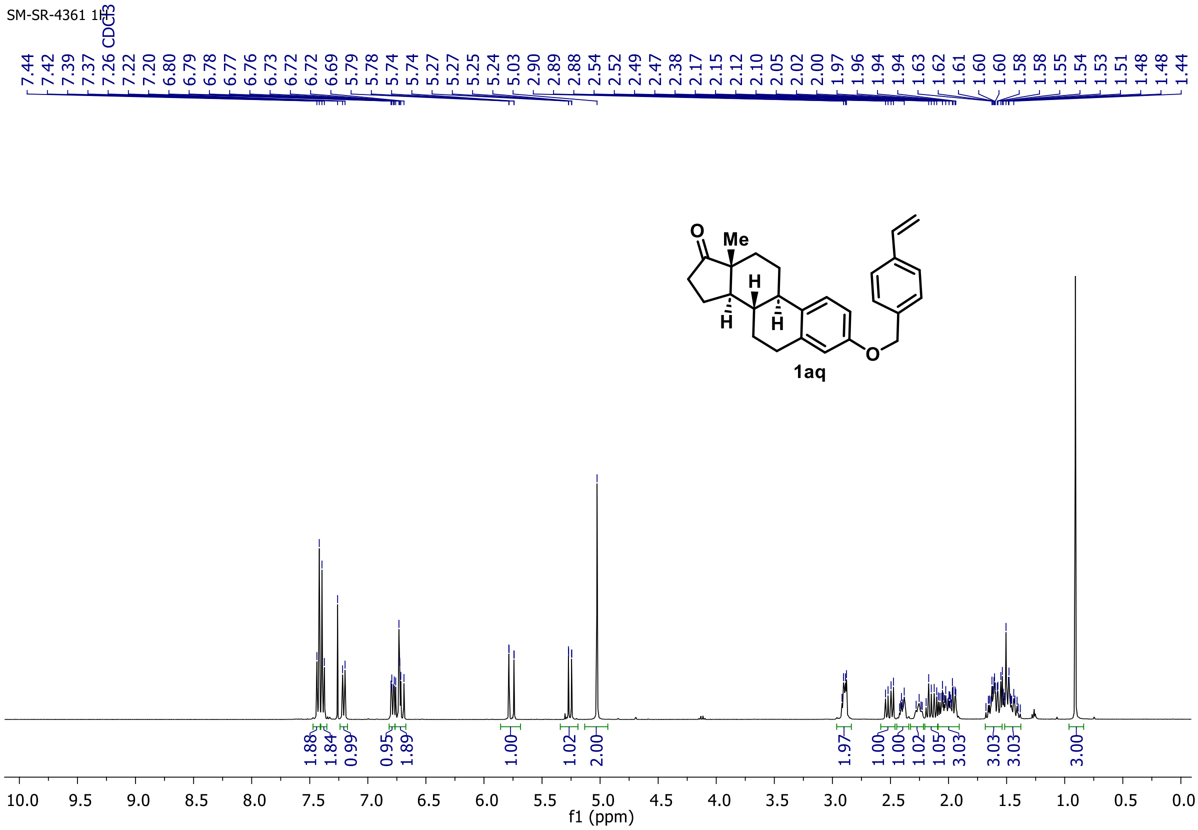


$^{13}C\{^{1}H\}$ NMR of **1aq** (101 MHz, $CDCl_3$):

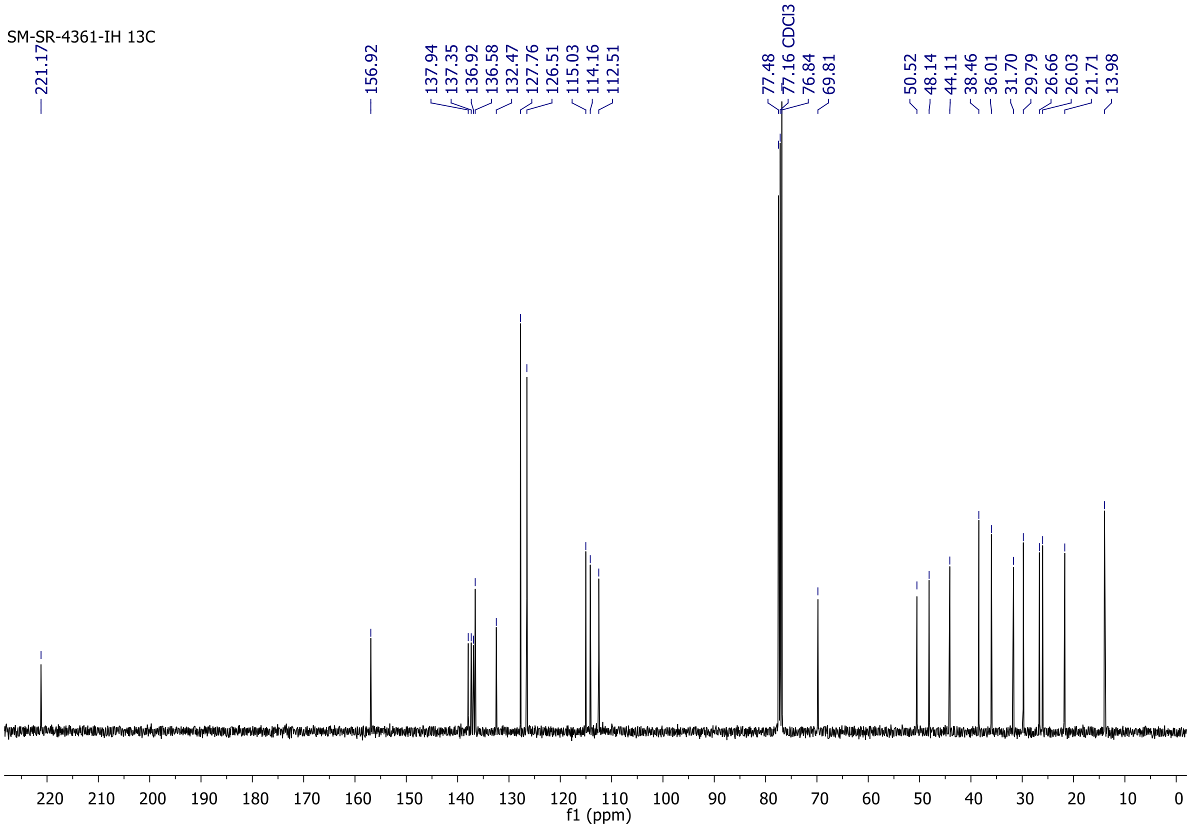

$^{1}$H NMR of **1ar** (400 MHz, $CDCl_3$):

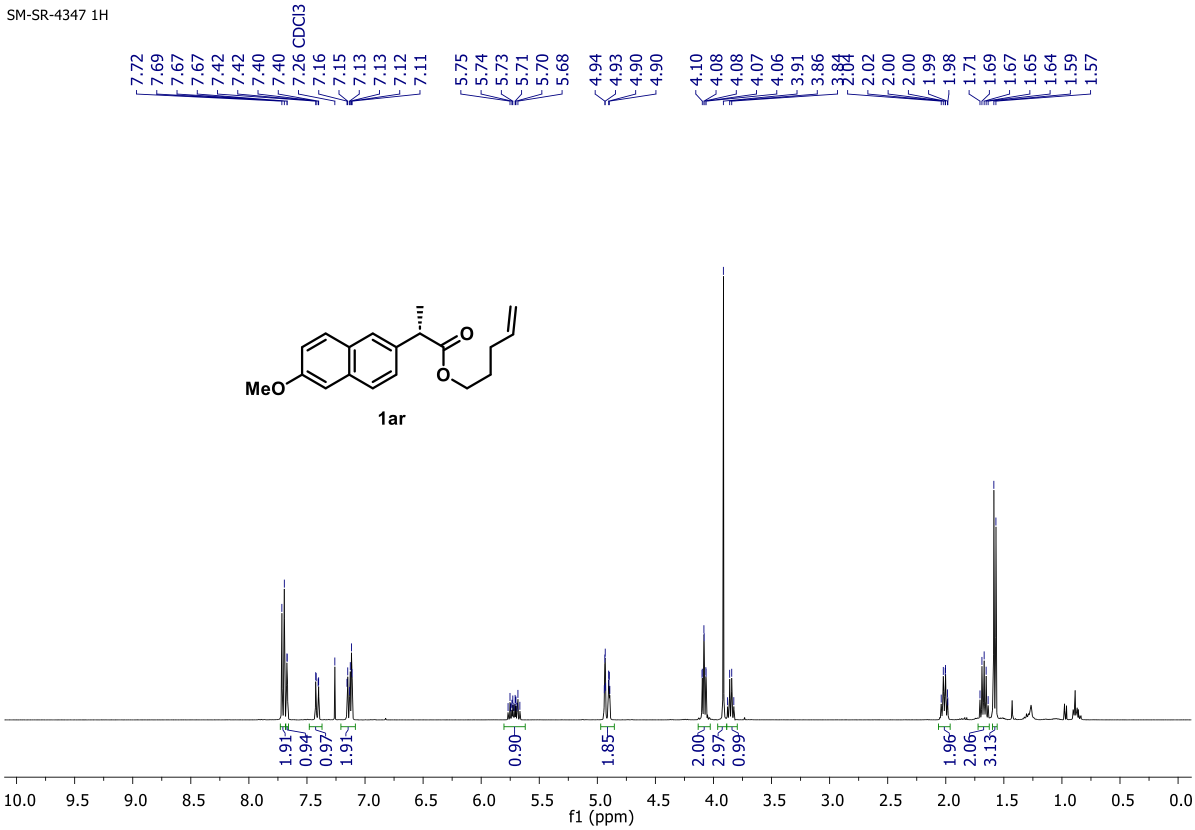


$^{13}$C{$^{1}$H} NMR of **1ar** (101 MHz, $CDCl_3$):

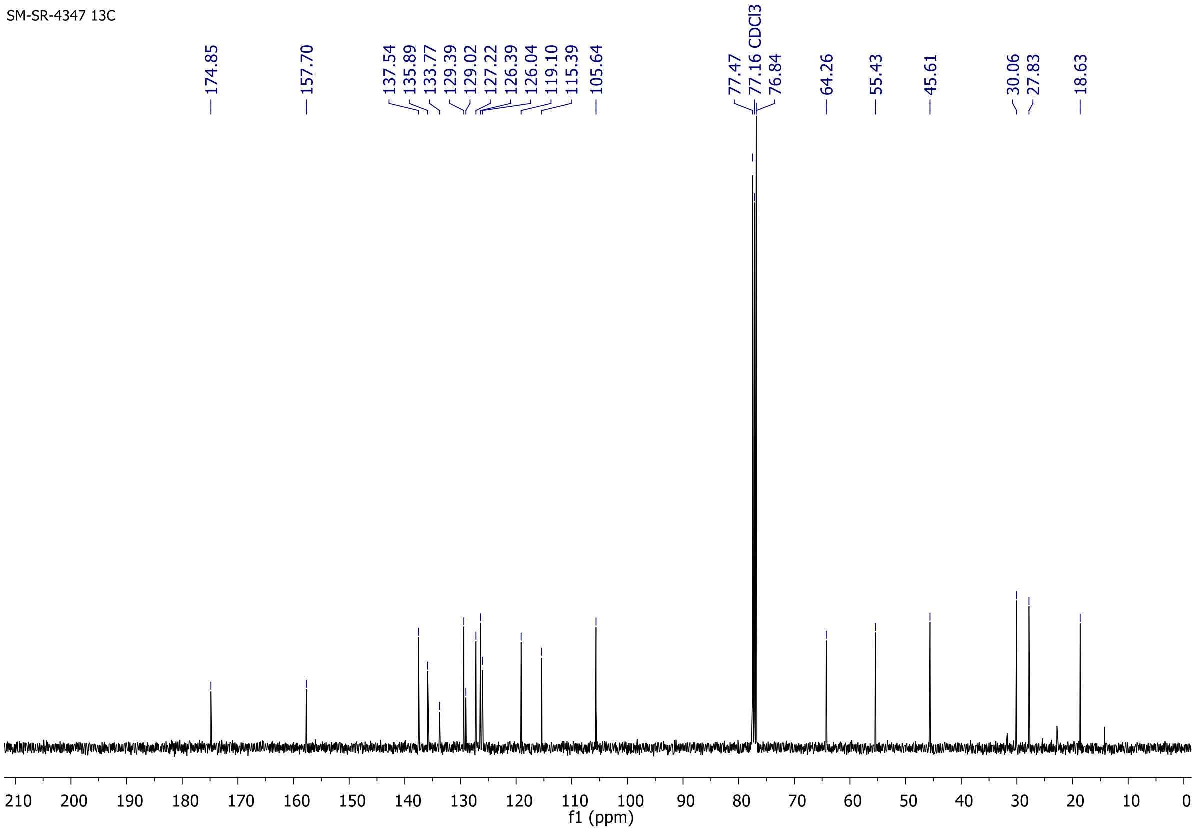

$^1$H NMR of **1as** (400 MHz, $CDCl_3$):

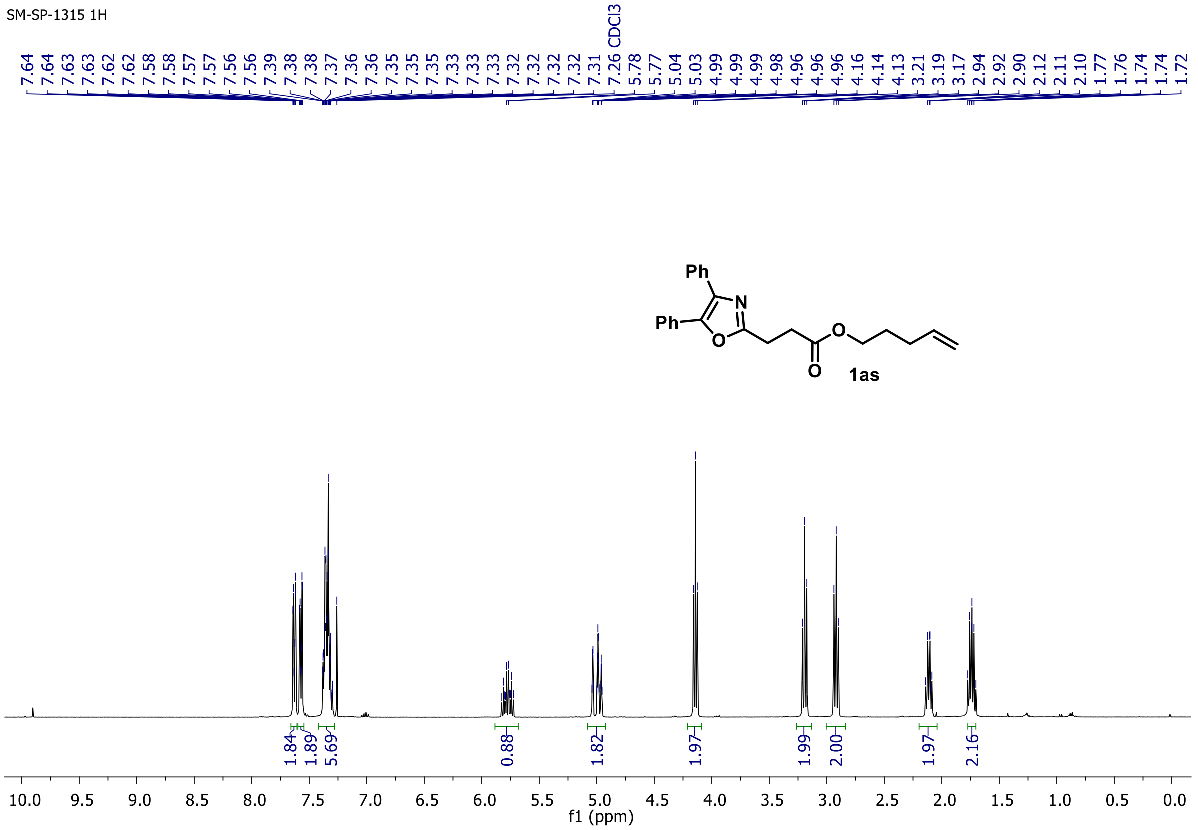


$^{13}$C{$^1$H} NMR of **1as** (101 MHz, $CDCl_3$):

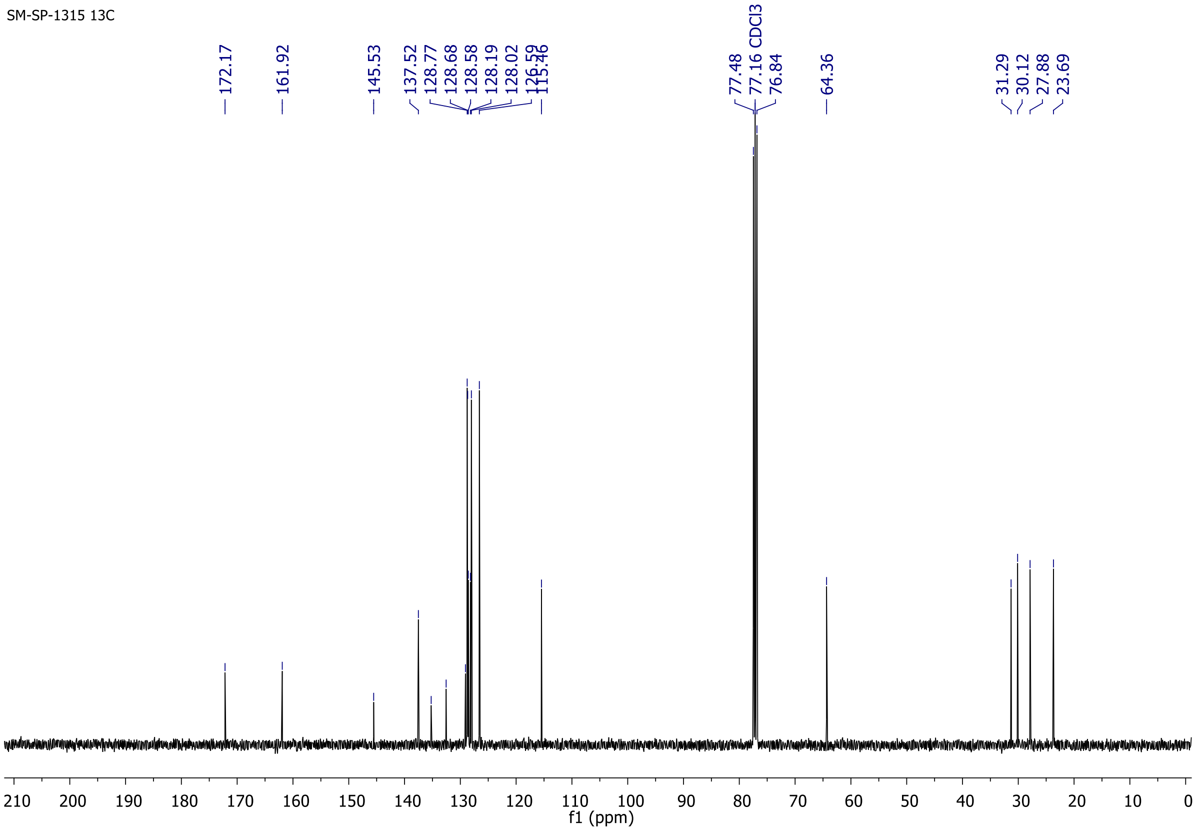

$^{1}$H NMR of **1at** (400 MHz, $CDCl_3$):

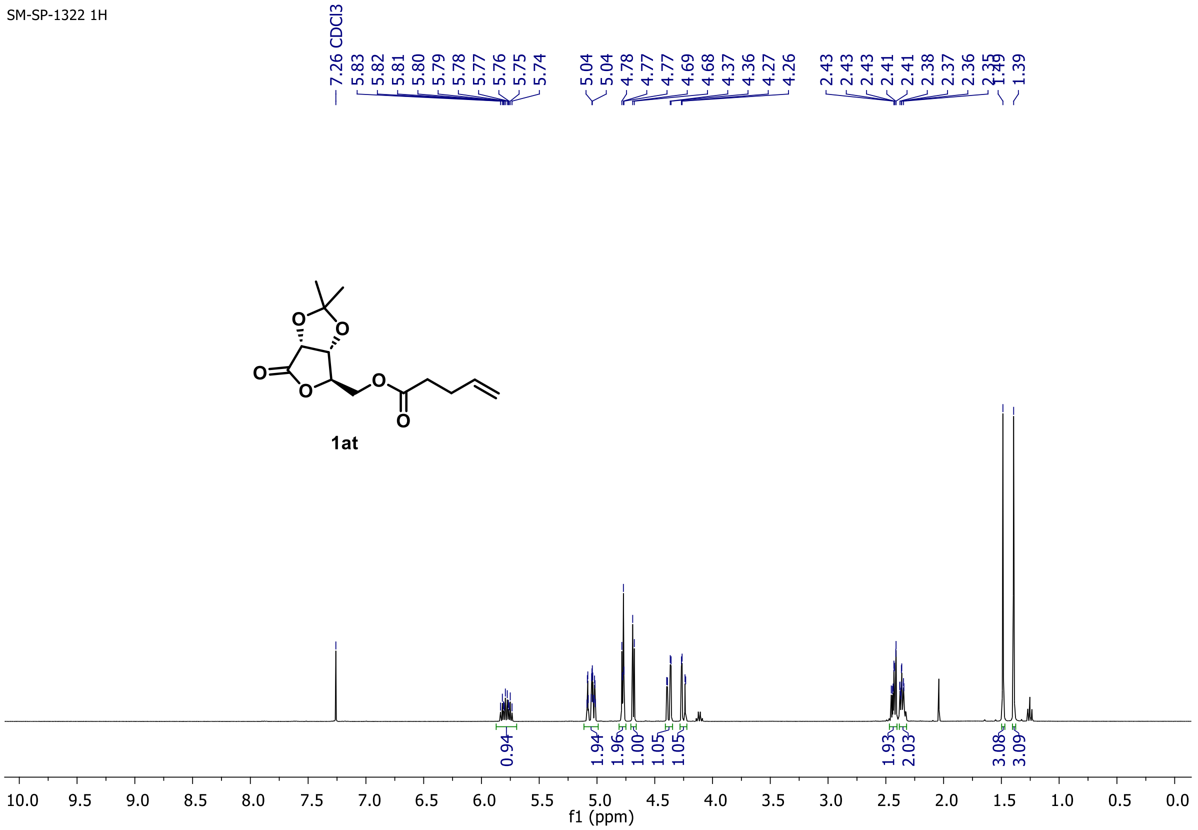


$^{13}$C{$^{1}$H} NMR of **1at** (101 MHz, $CDCl_3$):

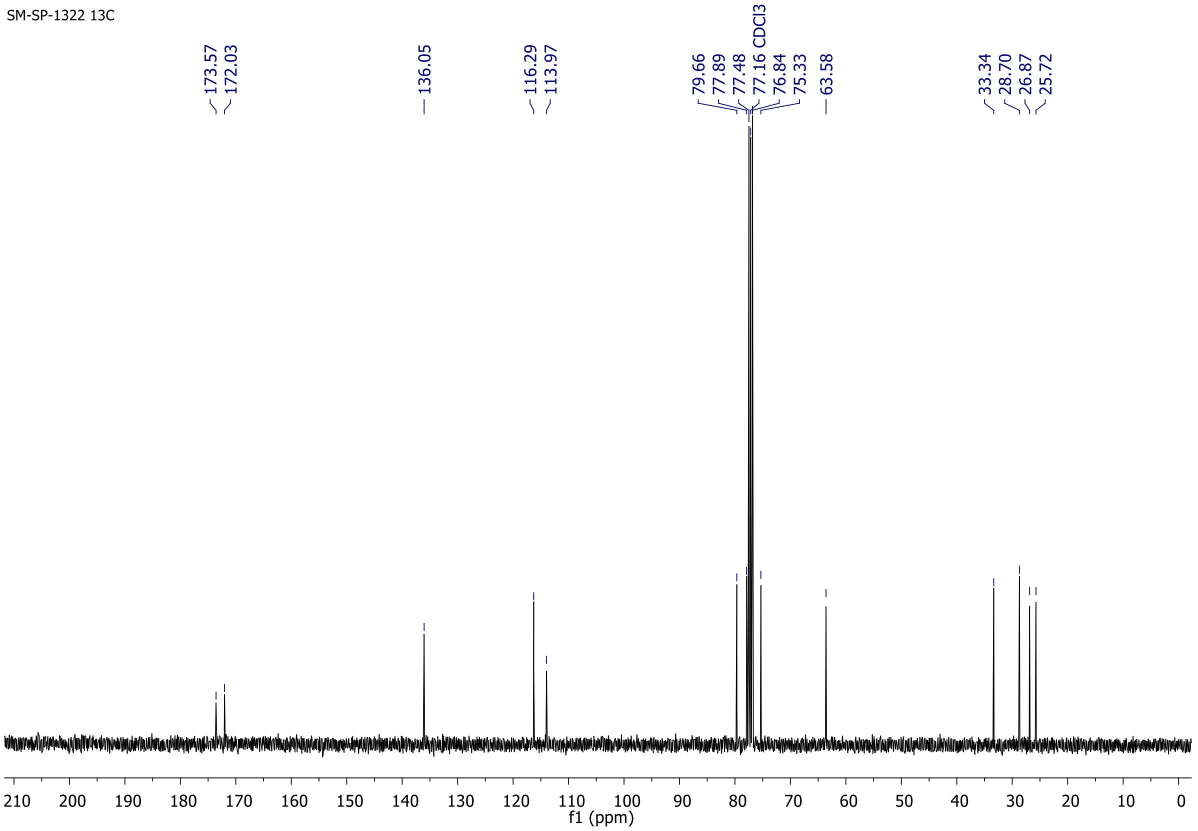

## $^1$H NMR of **1au** (400 MHz, $CDCl_3$):

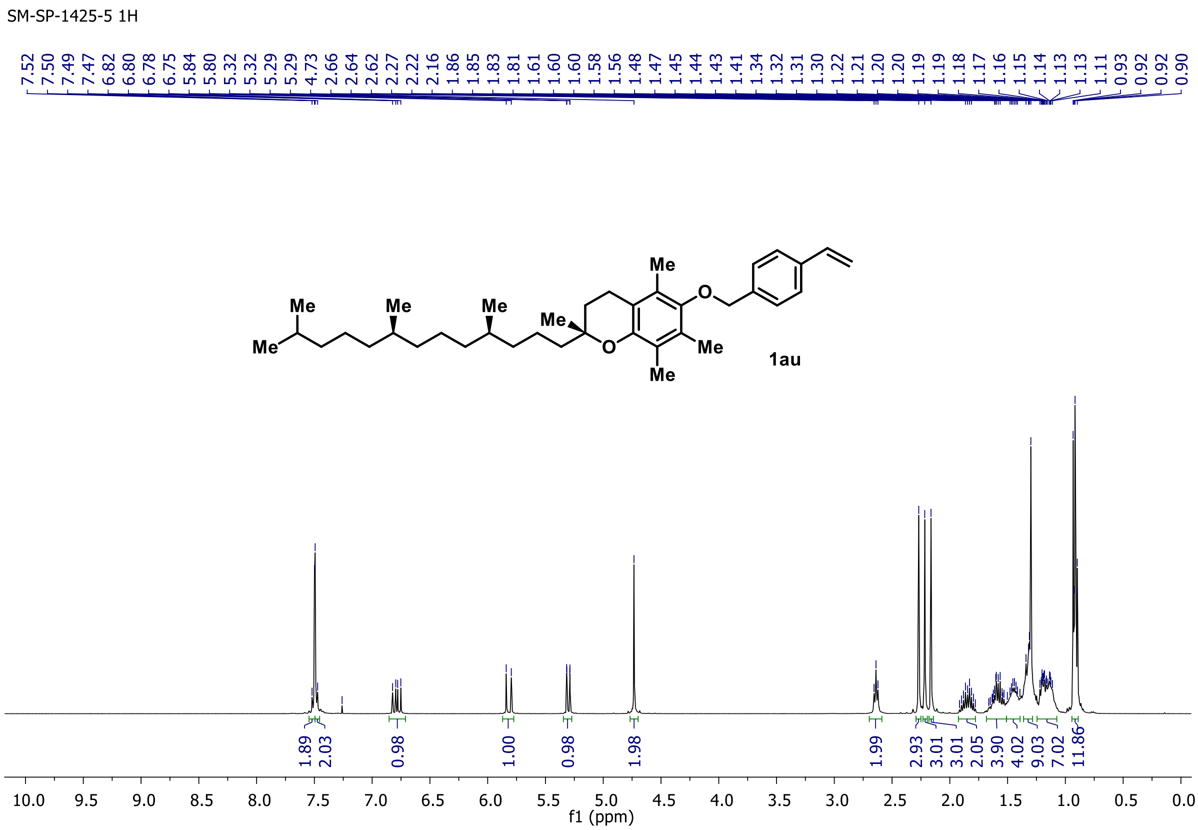


## $^{13}$C{$^1$H} NMR of **1au** (101 MHz, $CDCl_3$):

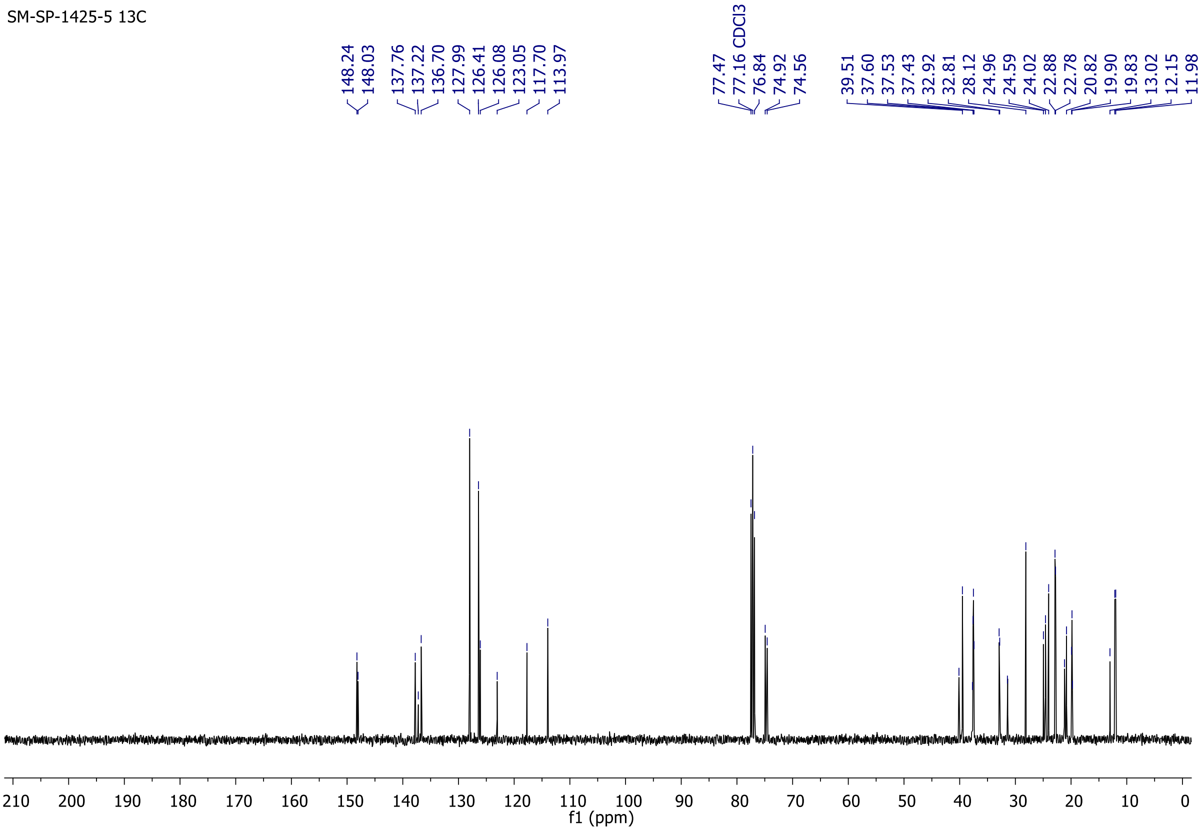

$^{1}$H NMR of **1av** (400 MHz, $CDCl_3$):

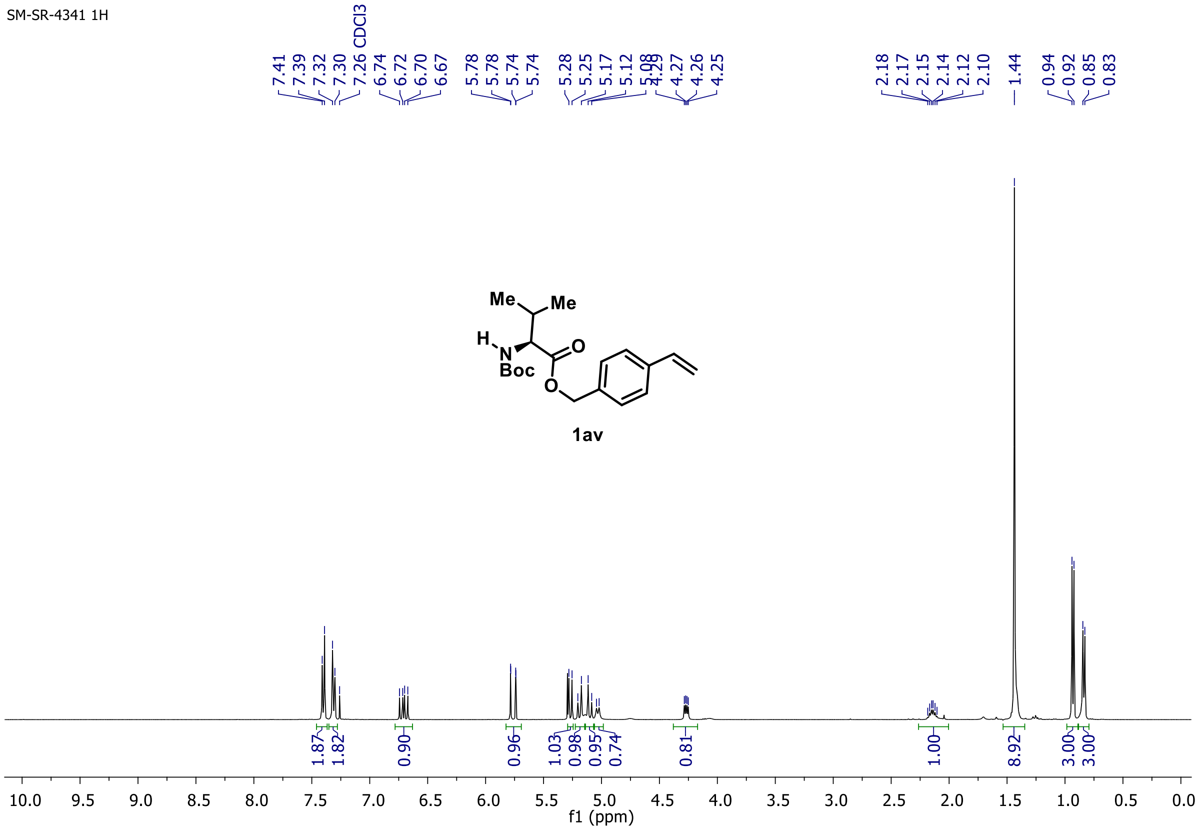


$^{13}$C{$^{1}$H} NMR of **1av** (101 MHz, $CDCl_3$):

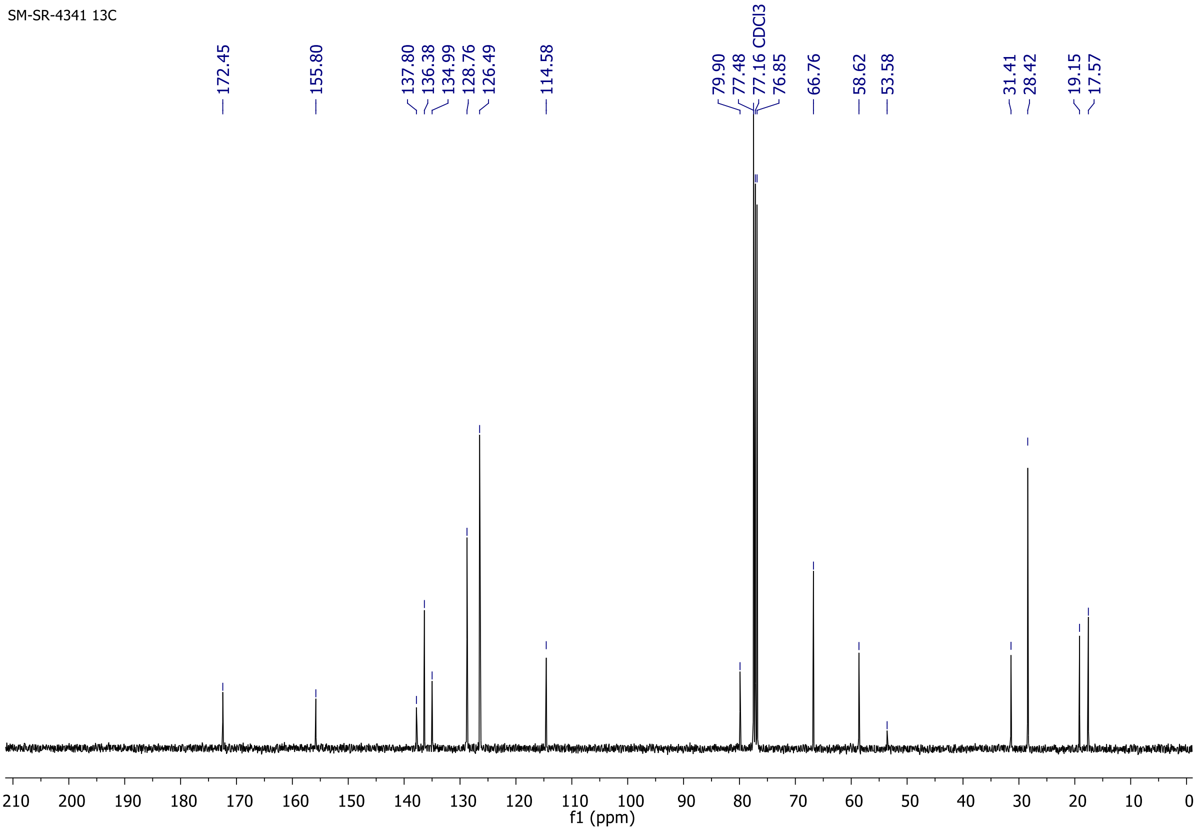

$^{1}$H NMR of **1aw** (400 MHz, $CDCl_3$):

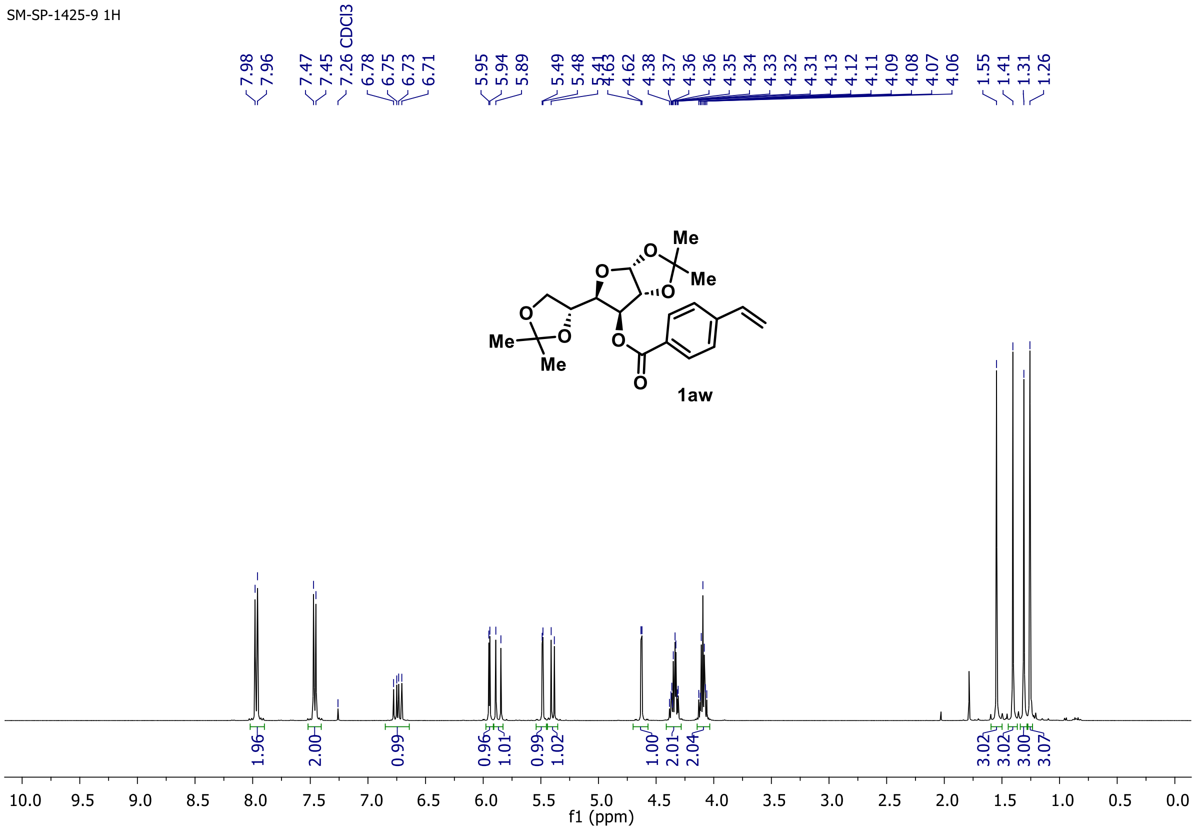


$^{13}C\{^{1}H\}$ NMR of **1aw** (101 MHz, $CDCl_3$):

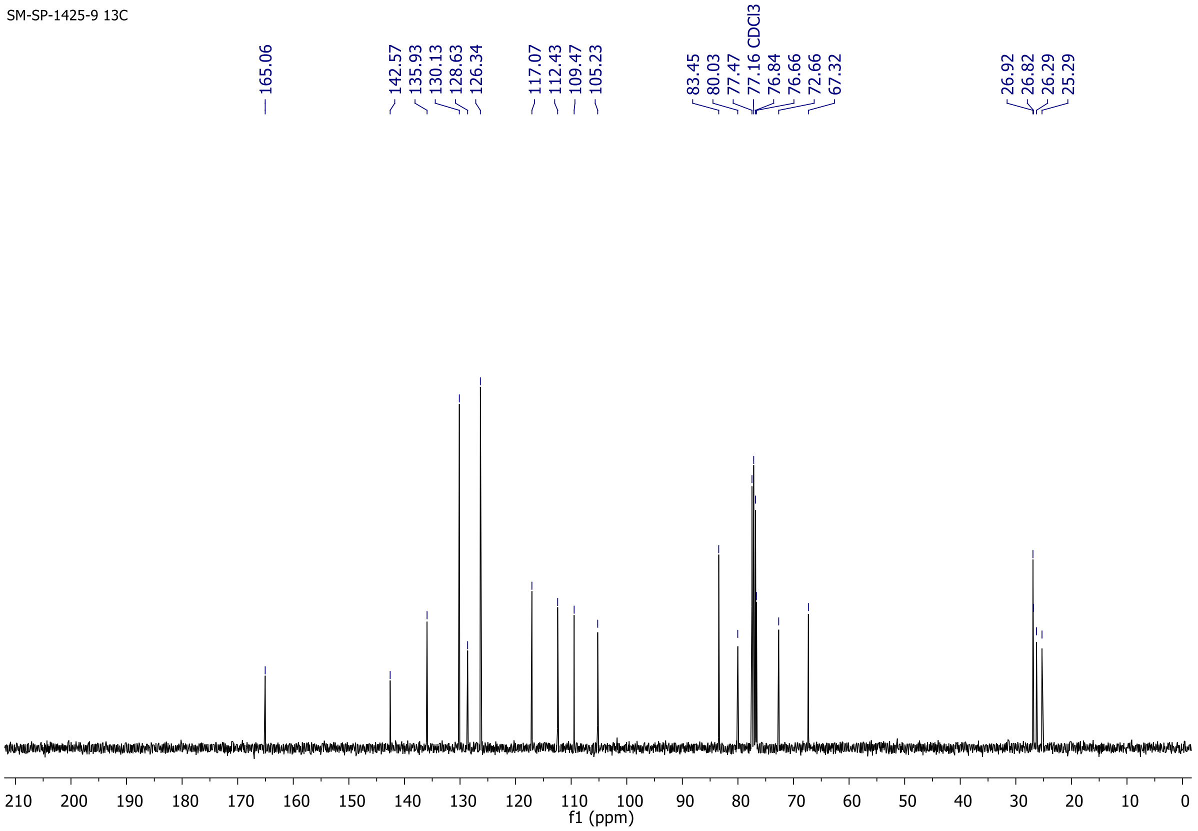

$^1$H NMR of **2a** (400 MHz, $CDCl_3$):

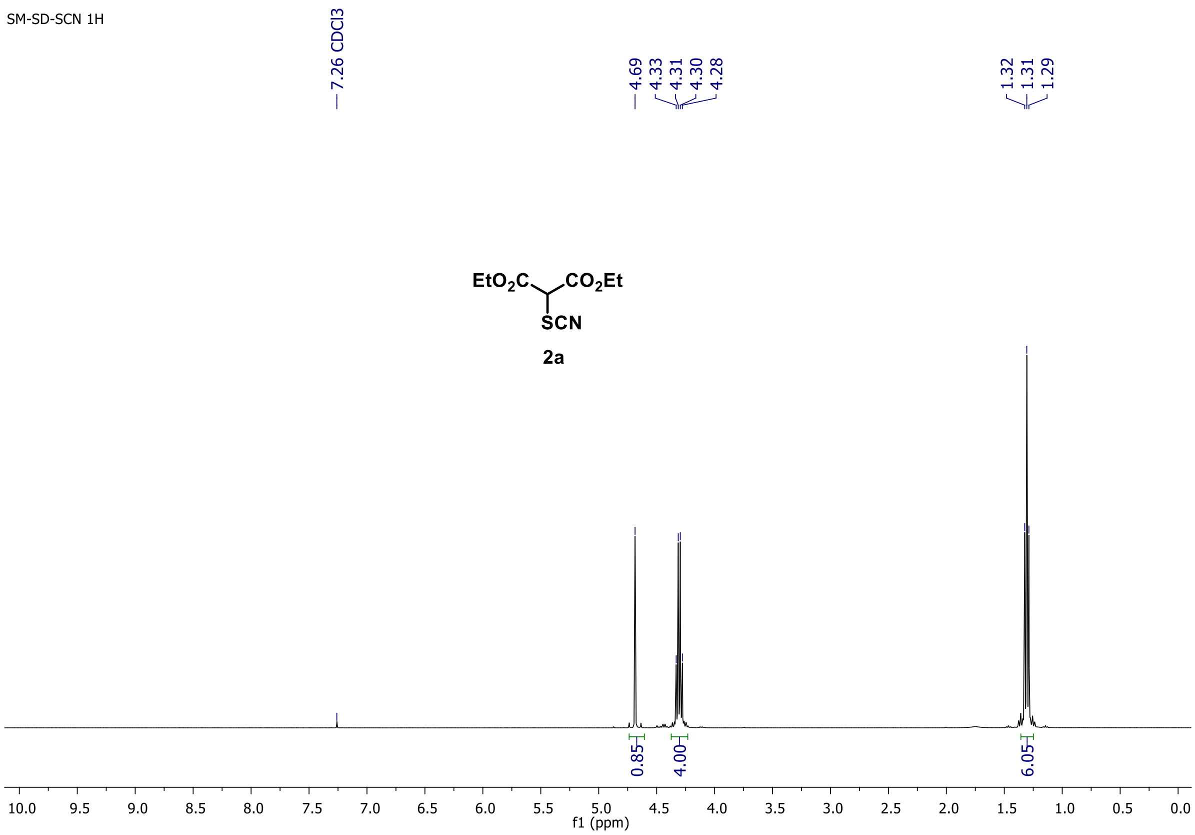


$^{13}C\{^1H\}$ NMR of **2a** (101 MHz, $CDCl_3$):

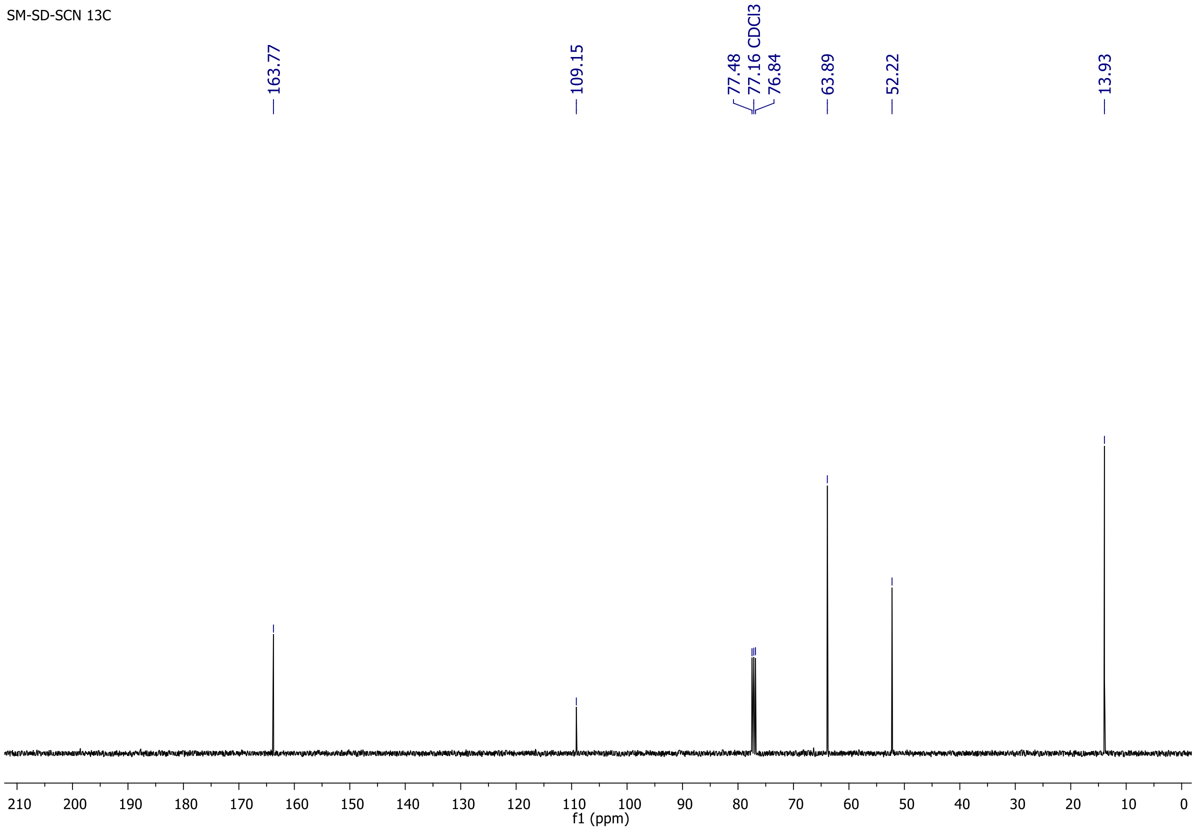

$^1$H NMR of **2b** (400 MHz, $CDCl_3$):

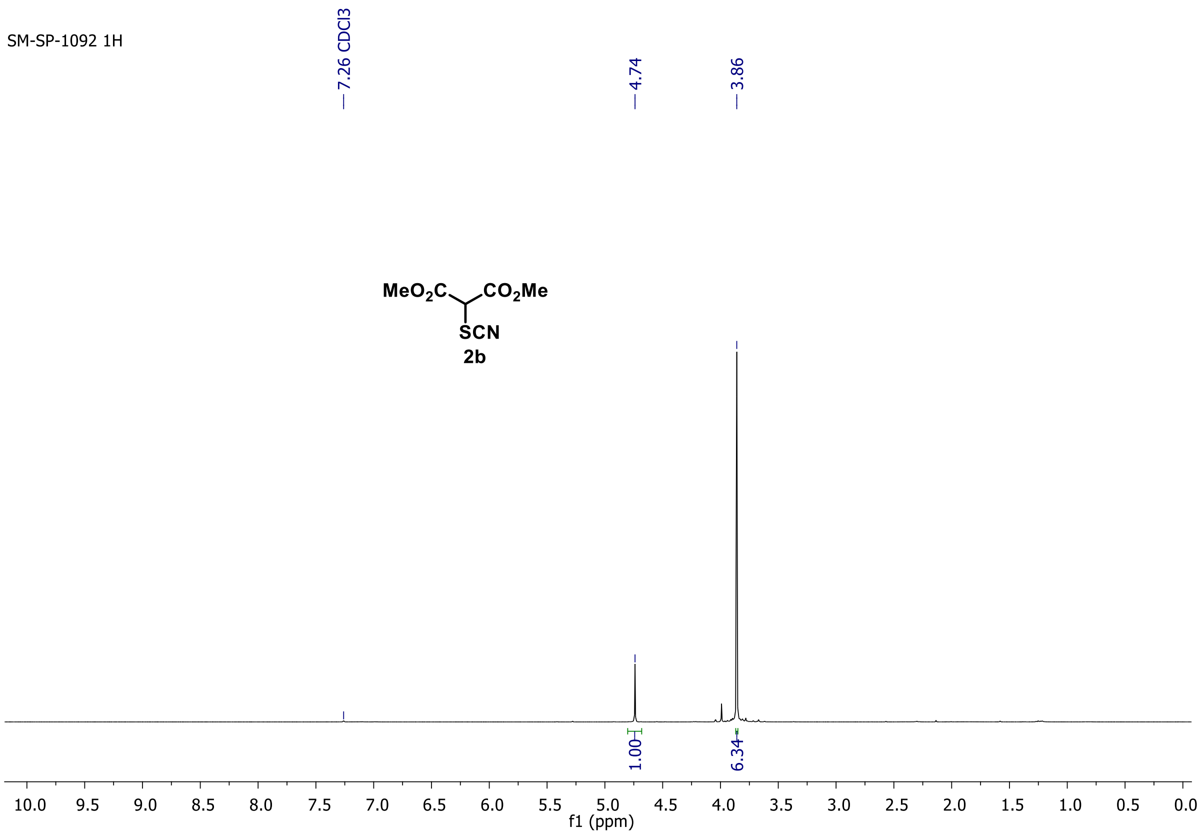


$^{13}C\{^1H\}$ NMR of **2b** (101 MHz, $CDCl_3$):

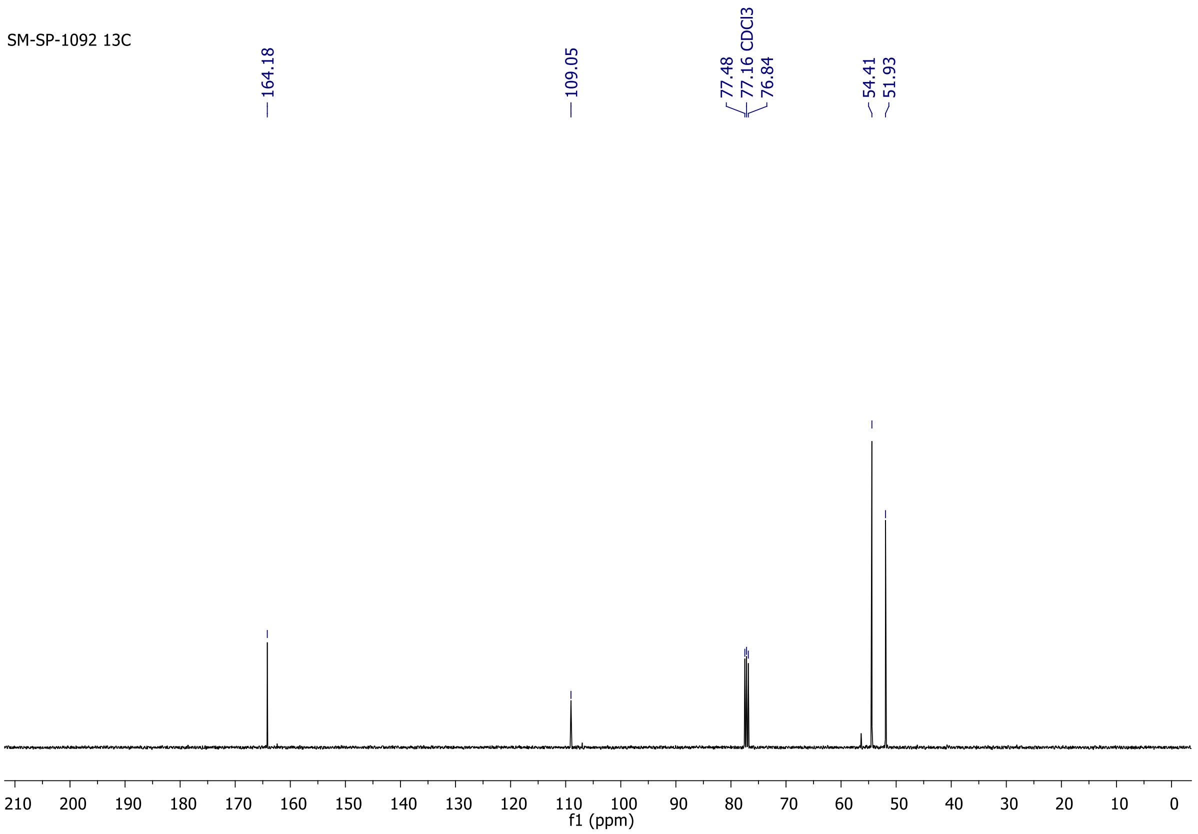

$^{1}$H NMR of **2c** (400 MHz, $CDCl_3$):

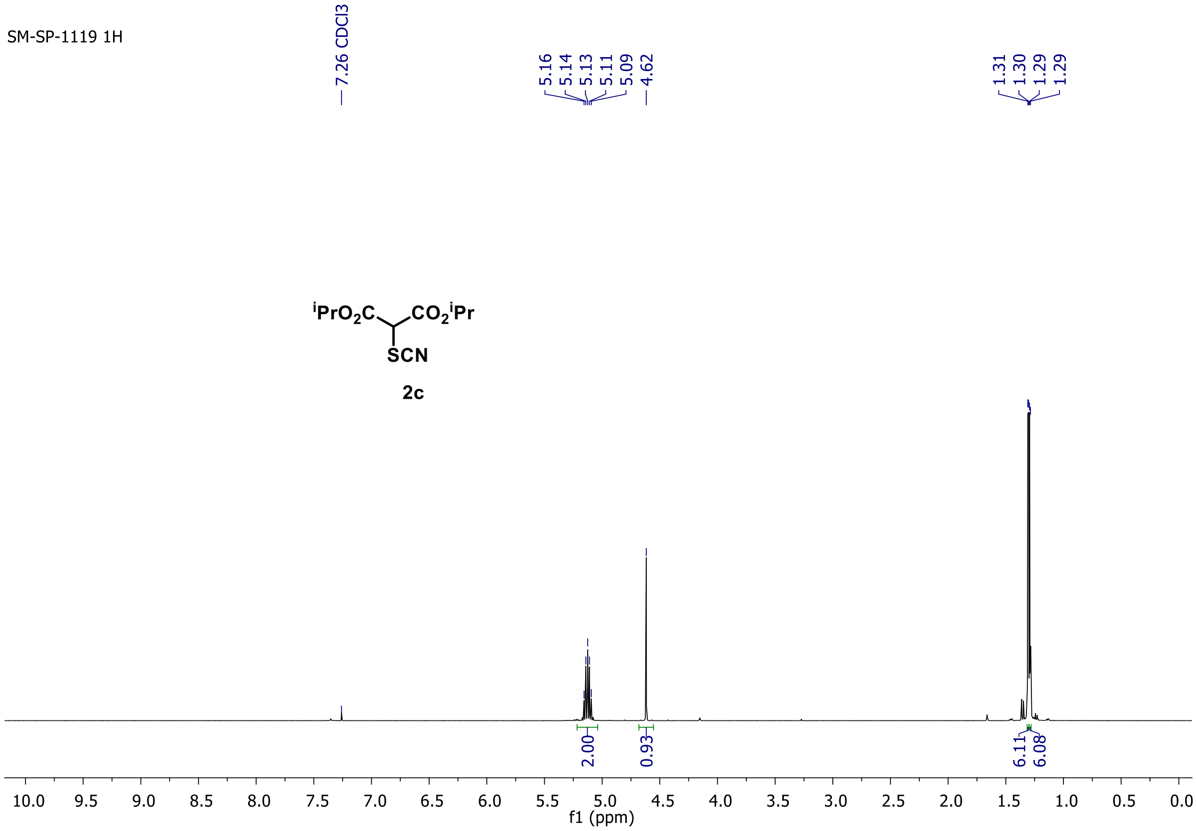


**2c**

$^{13}$C{$^{1}$H} NMR of **2c** (101 MHz, $CDCl_3$):

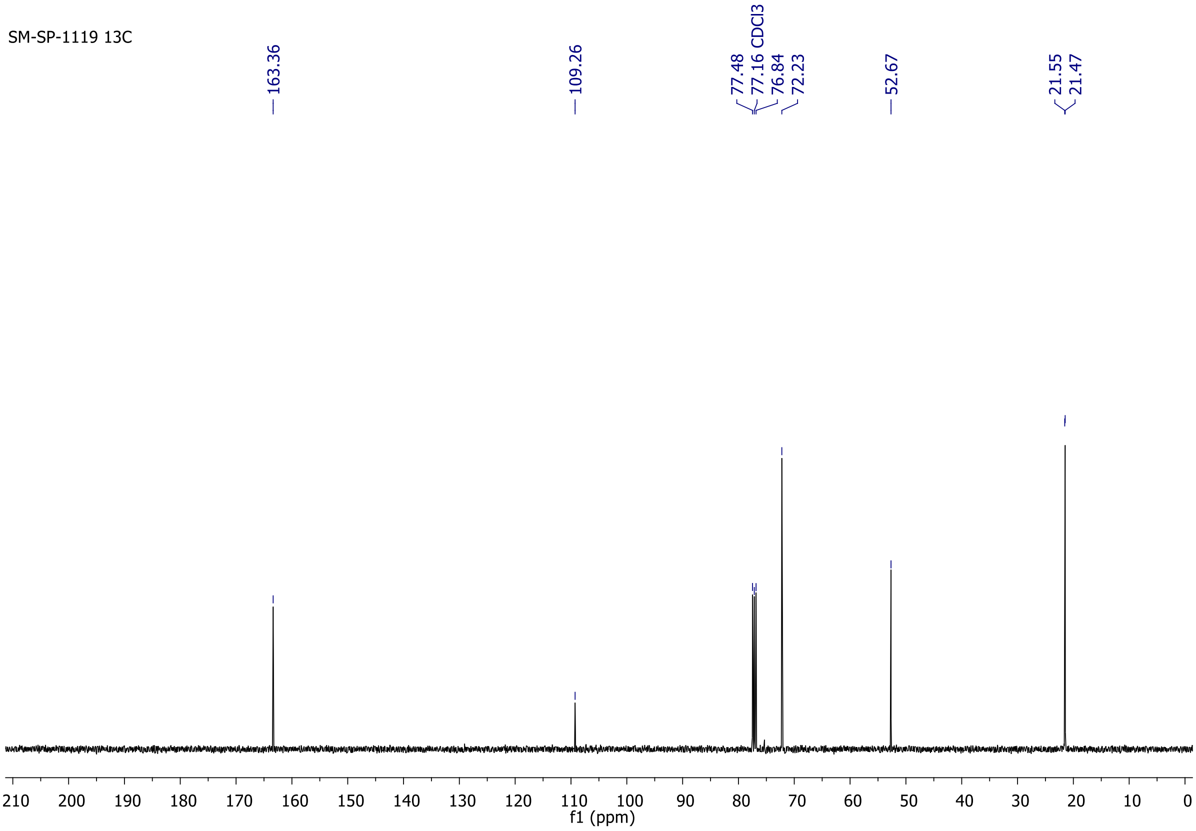

$^{1}H$ NMR of **2d** (400 MHz, $CDCl_3$):

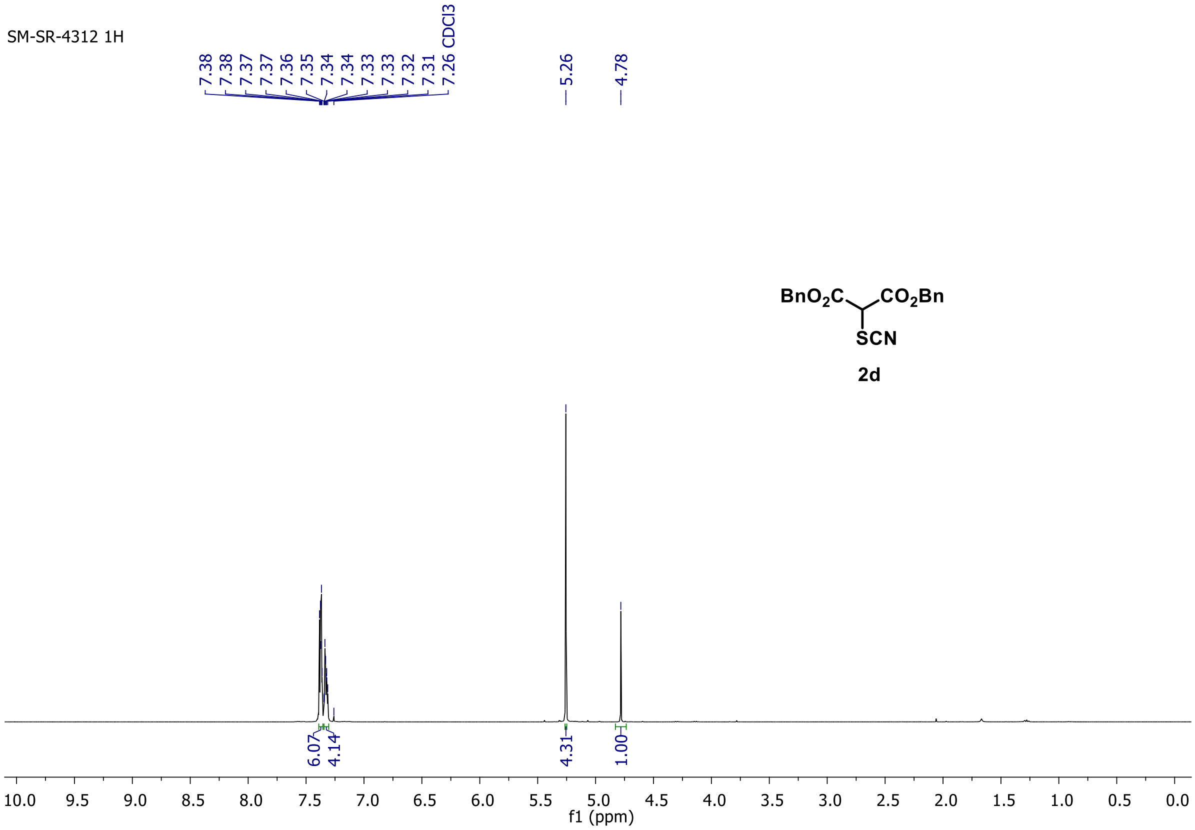


$^{13}C\{^{1}H\}$ NMR of **2d** (101 MHz, $CDCl_3$):

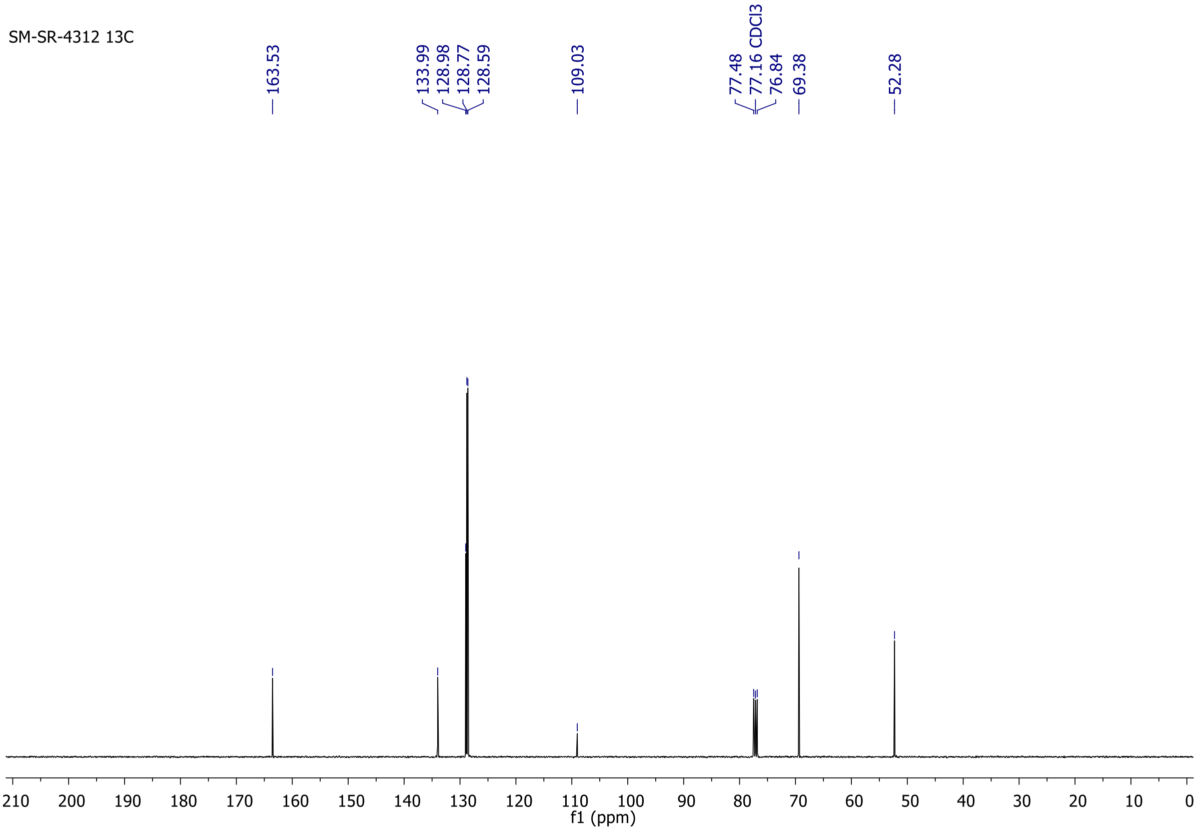

$^{1}H$ NMR of **2e** (400 MHz, $CDCl_3$):

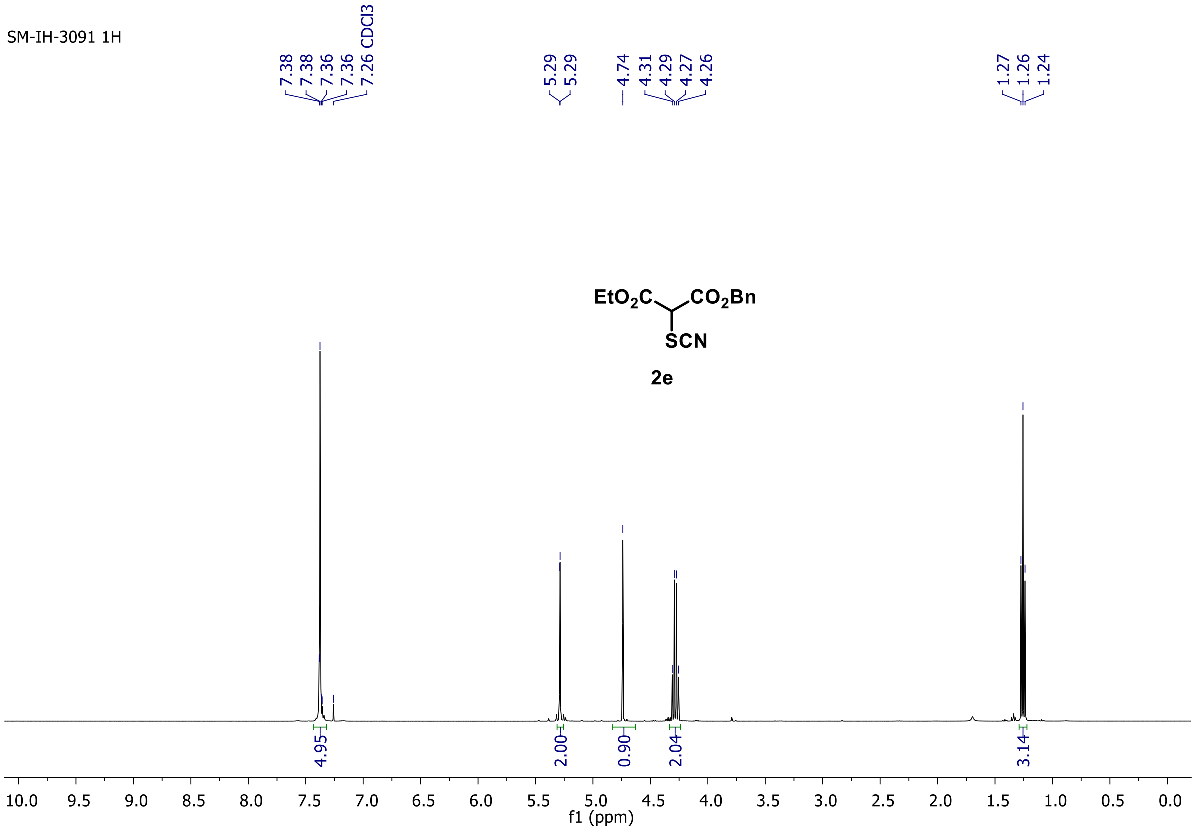


$^{13}C\{^{1}H\}$ NMR of **2e** (101 MHz, $CDCl_3$):

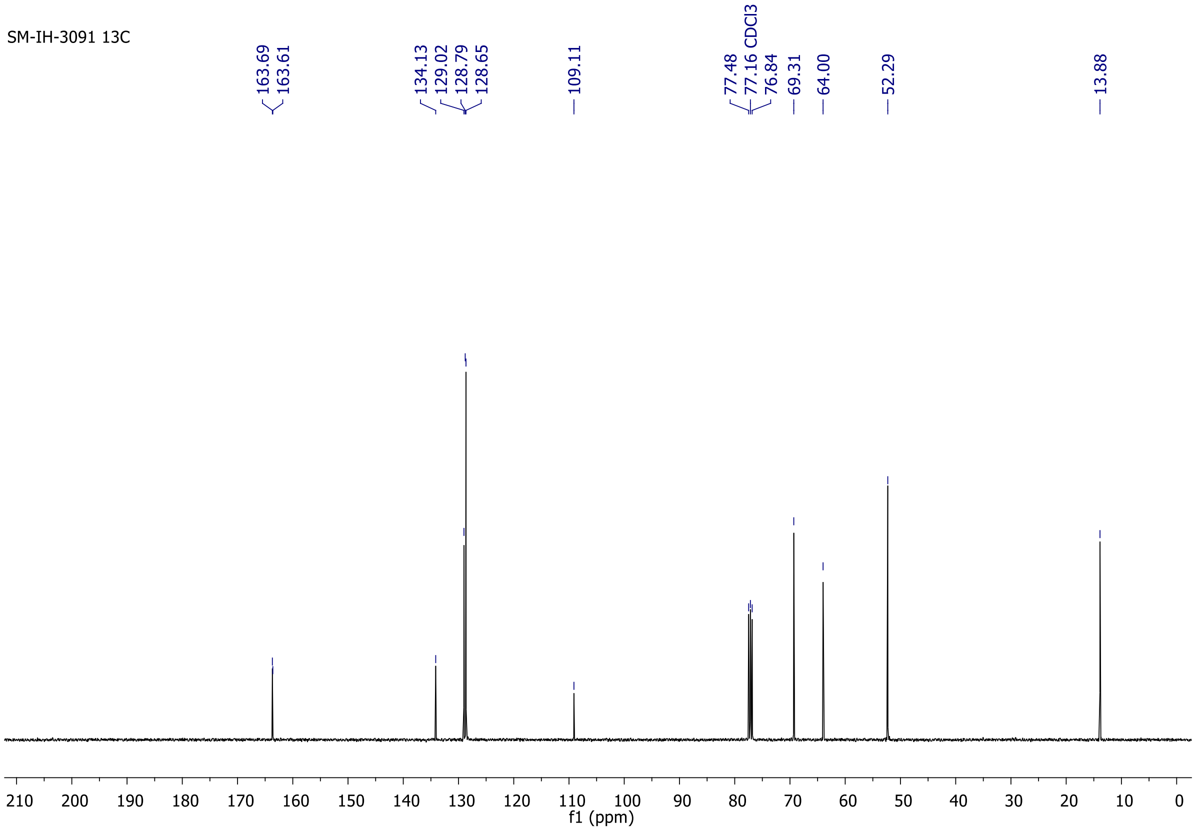

$^1H$ NMR of **2f** (400 MHz, $CDCl_3$):

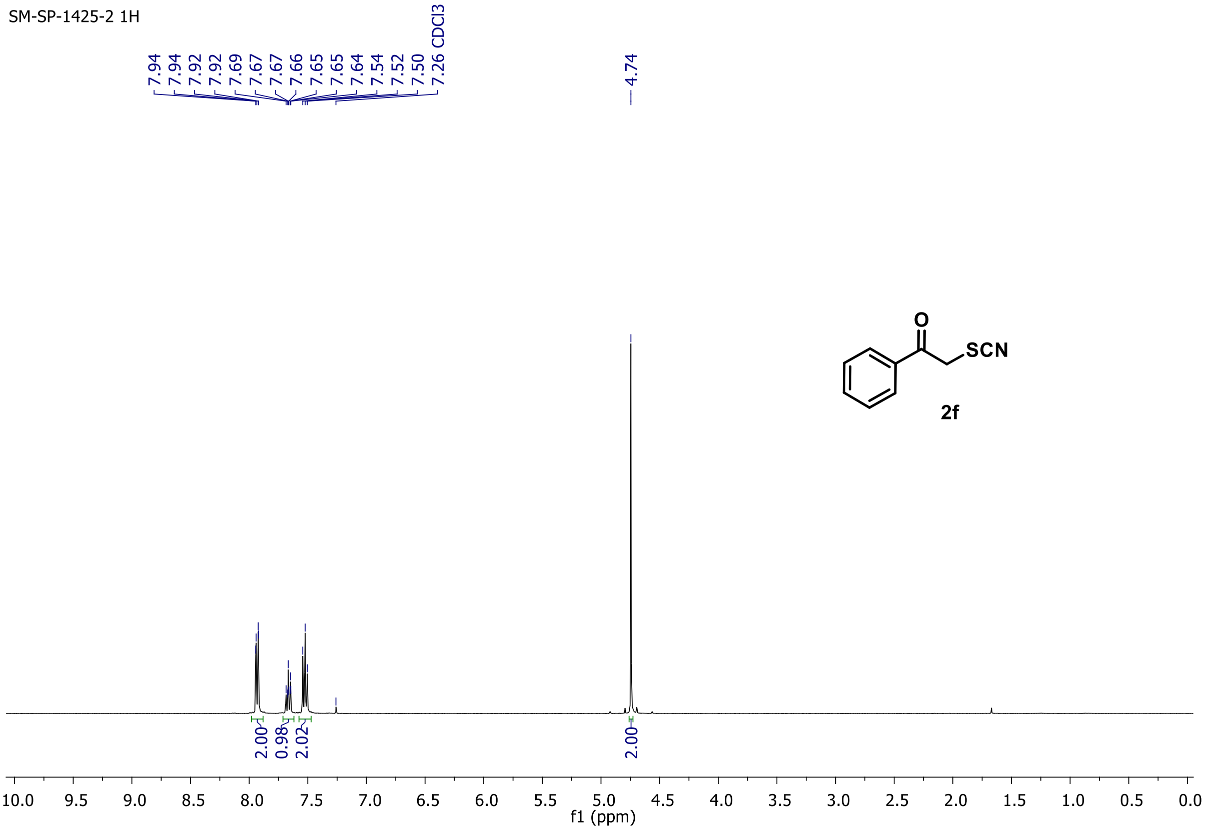


$^{13}C\{^1H\}$ NMR of **2f** (101 MHz, $CDCl_3$):

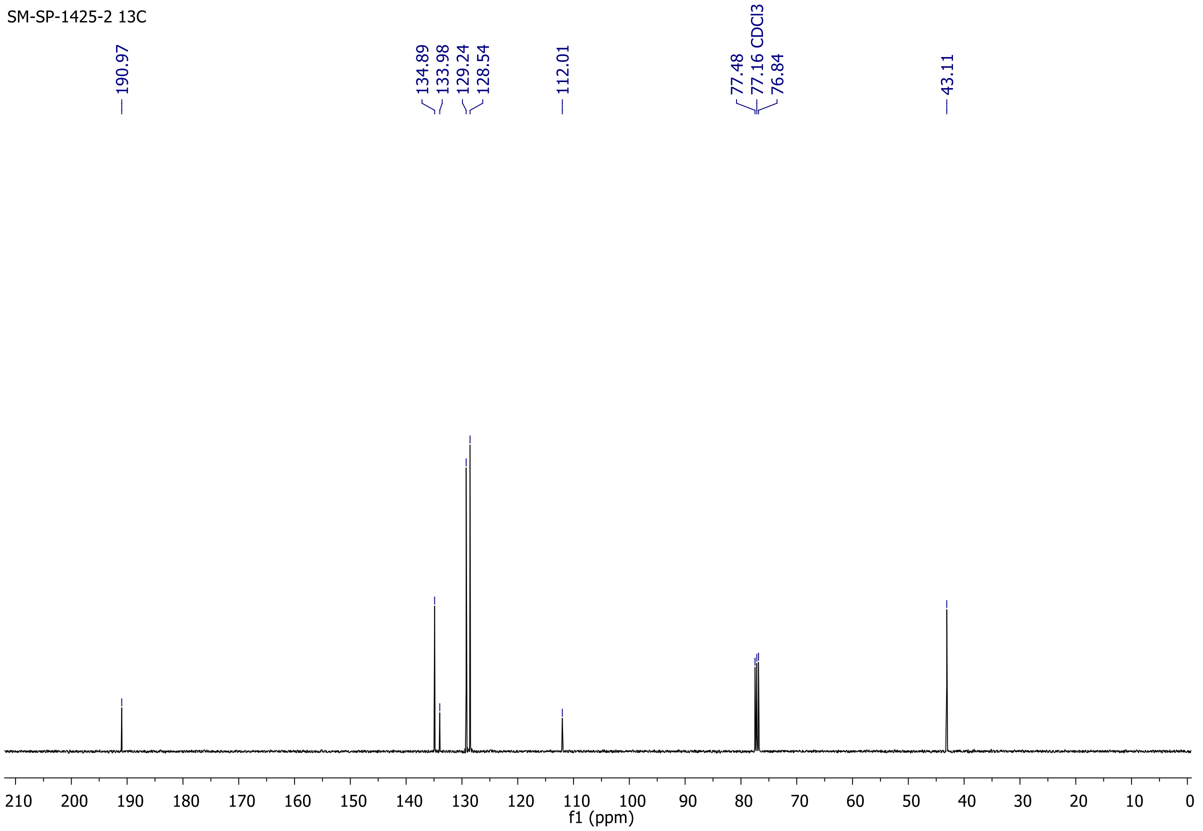

$^1H$ NMR of **2g** (400 MHz, $CDCl_3$):

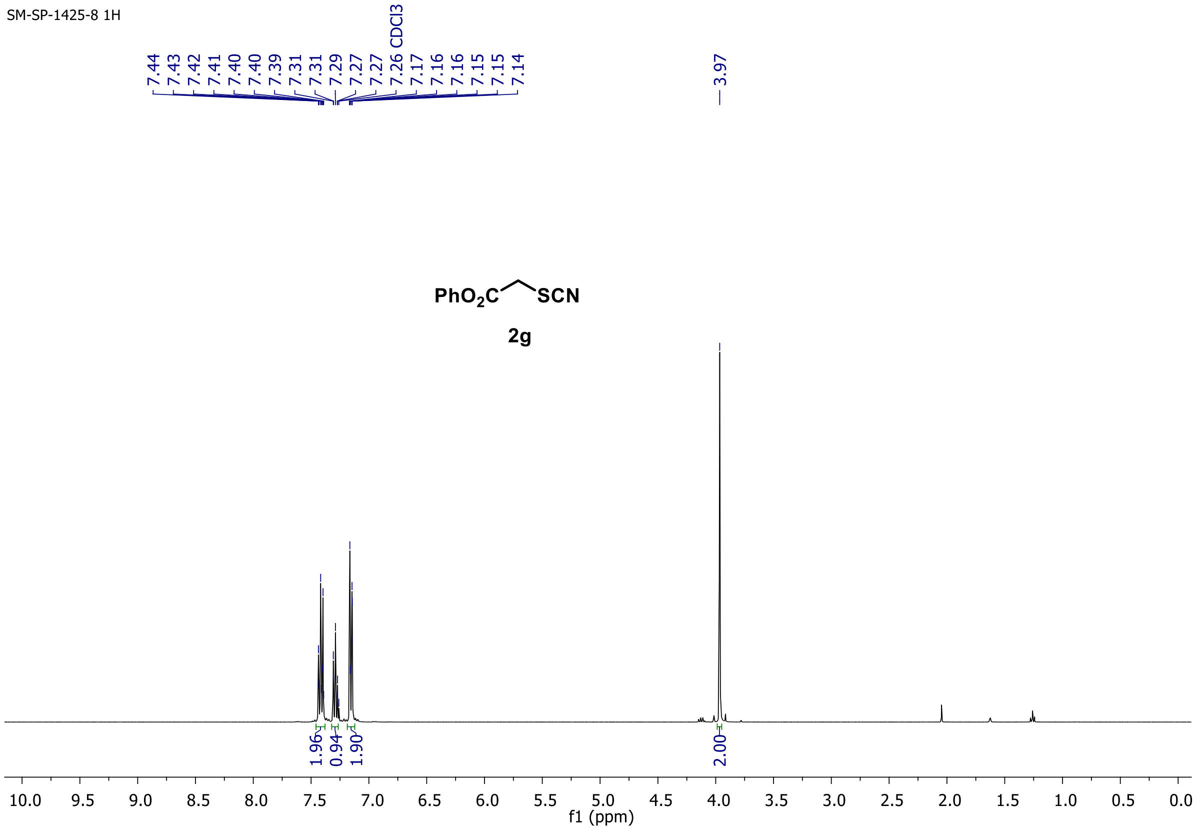


$^{13}C\{^1H\}$ NMR of **2g** (101 MHz, $CDCl_3$):

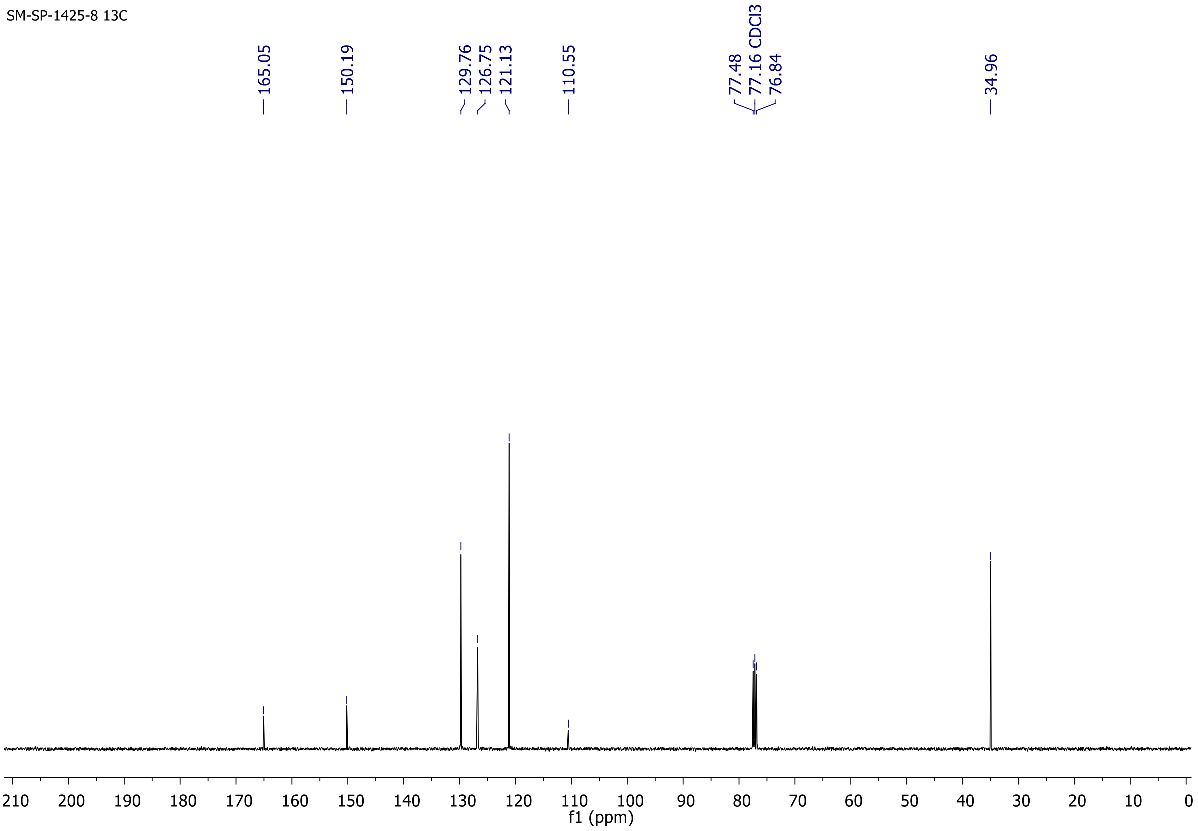

$^1$H NMR of **2h** (400 MHz, $CDCl_3$):

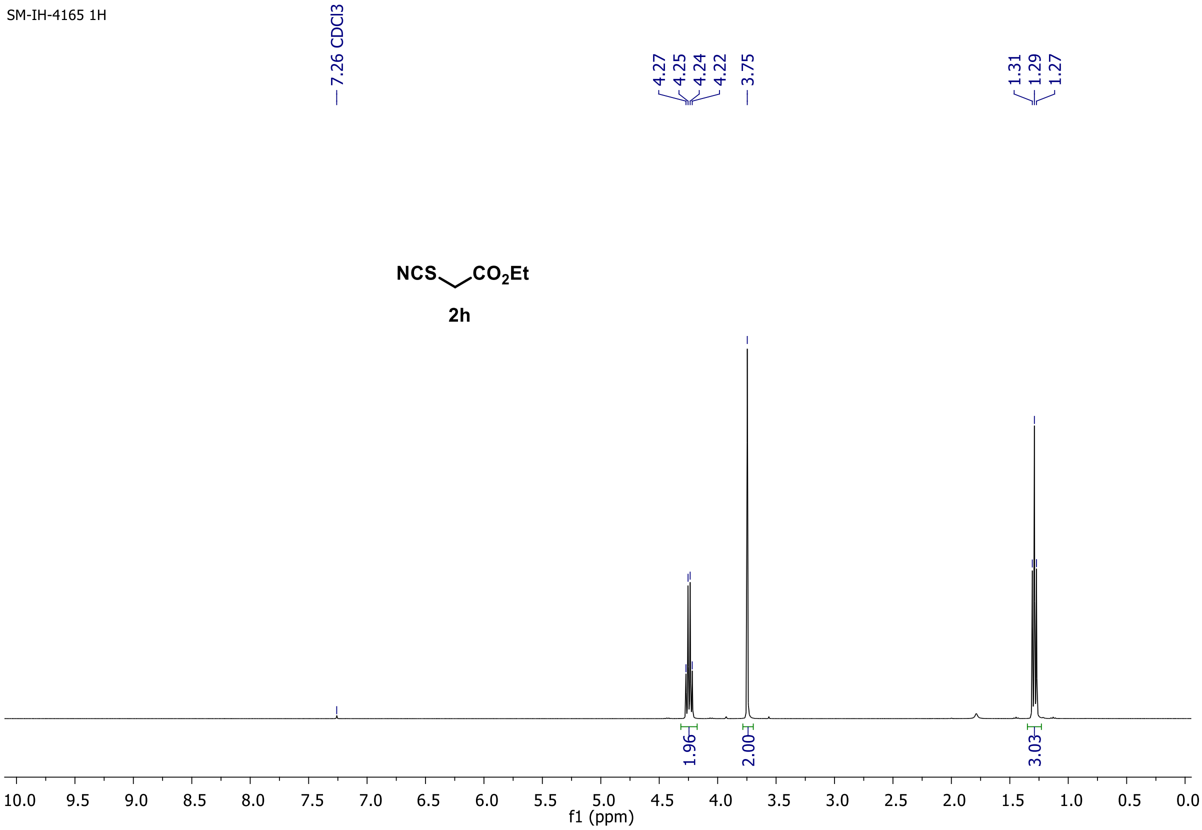


$^{13}C\{^1H\}$ NMR of **2h** (101 MHz, $CDCl_3$):

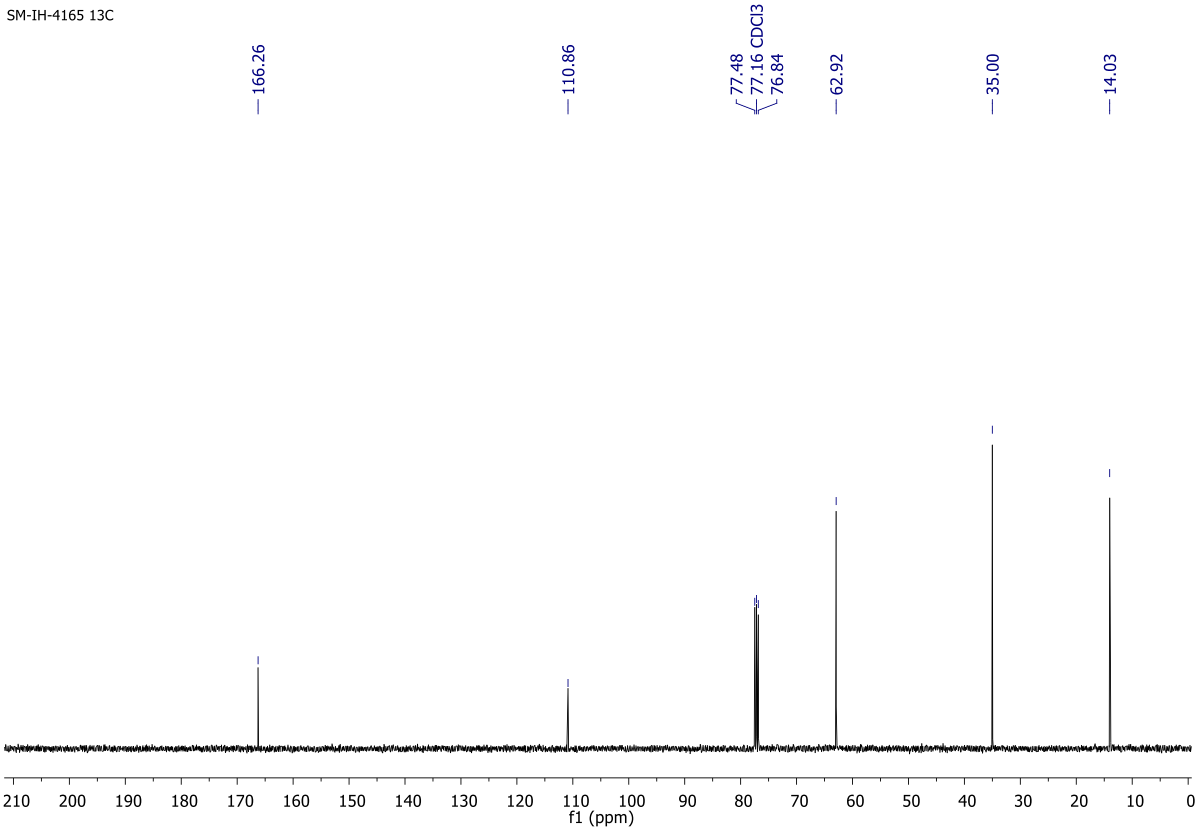

$^{1}H$ NMR of **2i** (400 MHz, $CDCl_3$):

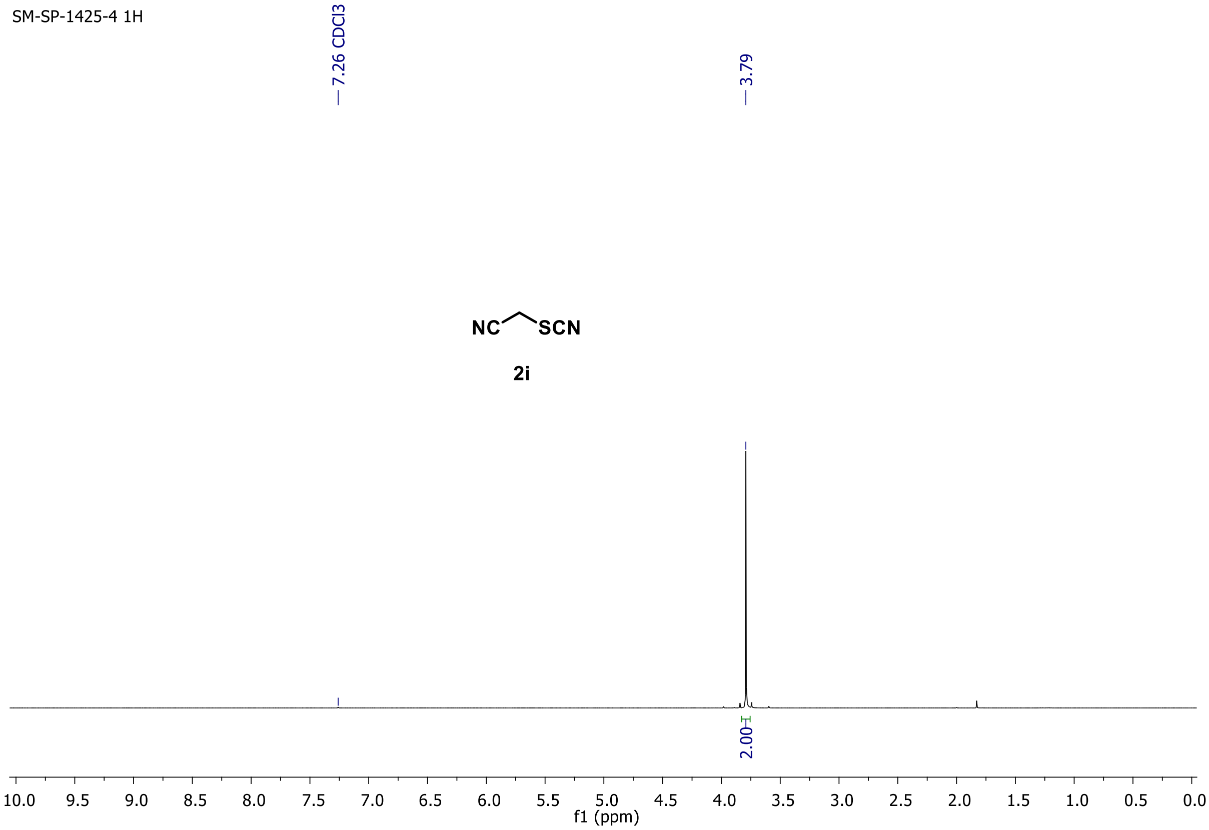


$^{13}C\{^{1}H\}$ NMR of **2i** (101 MHz, $CDCl_3$):

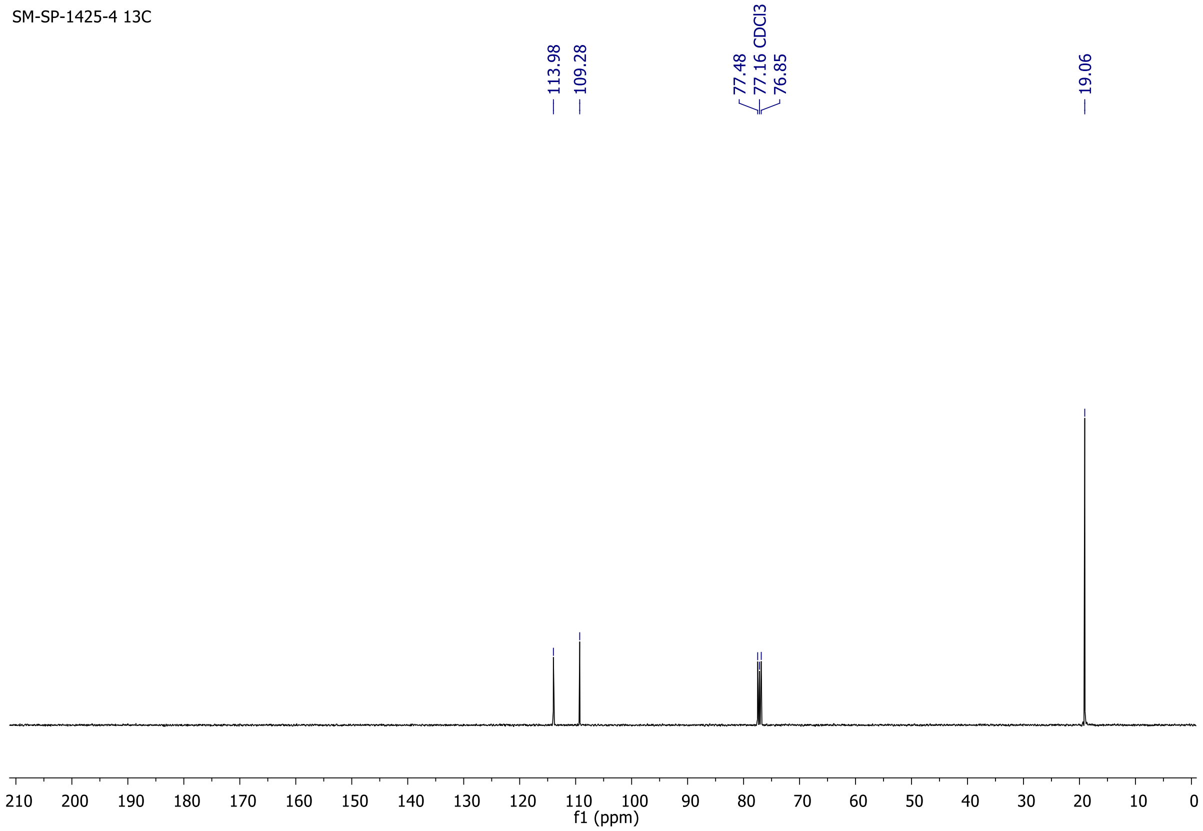

$^{1}H$ NMR of **2j** (400 MHz, $CDCl_3$):

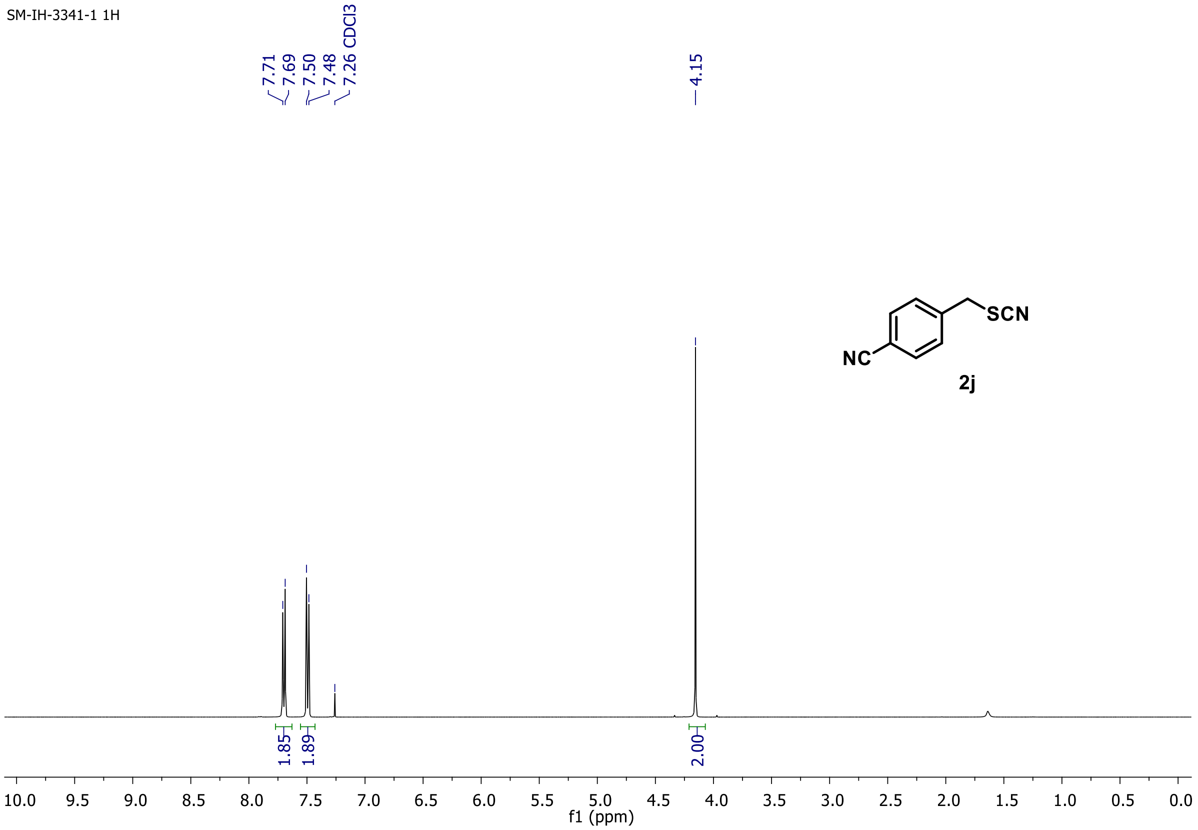


$^{13}C\{^{1}H\}$ NMR of **2j** (101 MHz, $CDCl_3$):

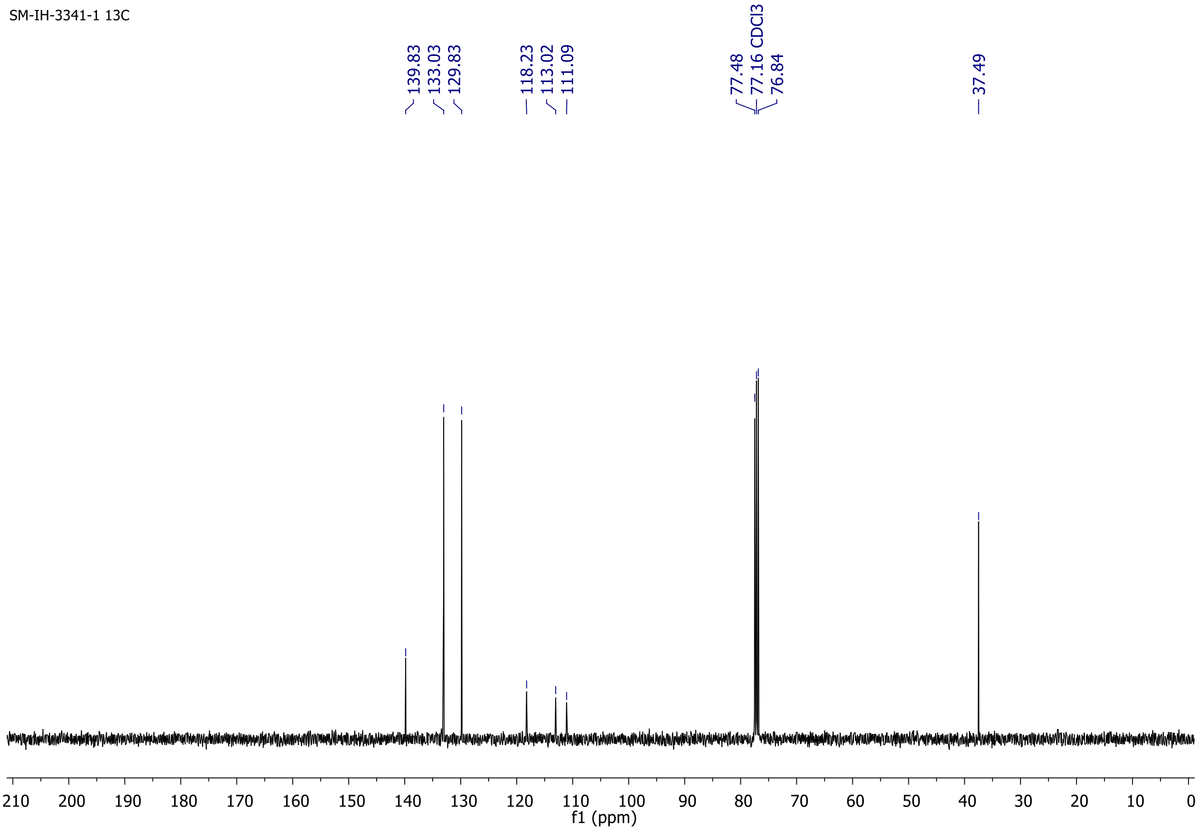

$^1H$ NMR of **2k** (400 MHz, $CDCl_3$):

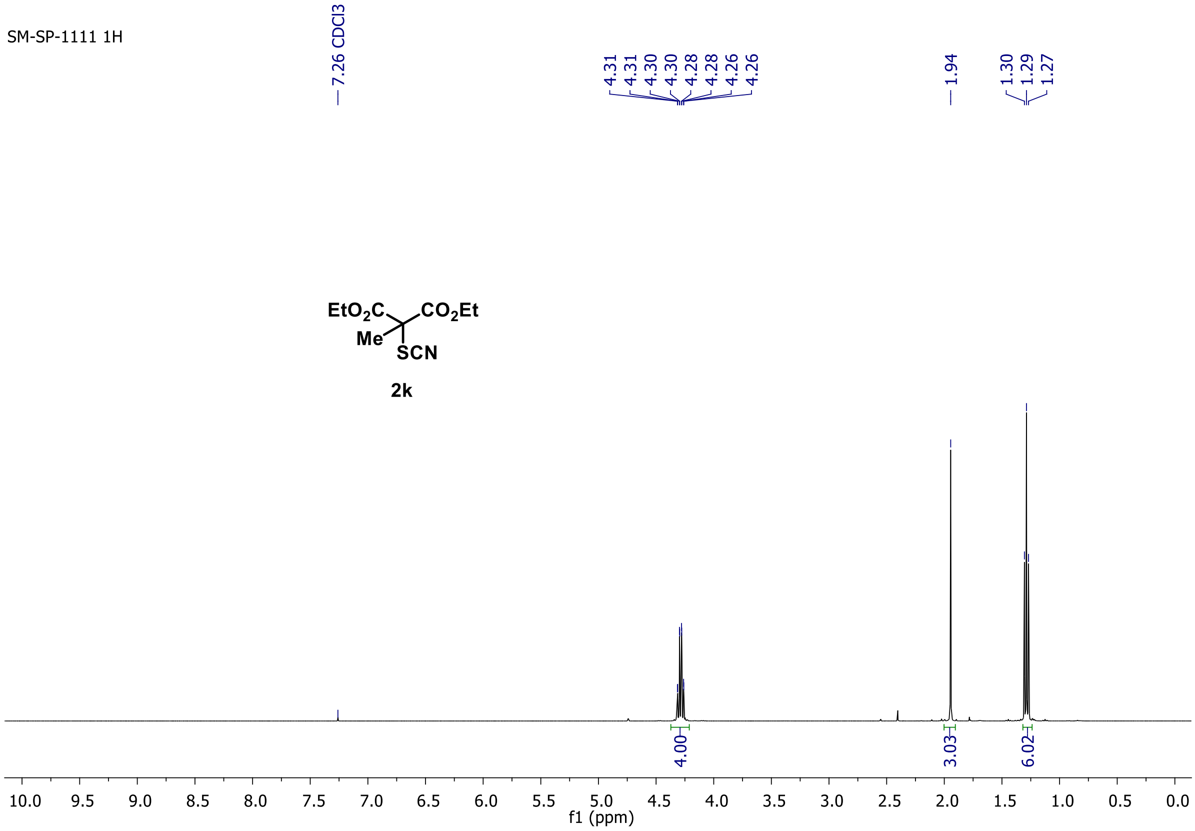


$^{13}C\{^1H\}$ NMR of **2k** (101 MHz, $CDCl_3$):

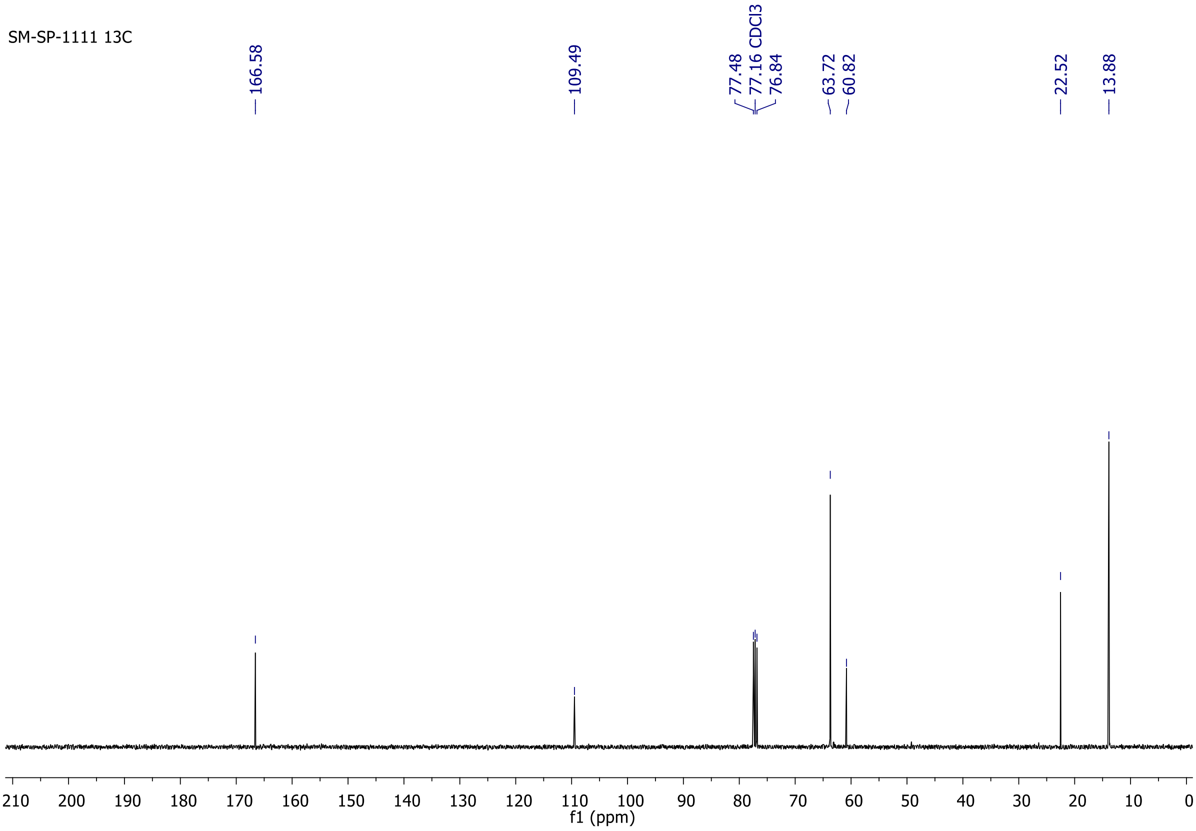

$^1$H NMR of **2l** (400 MHz, $CDCl_3$):

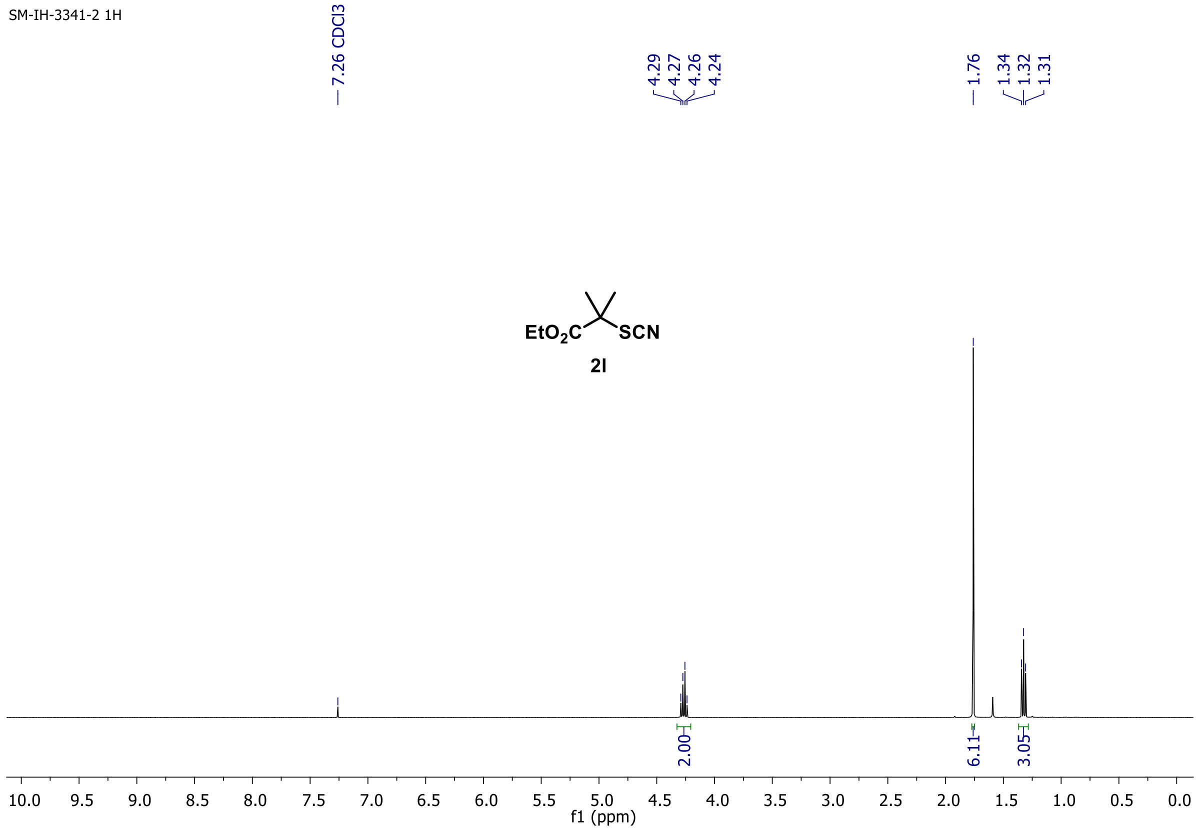


$^{13}C\{^1H\}$ NMR of **2l** (101 MHz, $CDCl_3$):

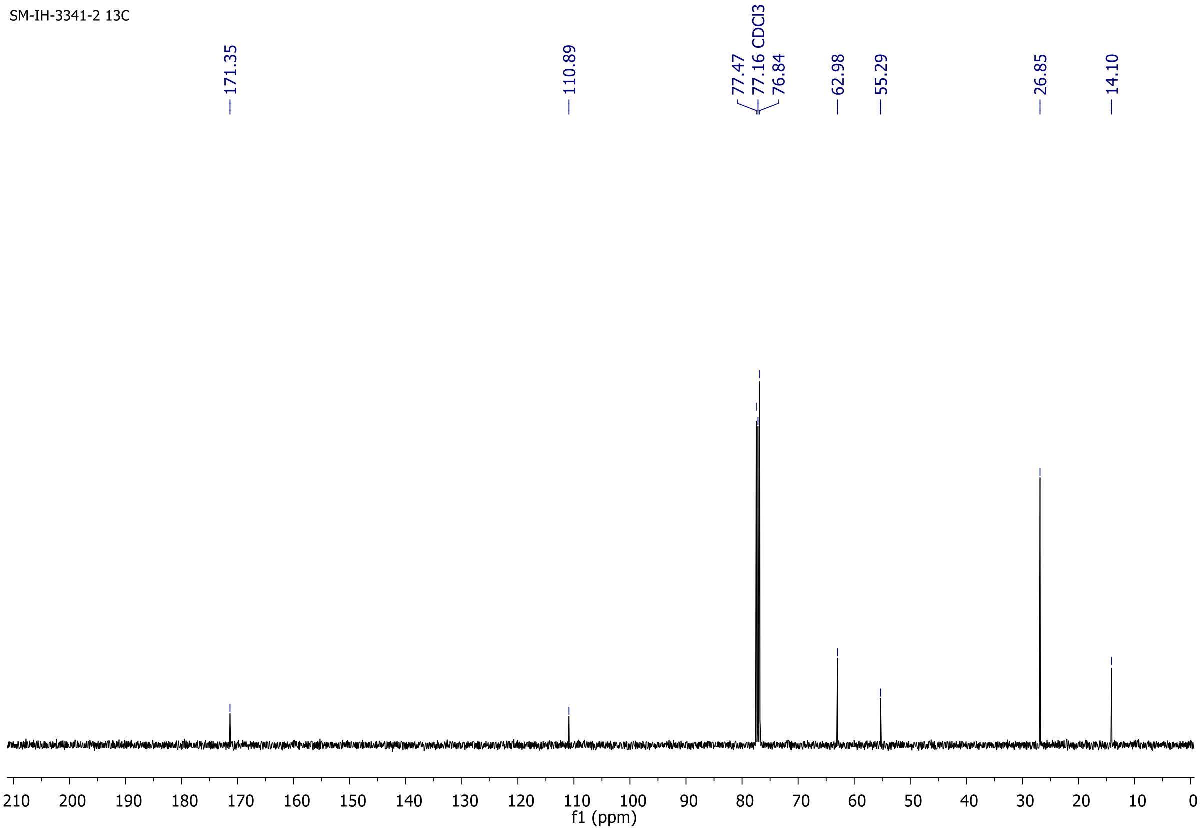

<sup></sup>

$^1$H NMR of **2m** (400 MHz, $CDCl_3$):

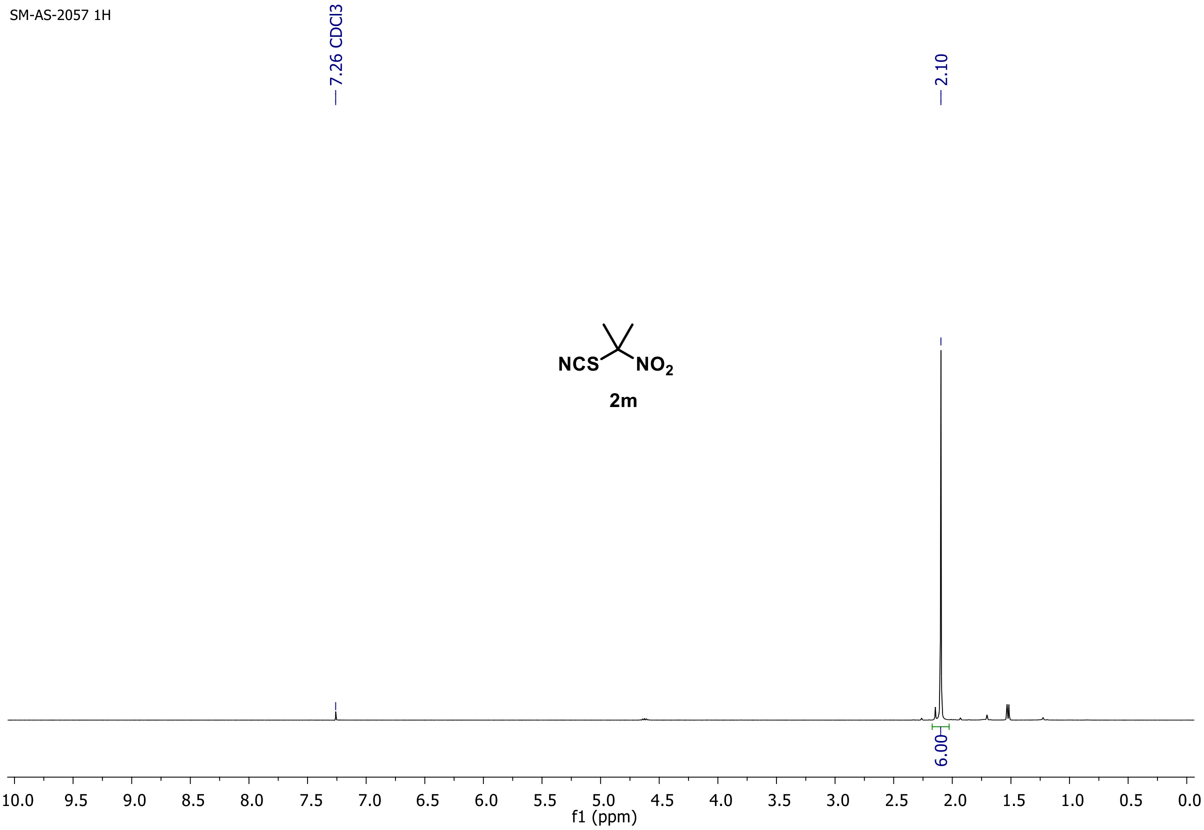


$^{13}C\{^1H\}$ NMR of **2m** (101 MHz, $CDCl_3$):

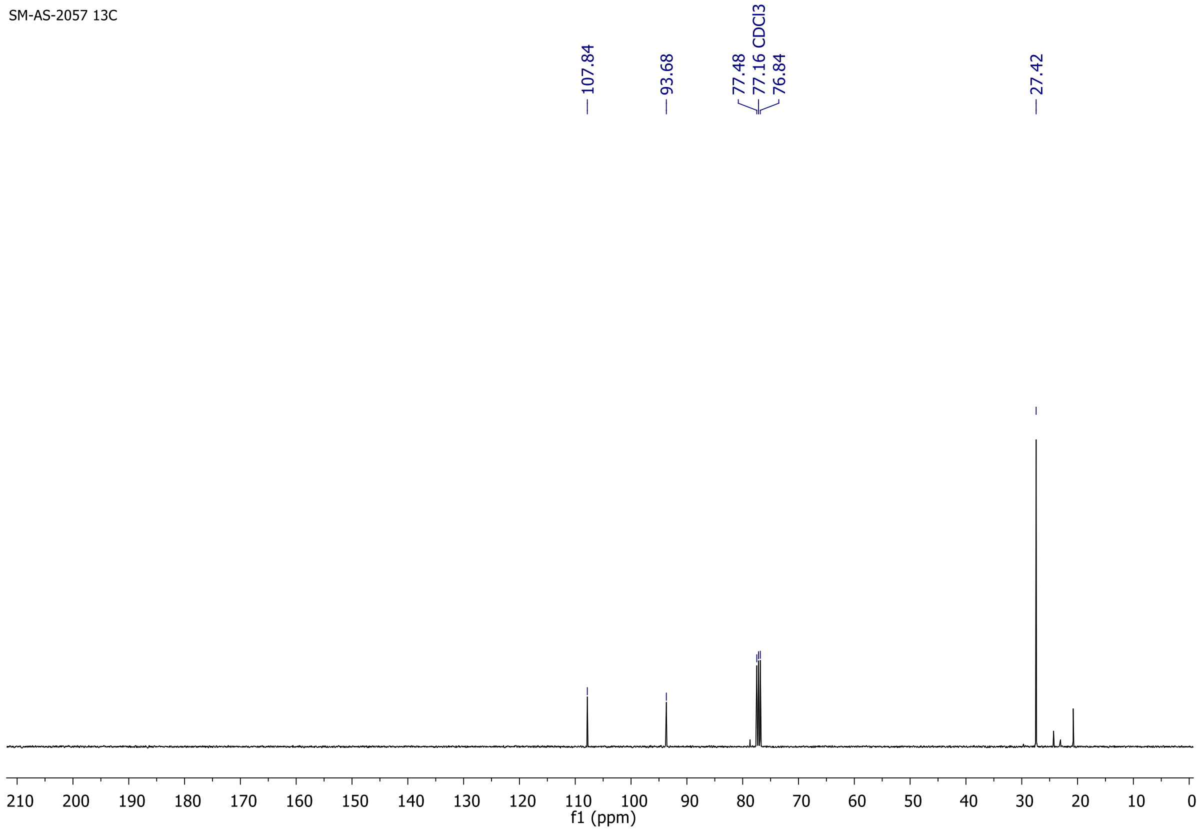

$^1$H NMR of **2n** (400 MHz, $CDCl_3$):

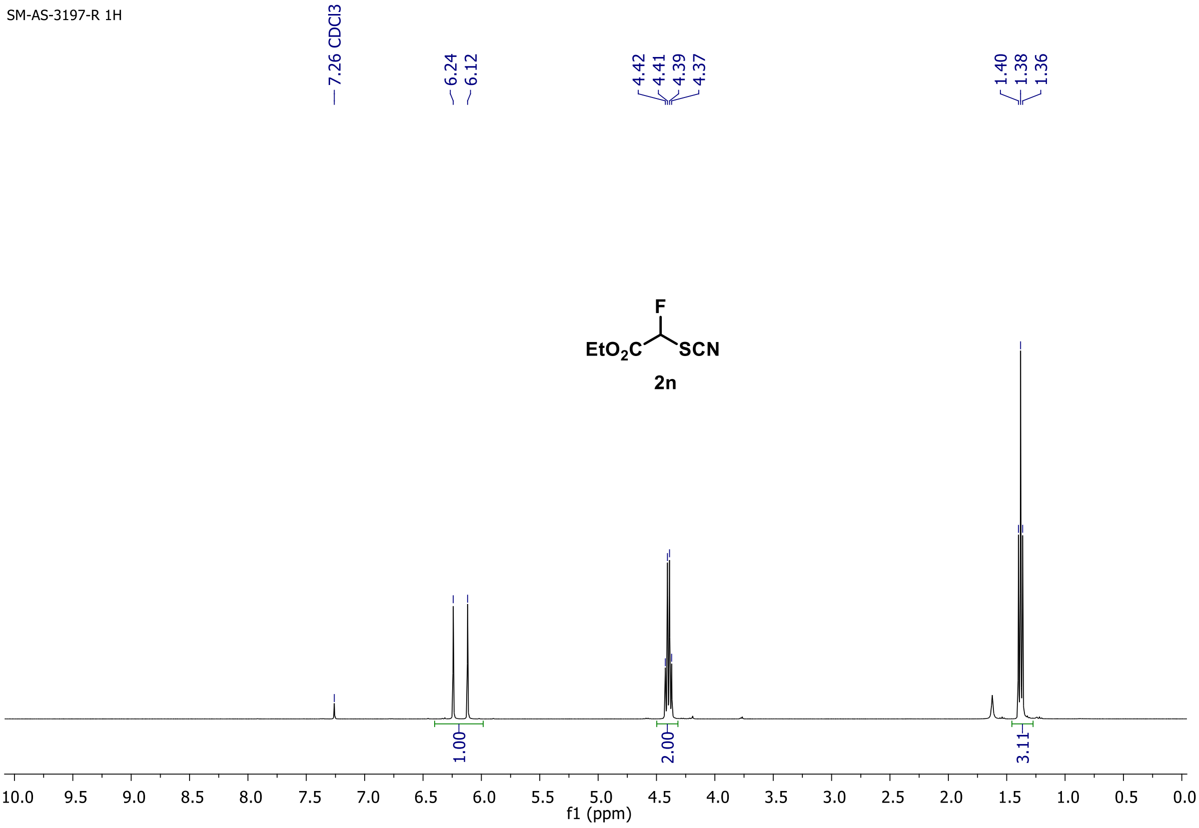


$^{13}$C{$^1$H} NMR of **2n** (101 MHz, $CDCl_3$):

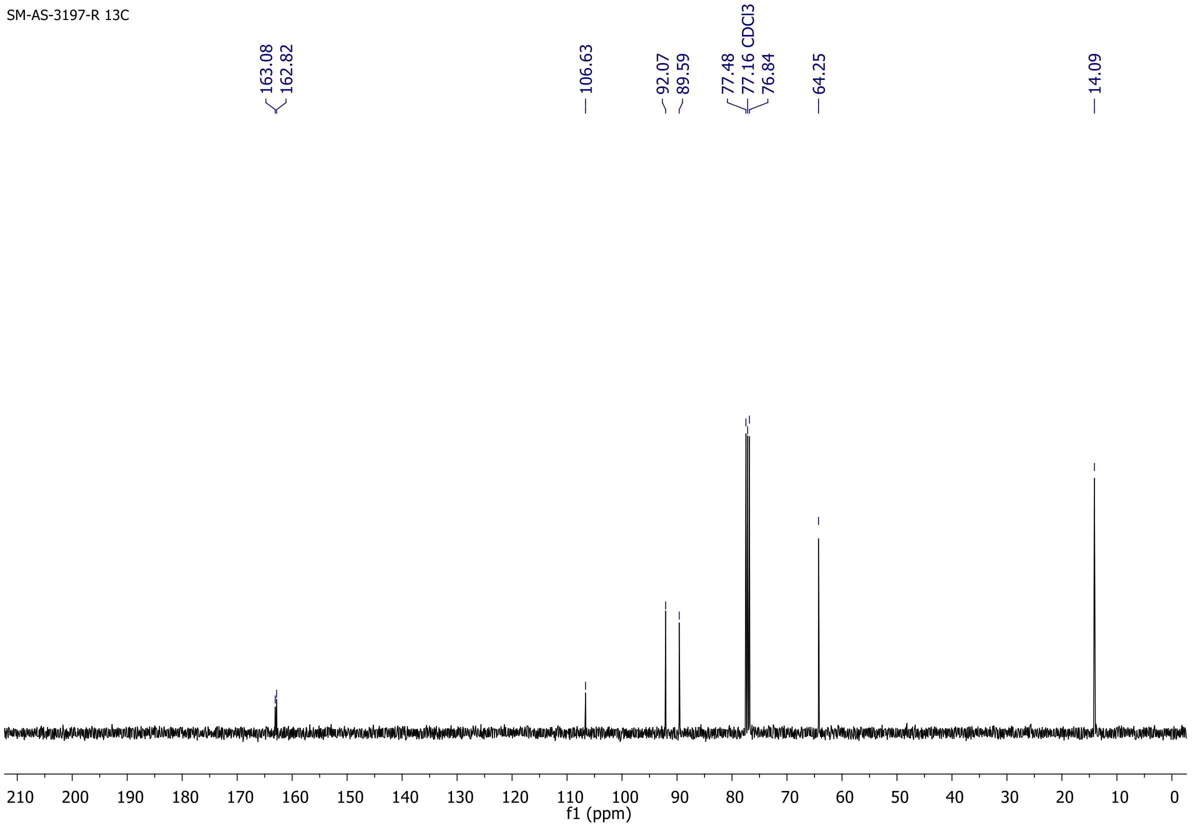

$^{19}F$ NMR of **2n** (377 MHz, $CDCl_3$):

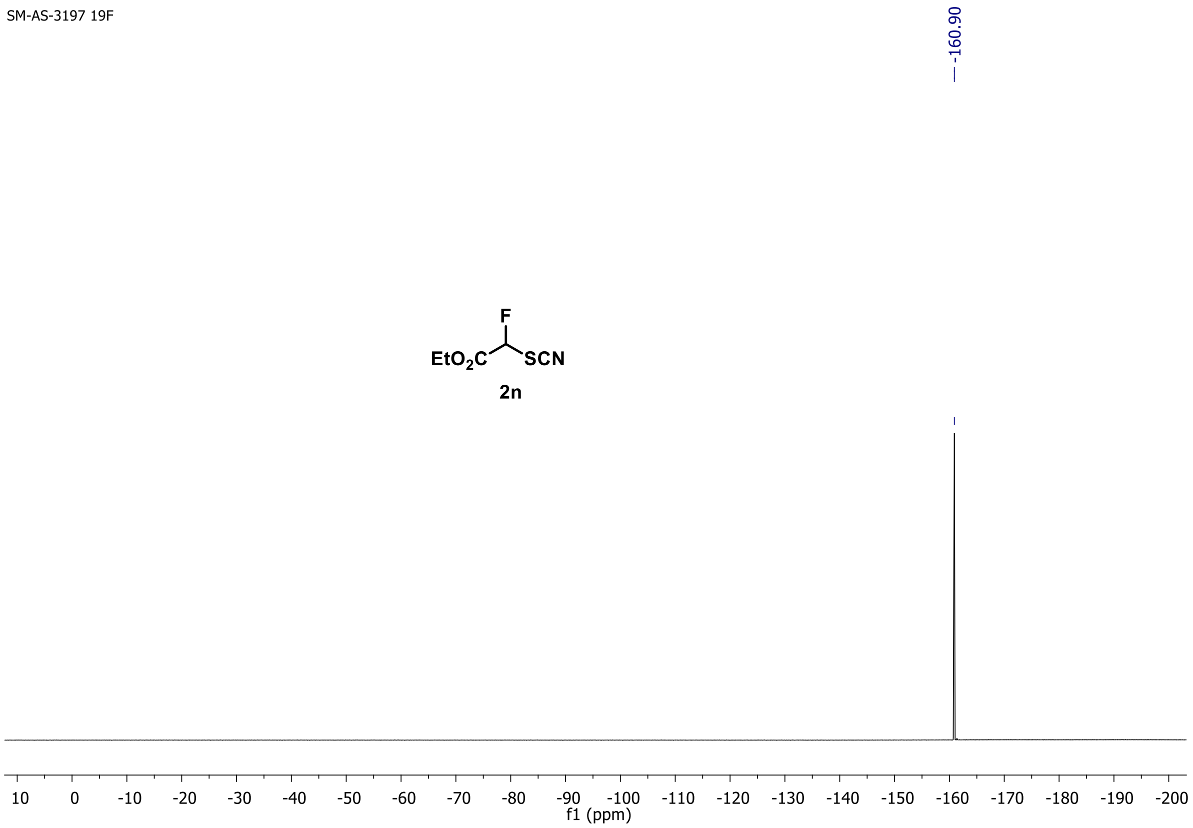

$^{1}H$ NMR of **2o** (400 MHz, $CDCl_3$):

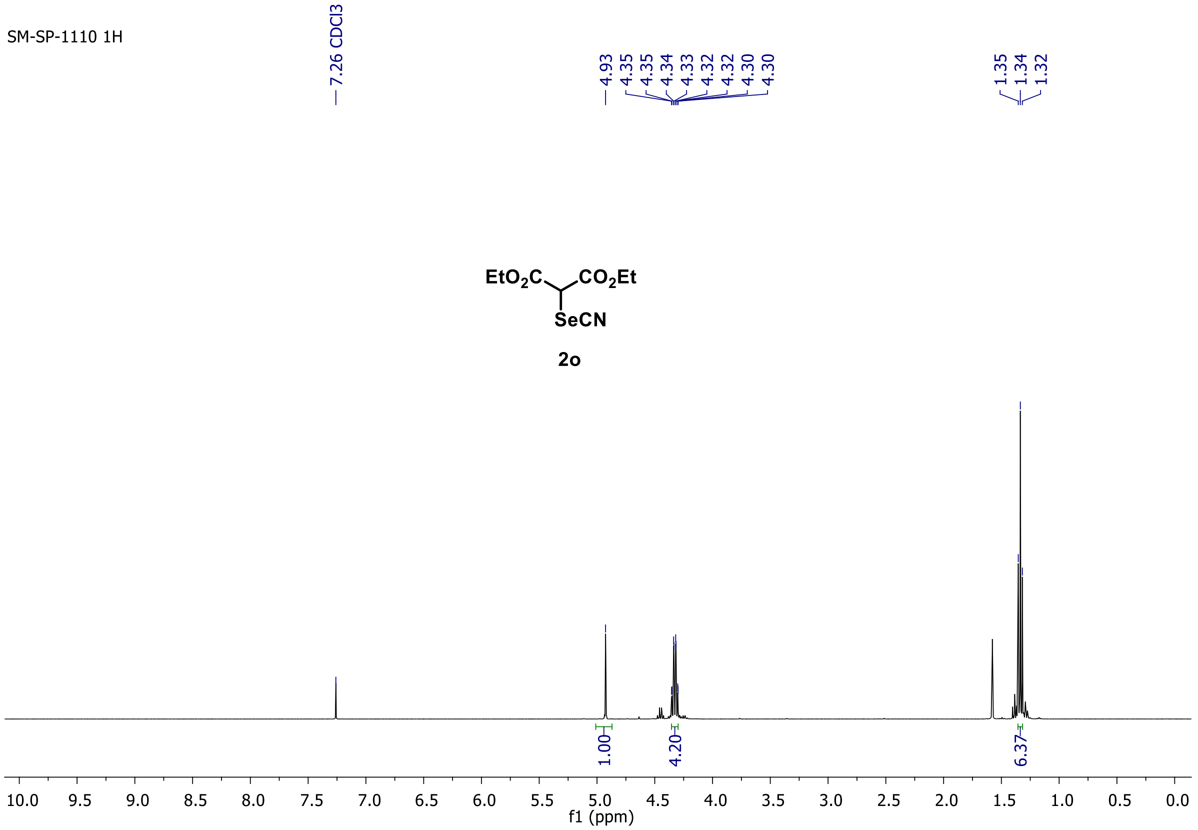


$^{13}C\{^{1}H\}$ NMR of **2o** (101 MHz, $CDCl_3$):

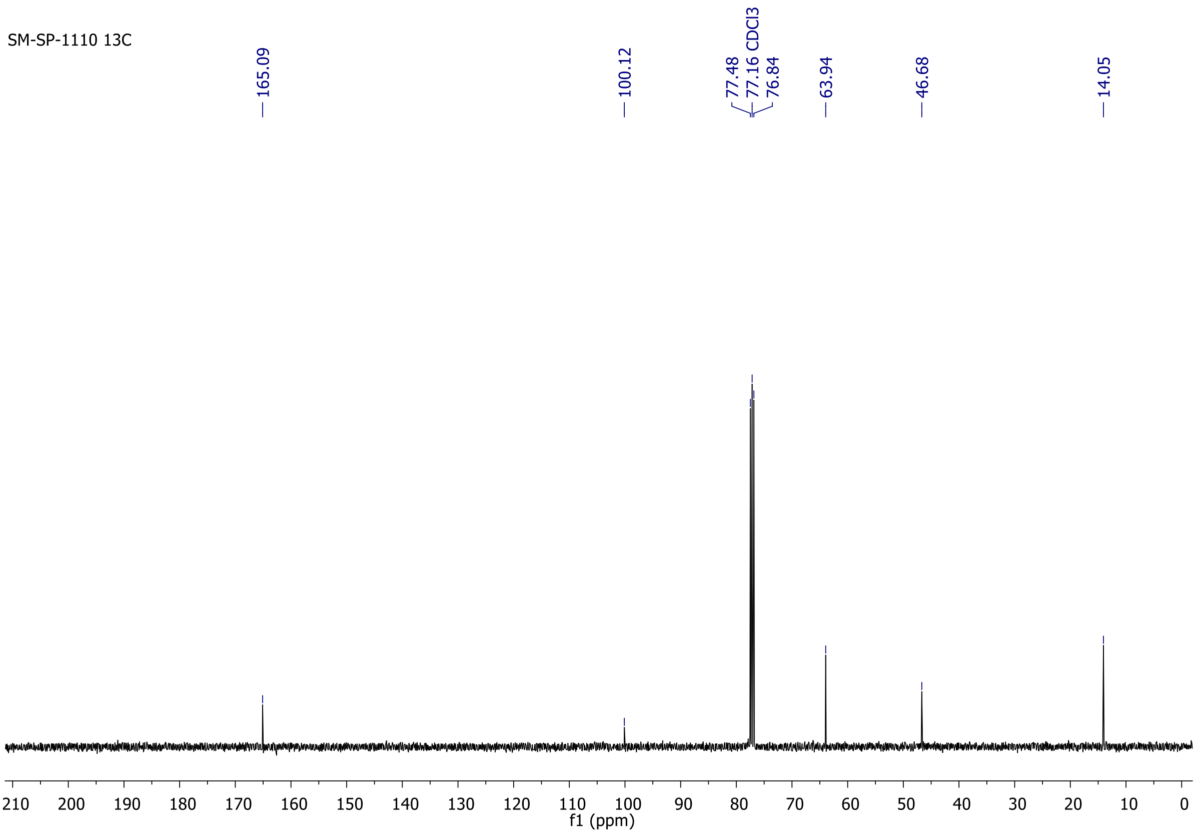

$^{1}H$ NMR of **2p** (400 MHz, $CDCl_3$):

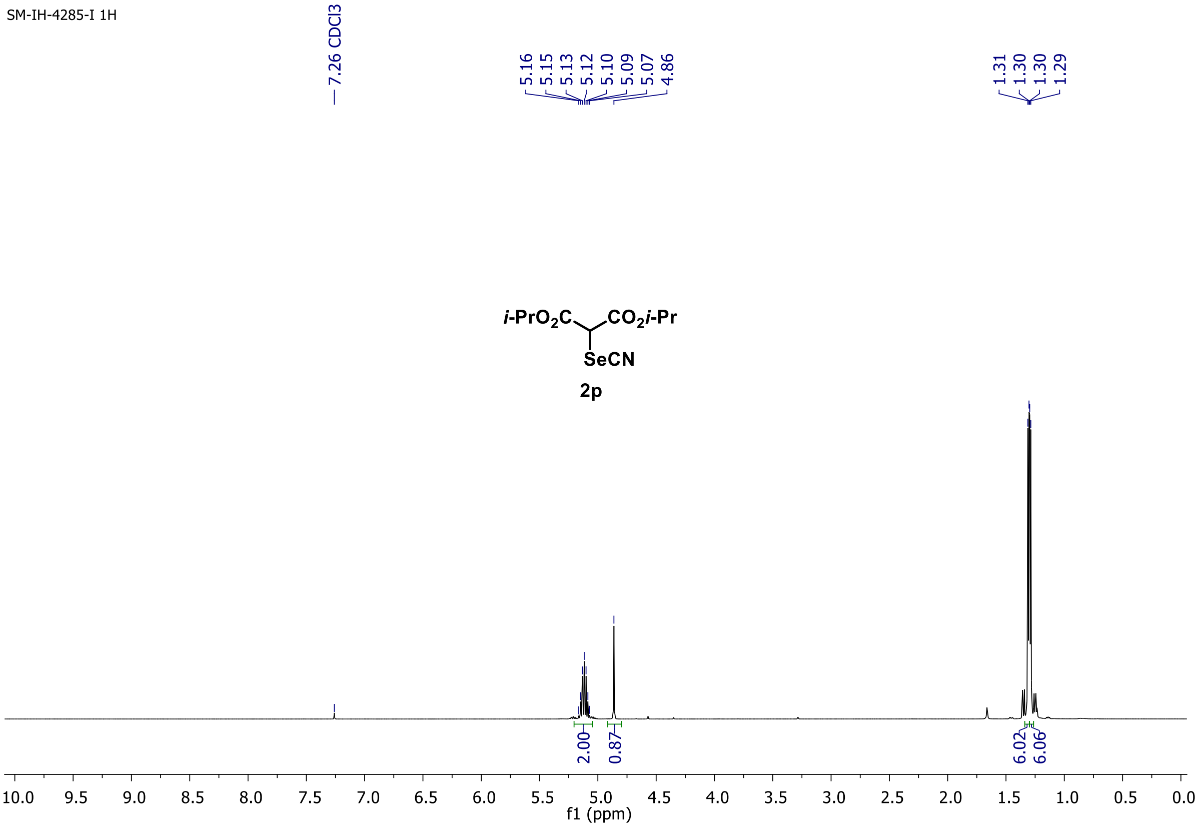


$^{13}C\{^{1}H\}$ NMR of **2p** (101 MHz, $CDCl_3$):

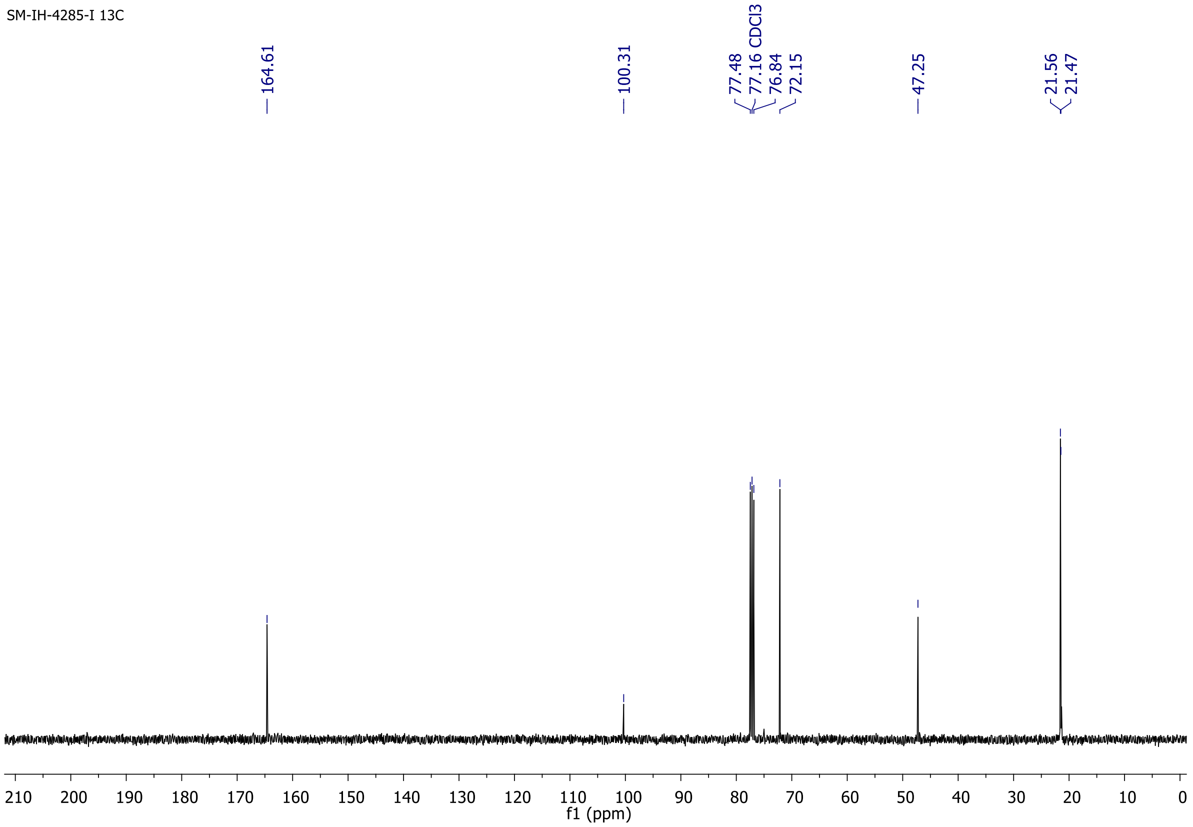

$^{1}H$ NMR of **2q** (400 MHz, $CDCl_3$):

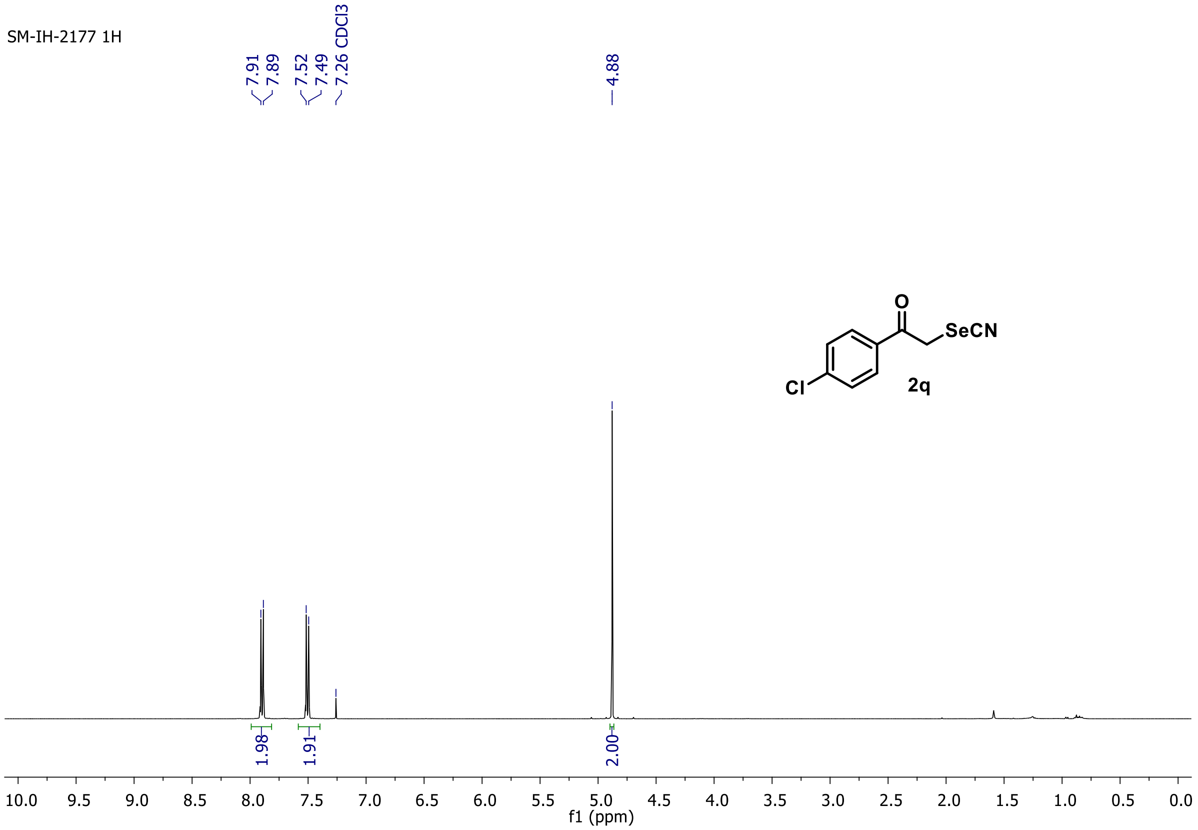


$^{13}C\{^{1}H\}$ NMR of **2q** (101 MHz, $CDCl_3$):

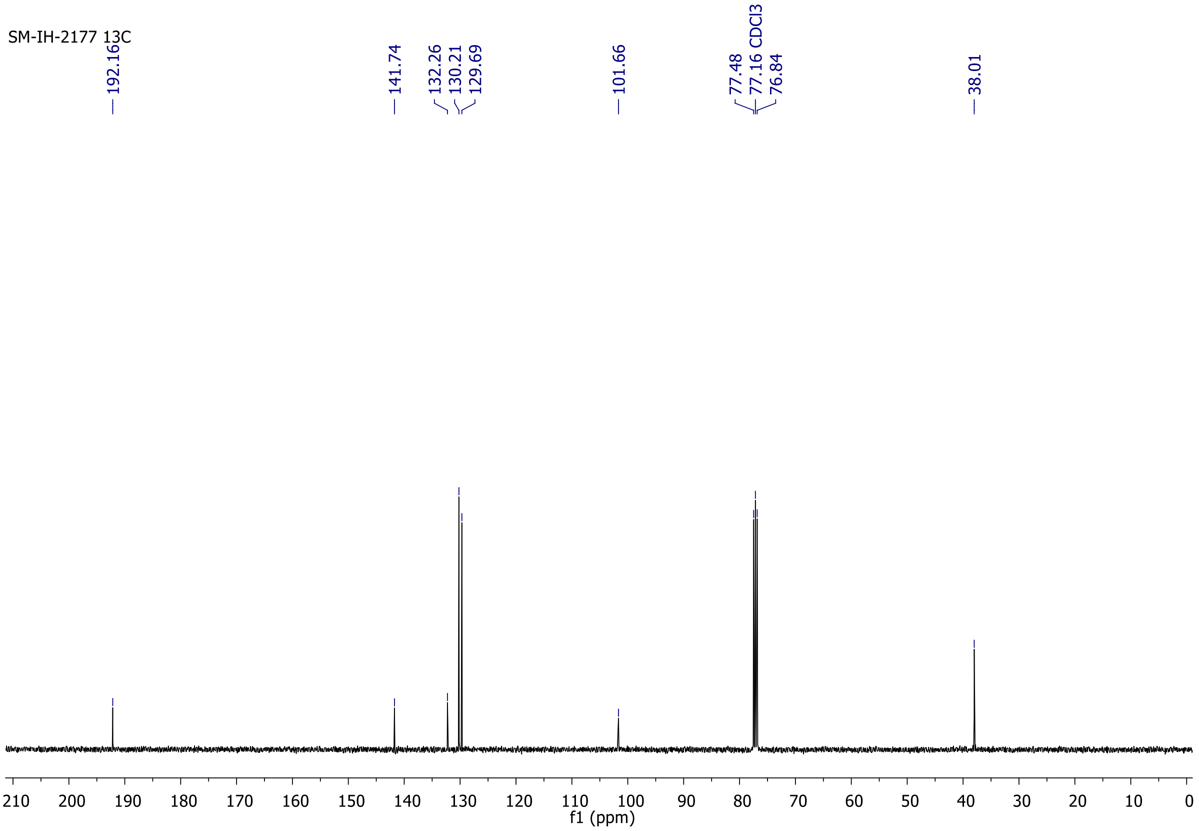

$^1$H NMR of **2r** (400 MHz, $CDCl_3$):

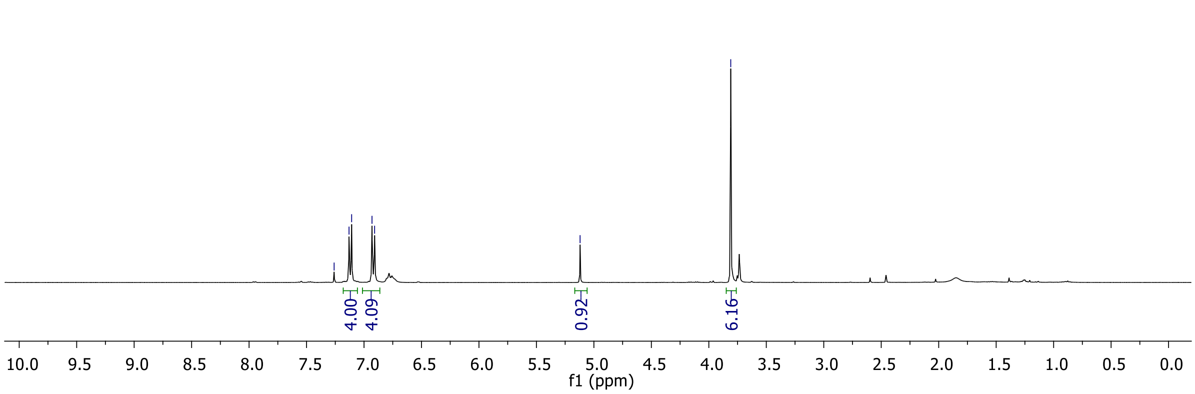


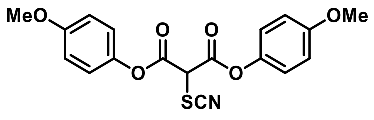

**2r**

$^{13}$C{$^1$H} NMR of **2r** (101 MHz, $CDCl_3$):

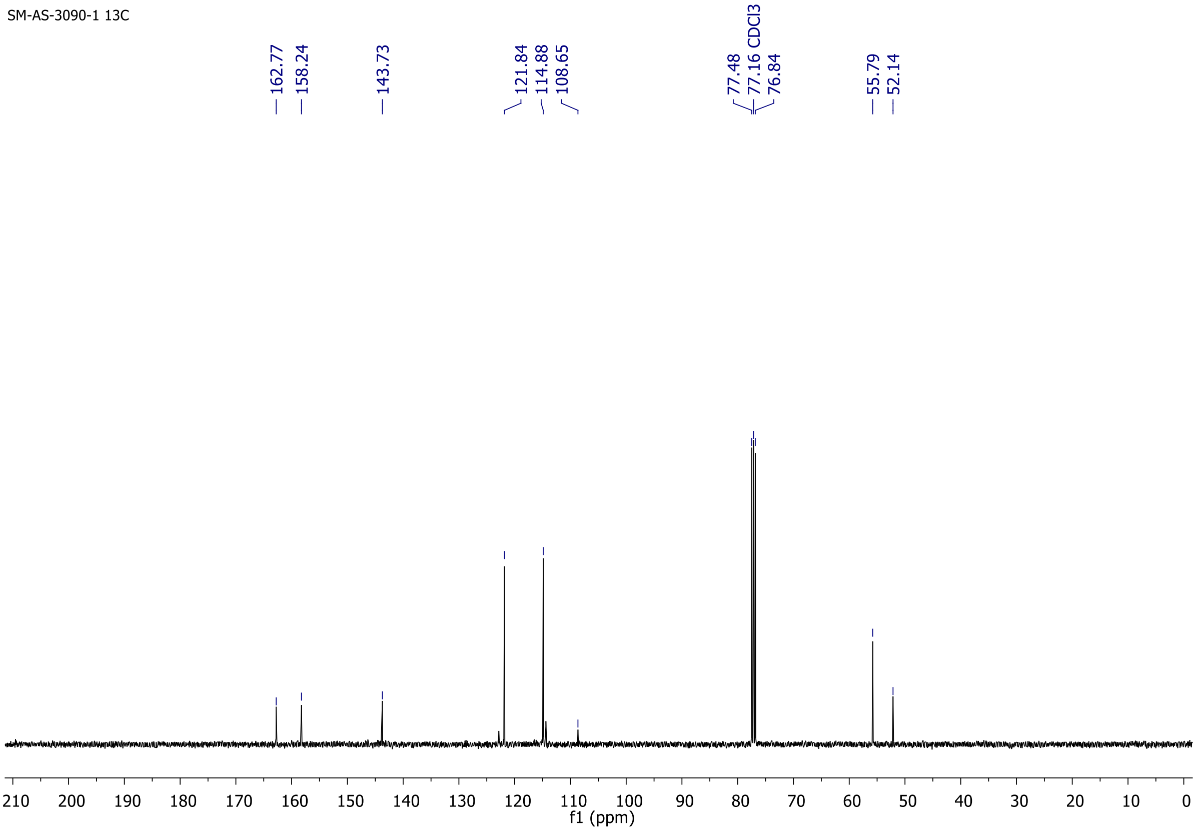

$^{1}$H NMR of **2s** (400 MHz, $CDCl_3$):

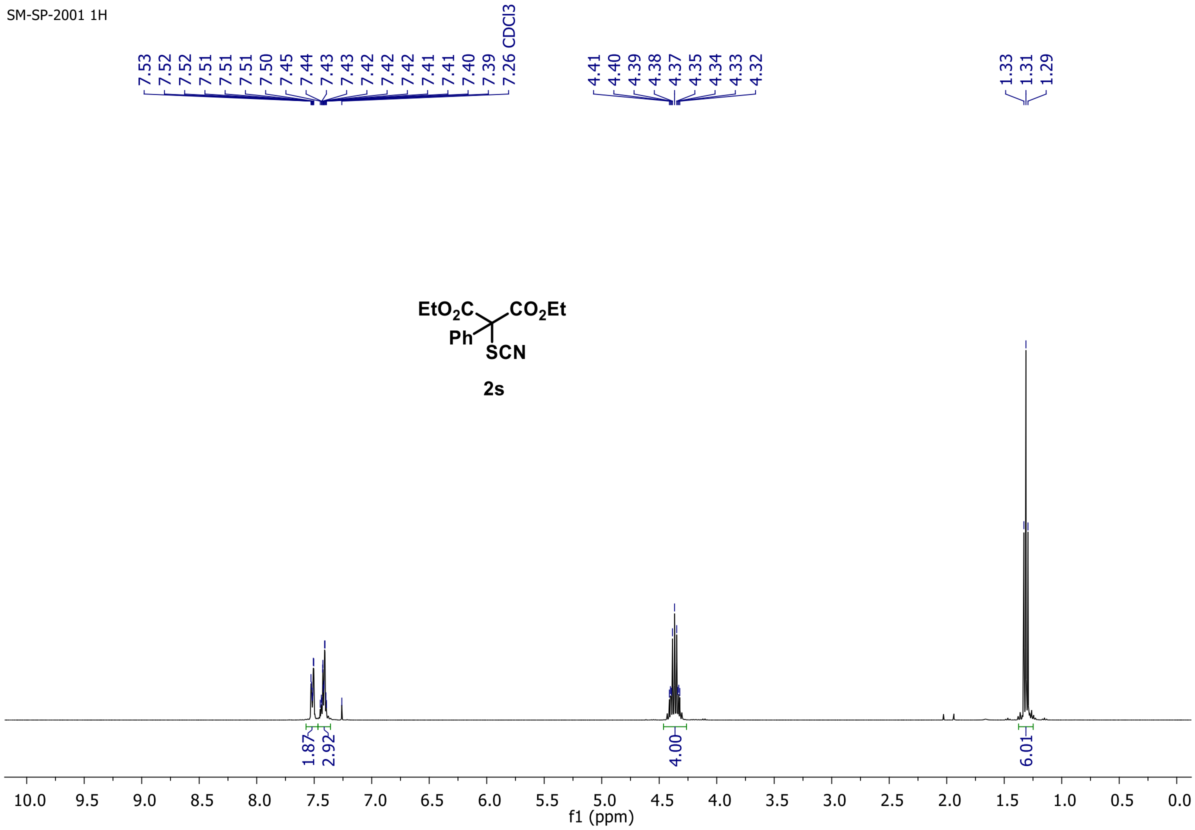


$^{13}$C{$^{1}$H} NMR of **2s** (101 MHz, $CDCl_3$):

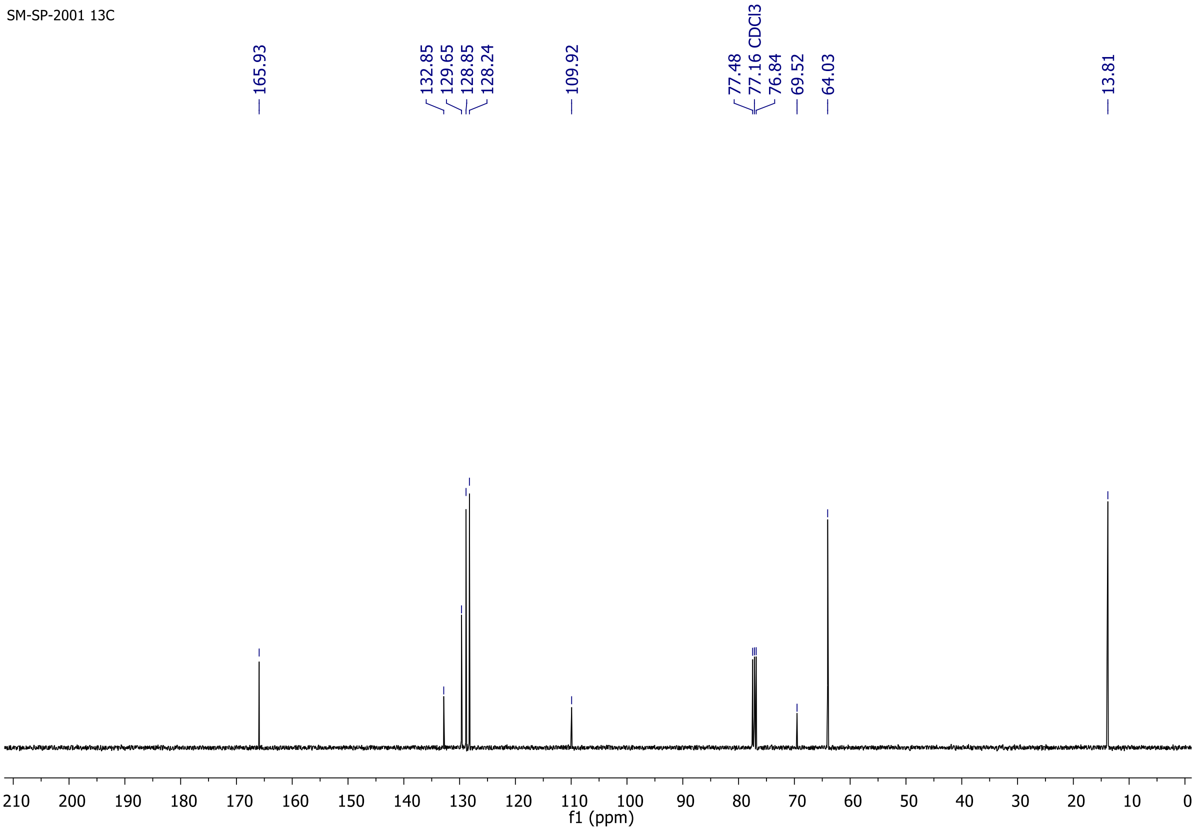

<sup></sup>

$^{1}$H NMR of **2t** (400 MHz, $CDCl_3$):

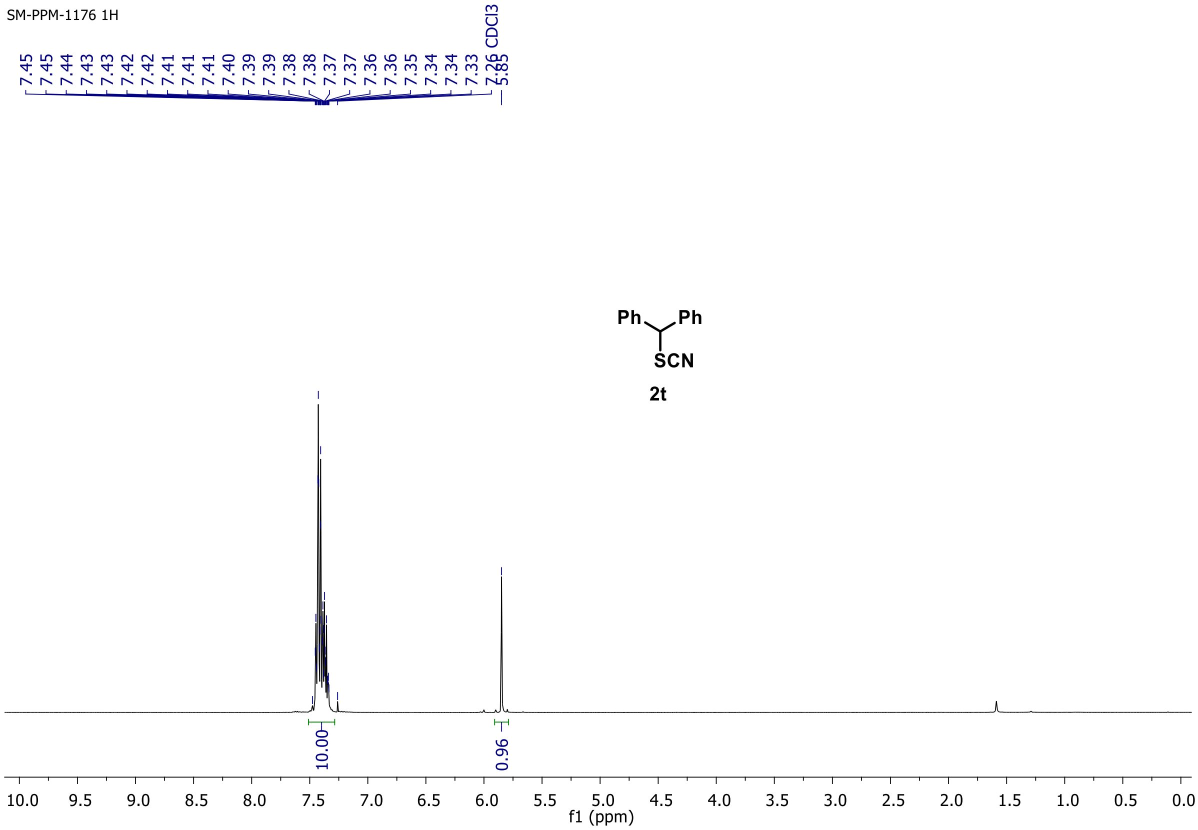


$^{13}$C{$^{1}$H} NMR of **2t** (101 MHz, $CDCl_3$):

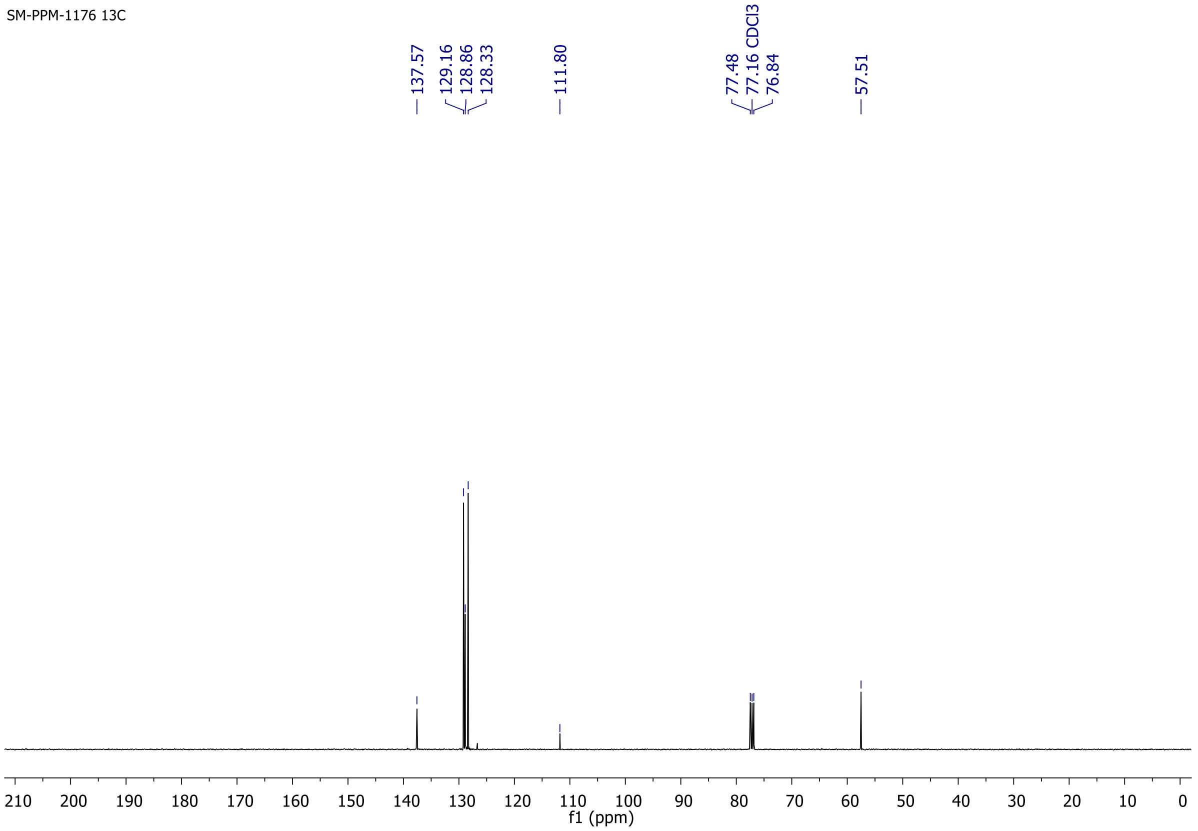

$^1H$ NMR of **2u** (400 MHz, $CDCl_3$):

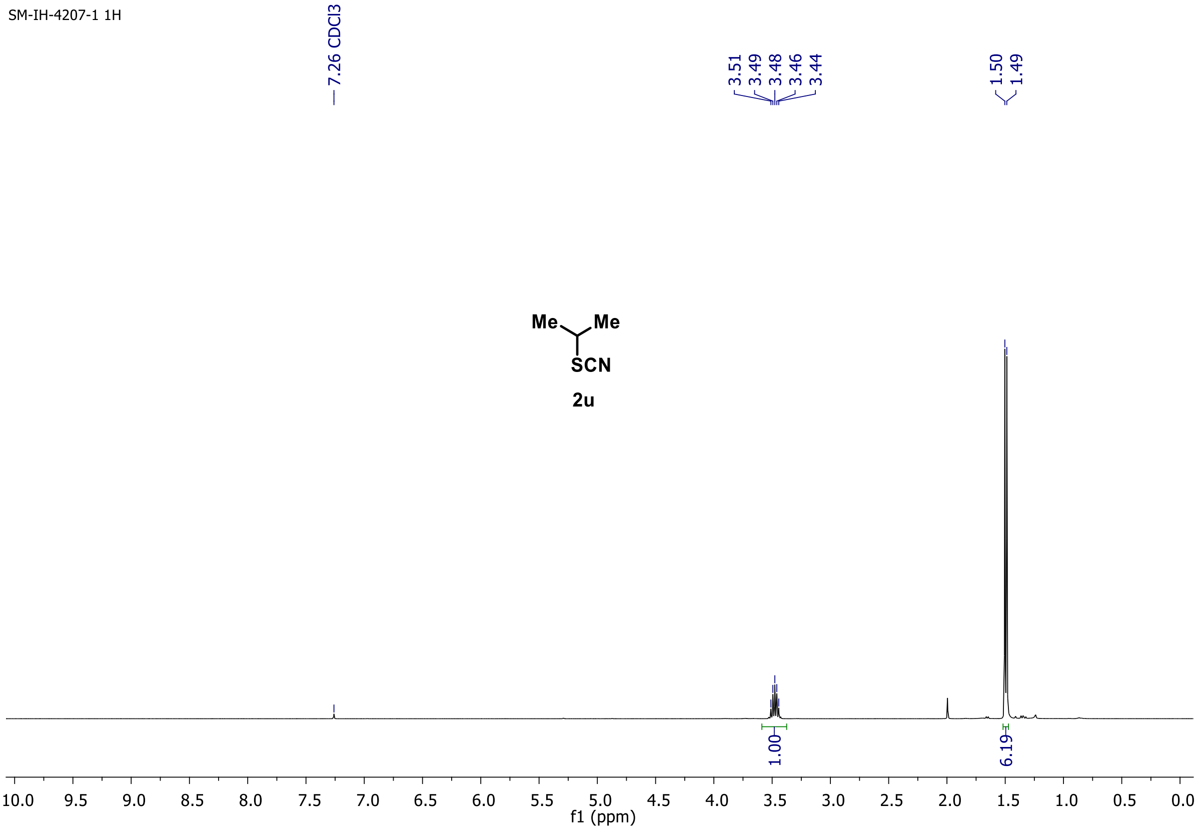


$^{13}C\{^1H\}$ NMR of **2u** (101 MHz, $CDCl_3$):

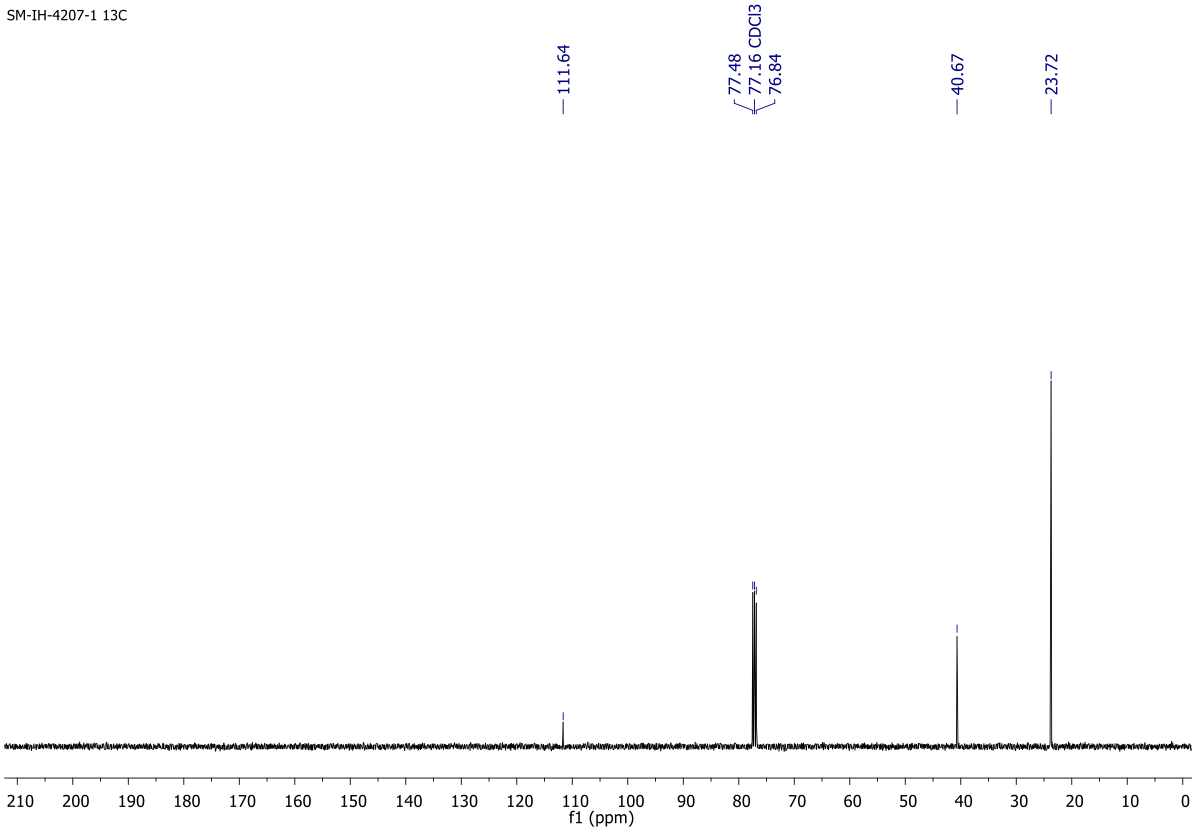

$^{1}$H NMR of **3a’** (400 MHz, $CDCl_3$):

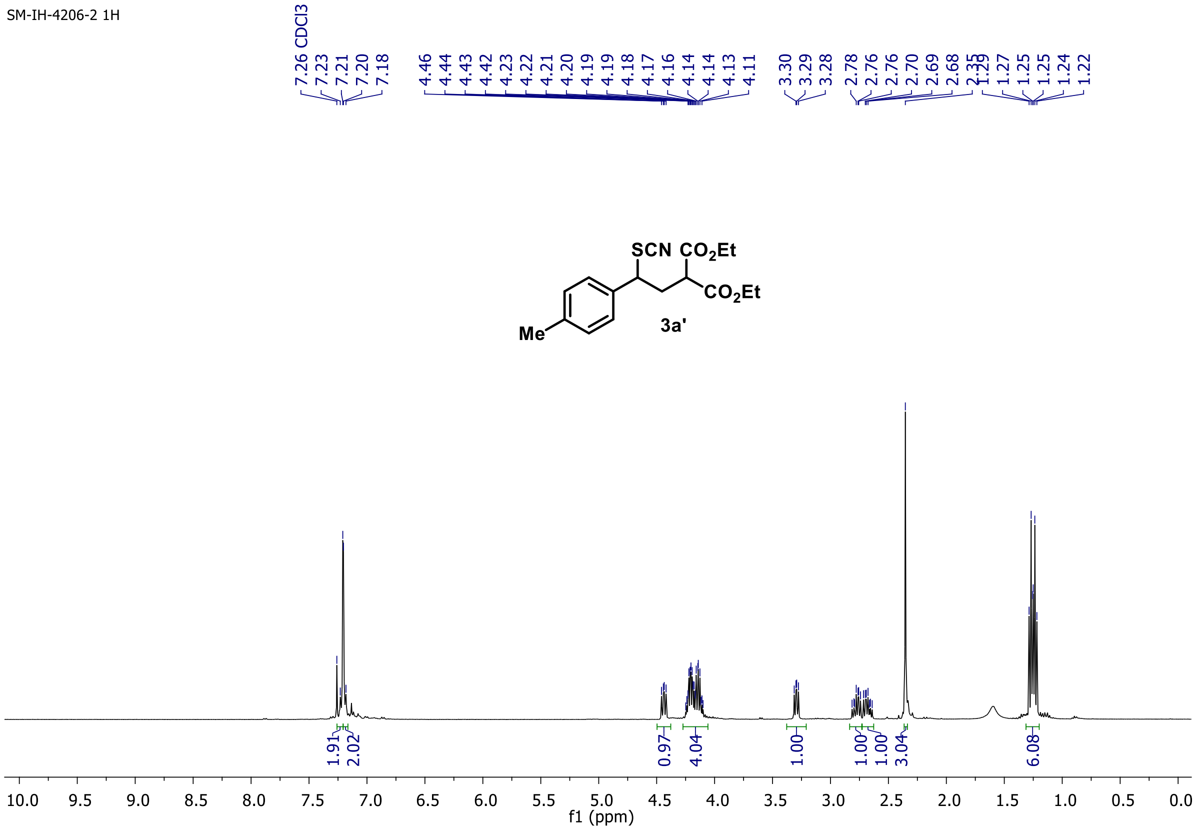


$^{13}$C{$^{1}$H} NMR of **3a’** (101 MHz, $CDCl_3$):

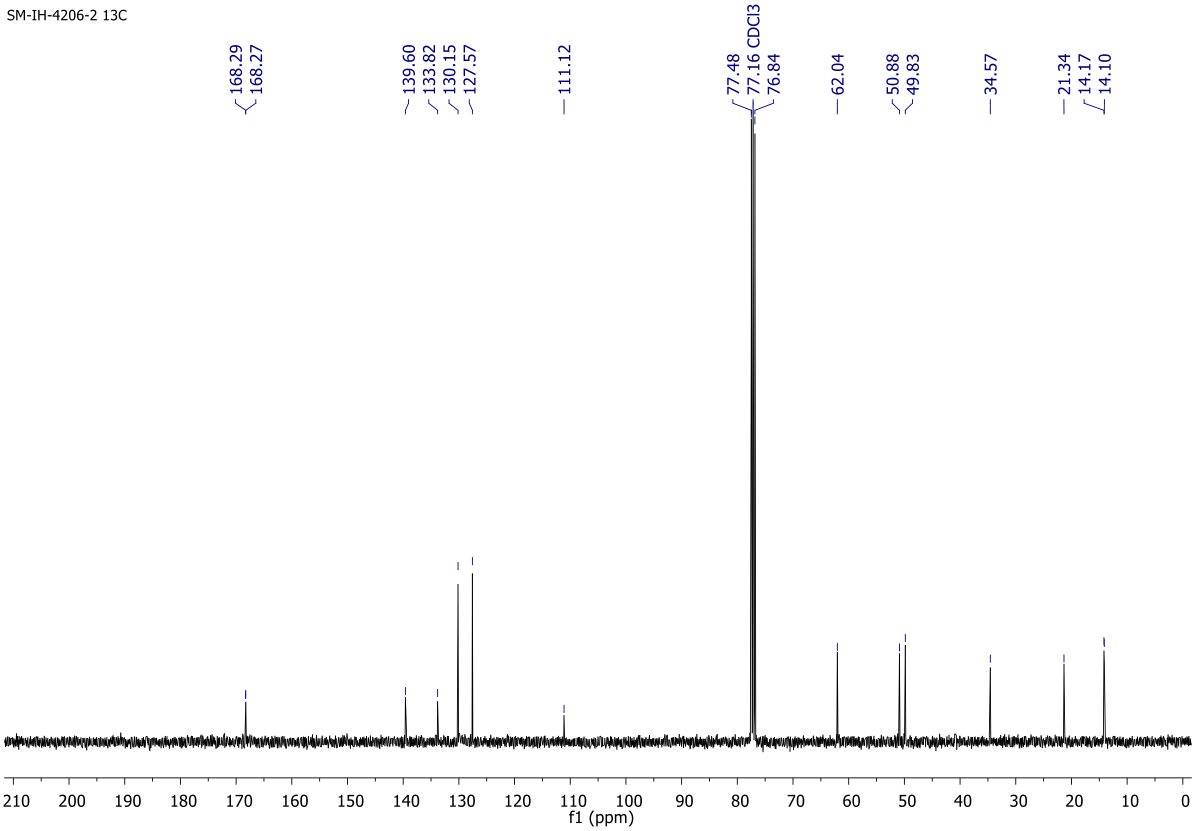

$^{1}H$ NMR of **3a** (400 MHz, $CDCl_3$):

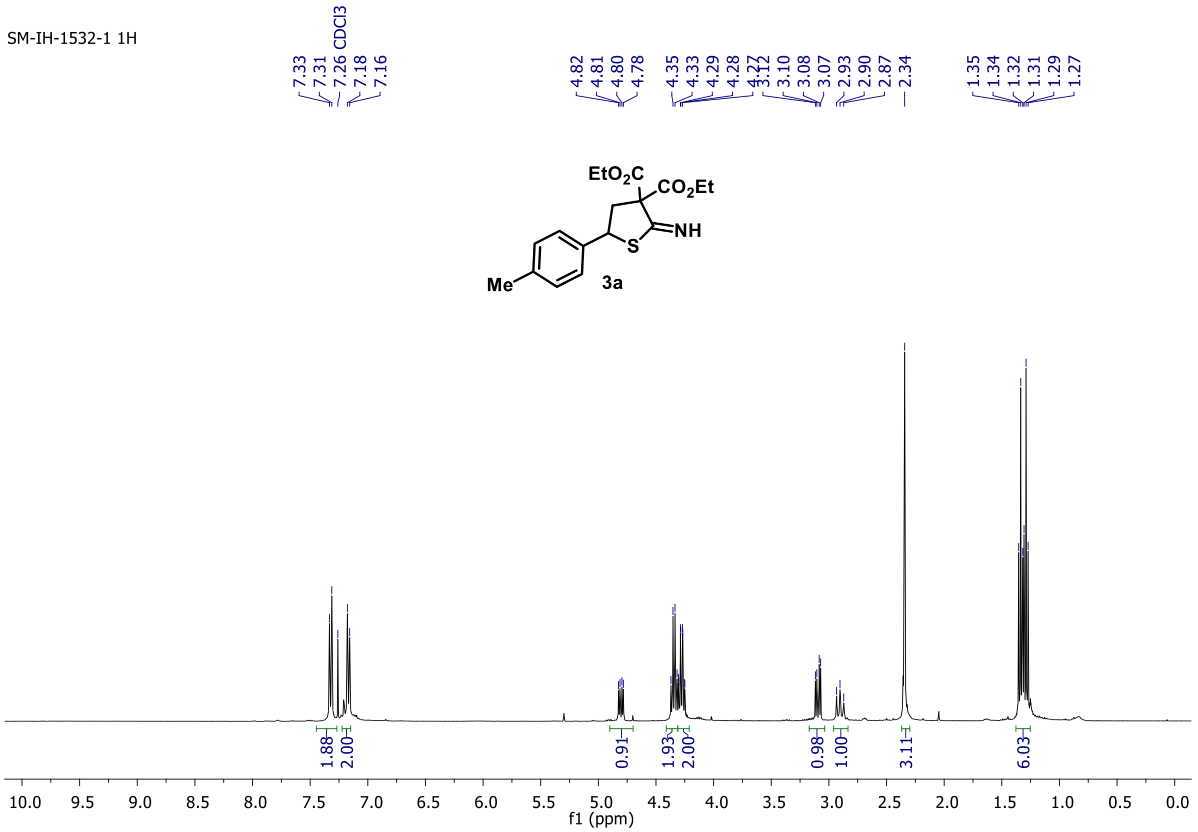


$^{13}C\{^{1}H\}$ NMR of **3a** (101 MHz, $CDCl_3$):

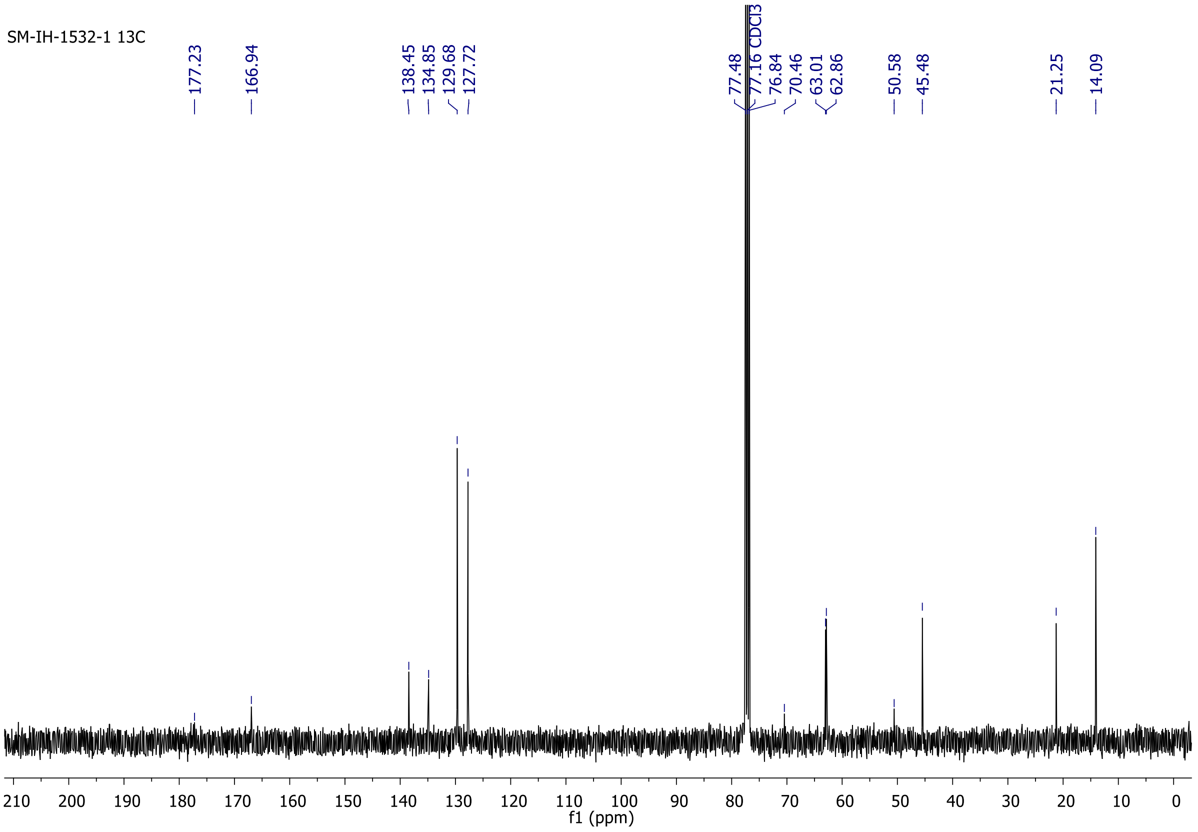

$^1$H NMR of **3b** (400 MHz, $CDCl_3$):

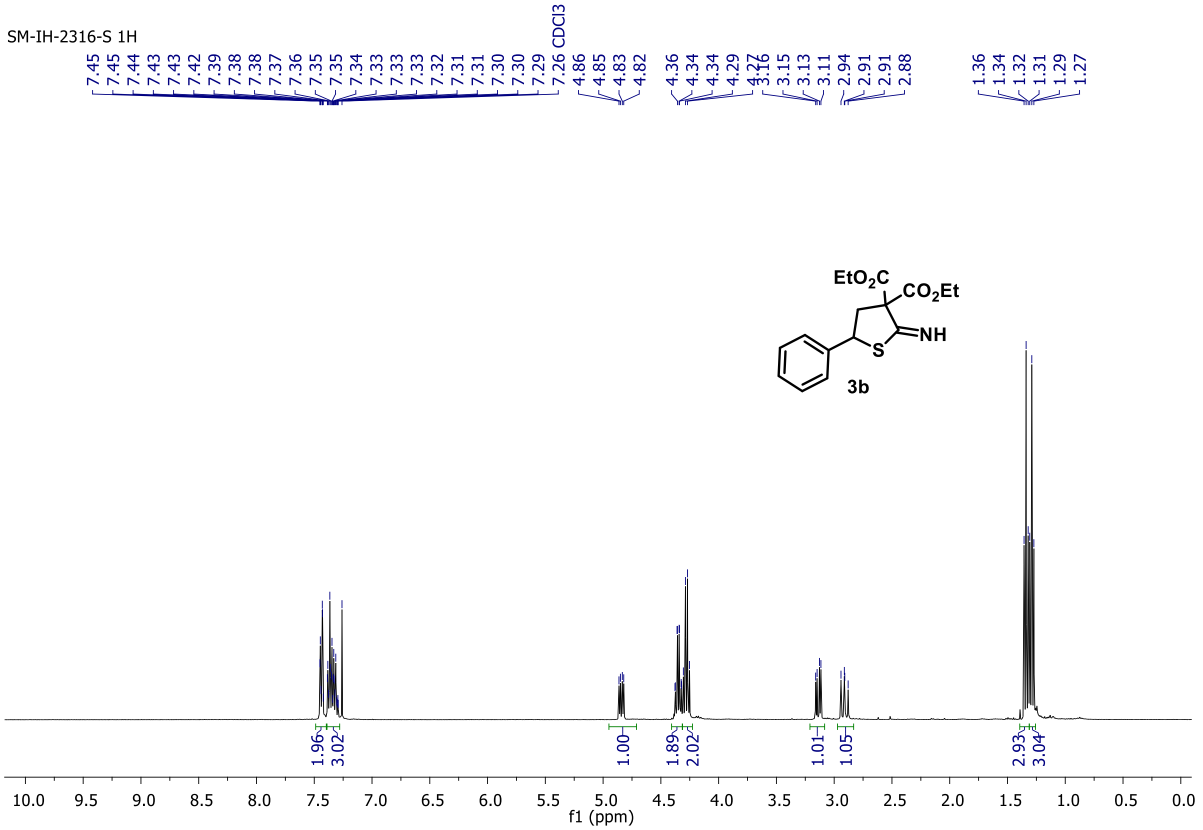


$^{13}C\{^1H\}$ NMR of **3b** (101 MHz, $CDCl_3$):

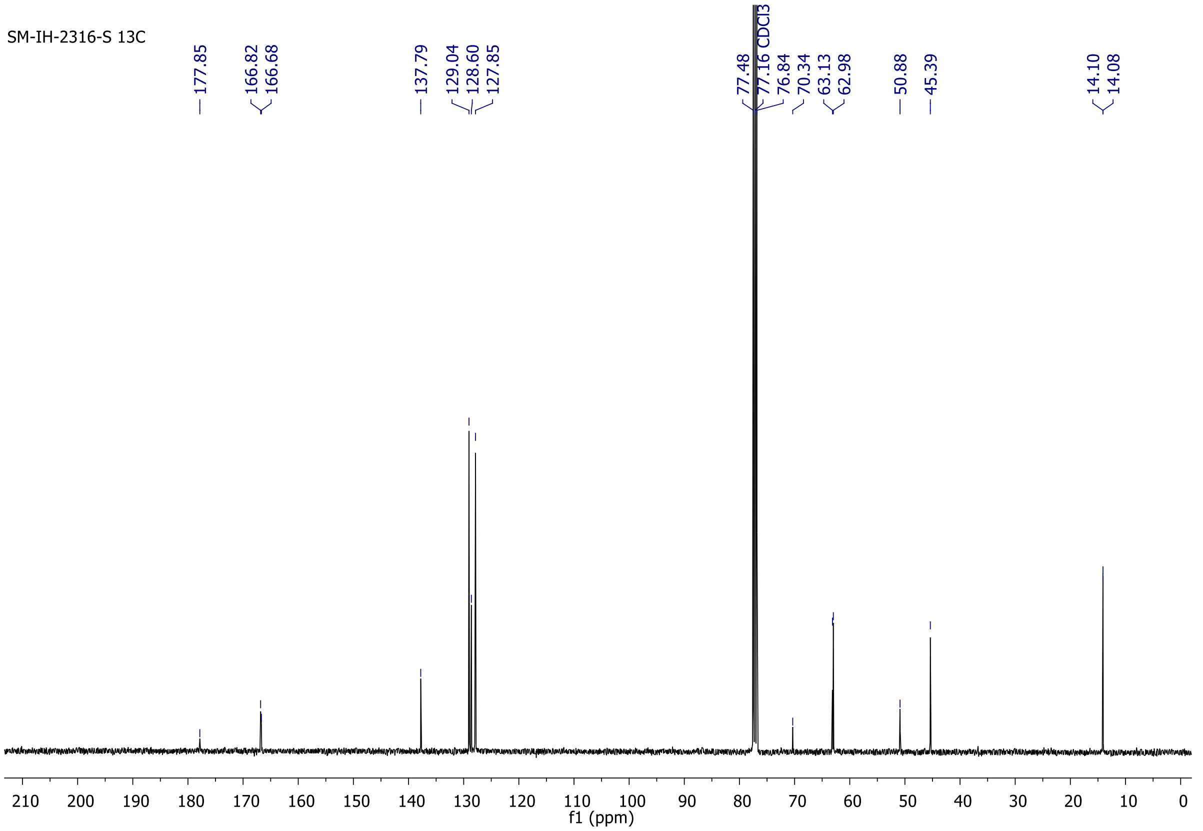

$^{1}H$ NMR of **3c** (400 MHz, $CDCl_3$):

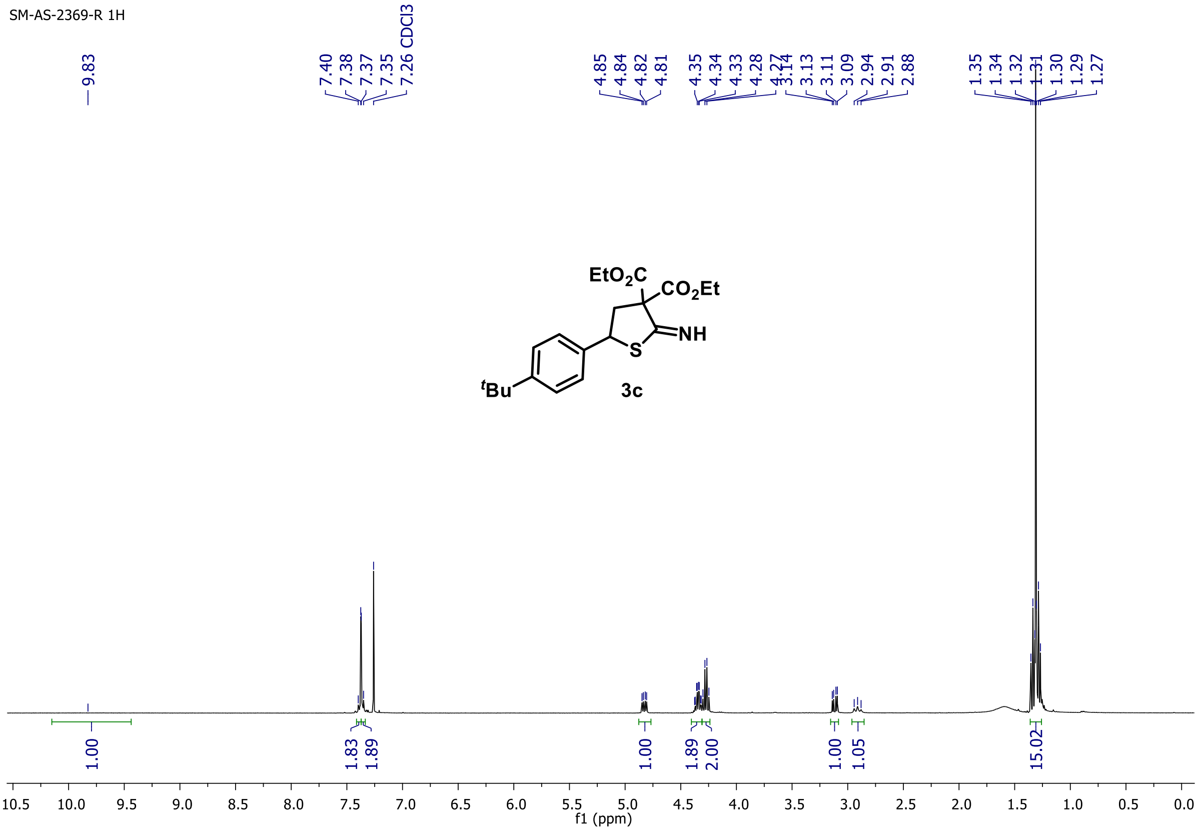


$^{13}C\{^{1}H\}$ NMR of **3c** (101 MHz, $CDCl_3$):

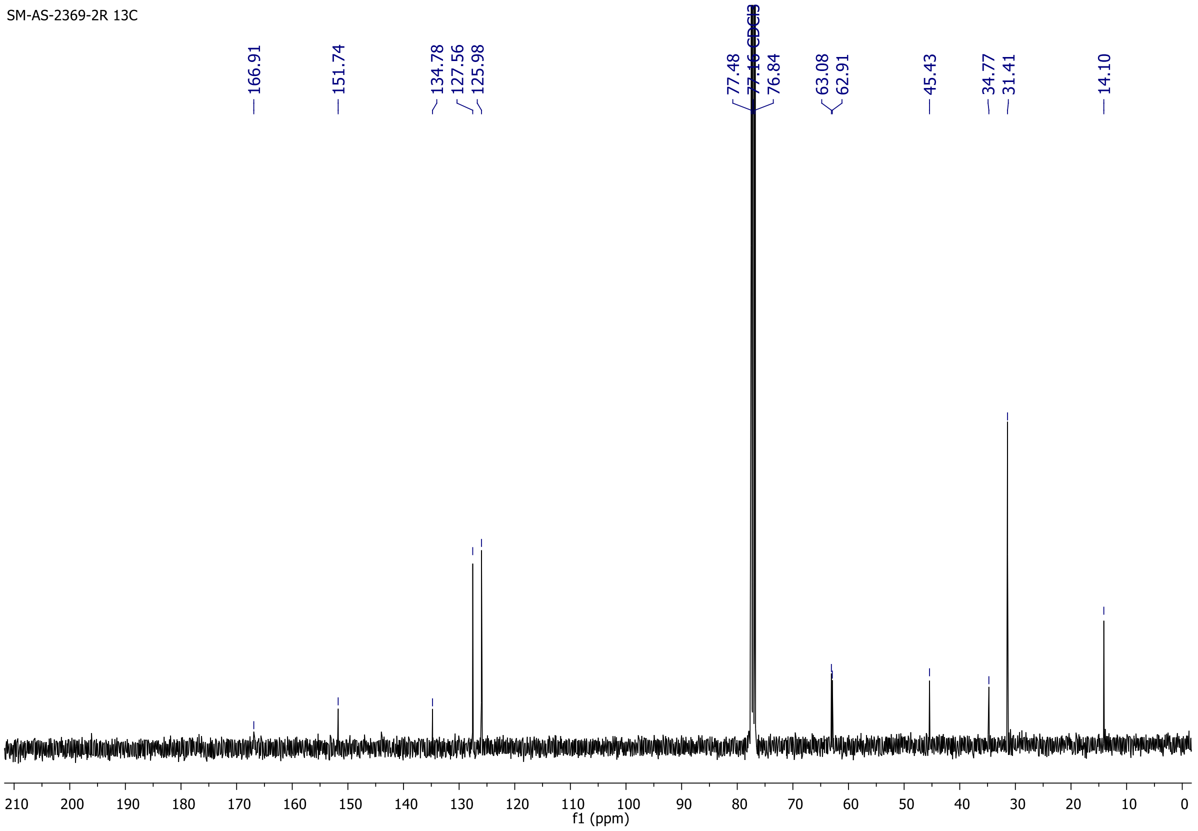

$^{1}H$ NMR of **3d** (400 MHz, DMSO-$d_6$):

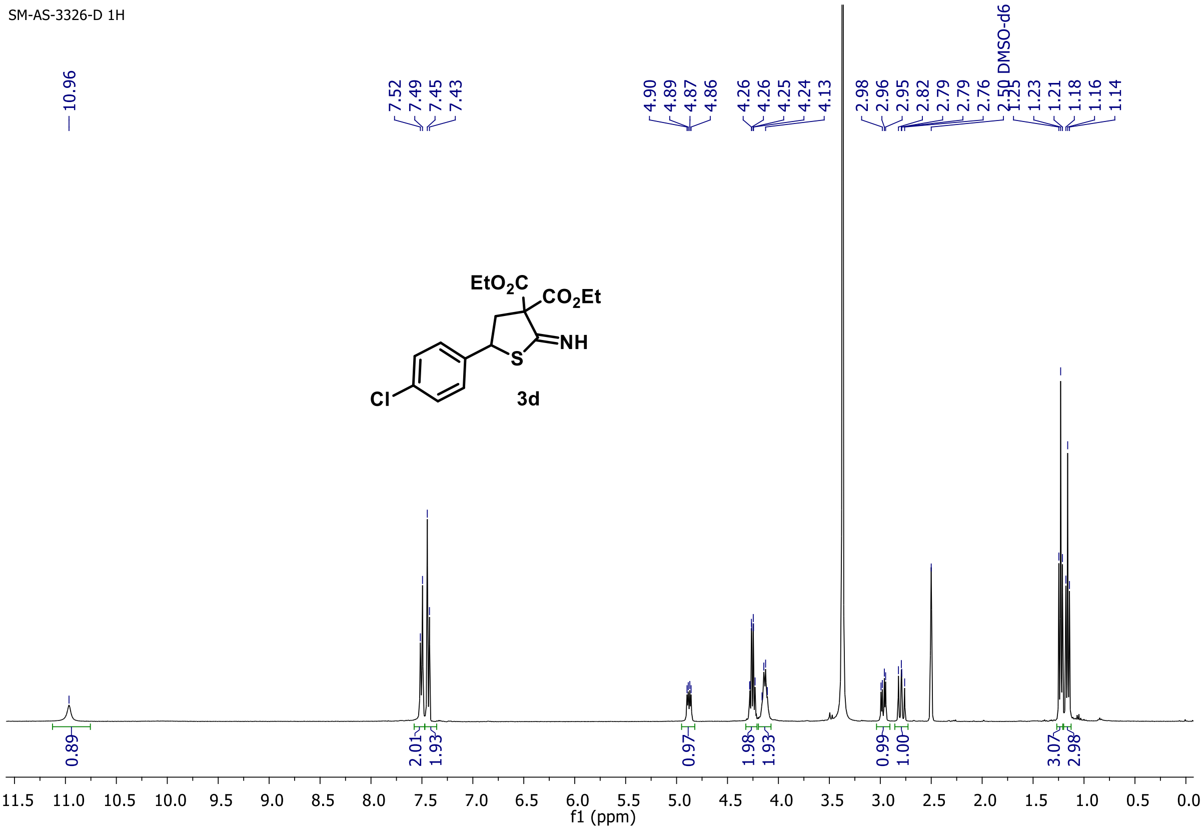


$^{13}C\{^{1}H\}$ NMR of **3d** (101 MHz, DMSO-$d_6$):

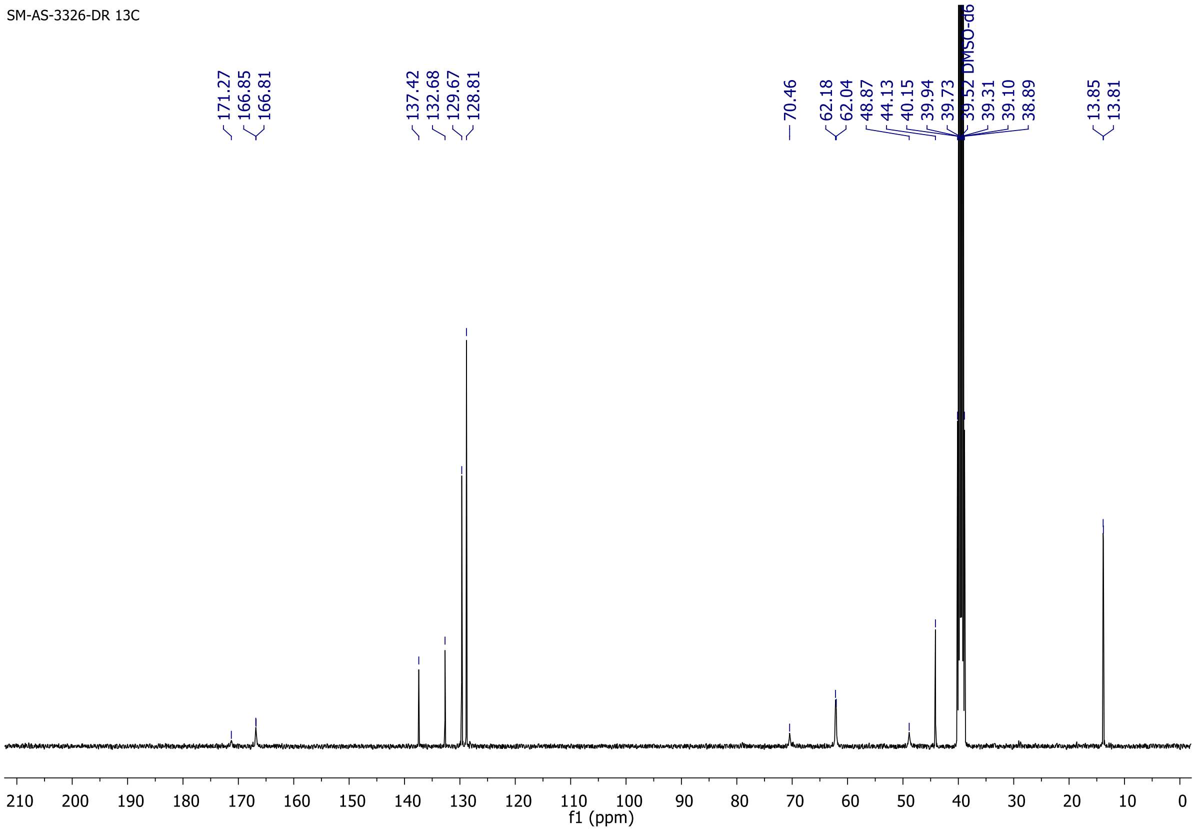

$^{1}H$ NMR of **3e** (400 MHz, DMSO-d$_6$):

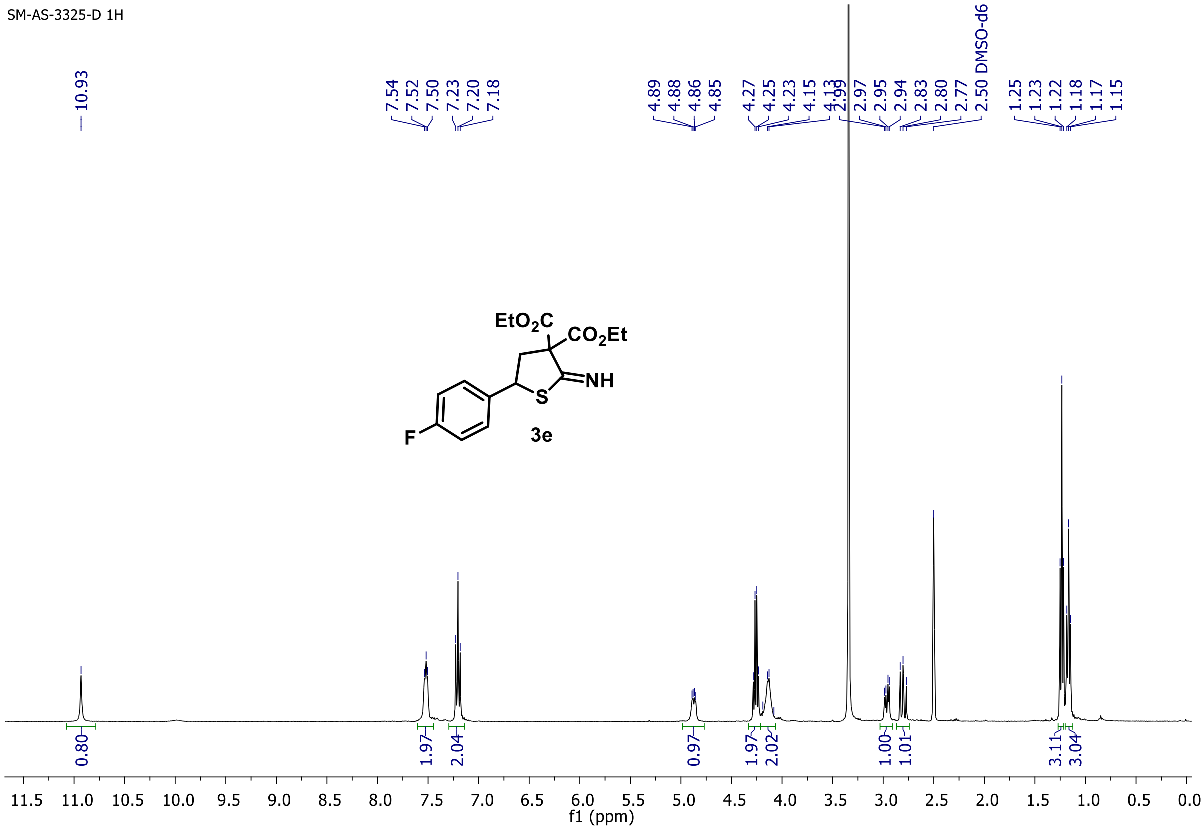


$^{13}C\{^{1}H\}$ NMR of **3e** (101 MHz, DMSO-d$_6$):

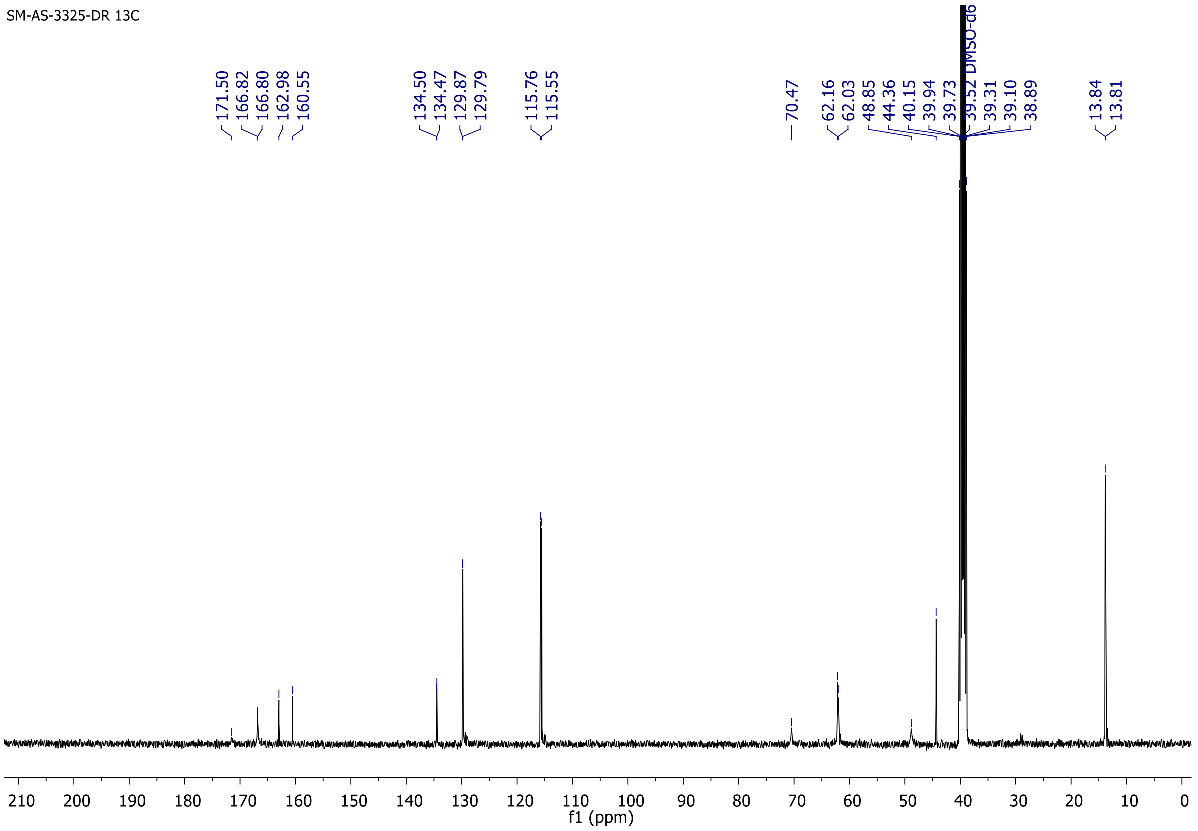

$^{19}F$ NMR of **3e** (377 MHz, DMSO-d$_6$):

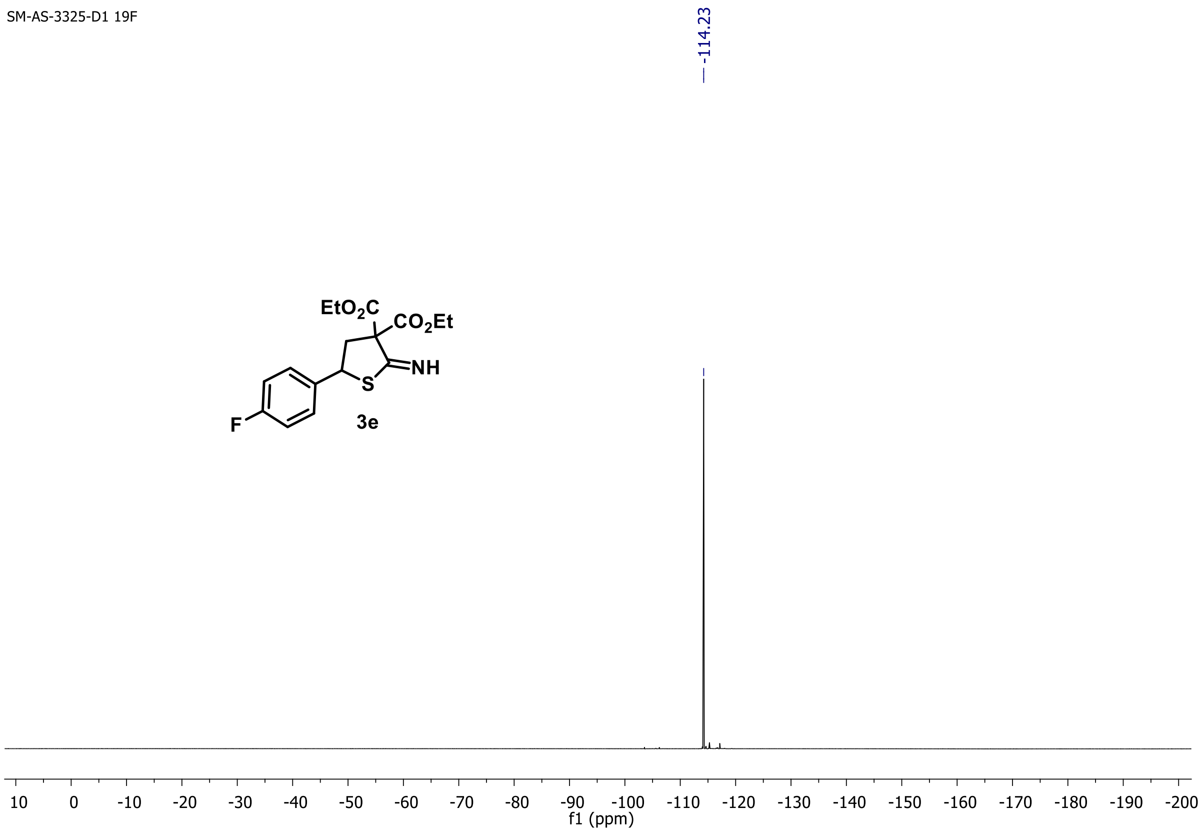

$^{1}H$ NMR of **3f** (400 MHz, $CDCl_3$):

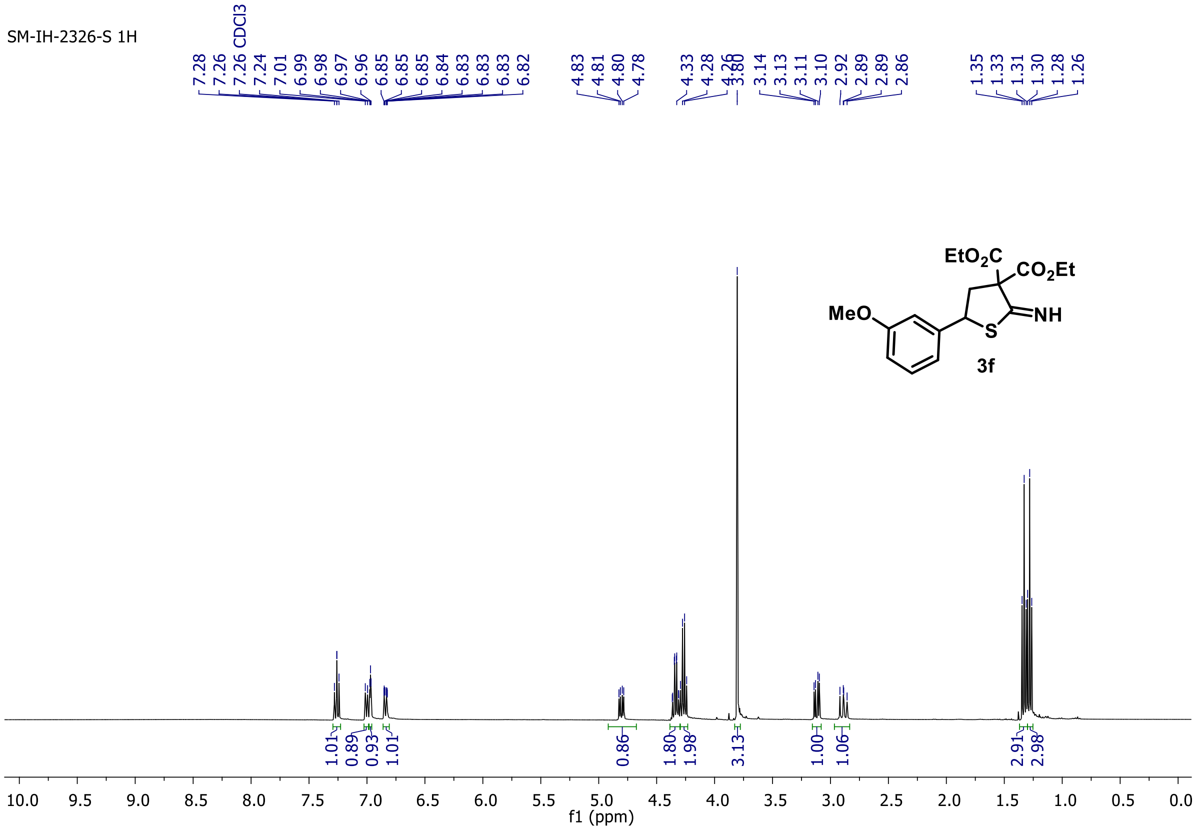

$^{13}C\{^{1}H\}$ NMR of **3f** (101 MHz, $CDCl_3$):

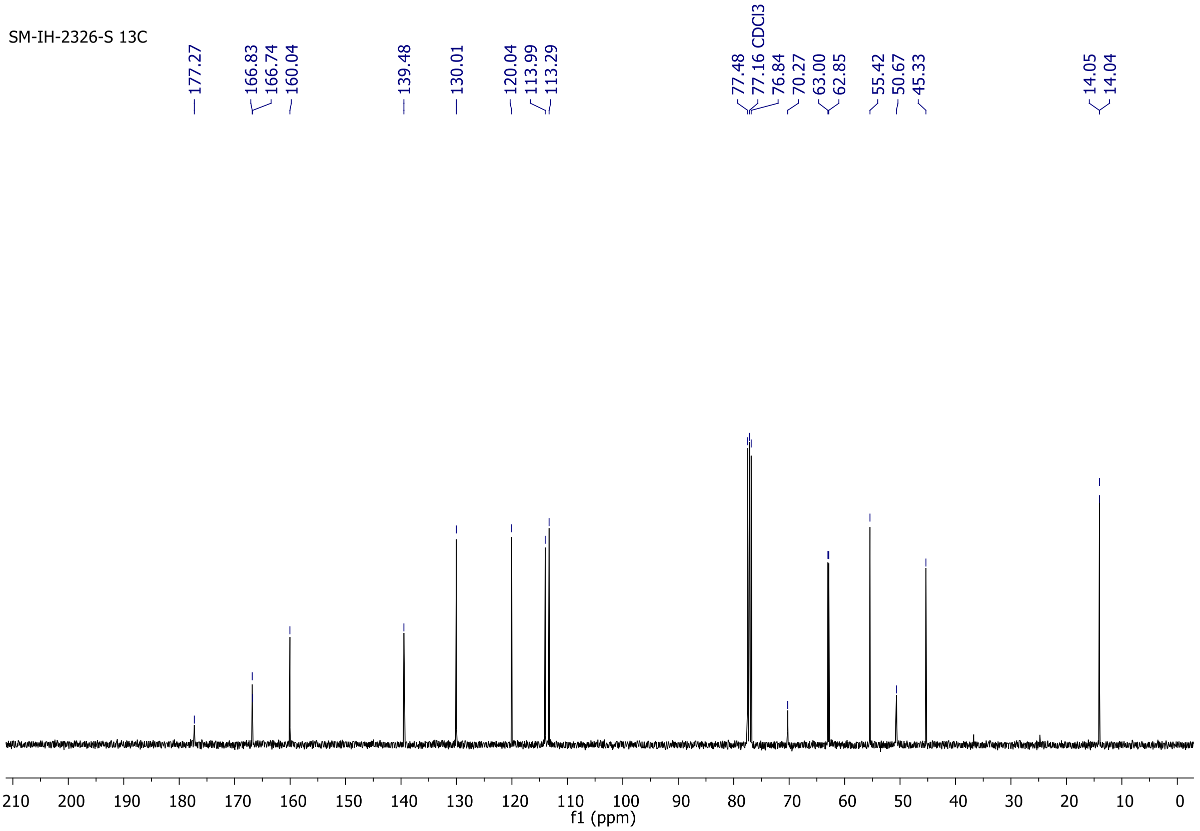

$^1$H NMR of **3g** (400 MHz, $CDCl_3$):

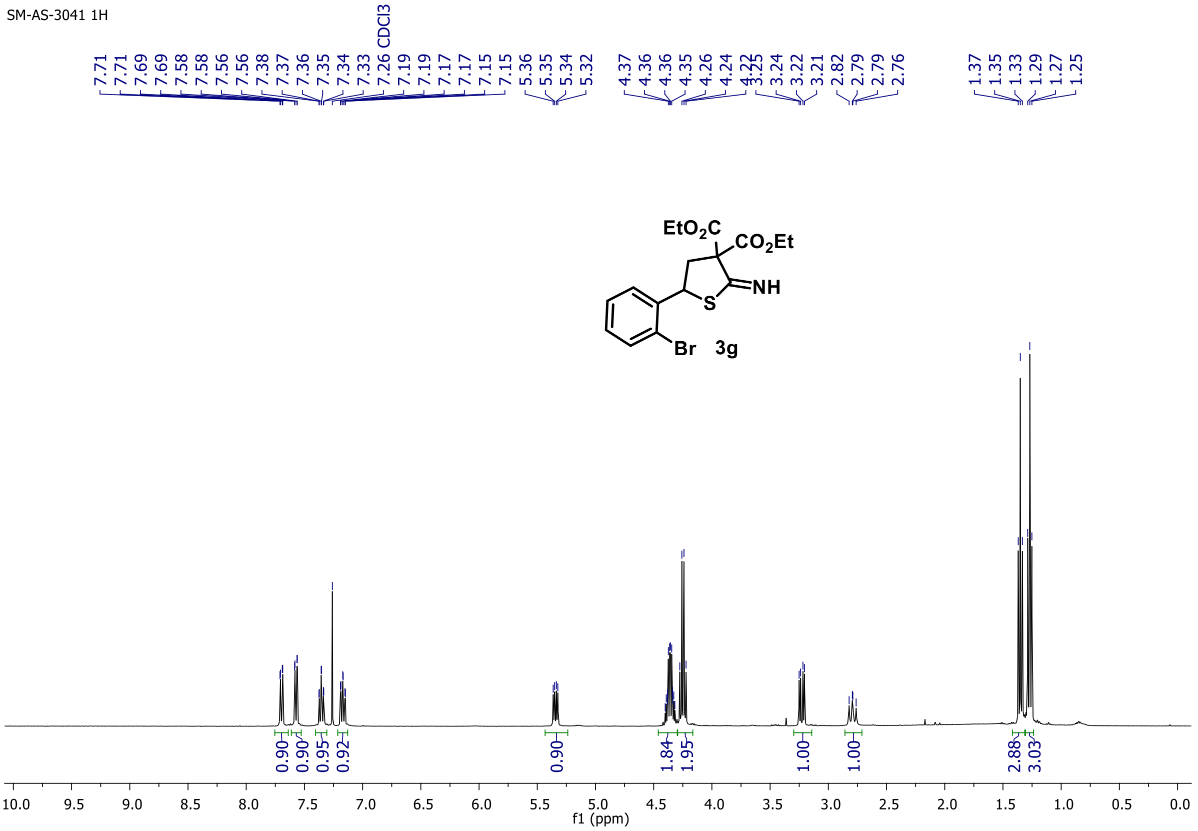


$^{13}C\{^1H\}$ NMR of **3g** (101 MHz, $CDCl_3$):

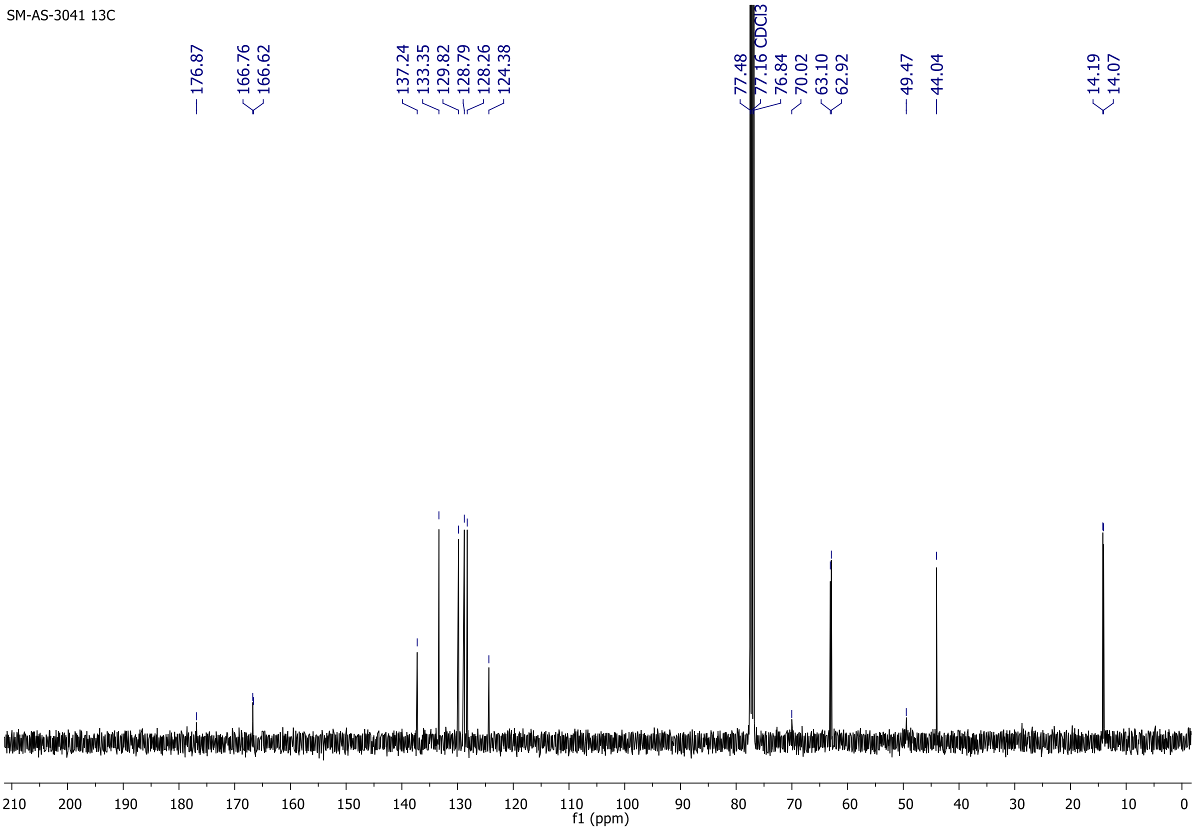

$^{1}$H NMR of **3h** (400 MHz, $CDCl_3$):

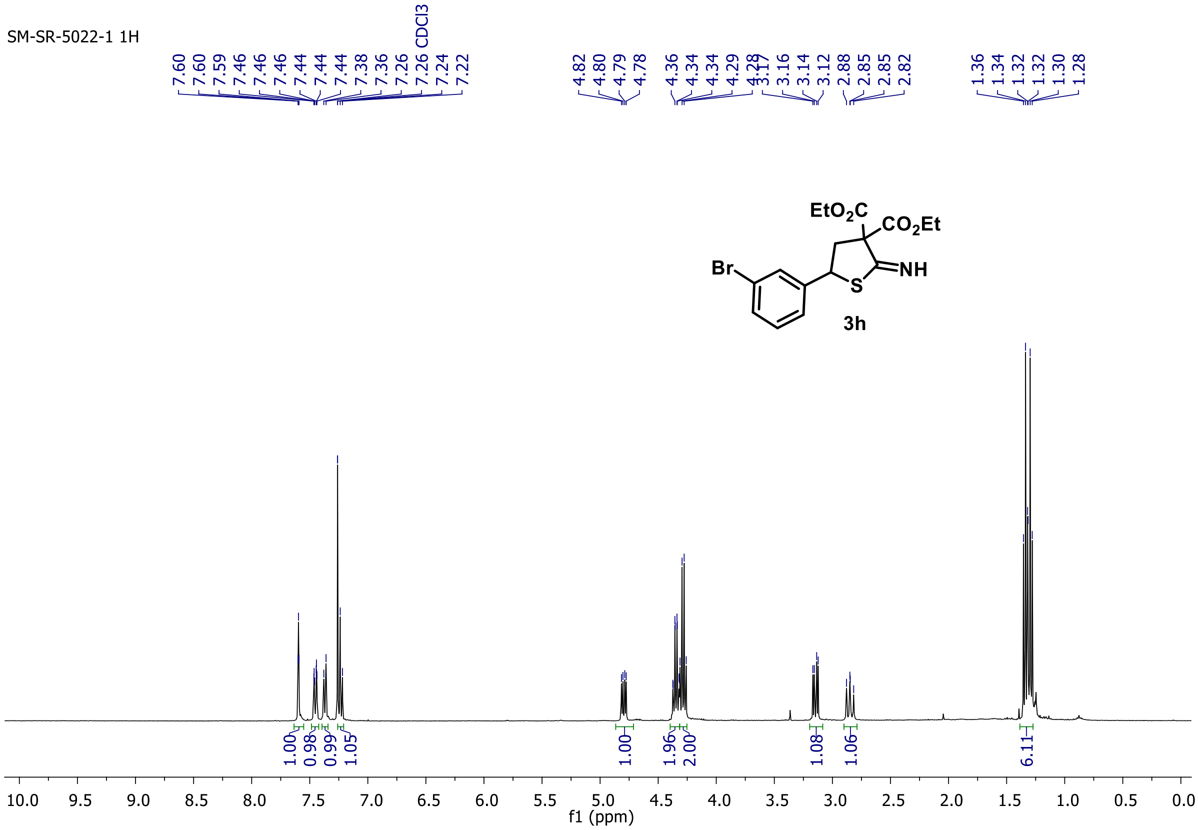


$^{13}$C{$^{1}$H} NMR of **3h** (101 MHz, $CDCl_3$):

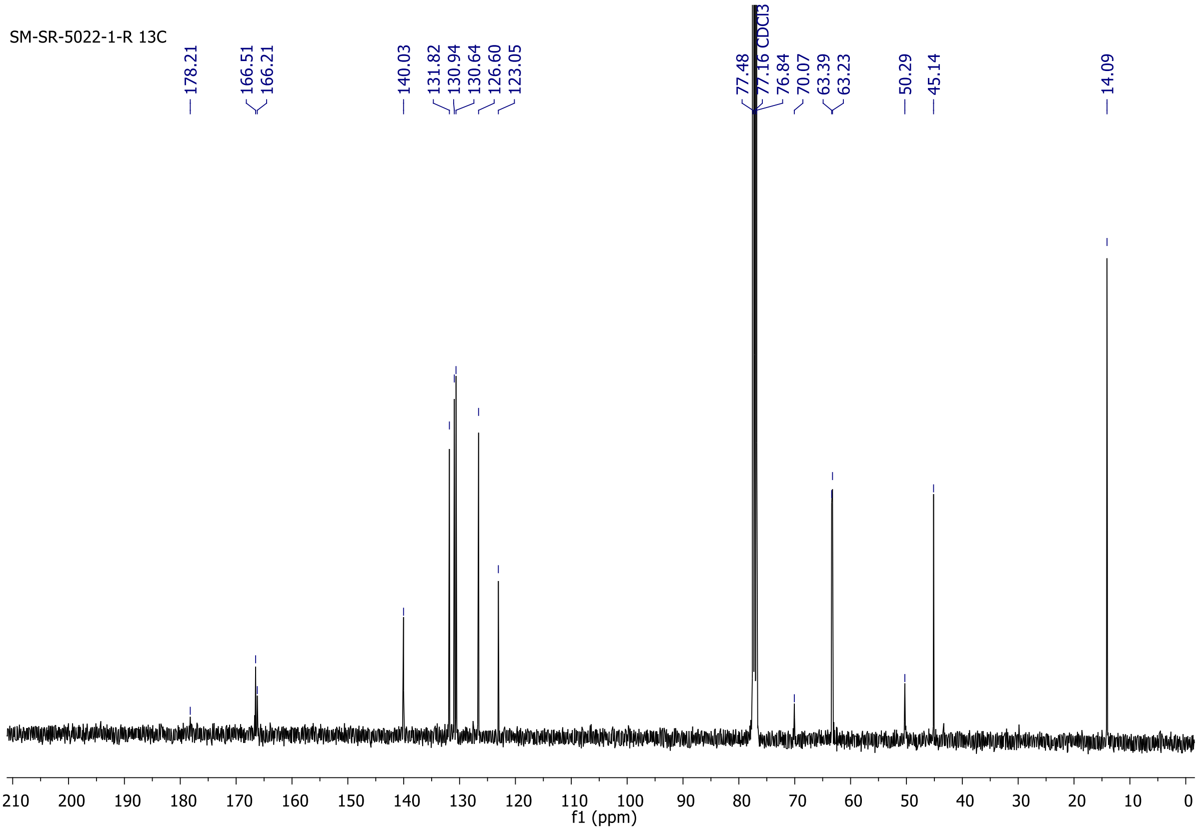

$^{1}H$ NMR of **3i** (400 MHz, $CDCl_3$):

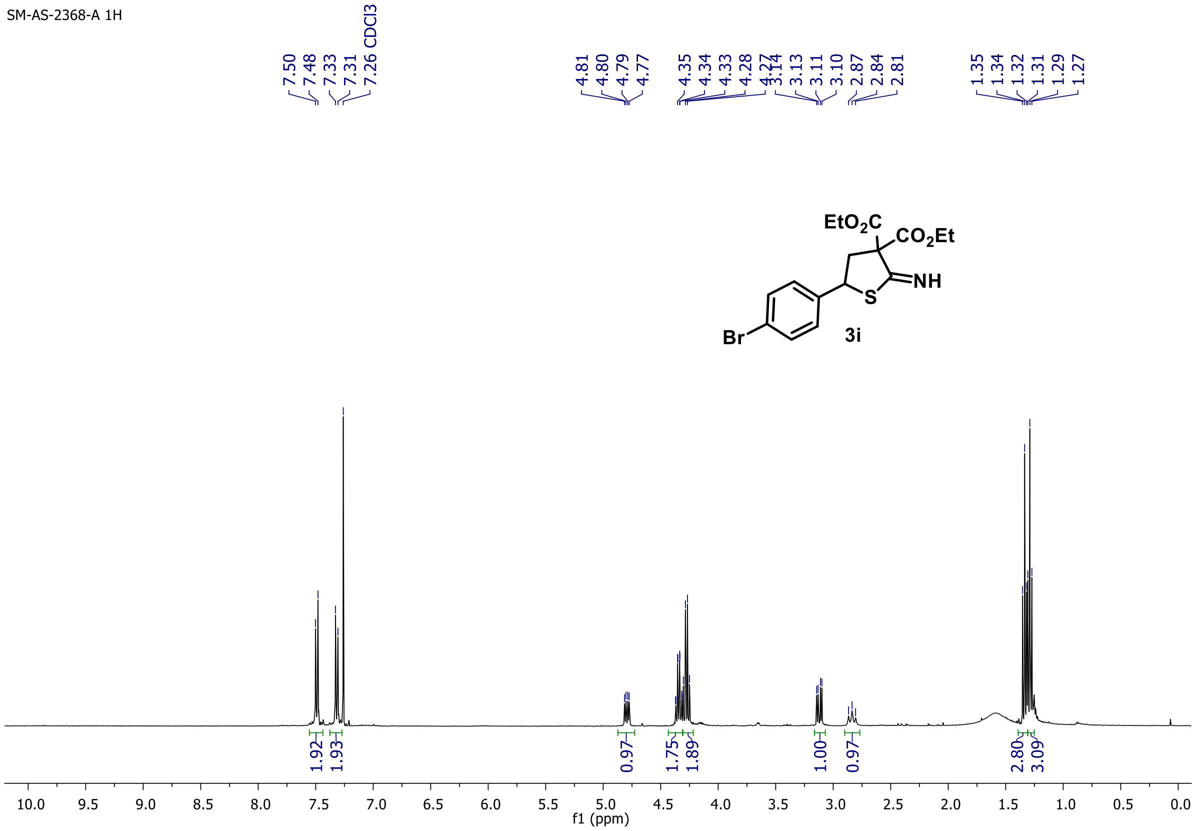


$^{13}C\{^{1}H\}$ NMR of **3i** (101 MHz, $CDCl_3$):

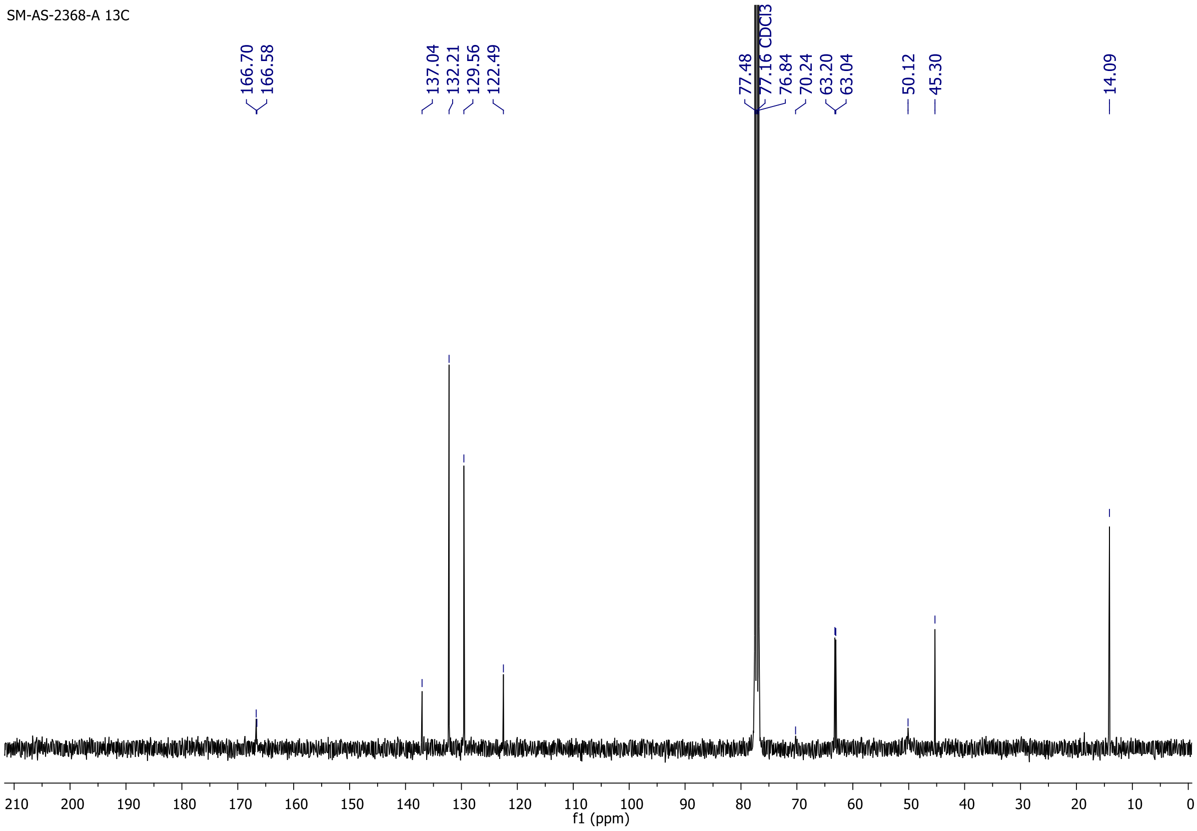

$^{1}H$ NMR of **3j** (400 MHz, $CDCl_3$):

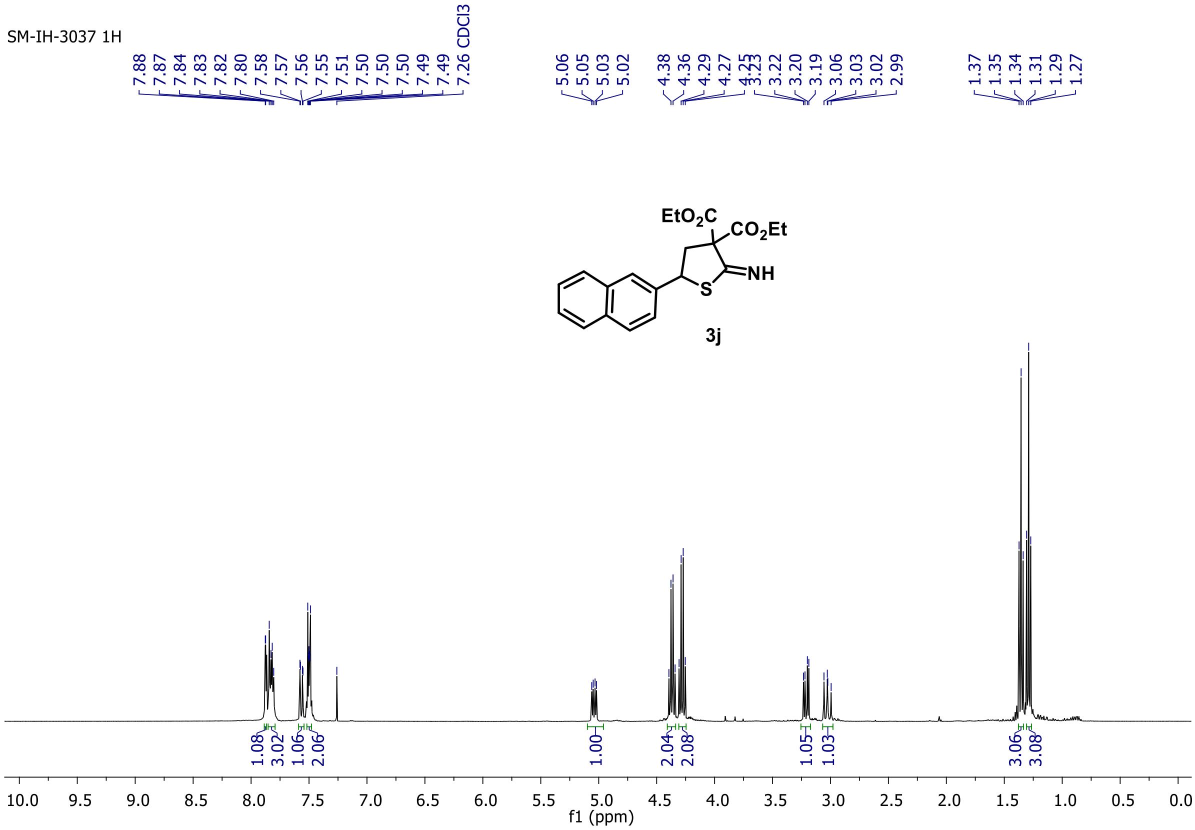


$^{13}C\{^{1}H\}$ NMR of **3j** (101 MHz, $CDCl_3$):

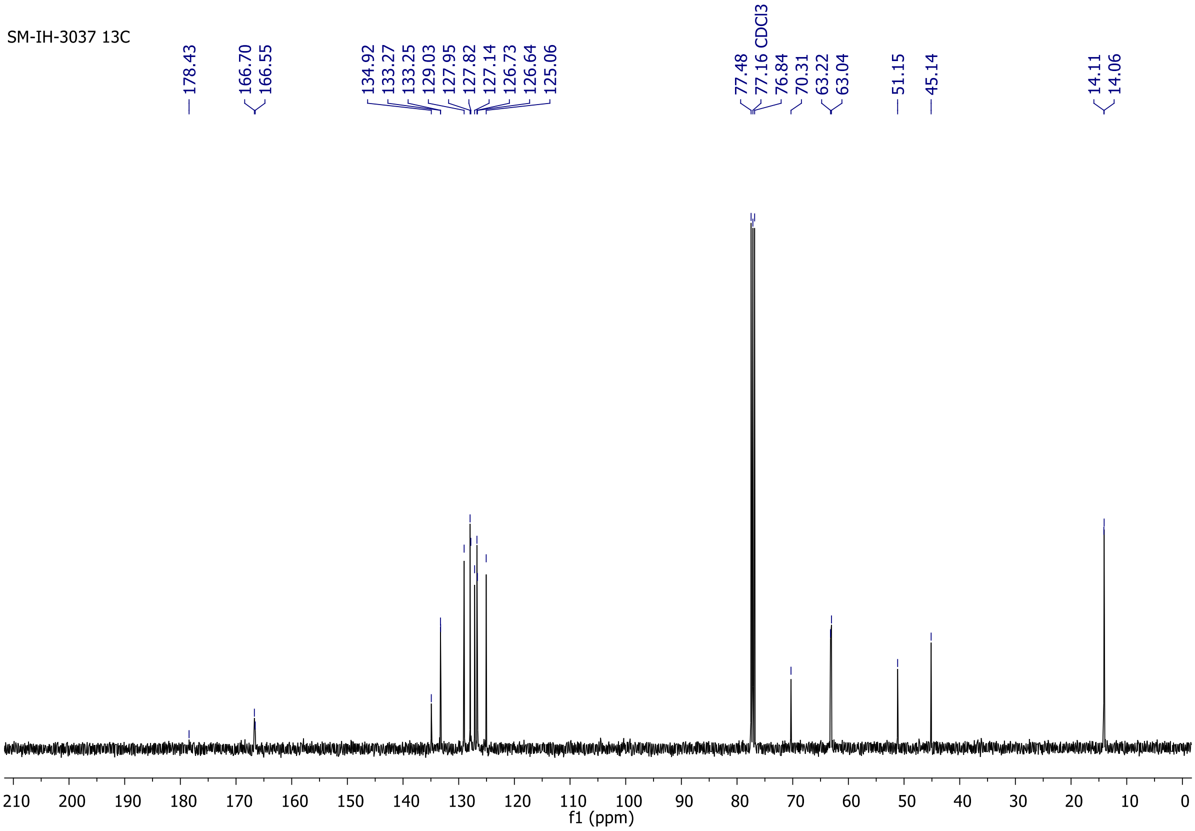

$^{1}$H NMR of **3k** (400 MHz, DMSO-d$_6$):

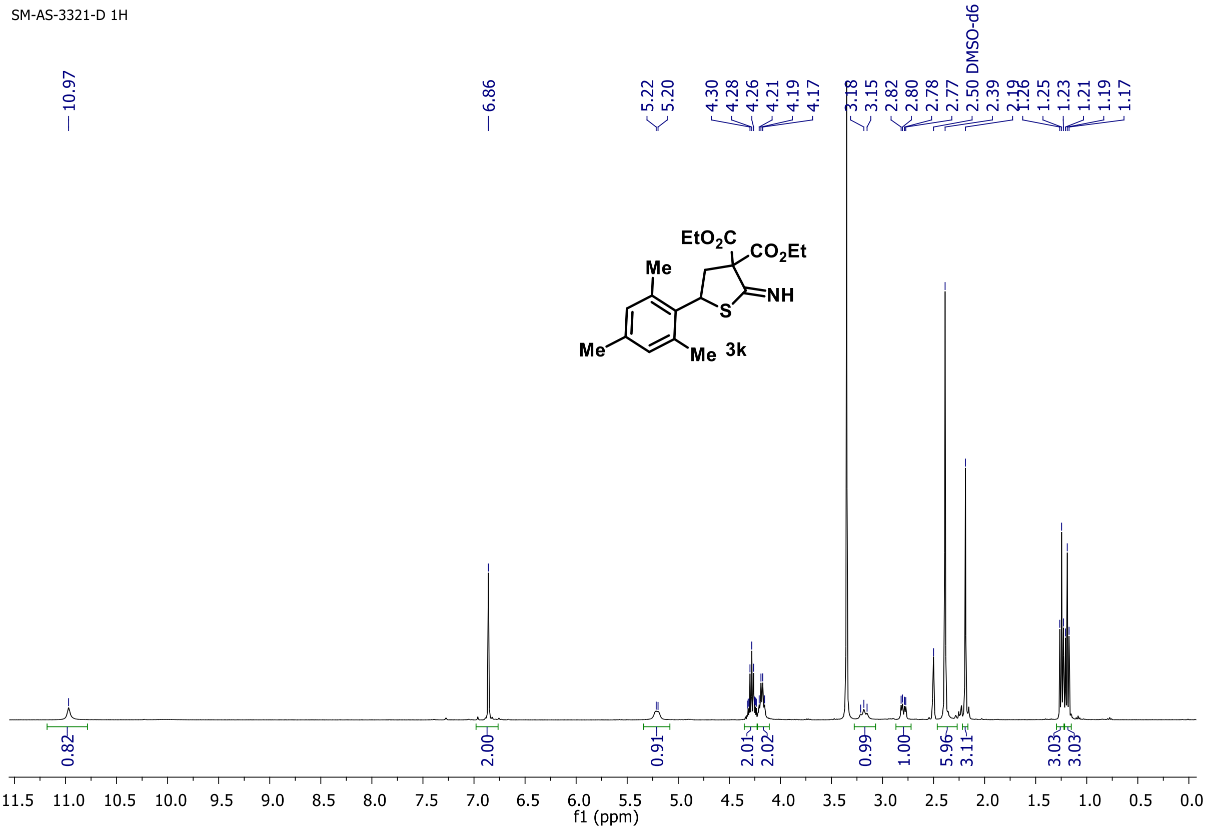


$^{13}$C{$^{1}$H} NMR of **3k** (101 MHz, DMSO-d$_6$):

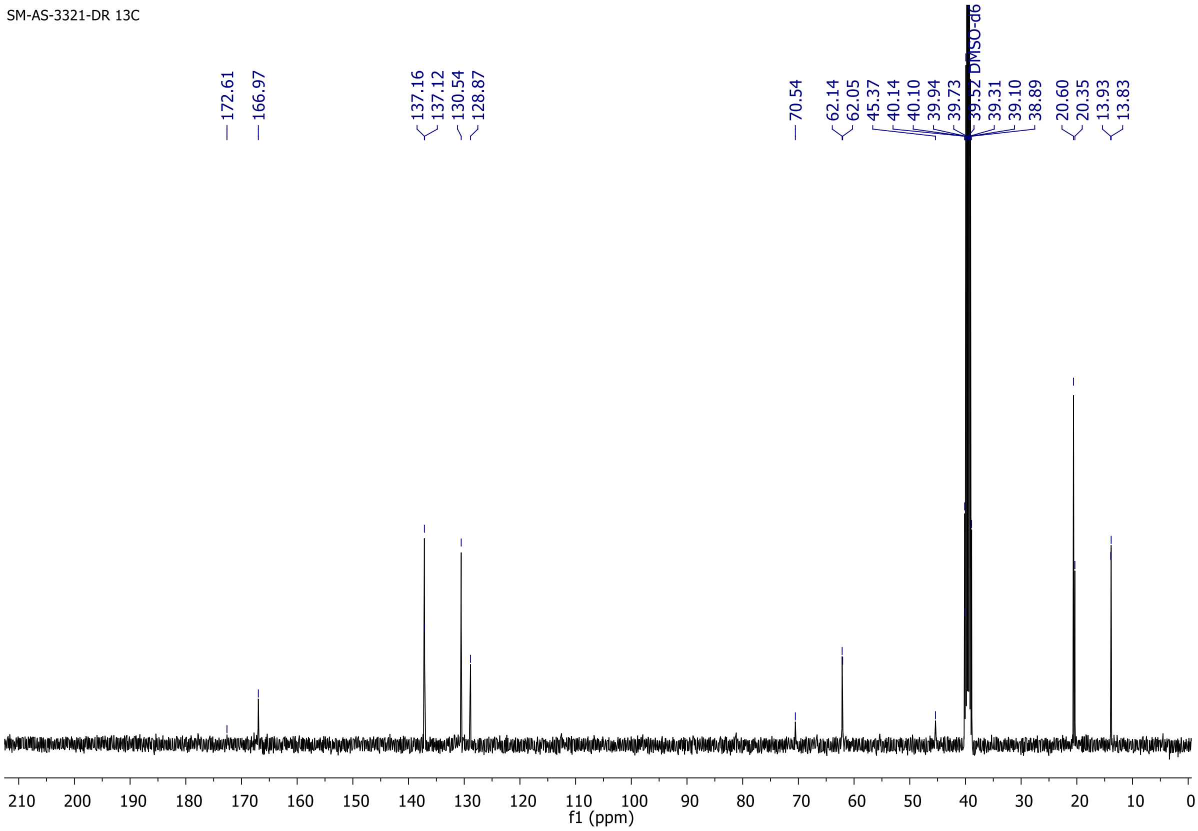

$^1H$ NMR of **3l** (400 MHz, $CDCl_3$):

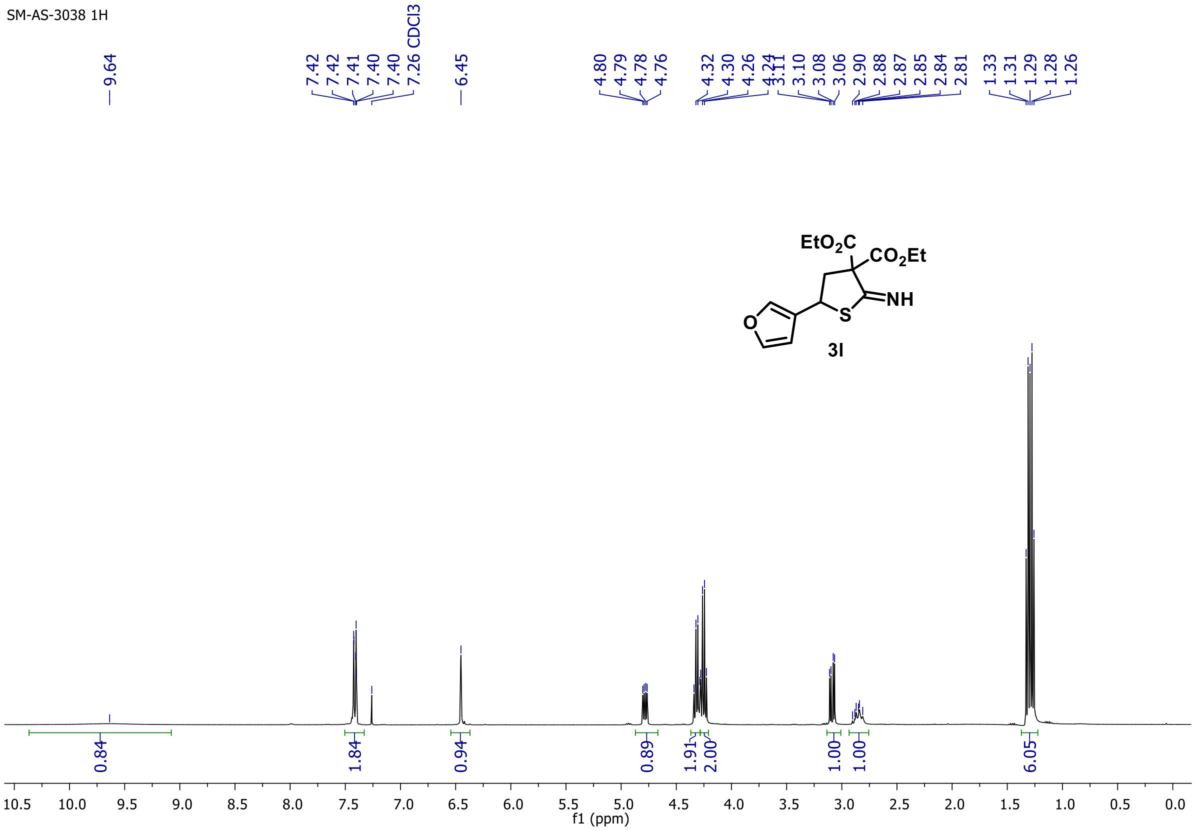


$^{13}C\{^1H\}$ NMR of **3l** (101 MHz, $CDCl_3$):

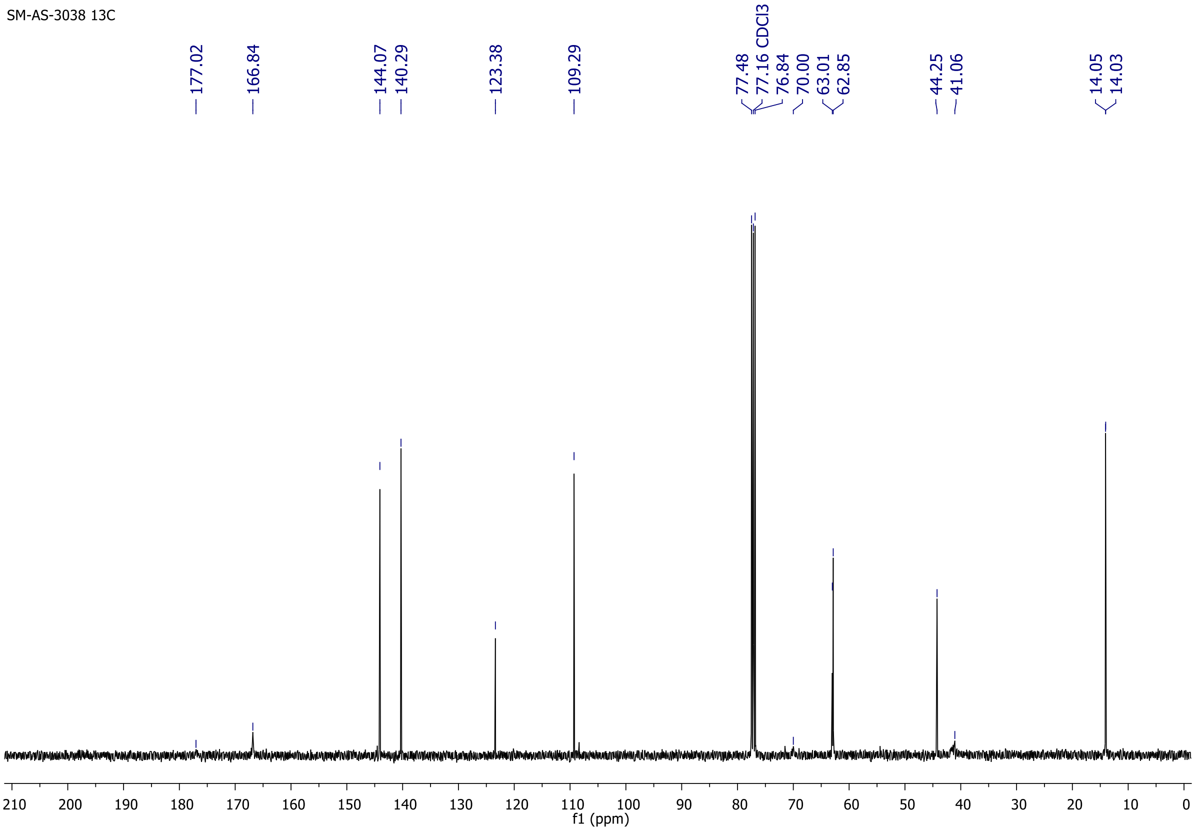

$^{1}H$ NMR of **3m** (400 MHz, DMSO-$d_6$):

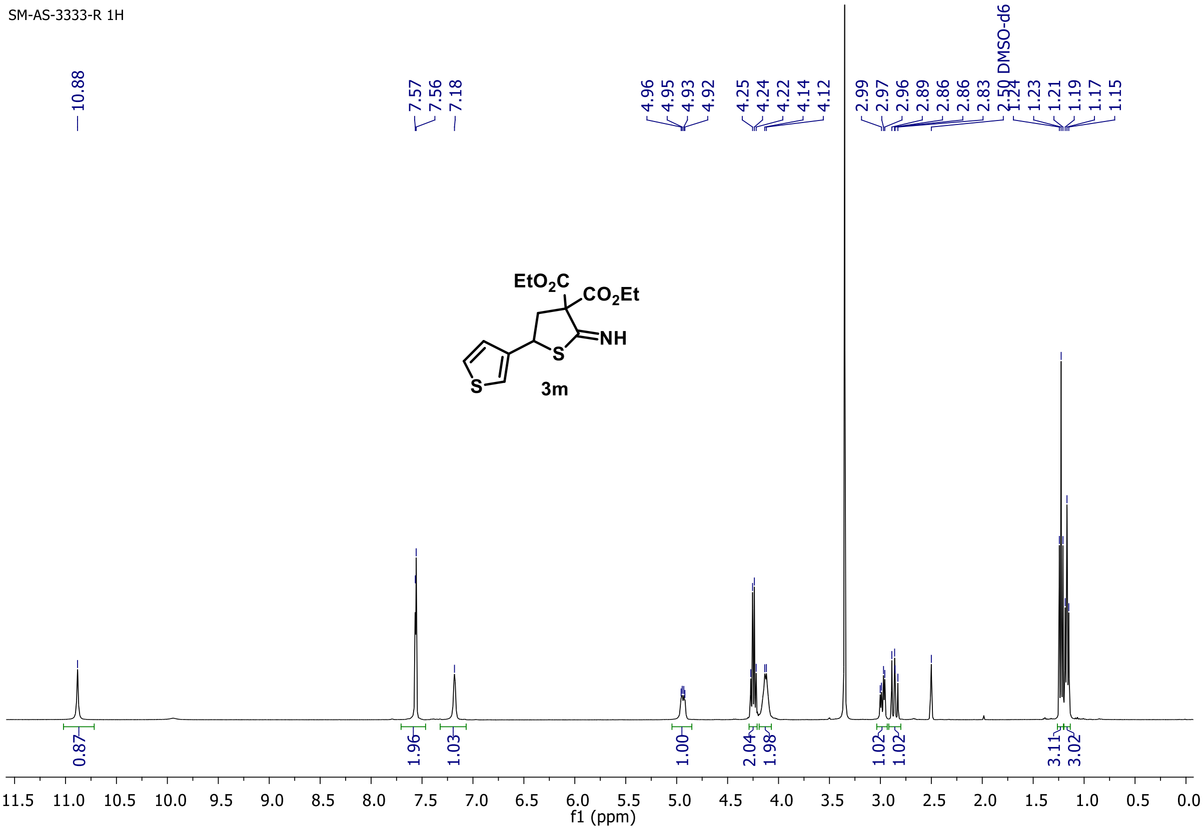


$^{13}C\{^{1}H\}$ NMR of **3m** (101 MHz, DMSO-$d_6$):

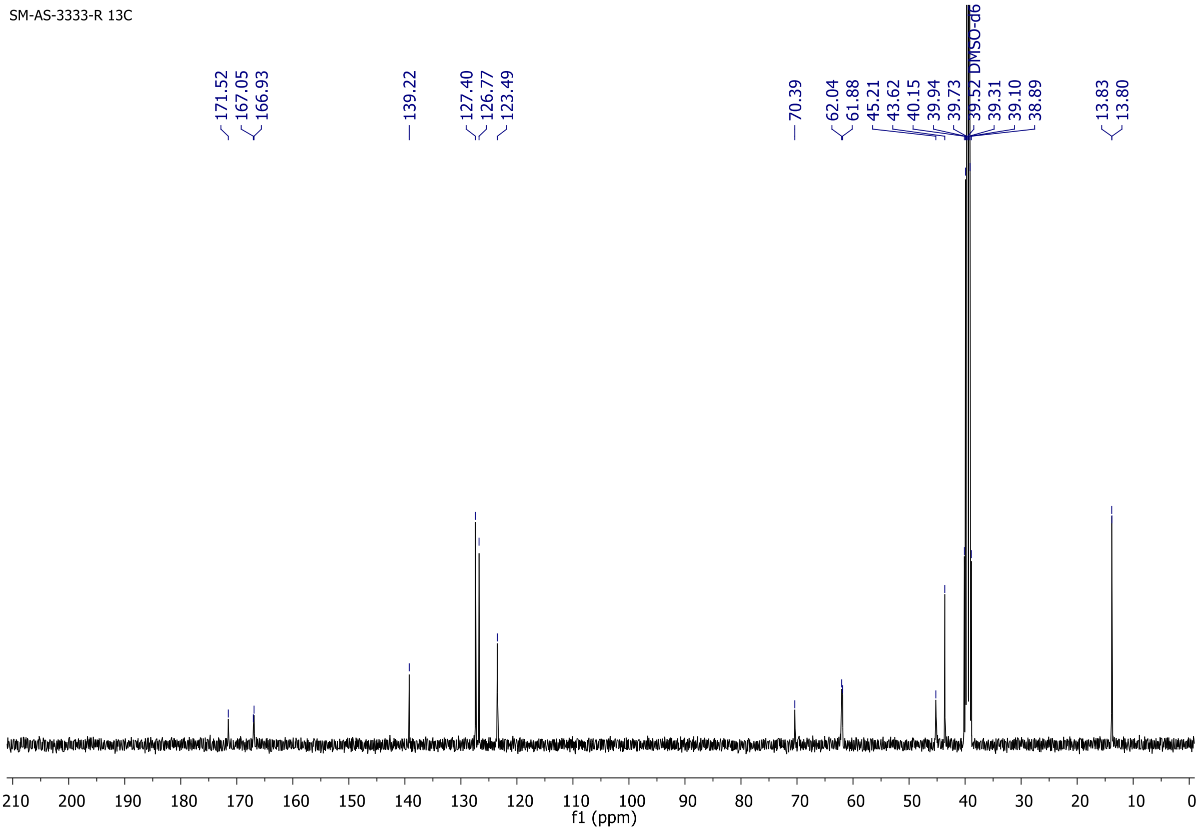

$^1$H NMR of **3n** (400 MHz, $CDCl_3$):

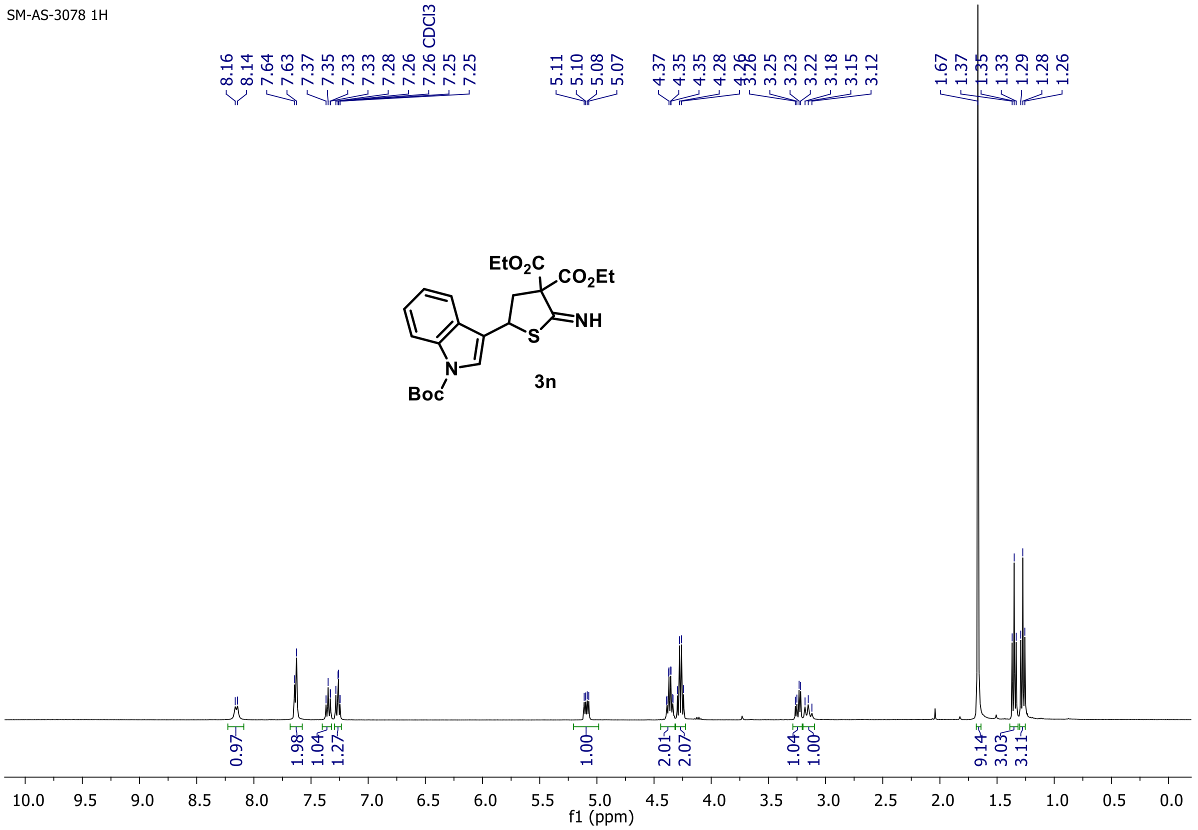


$^{13}C\{^1H\}$ NMR of **3n** (101 MHz, $CDCl_3$):

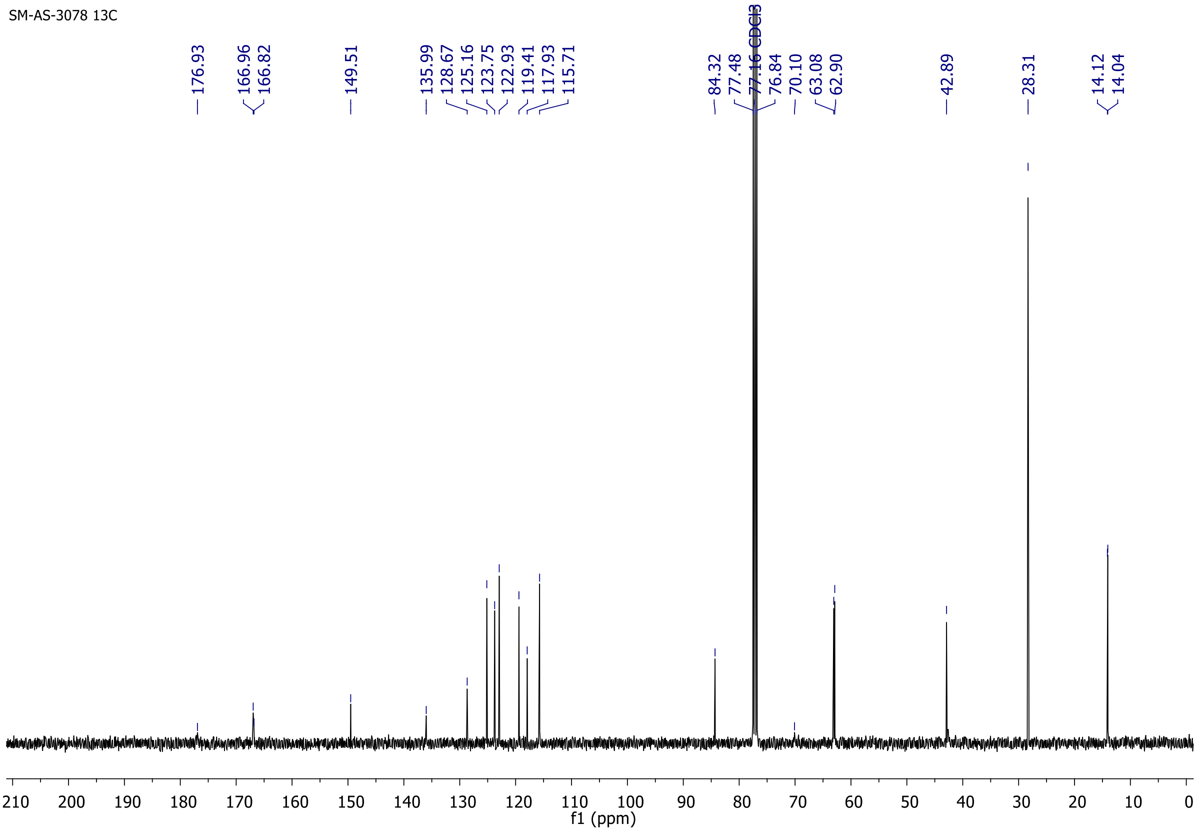

$^{1}$H NMR of **3o** (400 MHz, $CDCl_3$):

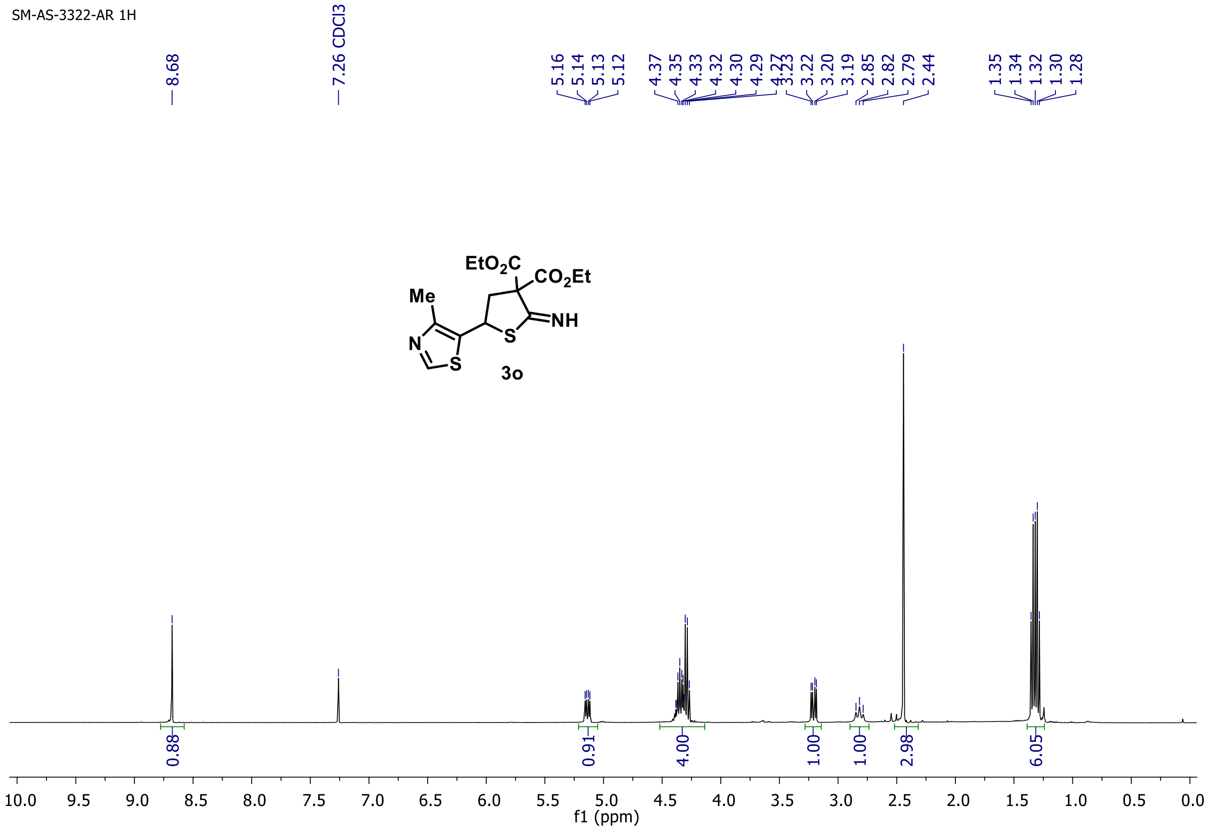


$^{13}$C{$^{1}$H} NMR of **3o** (101 MHz, $CDCl_3$):

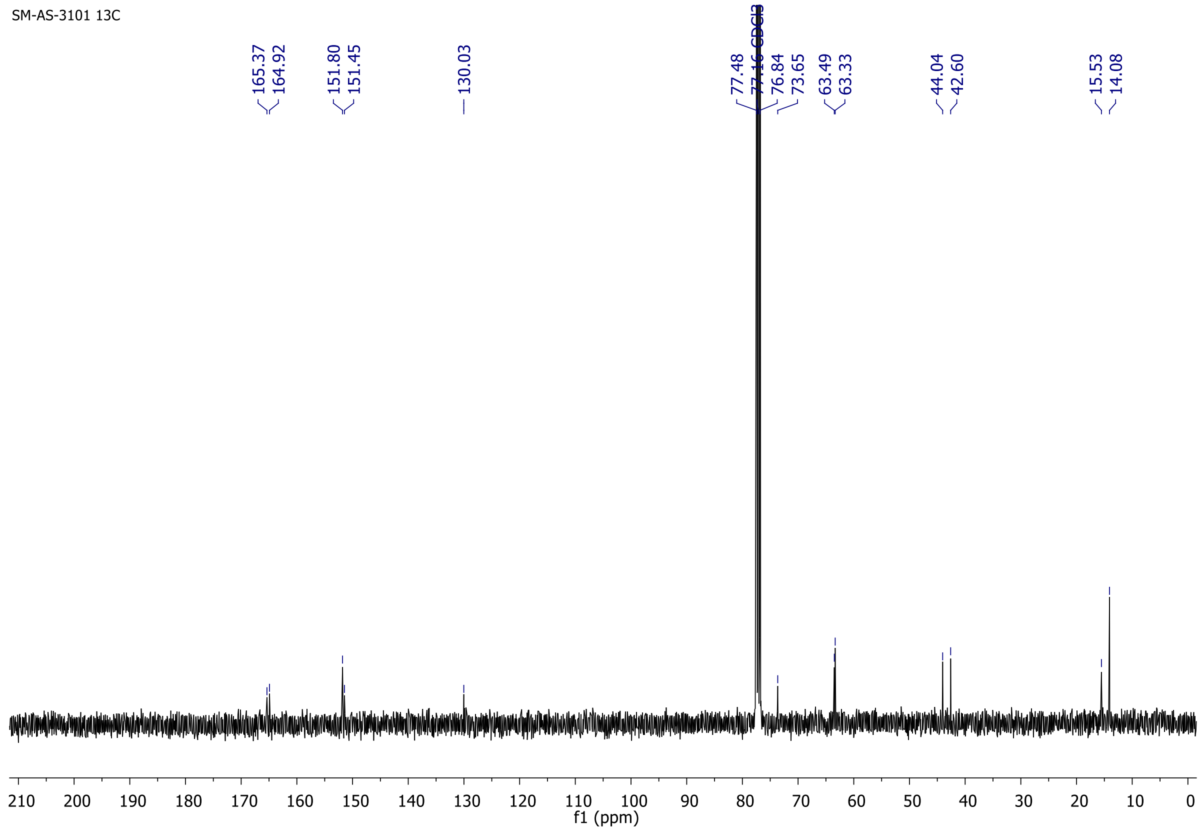

$^{1}H$ NMR of **3p** (400 MHz, $CDCl_3$):

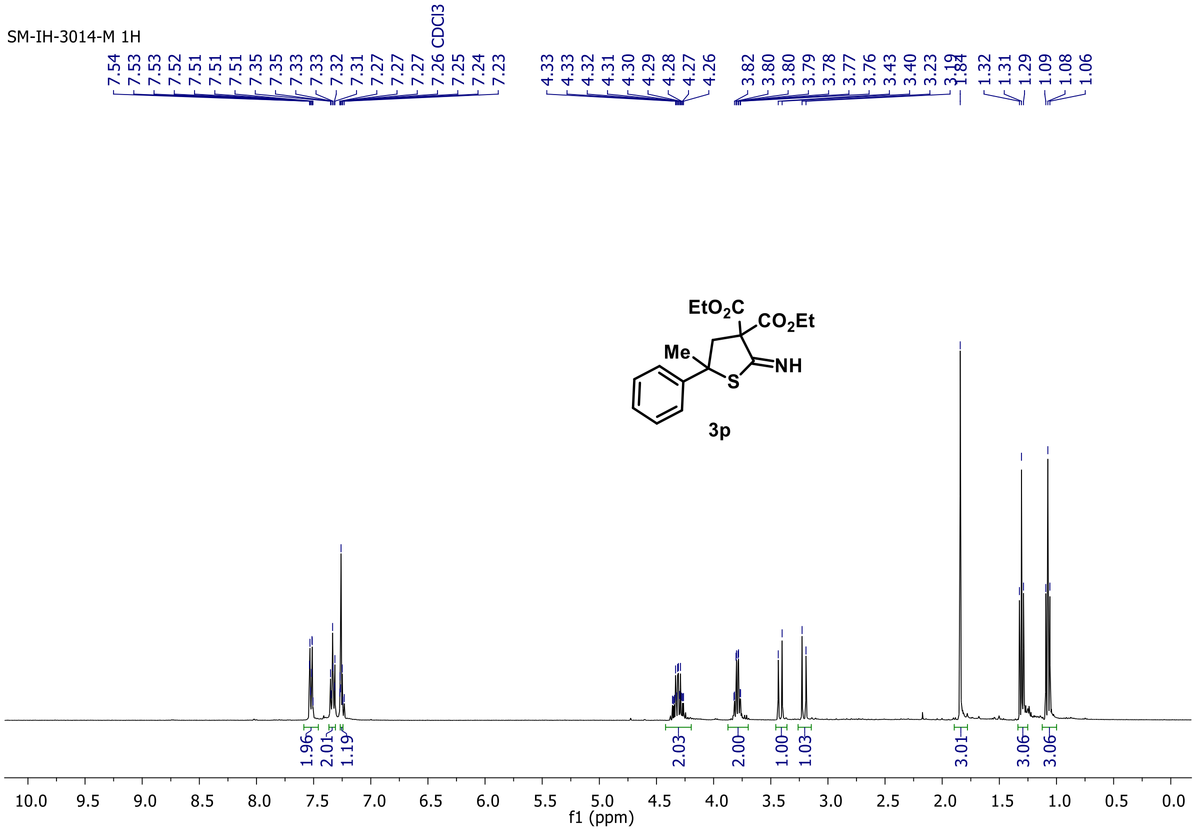


$^{13}C\{^{1}H\}$ NMR of **3p** (101 MHz, $CDCl_3$):

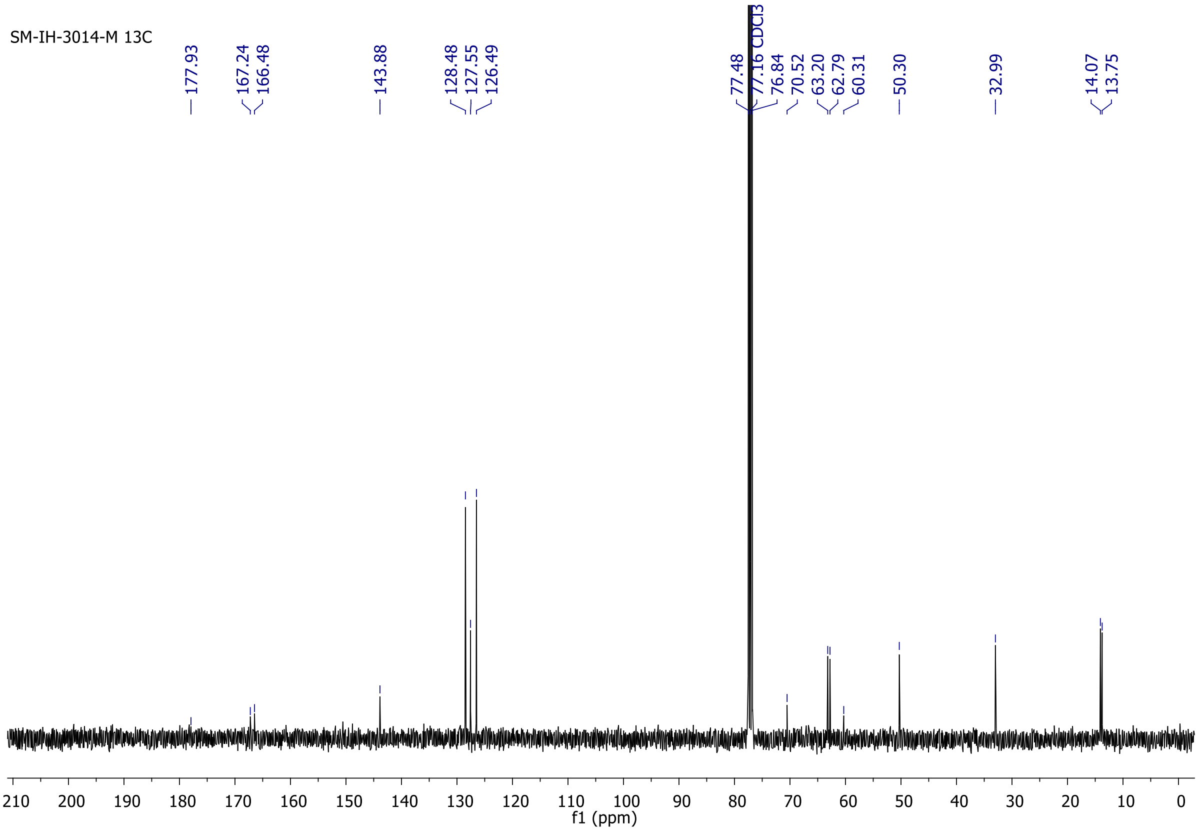

$^{1}$H NMR of **3q** (400 MHz, $CDCl_3$):

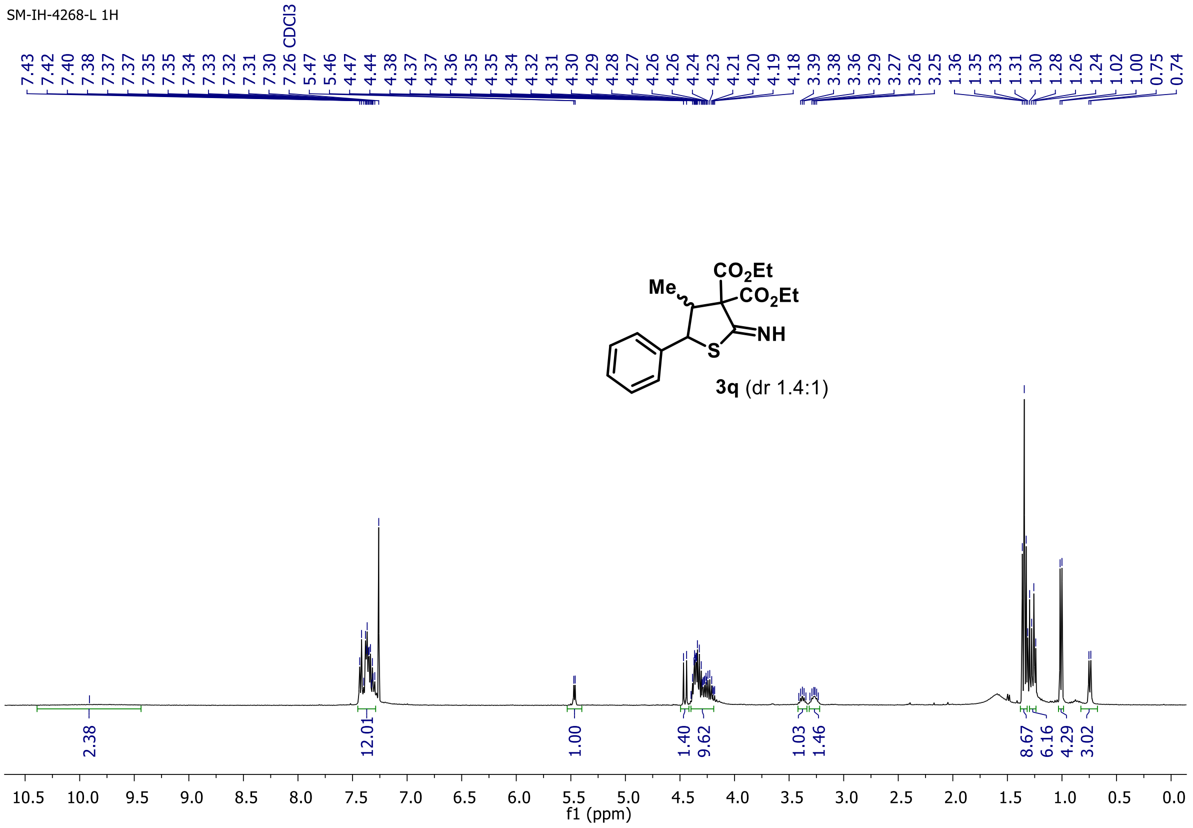


$^{13}$C{$^{1}$H} NMR of **3q** (101 MHz, $CDCl_3$):

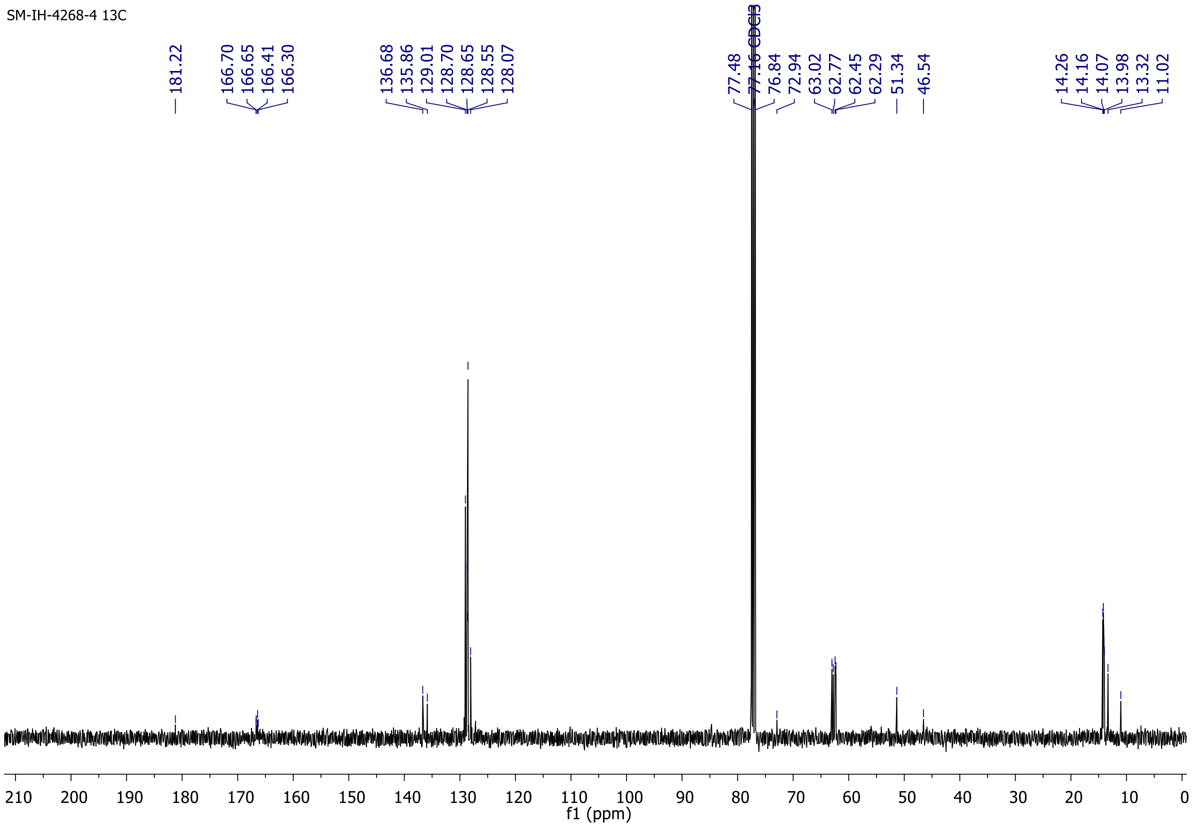

$^{1}H$ NMR of **3r** (400 MHz, $CDCl_3$):

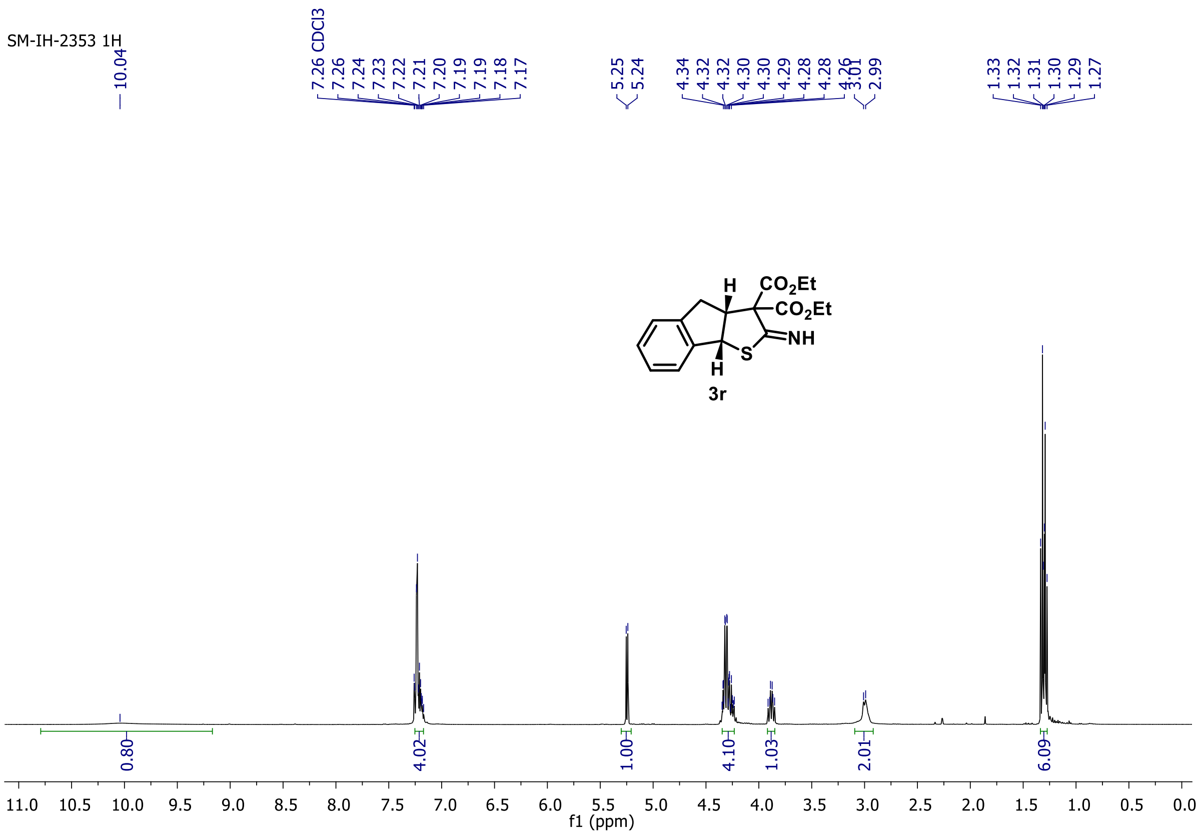


$^{13}C\{^{1}H\}$ NMR of **3r** (101 MHz, $CDCl_3$):

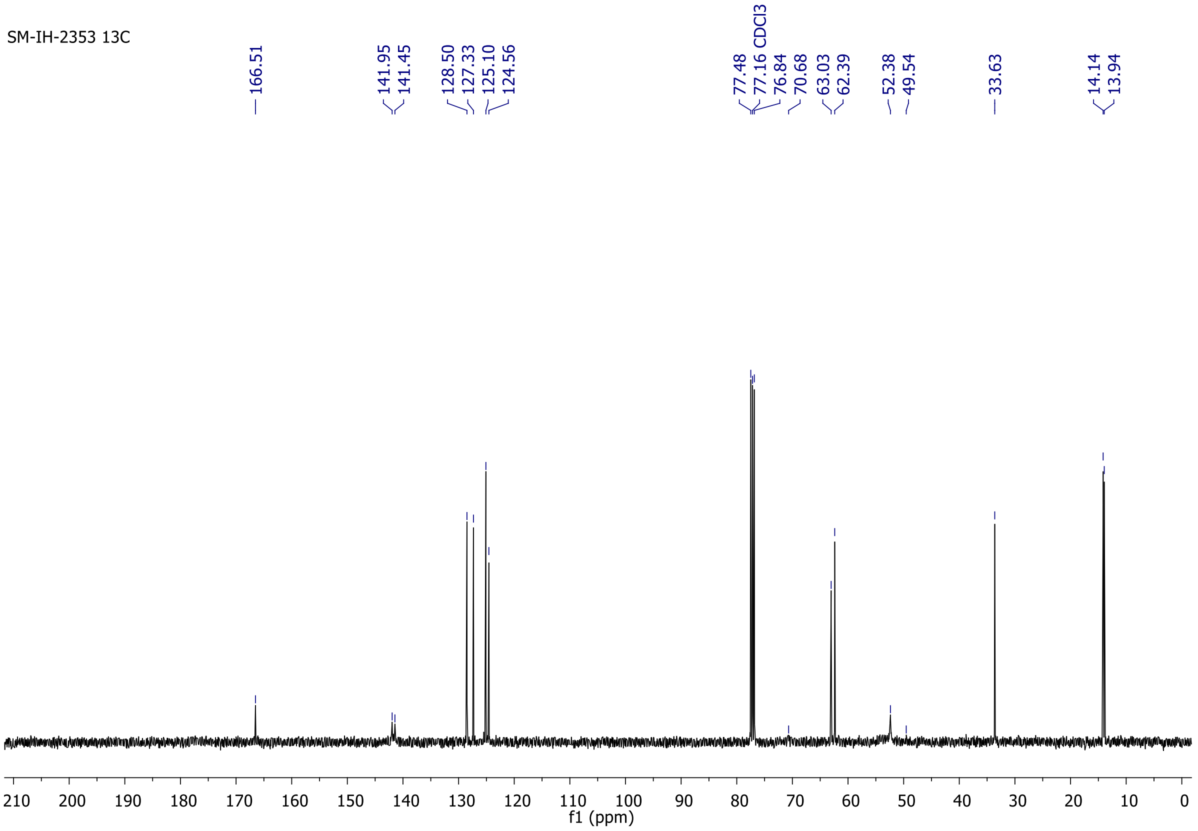

$^1H$-$^1H$ COSY NMR of **3r**:

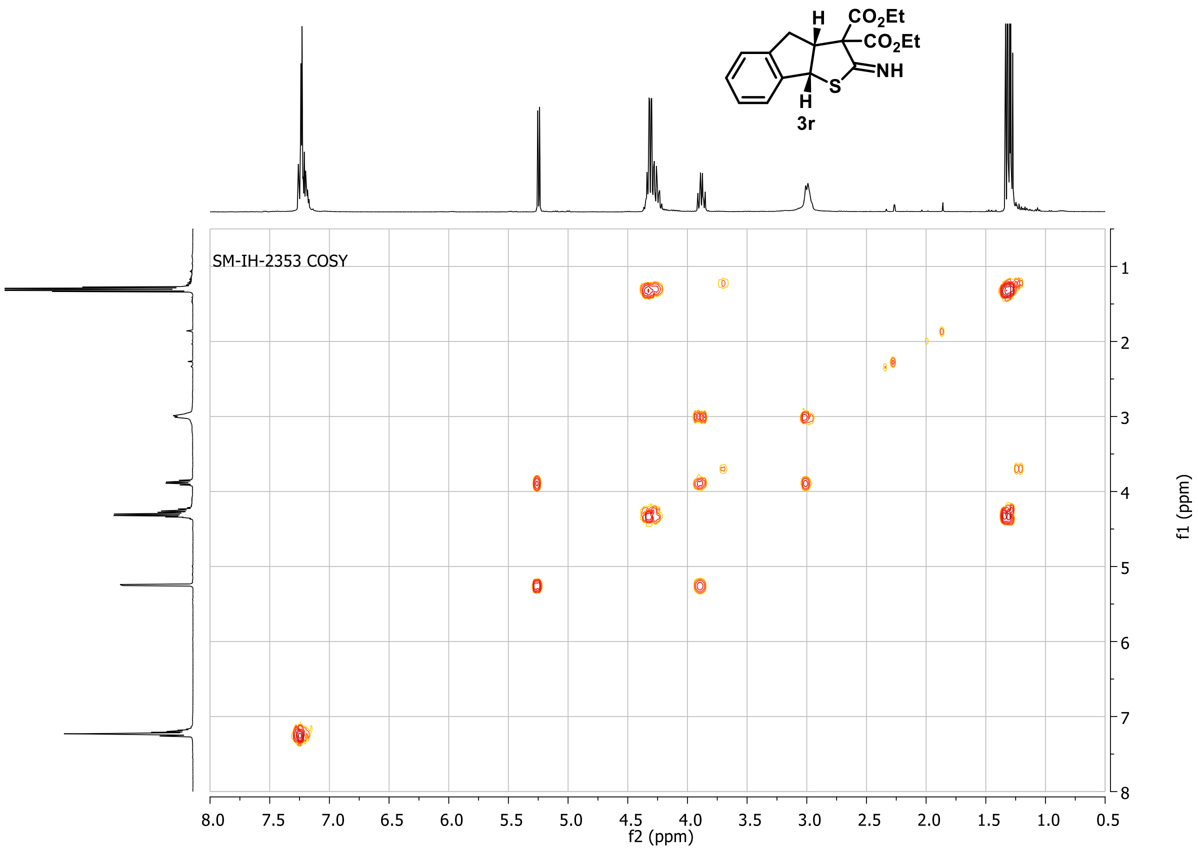


$^1H$-$^{13}C$ HSQC NMR of **3r**:

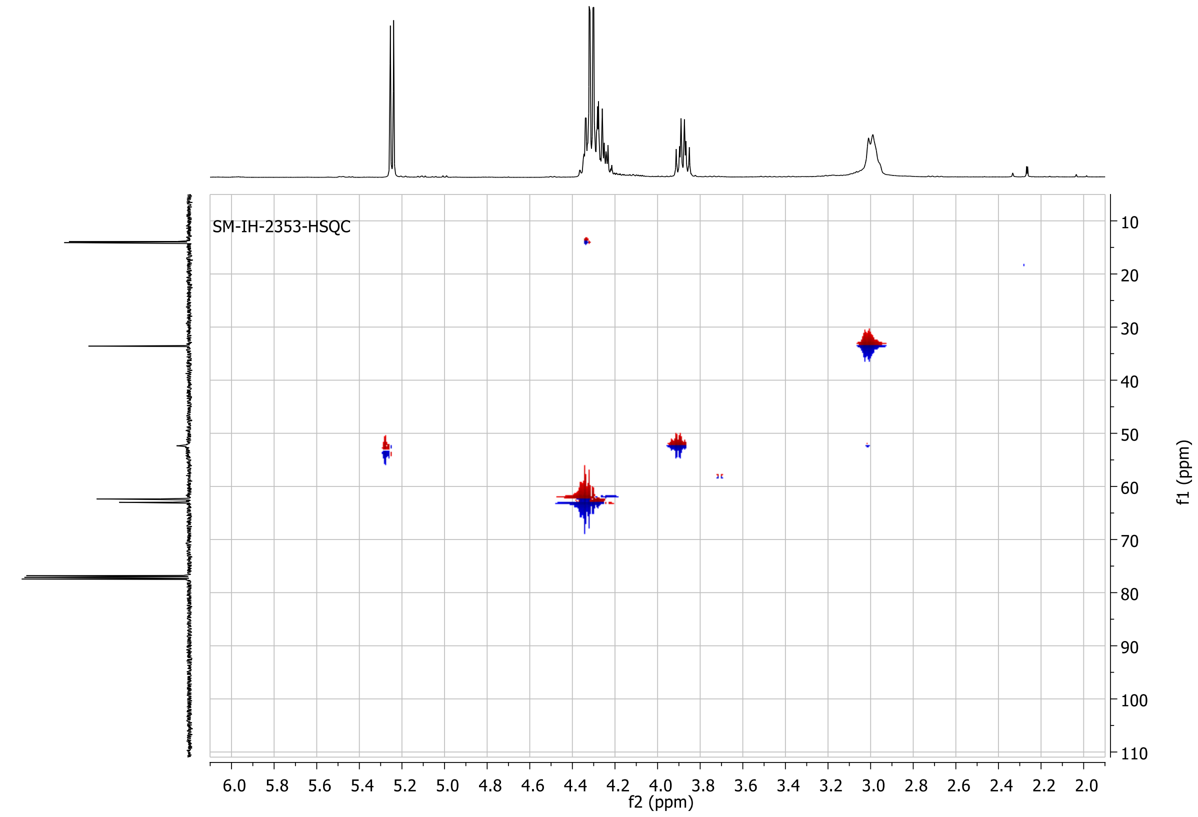

$^1H$-$^1H$ NOESY NMR of **3r**:

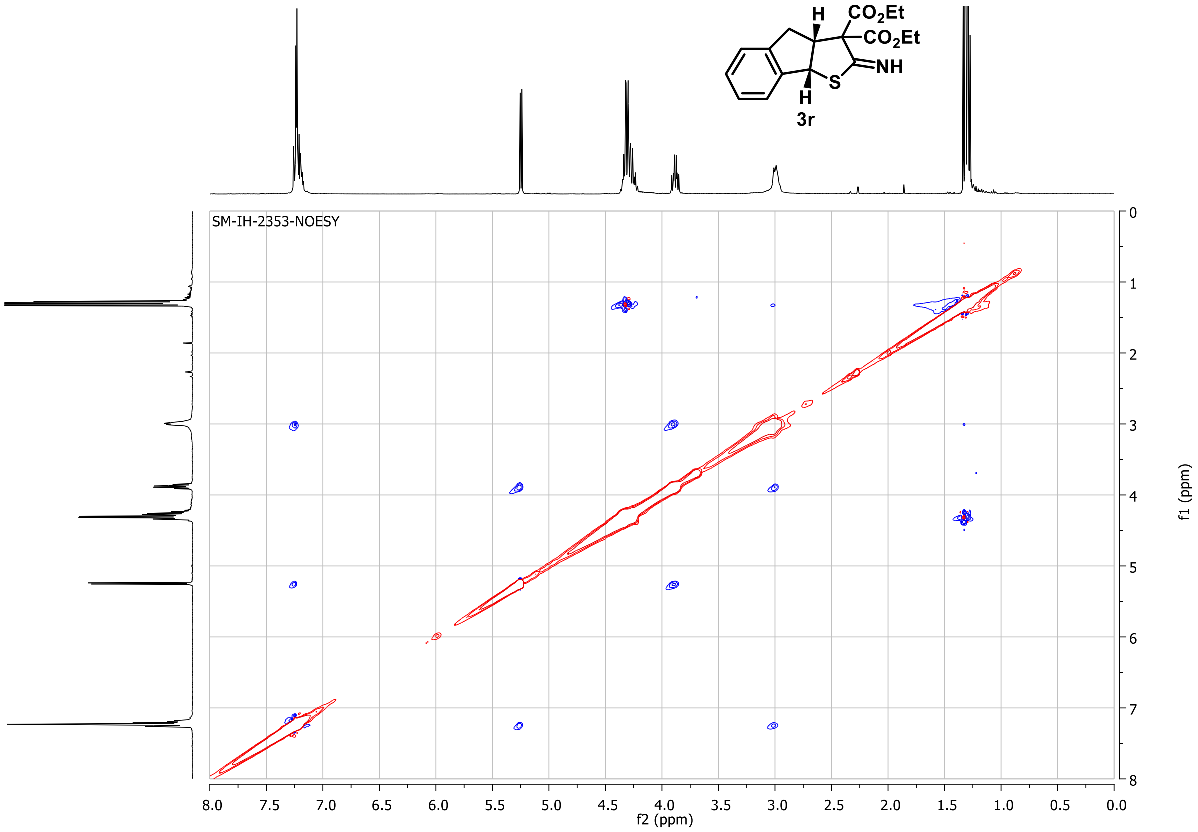

$^{1}H$ NMR of **3s** (400 MHz, $CDCl_3$):

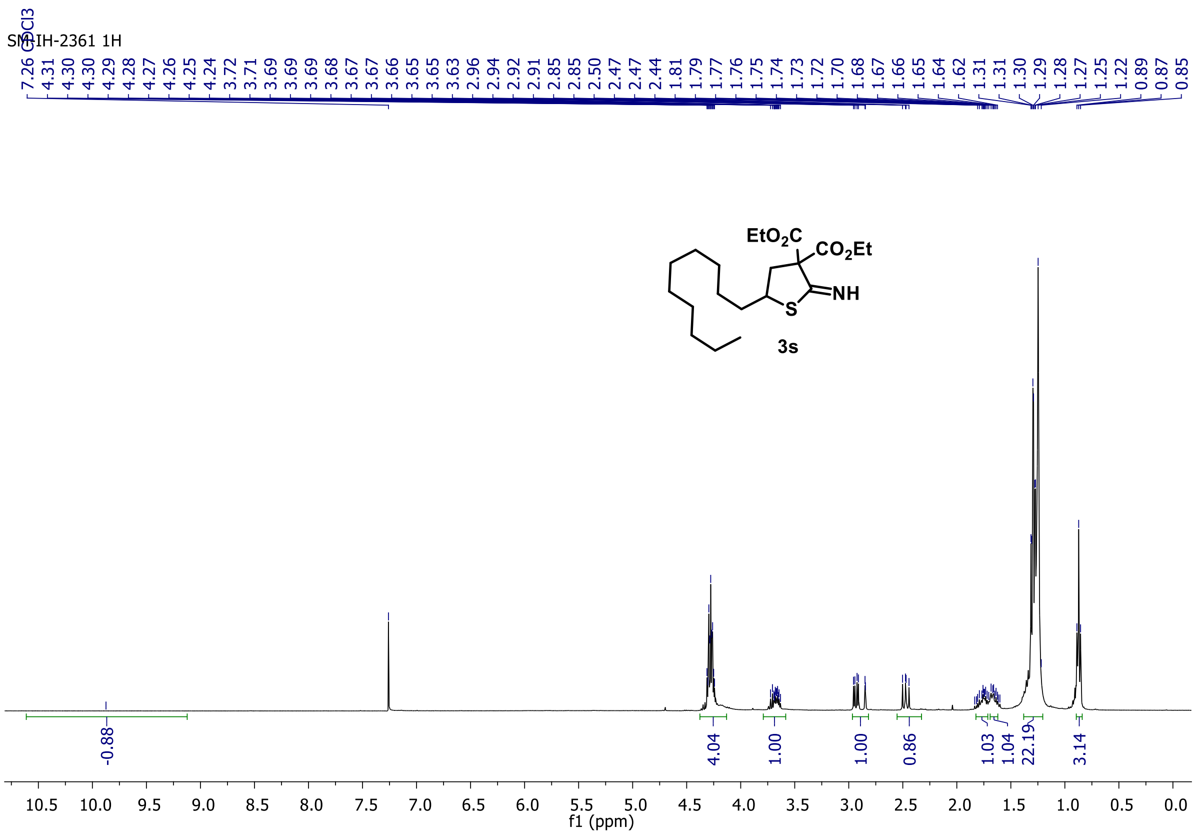


$^{13}C\{^{1}H\}$ NMR of **3s** (101 MHz, $CDCl_3$):

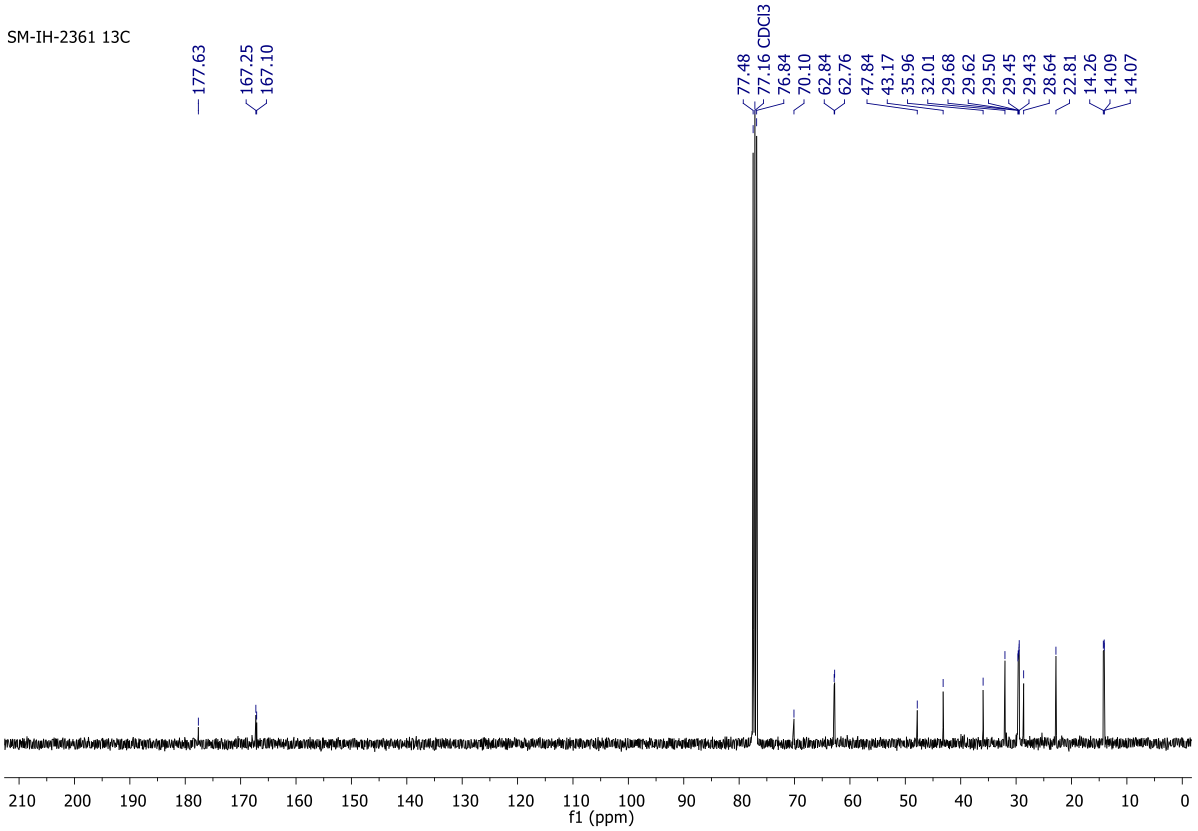

$^{1}H$ NMR of **3t** (400 MHz, $CDCl_3$):

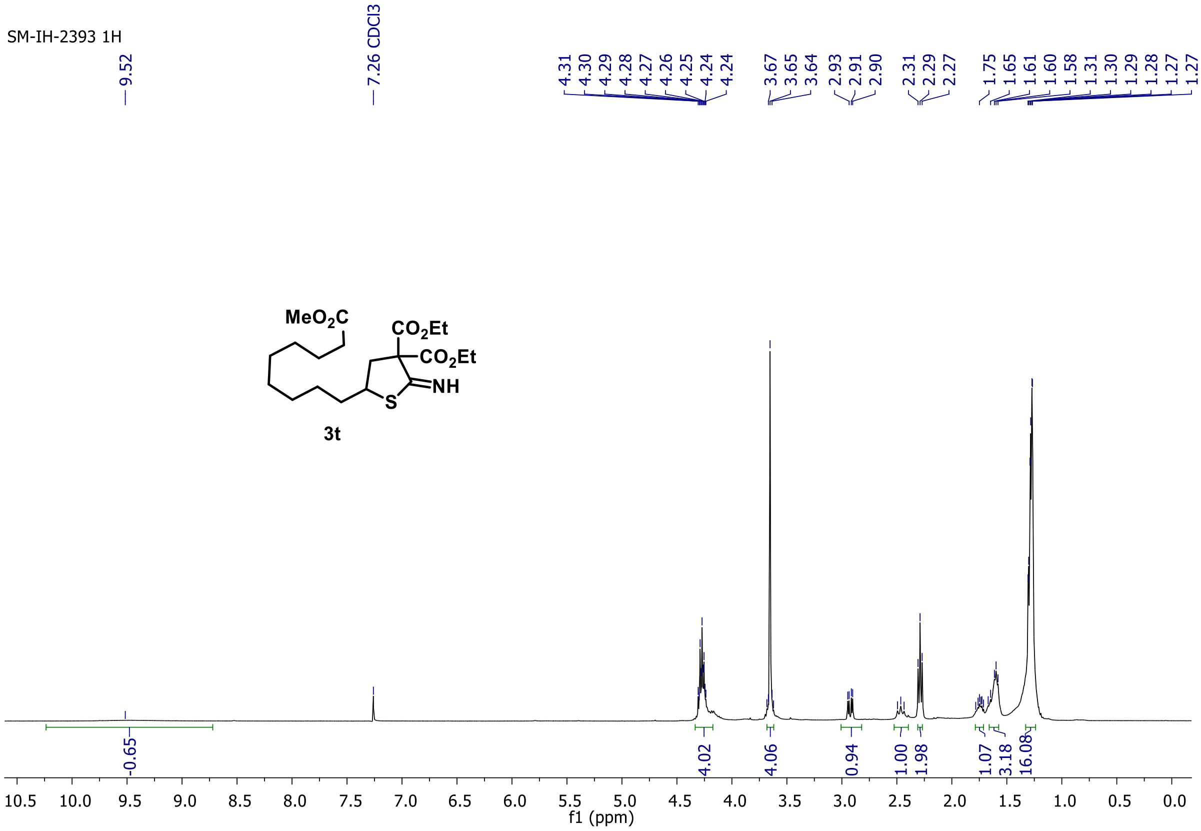


$^{13}C\{^{1}H\}$ NMR of **3t** (101 MHz, $CDCl_3$):

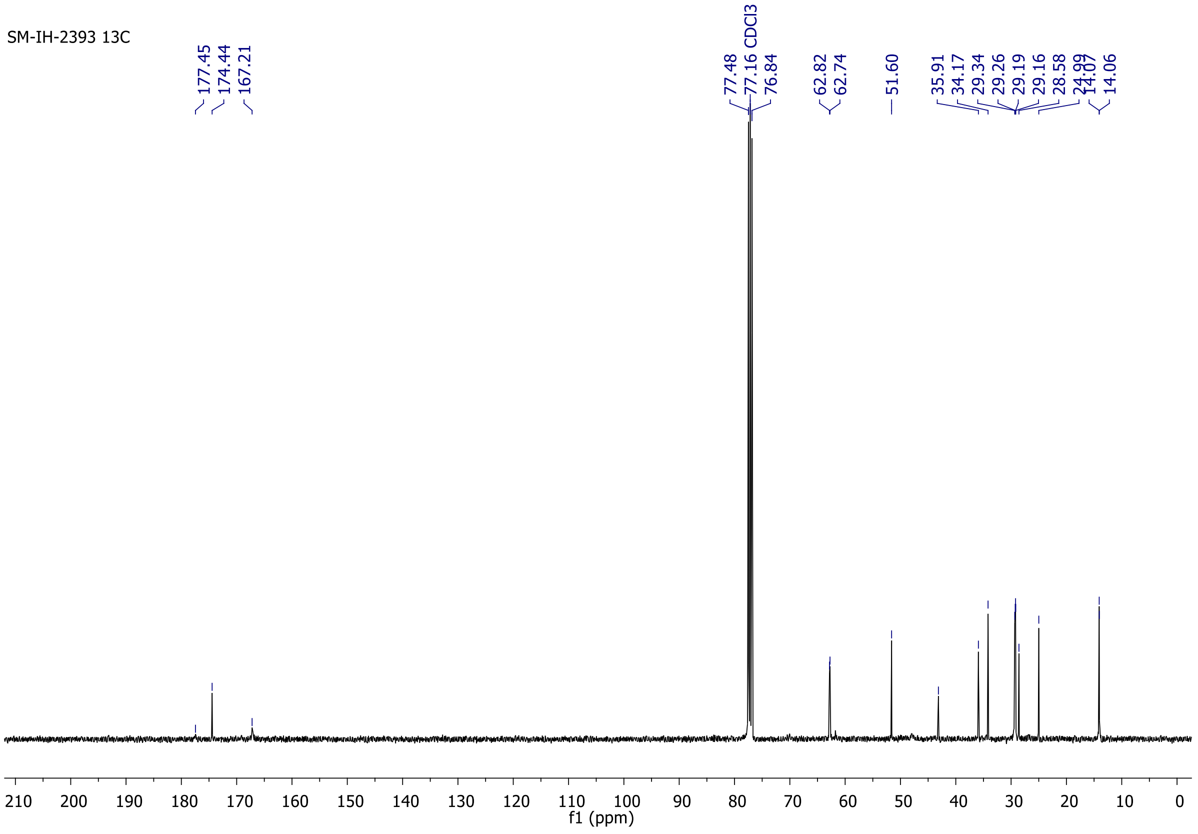

$^1H$ NMR of **3u** (400 MHz, $CDCl_3$):

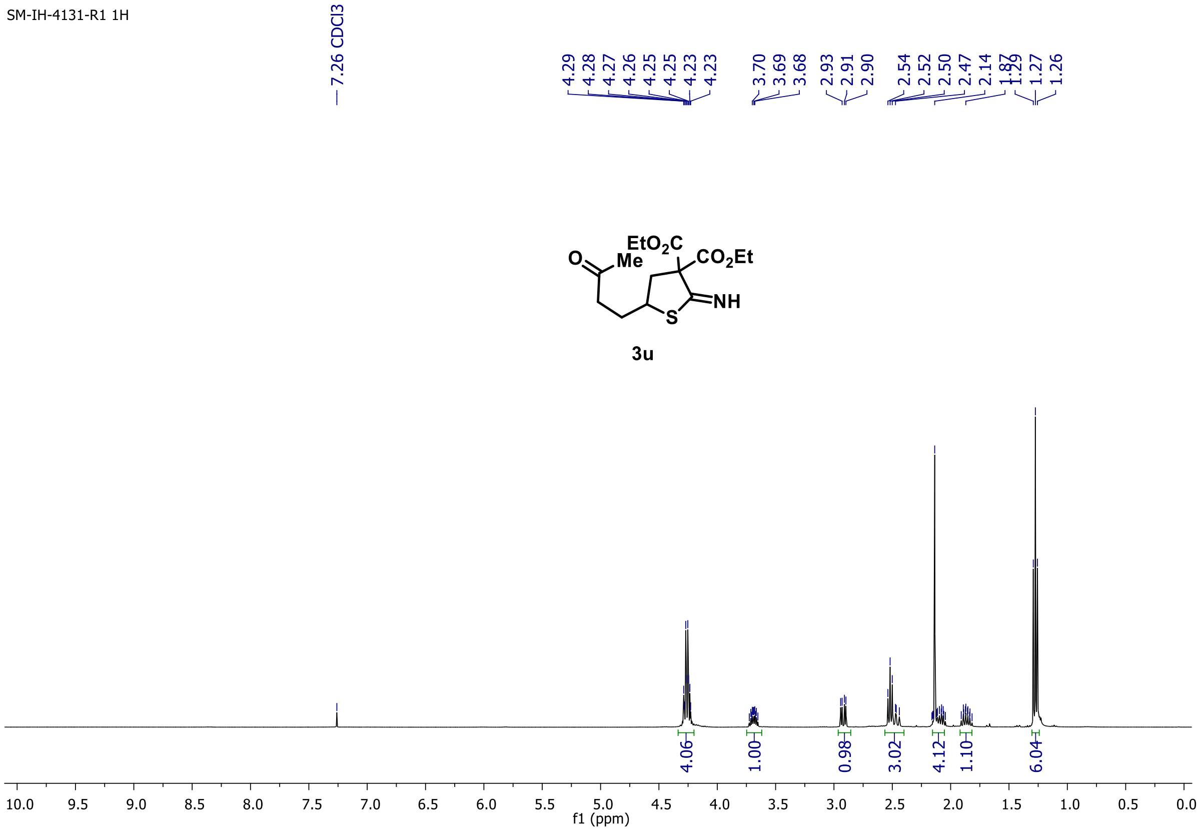


**3u**

$^{13}C\{^1H\}$ NMR of **3u** (101 MHz, $CDCl_3$):

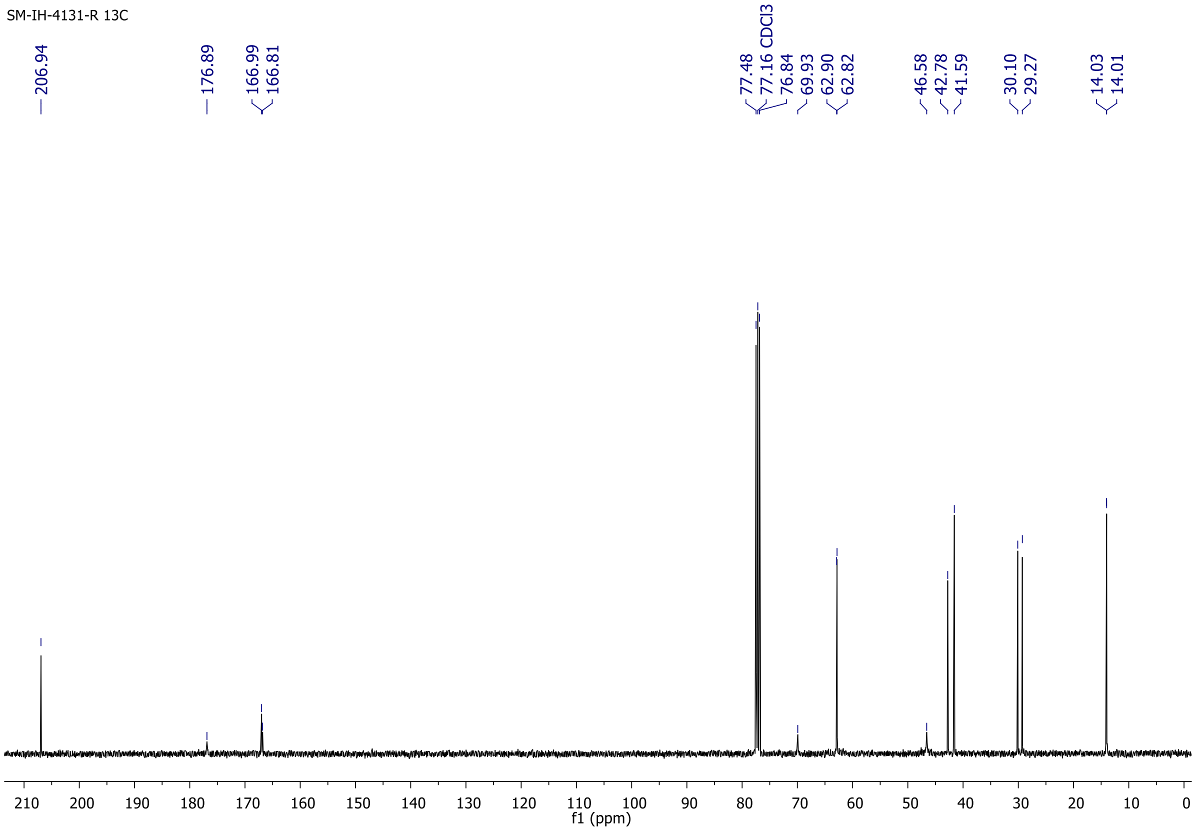

$^{1}H$ NMR of **3v** (400 MHz, $CDCl_3$):

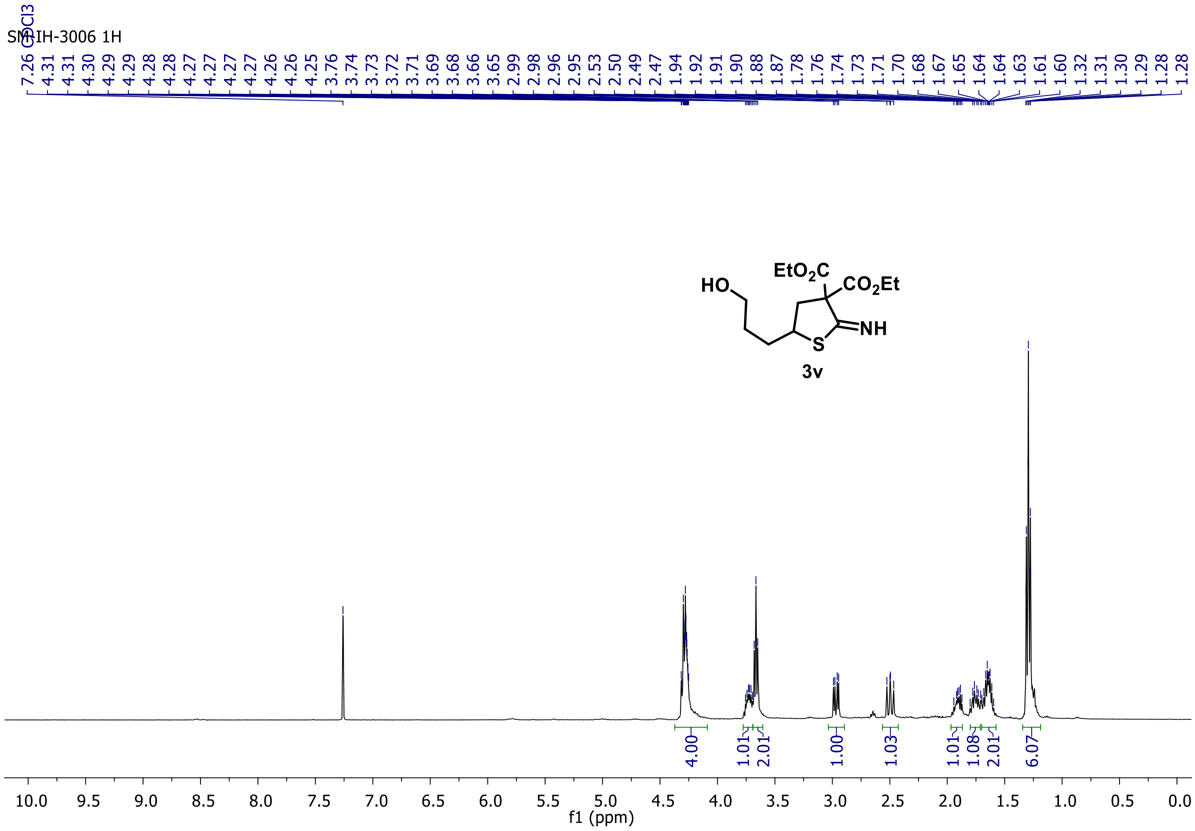


$^{13}C\{^{1}H\}$ NMR of **3v** (101 MHz, $CDCl_3$):

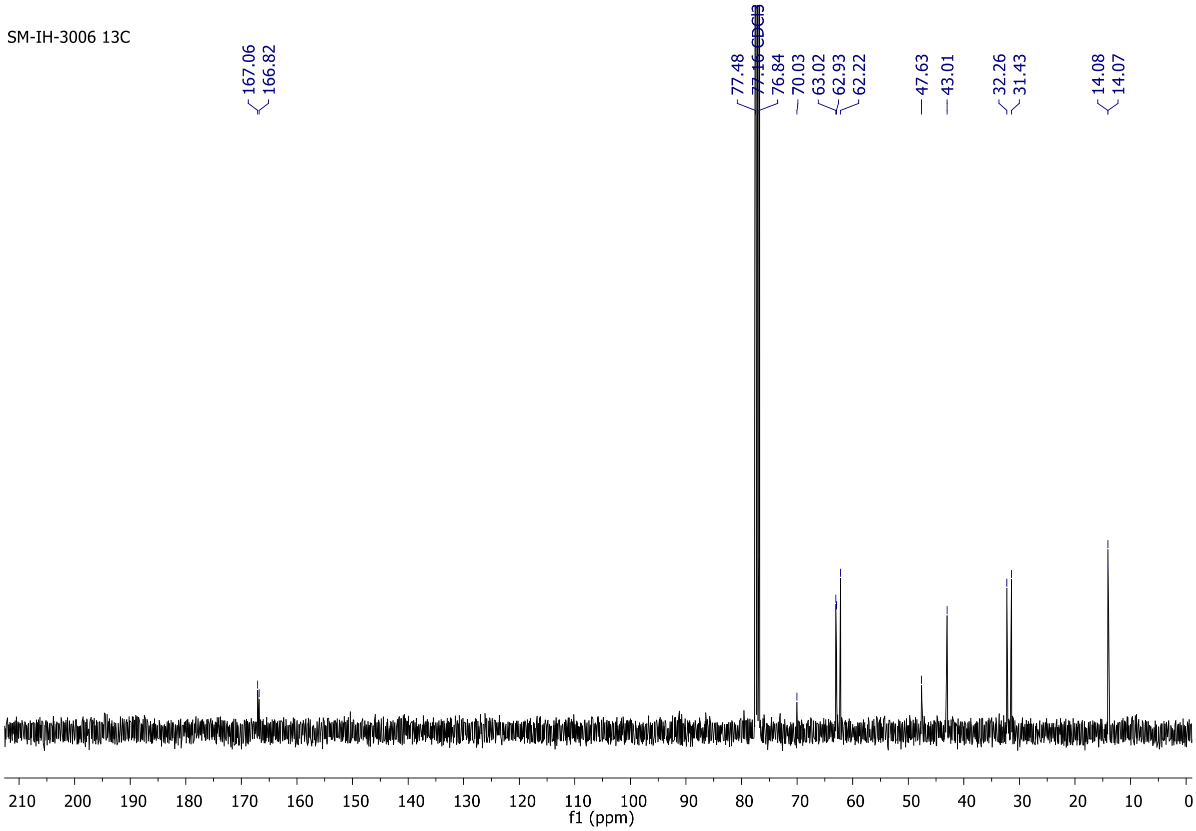

$^{1}H$ NMR of **3w** (400 MHz, $CDCl_3$):

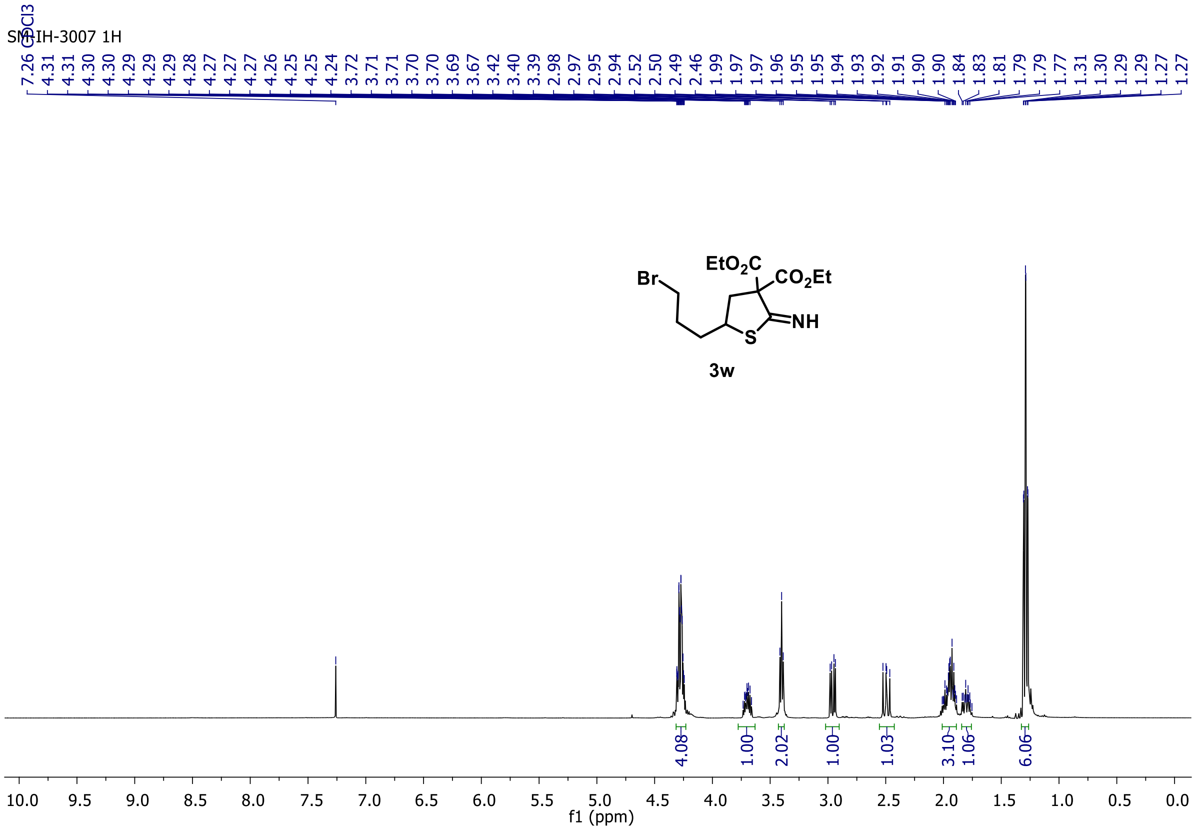


$^{13}C\{^{1}H\}$ NMR of **3w** (101 MHz, $CDCl_3$):

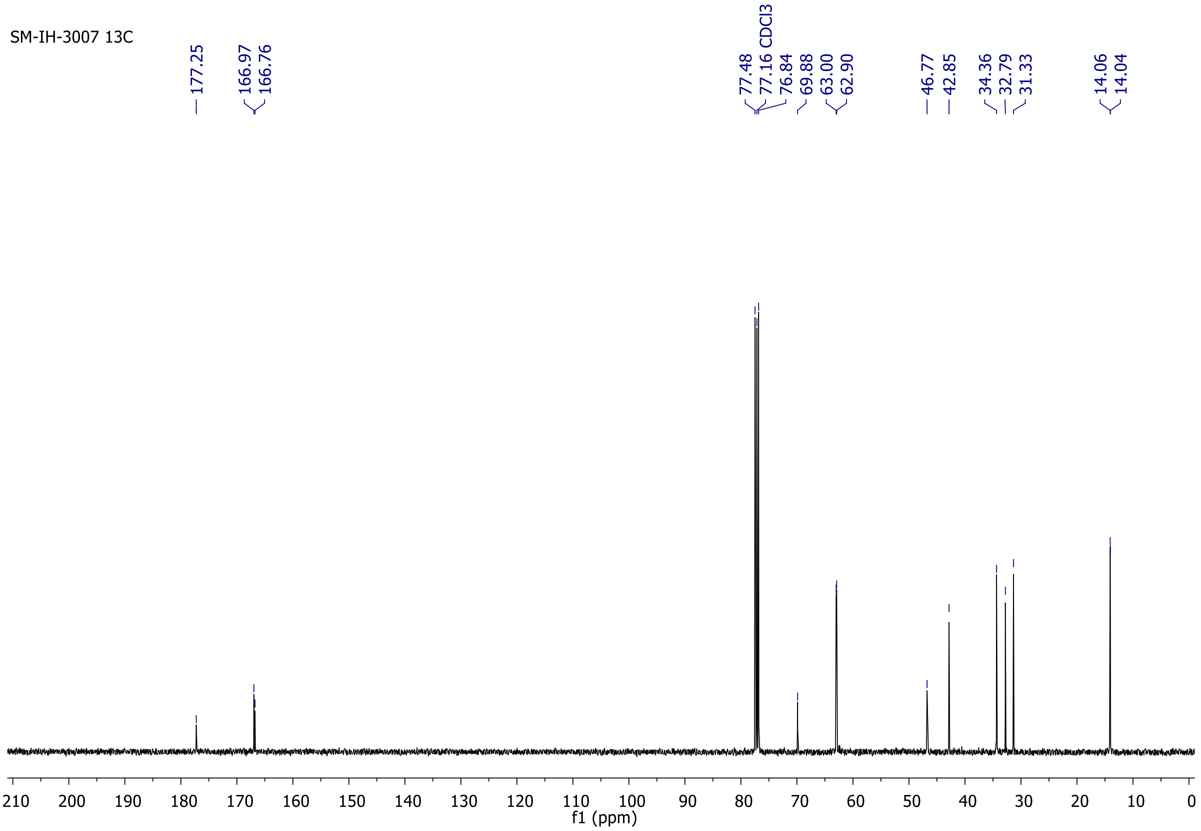

$^{1}H$ NMR of **3x** (400 MHz, $CDCl_3$):

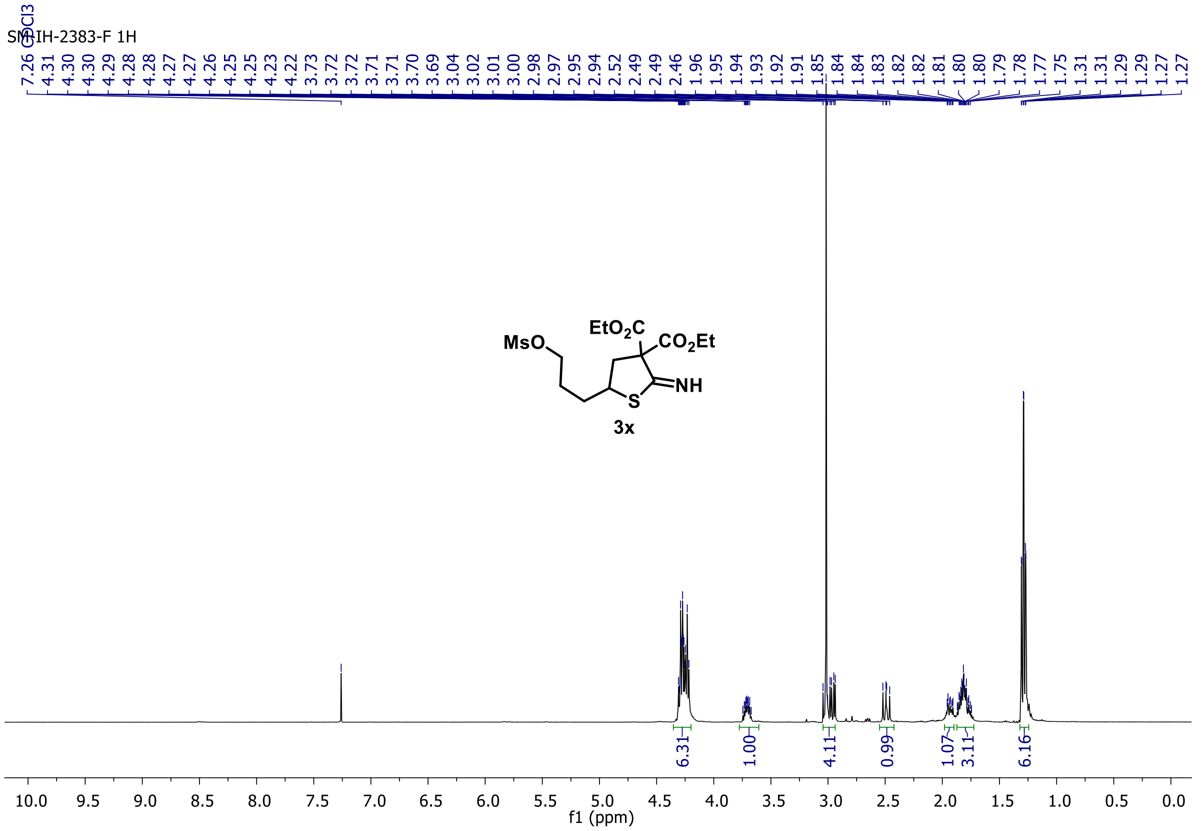


$^{13}C\{^{1}H\}$ NMR of **3x** (101 MHz, $CDCl_3$):

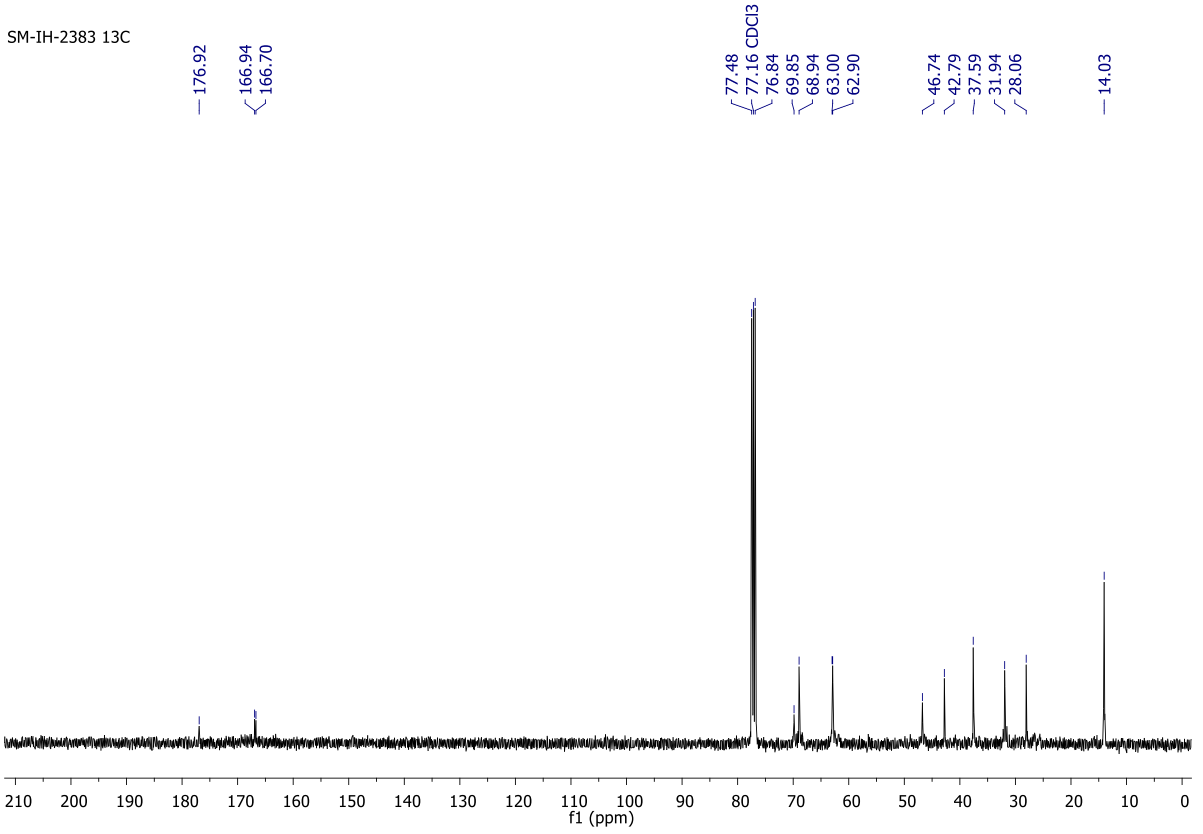

$^1H$ NMR of **3y** (400 MHz, $CDCl_3$):

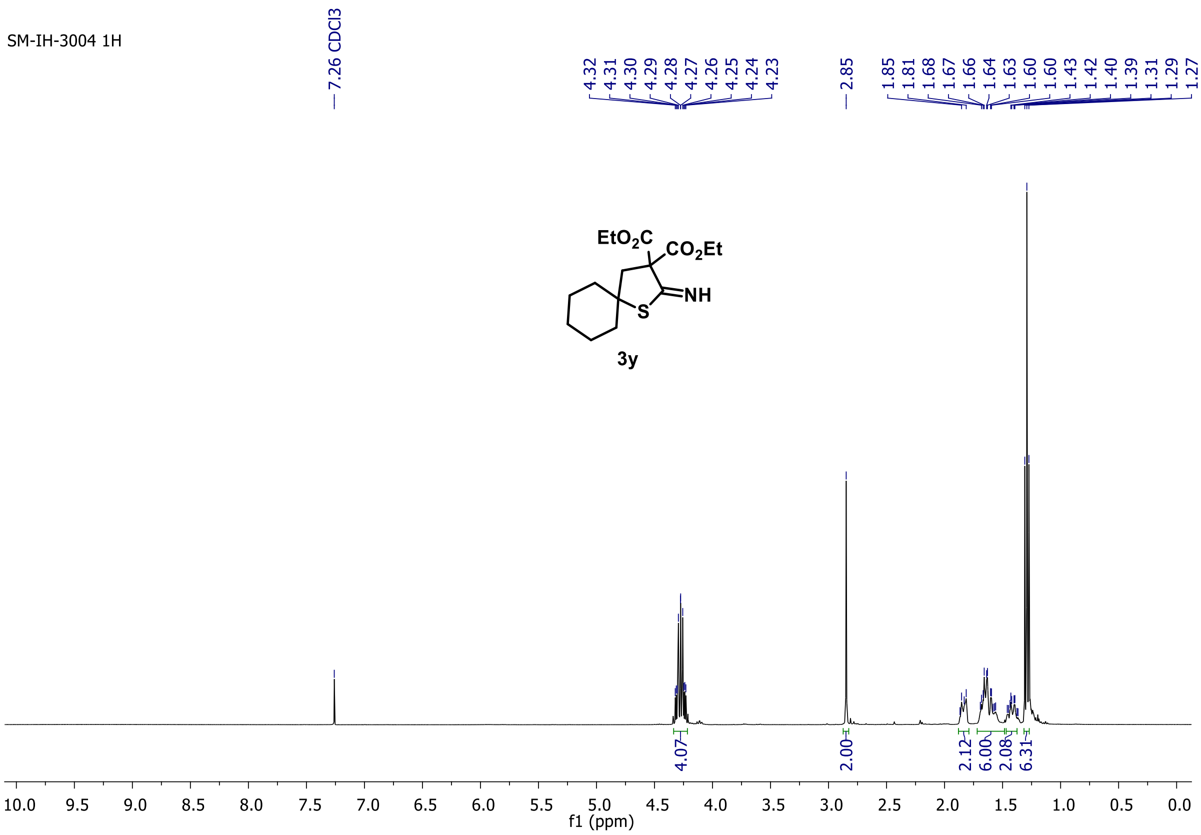


$^{13}C\{^1H\}$ NMR of **3y** (101 MHz, $CDCl_3$):

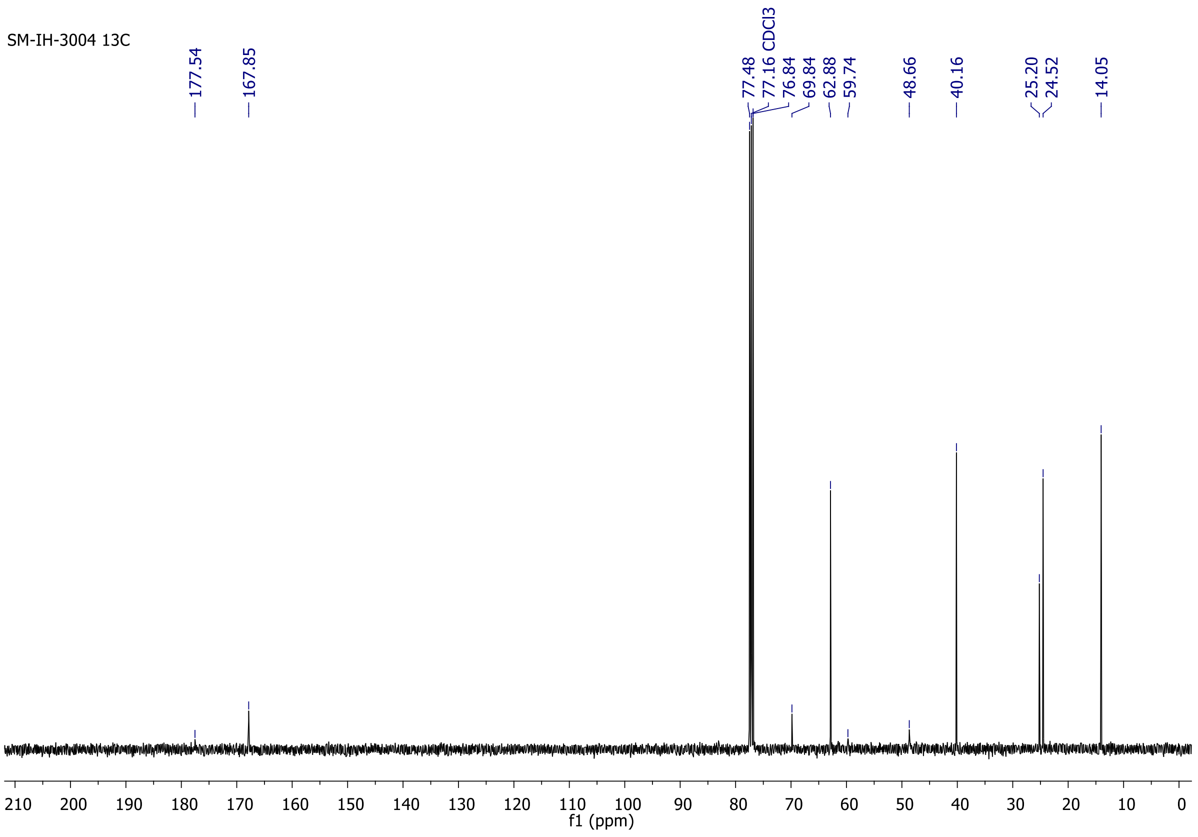

$^{1}H$ NMR of **3z** (400 MHz, $CDCl_3$):

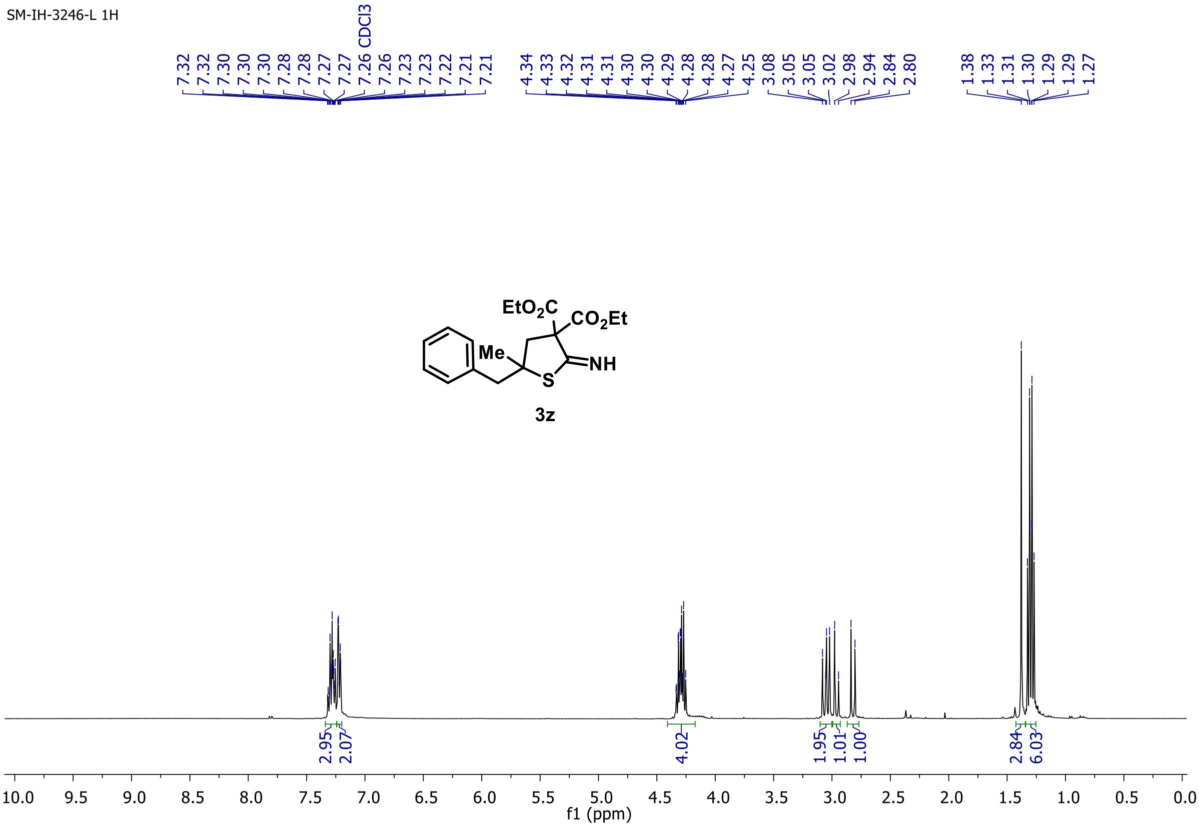


$^{13}C\{^{1}H\}$ NMR of **3z** (101 MHz, $CDCl_3$):

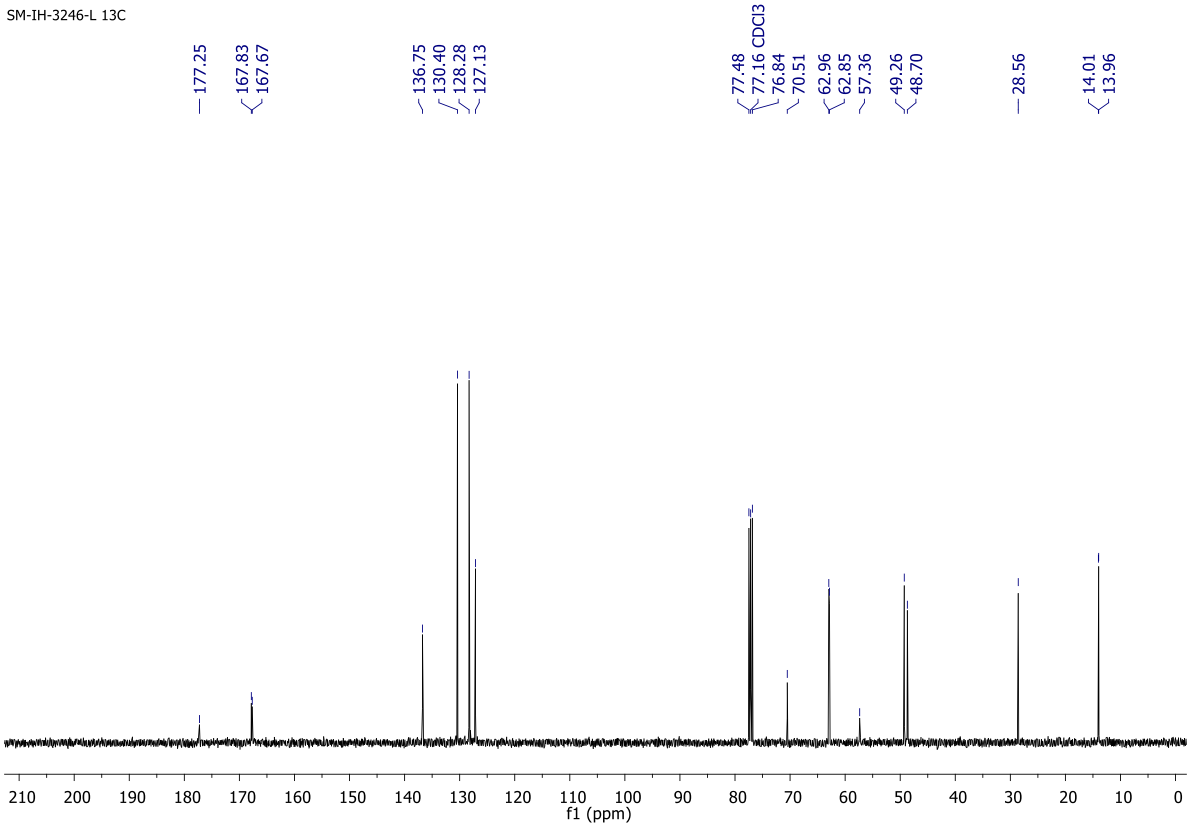

$^{1}$H NMR of **3aa** (400 MHz, $CDCl_3$):

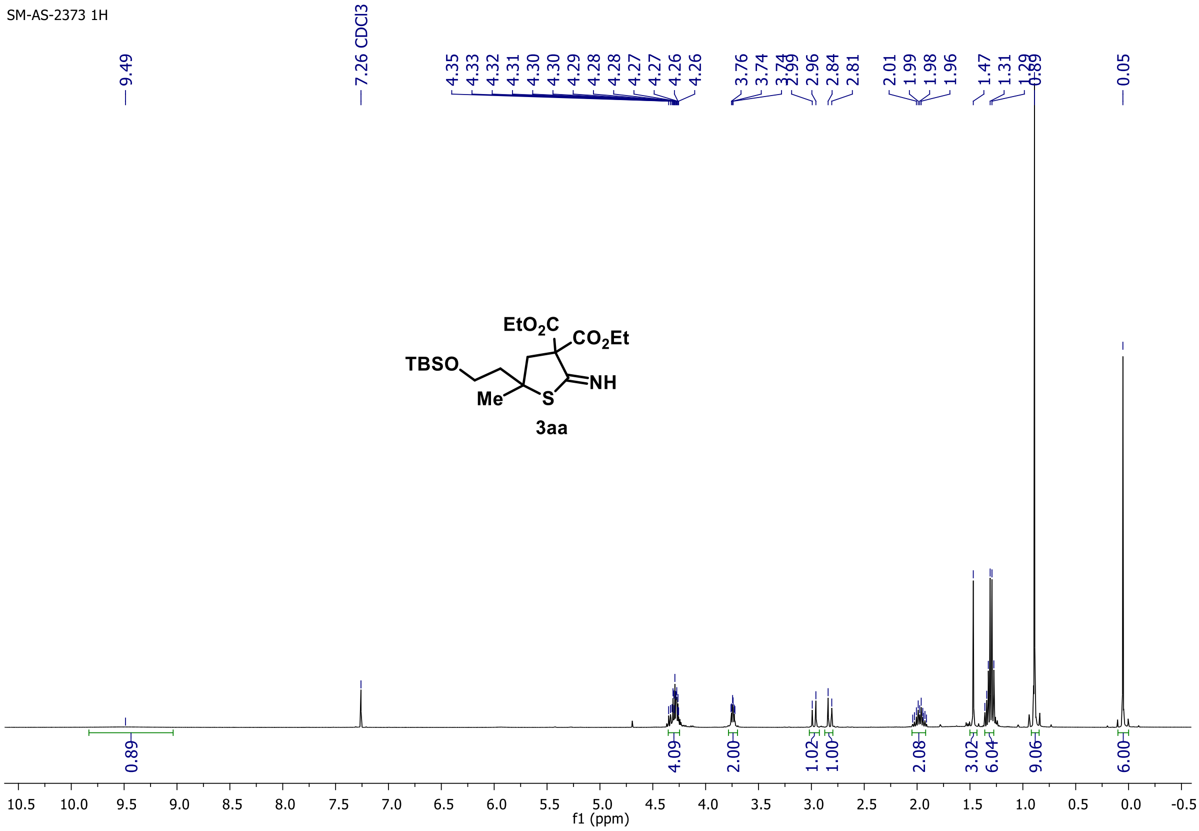


$^{13}$C{$^{1}$H} NMR of **3aa** (101 MHz, $CDCl_3$):

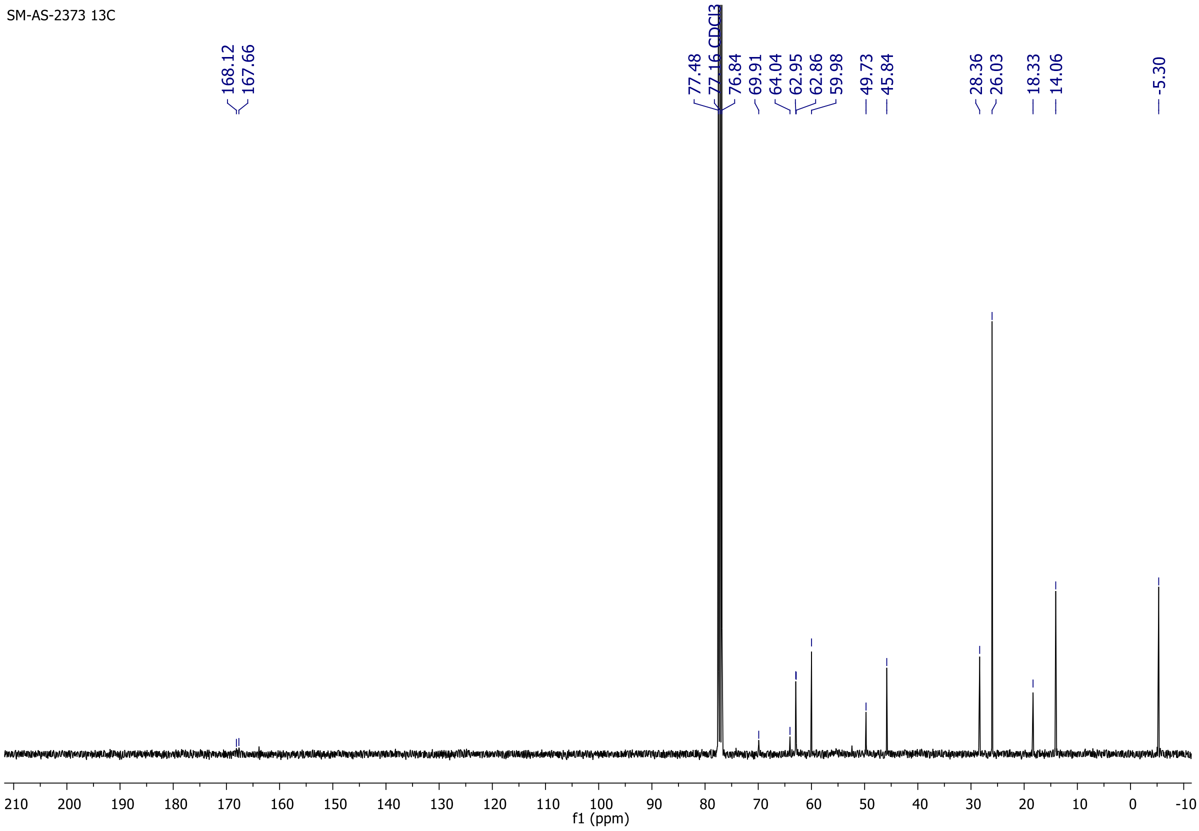

$^{1}H$ NMR of **3ab** (400 MHz, $CDCl_3$):

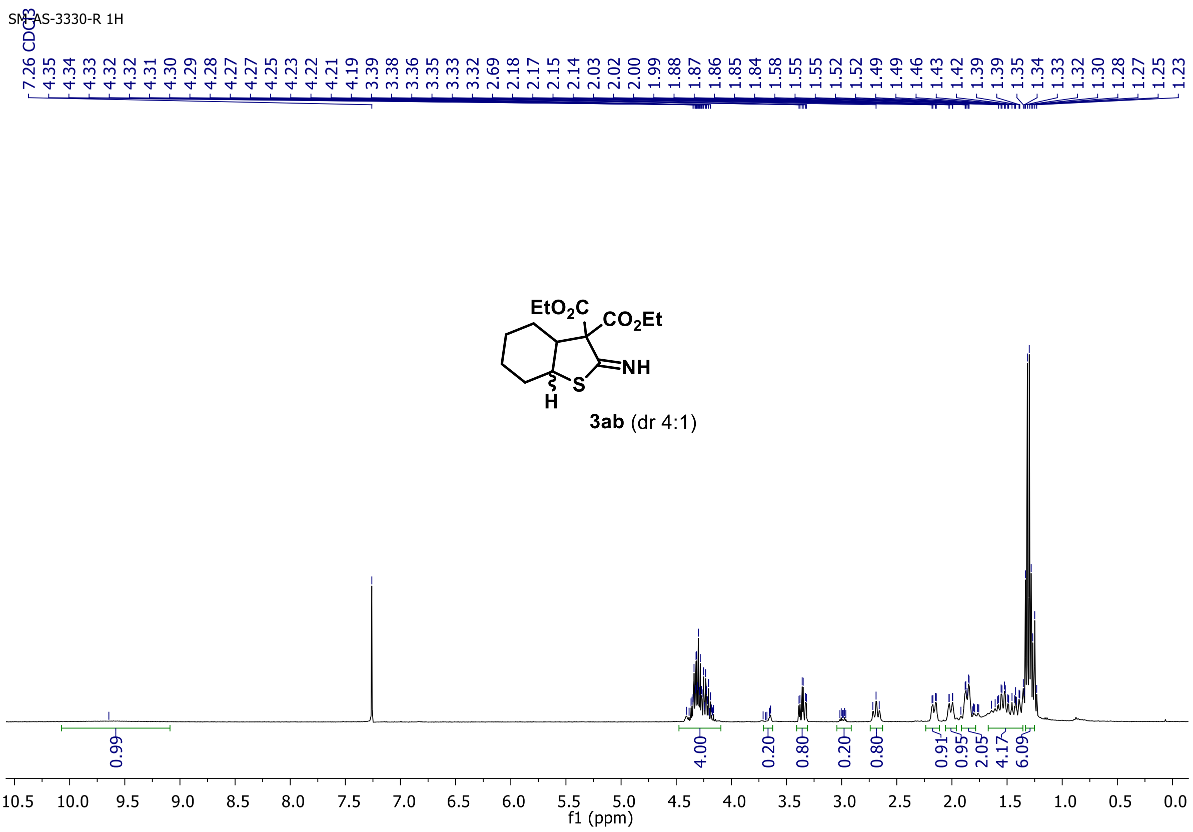


$^{13}C\{^{1}H\}$ NMR of **3ab** (101 MHz, $CDCl_3$):

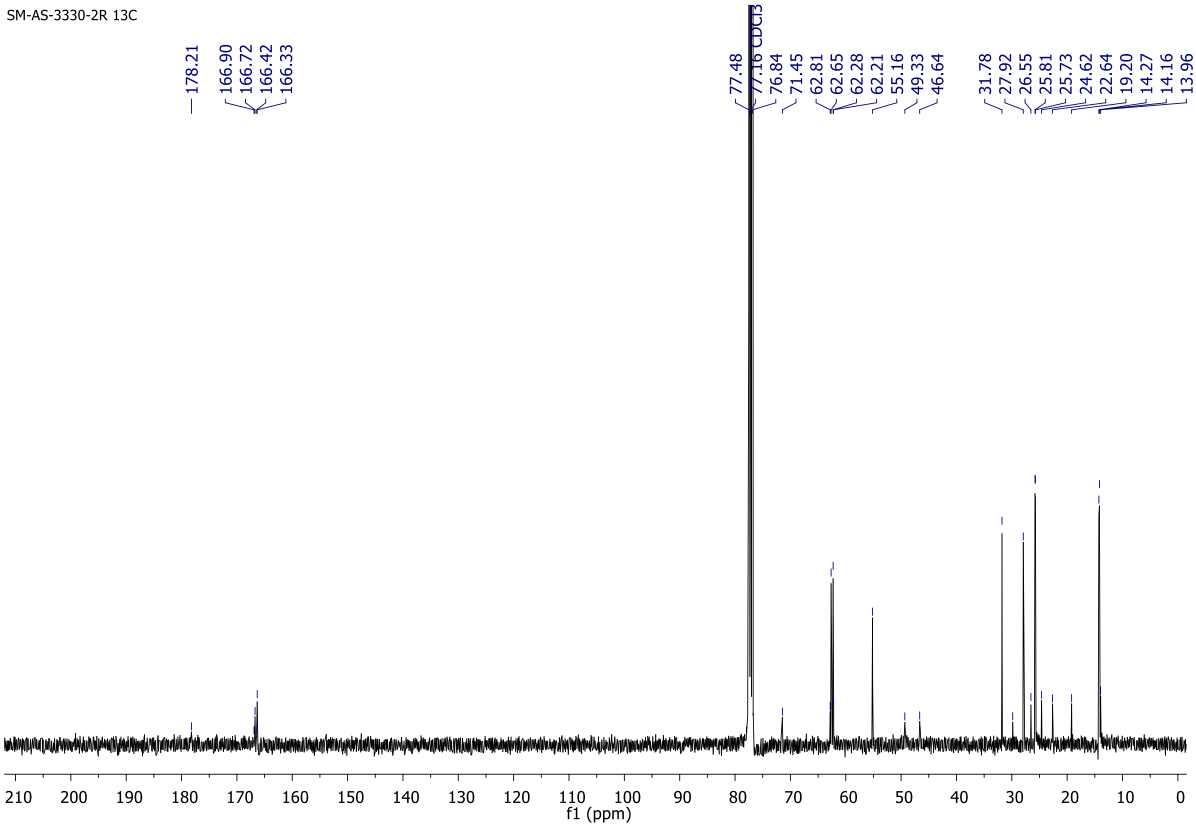

$^1$H NMR of **3ac** (400 MHz, $CDCl_3$):

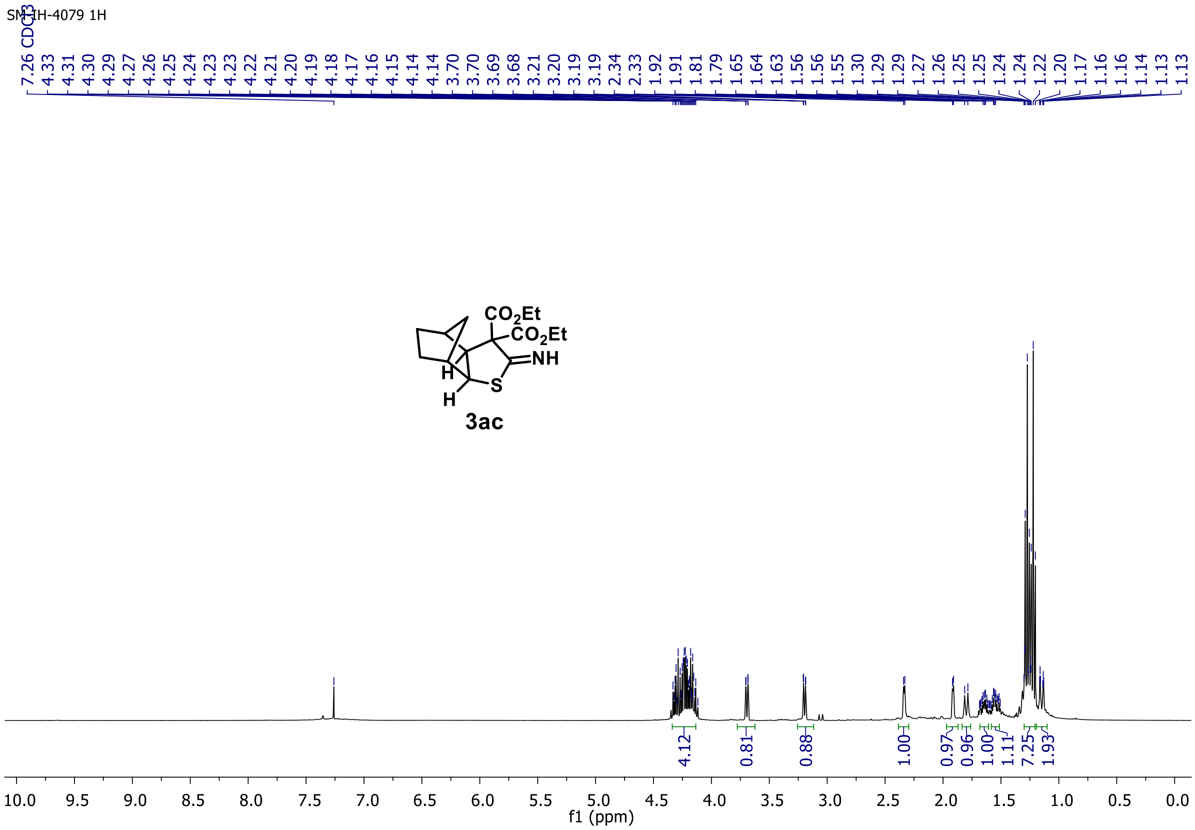


$^{13}$C{$^1$H} NMR of **3ac** (101 MHz, $CDCl_3$):

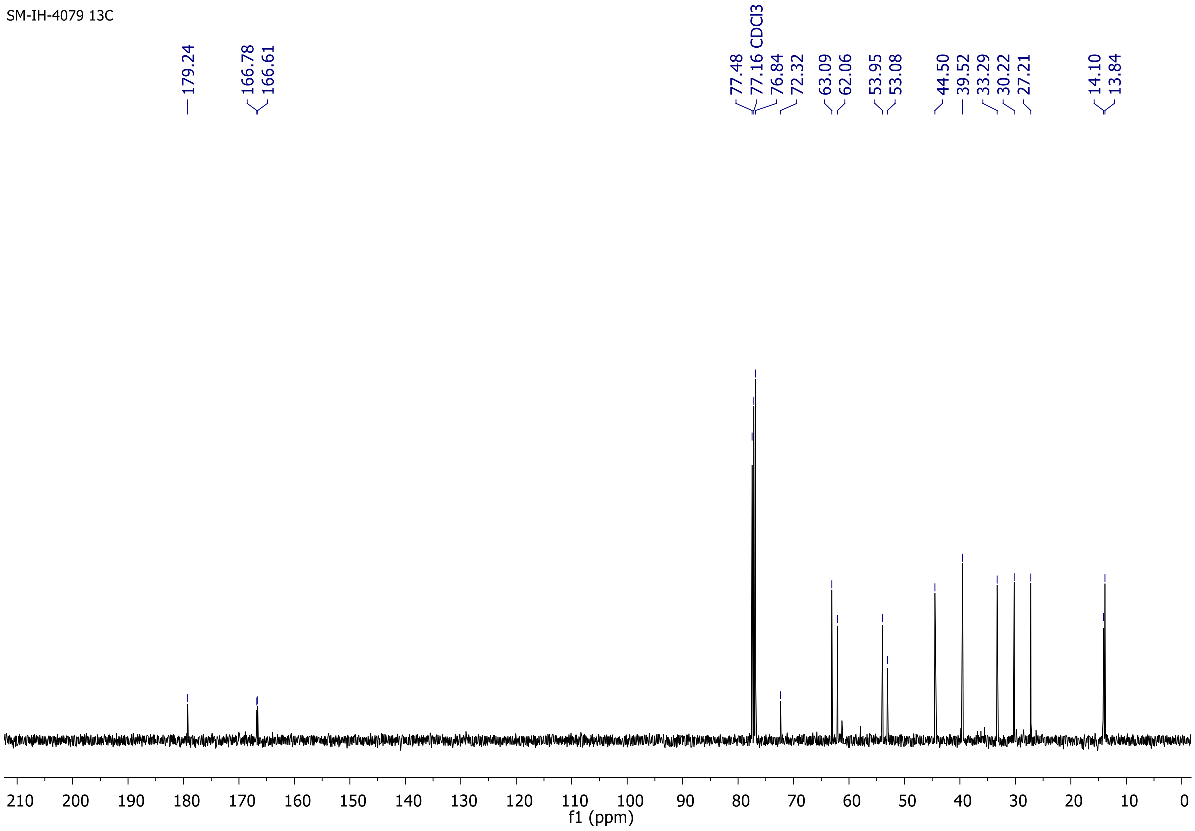

$^1$H-$^1$H COSY NMR of **3ac**:

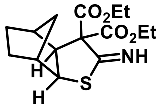


**3ac**

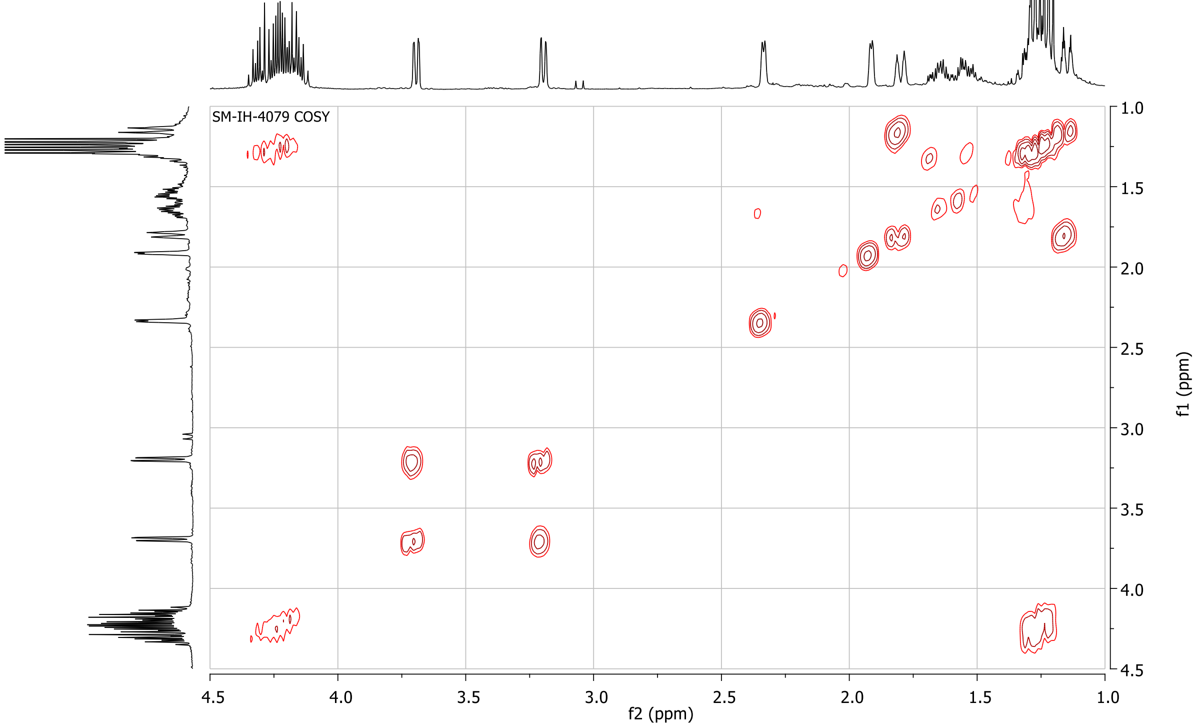


$^1$H-$^{13}$C HSQC NMR of **3ac**:

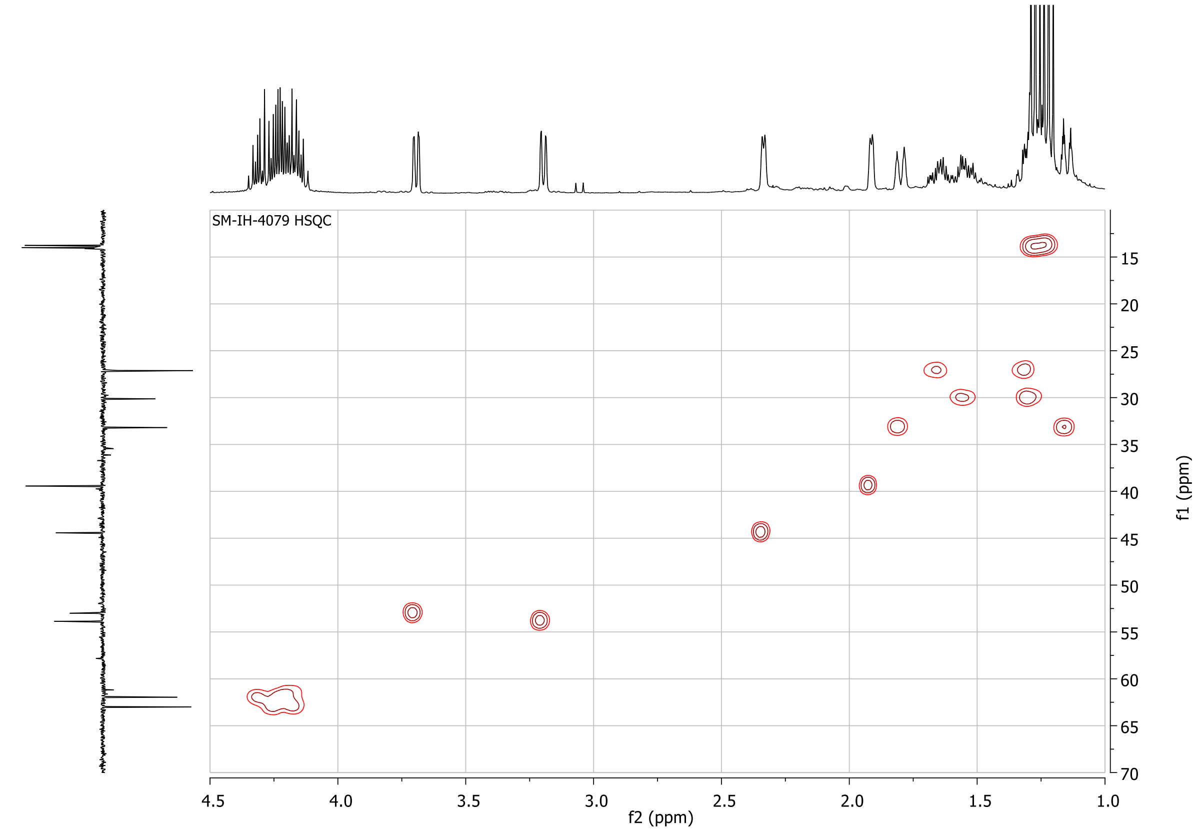

$^{1}$H-$^{1}$H NOESY NMR of **3ac**:

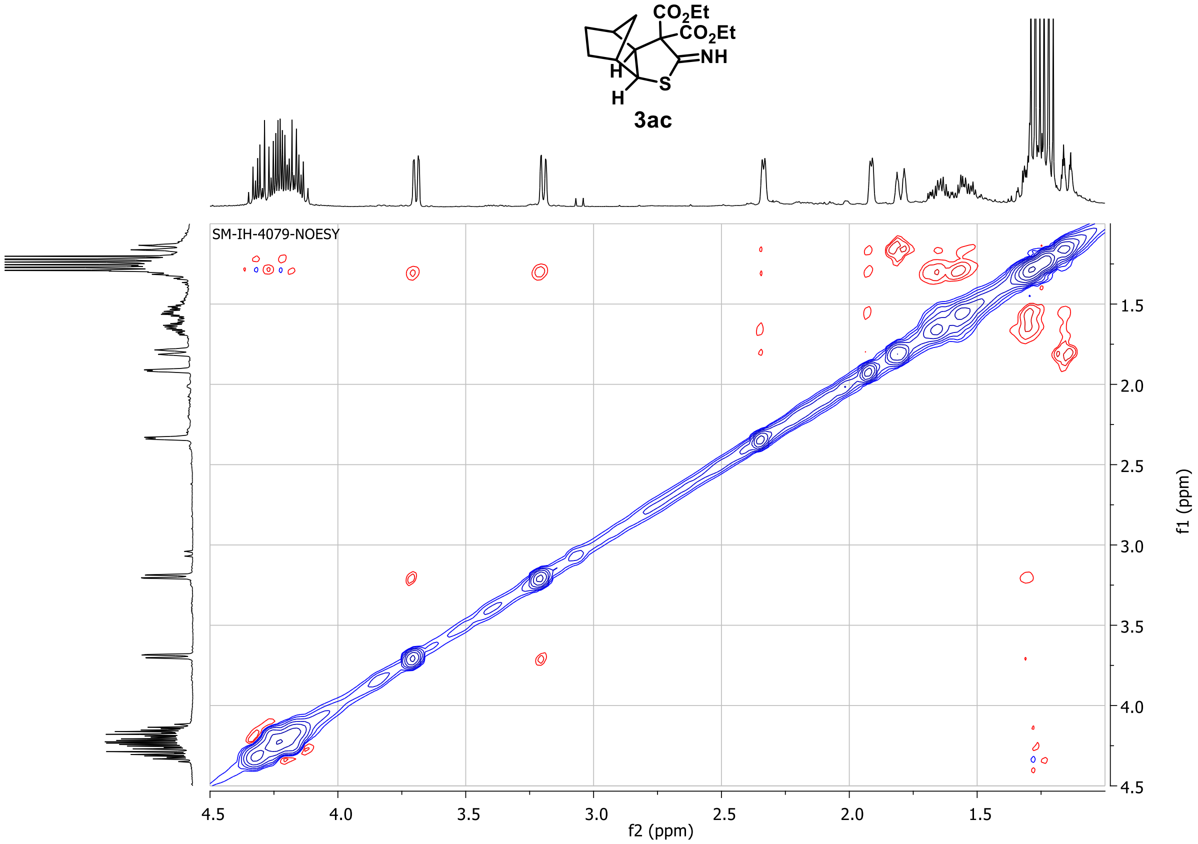

**3ac**

$^{1}H$ NMR of **3ad** (400 MHz, $CDCl_3$):

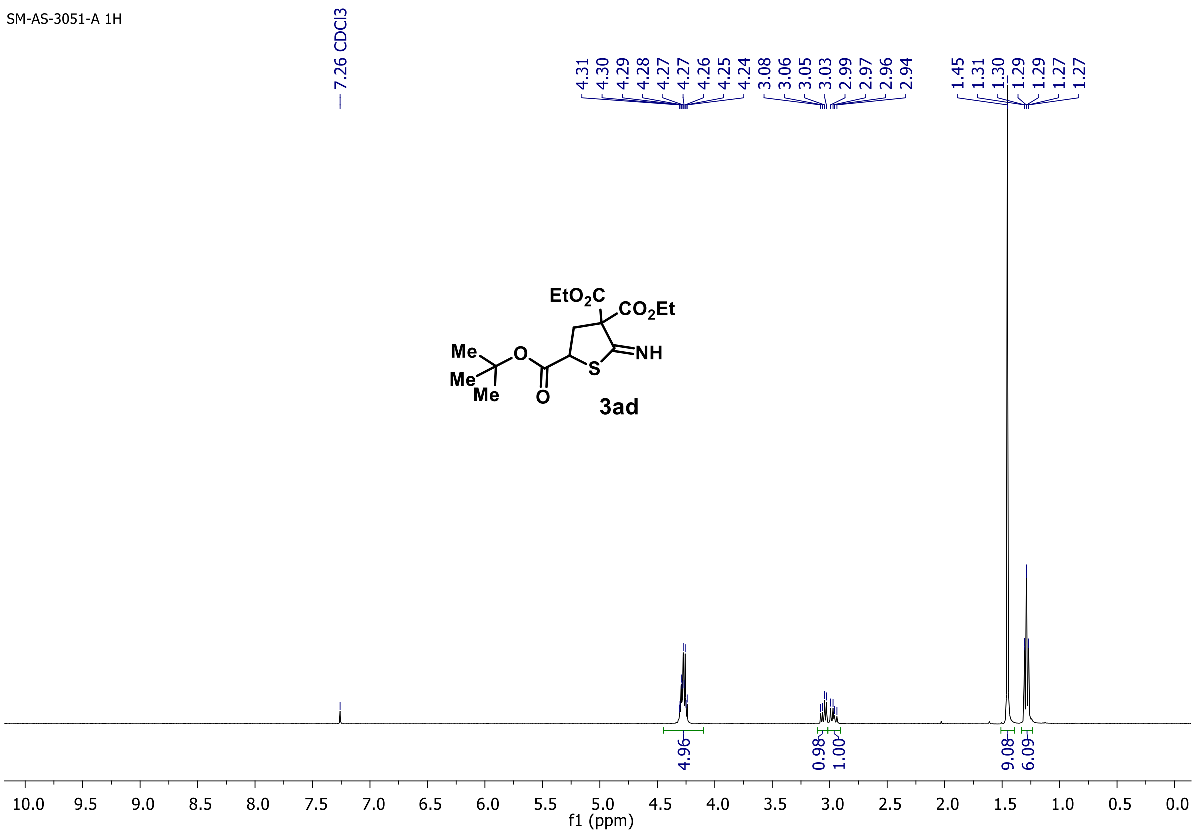


$^{13}C\{^{1}H\}$ NMR of **3ad** (101 MHz, $CDCl_3$):

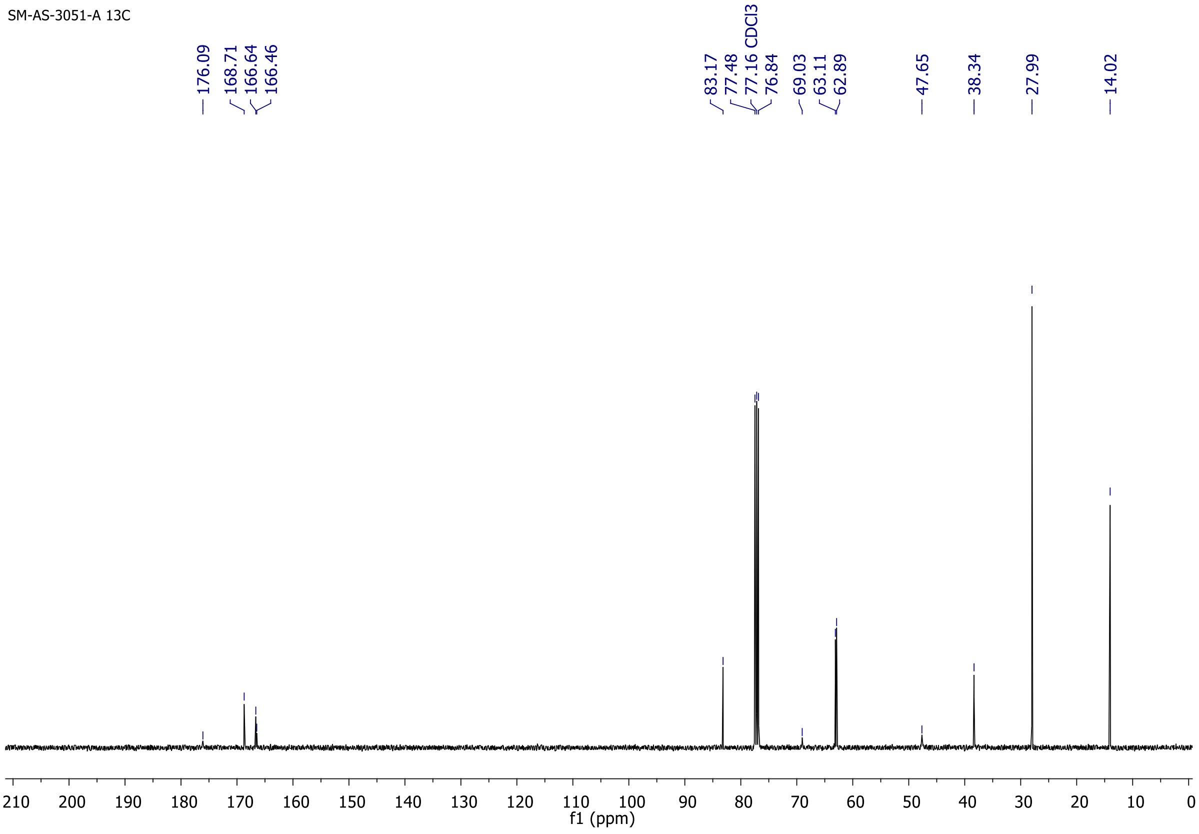

$^{1}H$ NMR of **3ae** (400 MHz, $CDCl_3$):

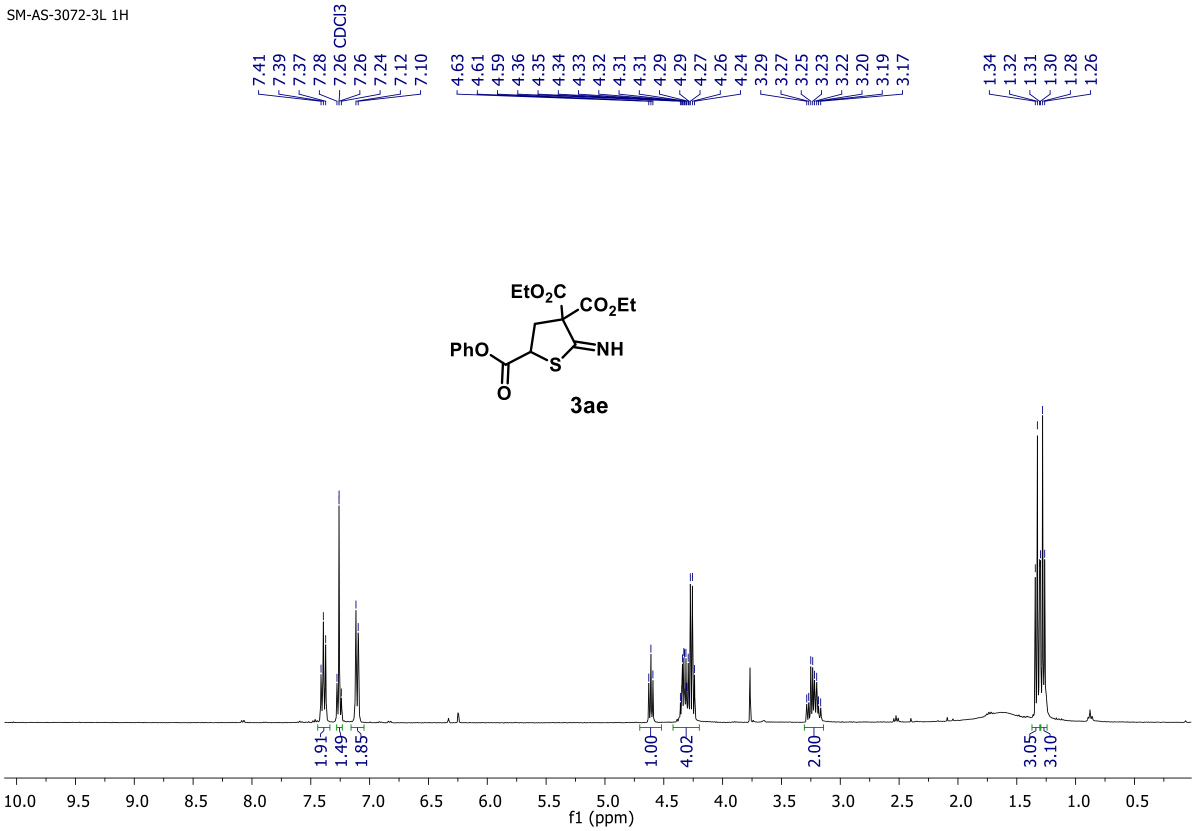


$^{13}C\{^{1}H\}$ NMR of **3ae** (101 MHz, $CDCl_3$):

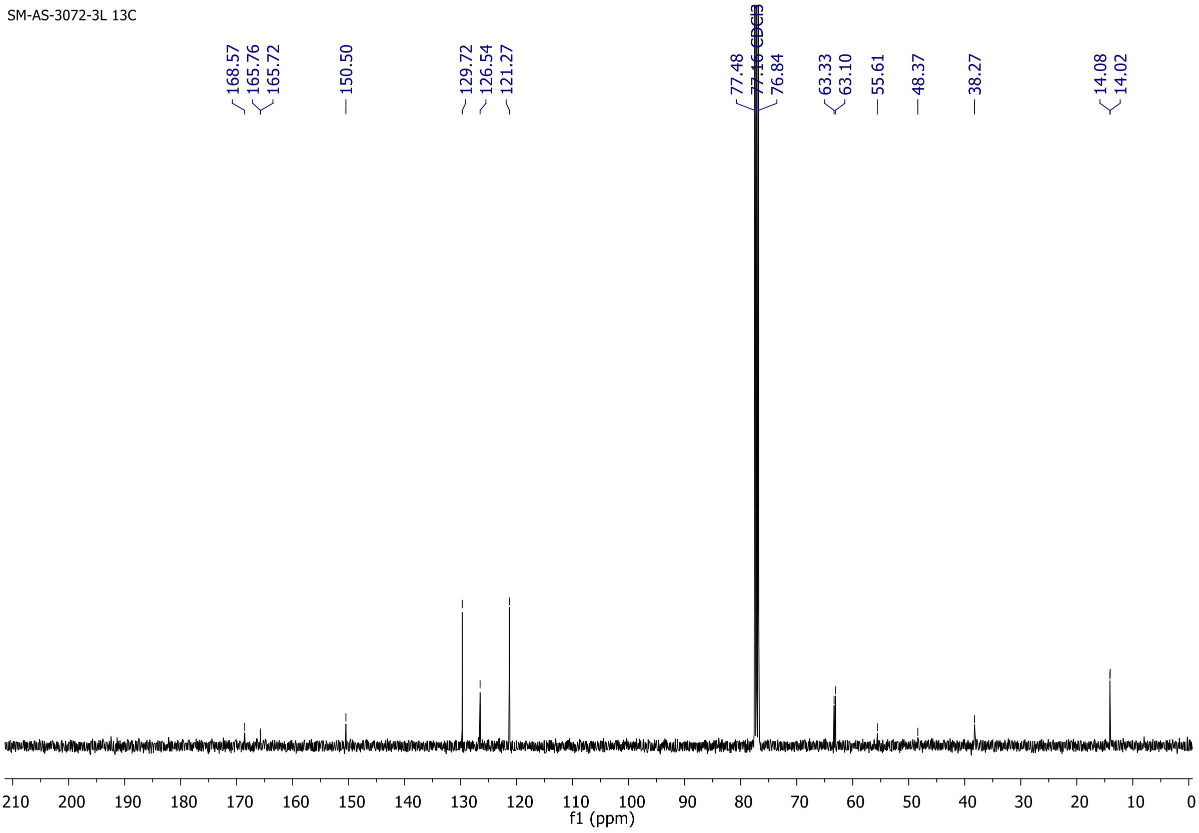

$^{1}$H NMR of **3af** (400 MHz, $CDCl_3$):

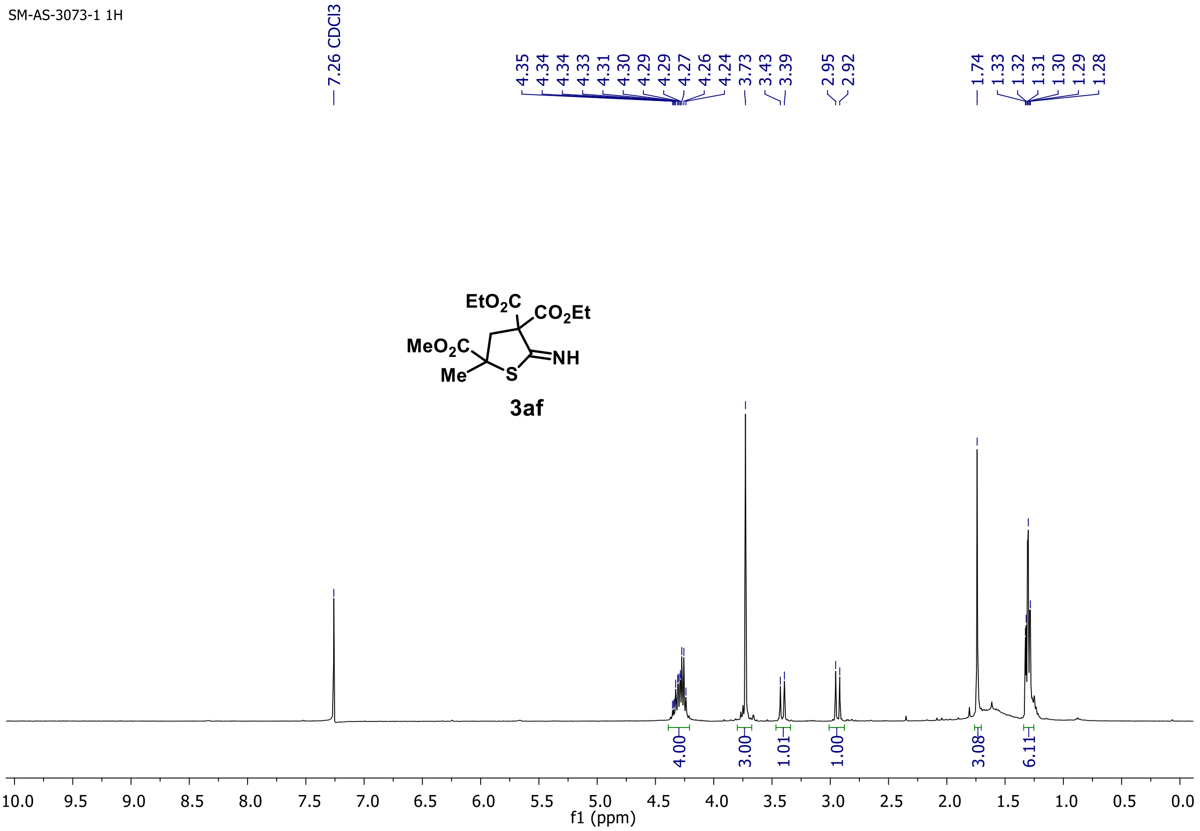


$^{13}$C{$^{1}$H} NMR of **3af** (101 MHz, $CDCl_3$):

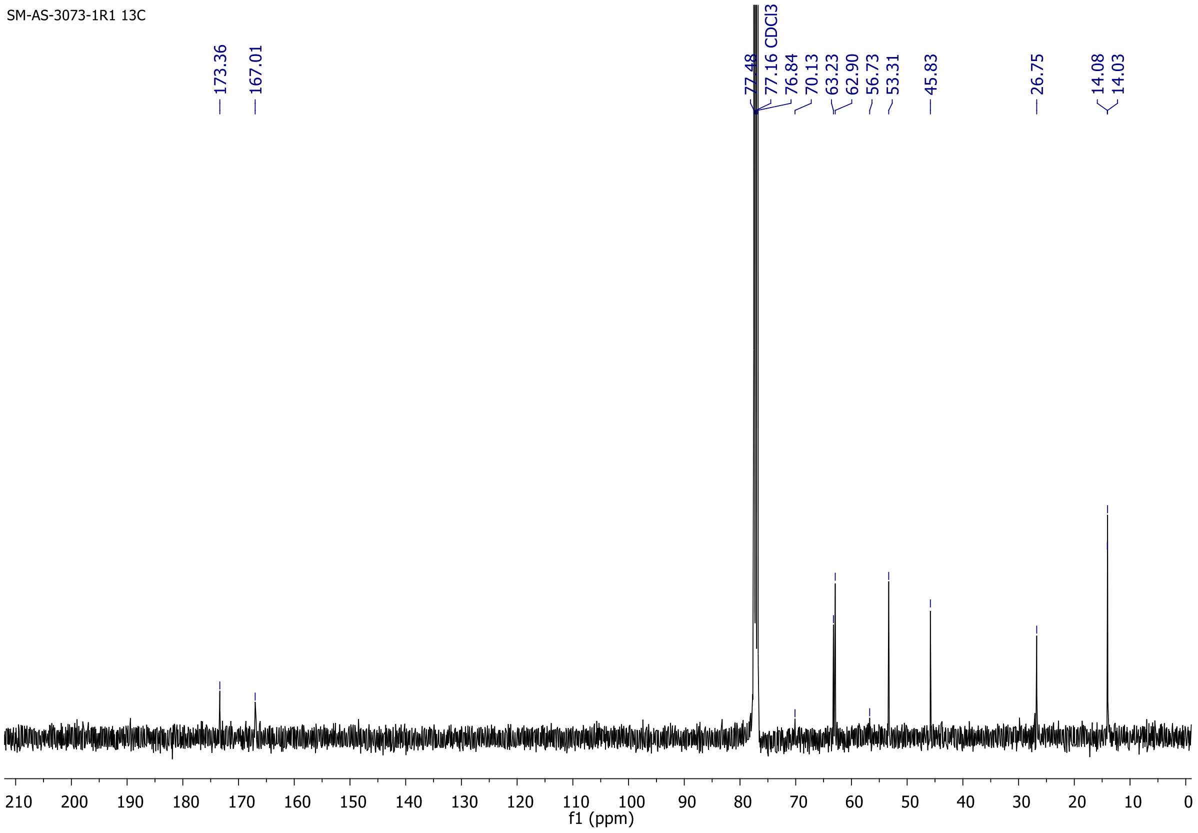

$^{1}$H NMR of **3ag** (400 MHz, $CDCl_3$):

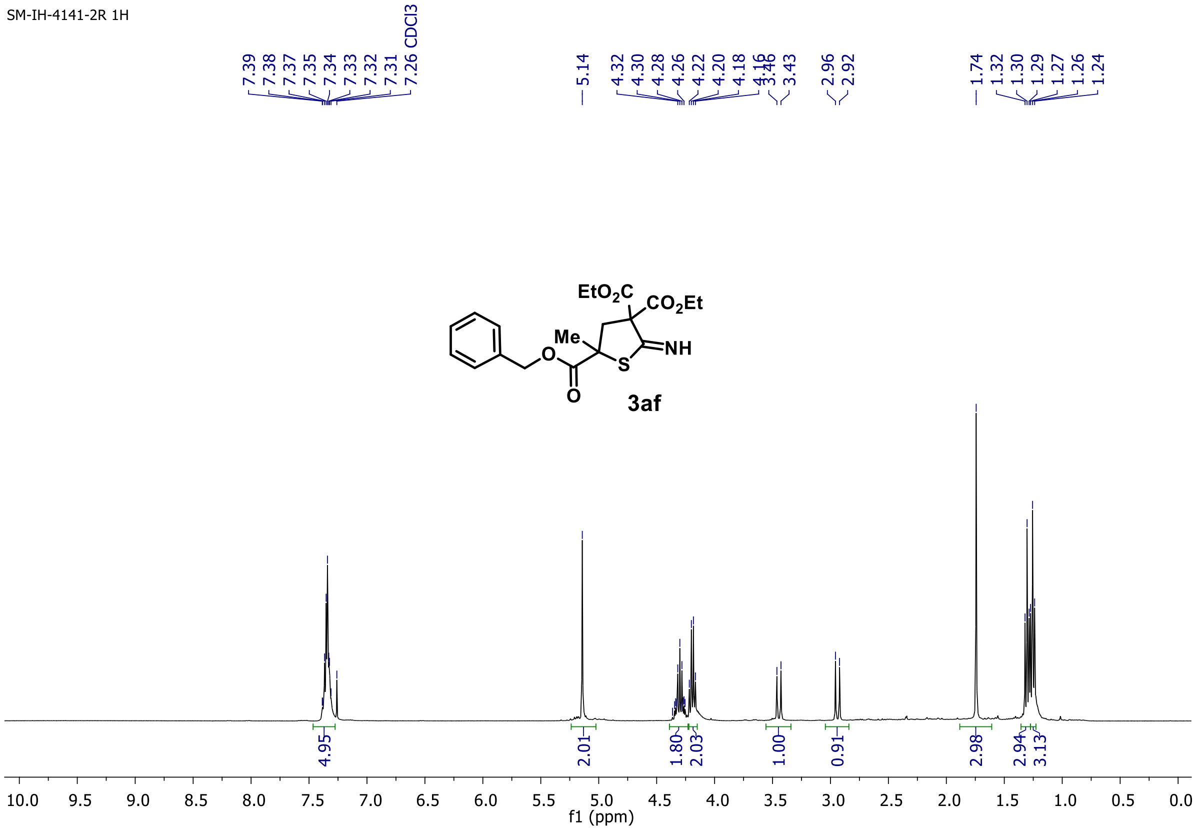


$^{13}$C{$^{1}$H} NMR of **3af** (101 MHz, $CDCl_3$):

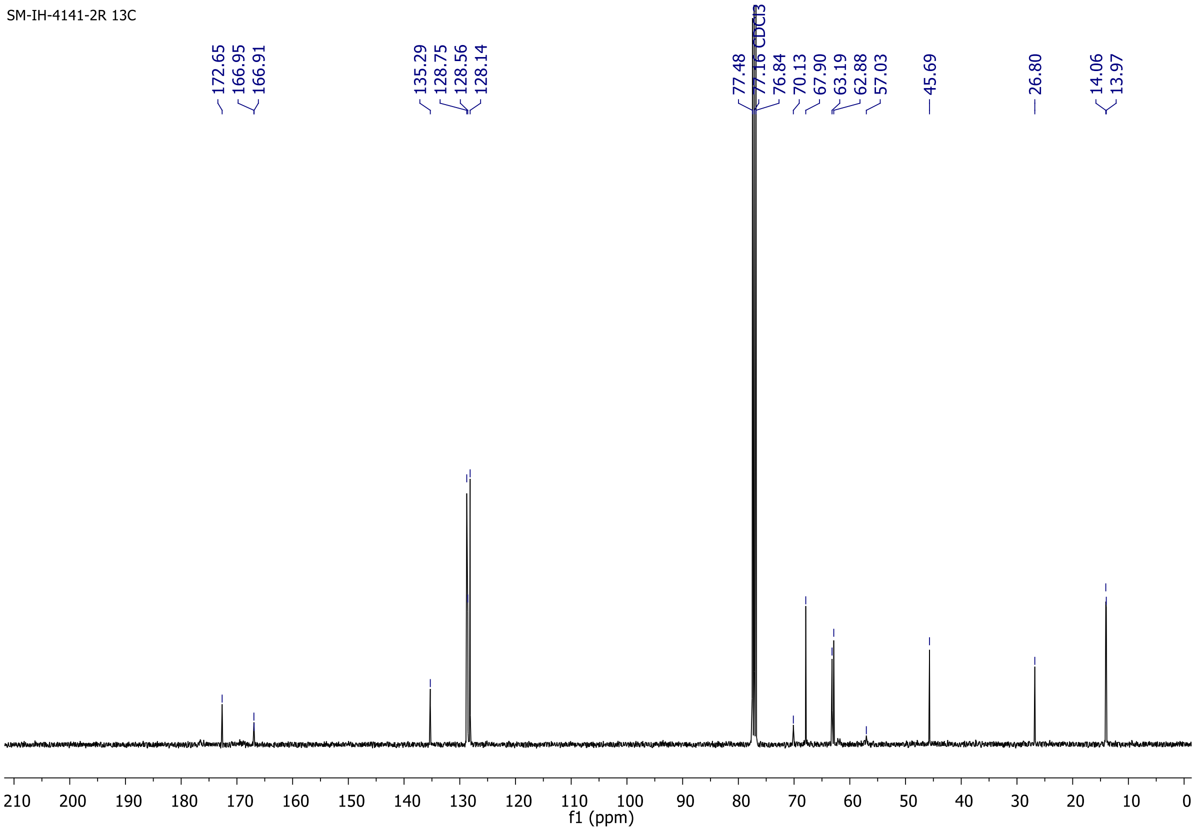

$^{1}H$ NMR of **3ah** (400 MHz, DMSO-d$_6$):

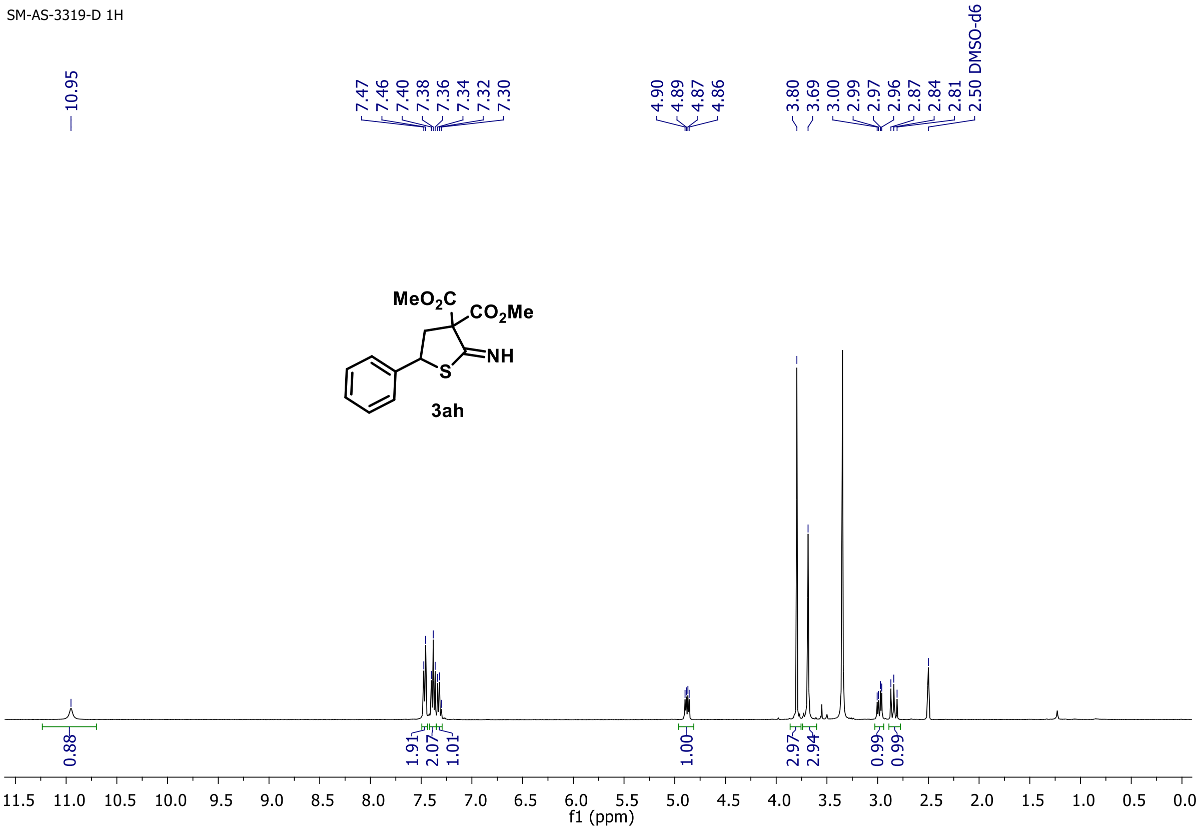


$^{13}C\{^{1}H\}$ NMR of **3ah** (101 MHz, DMSO-d$_6$):

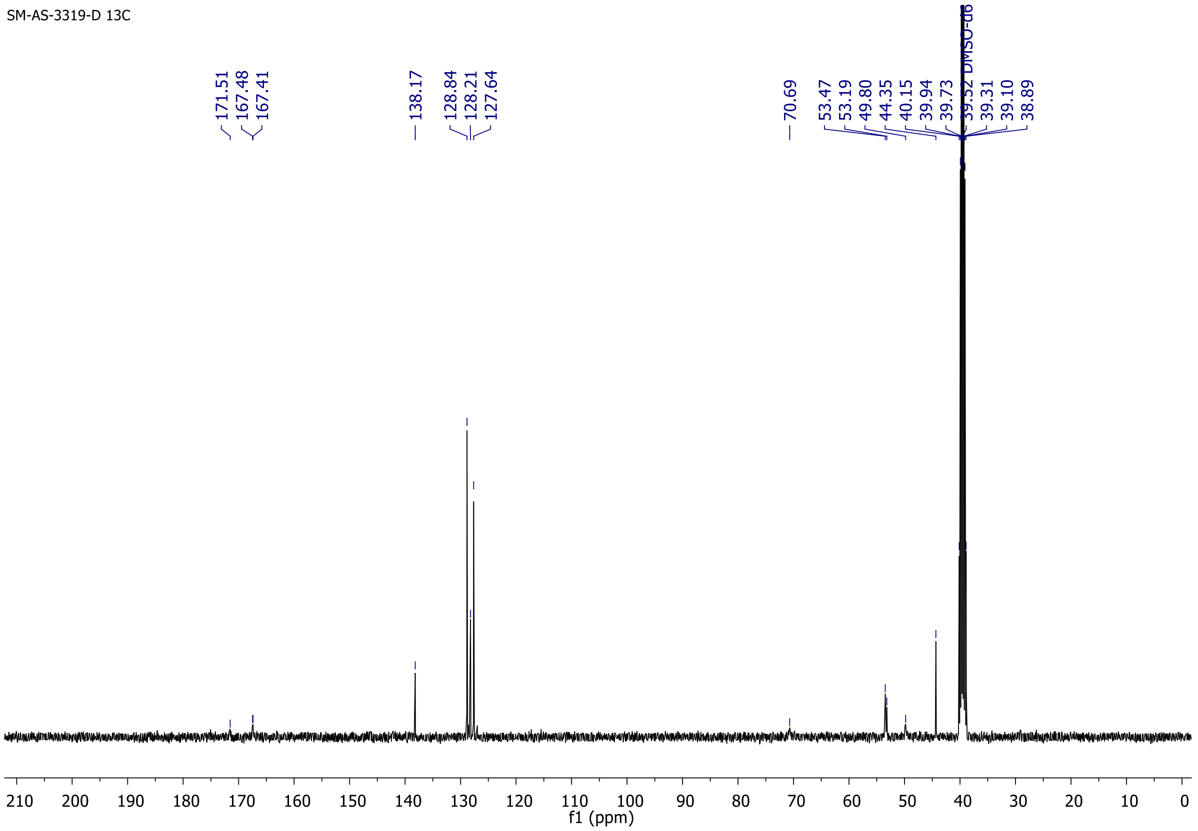

$^{1}H$ NMR of **3ai** (400 MHz, DMSO-d$_6$):

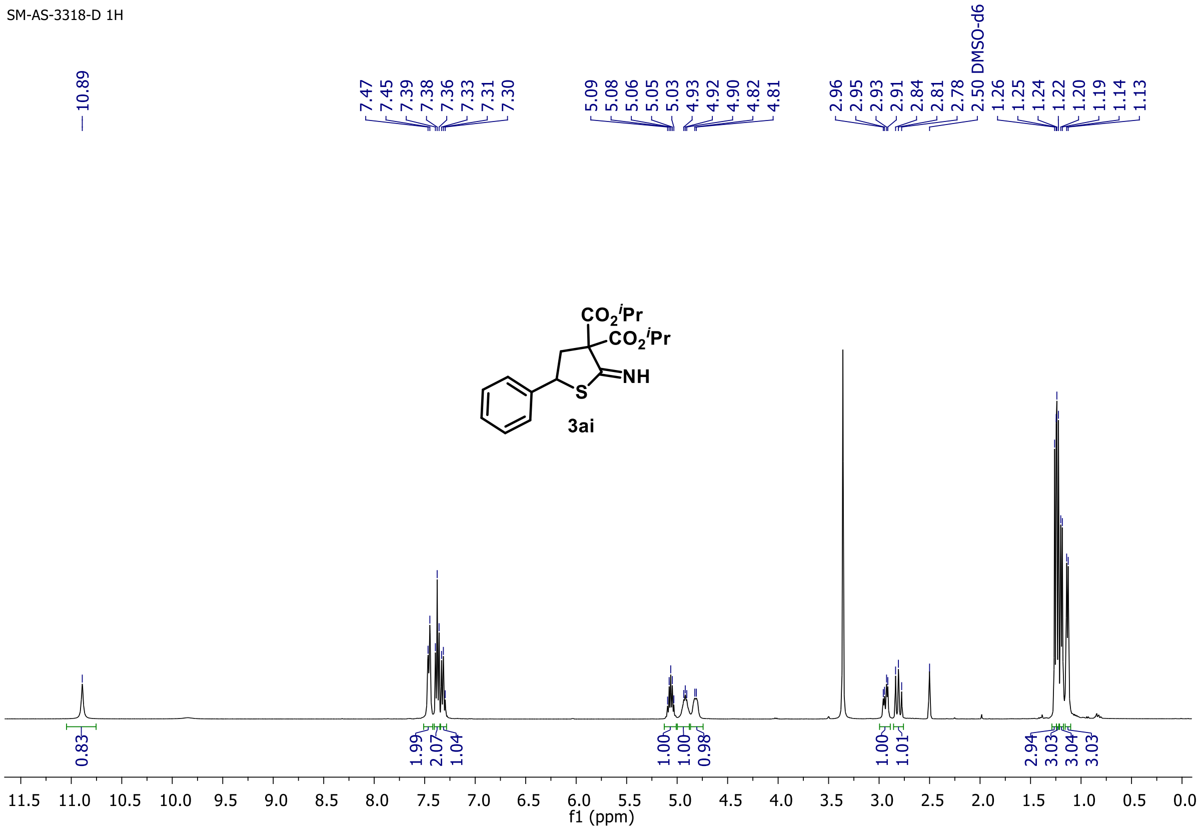


$^{13}C\{^{1}H\}$ NMR of **3ai** (101 MHz, DMSO-d$_6$):

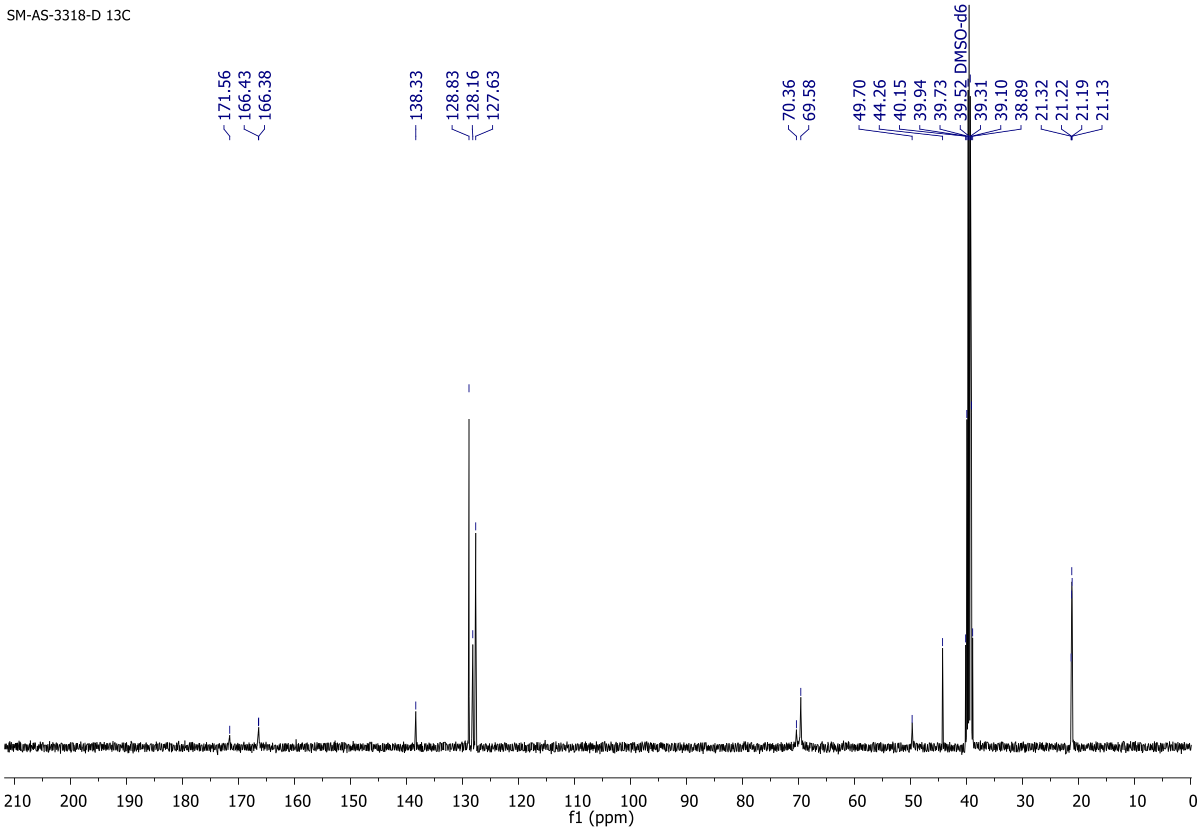

$^1$H NMR of **3aj** (400 MHz, $CDCl_3$):

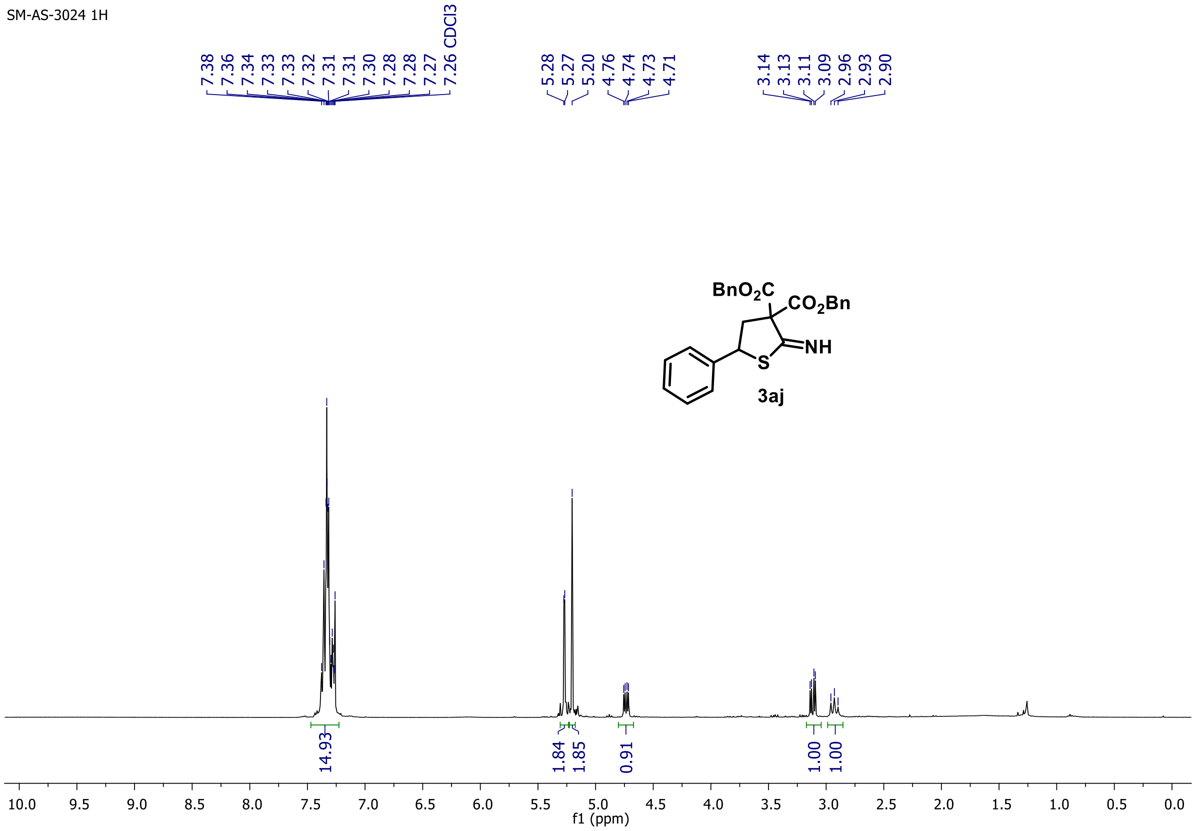


$^{13}$C{$^1$H} NMR of **3aj** (101 MHz, $CDCl_3$):

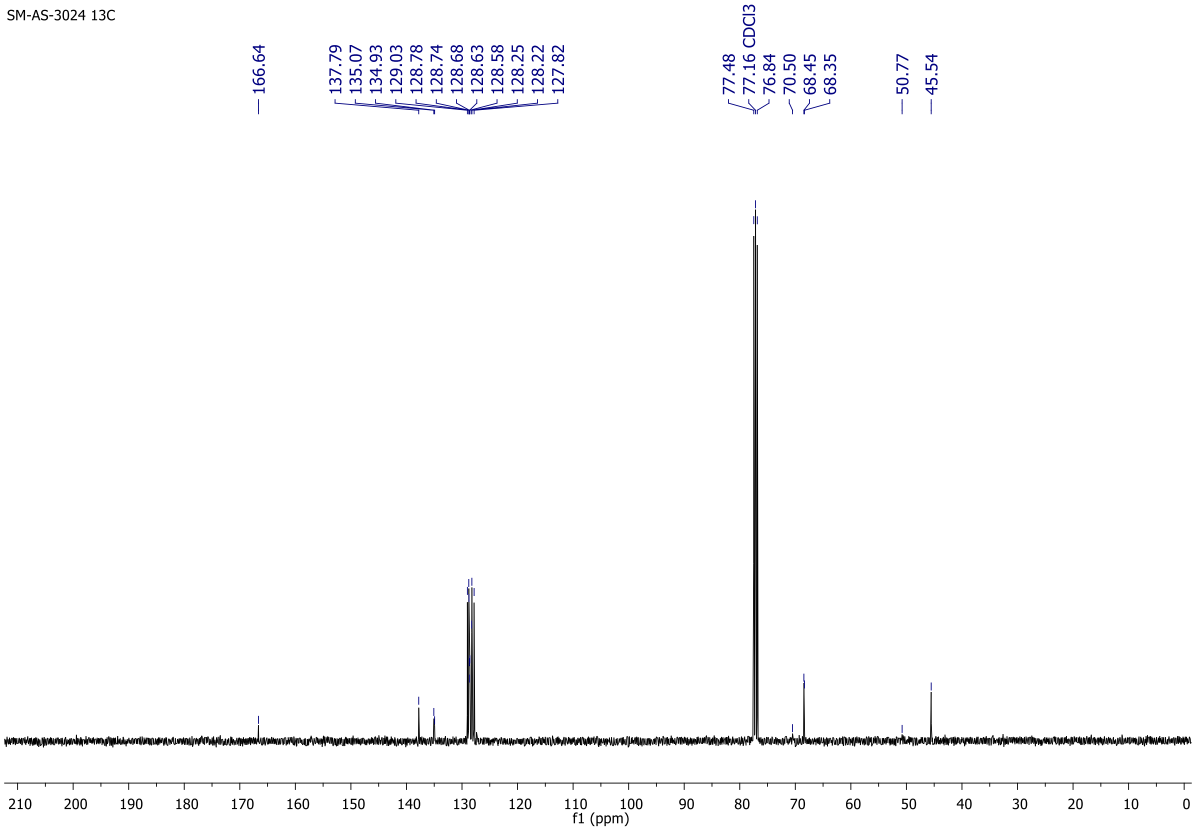

$^{1}$H NMR of **3ak** (400 MHz, $CDCl_3$):

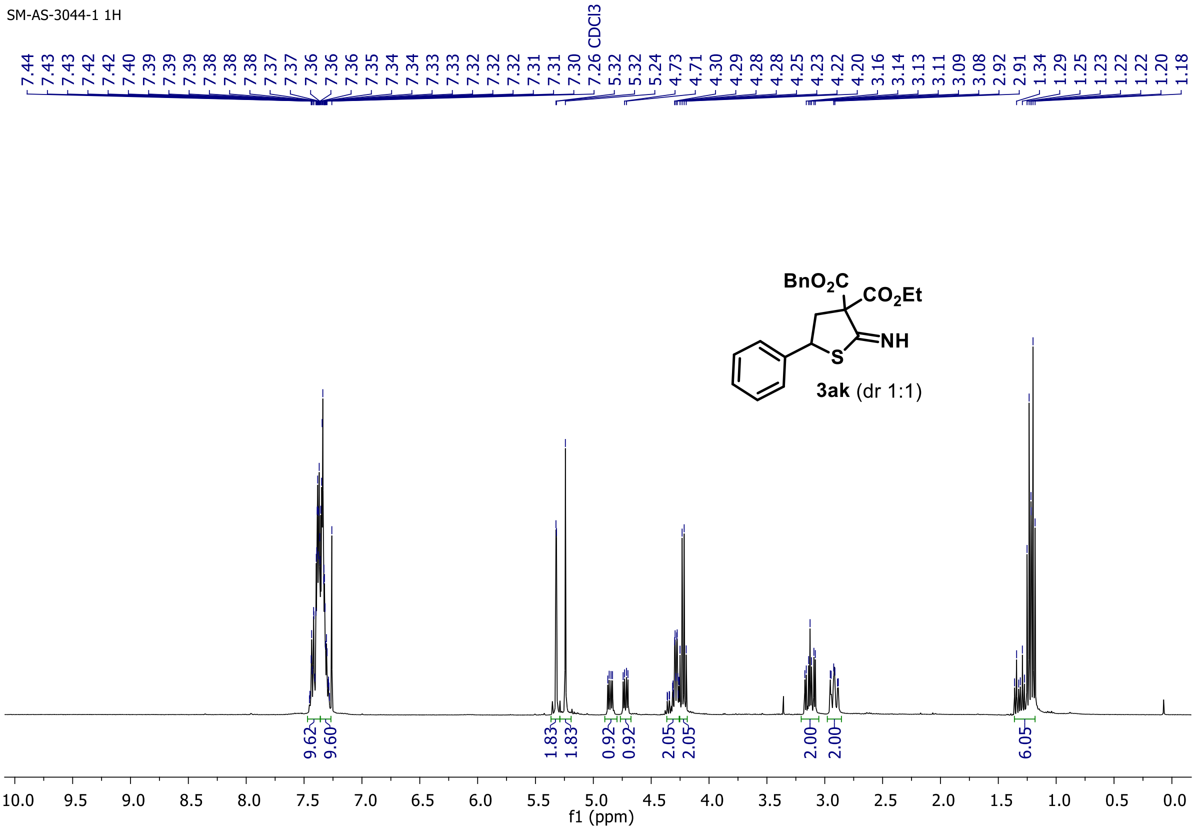


$^{13}$C{$^{1}$H} NMR of **3ak** (101 MHz, $CDCl_3$):

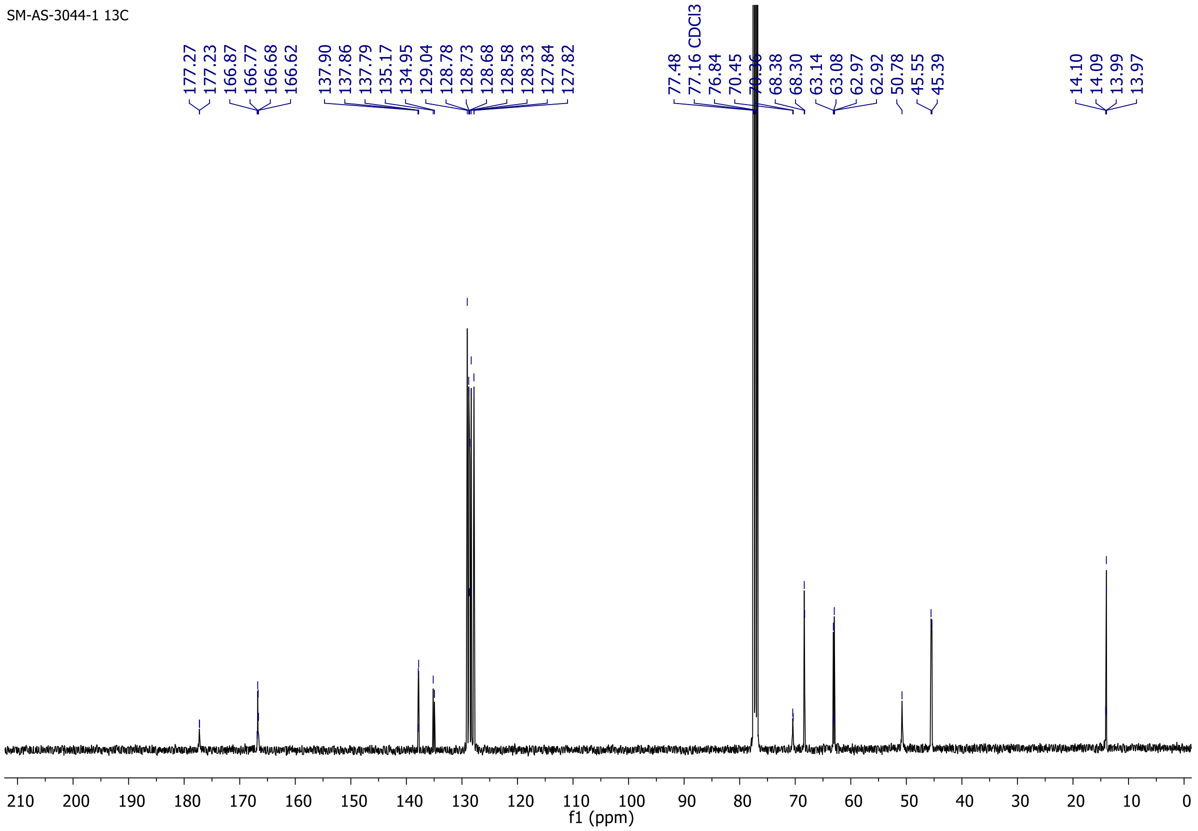

$^{1}H$ NMR of **3al** (400 MHz, $CDCl_3$):

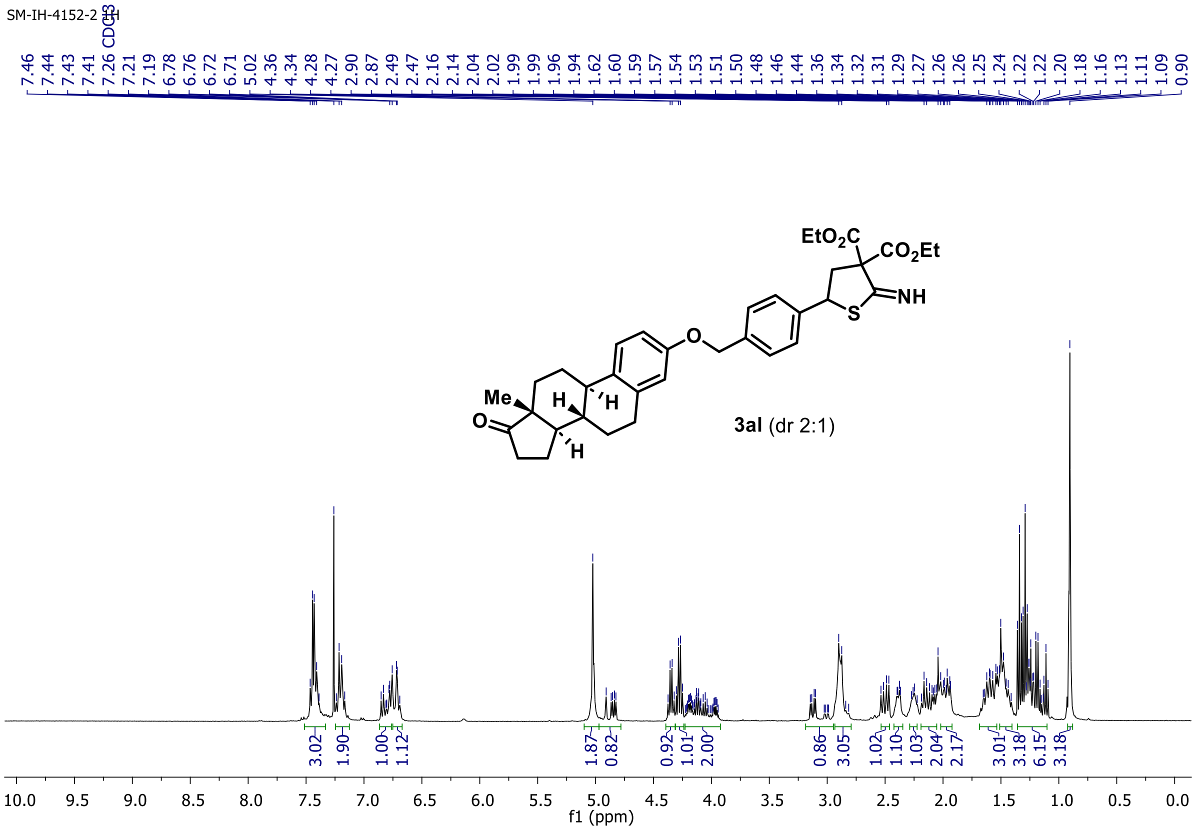


$^{13}C\{^{1}H\}$ NMR of **3al** (101 MHz, $CDCl_3$):

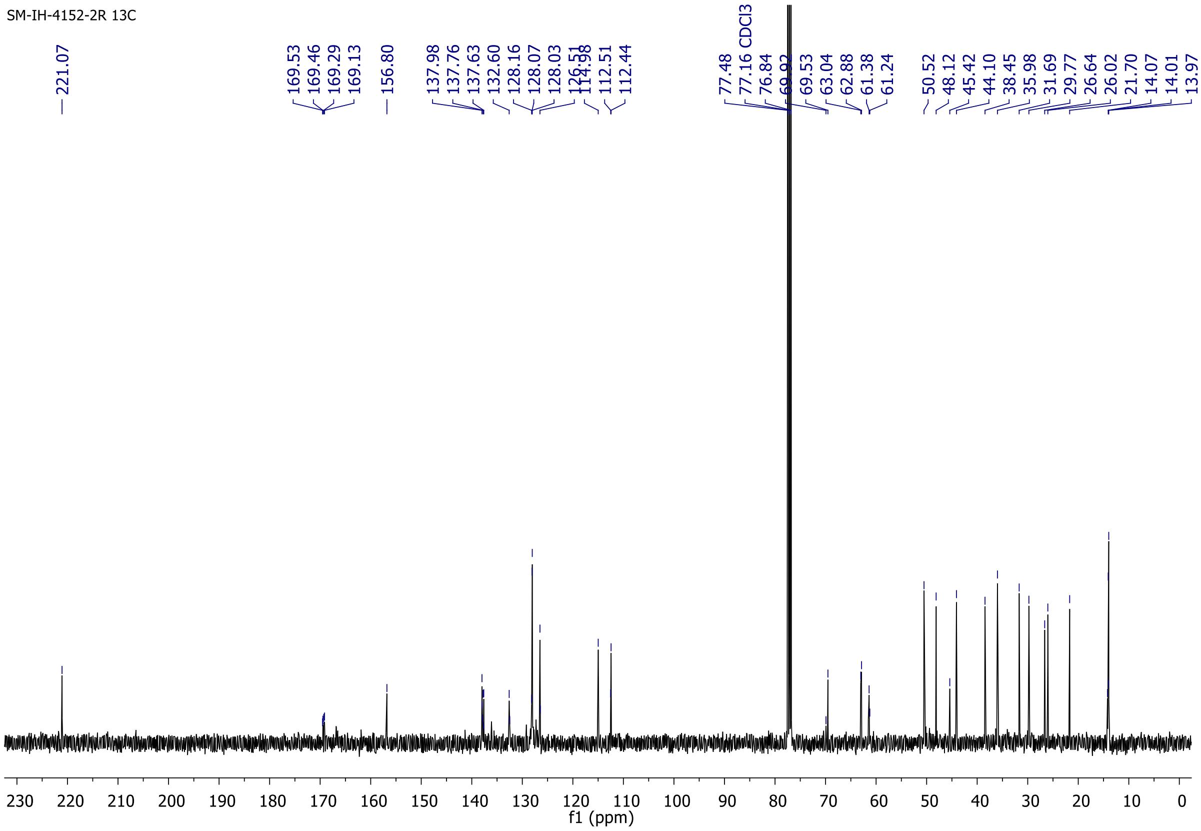

$^{1}H$ NMR of **3am** (400 MHz, $CDCl_3$):

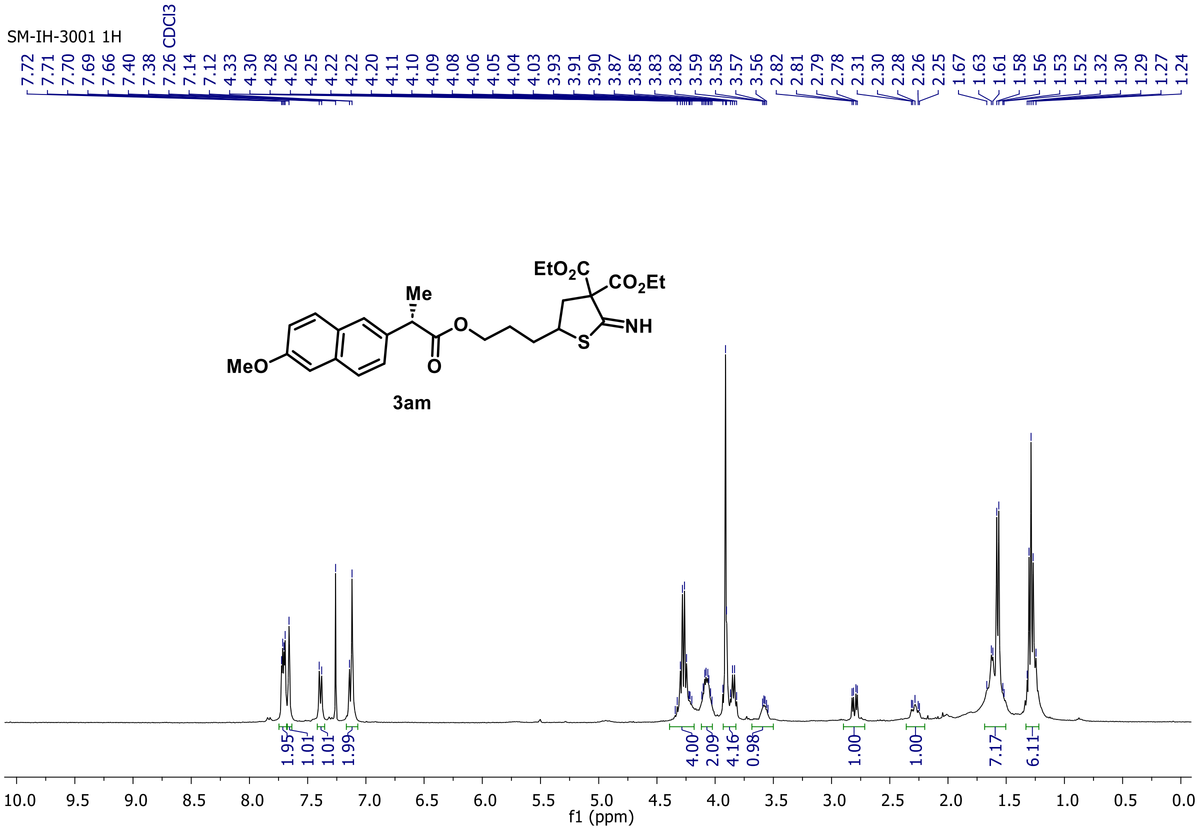


$^{13}C\{^{1}H\}$ NMR of **3am** (101 MHz, $CDCl_3$):

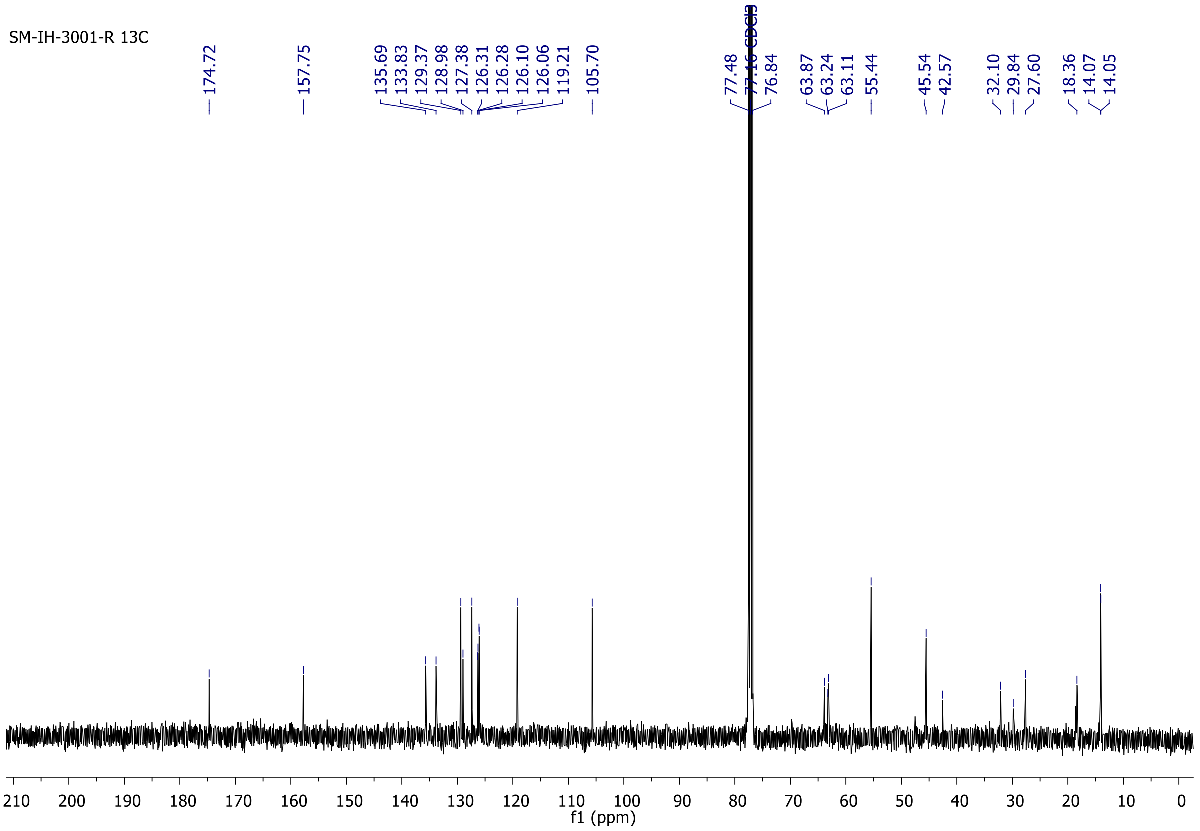

$^{1}H$ NMR of **3an** (400 MHz, $CDCl_3$):

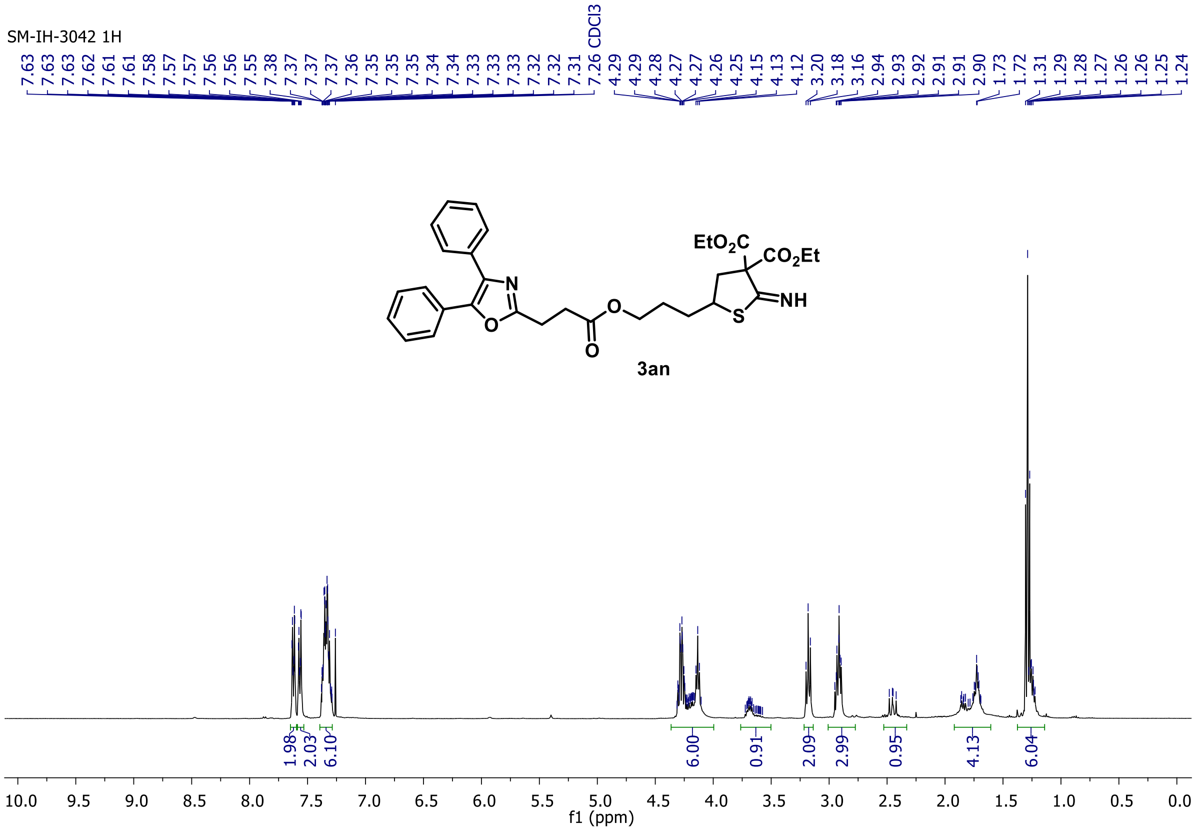


$^{13}C\{^{1}H\}$ NMR of **3an** (101 MHz, $CDCl_3$):

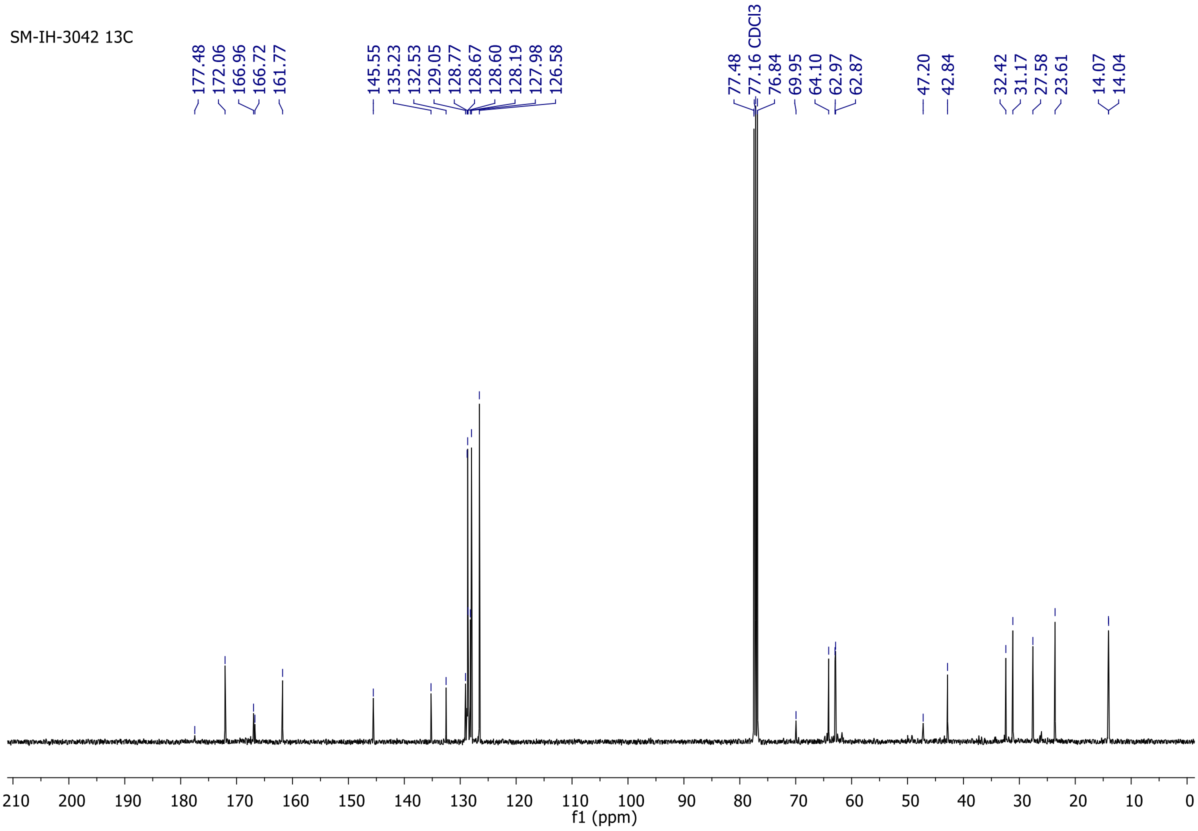

$^1$H NMR of **3ao** (400 MHz, $CDCl_3$):

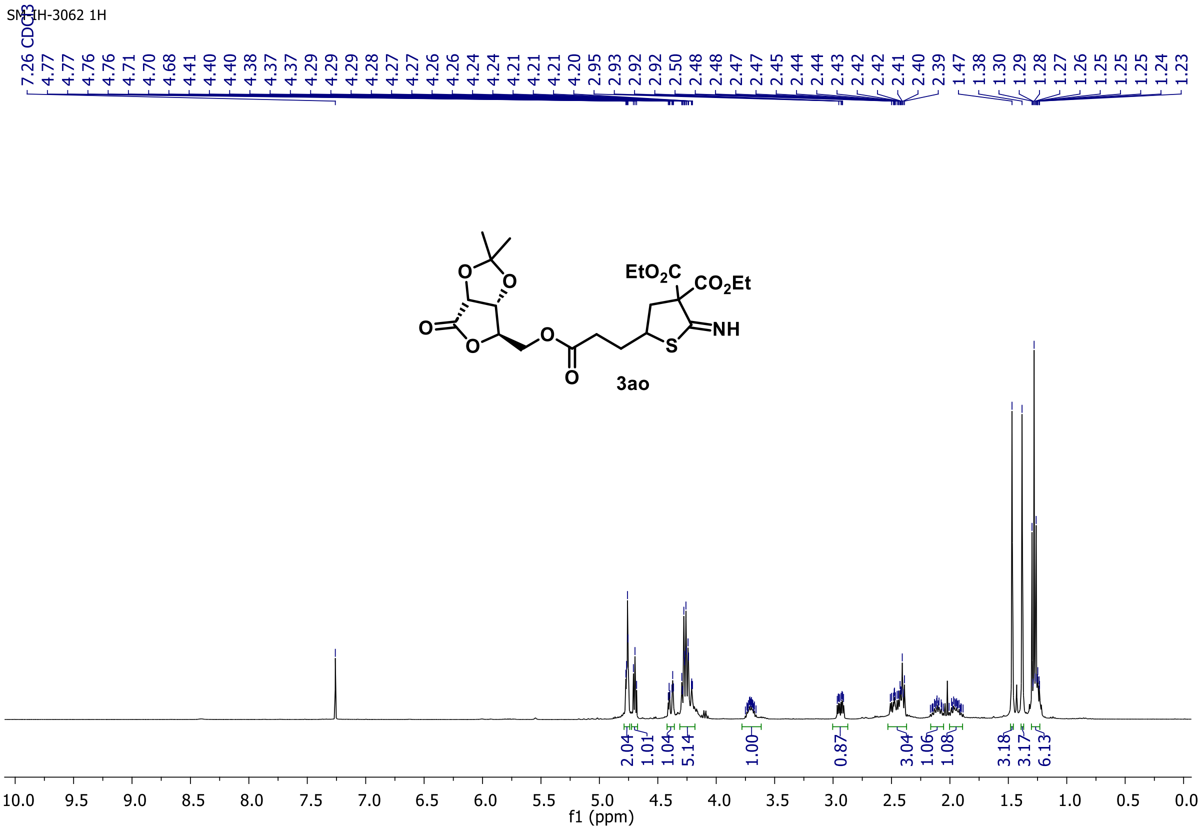


$^{13}C\{^1H\}$ NMR of **3ao** (101 MHz, $CDCl_3$):

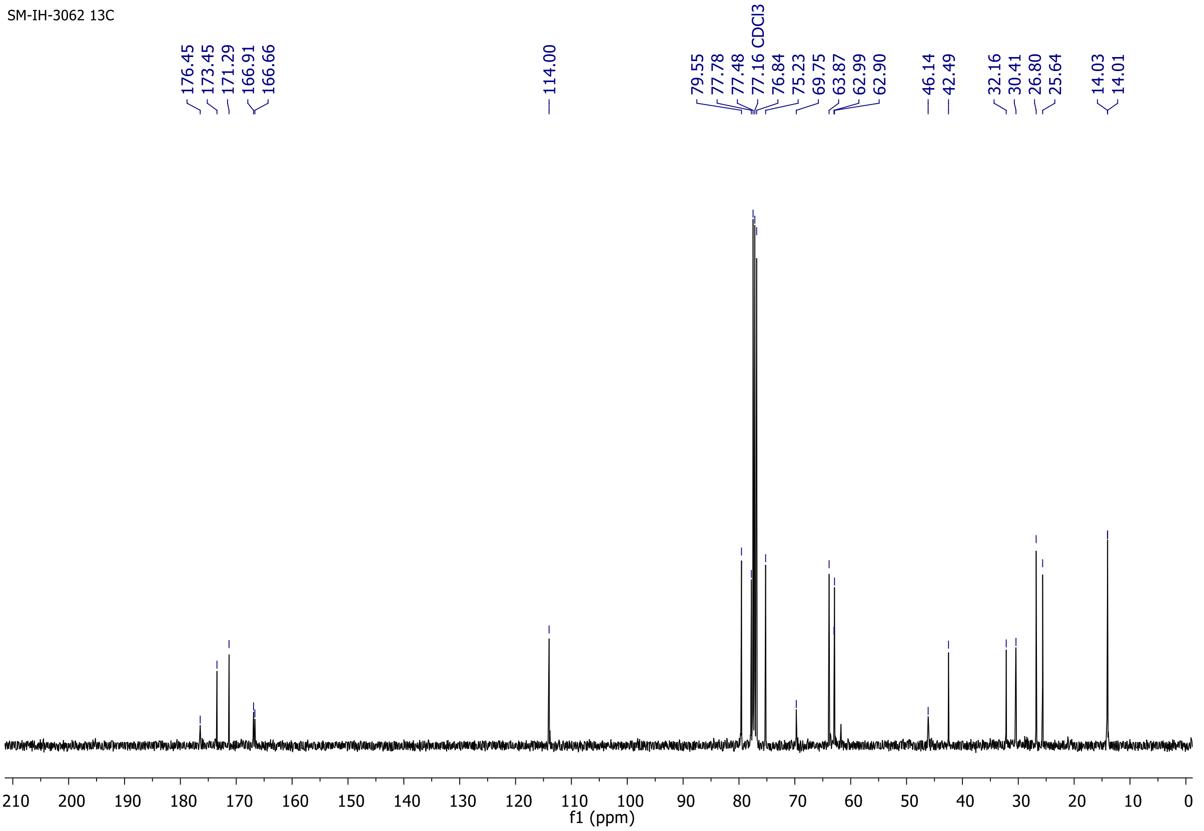

$^{1}H$ NMR of **4a** (400 MHz, $CDCl_3$):

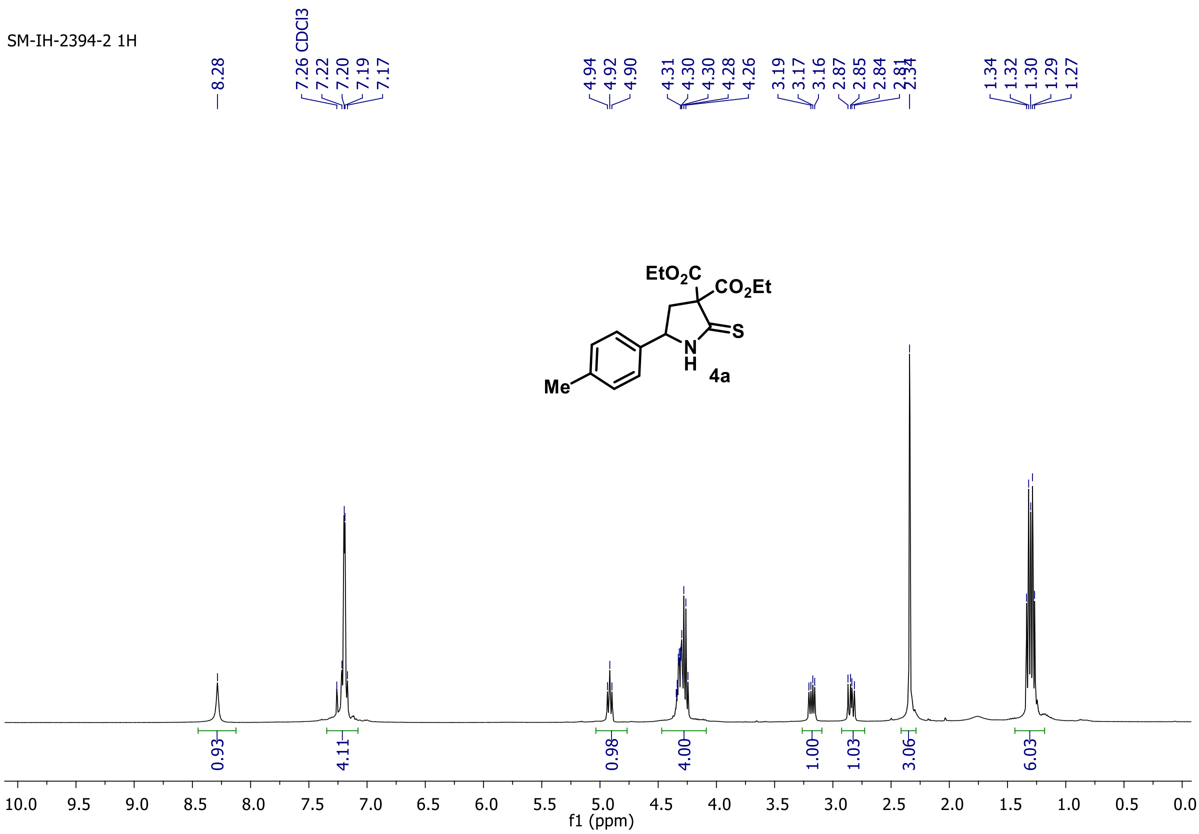


$^{13}C\{^{1}H\}$ NMR of **4a** (101 MHz, $CDCl_3$):

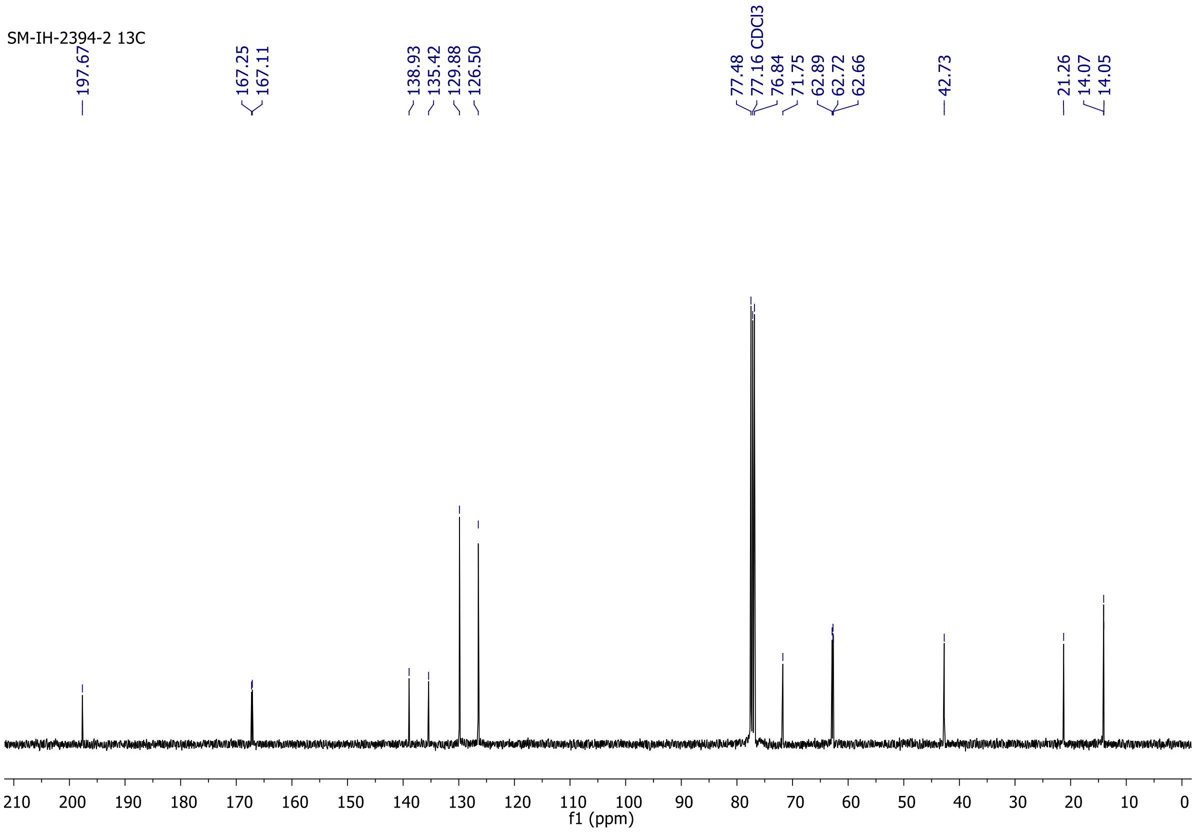

$^{1}$H NMR of **4b** (400 MHz, $CDCl_3$):

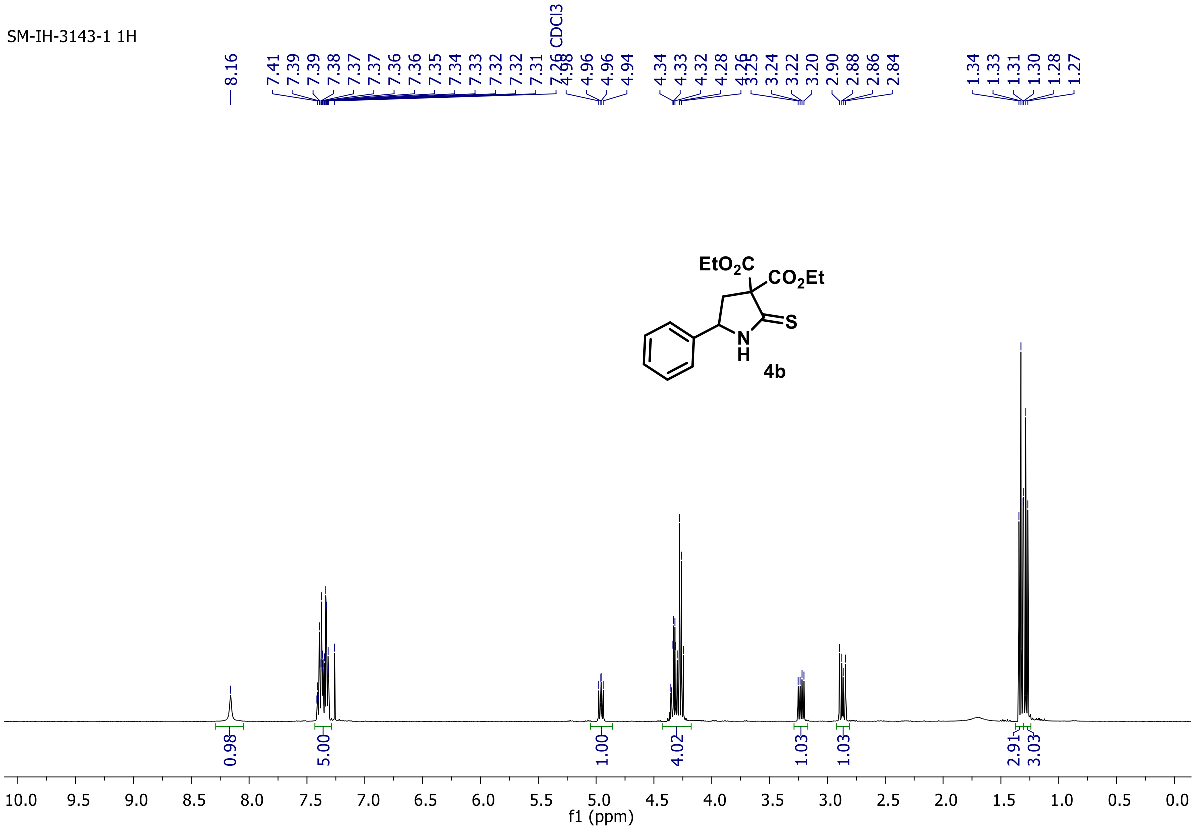


$^{13}$C{$^{1}$H} NMR of **4b** (101 MHz, $CDCl_3$):

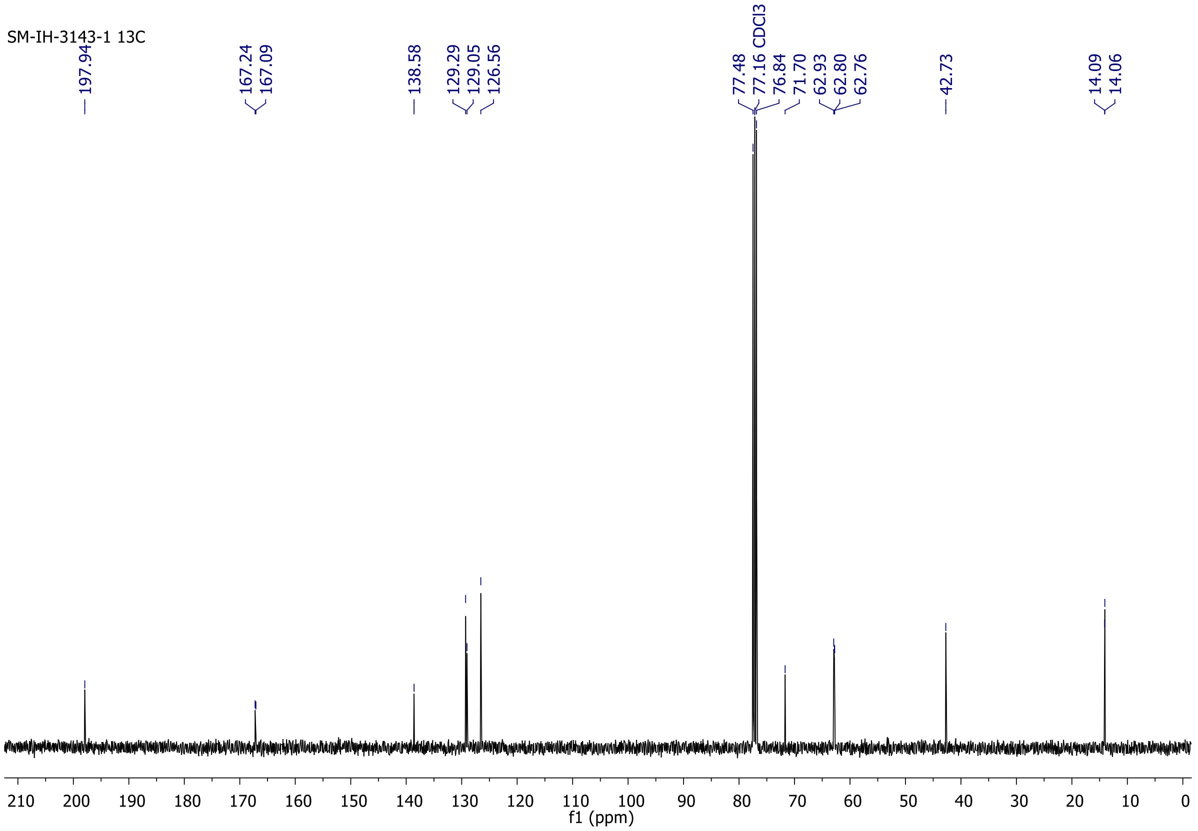

$^1$H NMR of **4c** (400 MHz, $CDCl_3$):

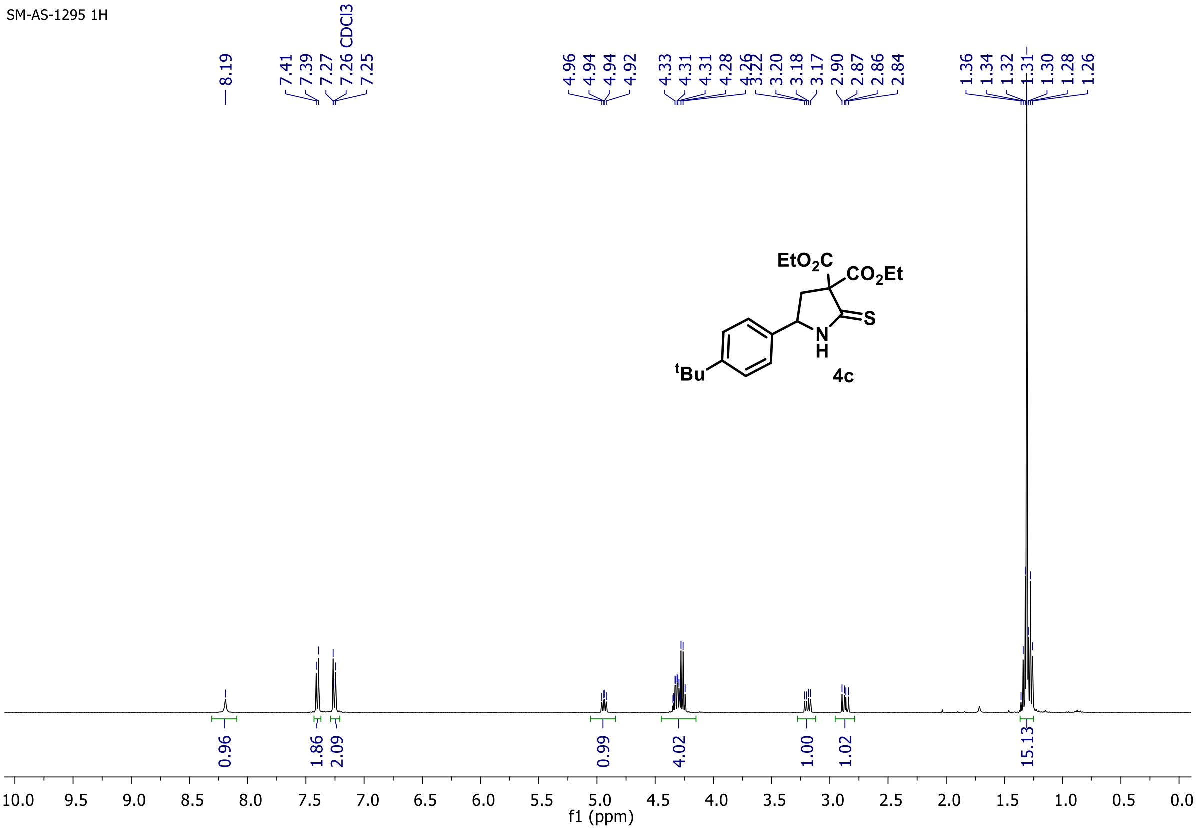


$^{13}C\{^1H\}$ NMR of **4c** (101 MHz, $CDCl_3$):

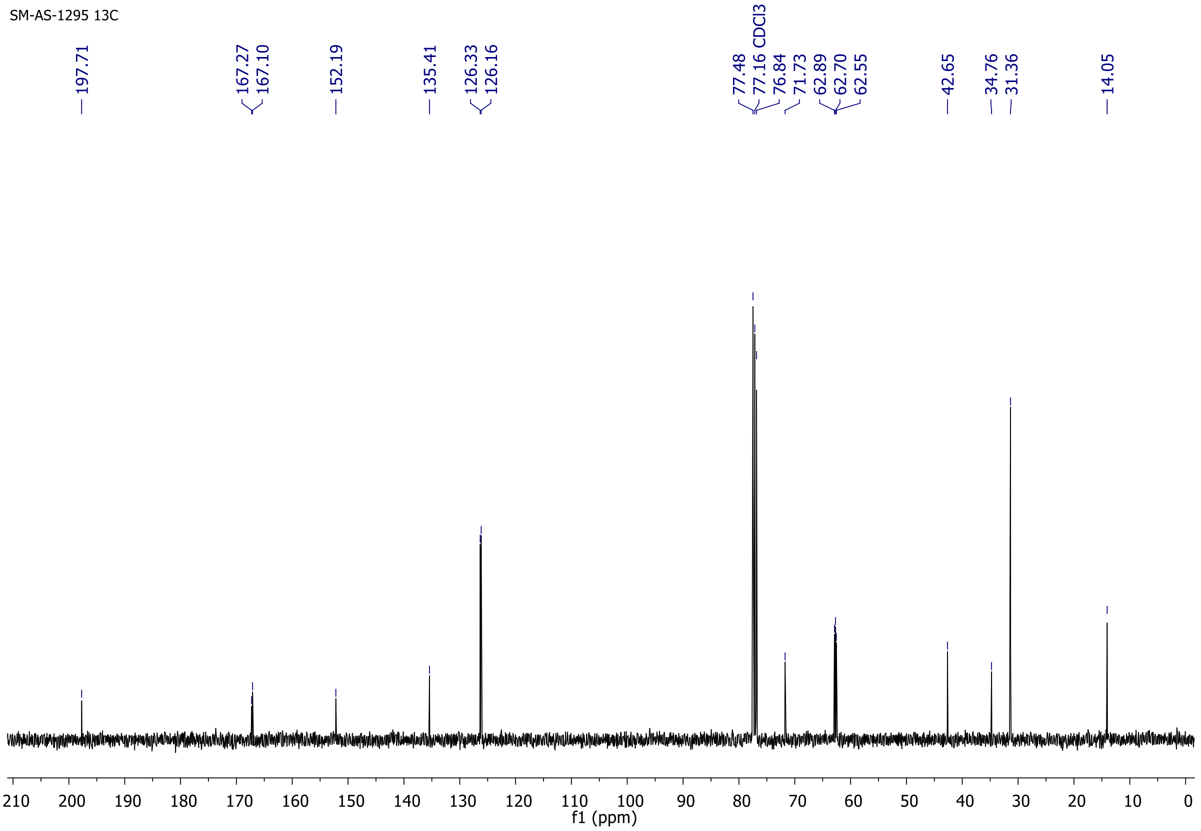

$^{1}$H NMR of **4d** (400 MHz, $CDCl_3$):

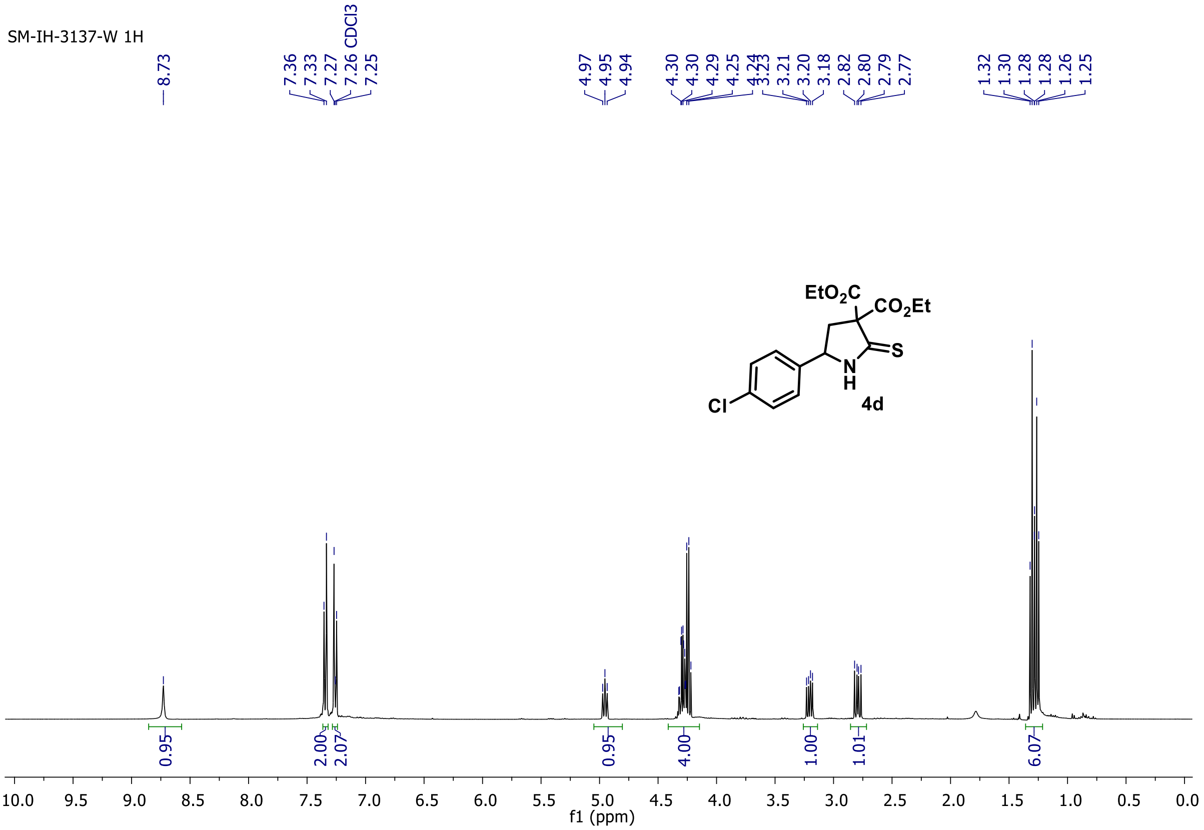


$^{13}$C{$^{1}$H} NMR of **4d** (101 MHz, $CDCl_3$):

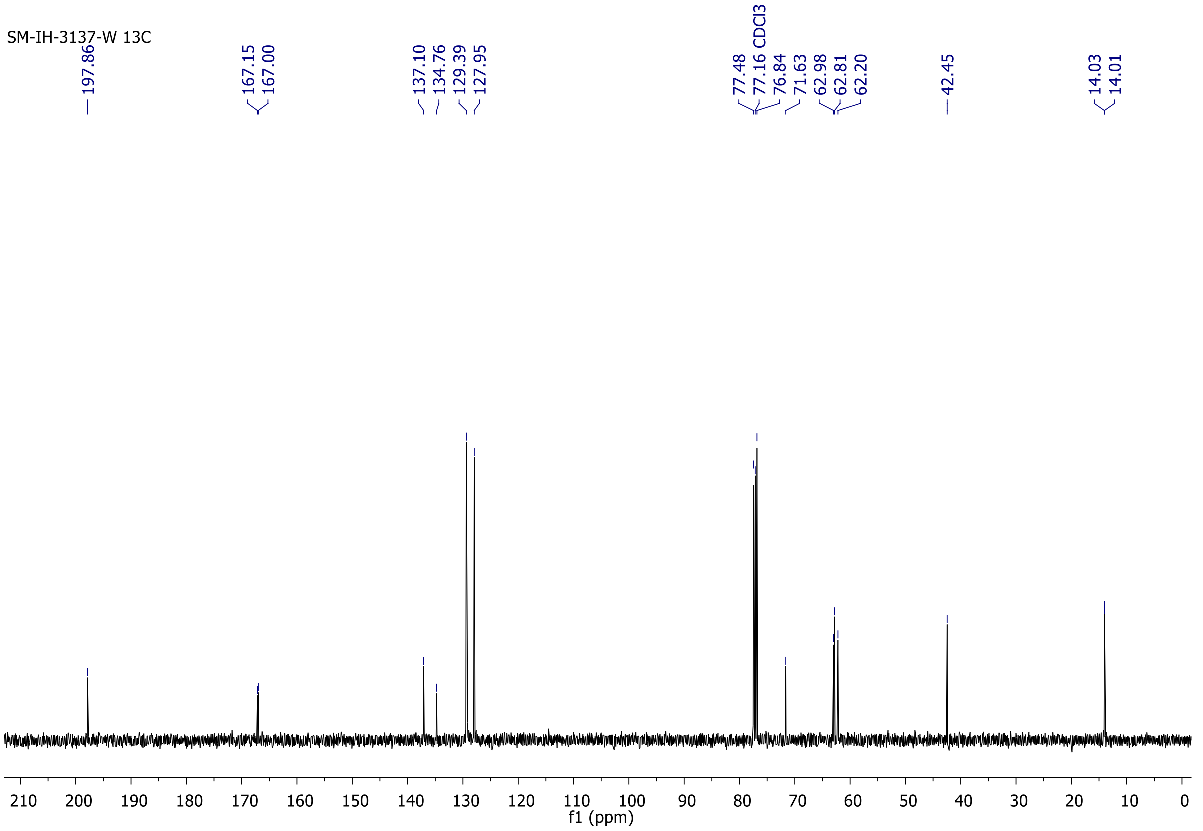

$^{1}H$ NMR of **4e** (400 MHz, $CDCl_3$):

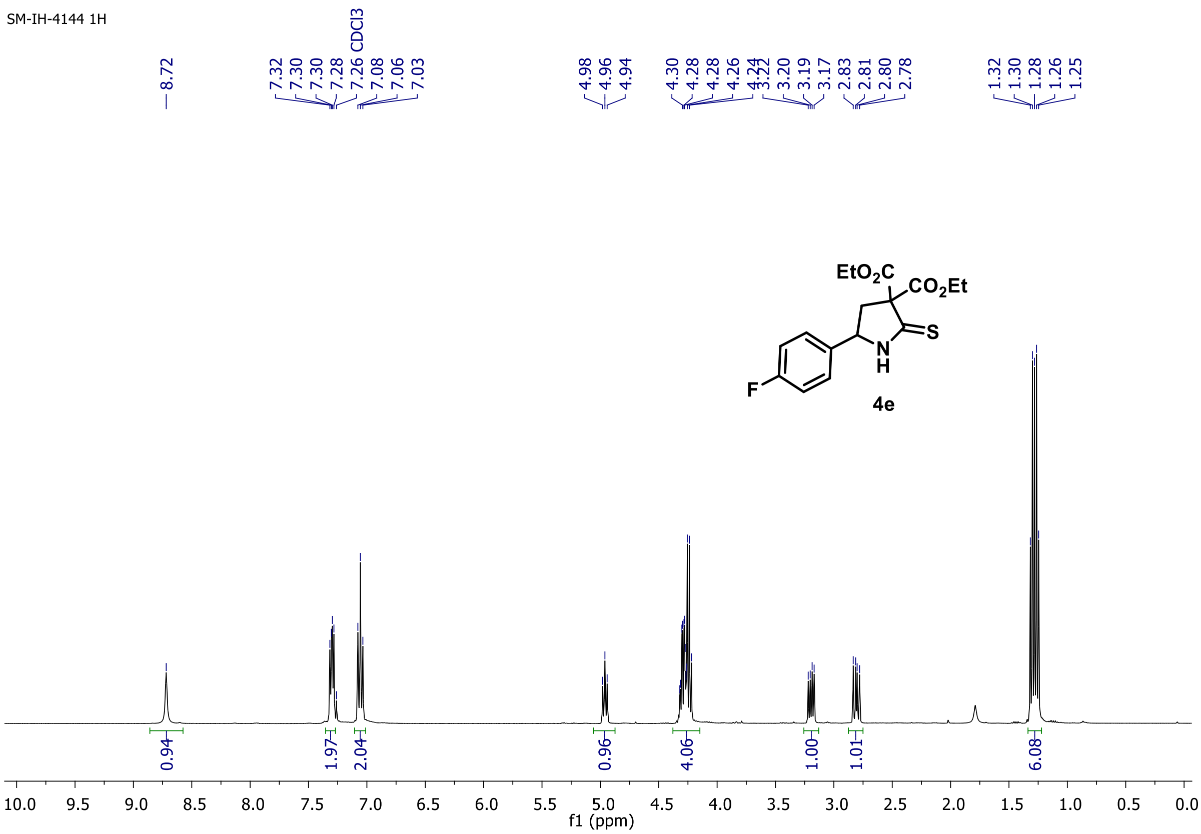


$^{13}C\{^{1}H\}$ NMR of **4e** (101 MHz, $CDCl_3$):

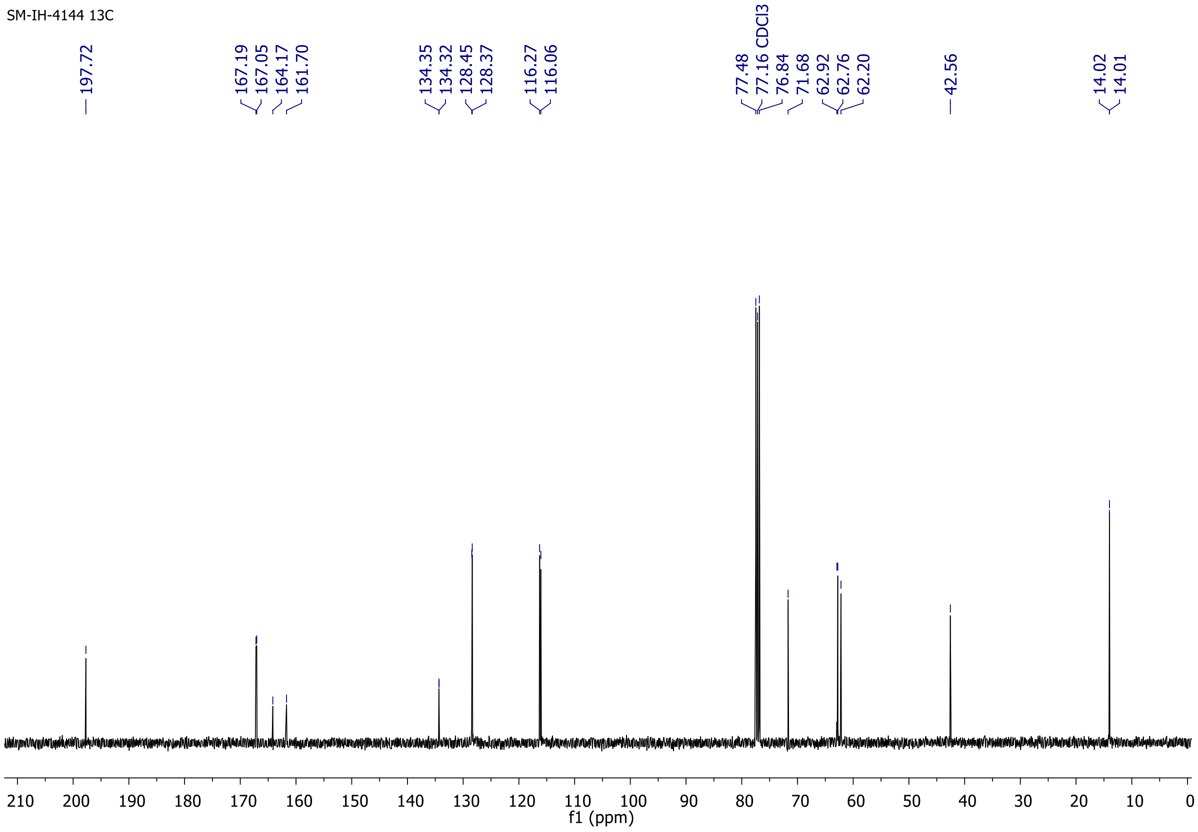

$^{19}F$ NMR of **4e** (377 MHz, $CDCl_3$):

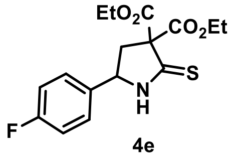

**4e**

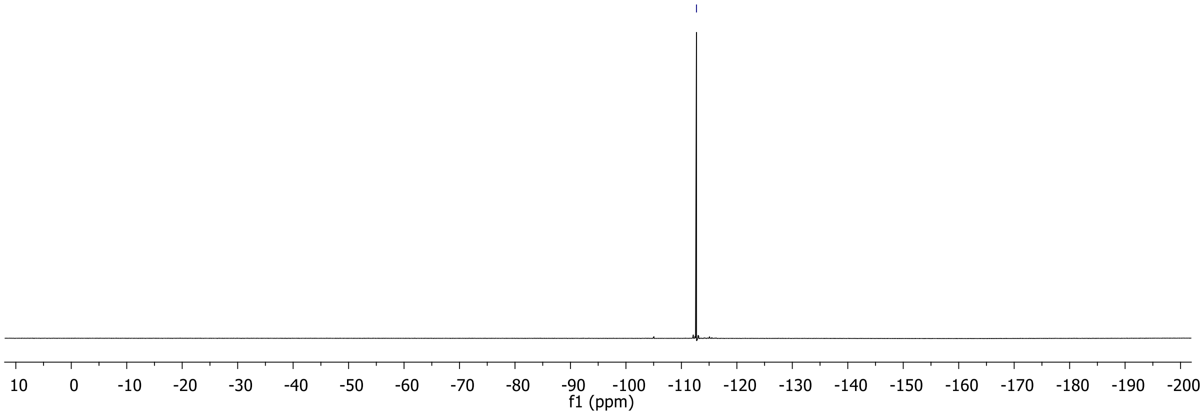

$^{1}H$ NMR of **4f** (400 MHz, $CDCl_3$):

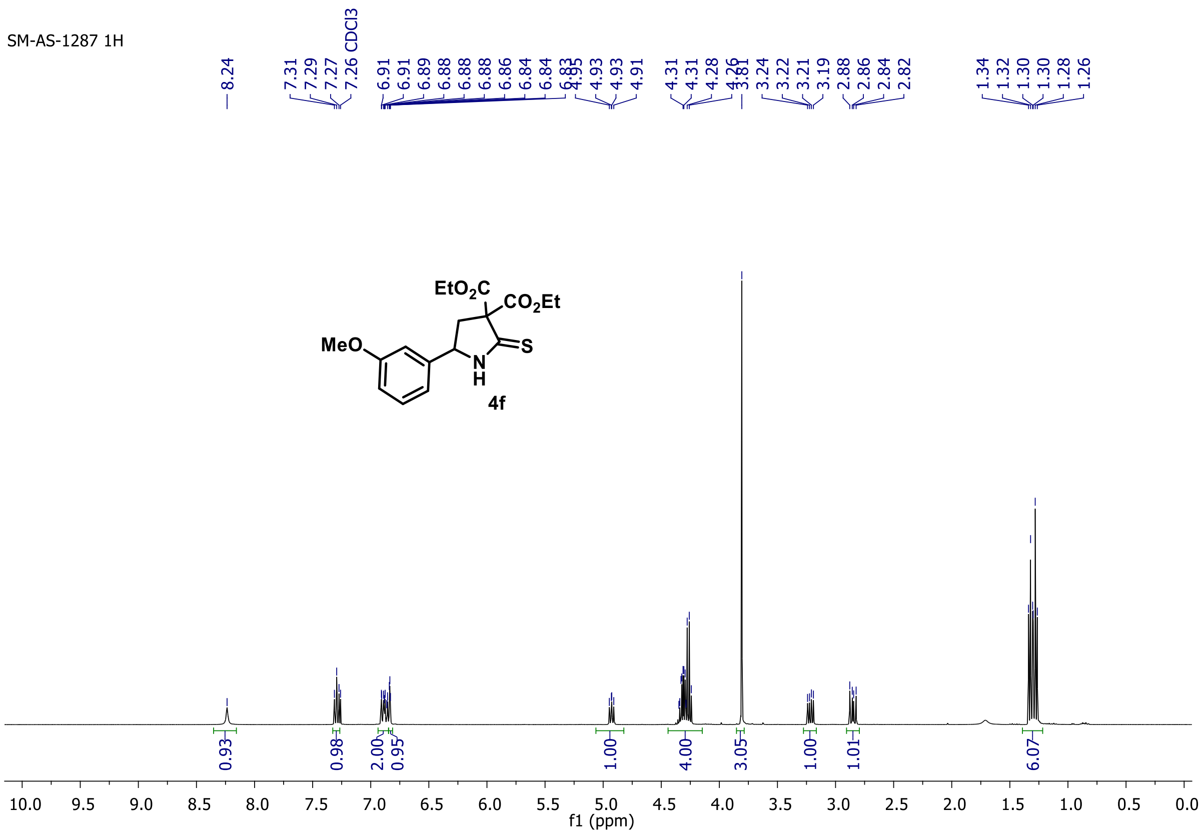


$^{13}C\{^{1}H\}$ NMR of **4f** (101 MHz, $CDCl_3$):

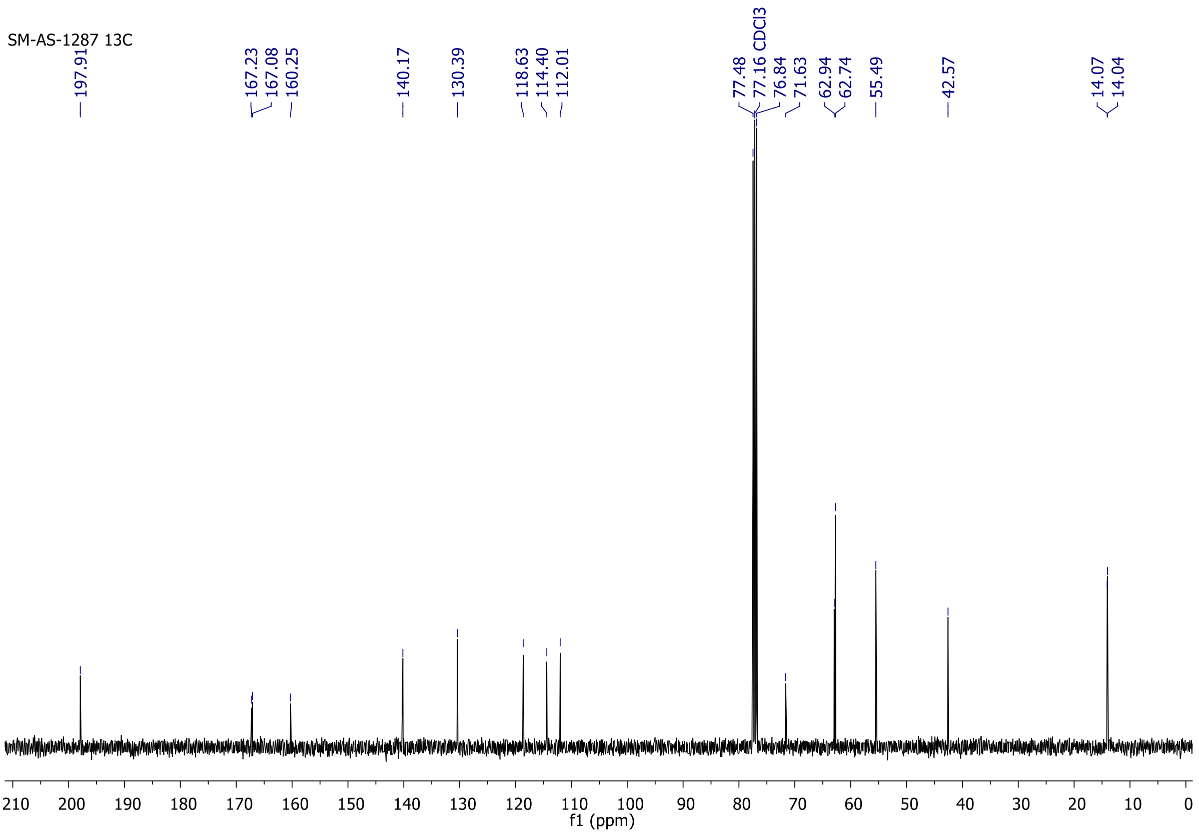

$^{1}H$ NMR of **4g** (400 MHz, $CDCl_3$):

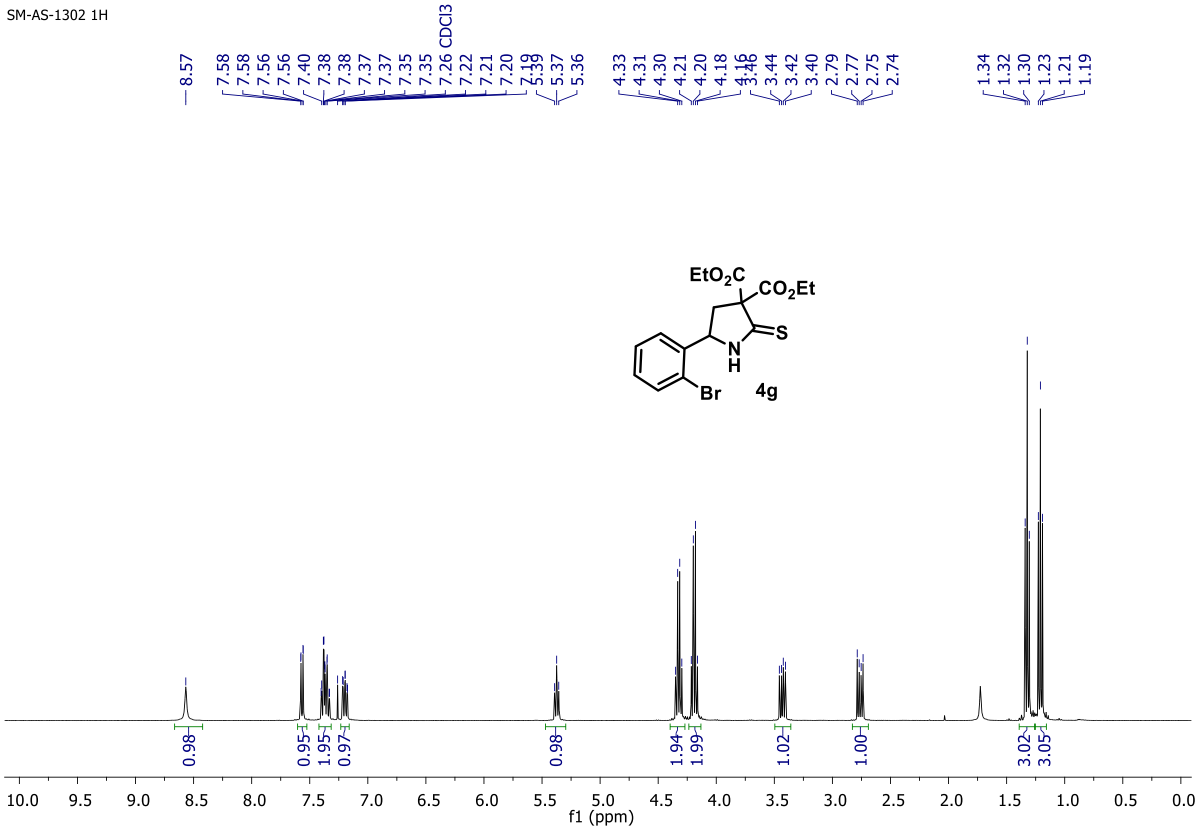


$^{13}C\{^{1}H\}$ NMR of **4g** (101 MHz, $CDCl_3$):

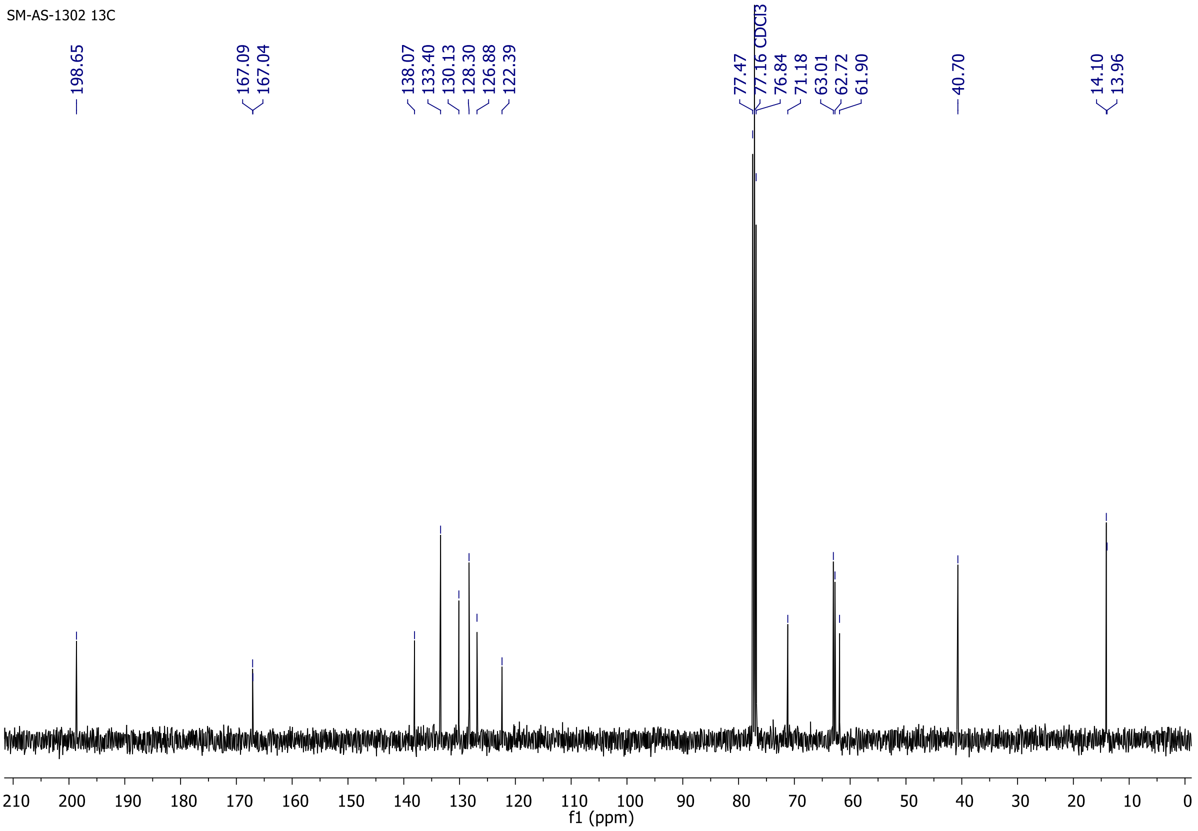

$^{1}H$ NMR of **4h** (400 MHz, $CDCl_3$):

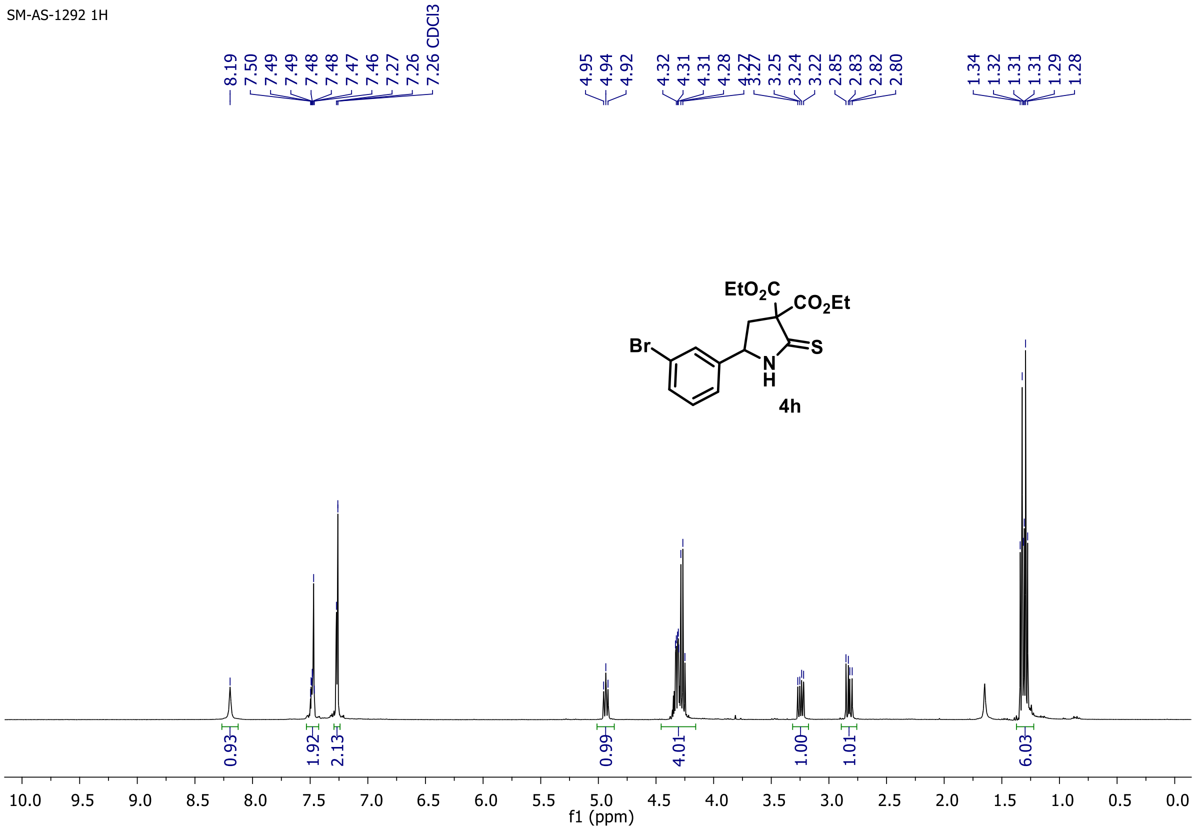


$^{13}C\{^{1}H\}$ NMR of **4h** (101 MHz, $CDCl_3$):

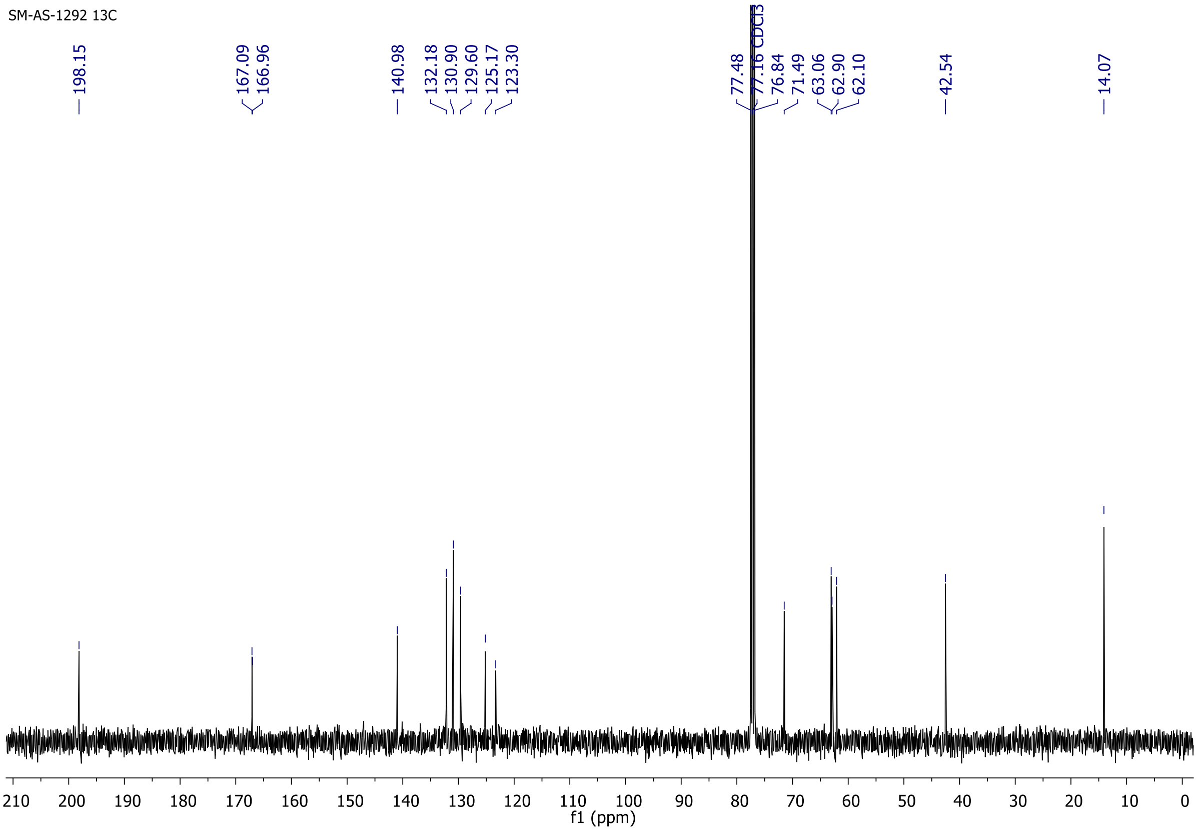

$^{1}H$ NMR of **4i** (400 MHz, $CDCl_3$):

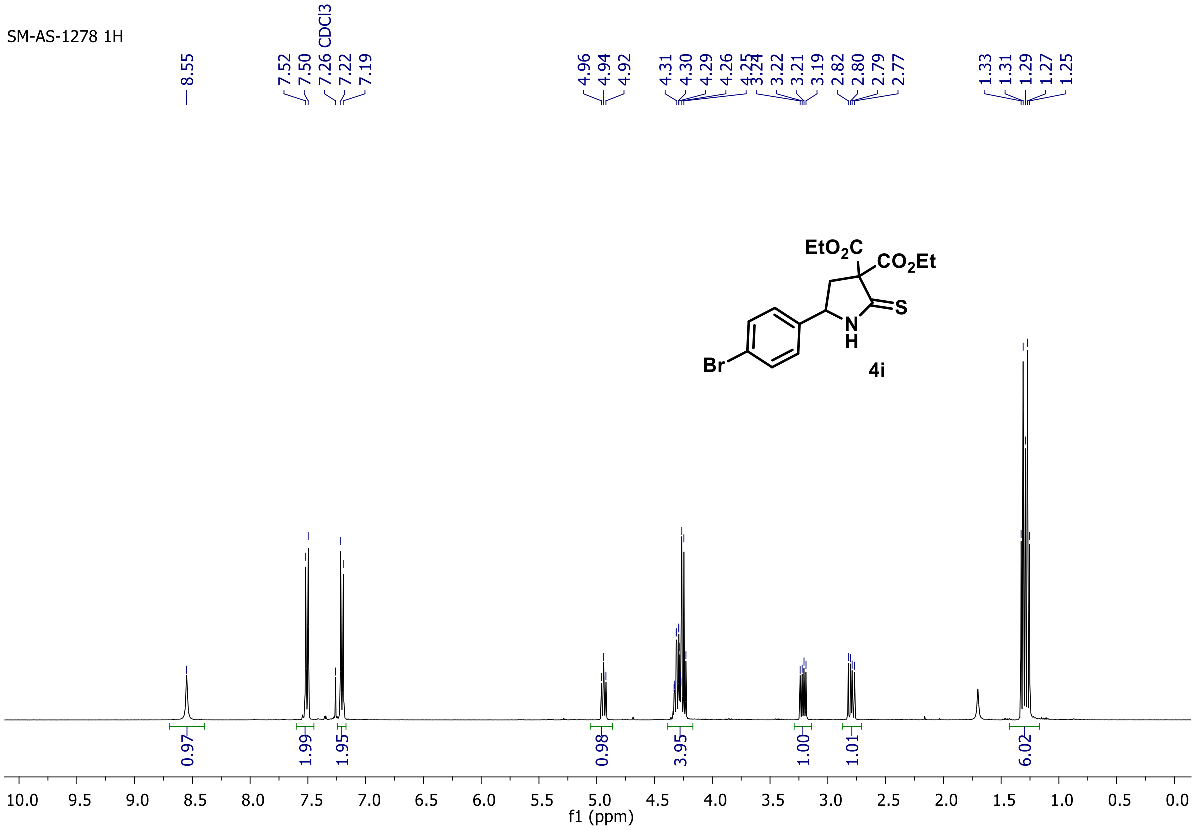


$^{13}C\{^{1}H\}$ NMR of **4i** (101 MHz, $CDCl_3$):

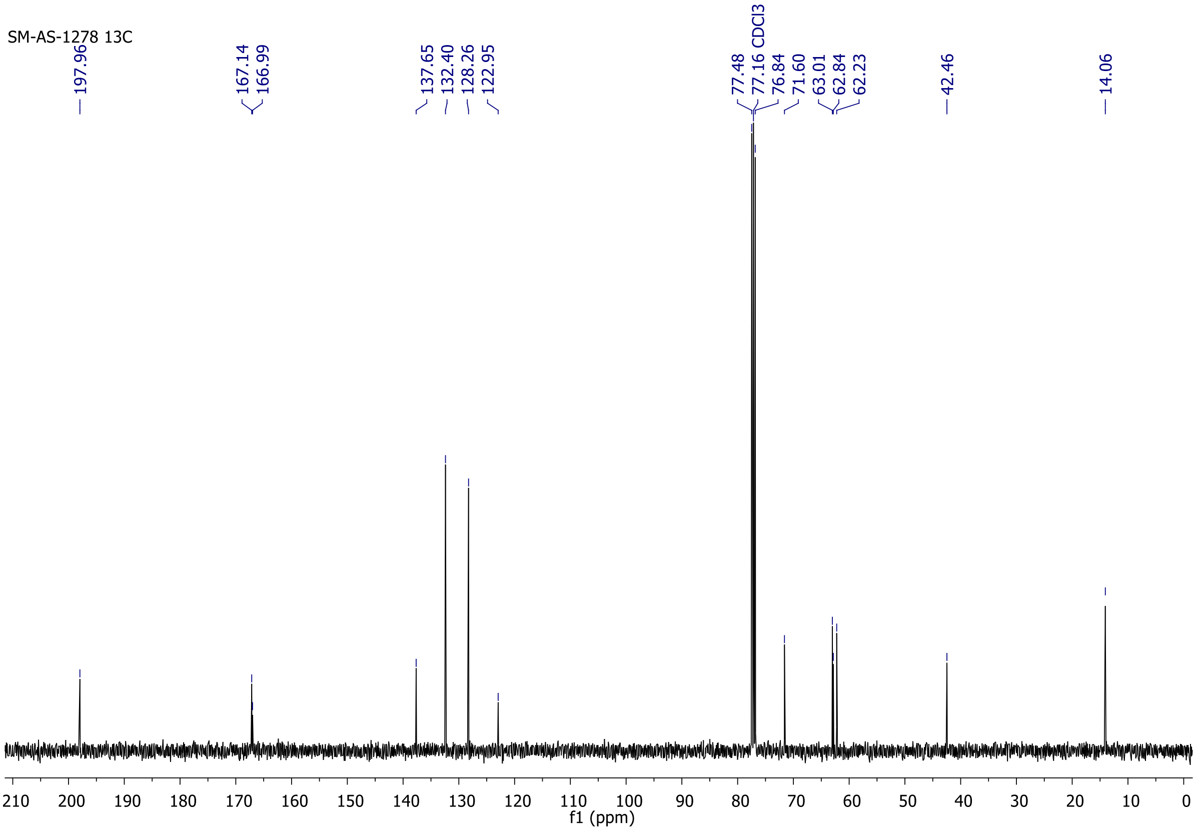

$^1H$ NMR of **4j** (400 MHz, $CDCl_3$):

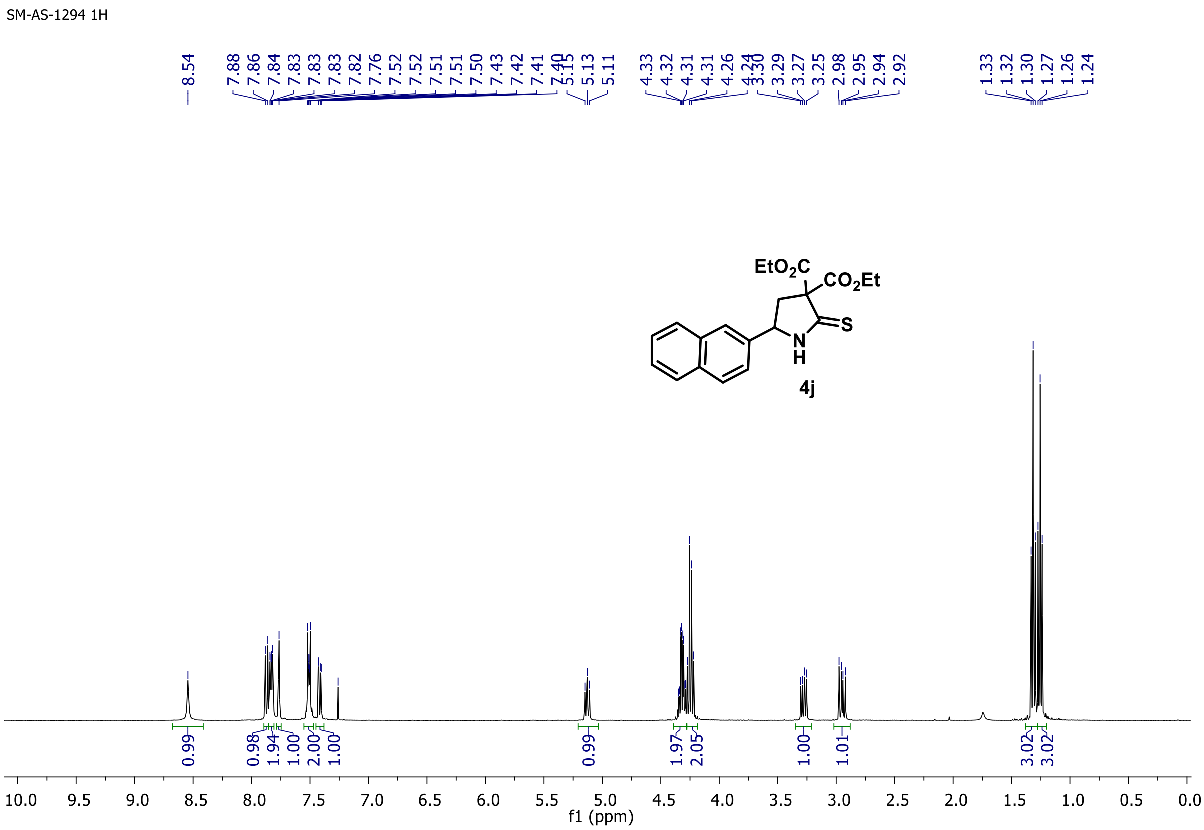


$^{13}C\{^1H\}$ NMR of **4j** (101 MHz, $CDCl_3$):

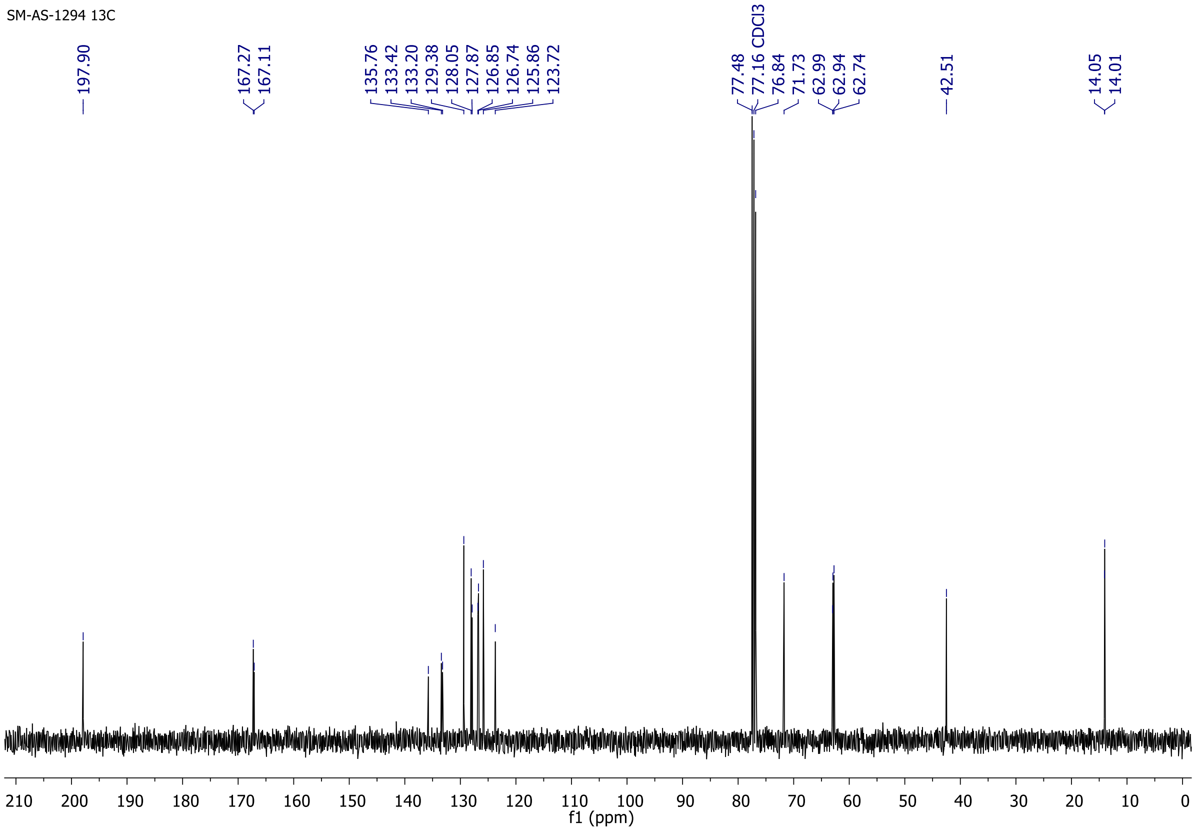

<sup></sup>

$^1$H NMR of **4k** (400 MHz, $CDCl_3$):

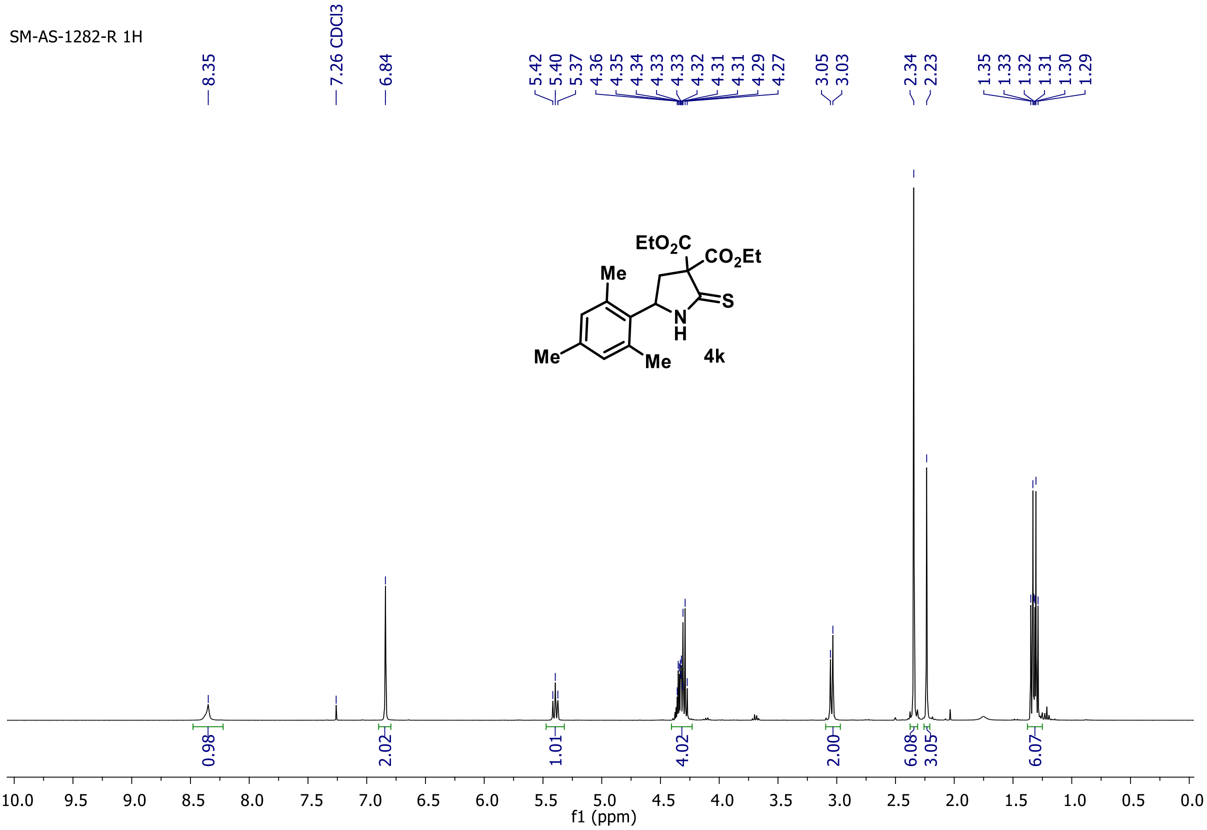


$^{13}C\{^1H\}$ NMR of **4k** (101 MHz, $CDCl_3$):

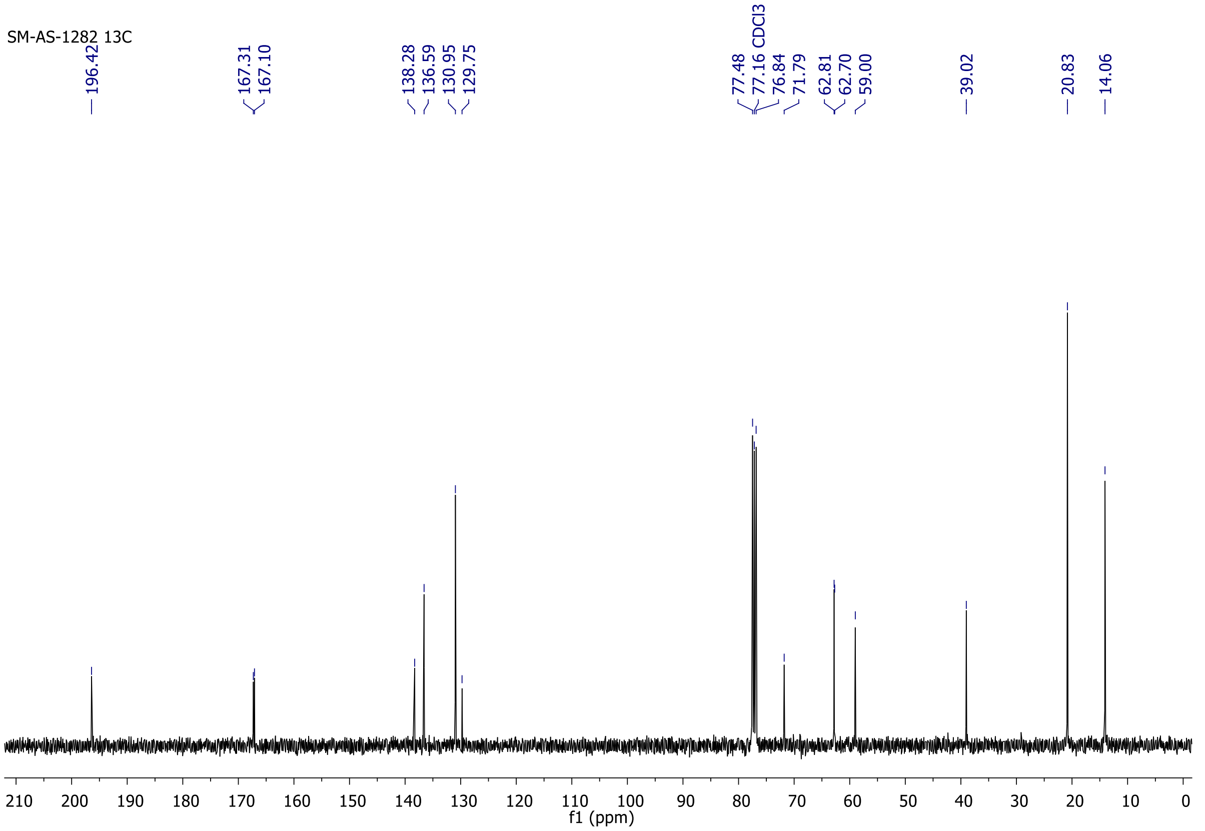

$^{1}H$ NMR of **4l** (400 MHz, $CDCl_3$):

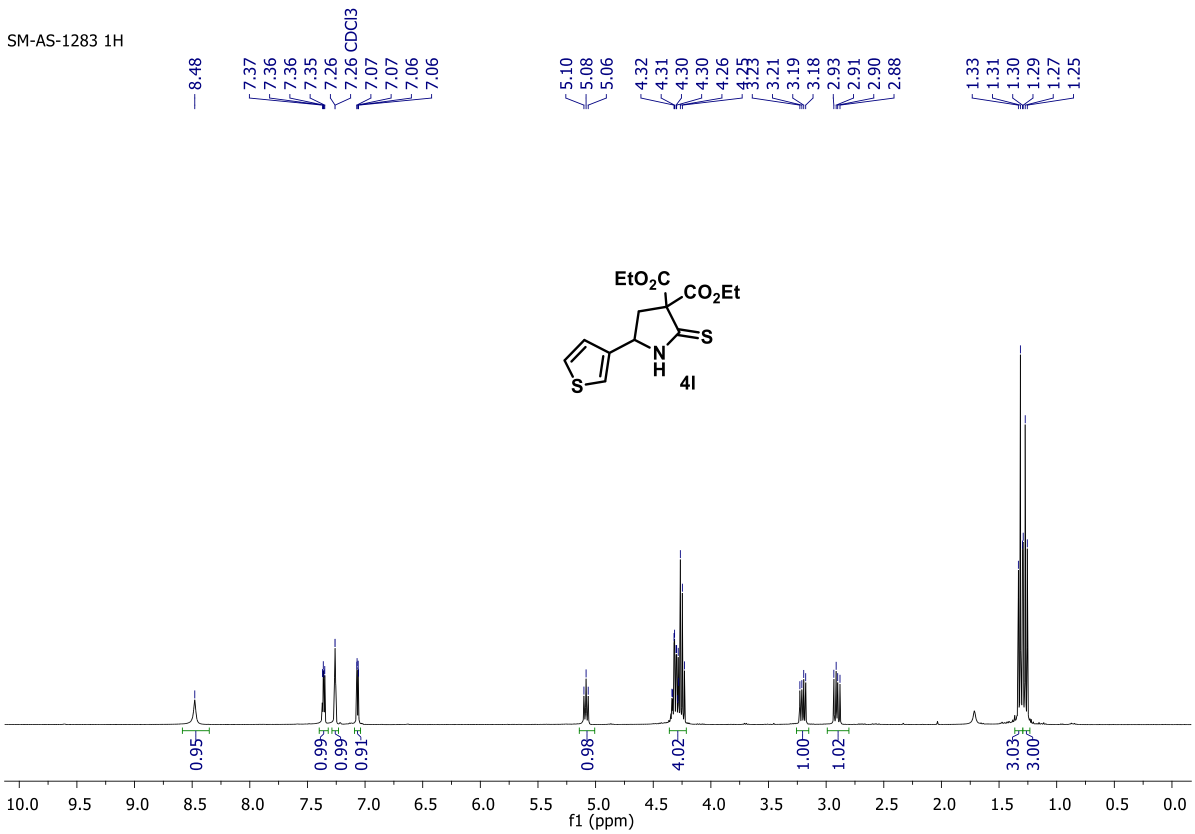


$^{13}C\{^{1}H\}$ NMR of **4l** (101 MHz, $CDCl_3$):

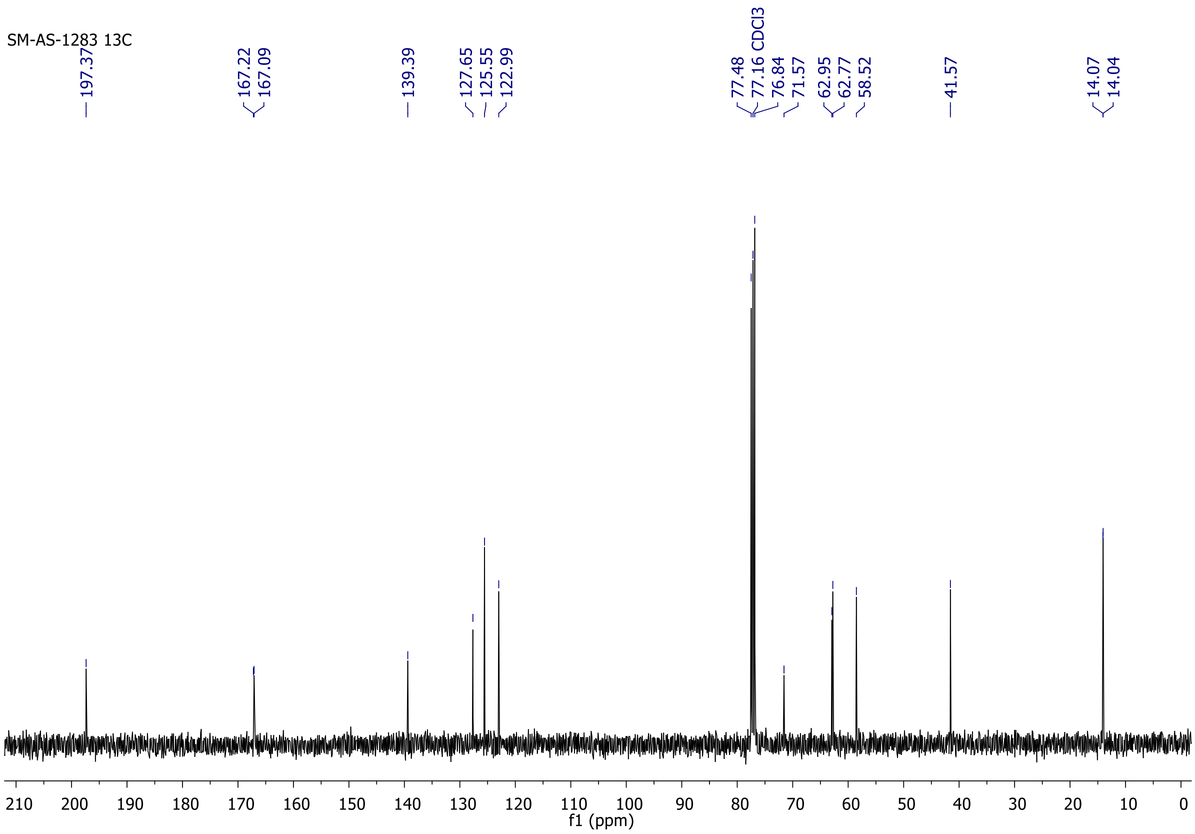

$^{1}$H NMR of **4m** (400 MHz, $CDCl_3$):

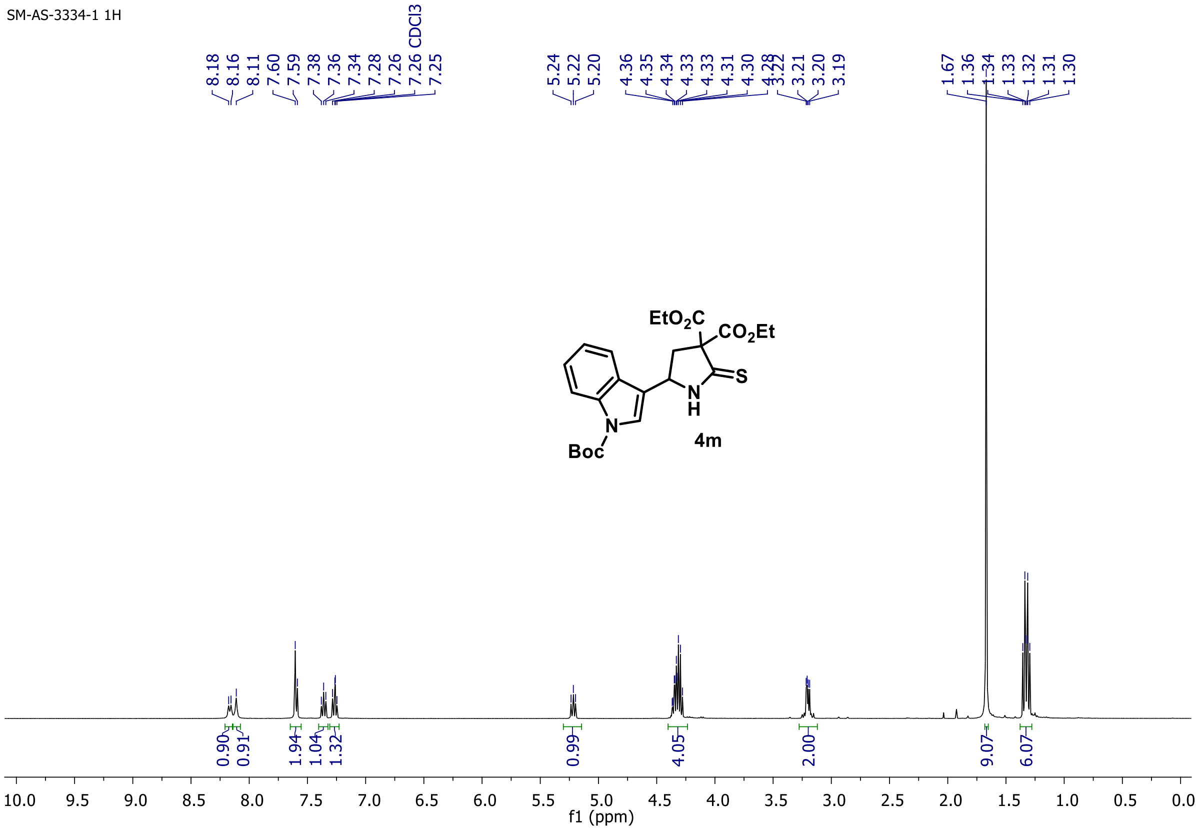


$^{13}$C{$^{1}$H} NMR of **4m** (101 MHz, $CDCl_3$):

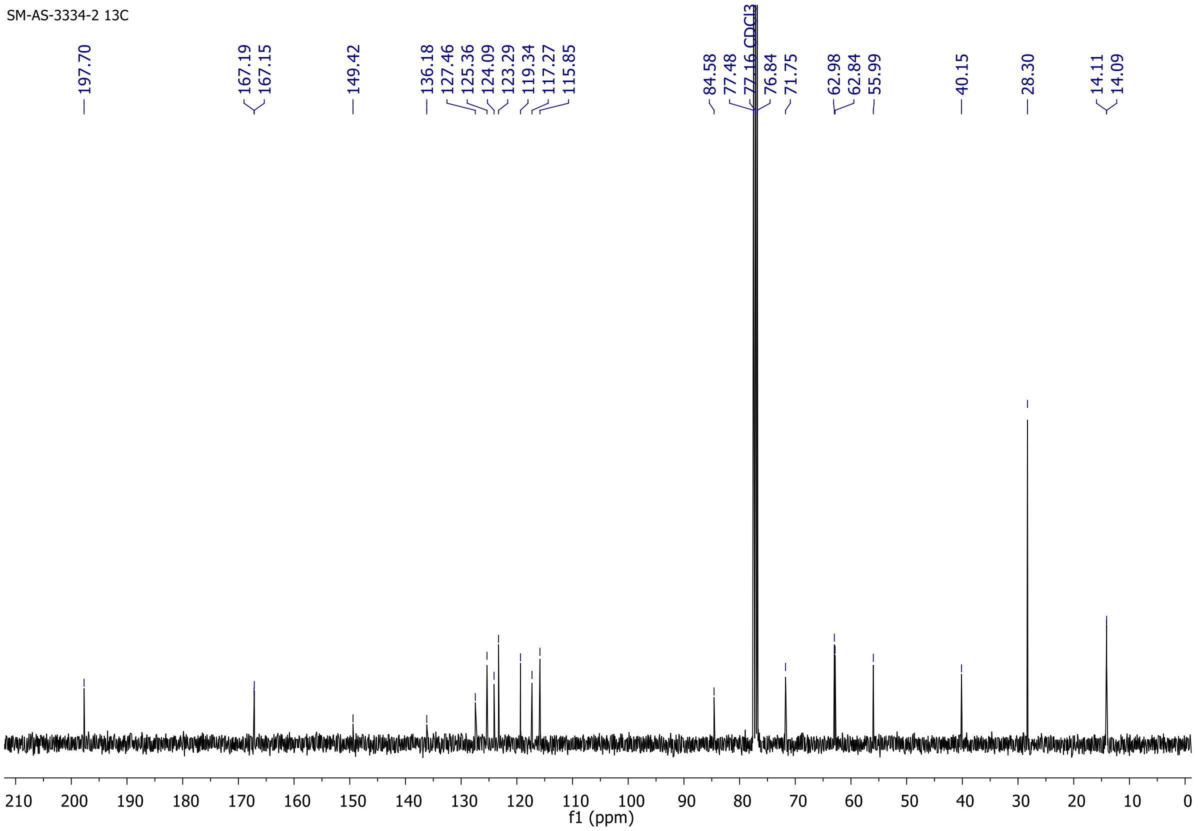

$^{1}H$ NMR of **4n** (400 MHz, $CDCl_3$):

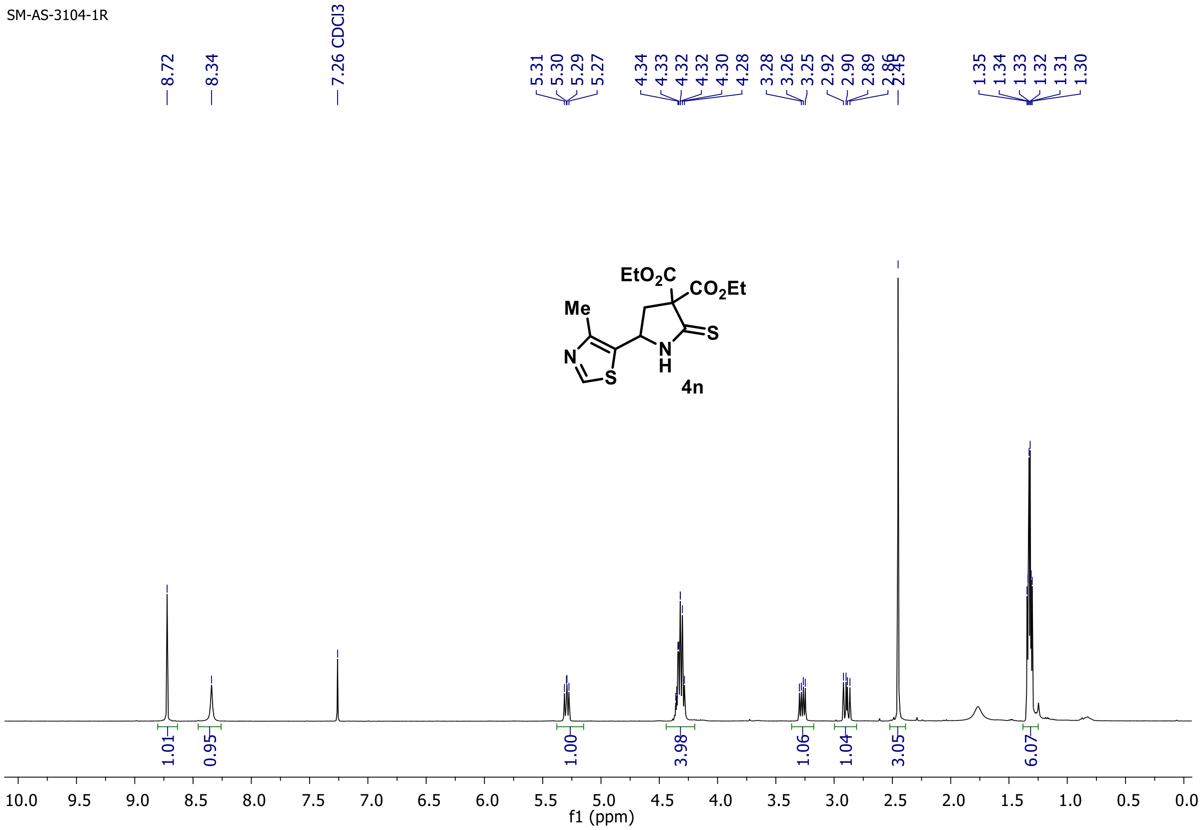


$^{13}C\{^{1}H\}$ NMR of **4n** (101 MHz, $CDCl_3$):

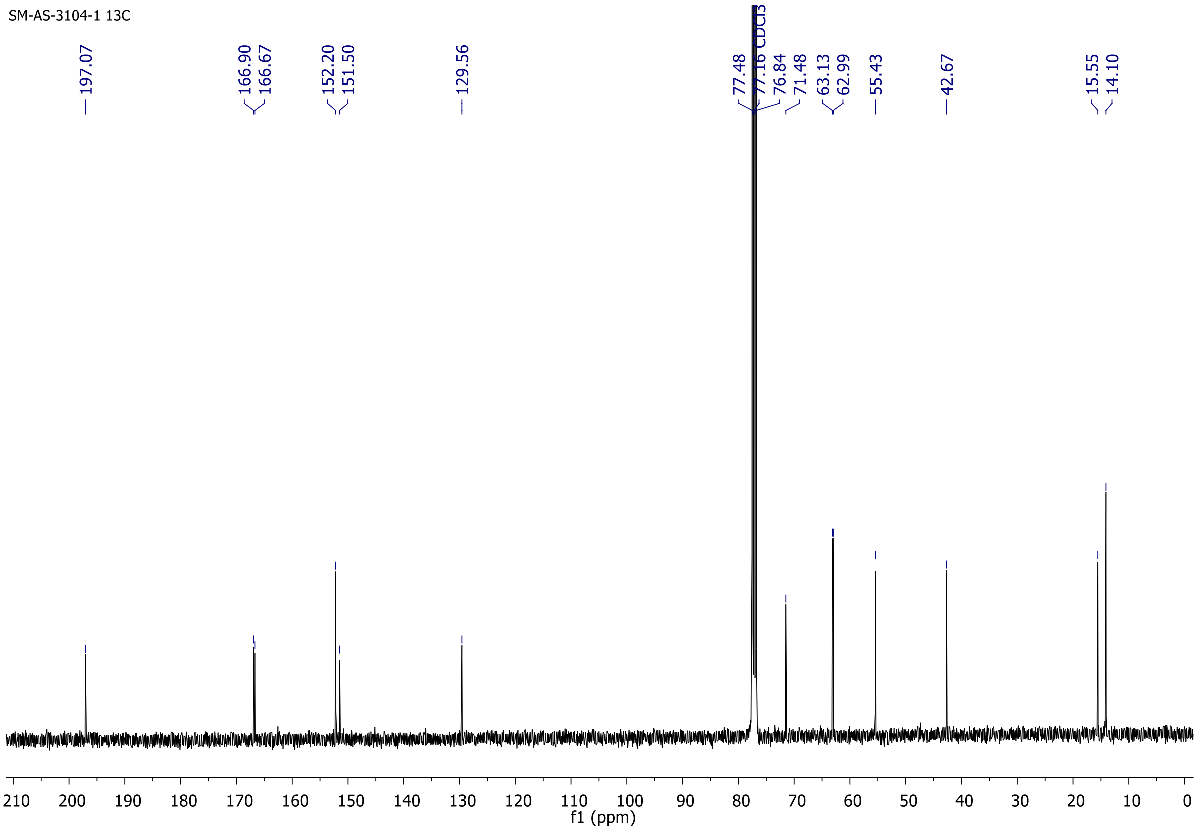

$^{1}H$ NMR of **4o** (400 MHz, $CDCl_3$):

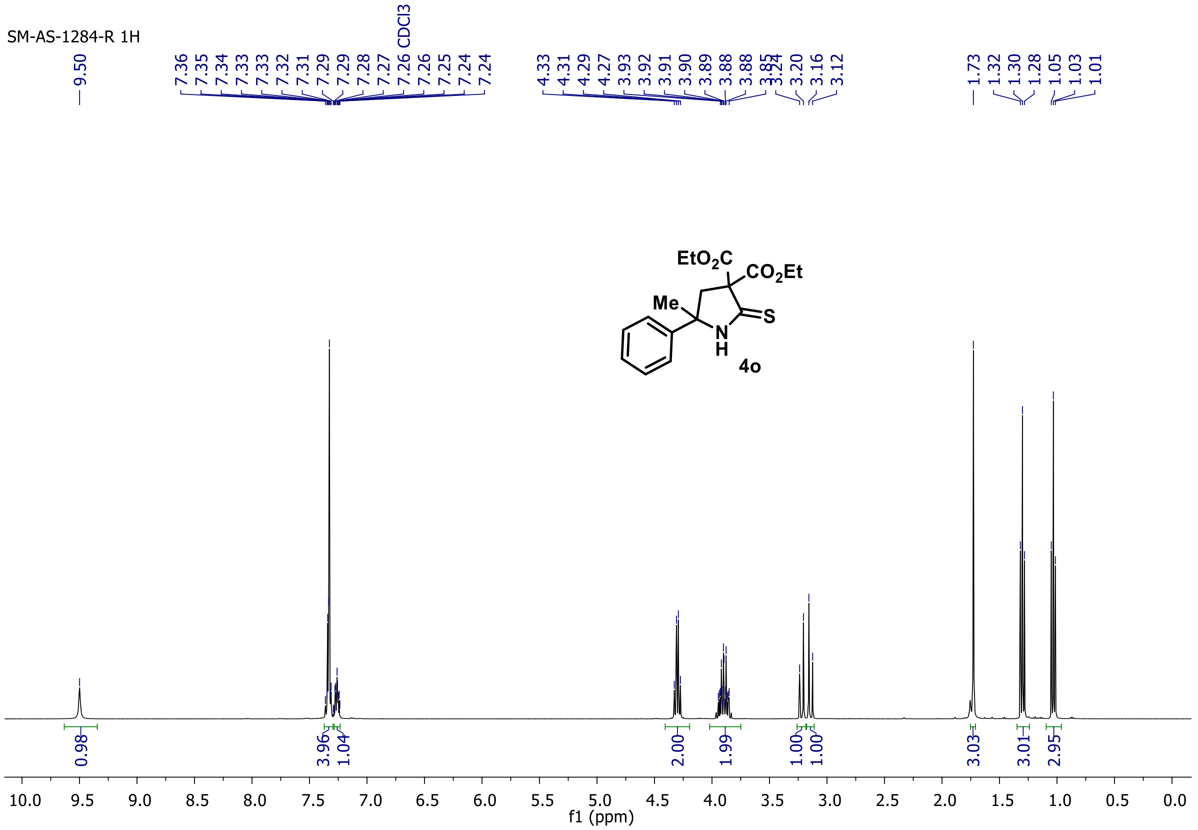


$^{13}C\{^{1}H\}$ NMR of **4o** (101 MHz, $CDCl_3$):

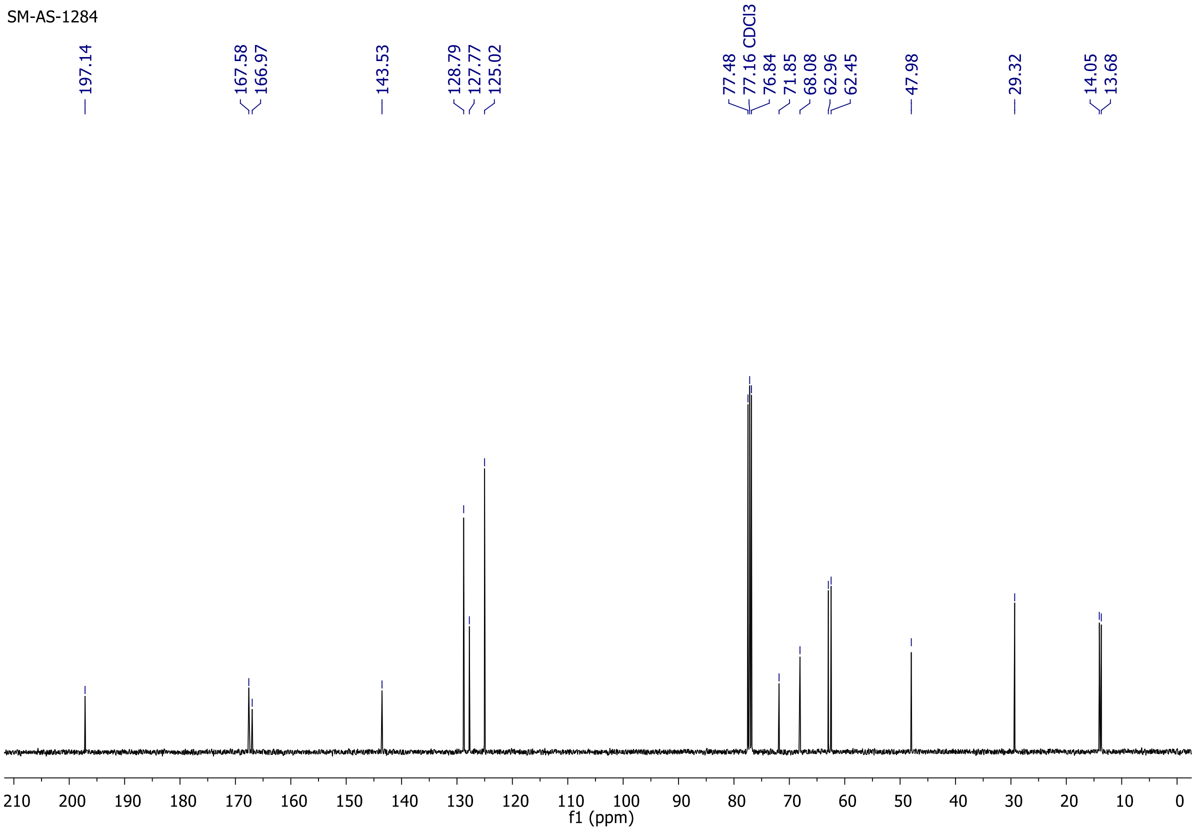

$^{1}H$ NMR of **4p** (400 MHz, $CDCl_3$):

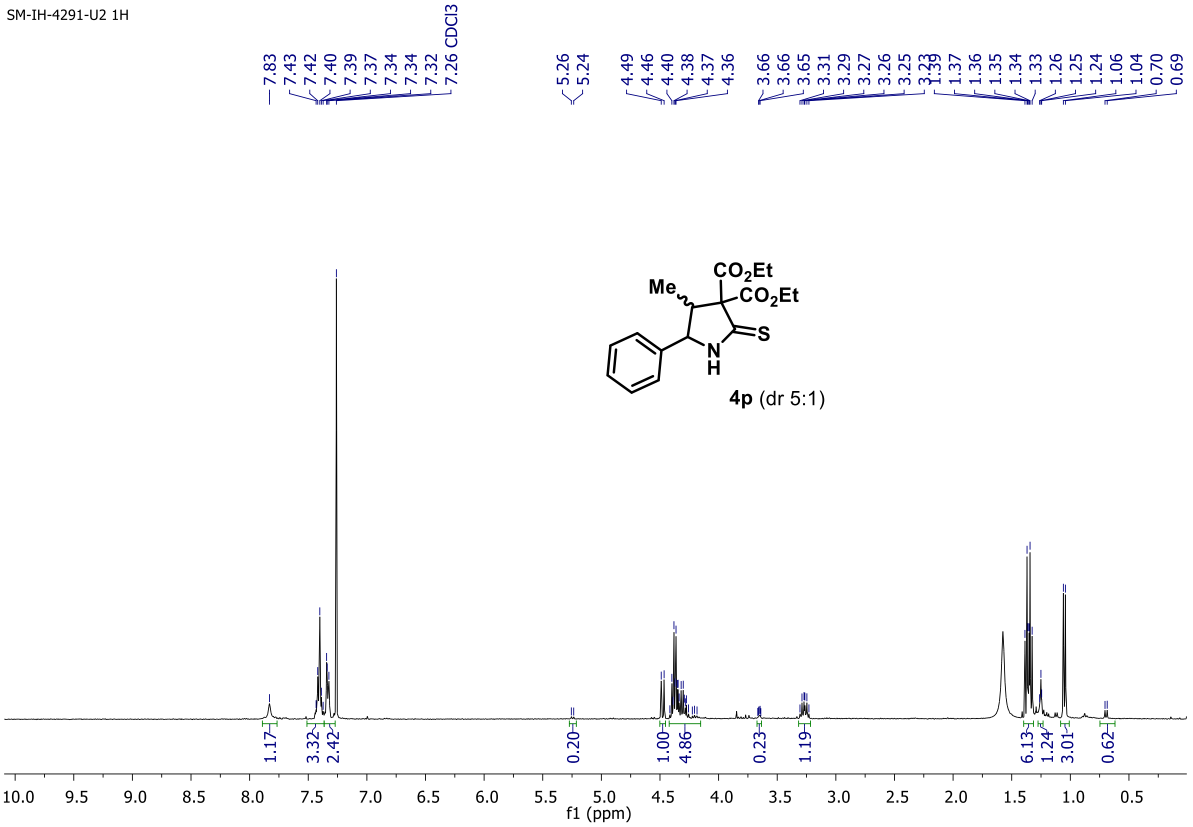


$^{13}C\{^{1}H\}$ NMR of **4p** (101 MHz, $CDCl_3$):

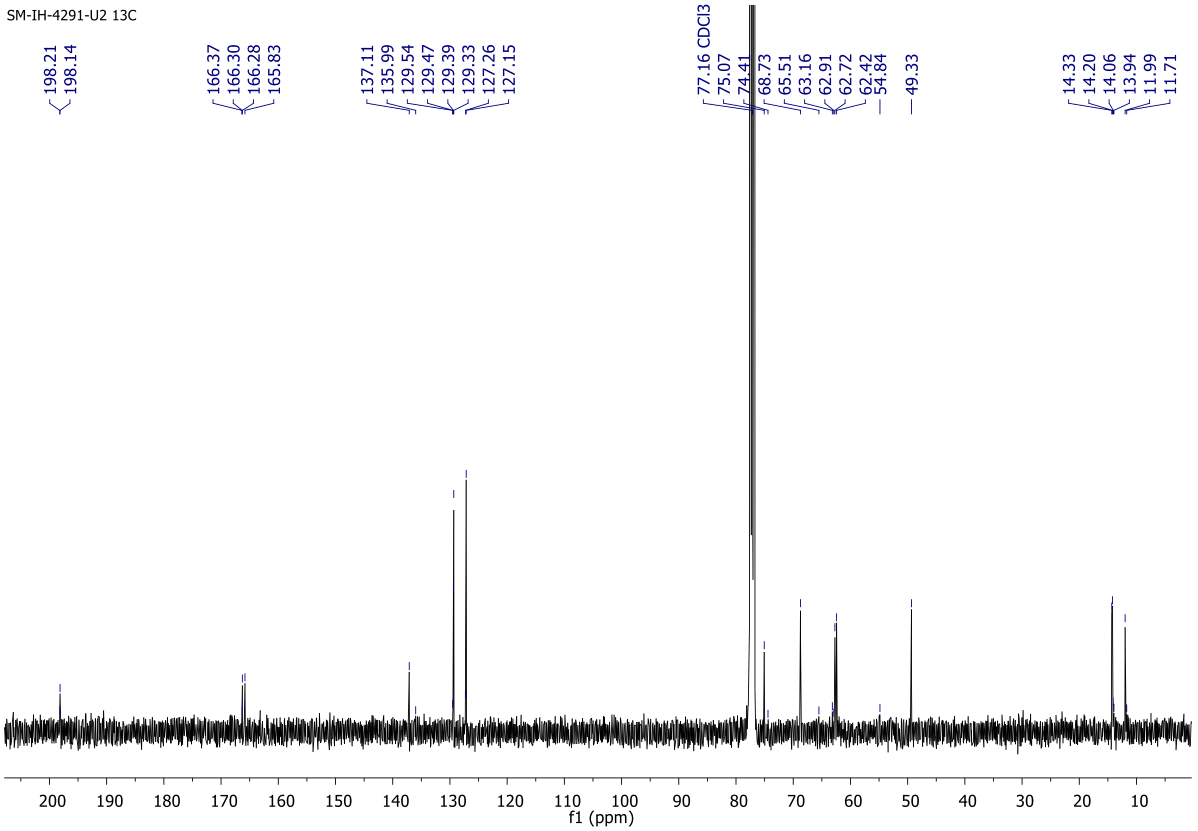

$^{1}$H NMR of **4q** (400 MHz, $CDCl_3$):

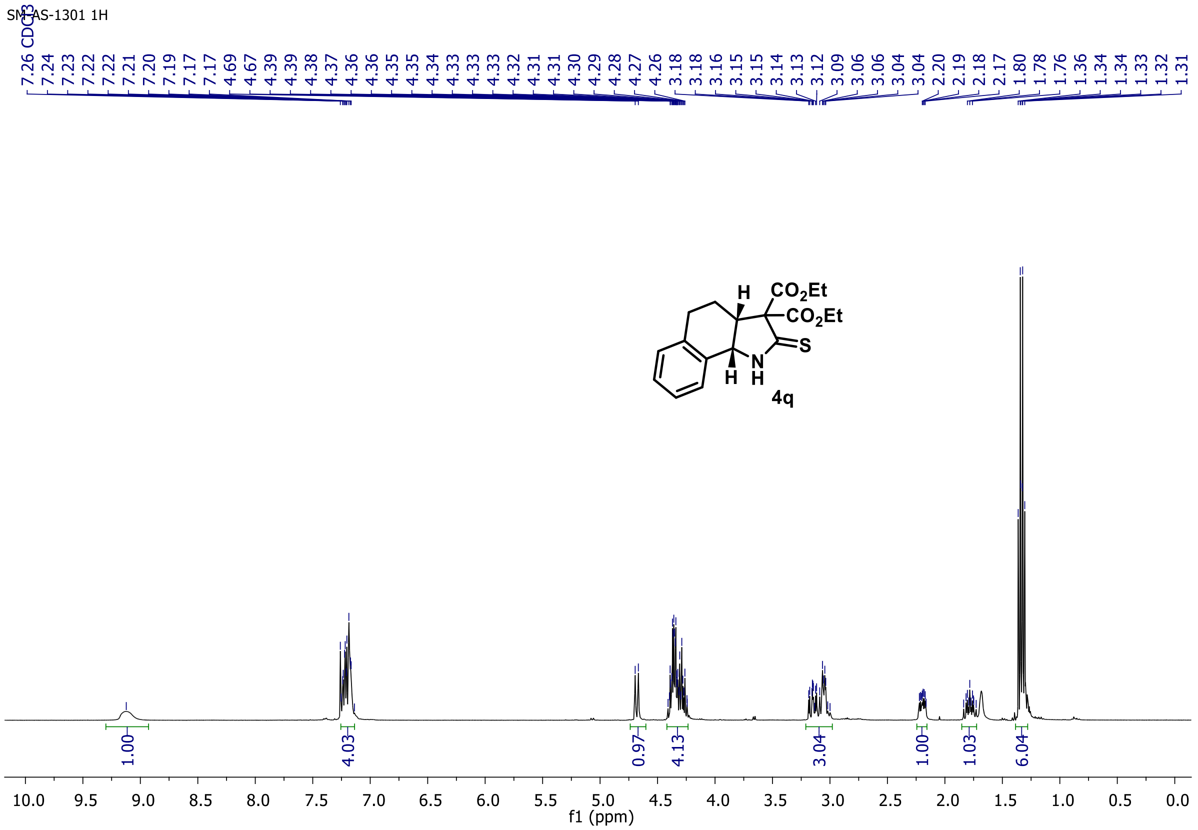


$^{13}$C{$^{1}$H} NMR of **4q** (101 MHz, $CDCl_3$):

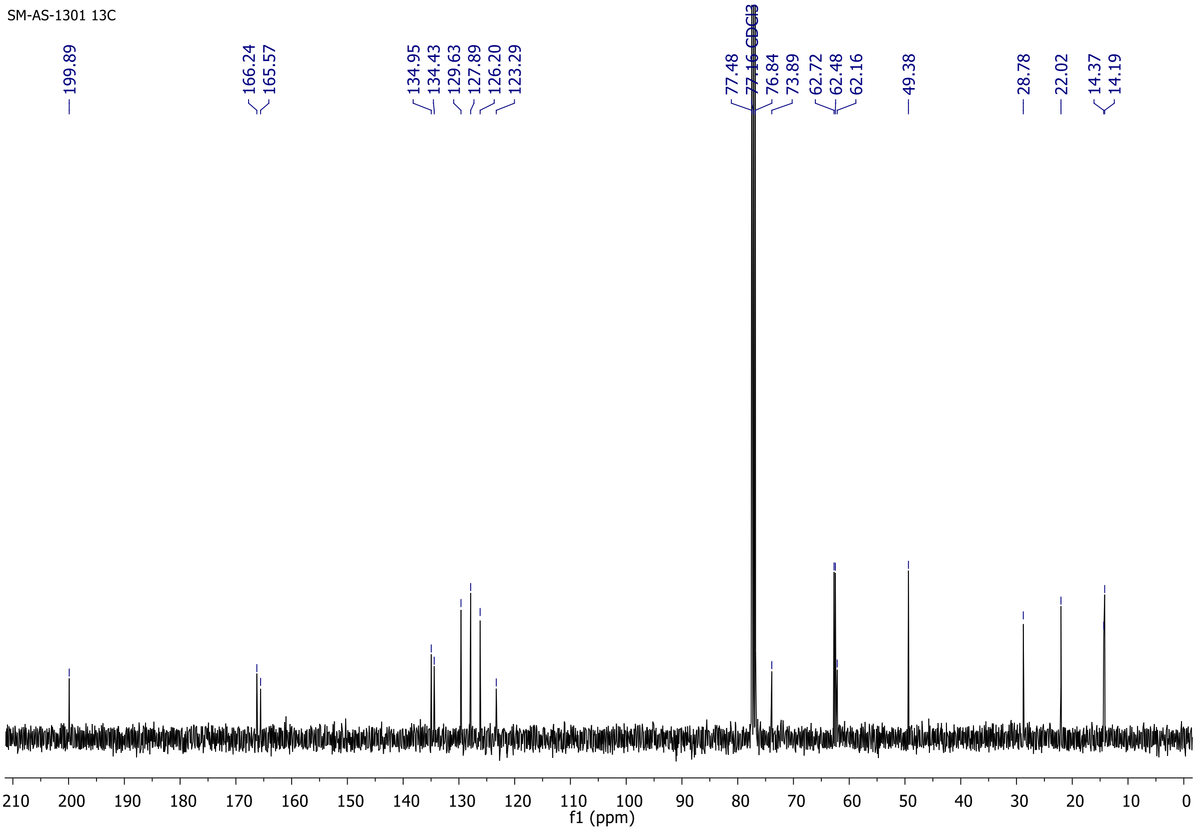

$^{1}$H-$^{1}$H COSY NMR of **4q**:

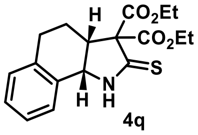


**4q**

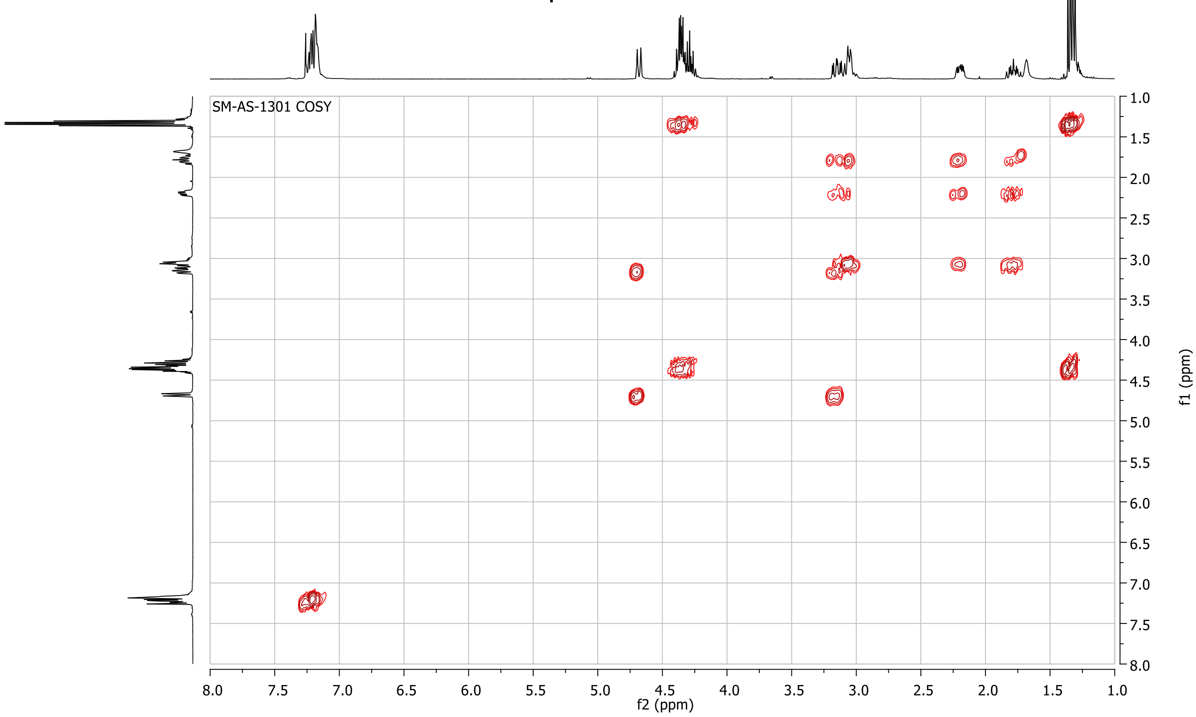


$^{1}$H-$^{13}$C HSQC NMR of **4q**:

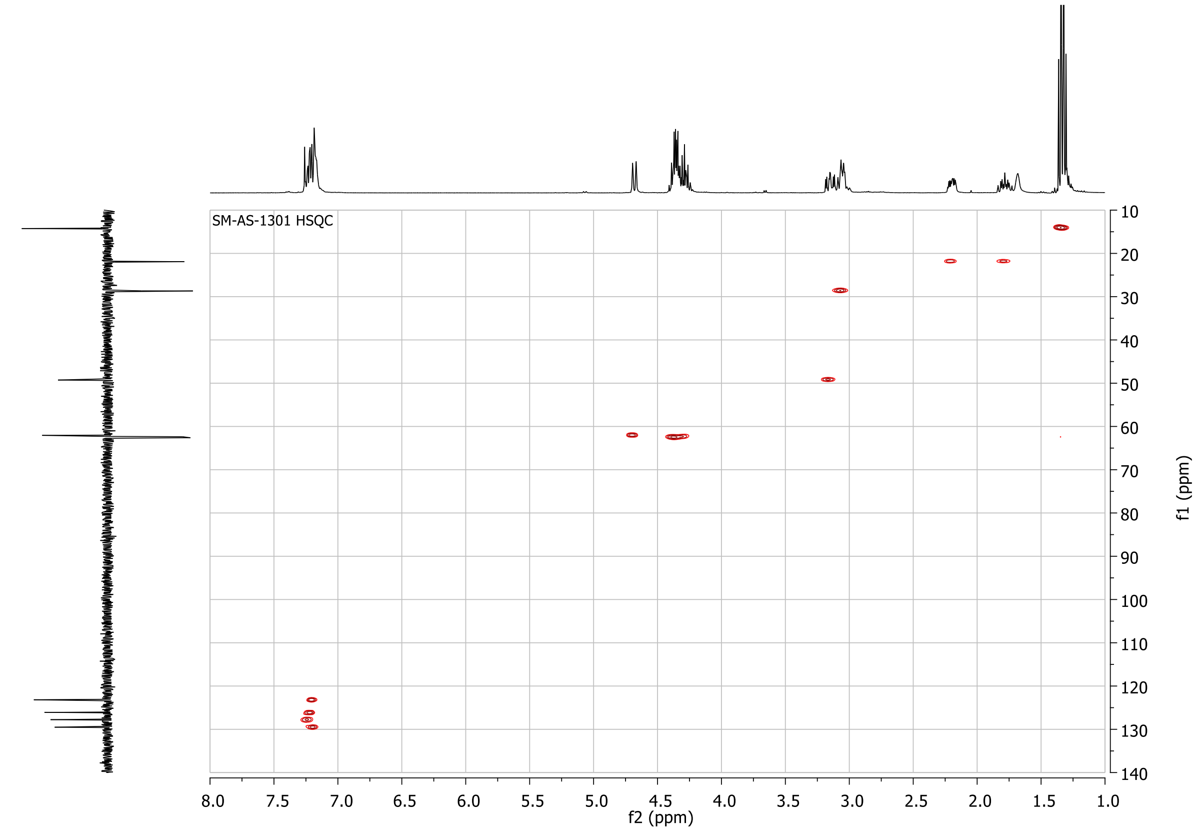

$^1H$-$^1H$ NOESY NMR of **4q**:

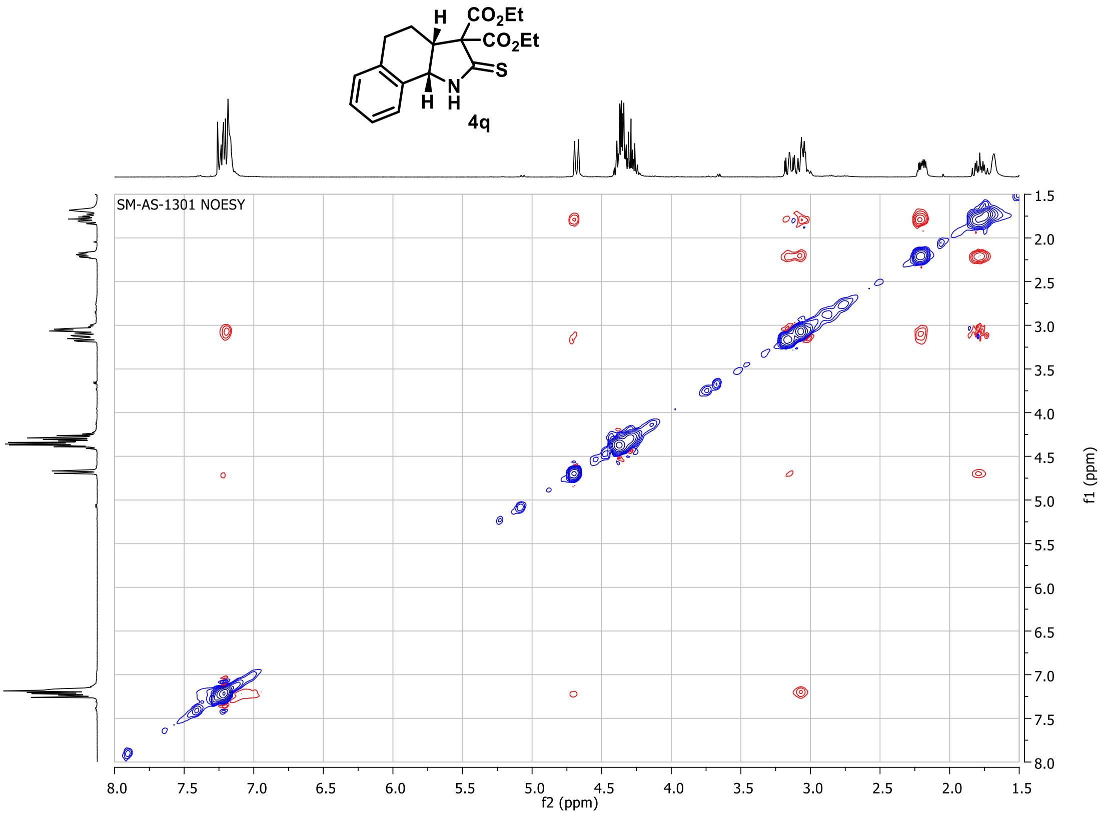

$^1$H NMR of **4r** (400 MHz, $CDCl_3$):

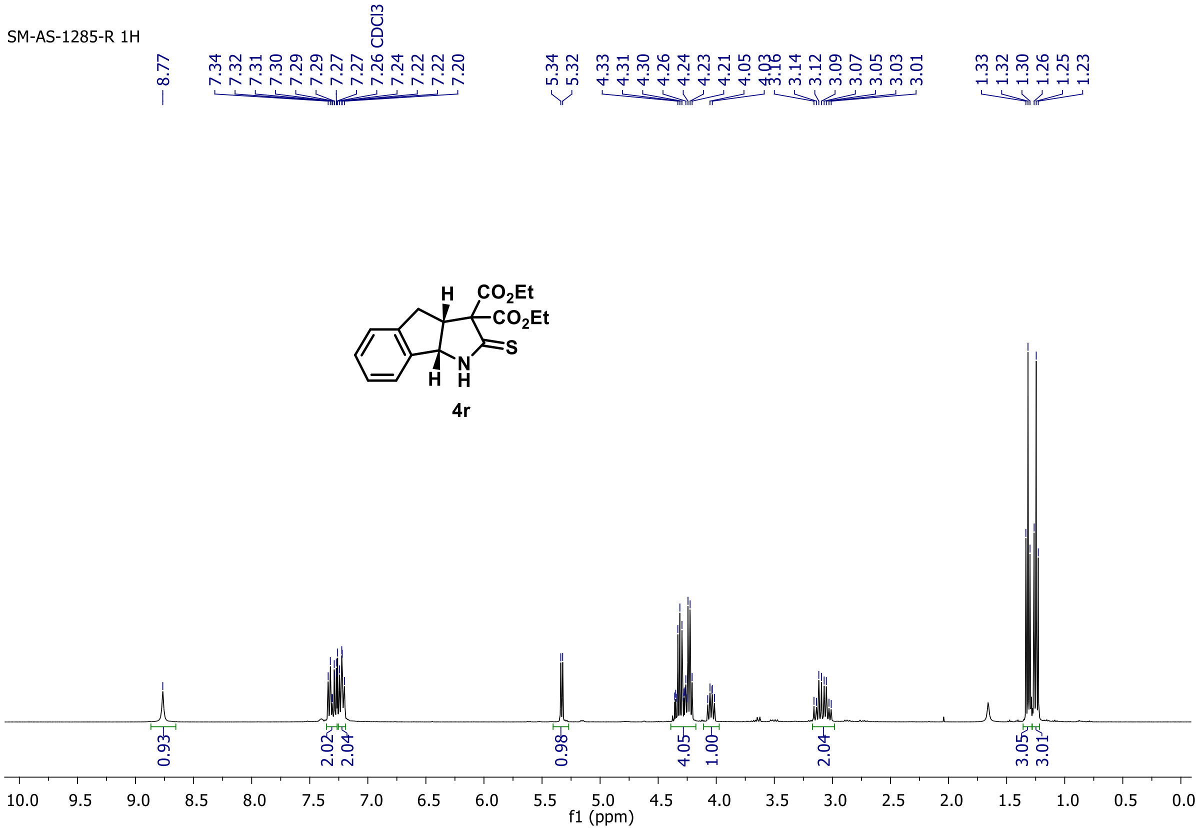


$^{13}$C{$^1$H} NMR of **4r** (101 MHz, $CDCl_3$):

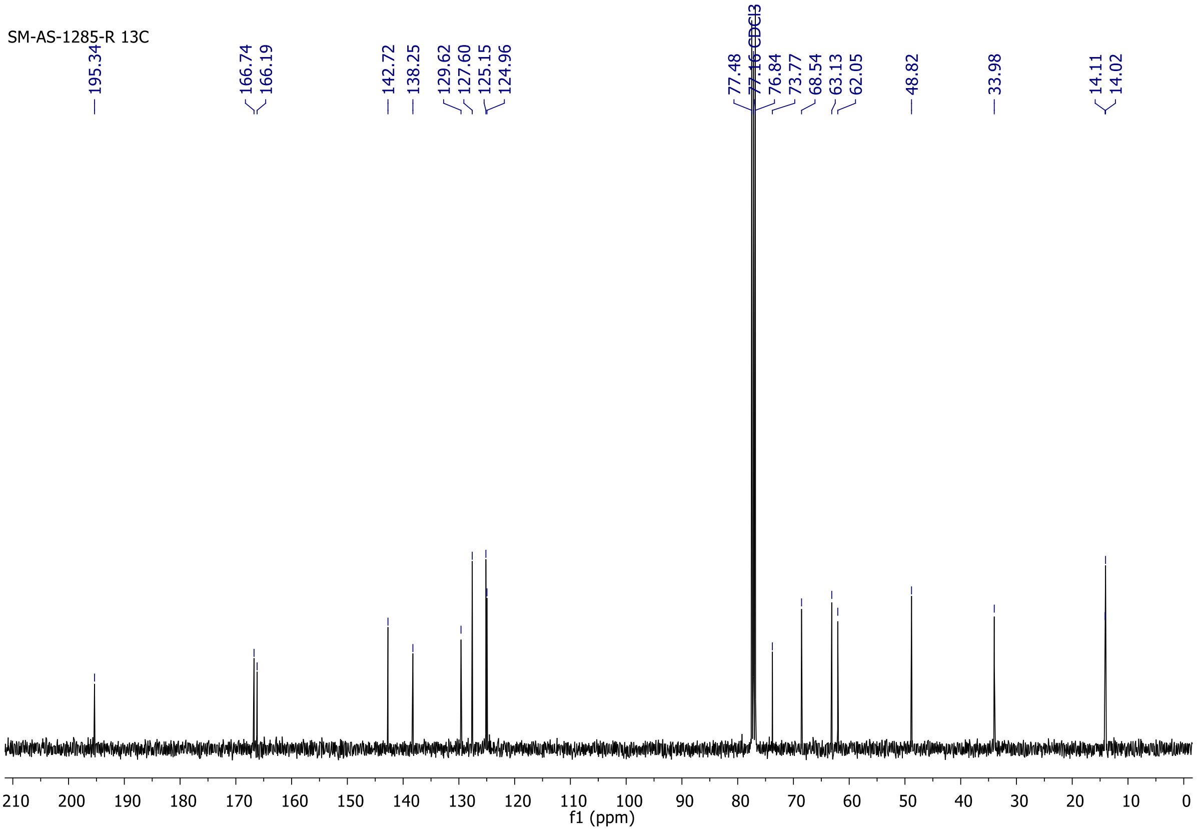

$^1H$-$^1H$ COSY NMR of **4r**:

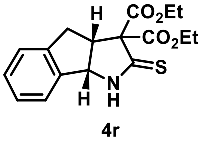


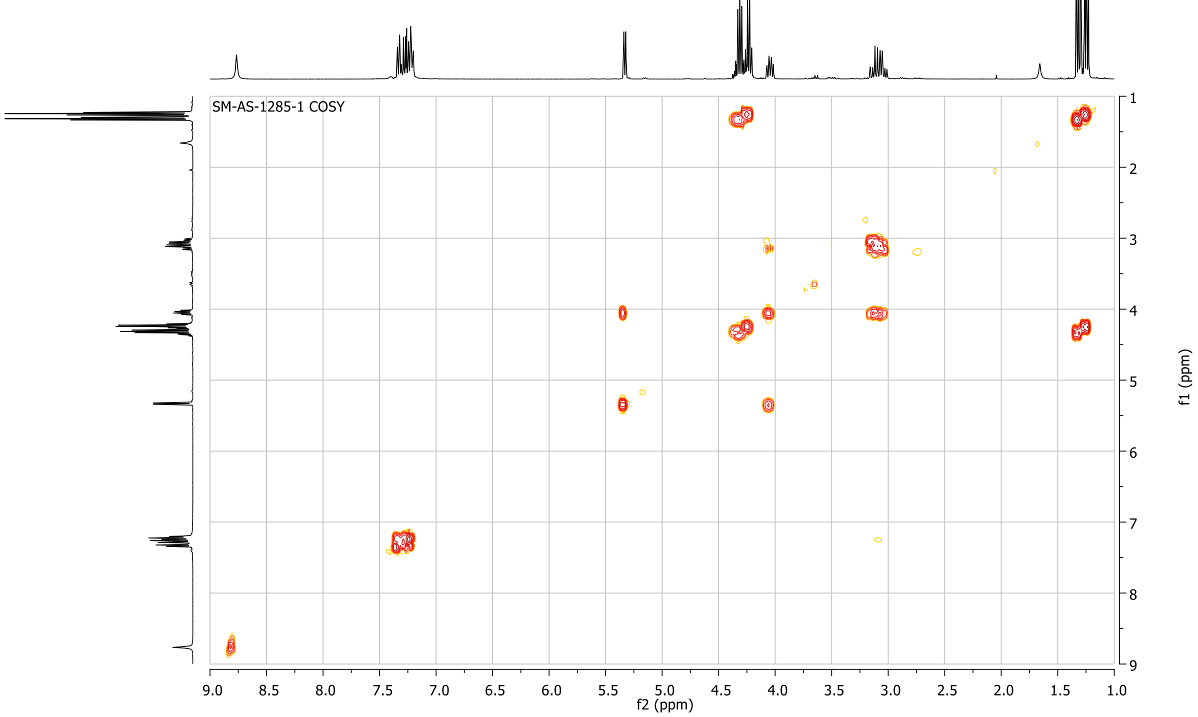


$^1H$-$^{13}C$ HSQC NMR of **4r**:

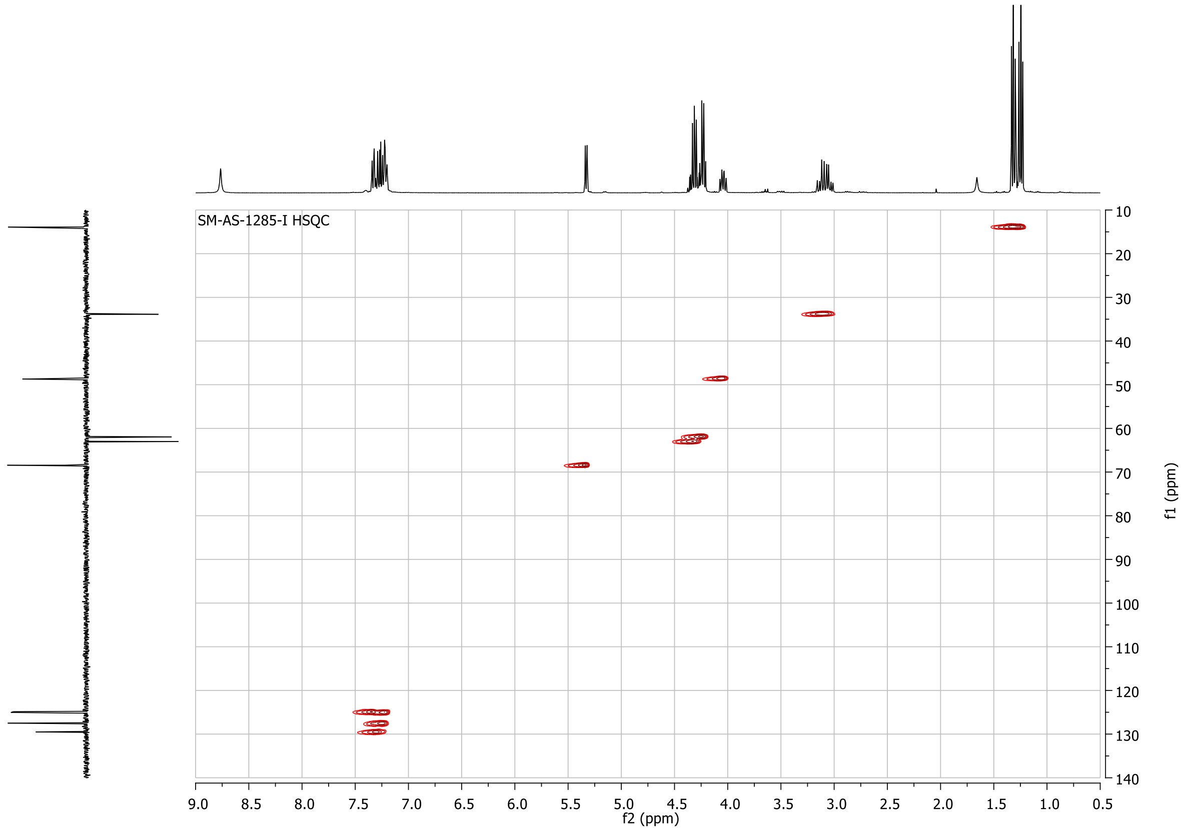

$^{1}H$-$^{1}H$ NOESY NMR of **4r**:

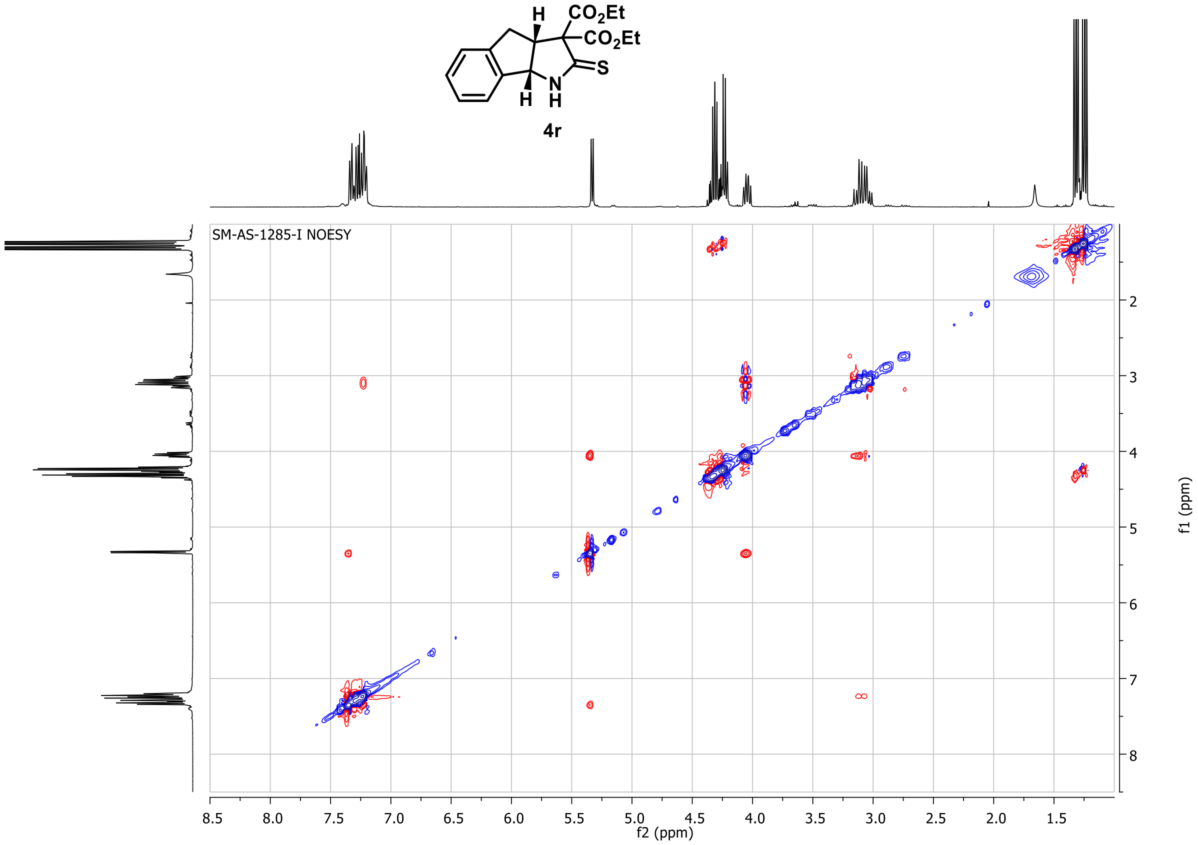

$^{1}H$ NMR of **4s** (400 MHz, $CDCl_3$):

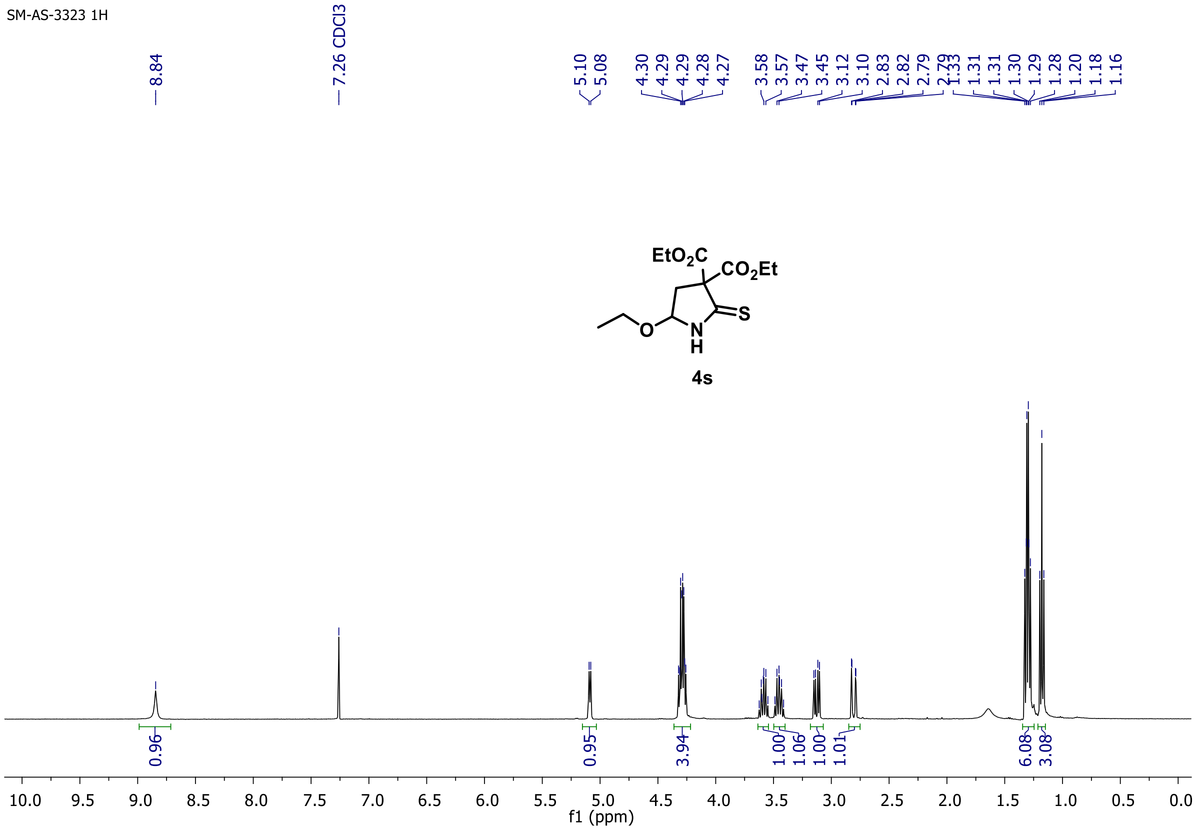


$^{13}C\{^{1}H\}$ NMR of **4s** (101 MHz, $CDCl_3$):

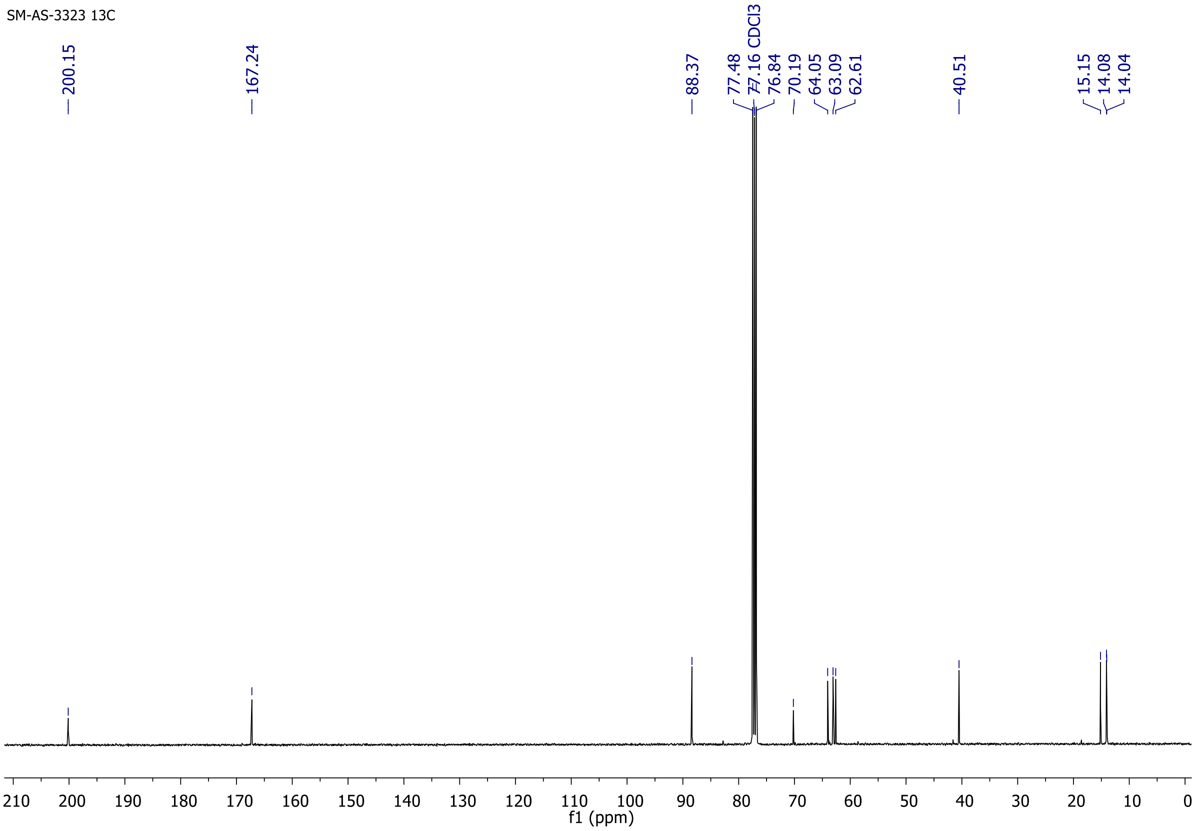

$^1H$ NMR of **4t** (400 MHz, DMSO-d$_6$):

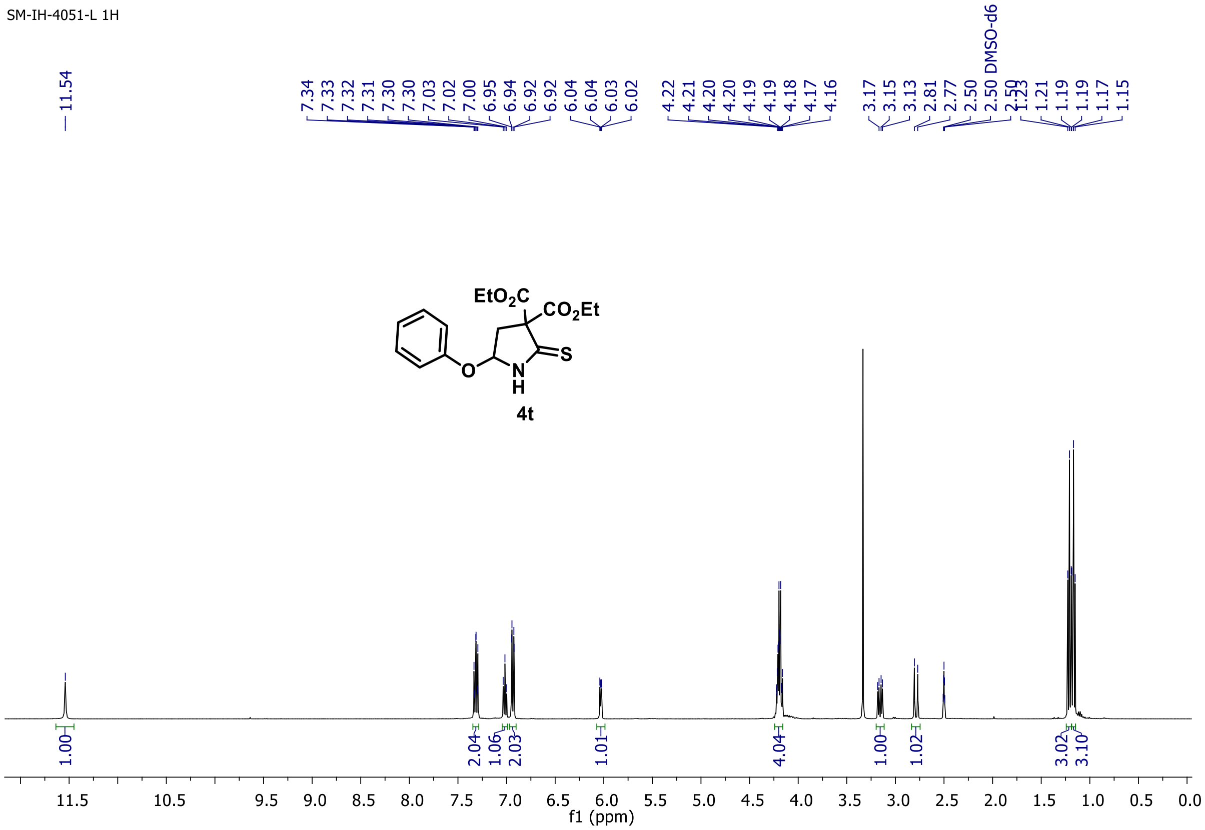


$^{13}C\{^1H\}$ NMR of **4t** (101 MHz, DMSO-d$_6$):

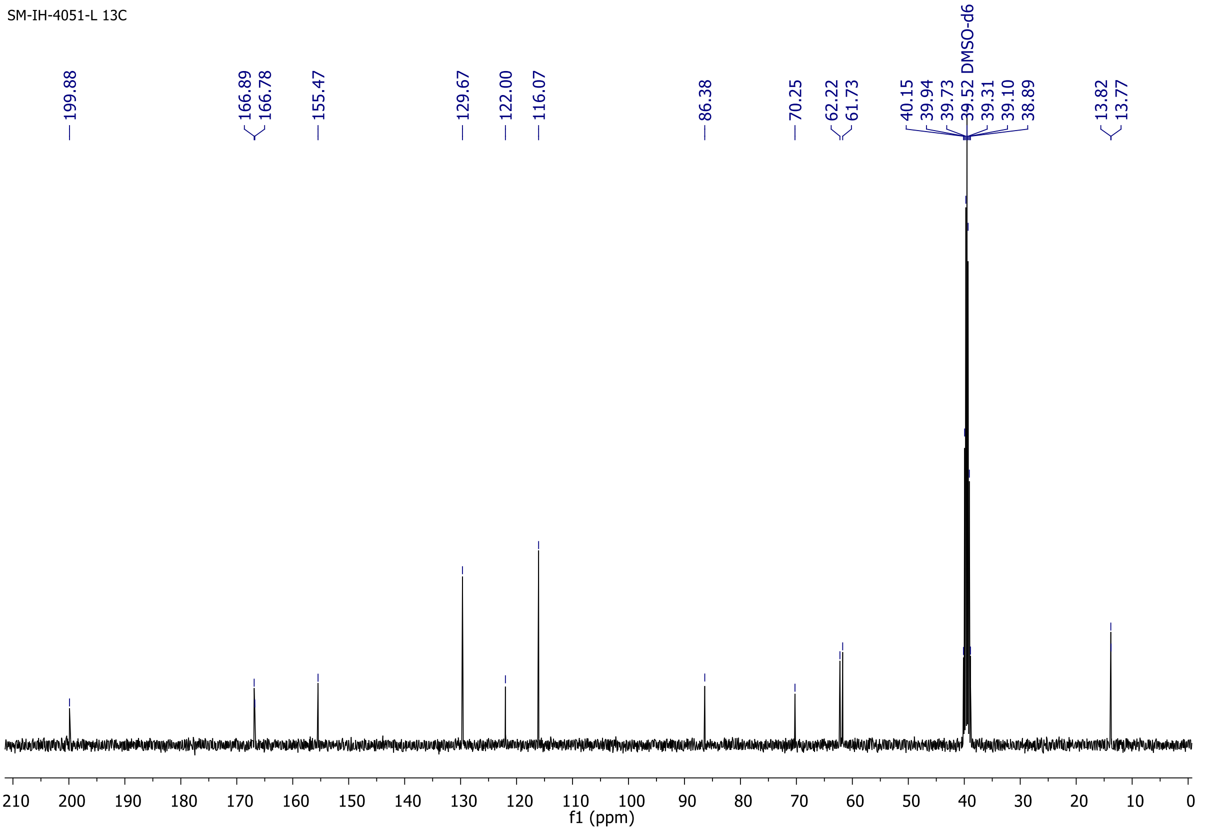

$^{1}H$ NMR of **4u** (400 MHz, $CDCl_3$):

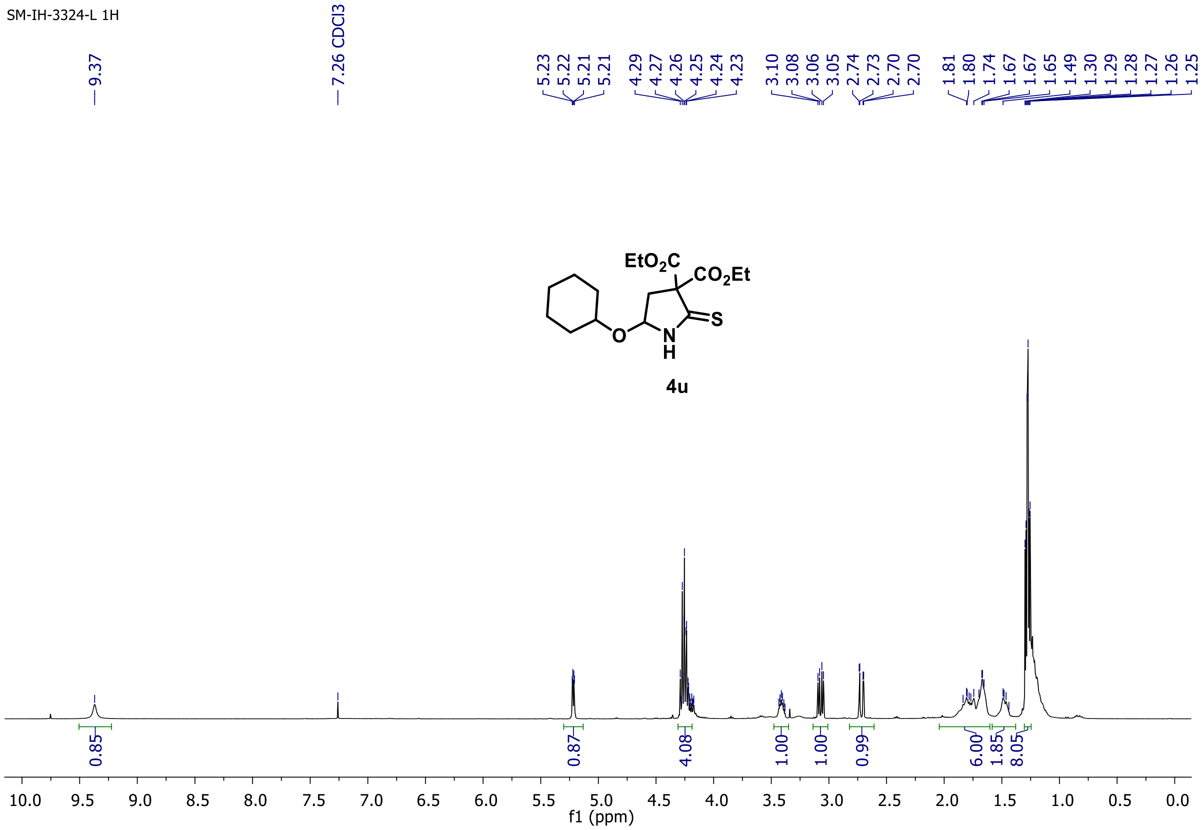


$^{13}C\{^{1}H\}$ NMR of **4u** (101 MHz, $CDCl_3$):

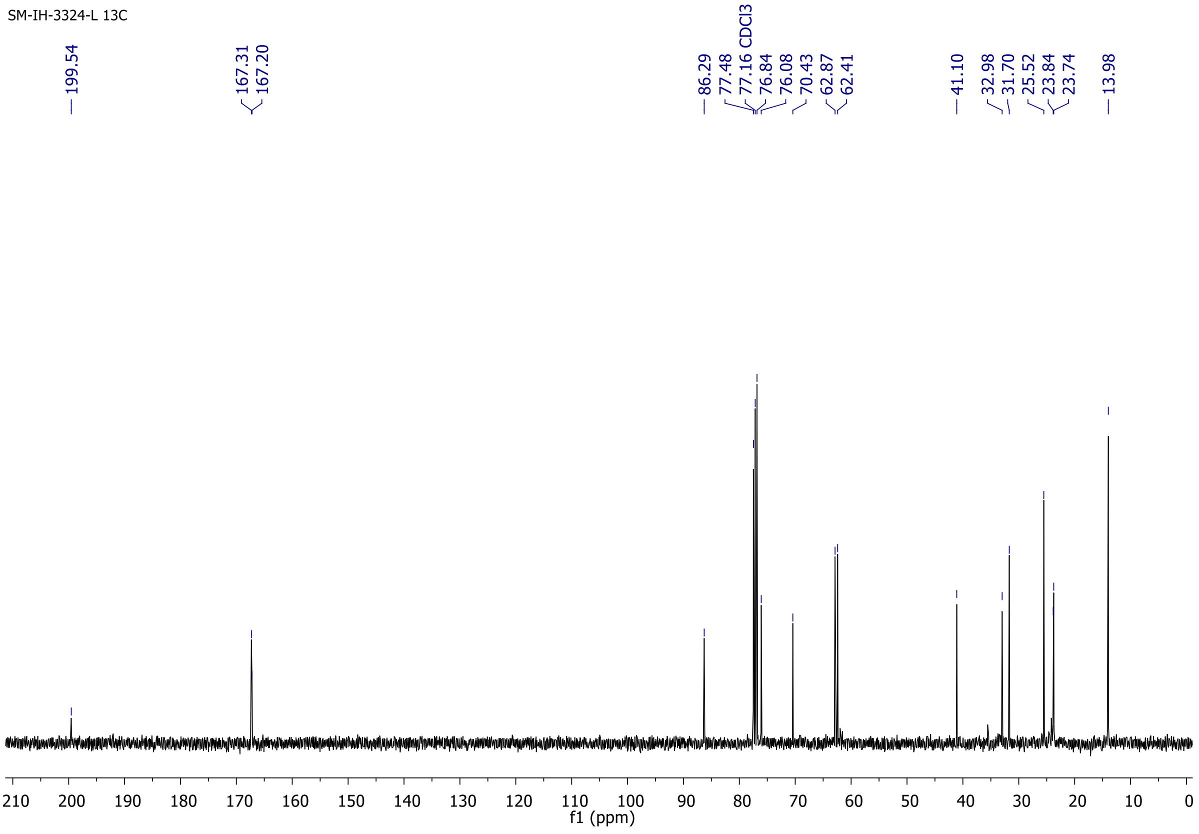

$^{1}$H NMR of **4v** (400 MHz, CDCl$_3$):

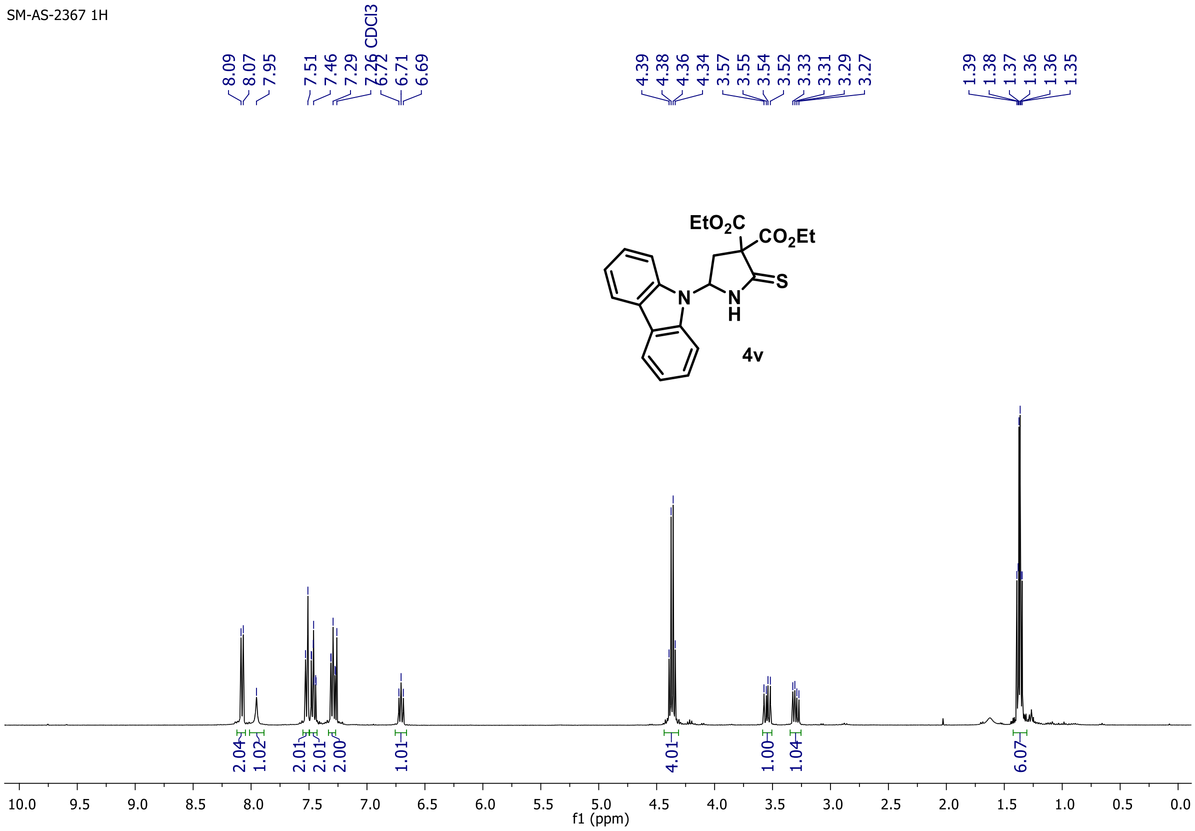


$^{13}$C{$^{1}$H} NMR of **4v** (101 MHz, CDCl$_3$):

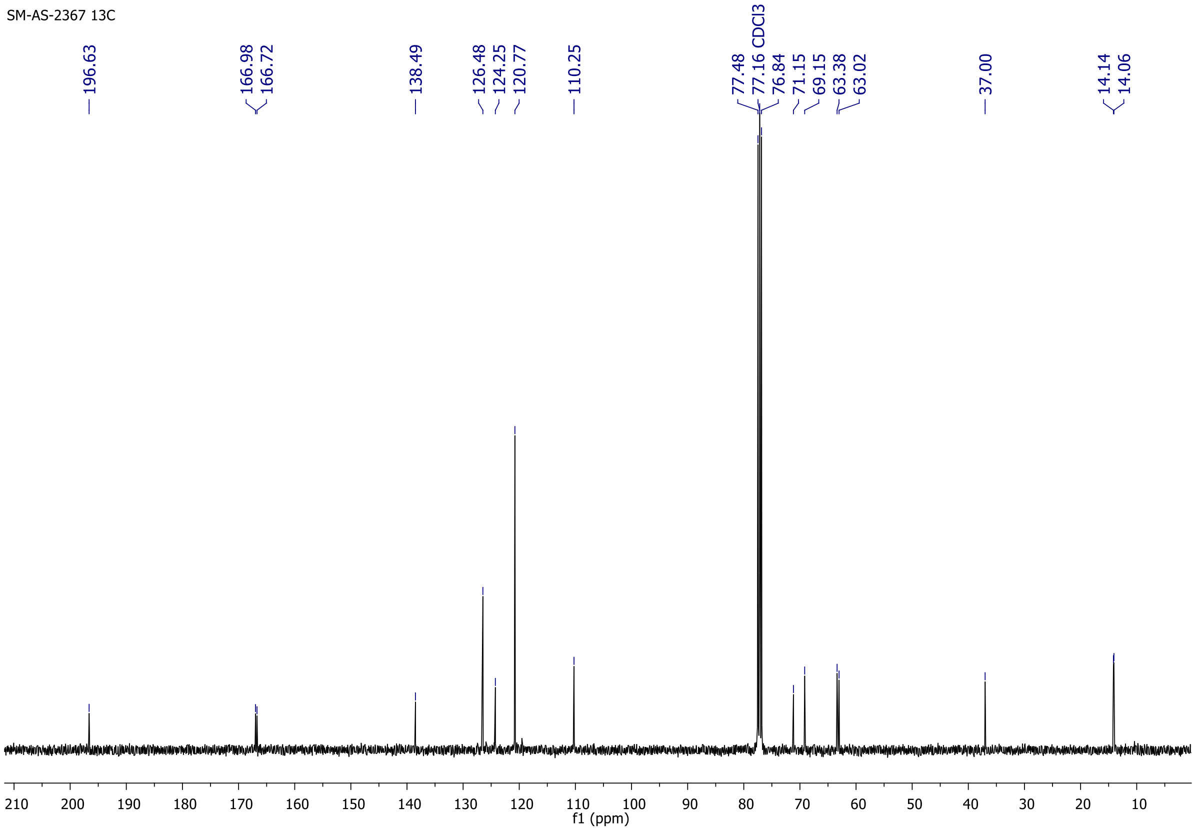

$^{1}H$ NMR of **4w** (400 MHz, $CDCl_3$):

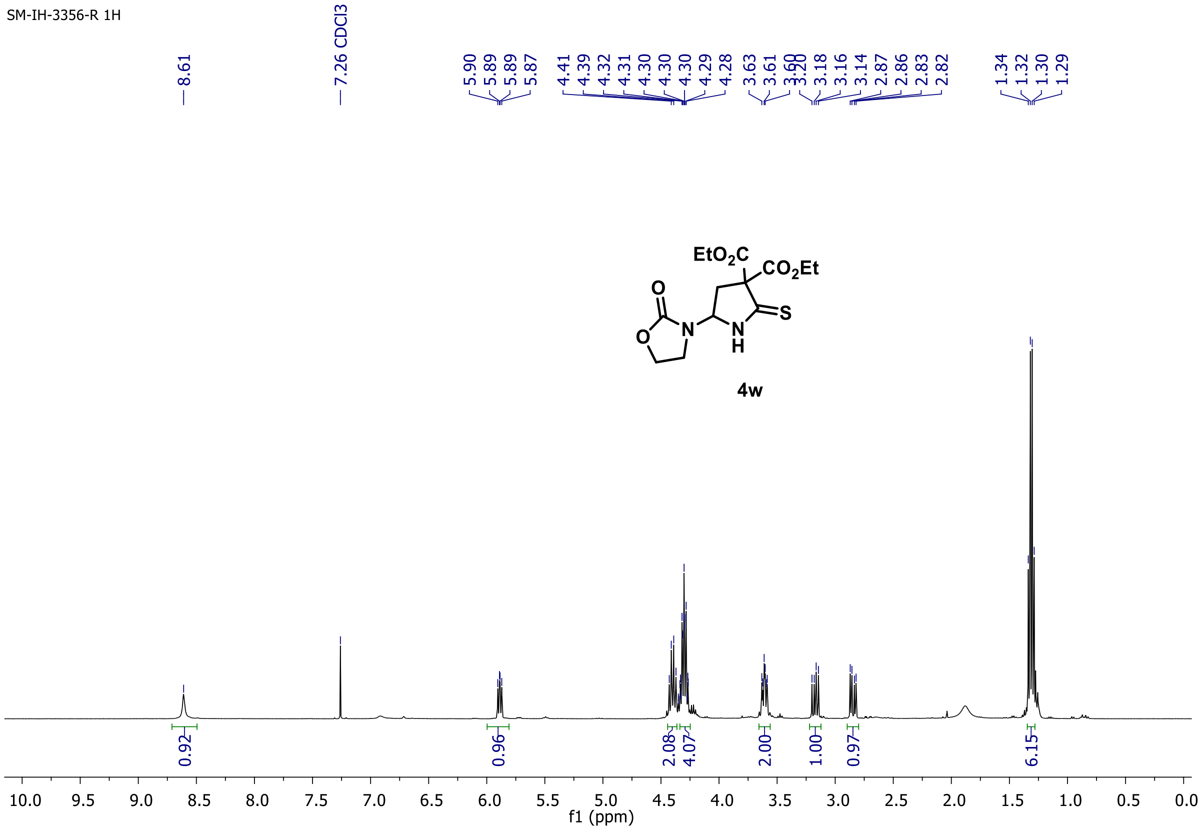


$^{13}C\{^{1}H\}$ NMR of **4w** (101 MHz, $CDCl_3$):

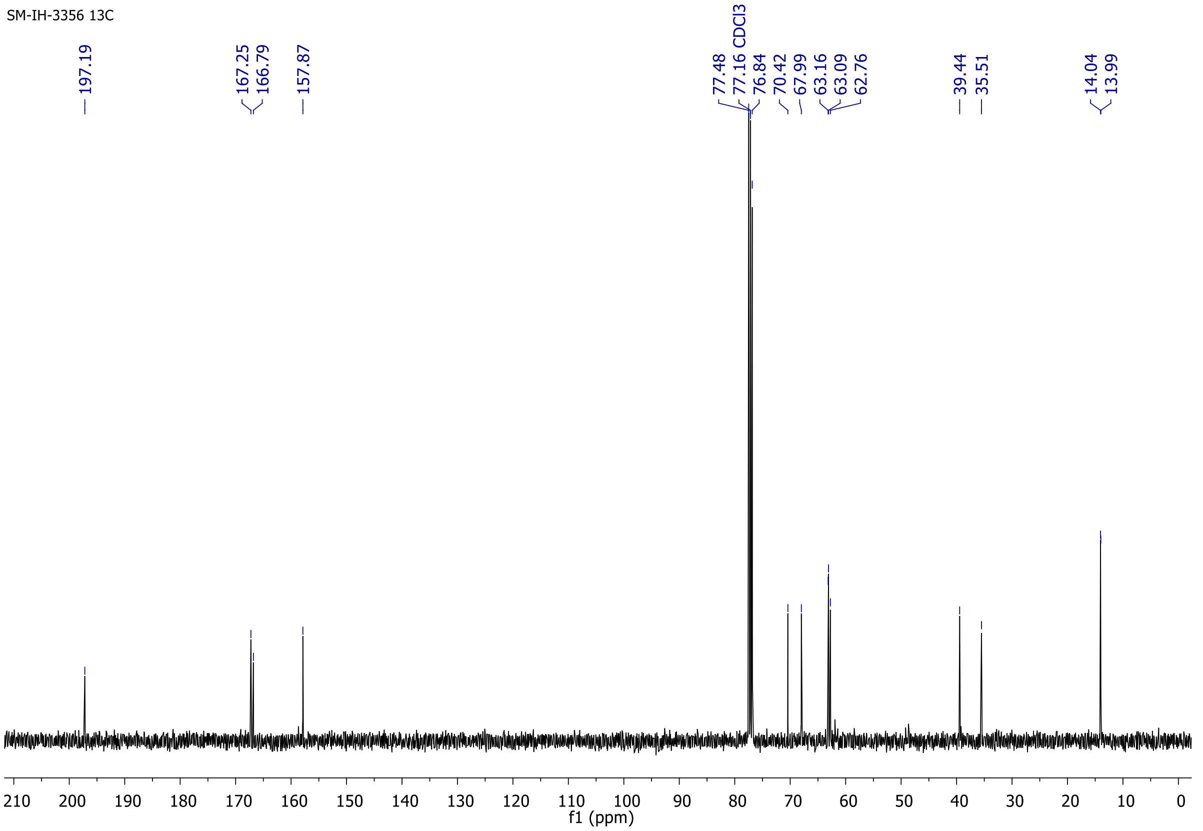

$^{1}$H NMR of **4x** (400 MHz, $CDCl_3$):

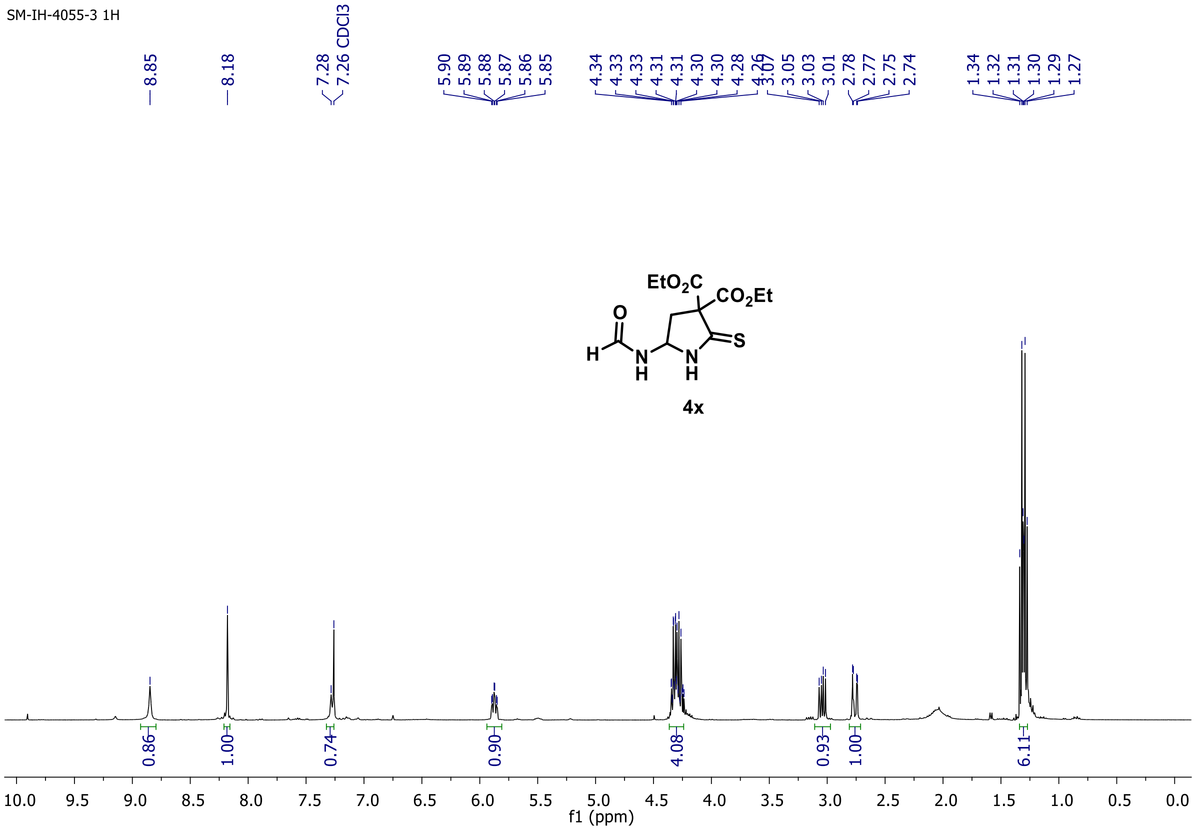


$^{13}$C{$^{1}$H} NMR of **4x** (101 MHz, $CDCl_3$):

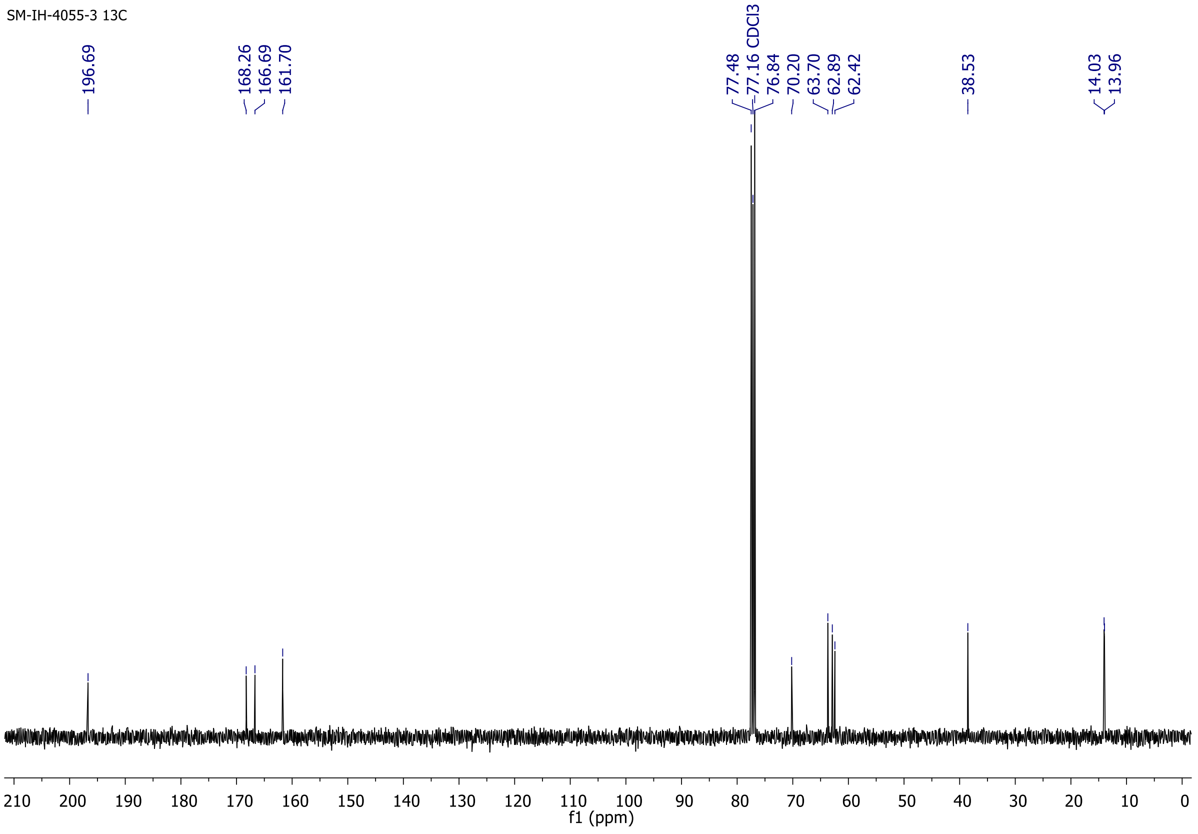

$^{1}H$ NMR of **4y** (400 MHz, $CDCl_3$):

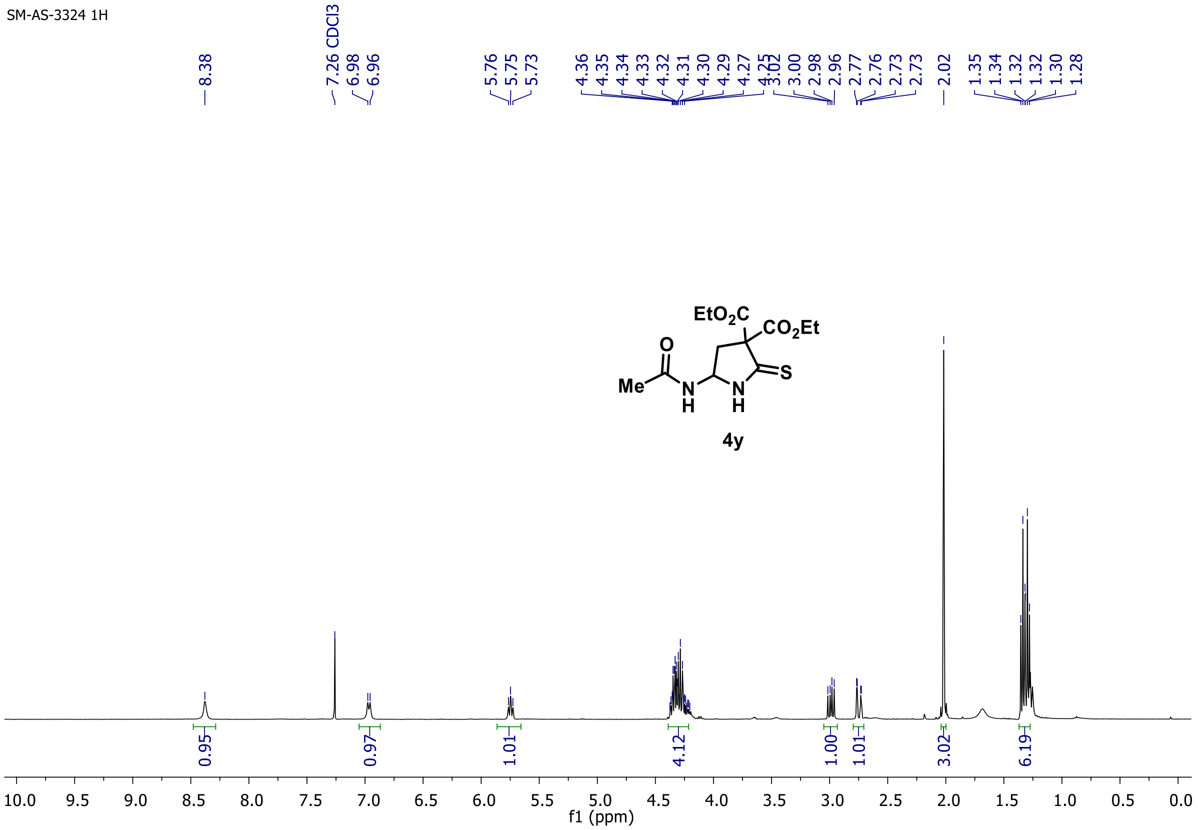


$^{13}C\{^{1}H\}$ NMR of **4y** (101 MHz, $CDCl_3$):

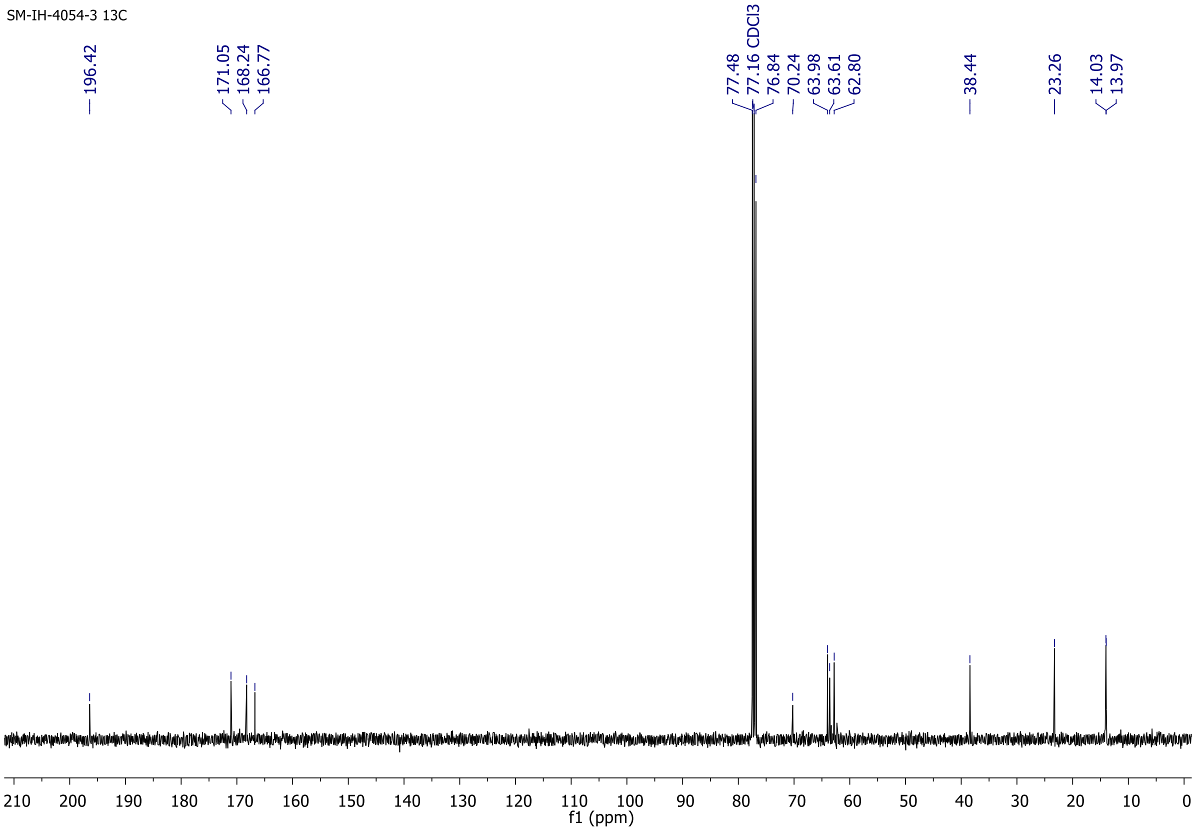

$^{1}H$ NMR of **4z** (400 MHz, $CDCl_3$):

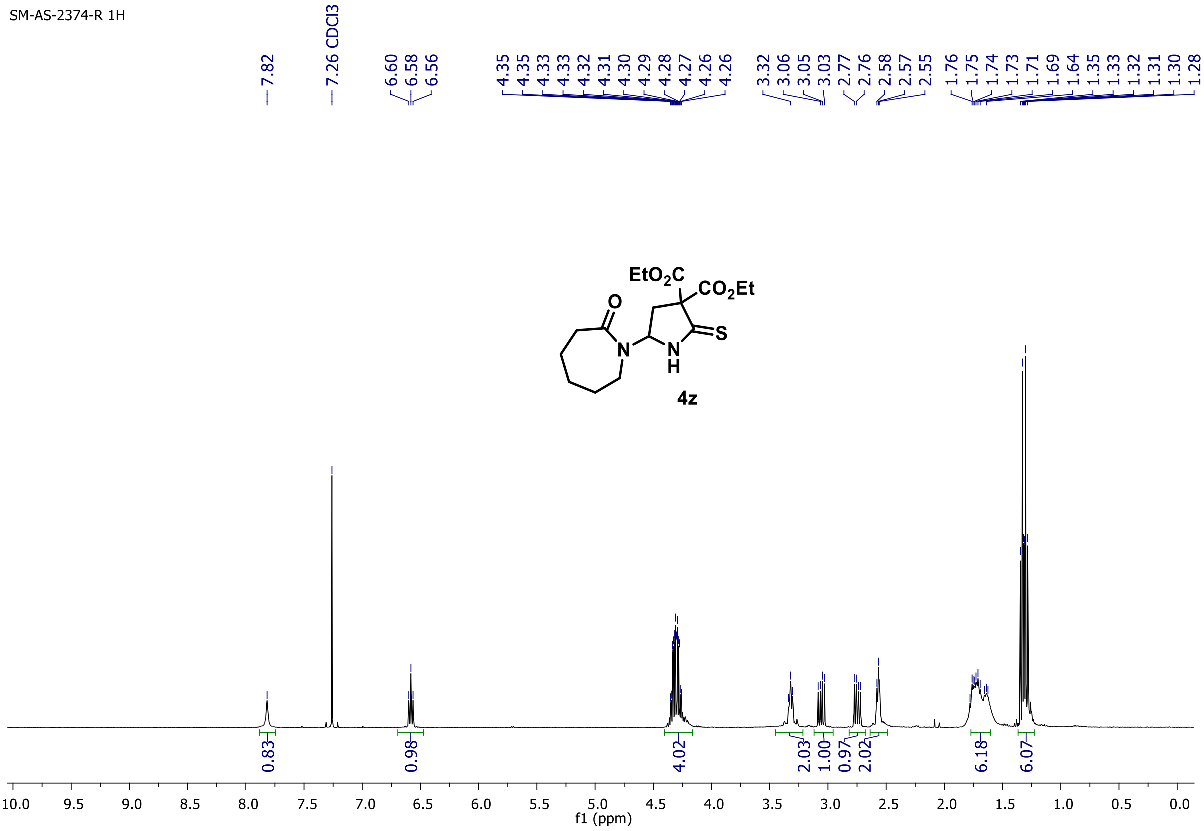


$^{13}C\{^{1}H\}$ NMR of **4z** (101 MHz, $CDCl_3$):

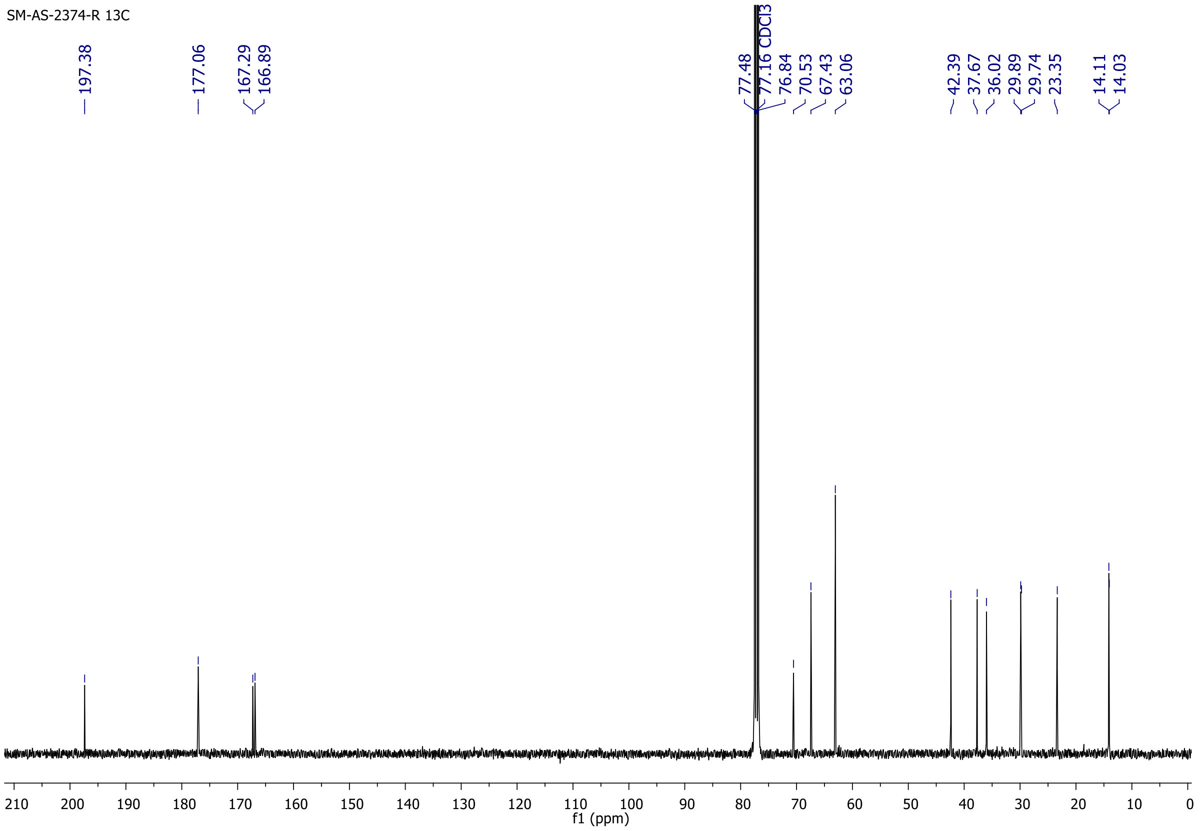

$^{1}H$ NMR of **4aa** (400 MHz, $CDCl_3$):

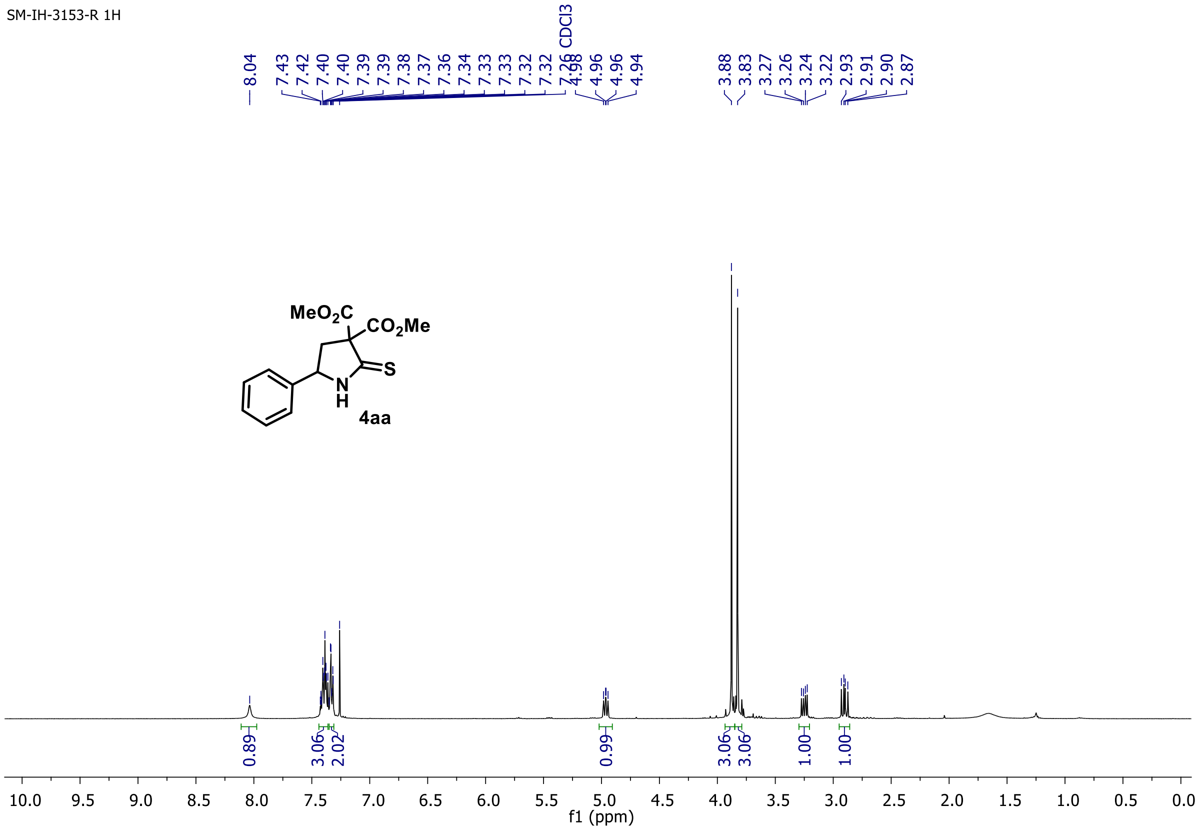


$^{13}C\{^{1}H\}$ NMR of **4aa** (101 MHz, $CDCl_3$):

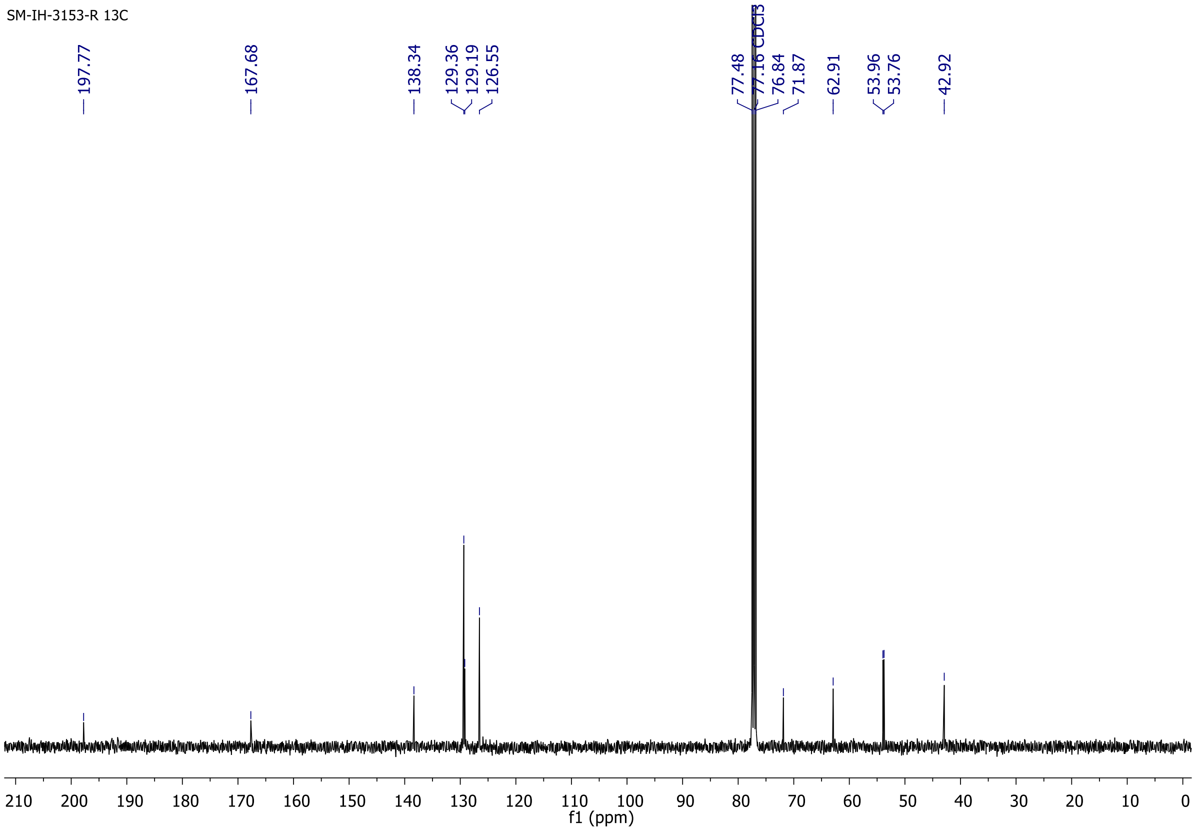

$^1H$ NMR of **4ab** (400 MHz, $CDCl_3$):

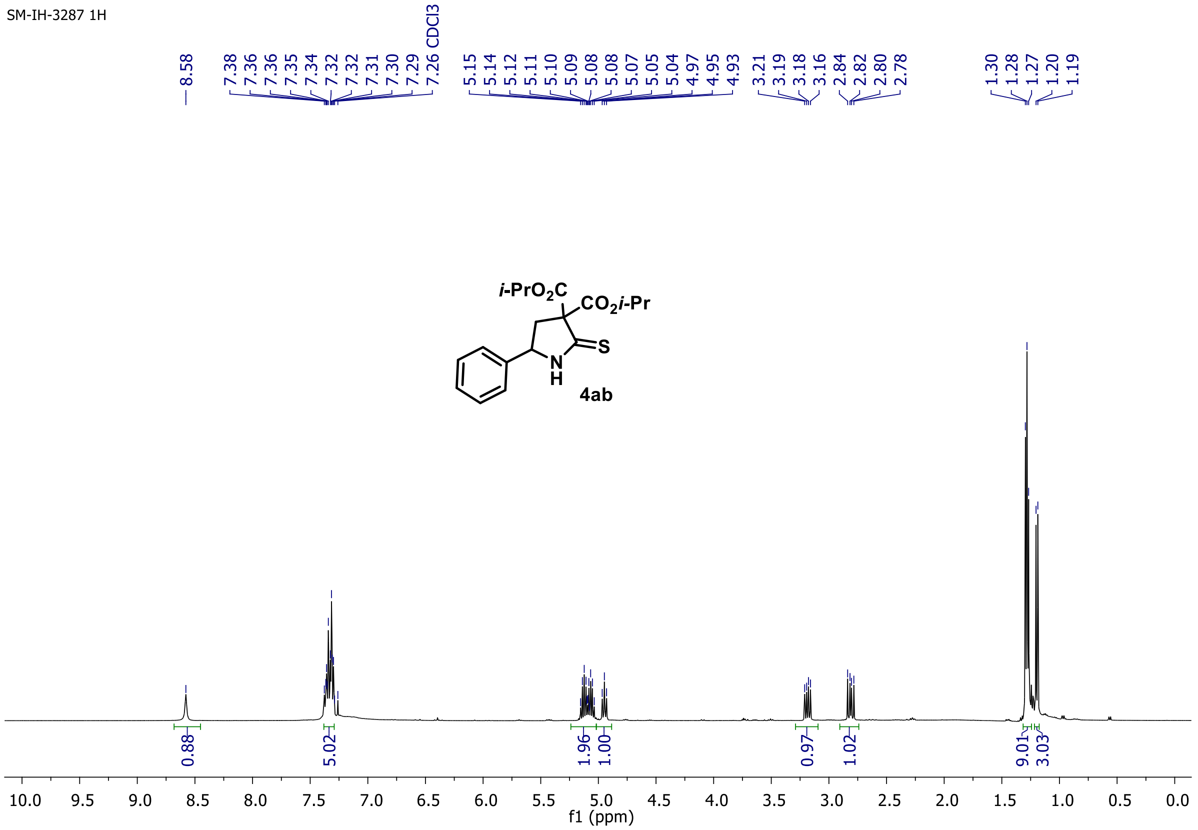


$^{13}C\{^1H\}$ NMR of **4ab** (101 MHz, $CDCl_3$):

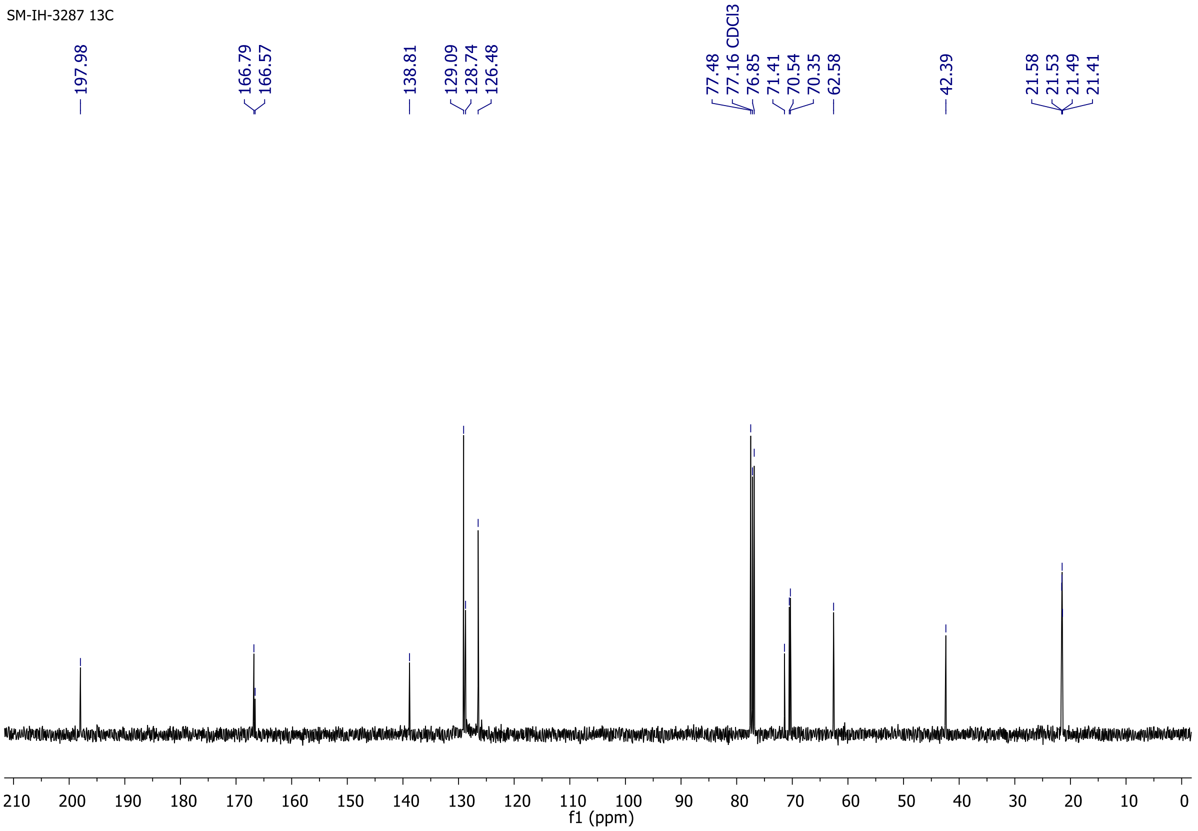

$^1$H NMR of **4ac** (400 MHz, $CDCl_3$):

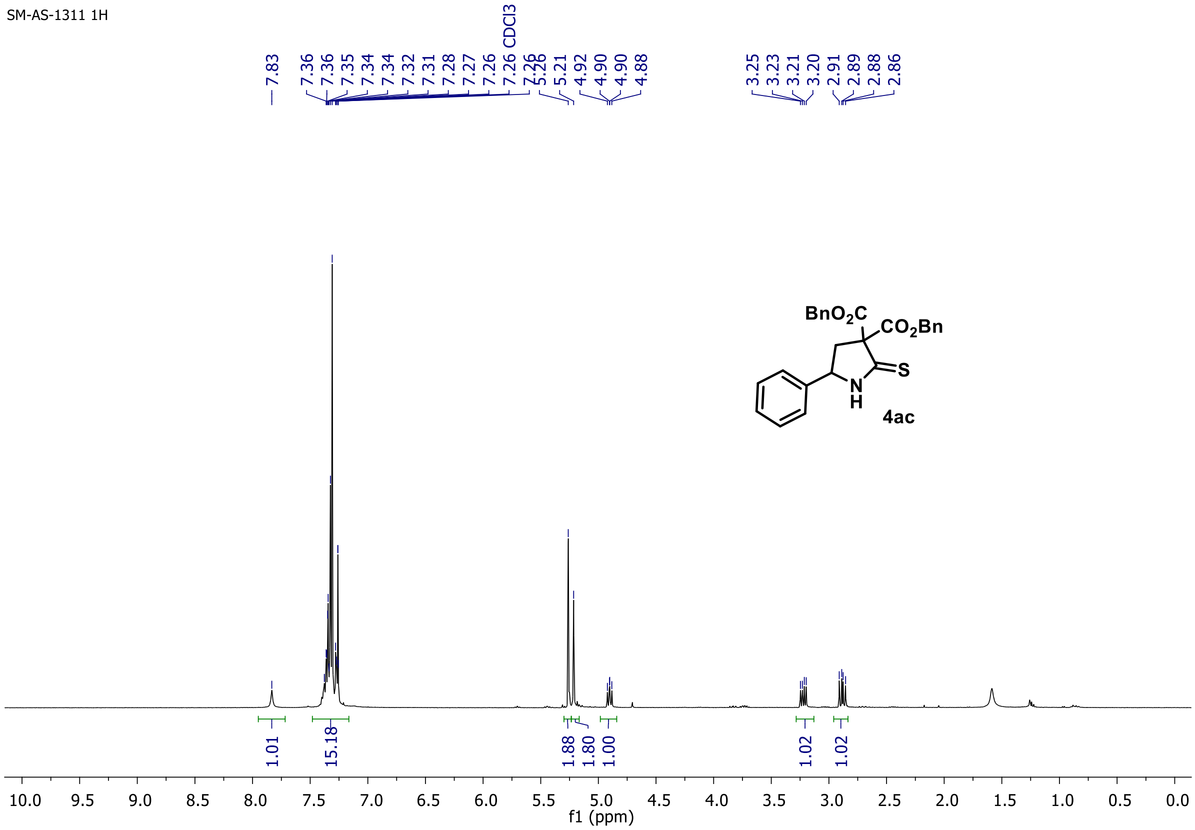


$^{13}C\{^1H\}$ NMR of **4ac** (101 MHz, $CDCl_3$):

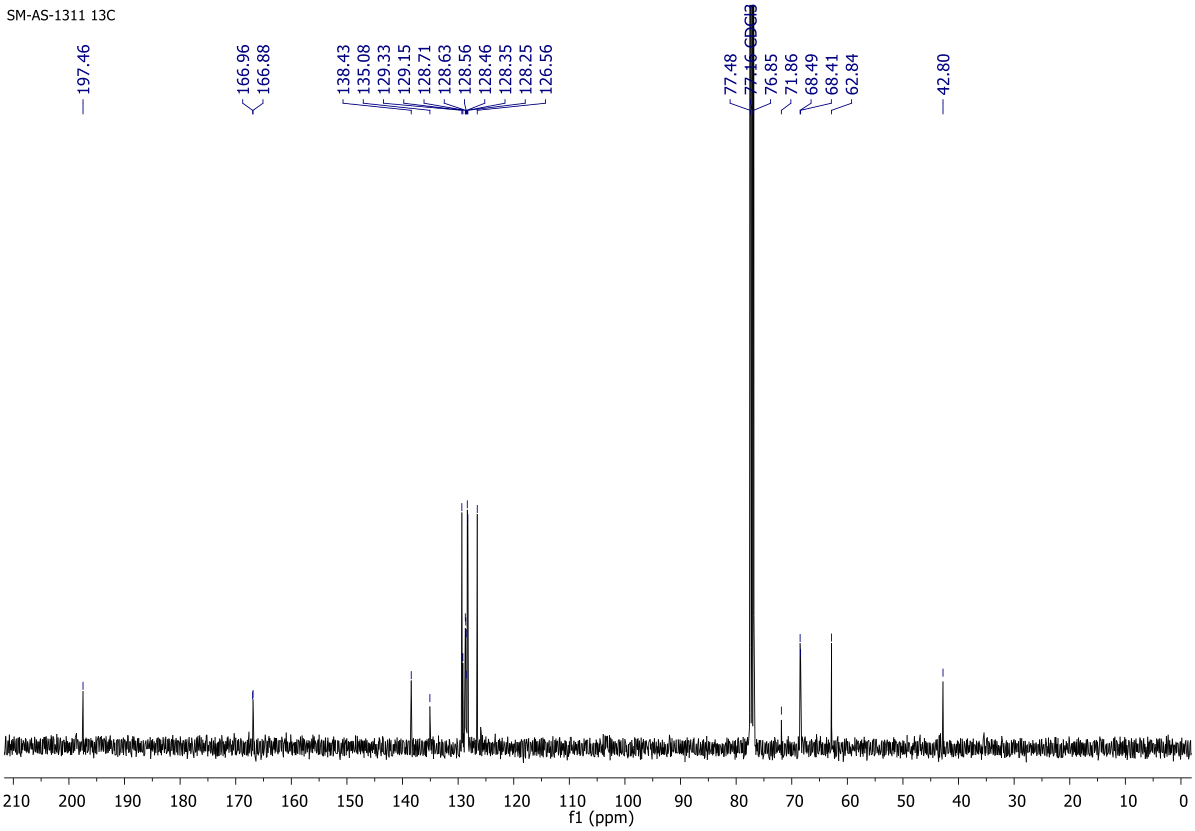

$^{1}$H NMR of **4ad** (400 MHz, $CDCl_3$):

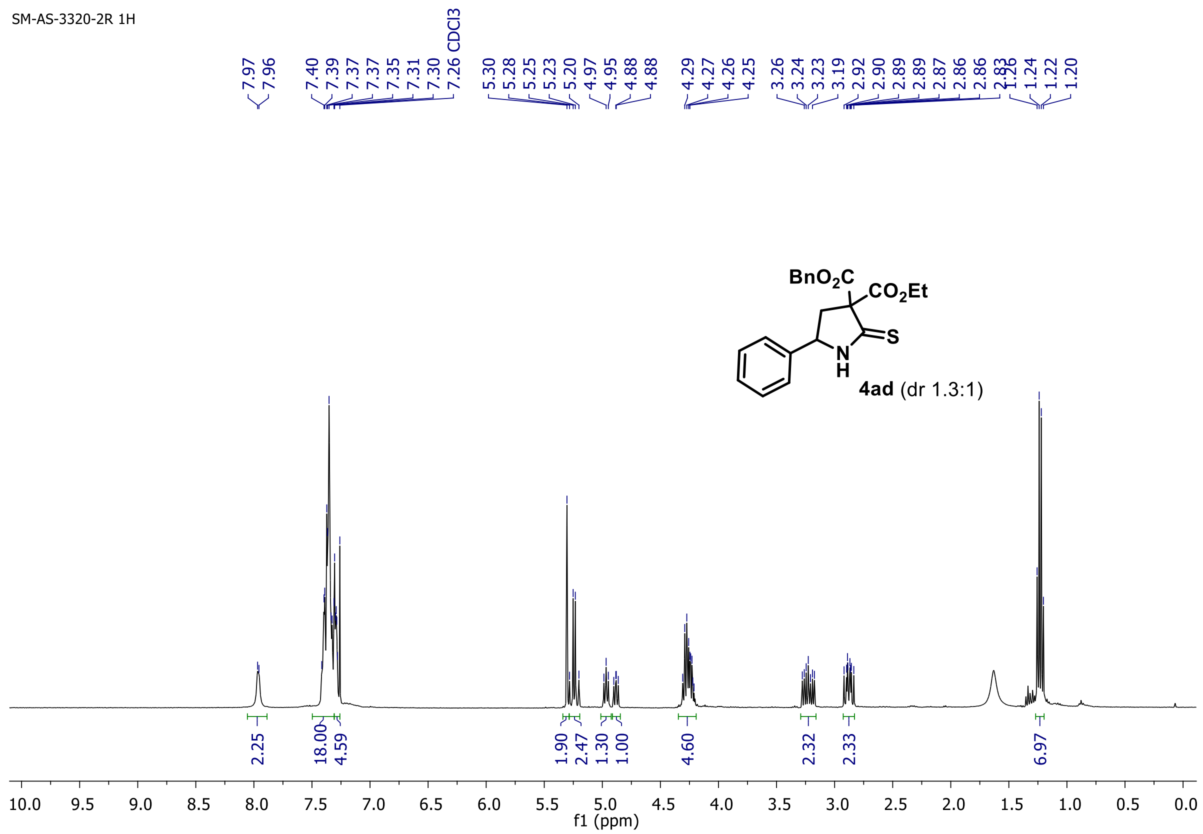


$^{13}$C{$^{1}$H} NMR of **4ad** (101 MHz, $CDCl_3$):

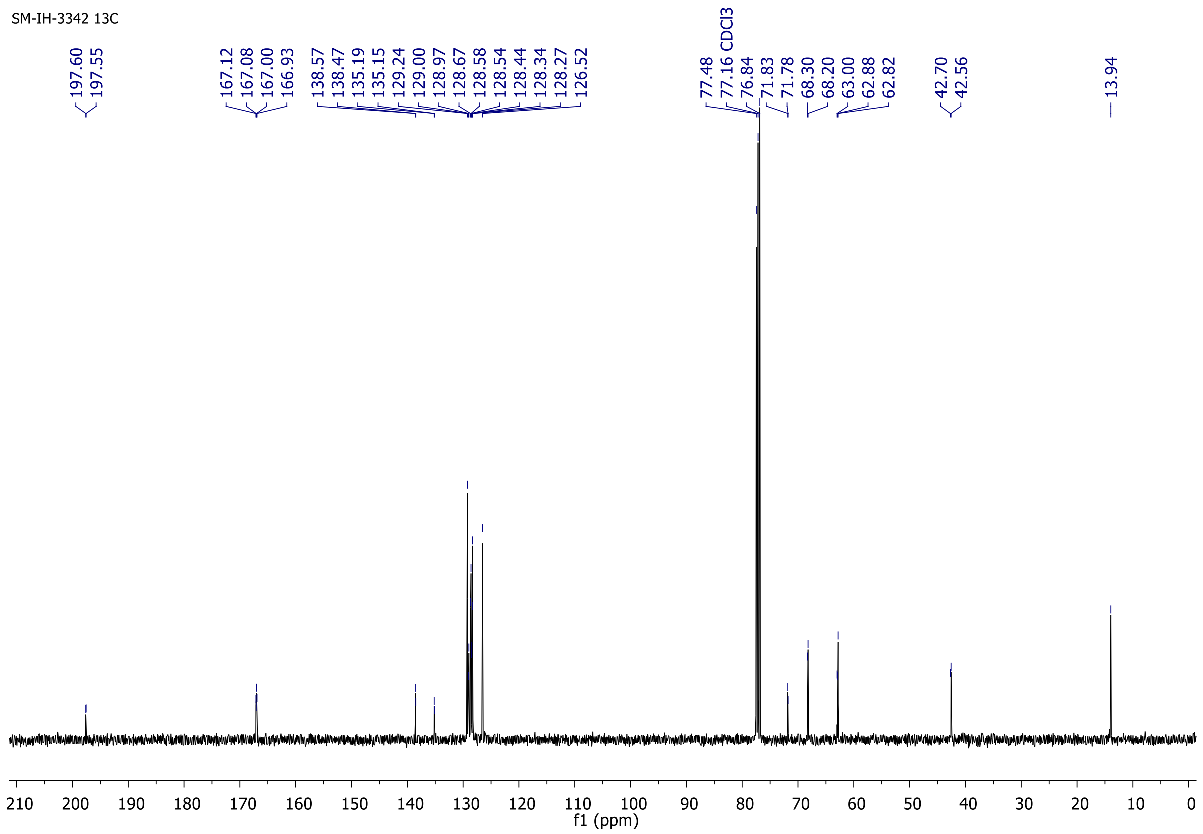

$^1$H NMR of **4ae** (400 MHz, $CDCl_3$):

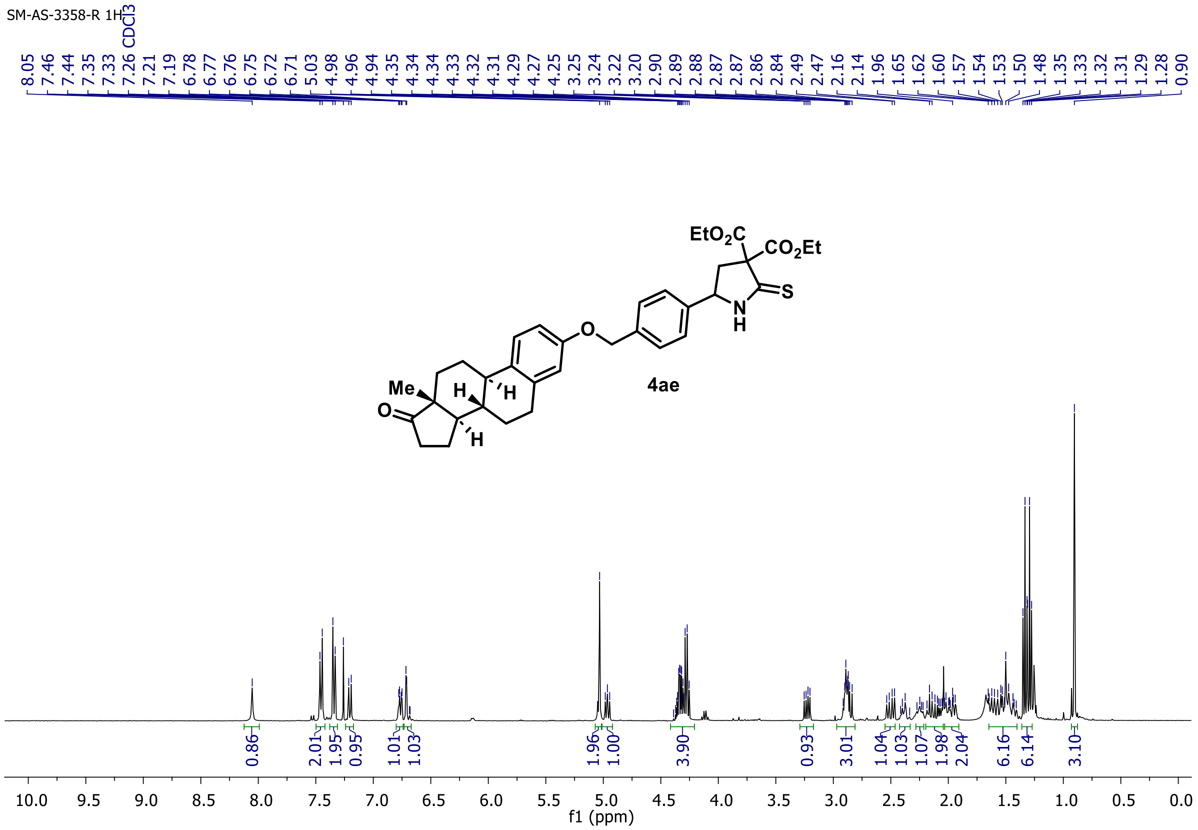


$^{13}C\{^1H\}$ NMR of **4ae** (101 MHz, $CDCl_3$):

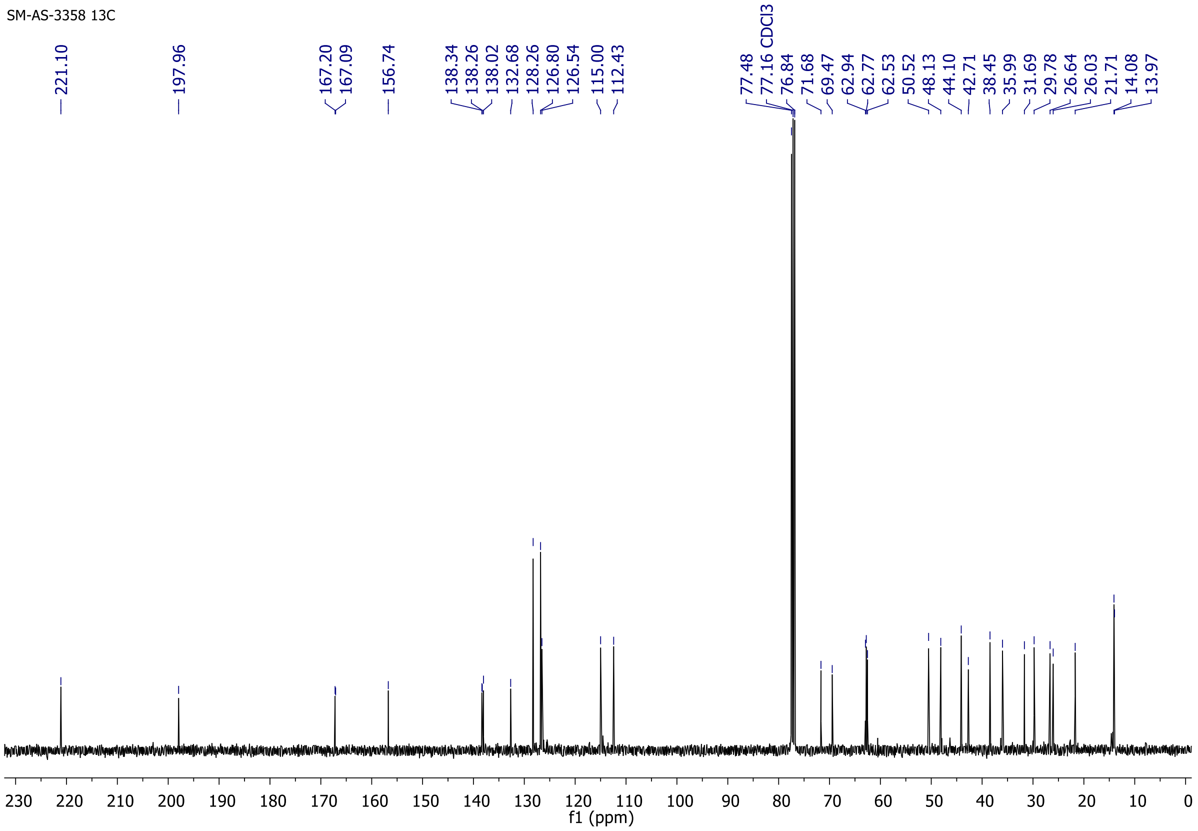

$^{1}H$ NMR of **4af** (400 MHz, $CDCl_3$):

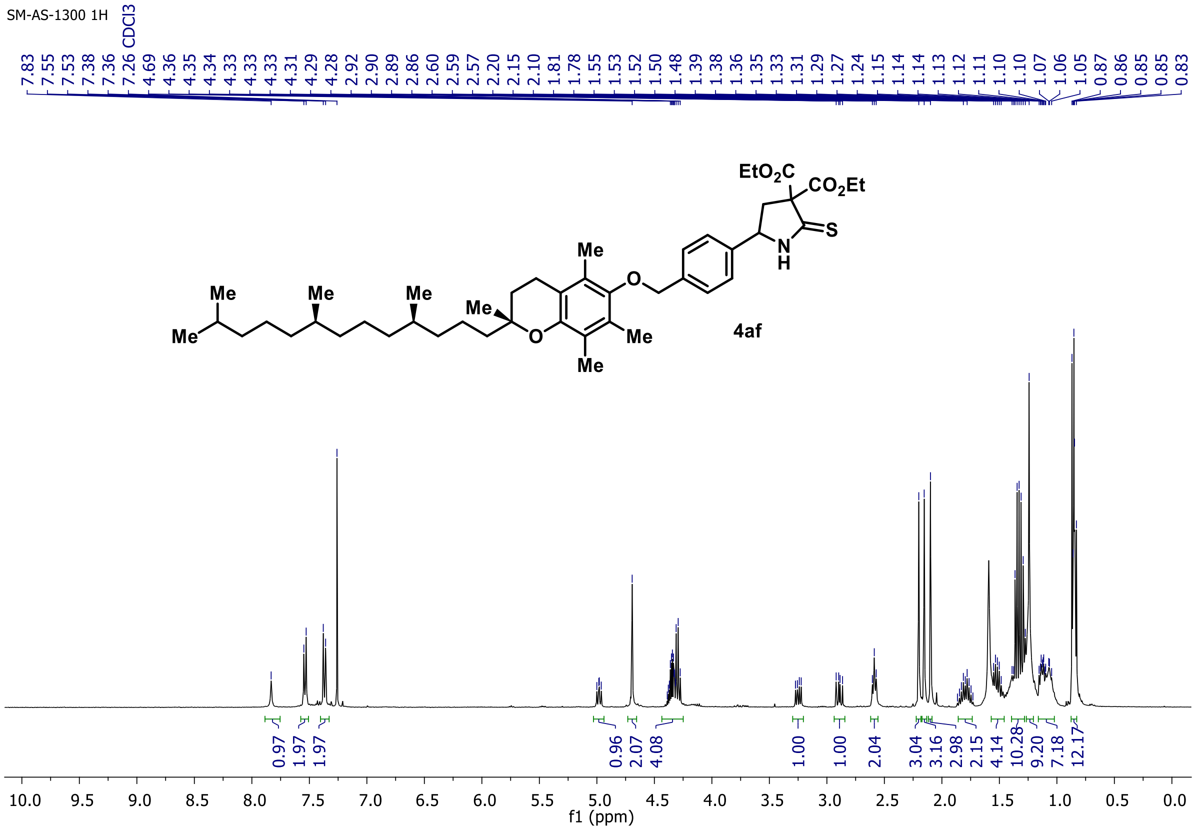


$^{13}C\{^{1}H\}$ NMR of **4af** (101 MHz, $CDCl_3$):

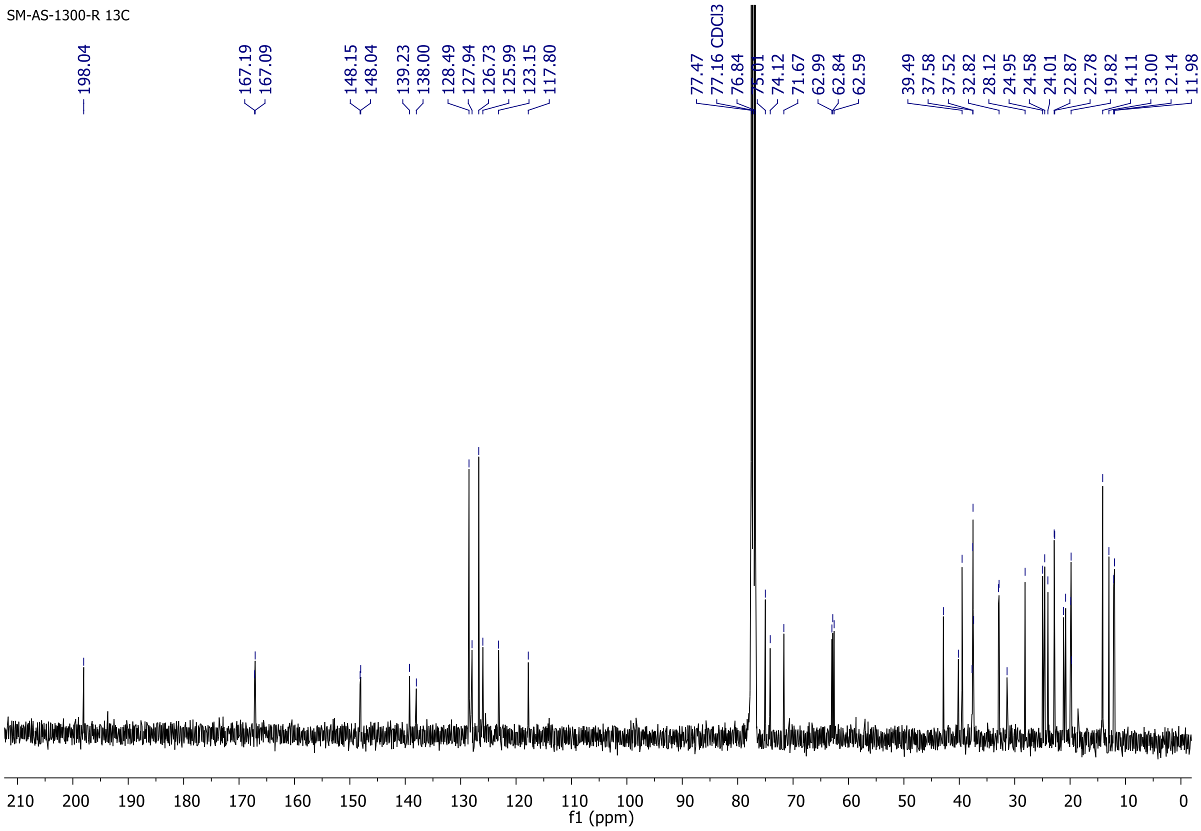

$^{1}$H NMR of **4ag** (400 MHz, $CDCl_3$):

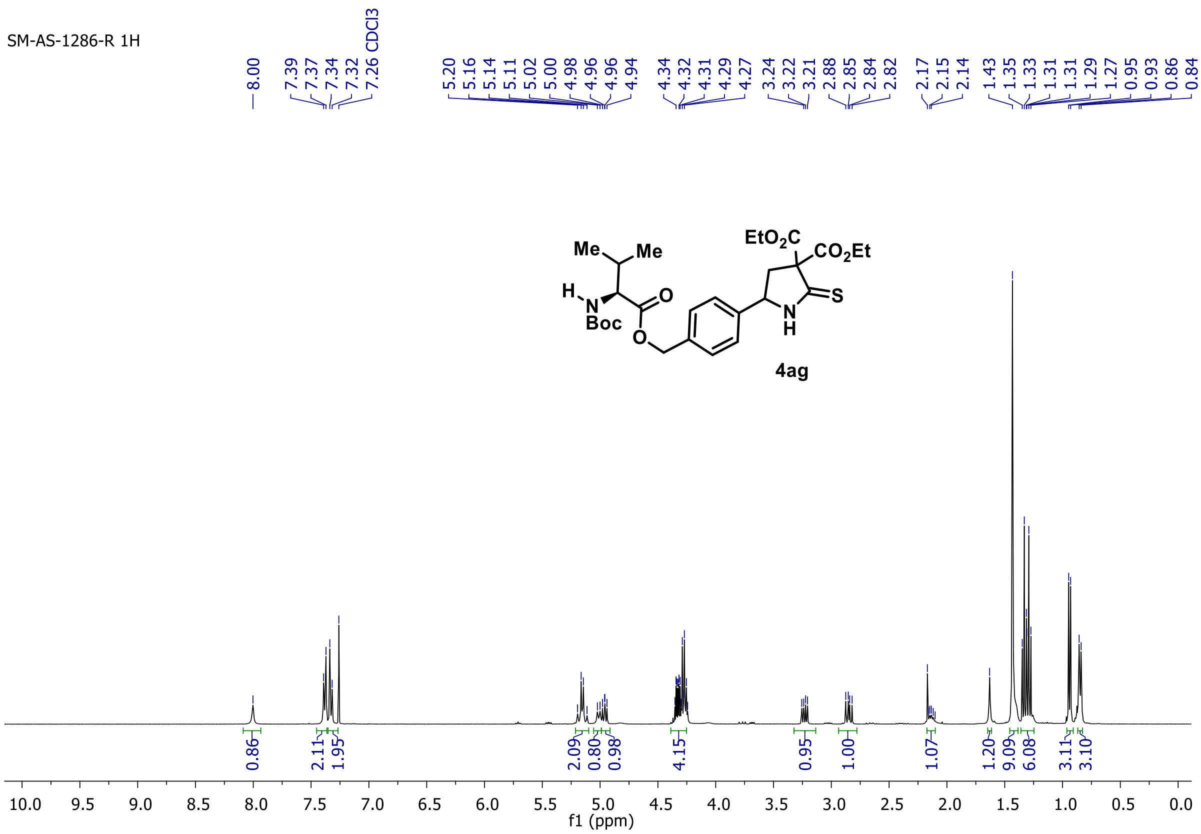


$^{13}$C{$^{1}$H} NMR of **4ag** (101 MHz, $CDCl_3$):

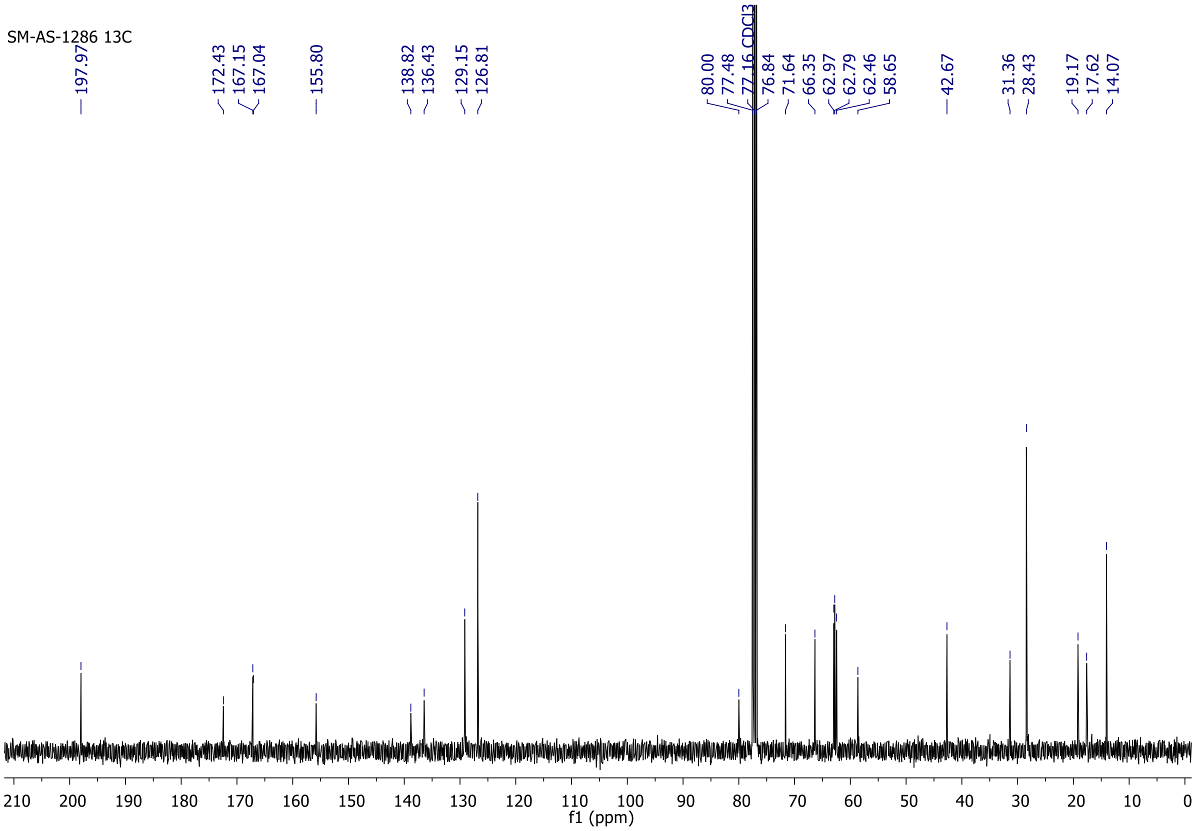

$^{1}H$ NMR of **4ah** (400 MHz, $CDCl_3$):

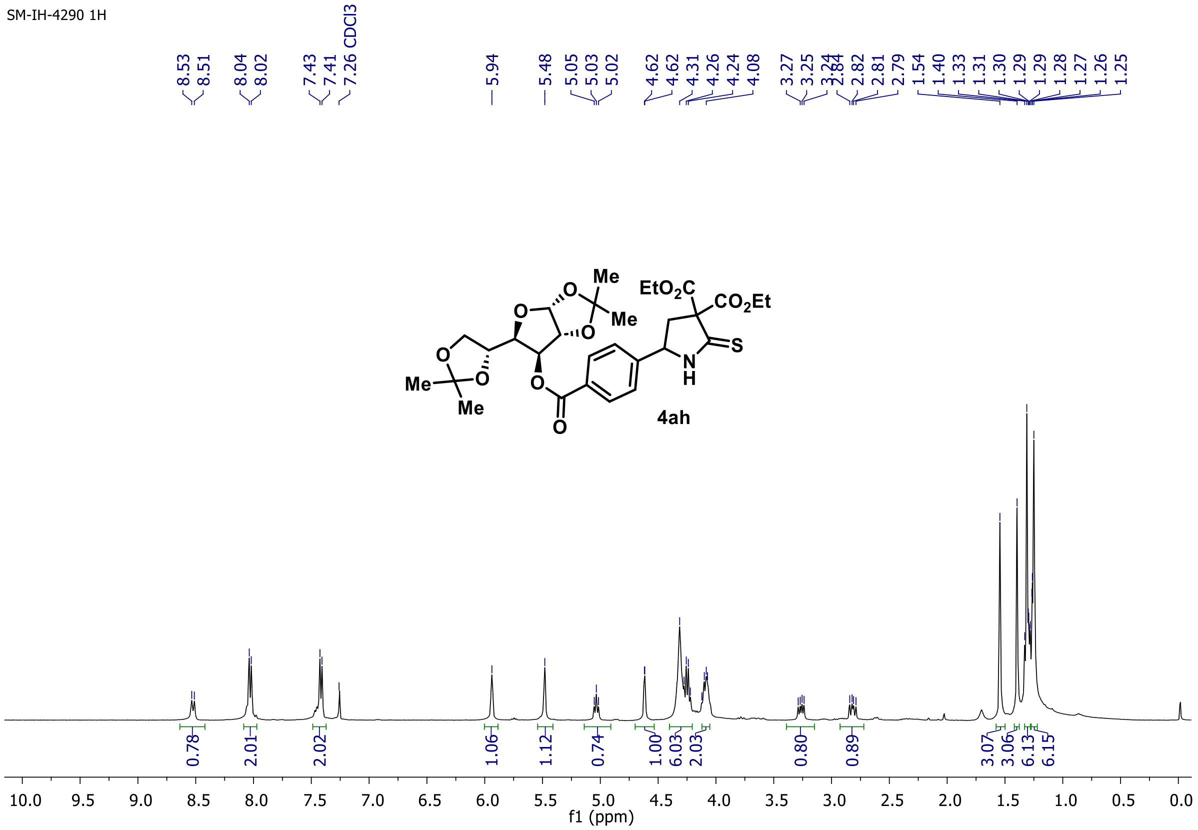


$^{13}C\{^{1}H\}$ NMR of **4ah** (101 MHz, $CDCl_3$):

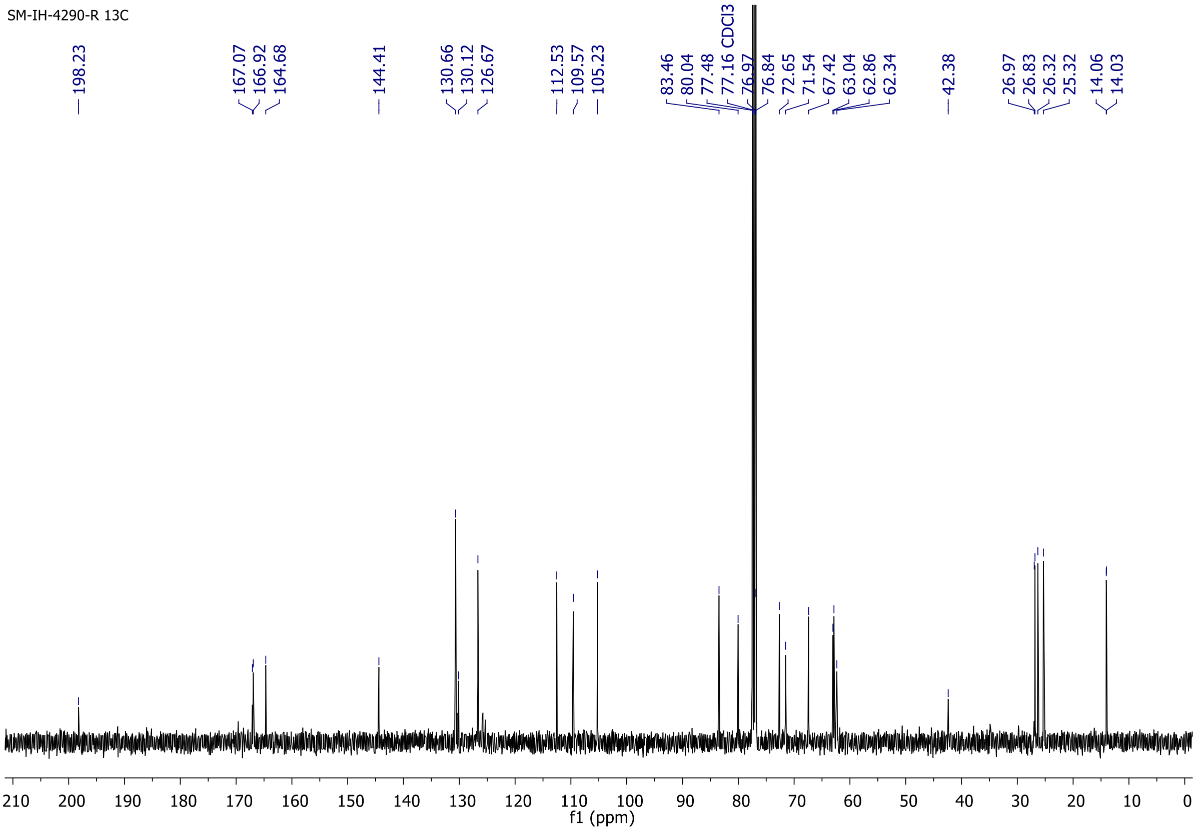

$^1$H NMR of **5a** (400 MHz, $CDCl_3$):

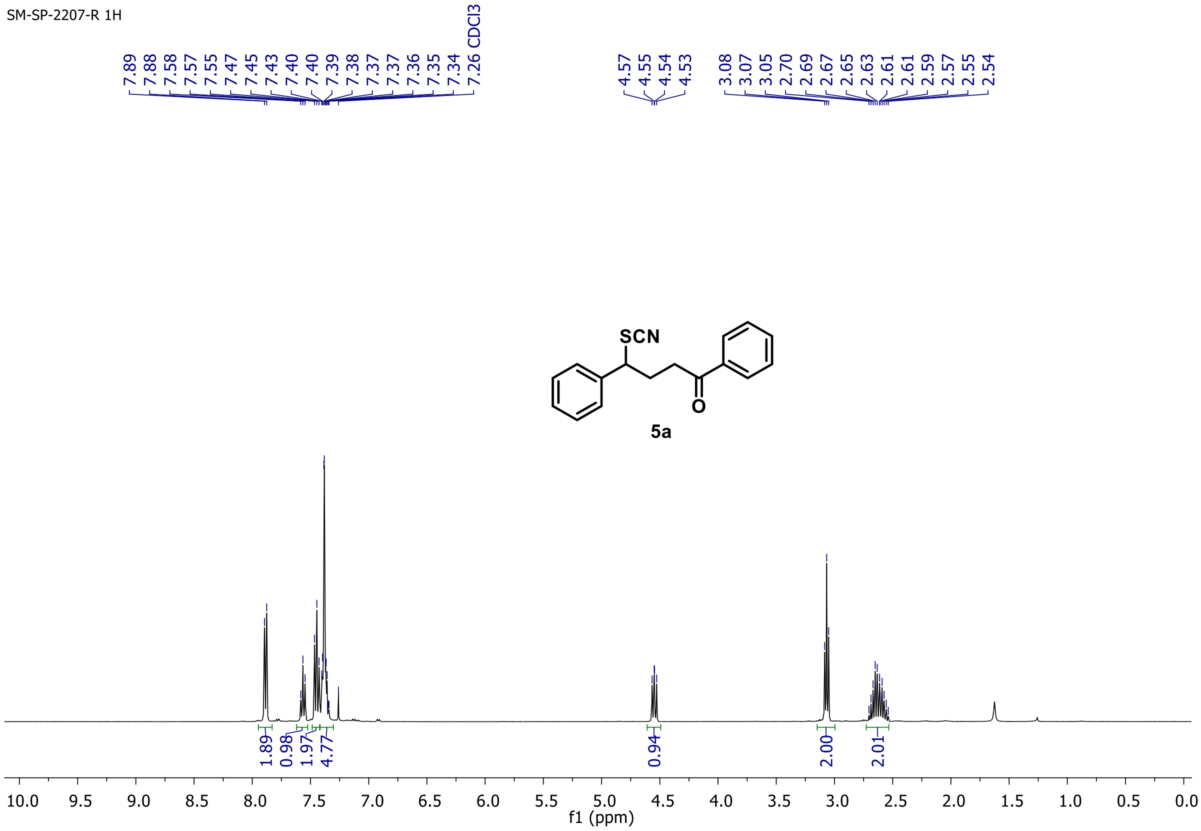


$^{13}C\{^1H\}$ NMR of **5a** (101 MHz, $CDCl_3$):

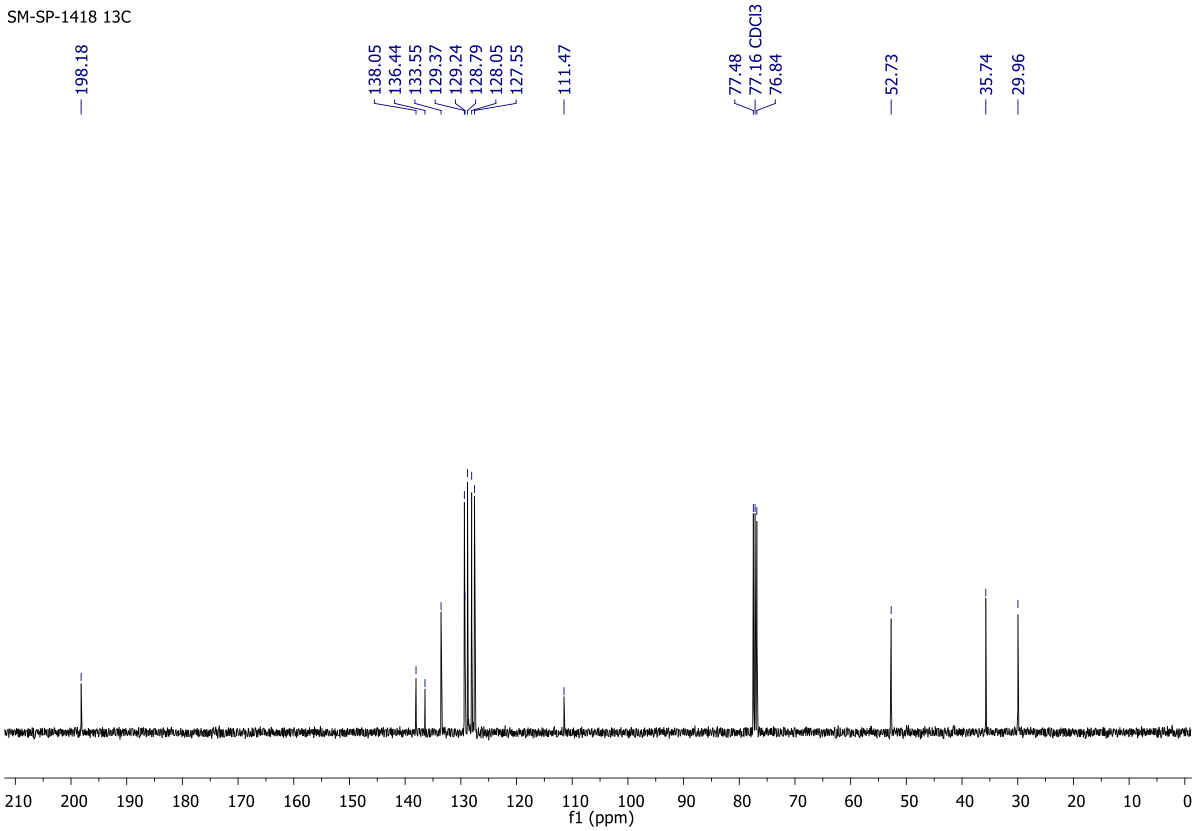

<sup></sup>

$^{1}$H NMR of **5b** (400 MHz, $CDCl_3$):

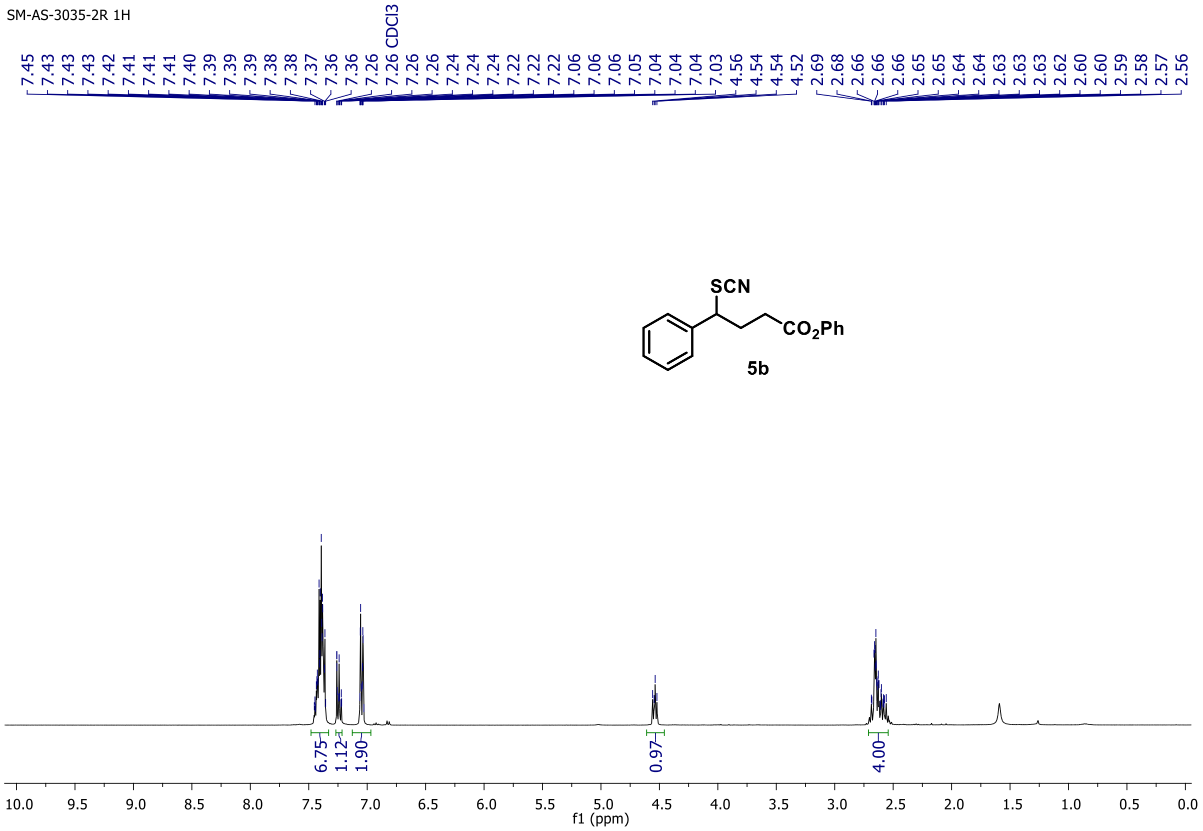


$^{13}$C{$^{1}$H} NMR of **5b** (101 MHz, $CDCl_3$):

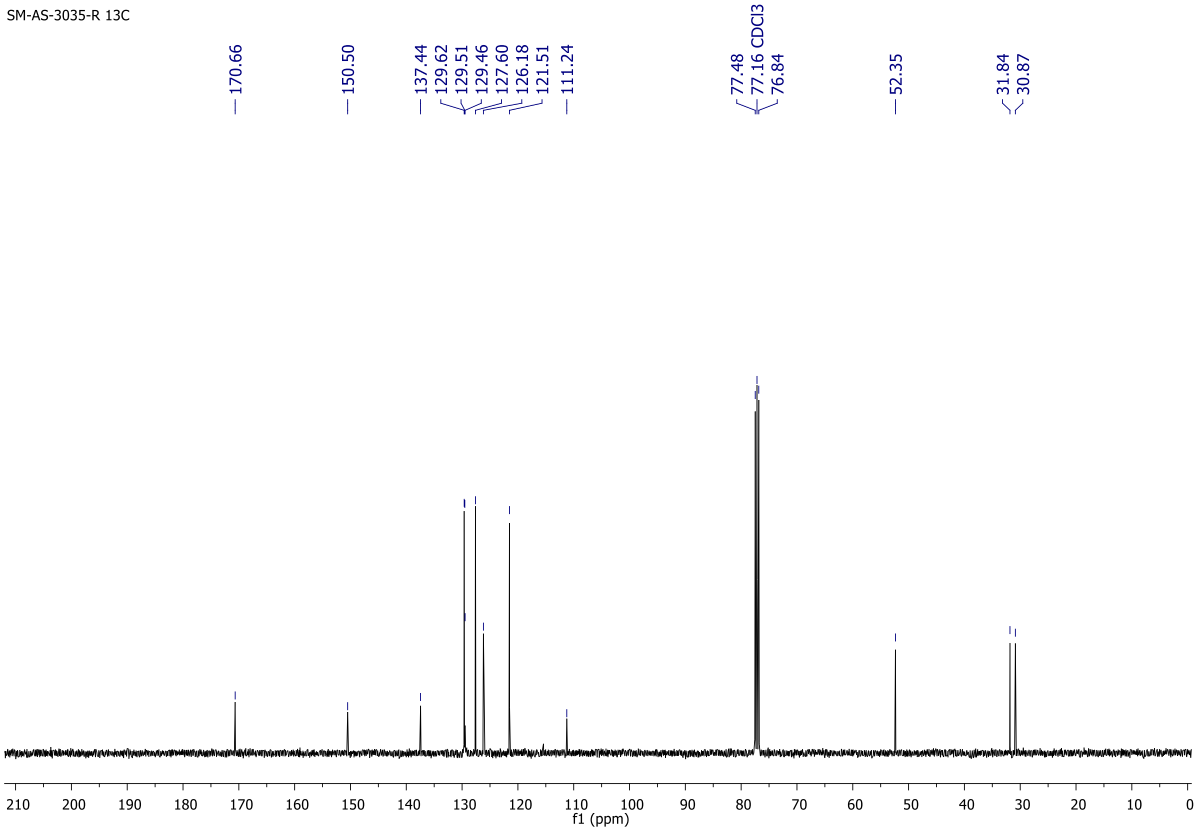

$^{1}$H NMR of **5c** (400 MHz, CDCl$_3$):

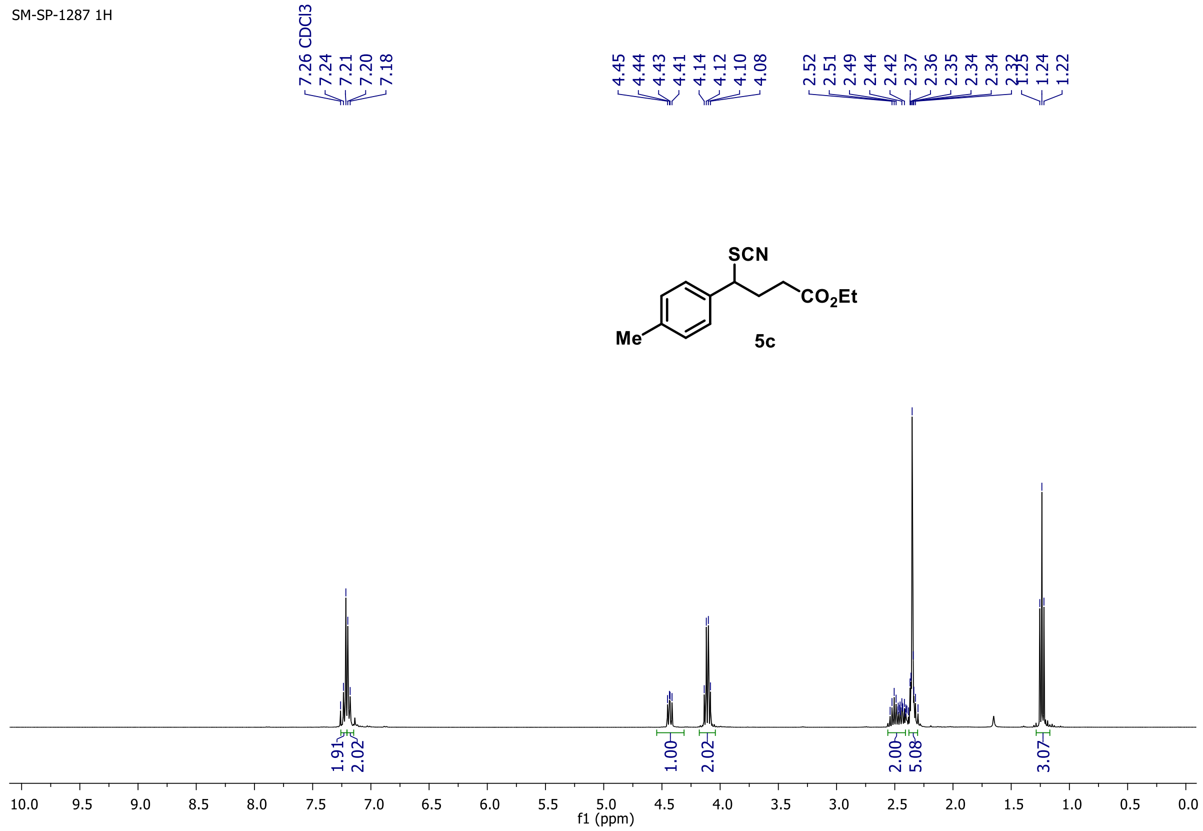


$^{13}$C{$^{1}$H} NMR of **5c** (101 MHz, CDCl$_3$):

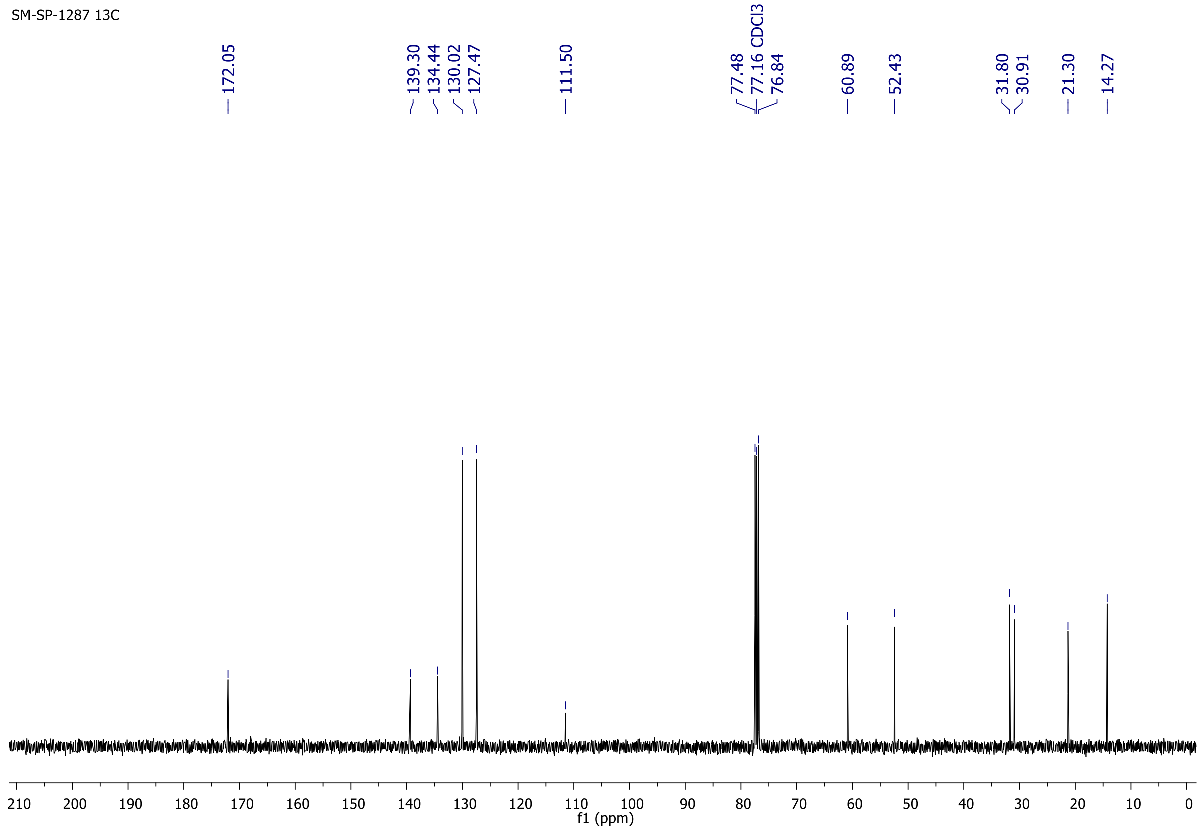

$^{1}H$ NMR of **5d** (400 MHz, $CDCl_3$):

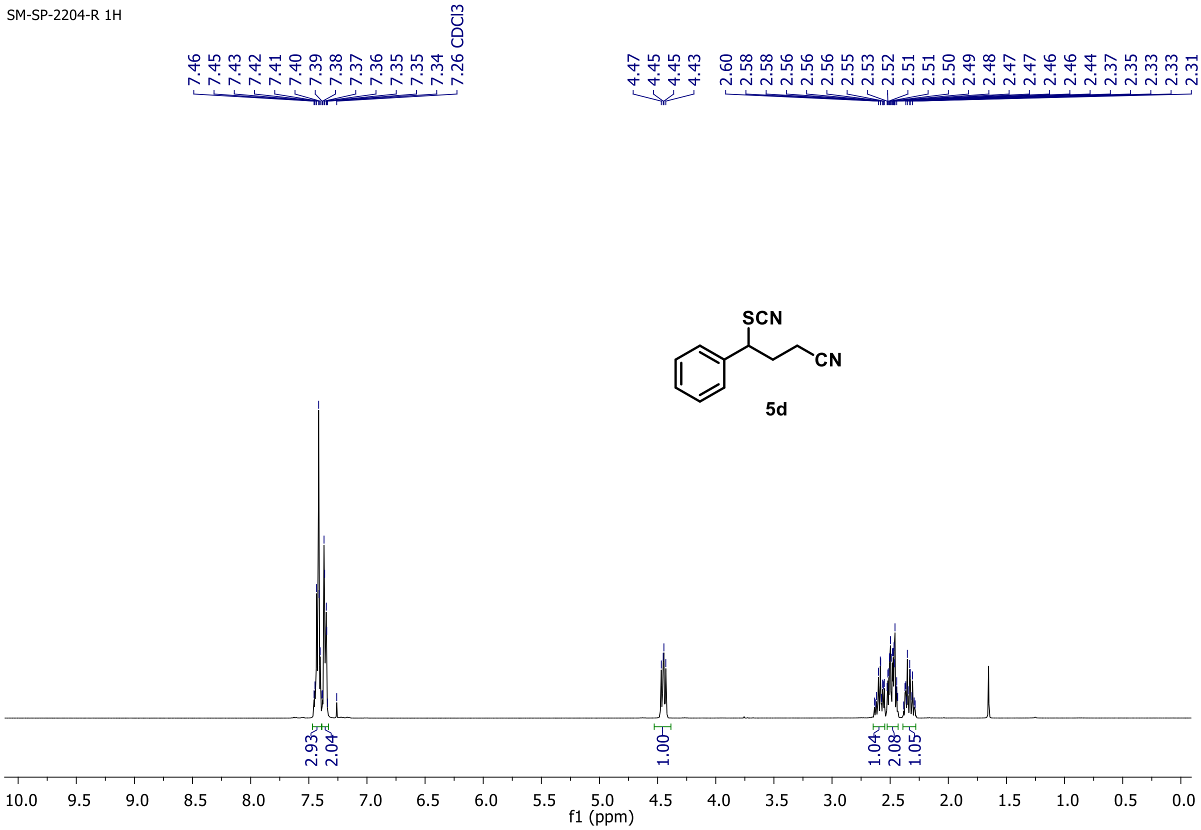


$^{13}C\{^{1}H\}$ NMR of **5d** (101 MHz, $CDCl_3$):

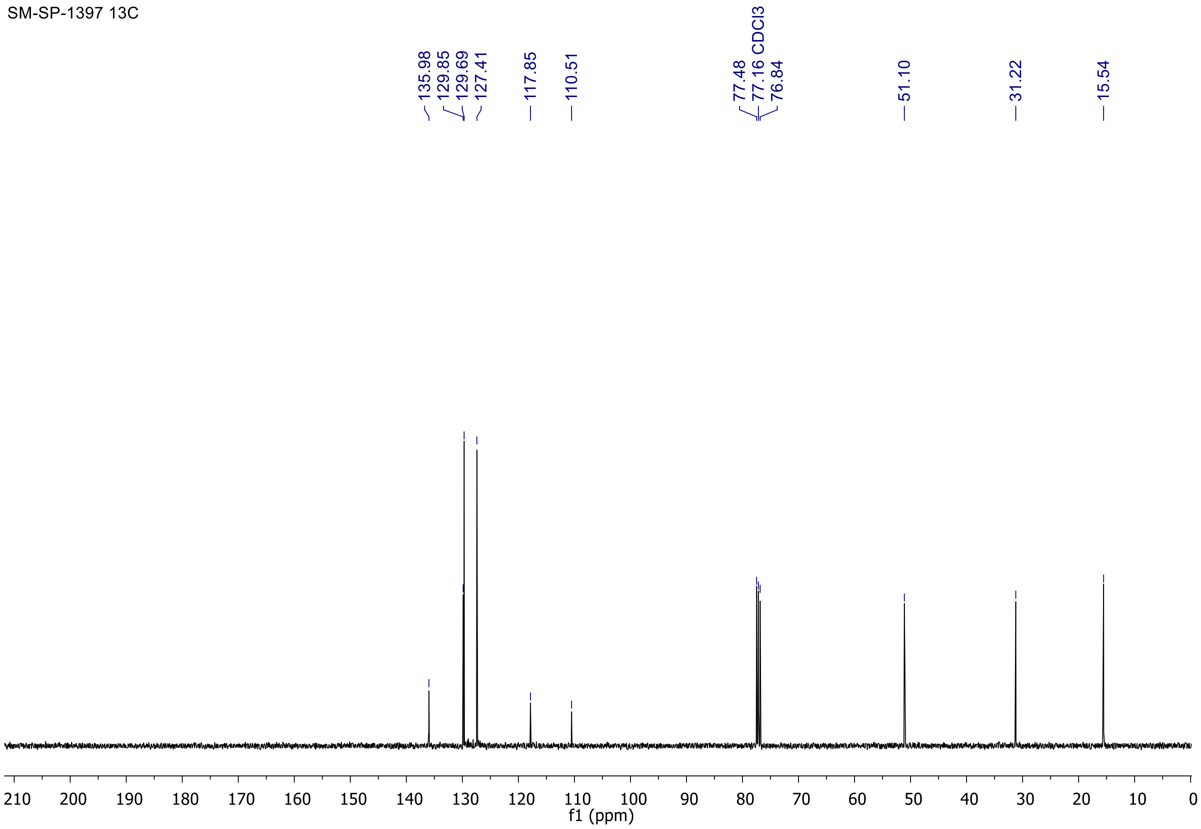

$^{1}H$ NMR of **5e** (400 MHz, $CDCl_3$):

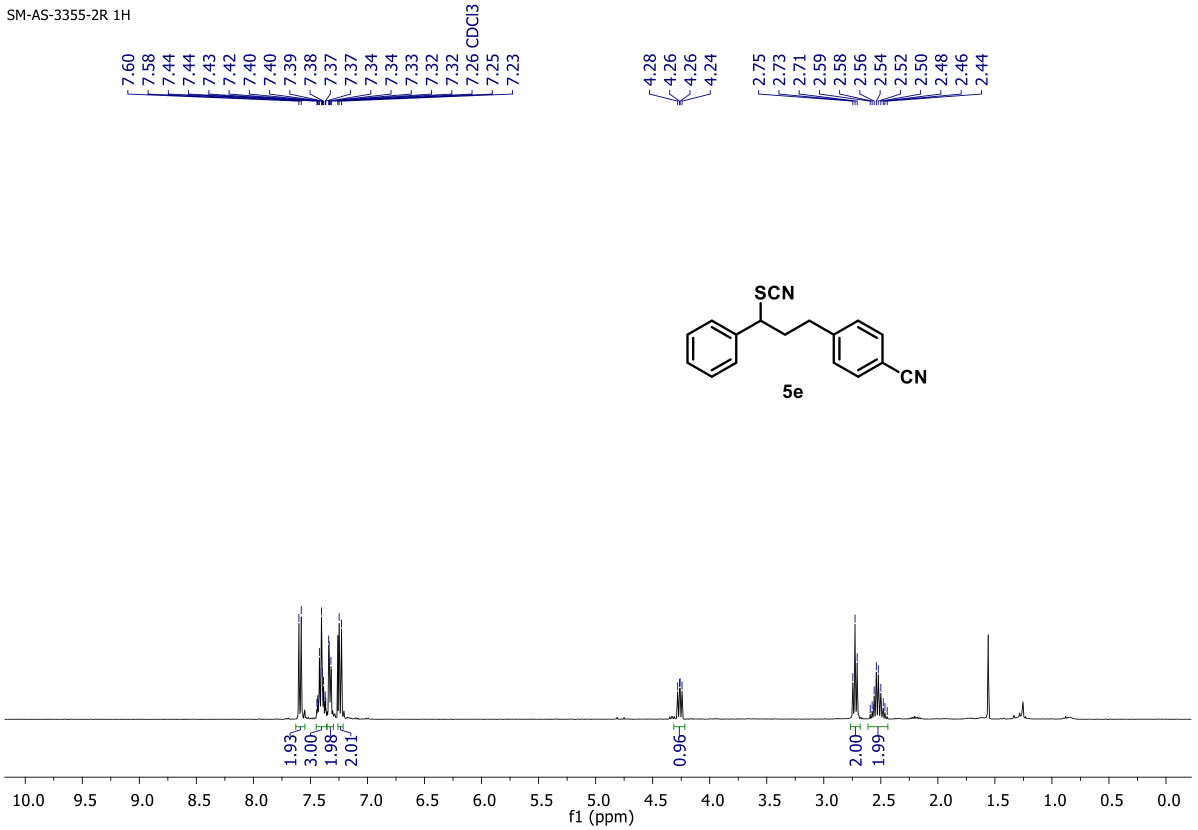


**5e**

$^{13}C\{^{1}H\}$ NMR of **5e** (101 MHz, $CDCl_3$):

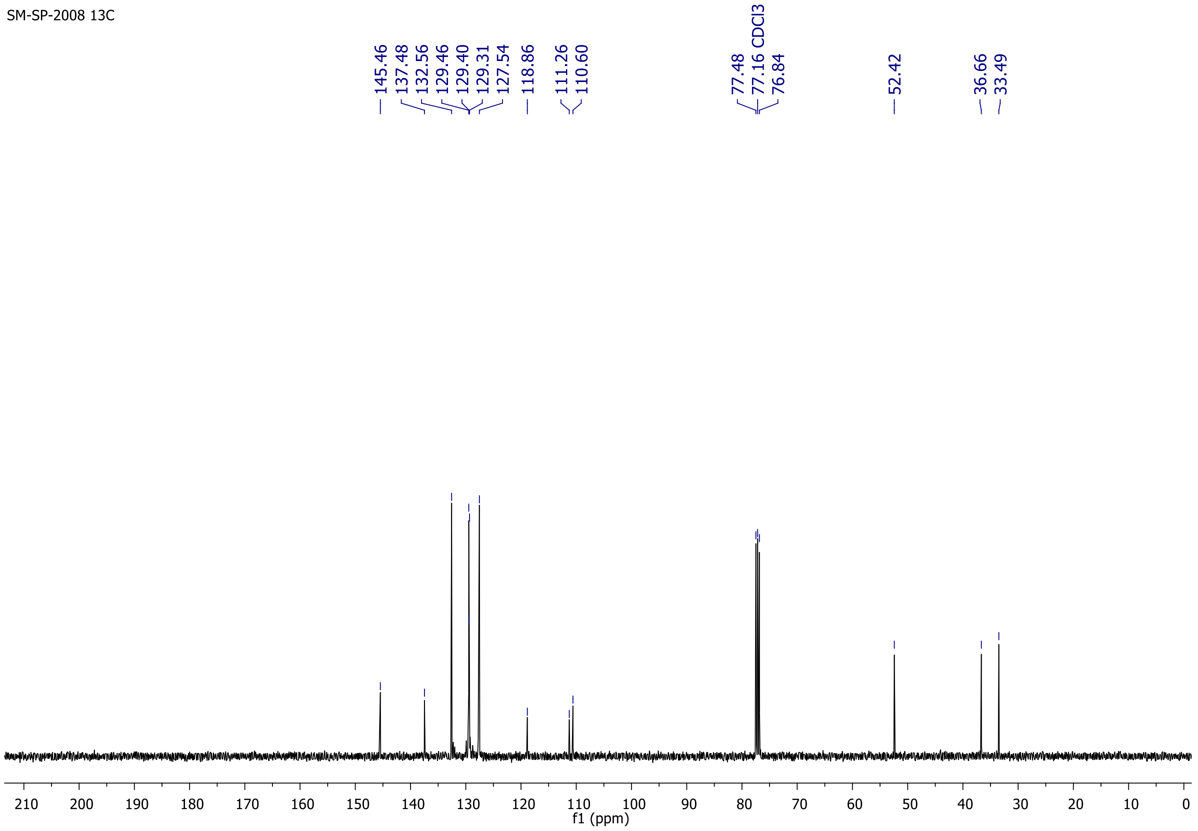

$^{1}H$ NMR of **5f** (400 MHz, $CDCl_3$):

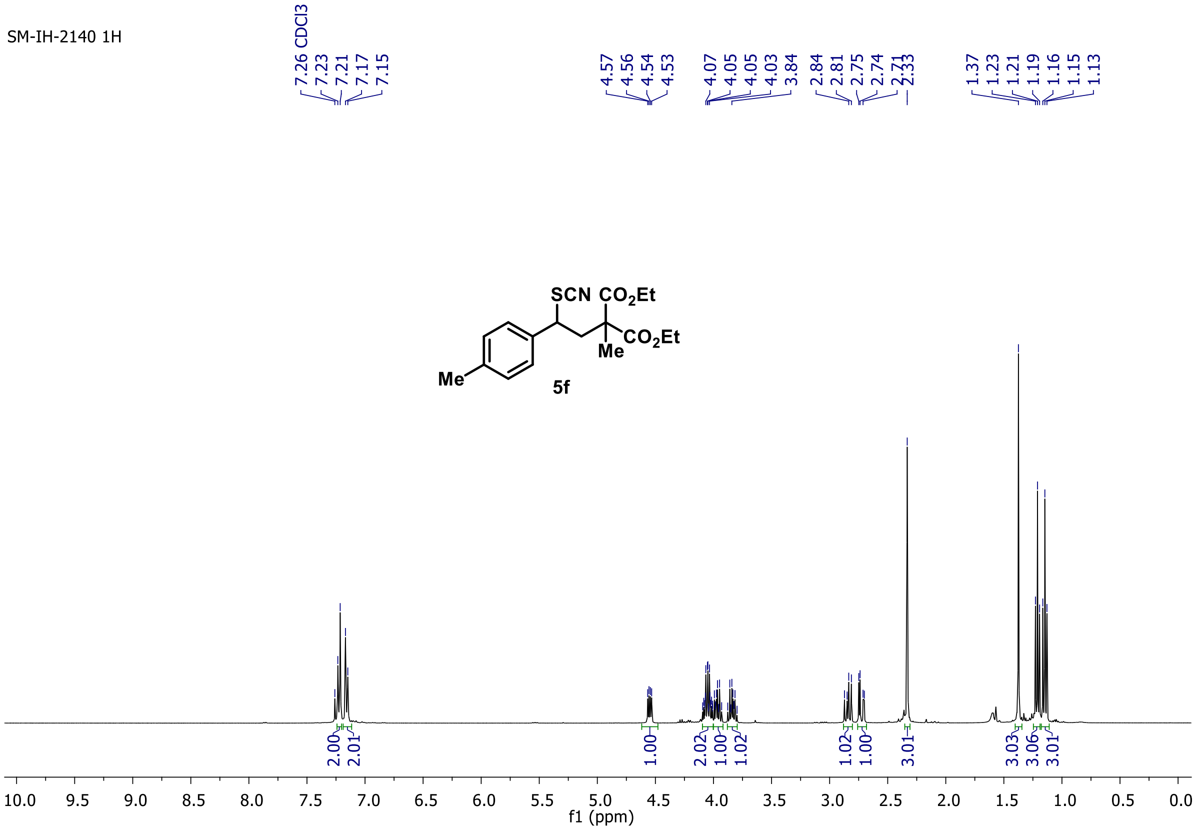


$^{13}C\{^{1}H\}$ NMR of **5f** (101 MHz, $CDCl_3$):

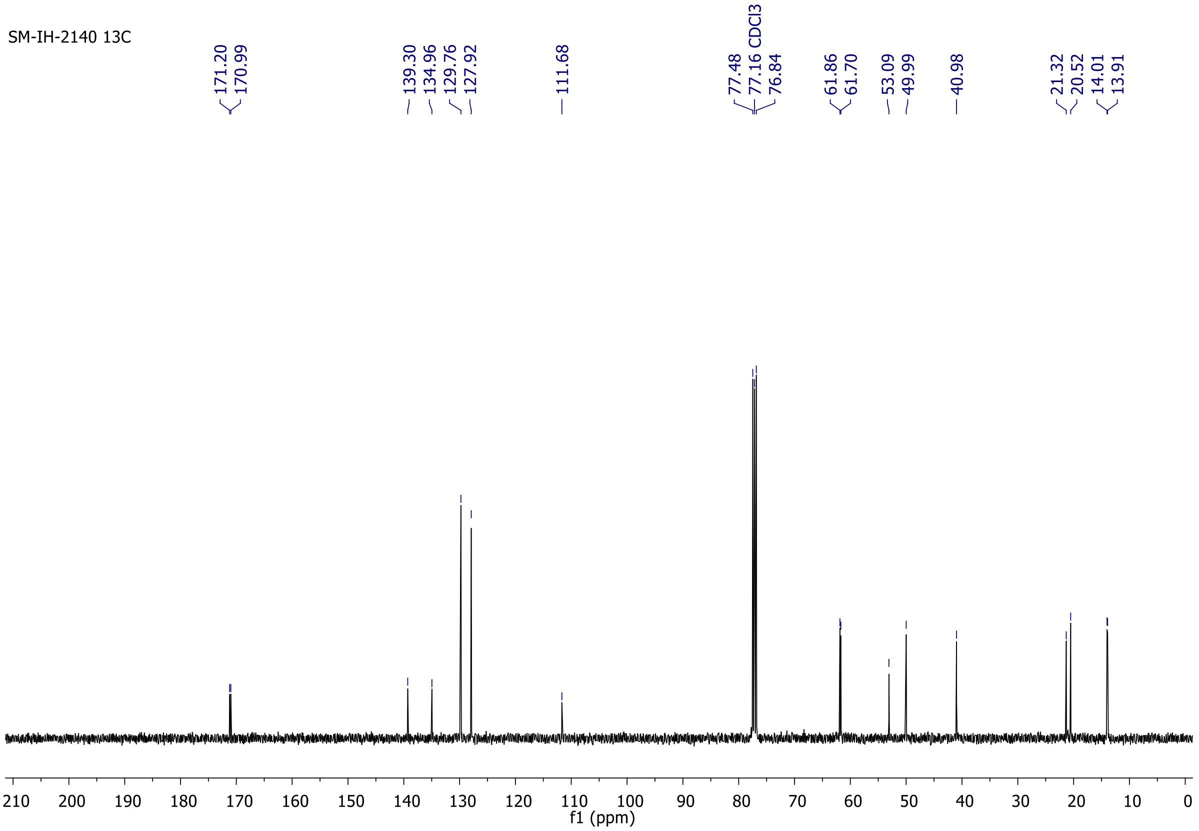

$^{1}$H NMR of **5g** (400 MHz, $CDCl_3$):

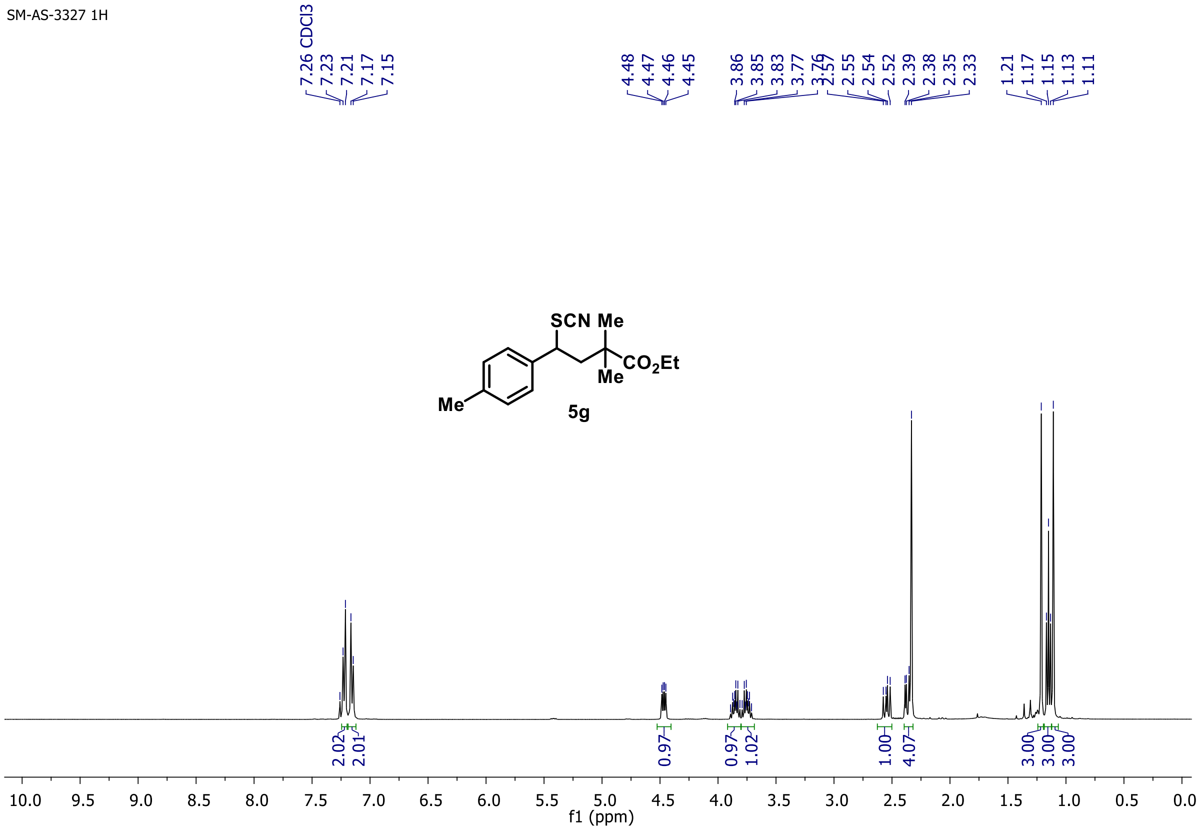


$^{13}$C{$^{1}$H} NMR of **5g** (101 MHz, $CDCl_3$):

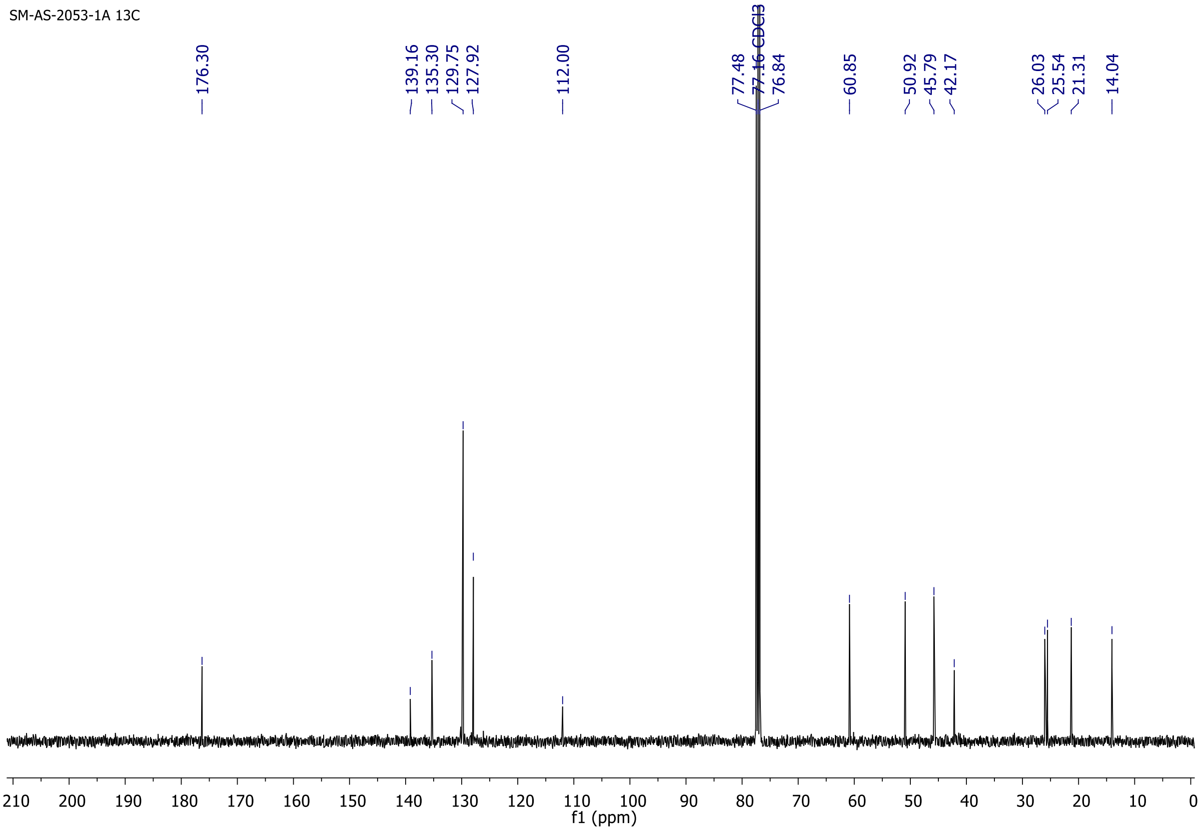

$^{1}H$ NMR of **5h** (400 MHz, $CDCl_3$):

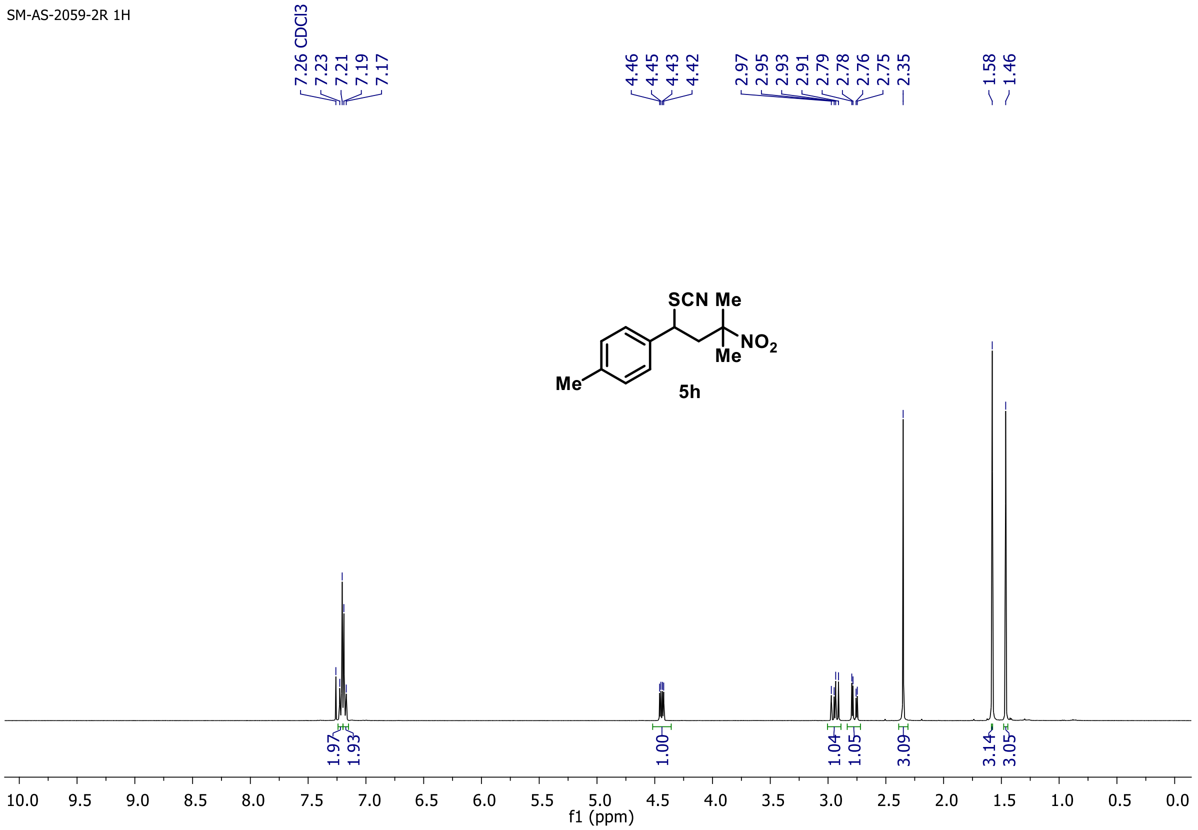


$^{13}C\{^{1}H\}$ NMR of **5h** (101 MHz, $CDCl_3$):

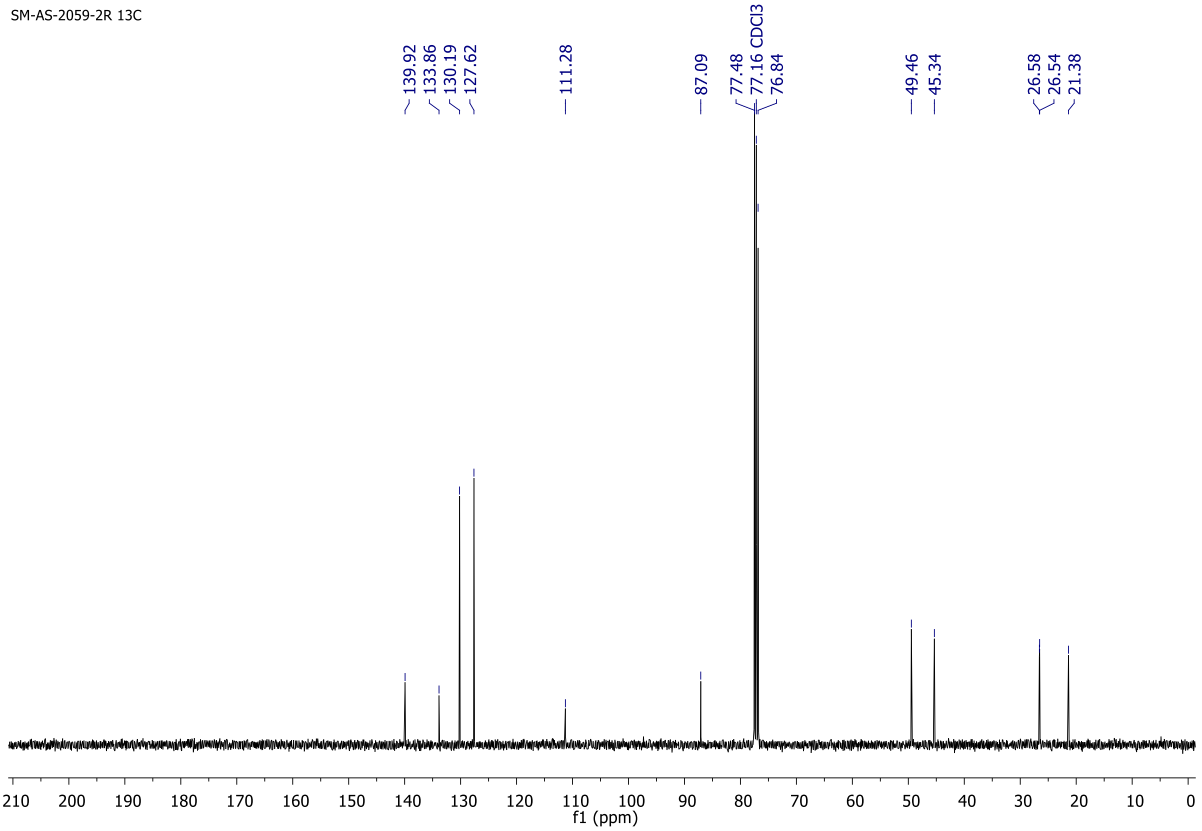

$^{1}H$ NMR of **5i** (400 MHz, $CDCl_3$):

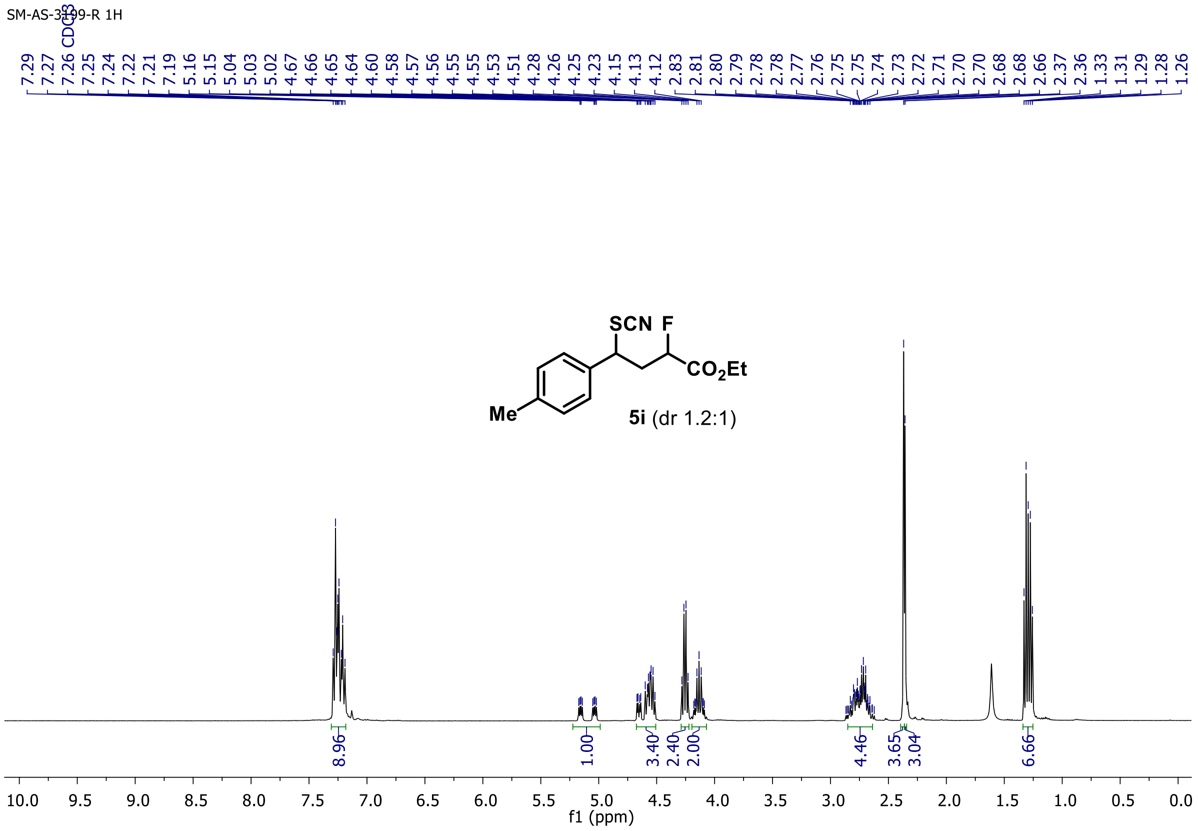


$^{13}C\{^{1}H\}$ NMR of **5i** (101 MHz, $CDCl_3$):

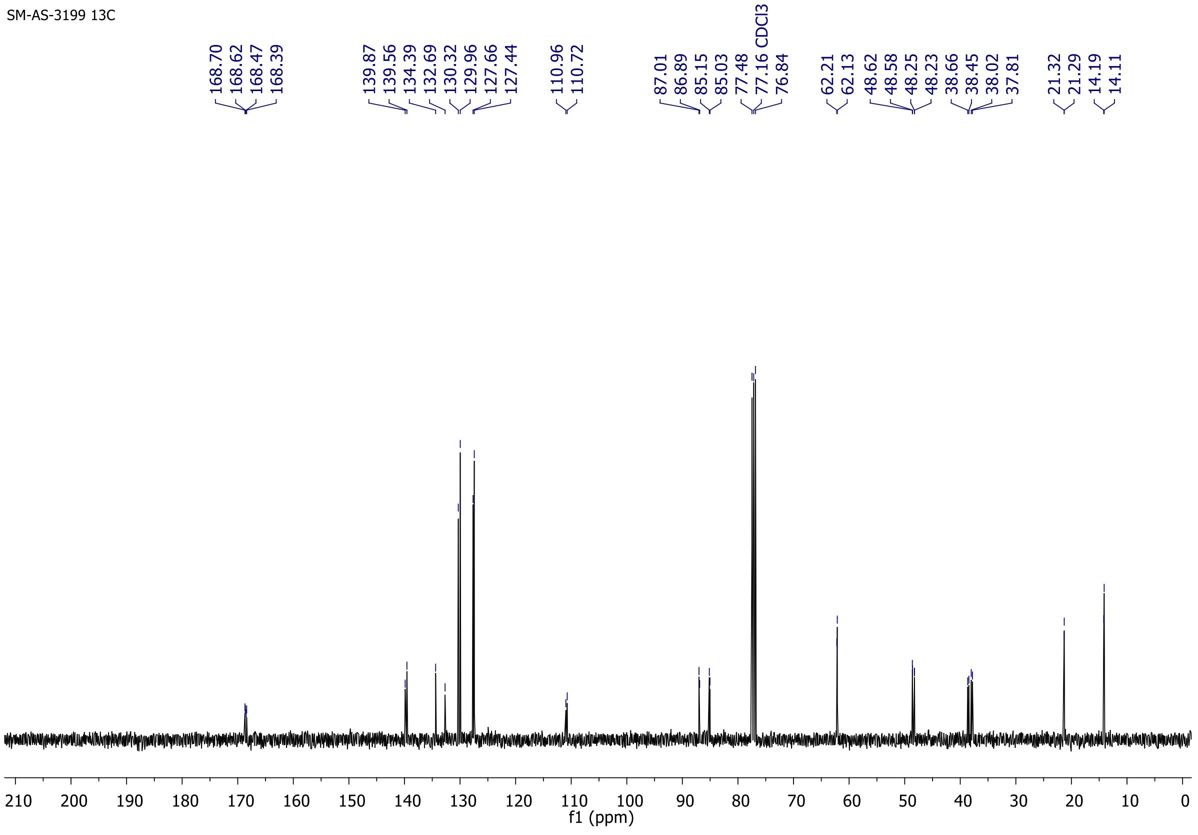

$^{19}$F NMR of **5i** (377 MHz, $CDCl_3$):

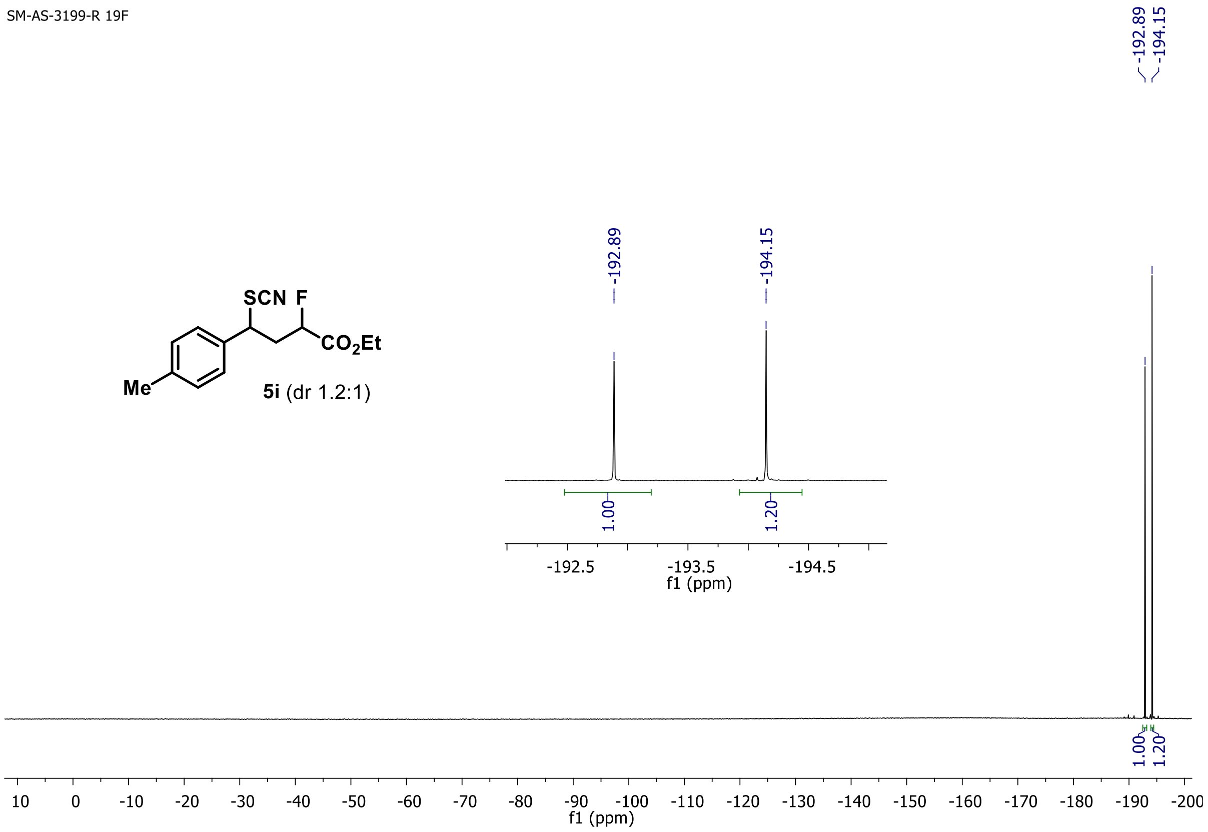

$^{1}H$ NMR of **6a** (400 MHz, $CDCl_3$):

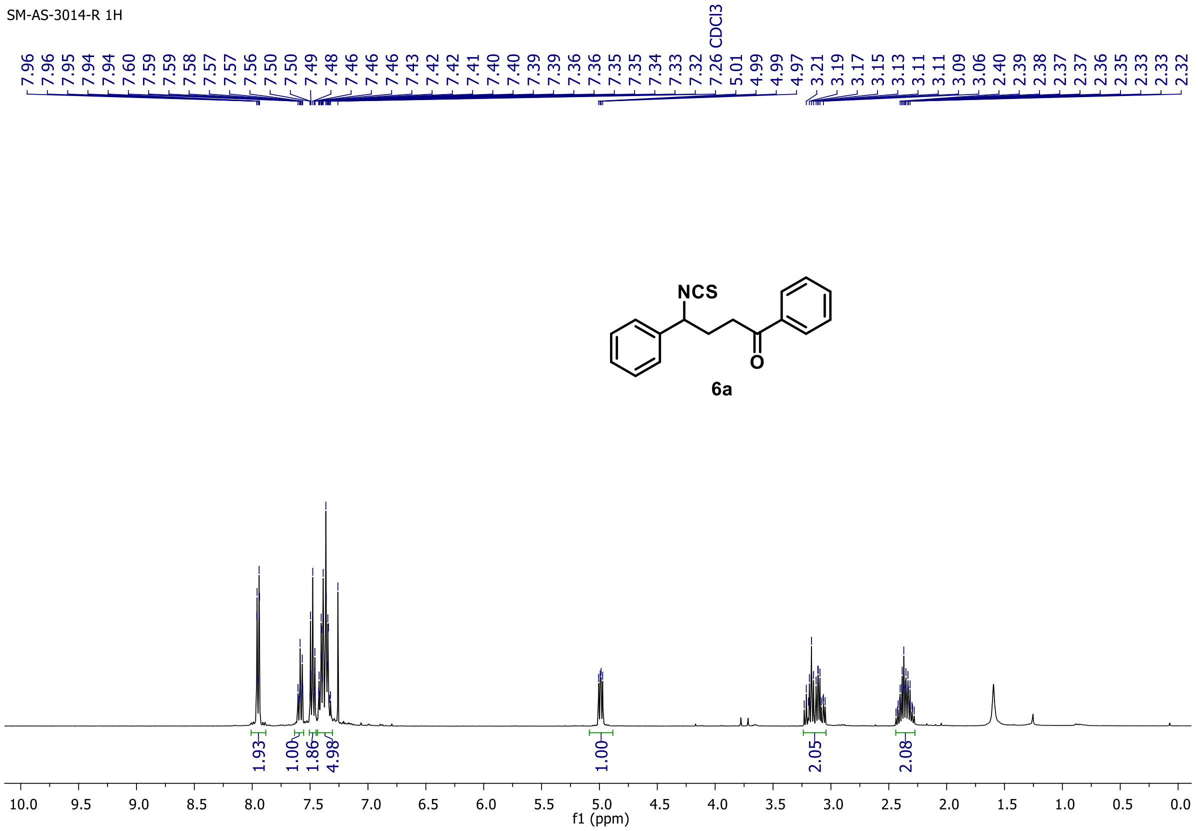


$^{13}C\{^{1}H\}$ NMR of **6a** (101 MHz, $CDCl_3$):

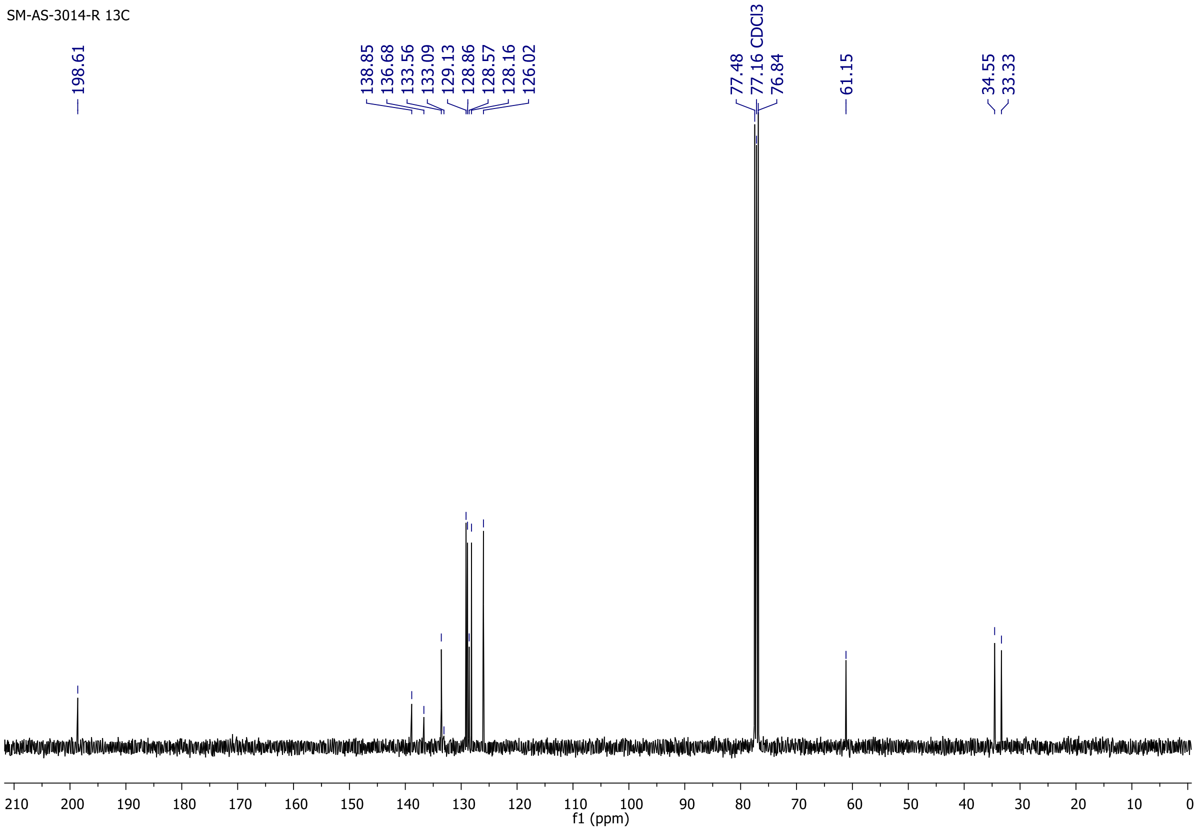

$^{1}H$ NMR of **6b** (400 MHz, $CDCl_3$):

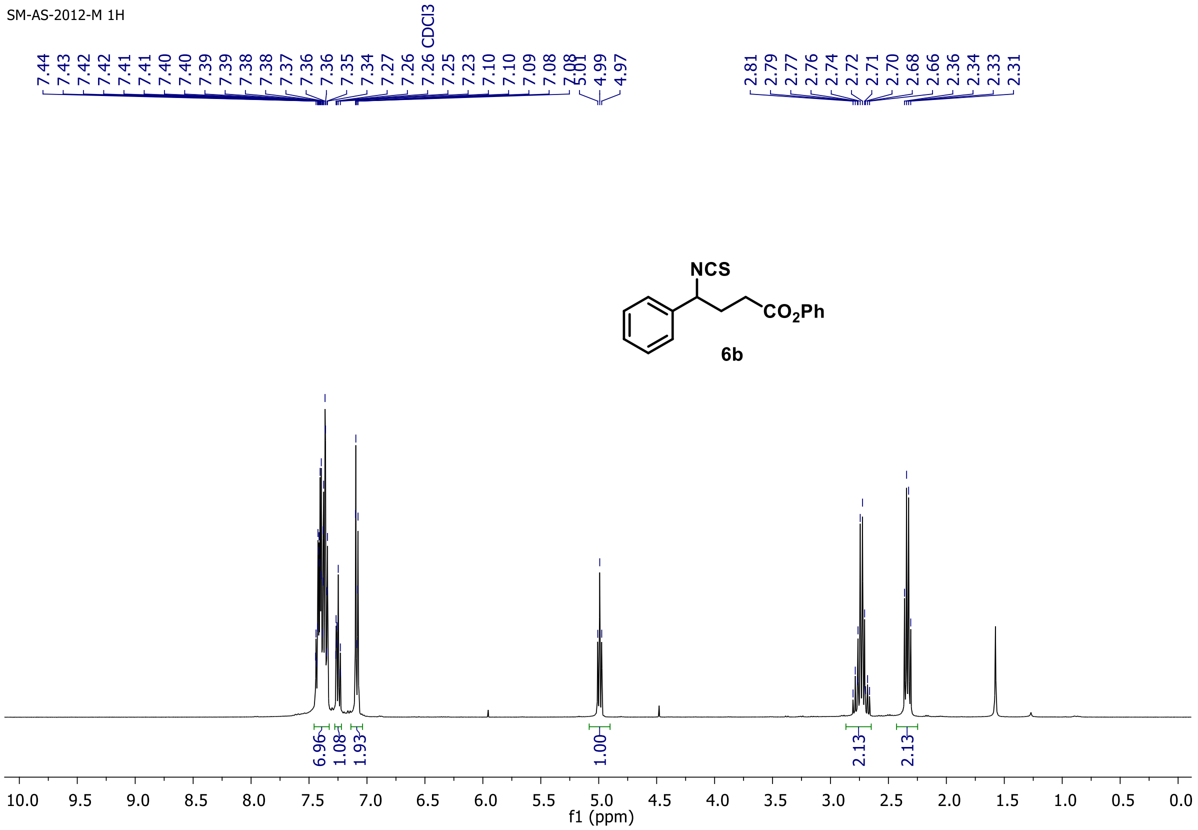


$^{13}C\{^{1}H\}$ NMR of **6b** (101 MHz, $CDCl_3$):

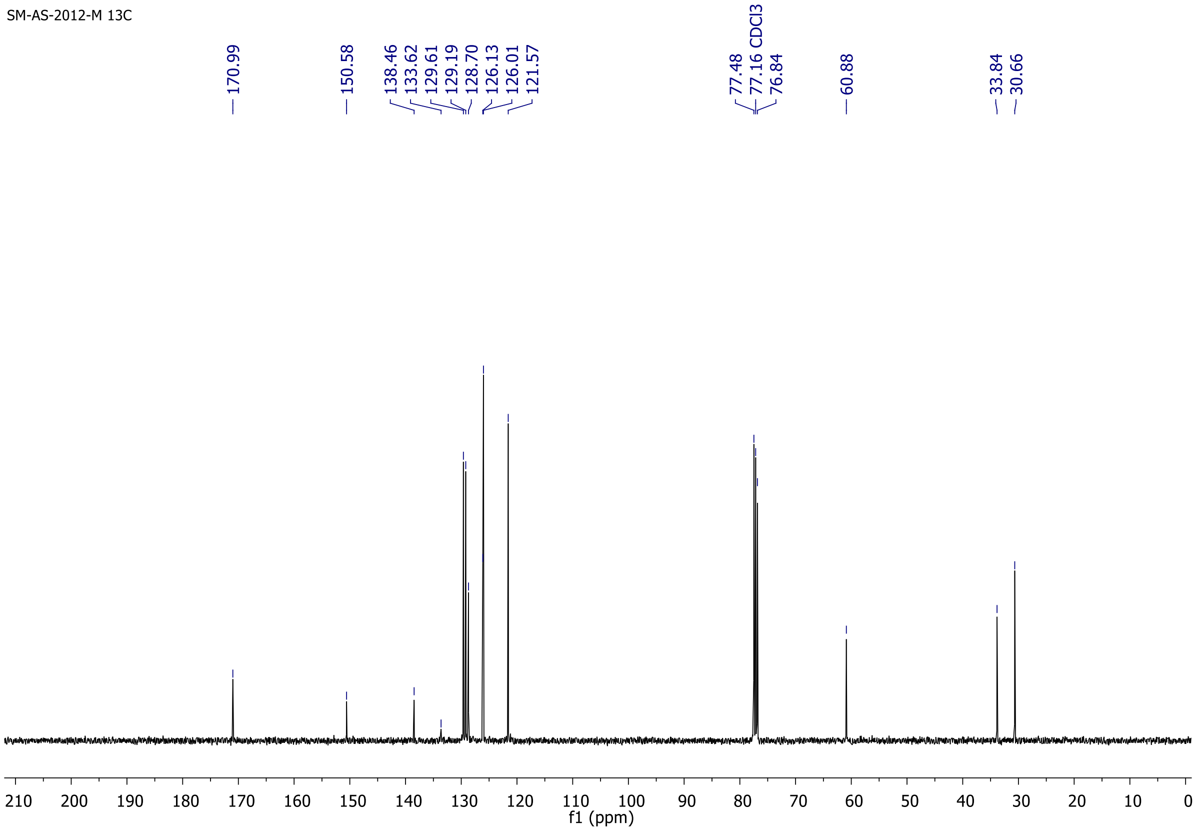

$^{1}$H NMR of **6c** (400 MHz, $CDCl_3$):

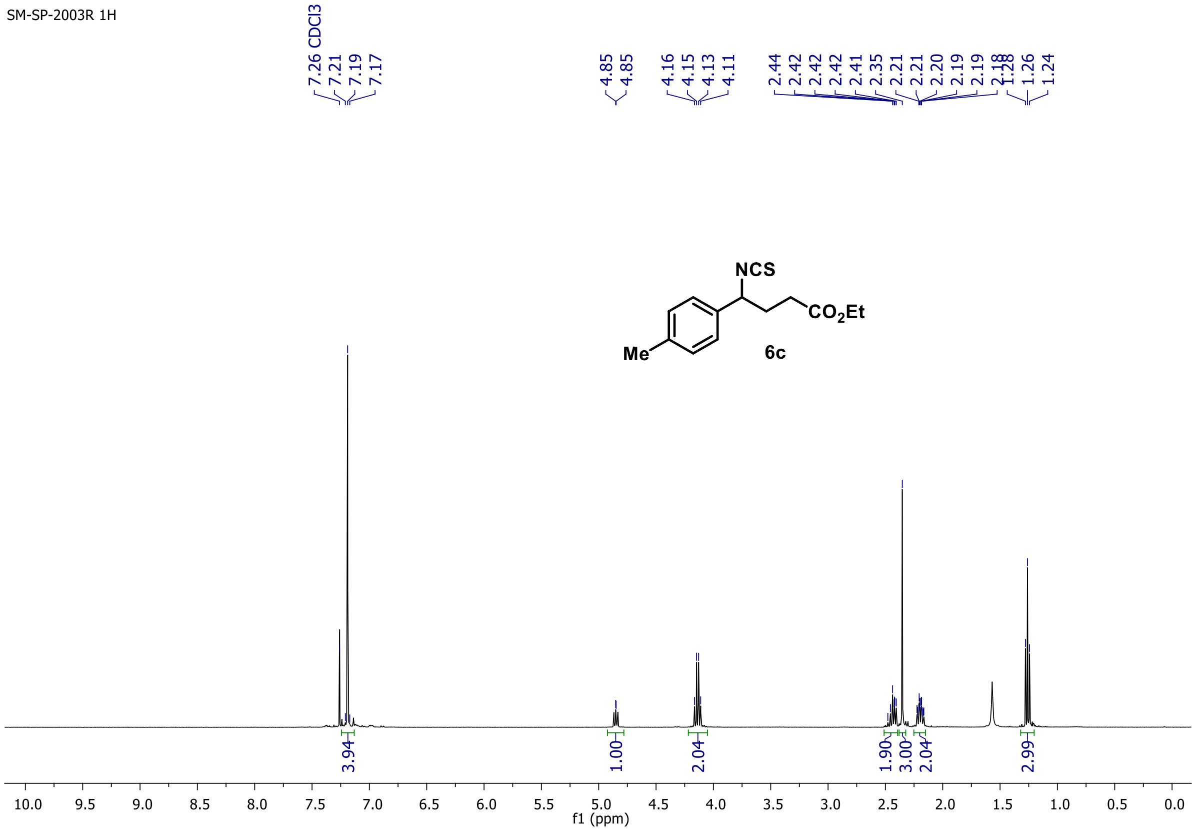


$^{13}$C{$^{1}$H} NMR of **6c** (101 MHz, $CDCl_3$):

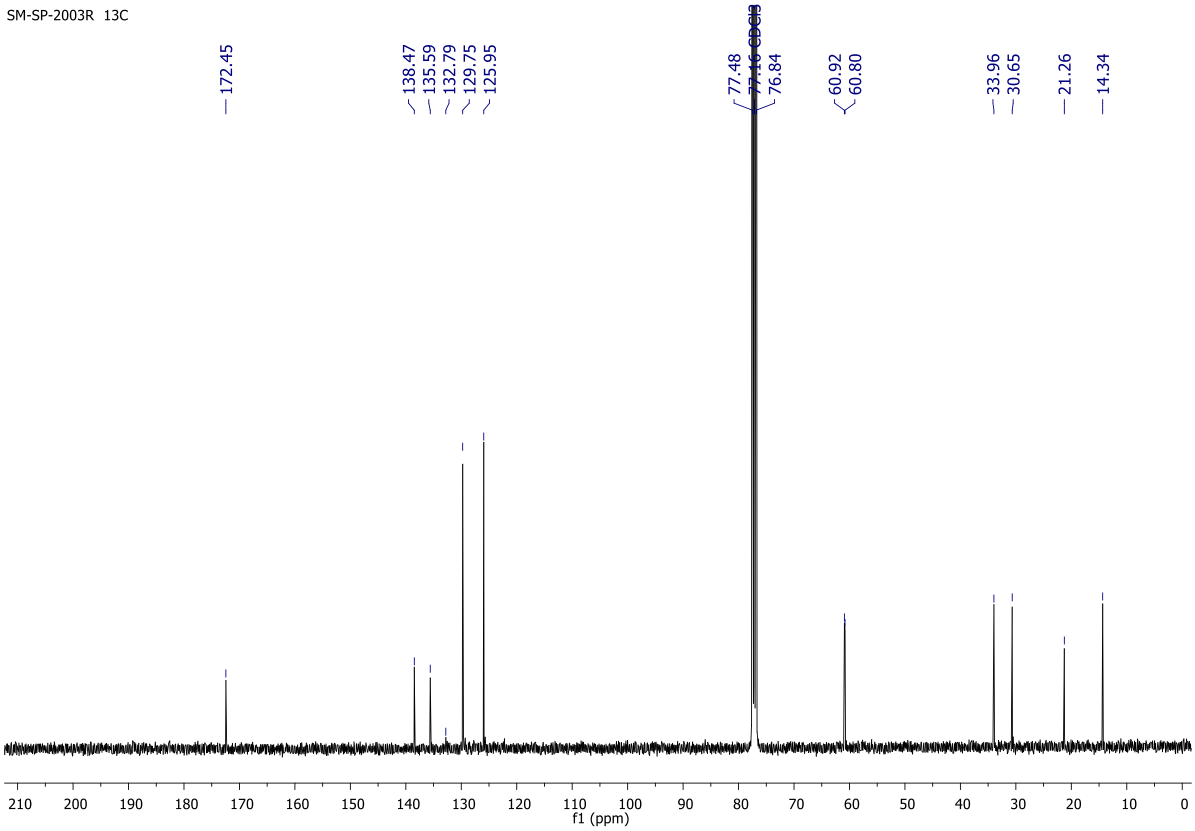

$^{1}H$ NMR of **6d** (400 MHz, $CDCl_3$):

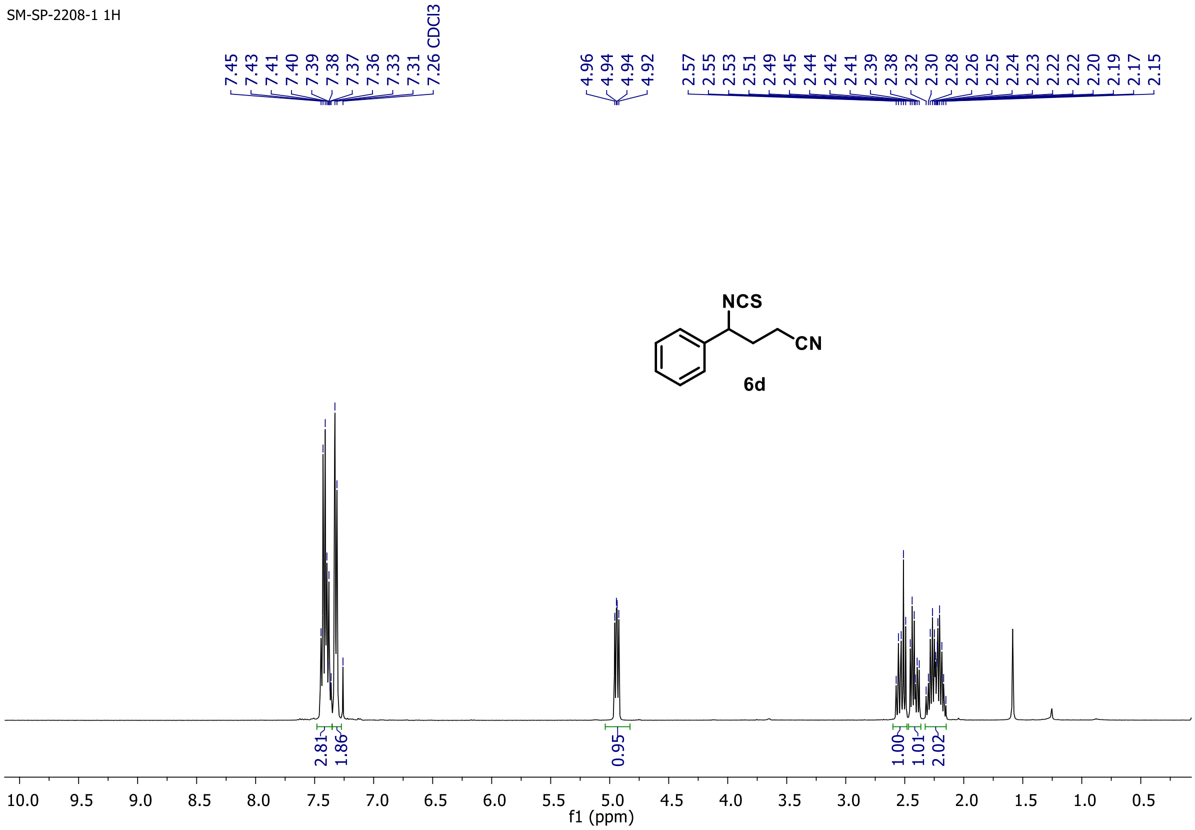


$^{13}C\{^{1}H\}$ NMR of **6d** (101 MHz, $CDCl_3$):

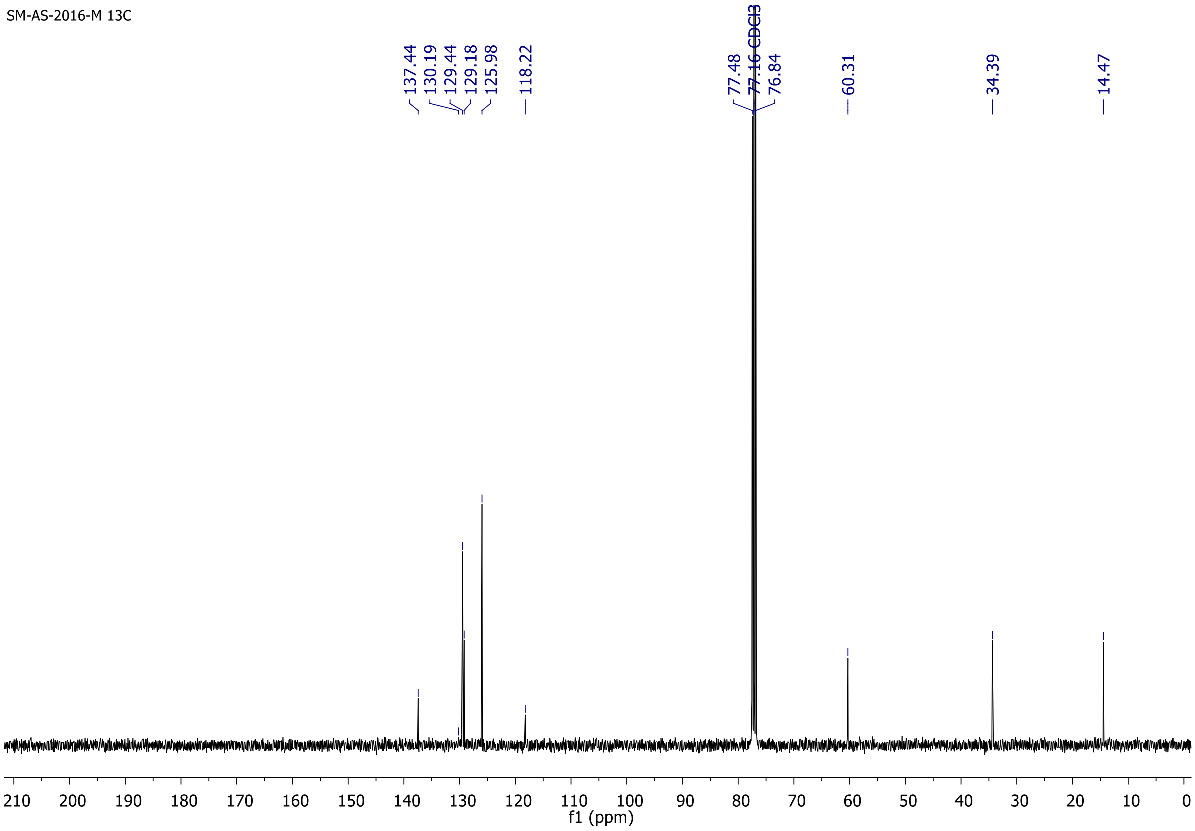

$^{1}H$ NMR of **6e** (400 MHz, $CDCl_3$):

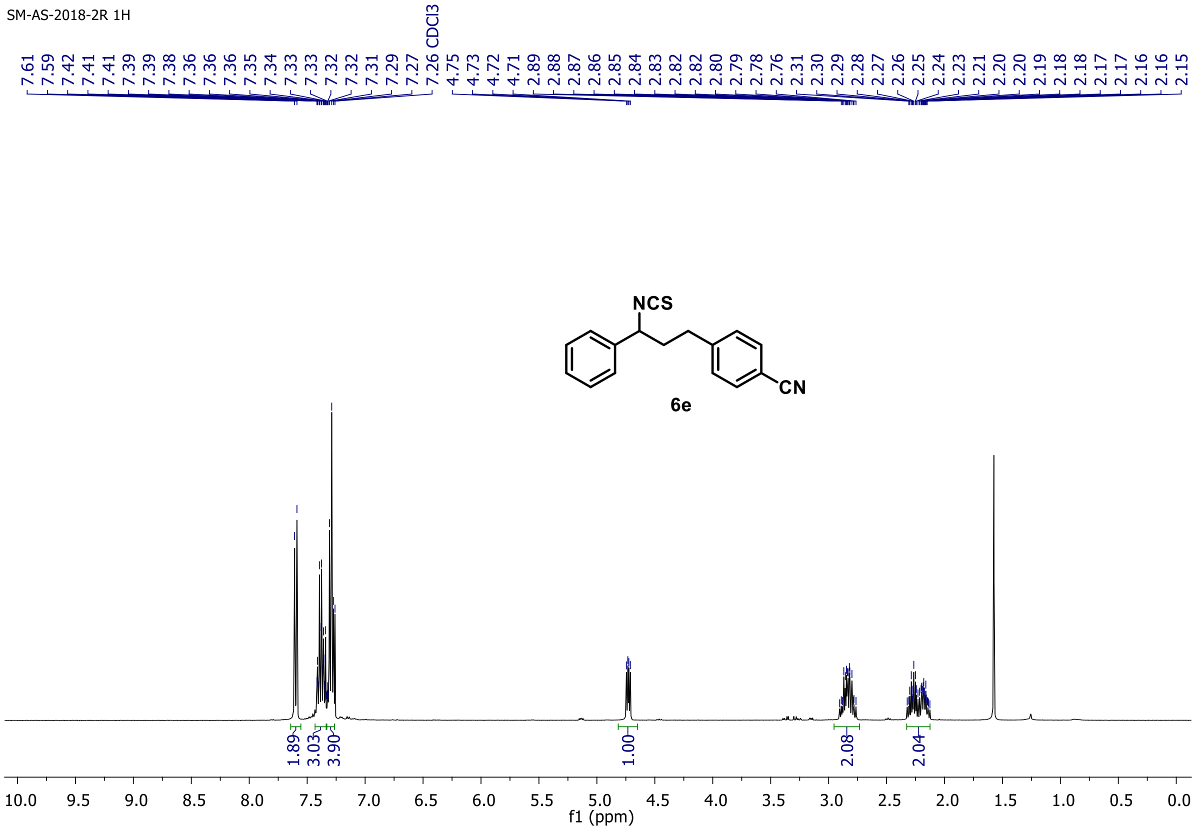


$^{13}C\{^{1}H\}$ NMR of **6e** (101 MHz, $CDCl_3$):

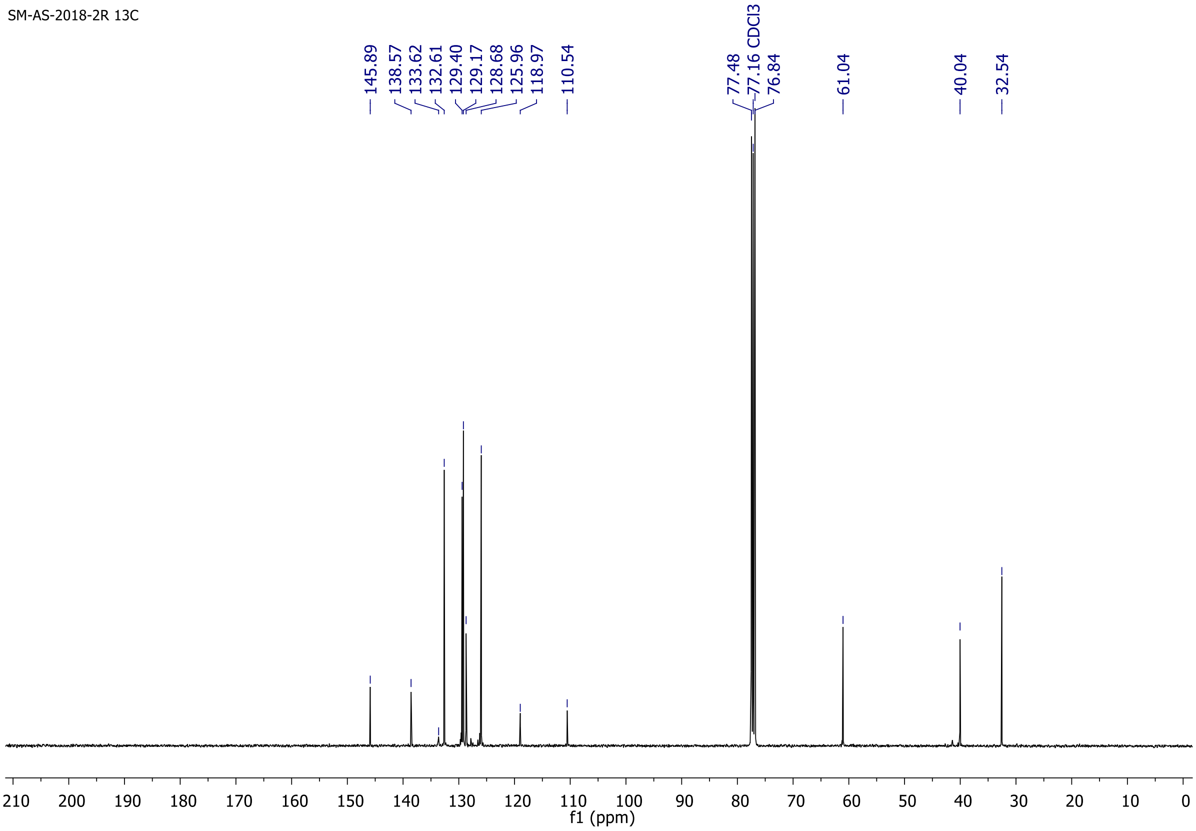

$^{1}H$ NMR of **6f** (400 MHz, $CDCl_3$):

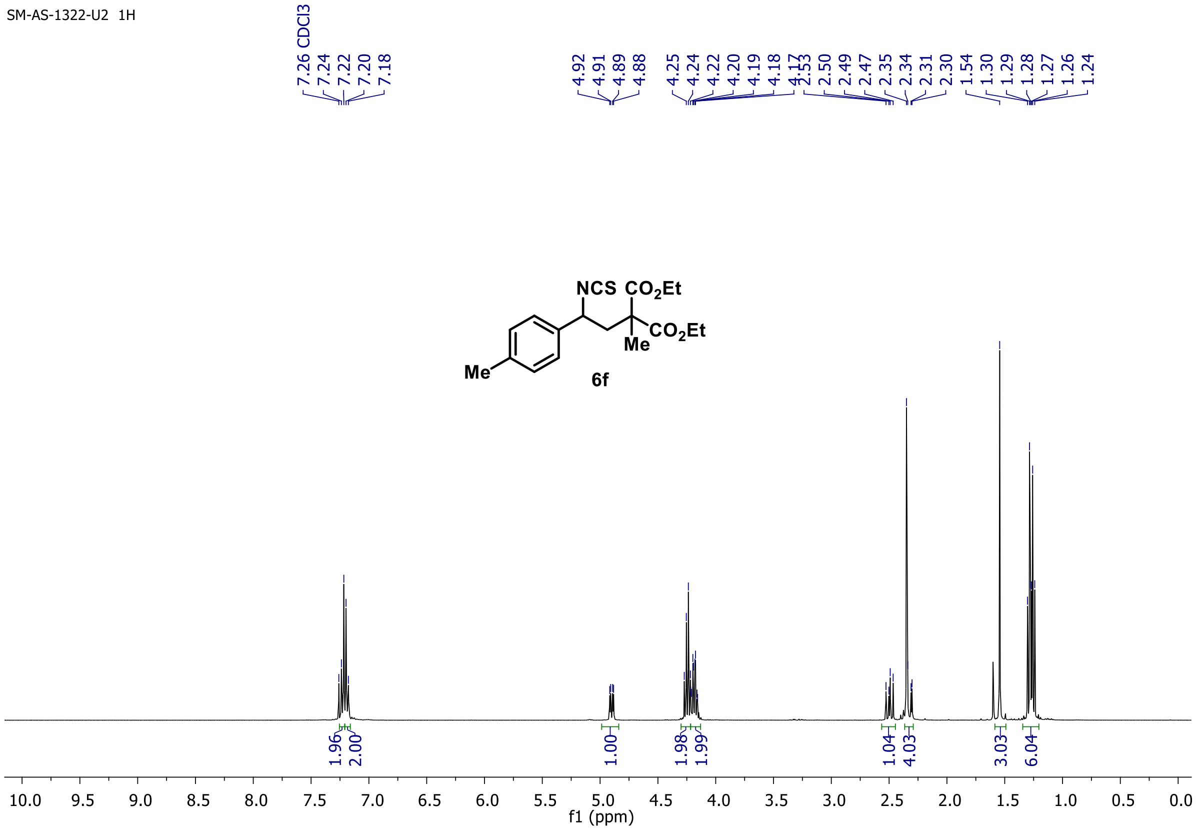


$^{13}C\{^{1}H\}$ NMR of **6f** (101 MHz, $CDCl_3$):

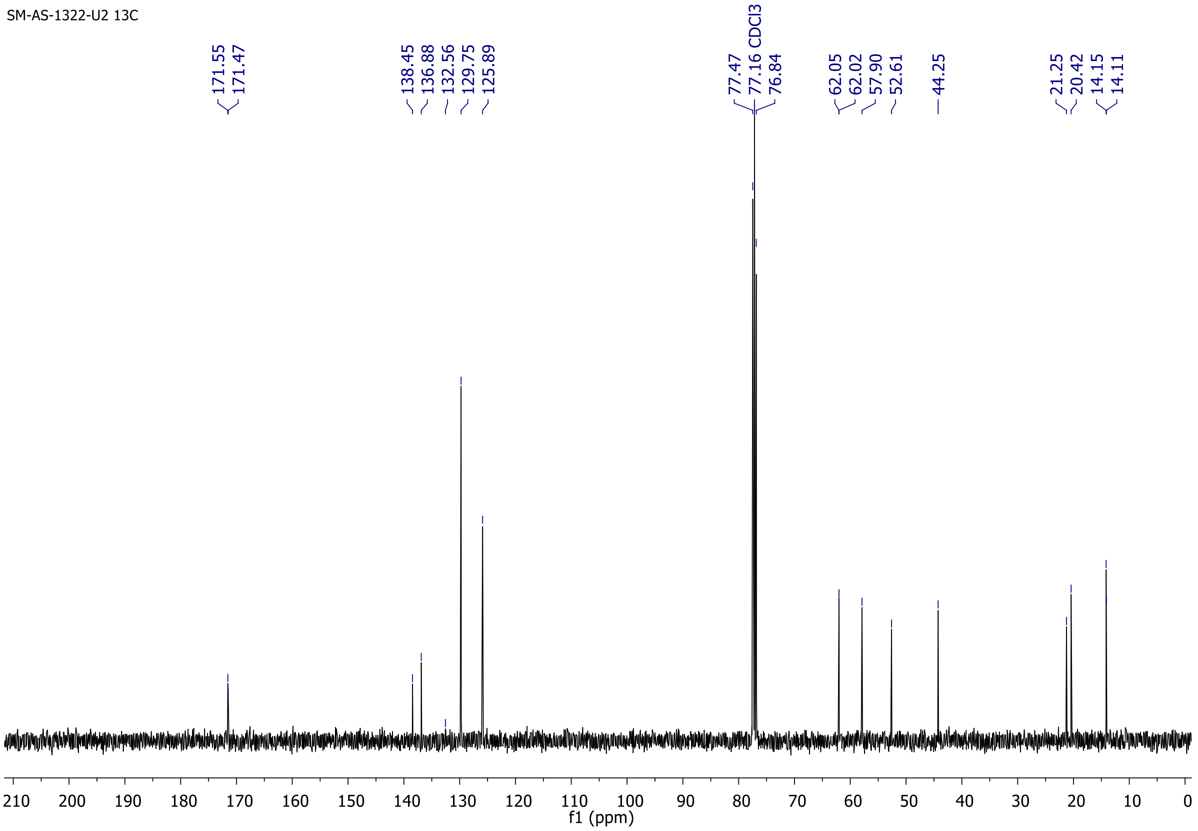

$^{1}H$ NMR of **6g** (400 MHz, $CDCl_3$):

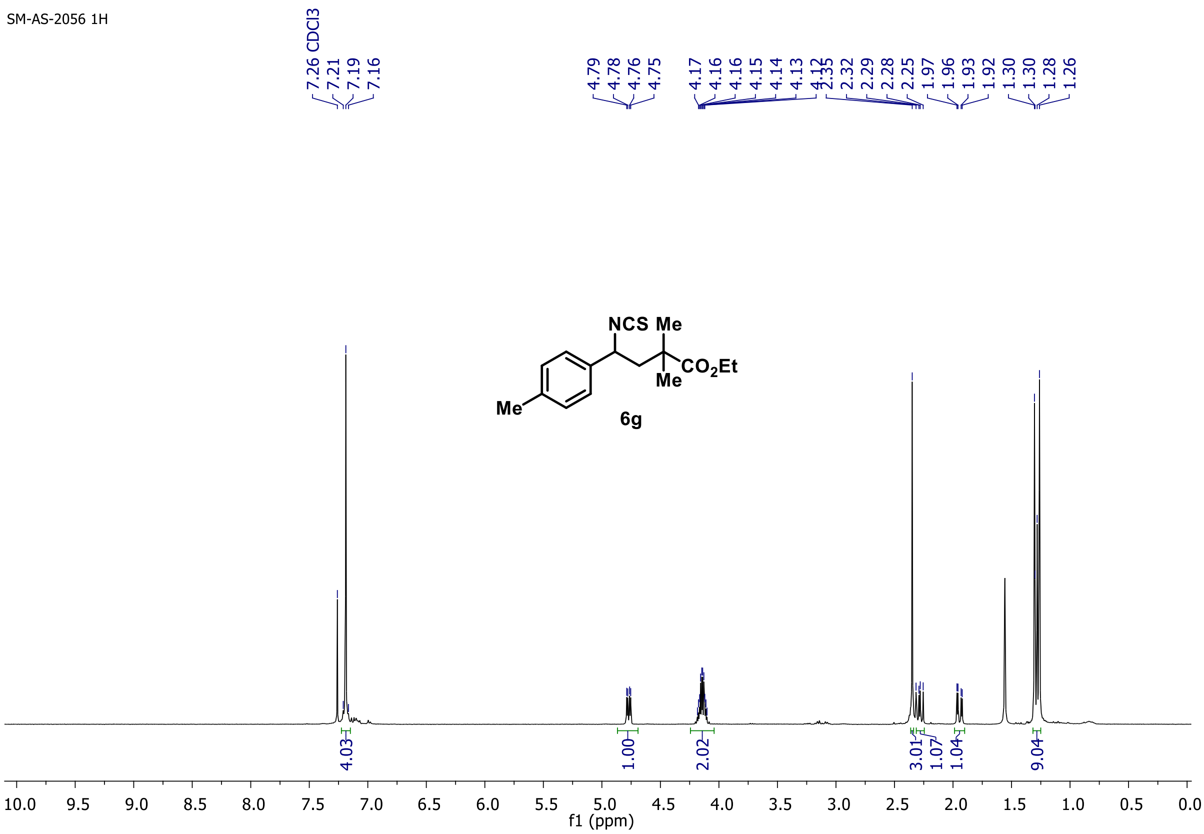


$^{13}C\{^{1}H\}$ NMR of **6g** (101 MHz, $CDCl_3$):

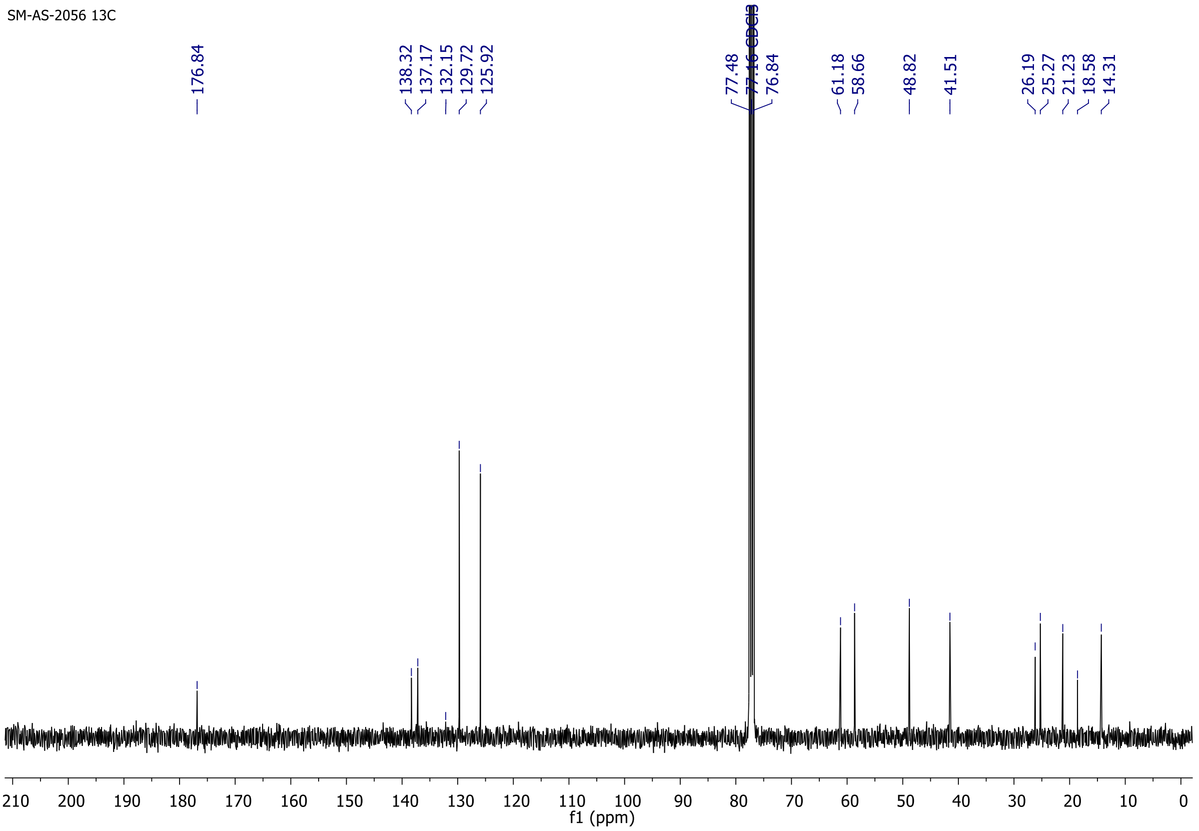

$^{1}$H NMR of **6h** (400 MHz, $CDCl_3$):

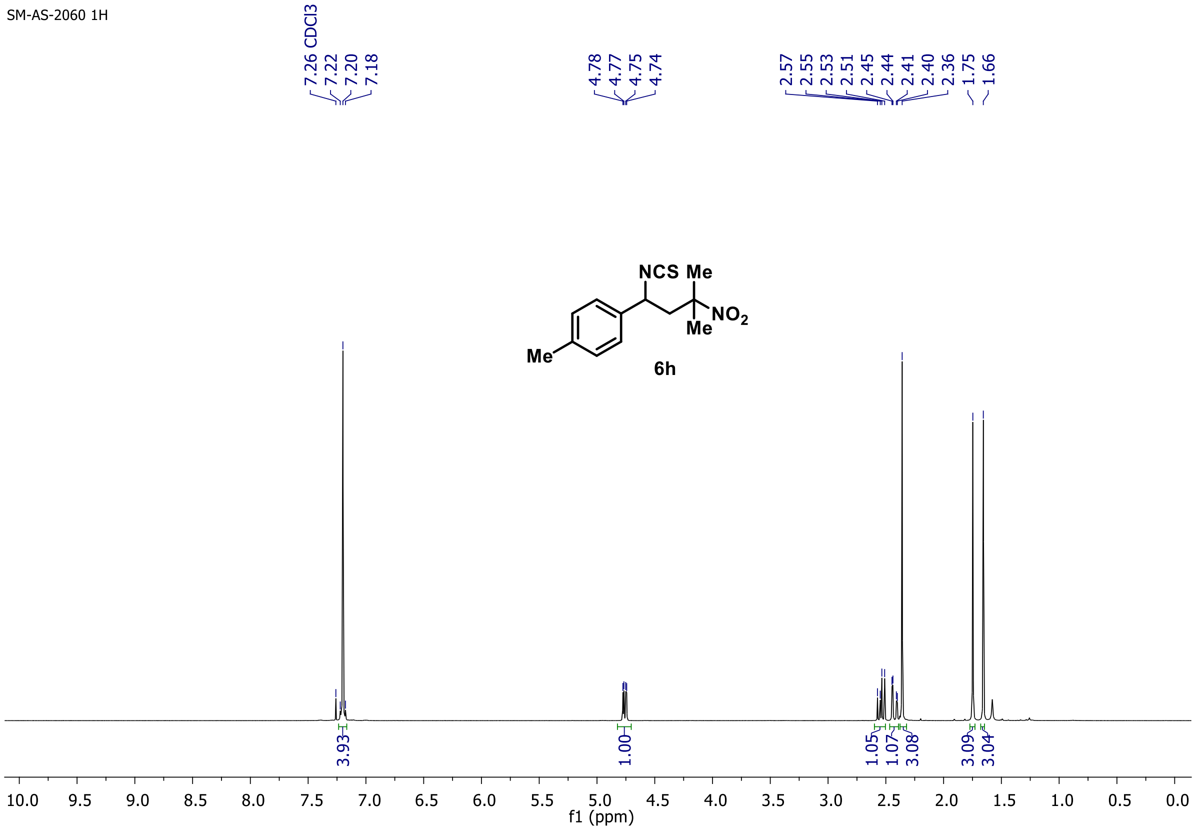


$^{13}$C{$^{1}$H} NMR of **6h** (101 MHz, $CDCl_3$):

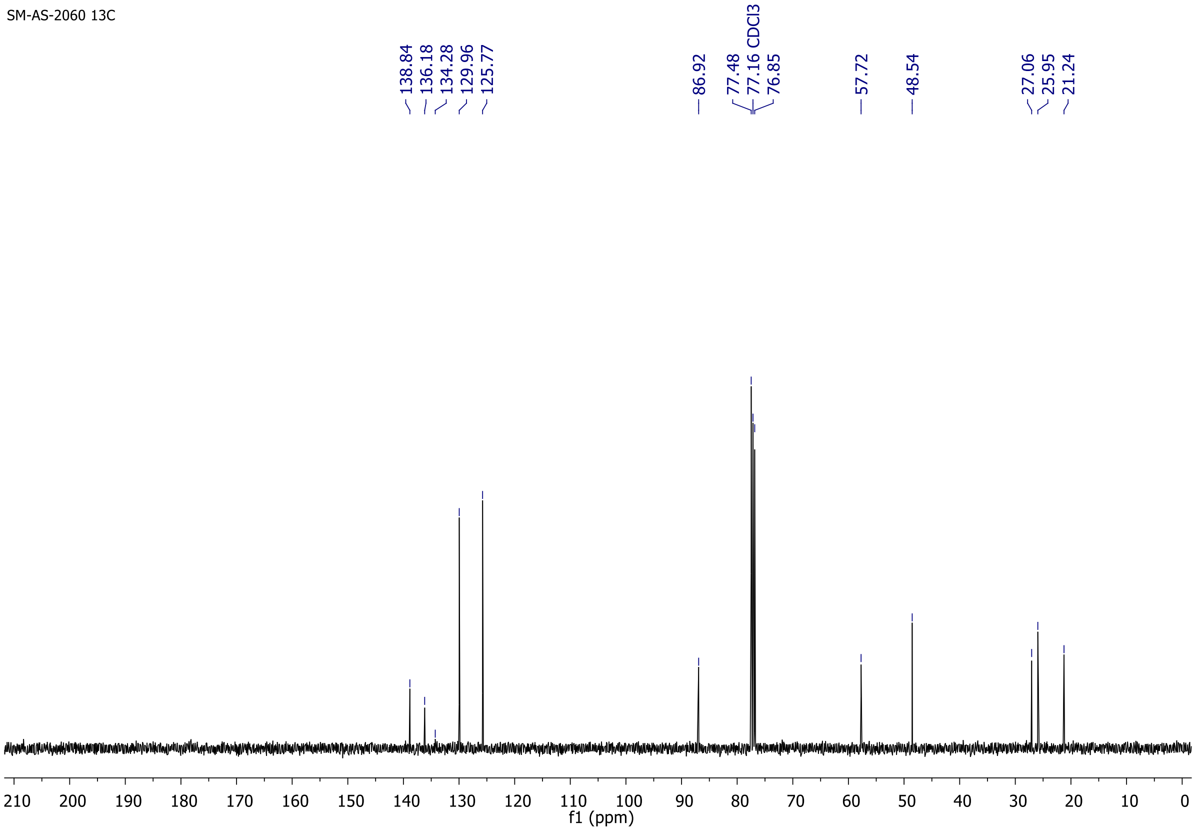

$^1H$ NMR of **6i** (400 MHz, $CDCl_3$):

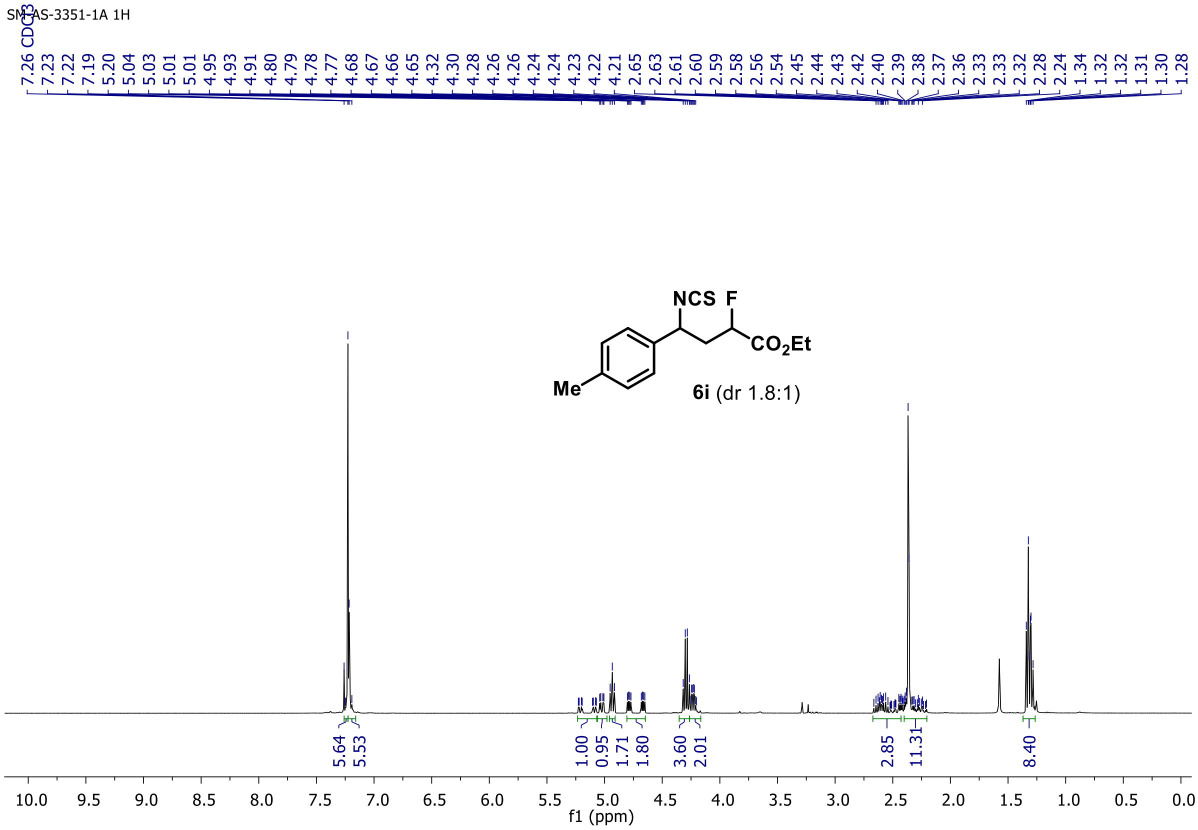


$^{13}C\{^1H\}$ NMR of **6i** (101 MHz, $CDCl_3$):

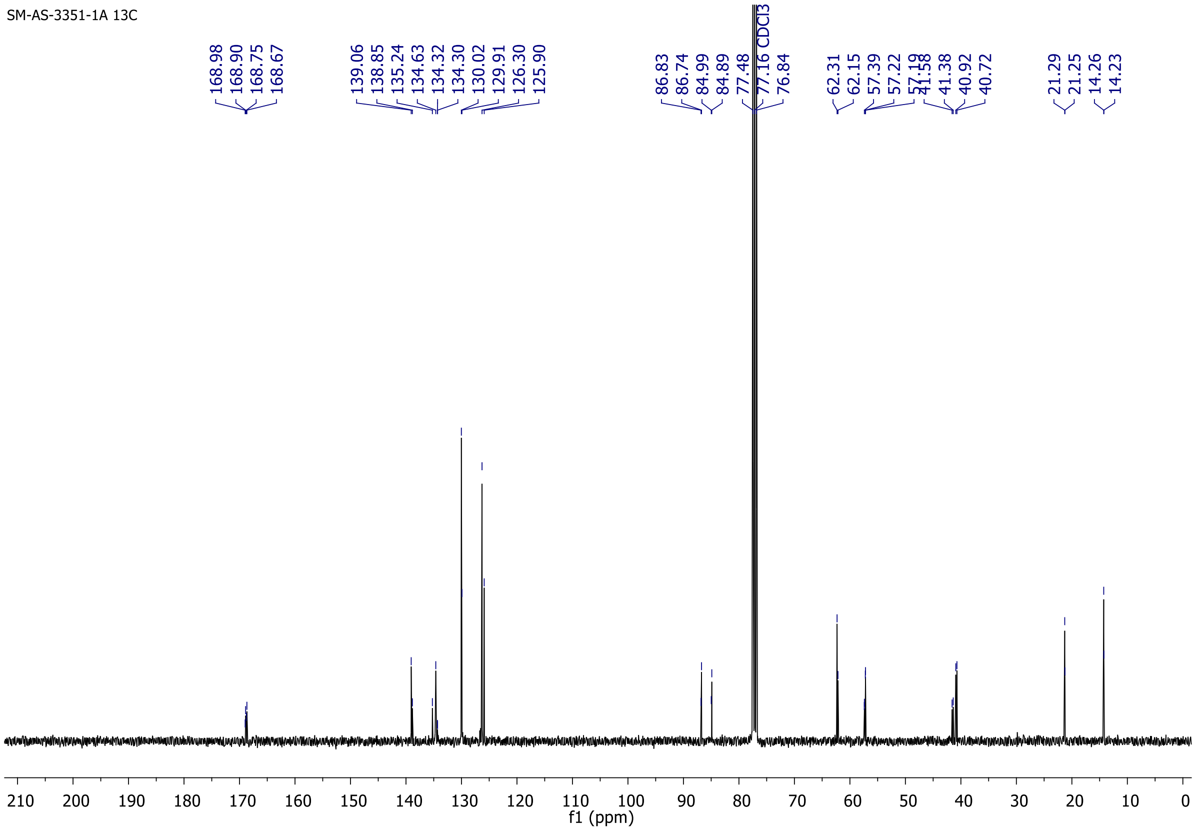

$^{19}$F NMR of **6i** (377 MHz, $CDCl_3$):

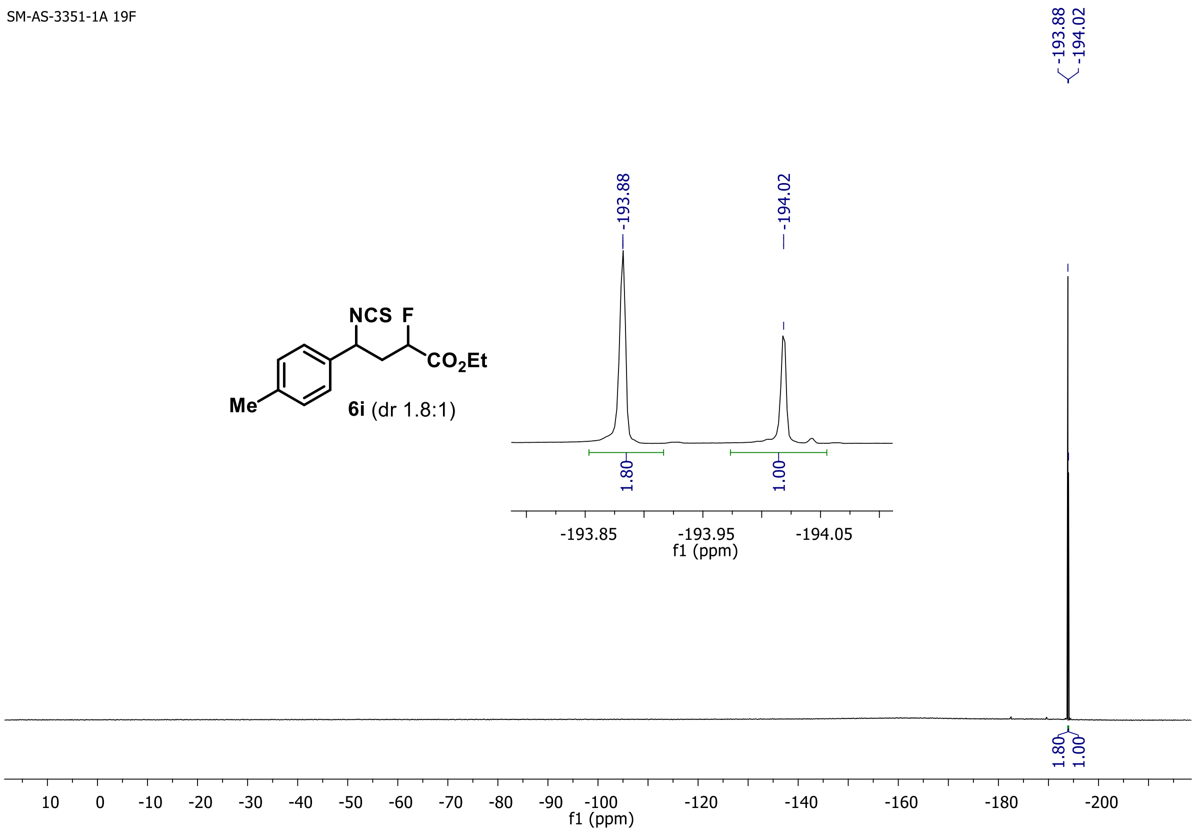

$^{1}H$ NMR of **7a** (400 MHz, DMSO-d$_6$):

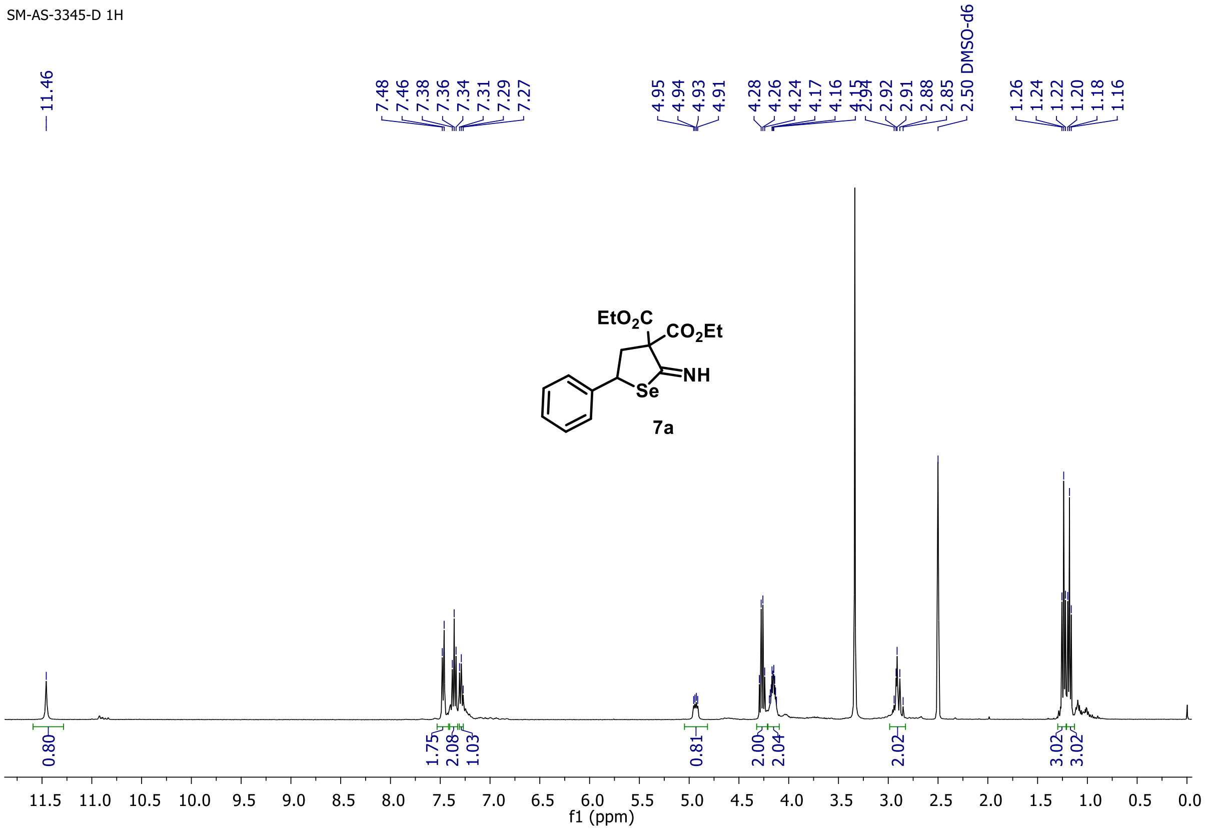


$^{13}C\{^{1}H\}$ NMR of **7a** (101 MHz, DMSO-d$_6$):

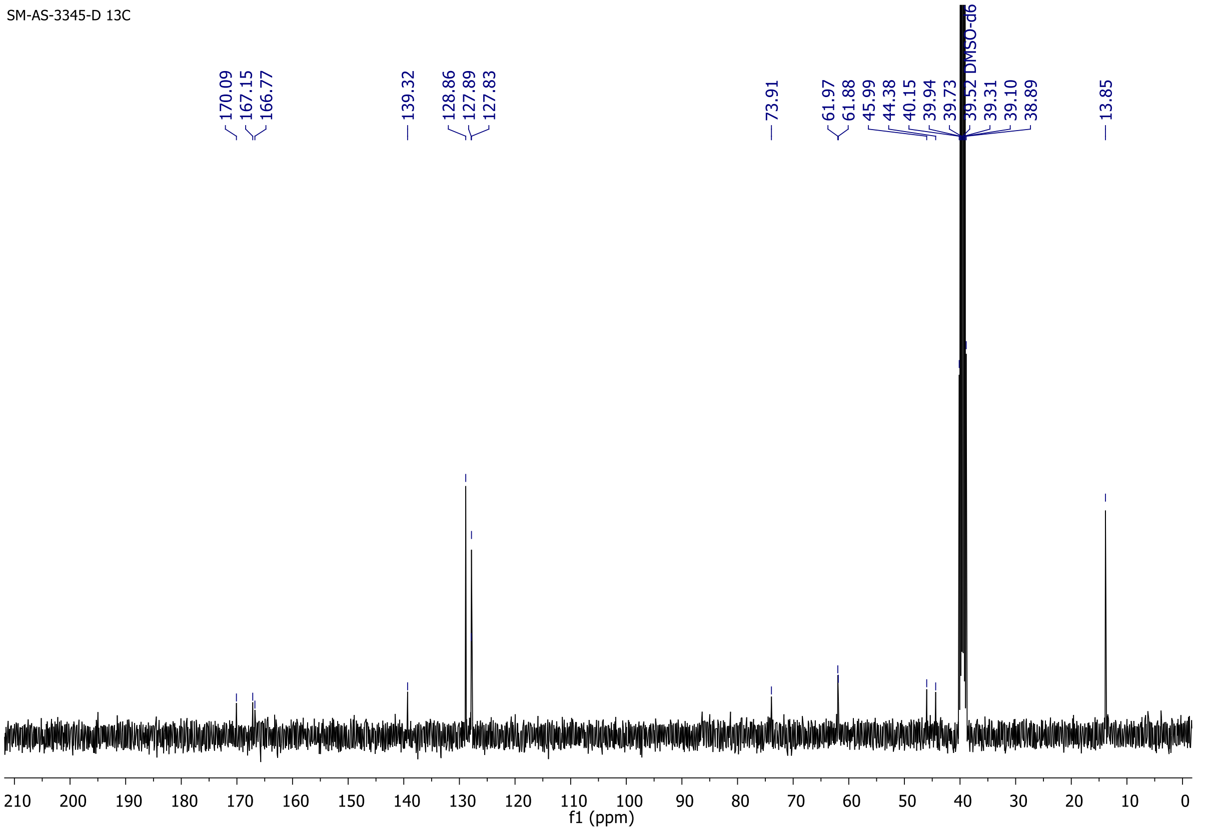

$^1$H NMR of **7b** (400 MHz, DMSO-d$_6$):

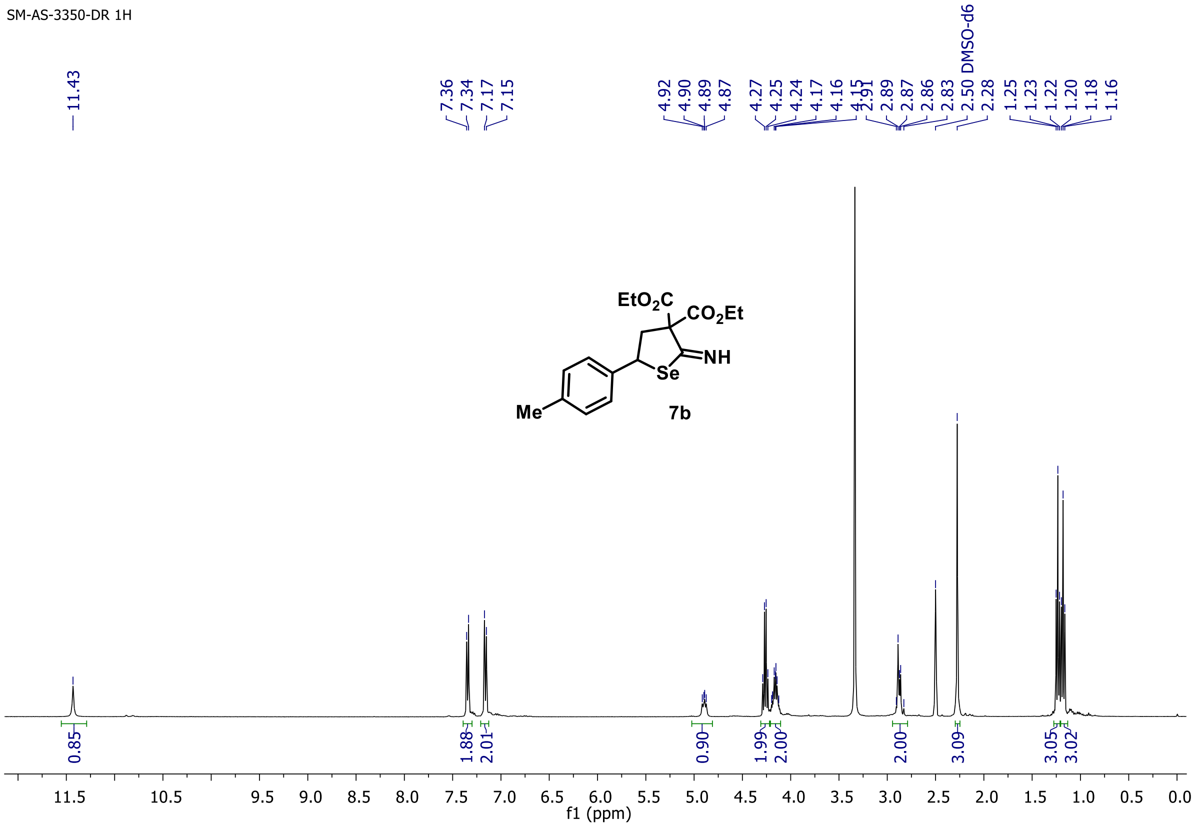


$^{13}$C{$^1$H} NMR of **7b** (101 MHz, DMSO-d$_6$):

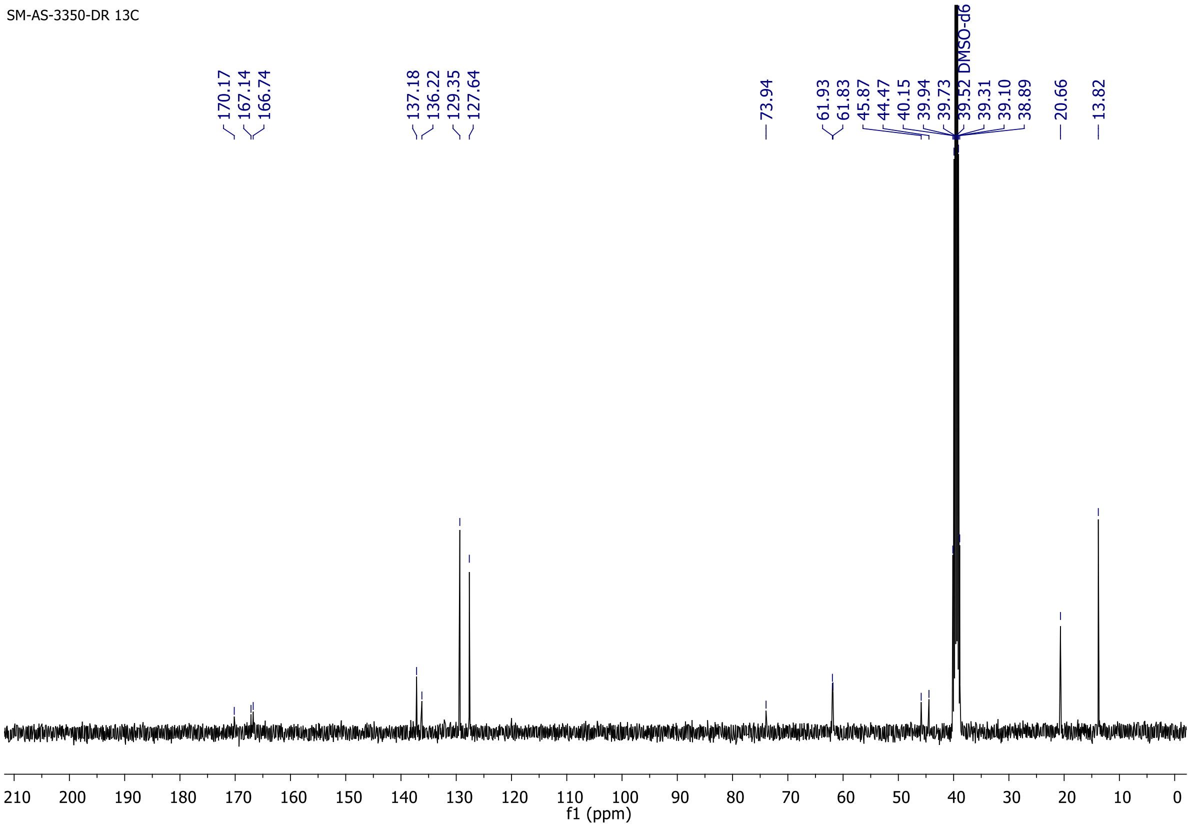

$^{1}H$ NMR of **7c** (400 MHz, DMSO-d$_6$):

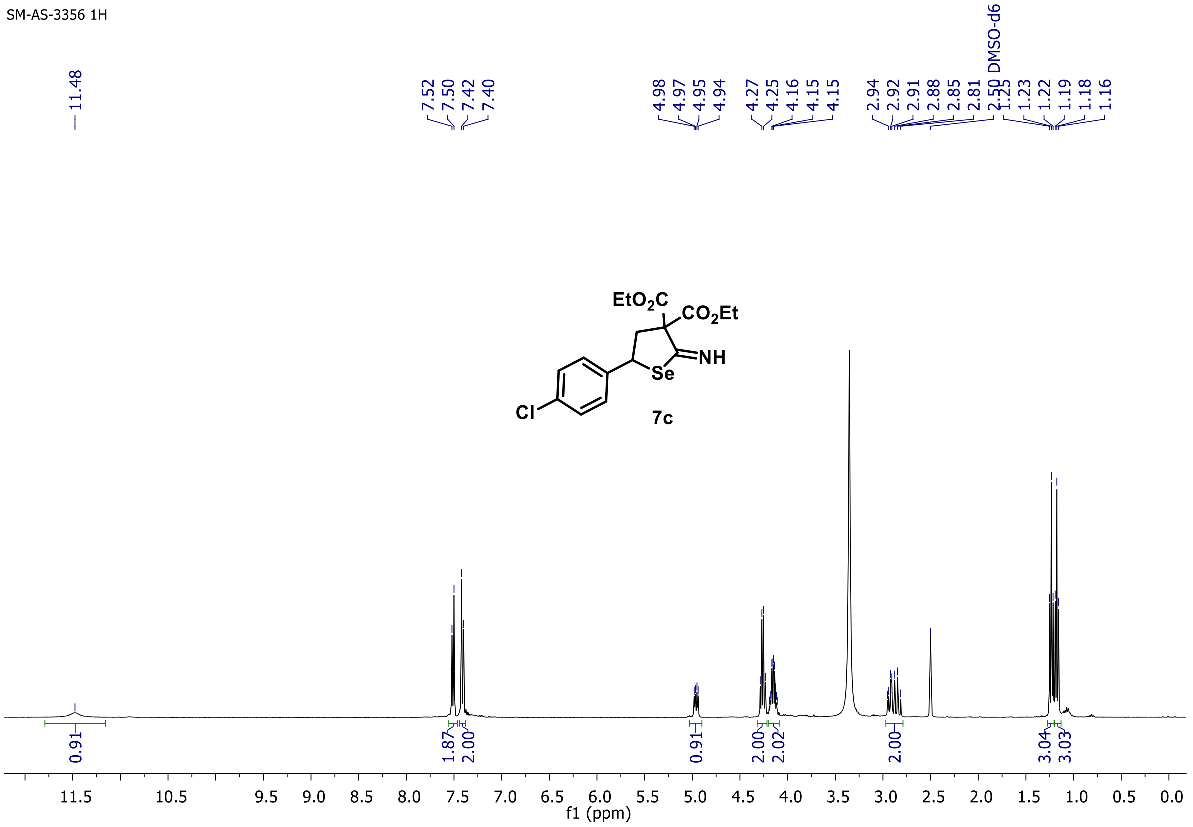


$^{13}C\{^{1}H\}$ NMR of **7c** (101 MHz, DMSO-d$_6$):

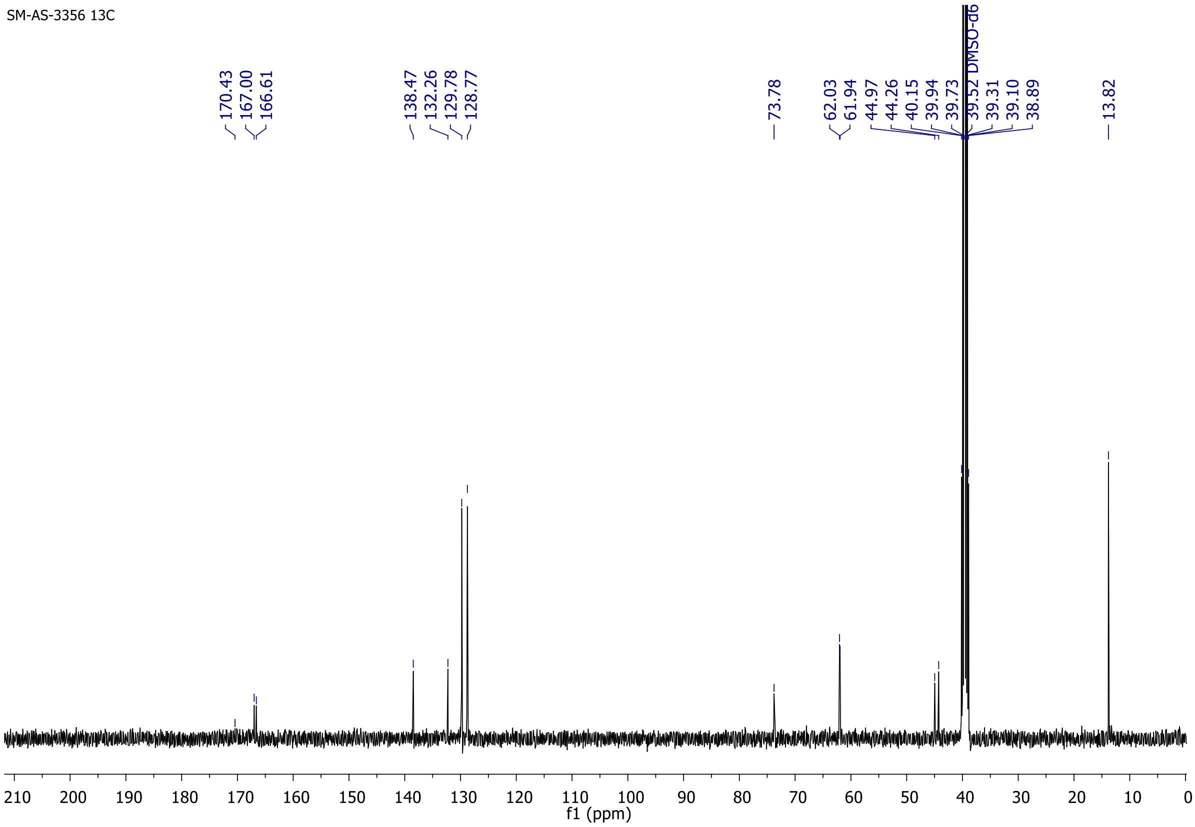

$^{1}$H NMR of **7d** (400 MHz, DMSO-d$_6$):

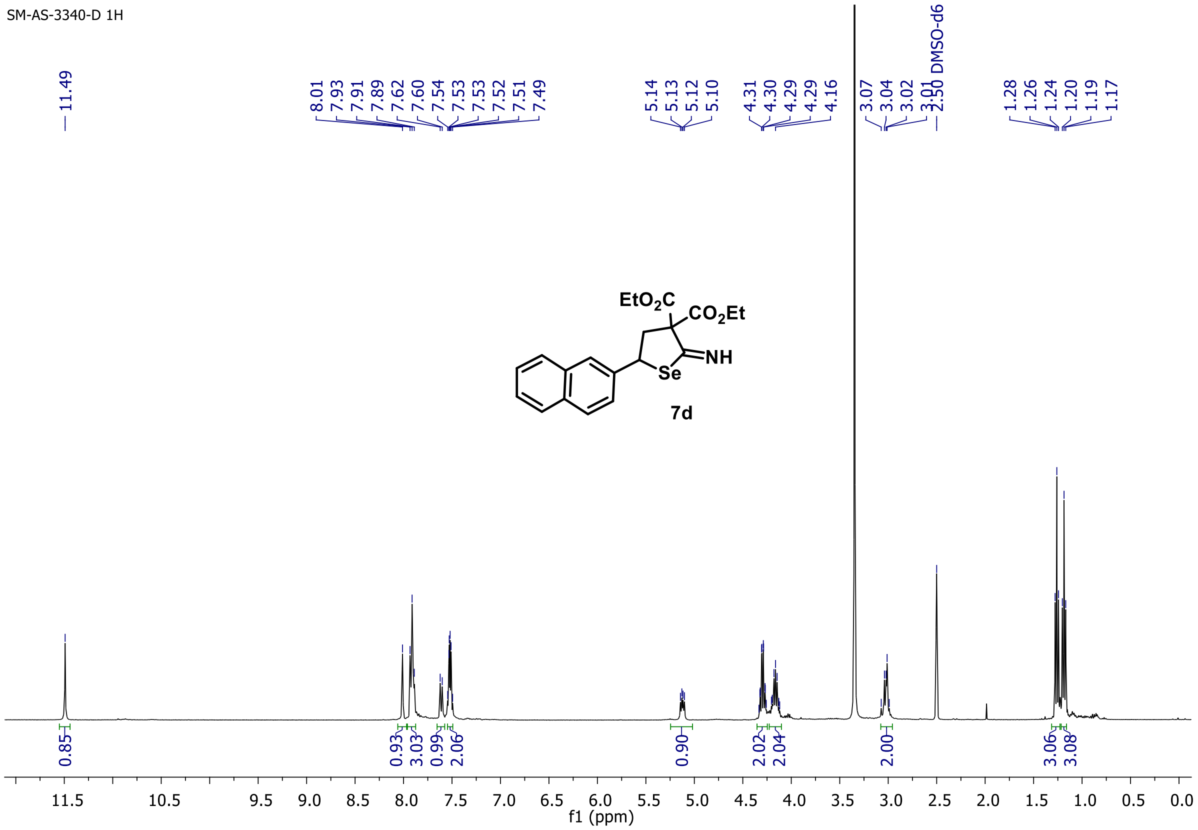


$^{13}$C{$^{1}$H} NMR of **7d** (101 MHz, DMSO-d$_6$):

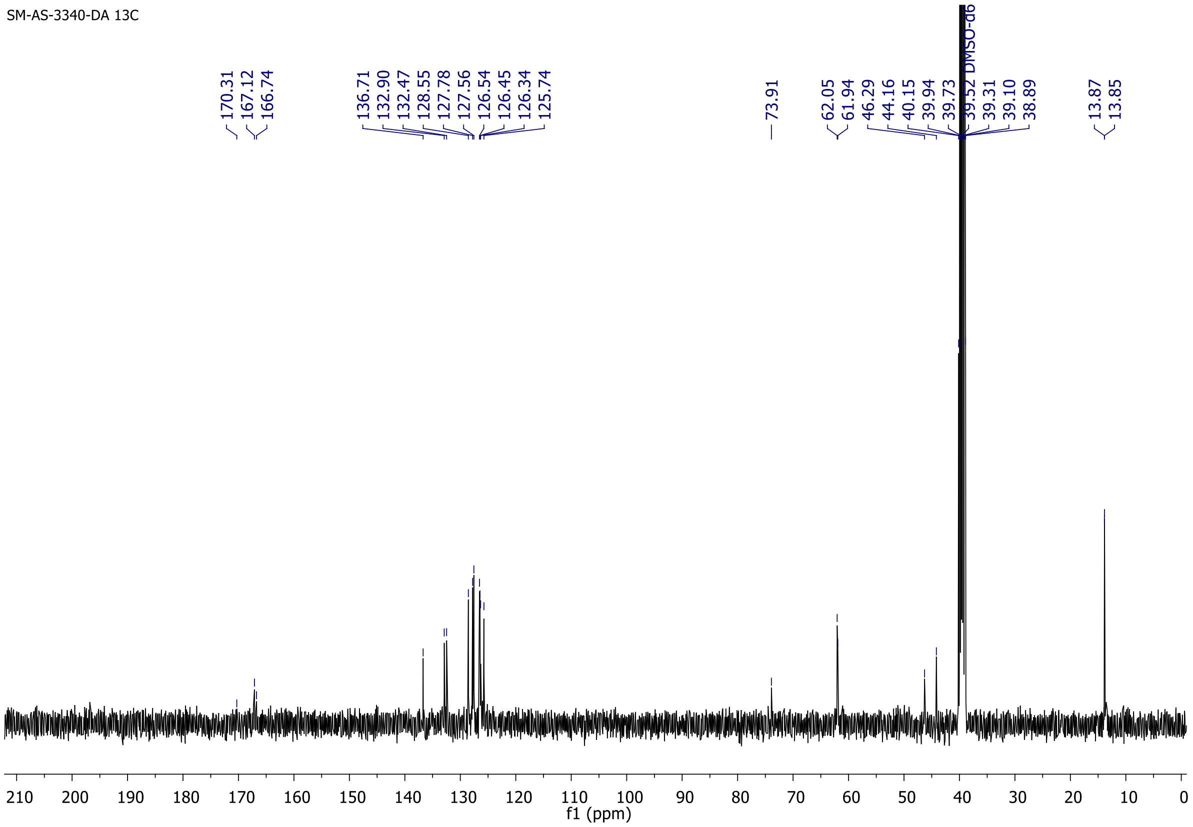

$^{1}$H NMR of **7e** (400 MHz, DMSO-d6):

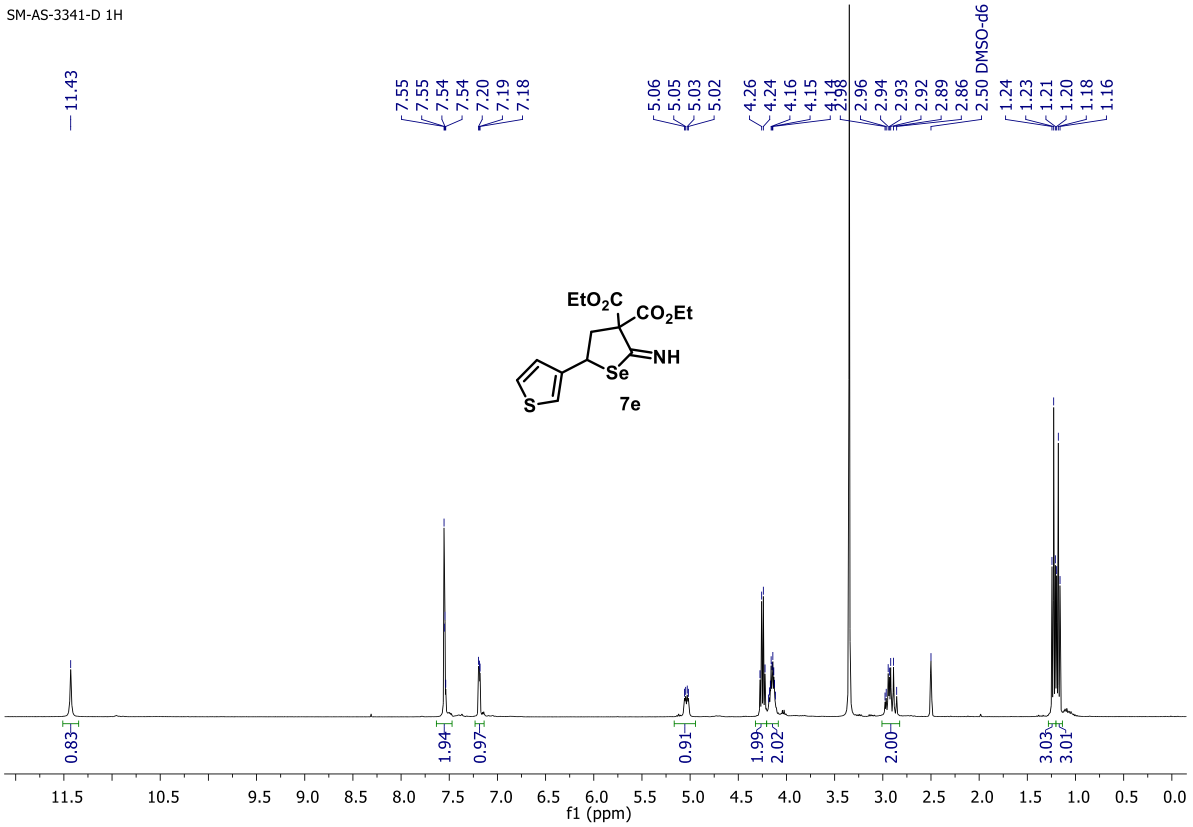


$^{13}$C{$^{1}$H} NMR of **7e** (101 MHz, DMSO-d6):

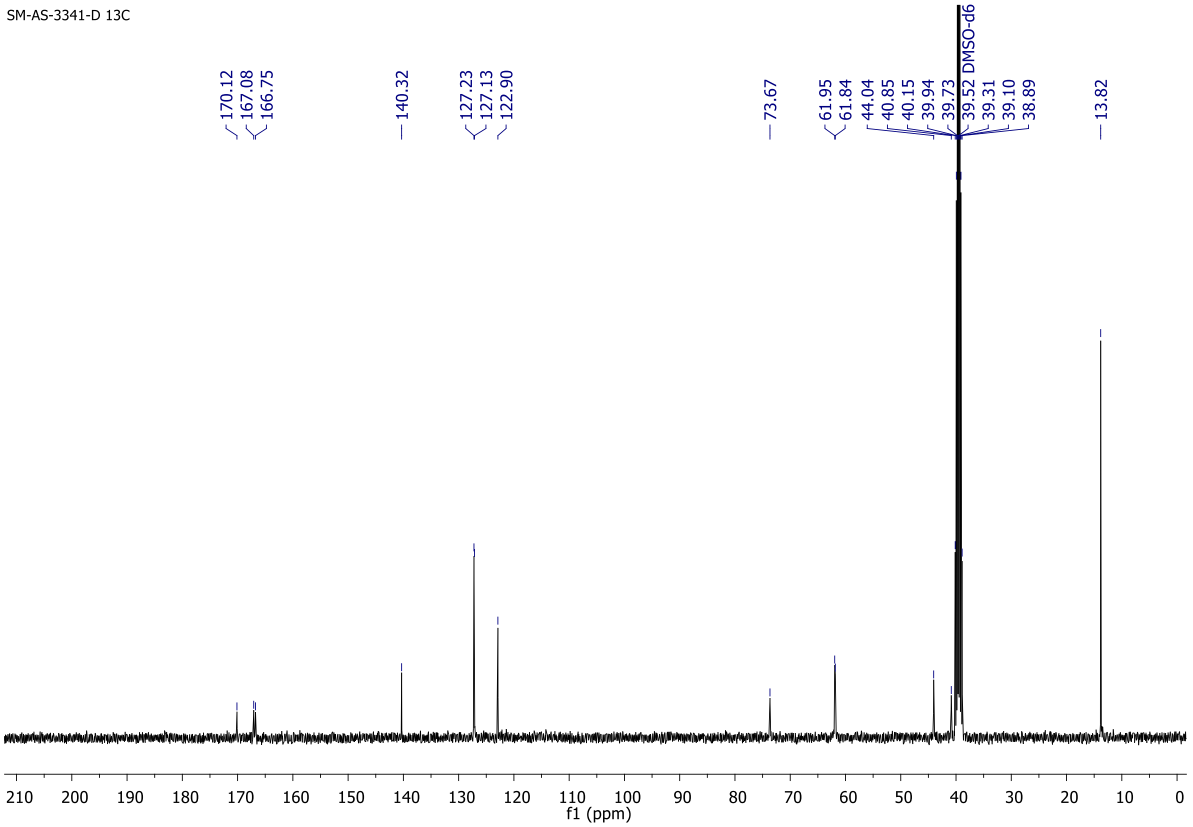

$^1$H NMR of **7f** (400 MHz, DMSO-d$_6$):

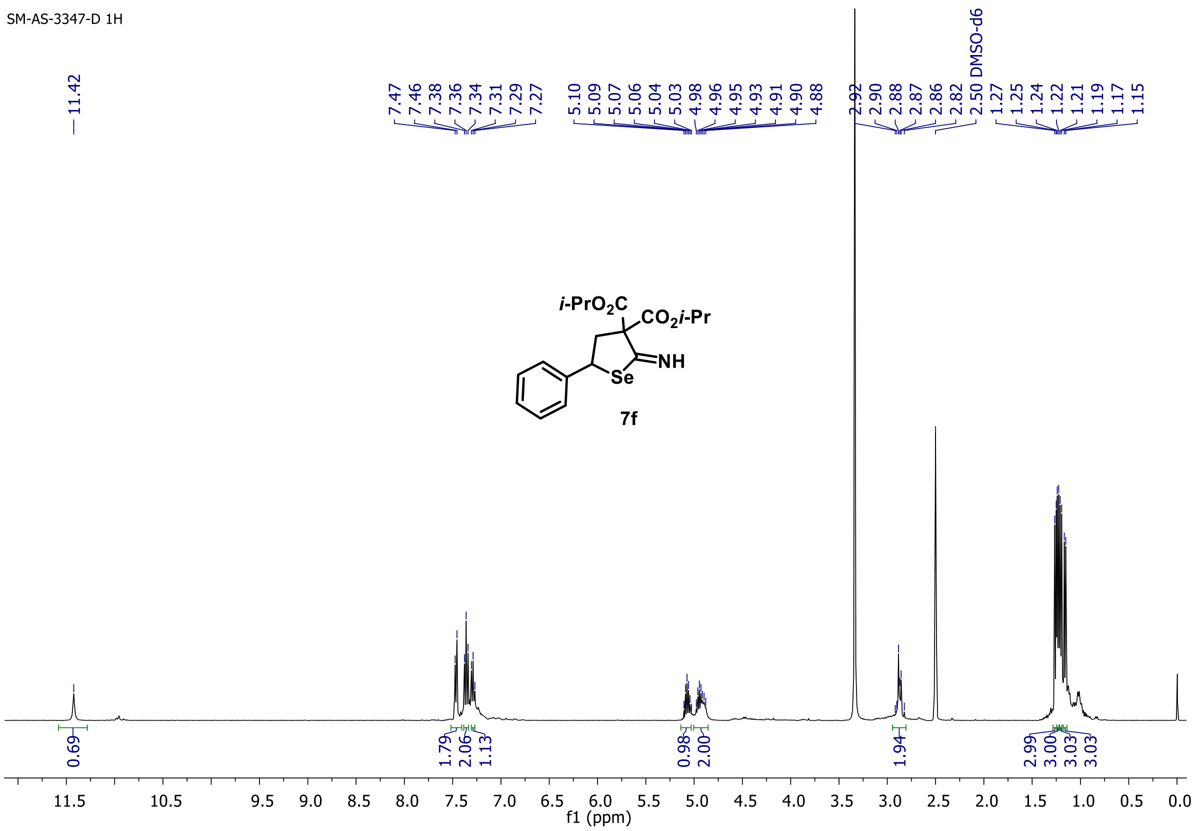


$^{13}$C{$^1$H} NMR of **7f** (101 MHz, DMSO-d$_6$):

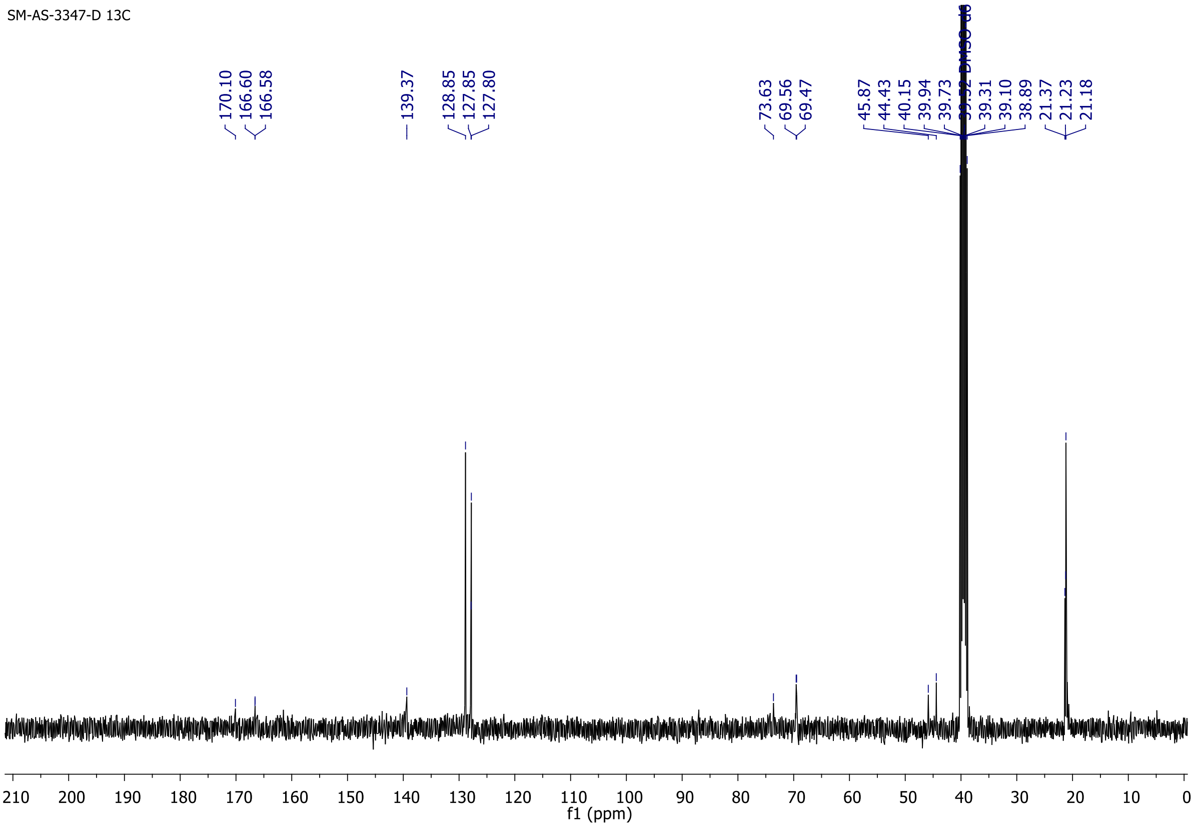

$^{1}$H NMR of **7g** (400 MHz, $CDCl_3$):

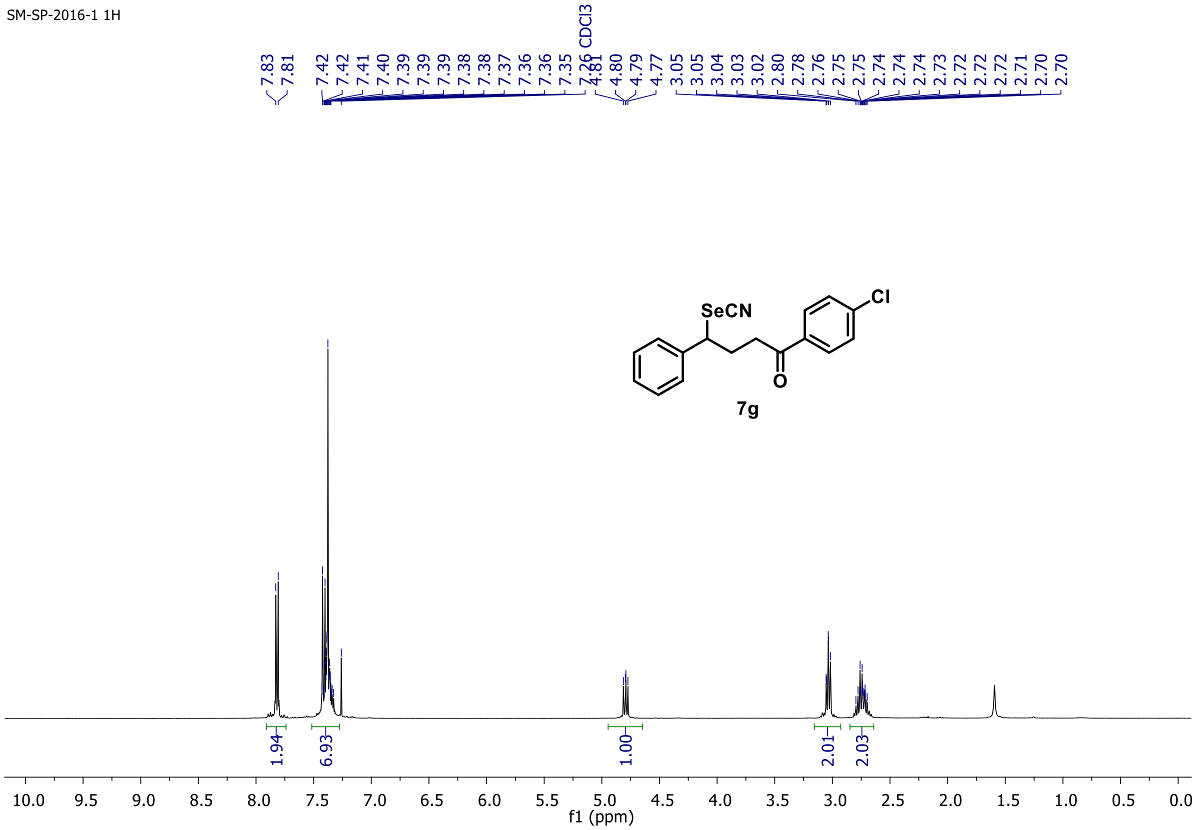


$^{13}$C{$^{1}$H} NMR of **7g** (101 MHz, $CDCl_3$):

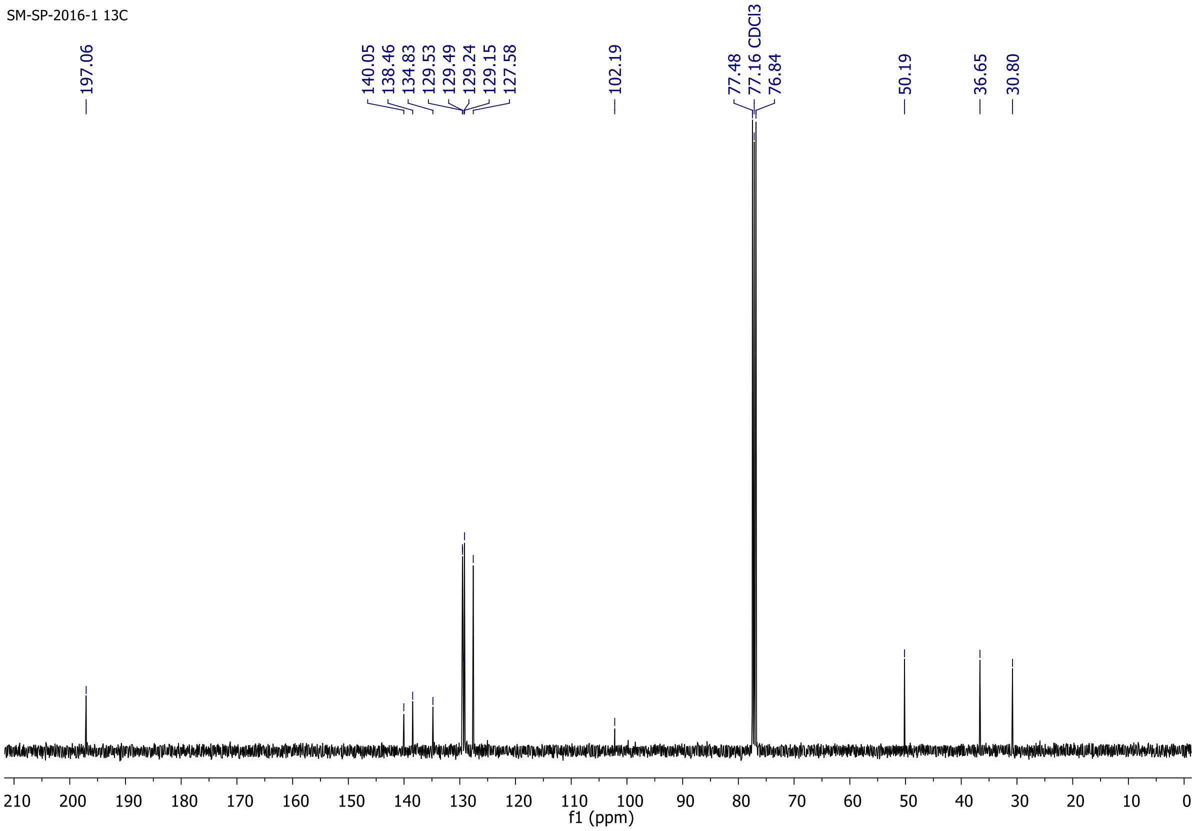

$^{1}H$ NMR of **8a** (400 MHz, $CDCl_3$):

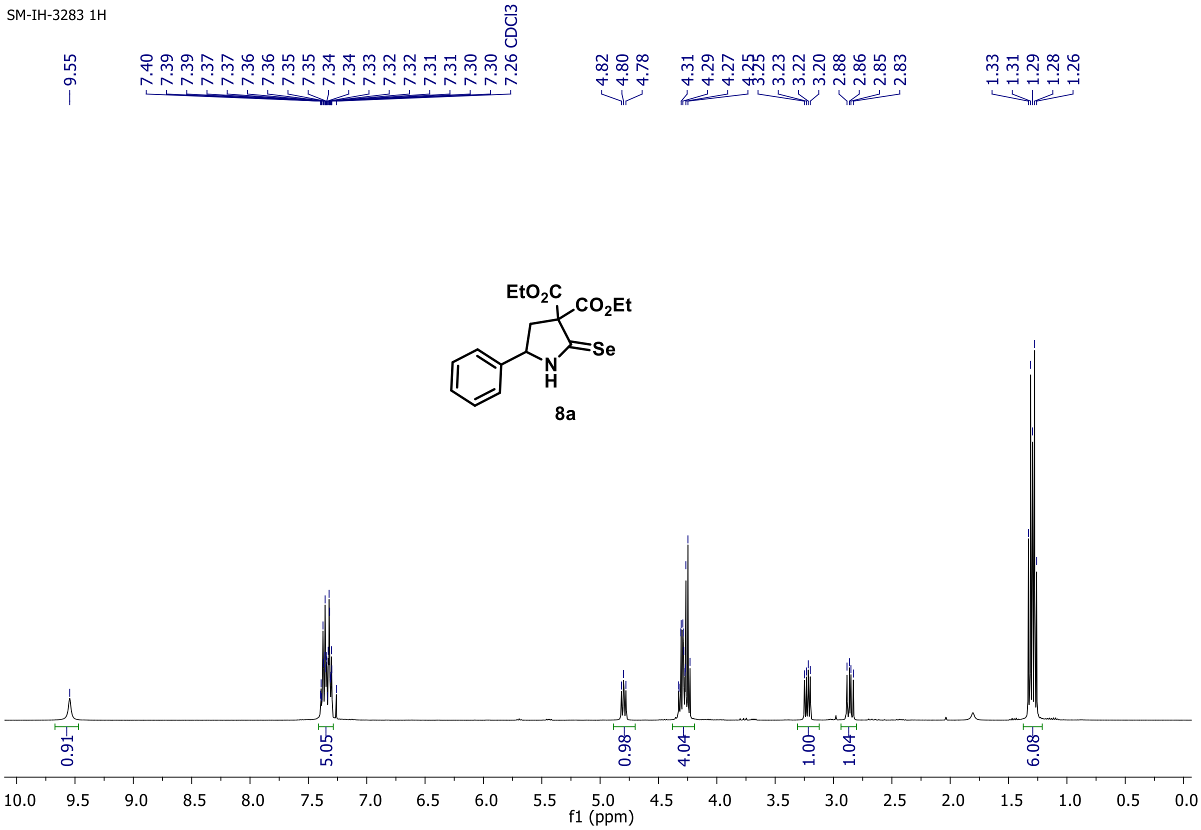


$^{13}C\{^{1}H\}$ NMR of **8a** (101 MHz, $CDCl_3$):

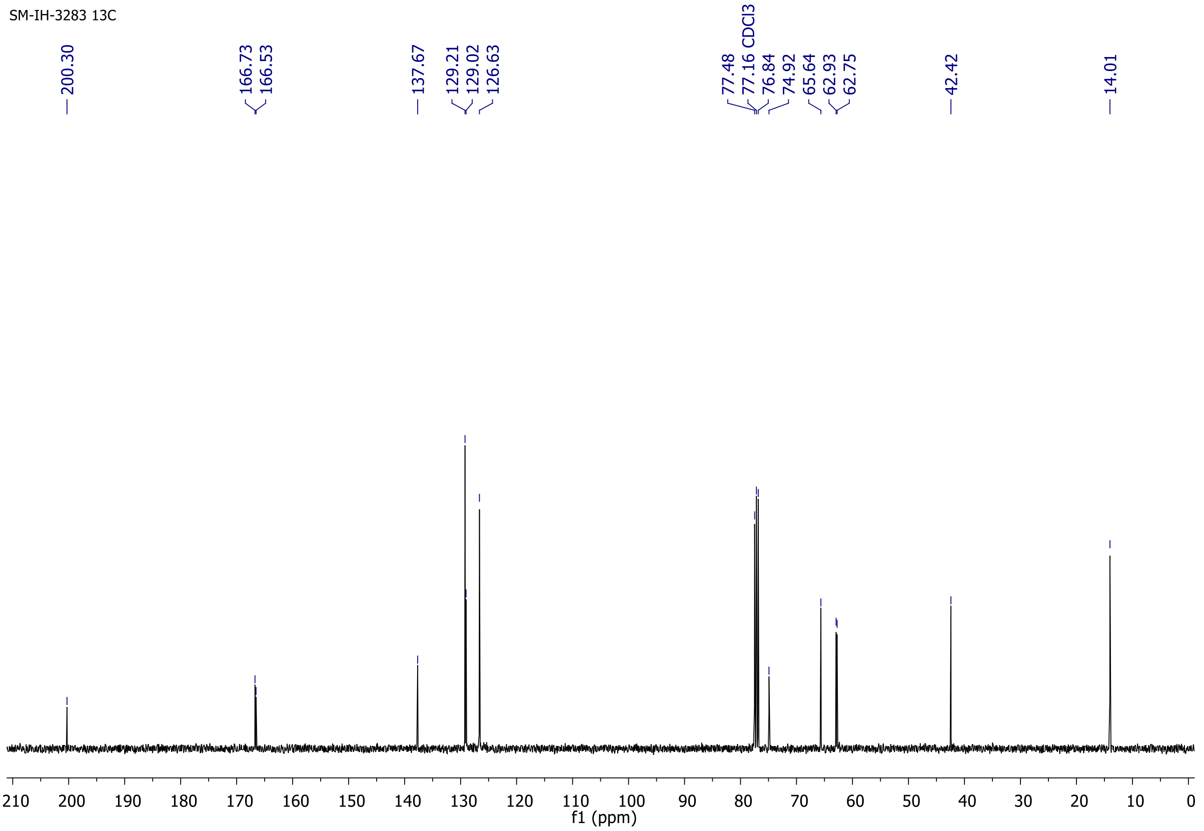

$^{1}$H NMR of **8b** (400 MHz, DMSO-$d_6$):

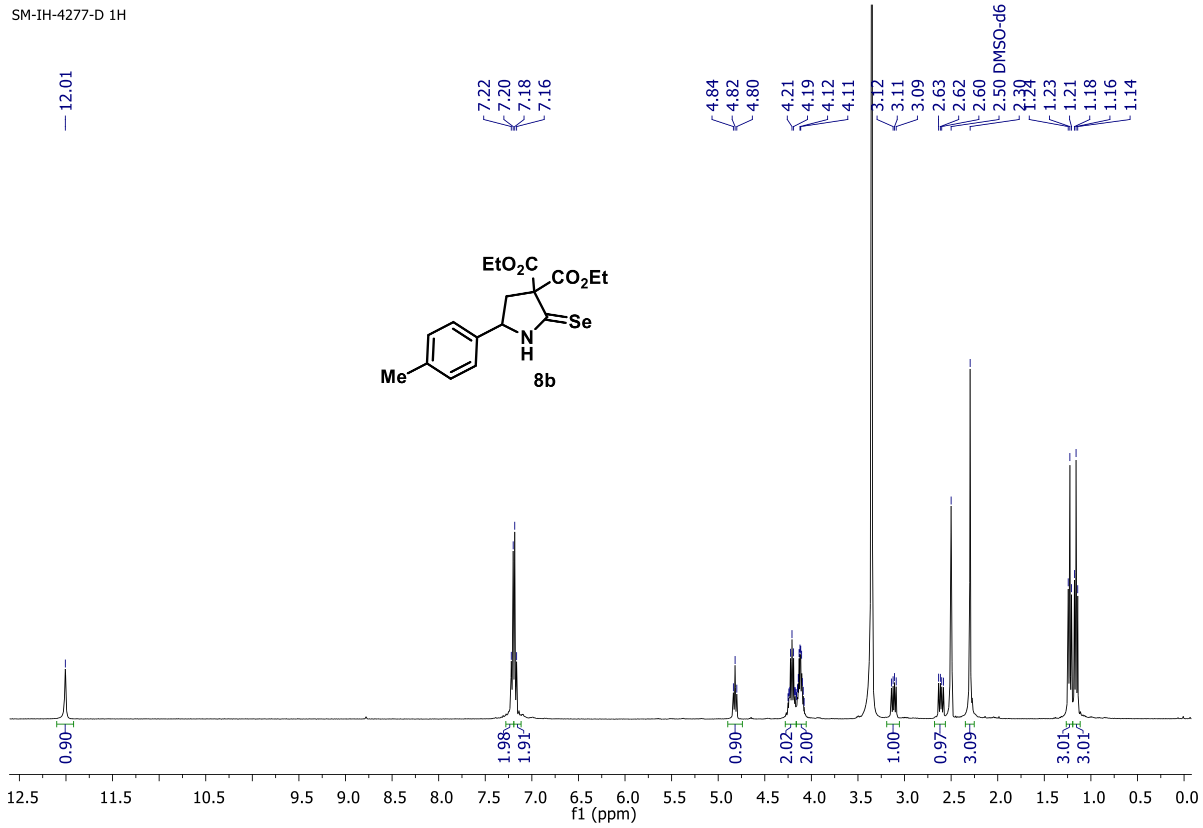


$^{13}$C{$^{1}$H} NMR of **8b** (101 MHz, DMSO-$d_6$):

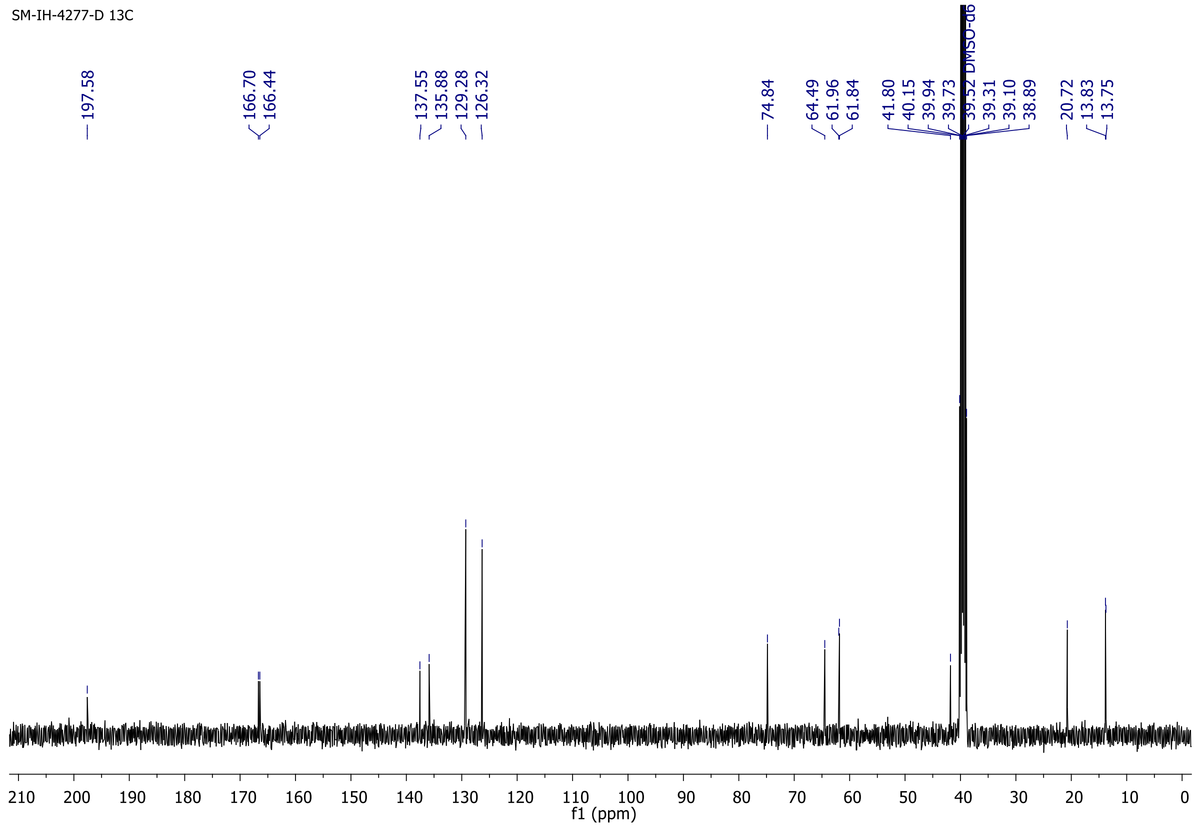

$^{1}$H NMR of **8c** (400 MHz, DMSO-d$_6$):

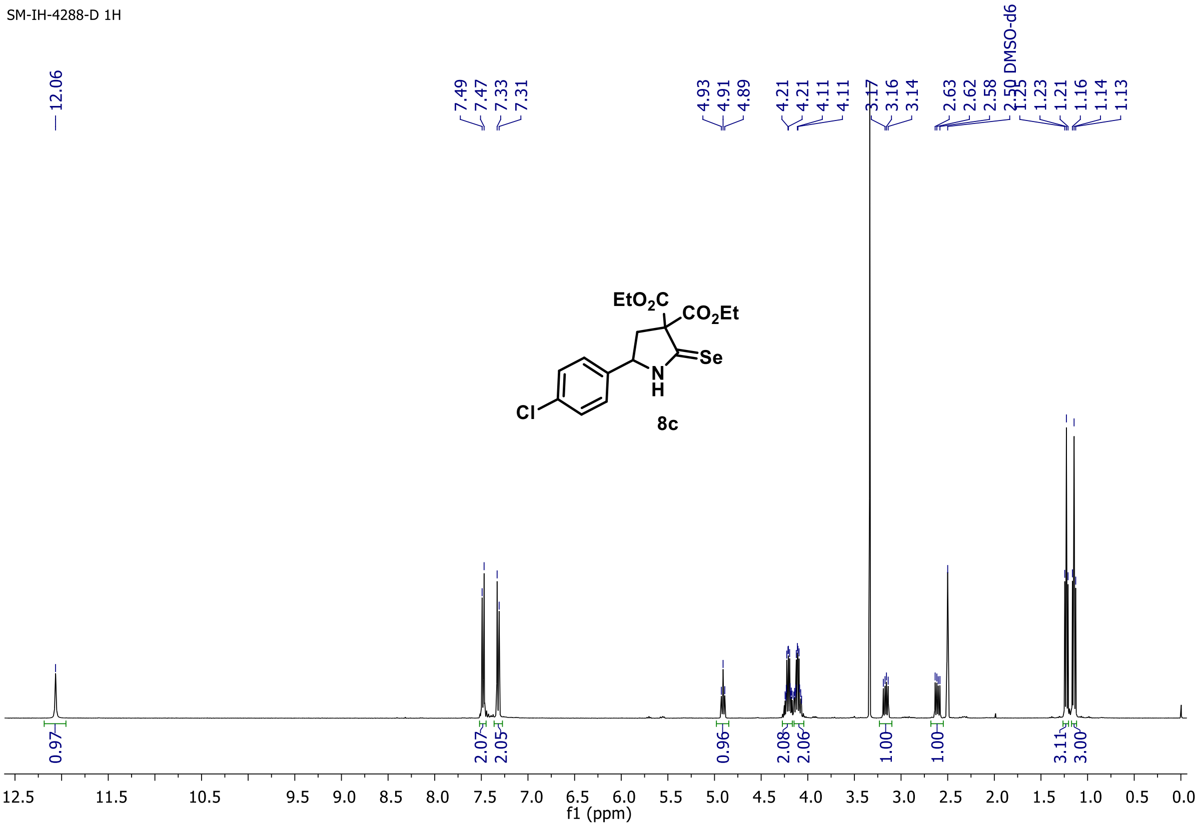


$^{13}$C{$^{1}$H} NMR of **8c** (101 MHz, DMSO-d$_6$):

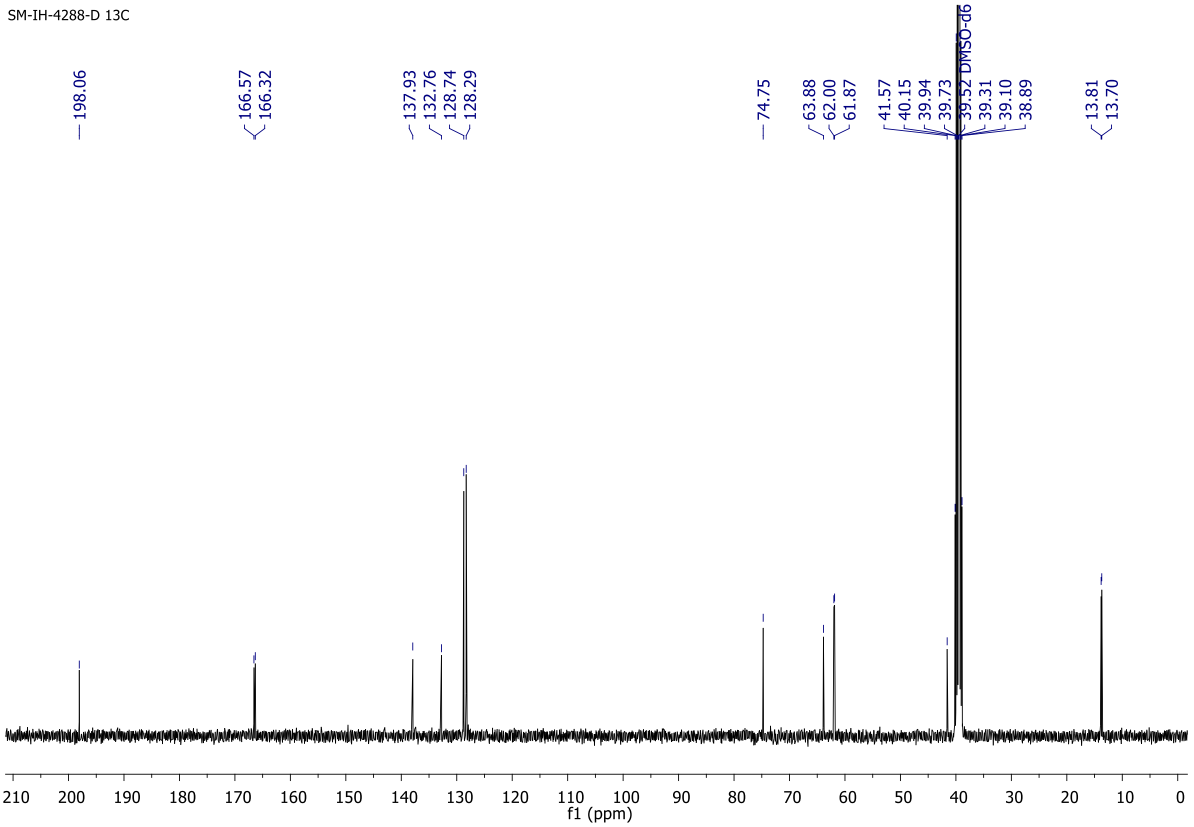

$^{1}H$ NMR of **8d** (400 MHz, DMSO-d6):

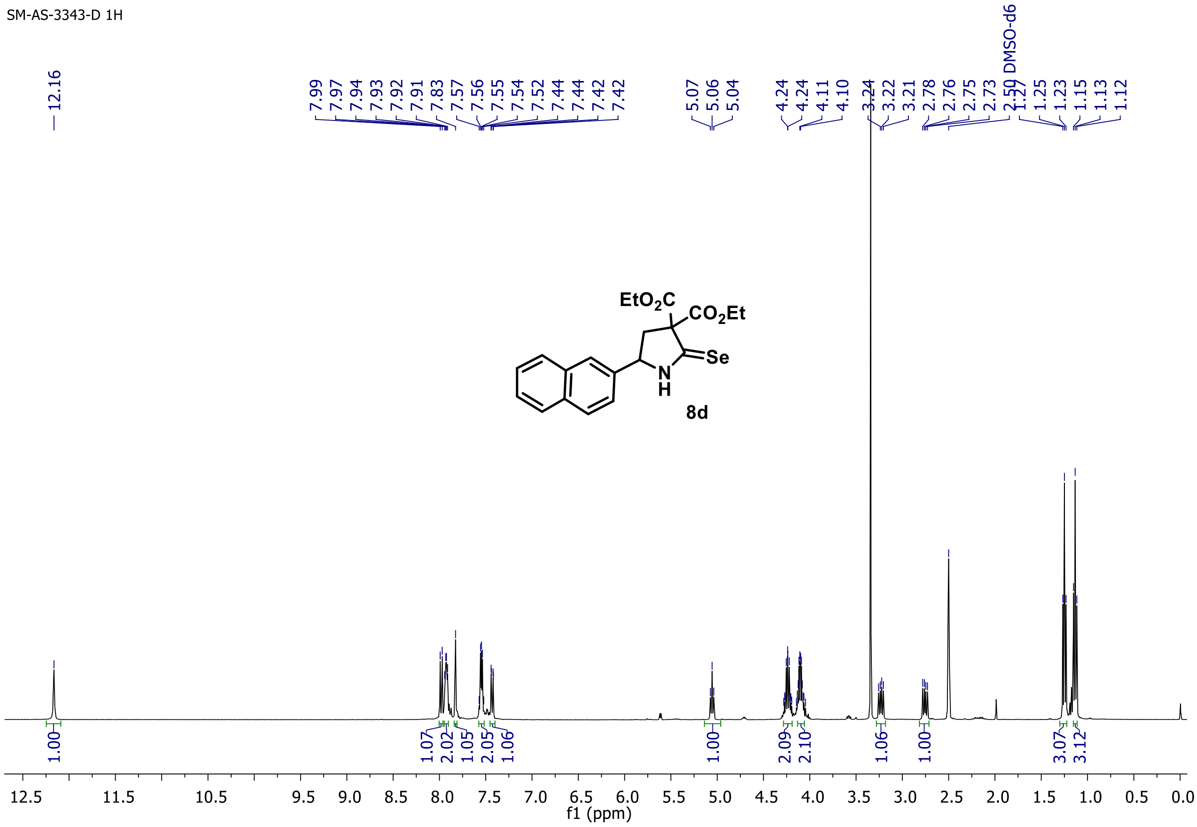


$^{13}C\{^{1}H\}$ NMR of **8d** (101 MHz, DMSO-d6):

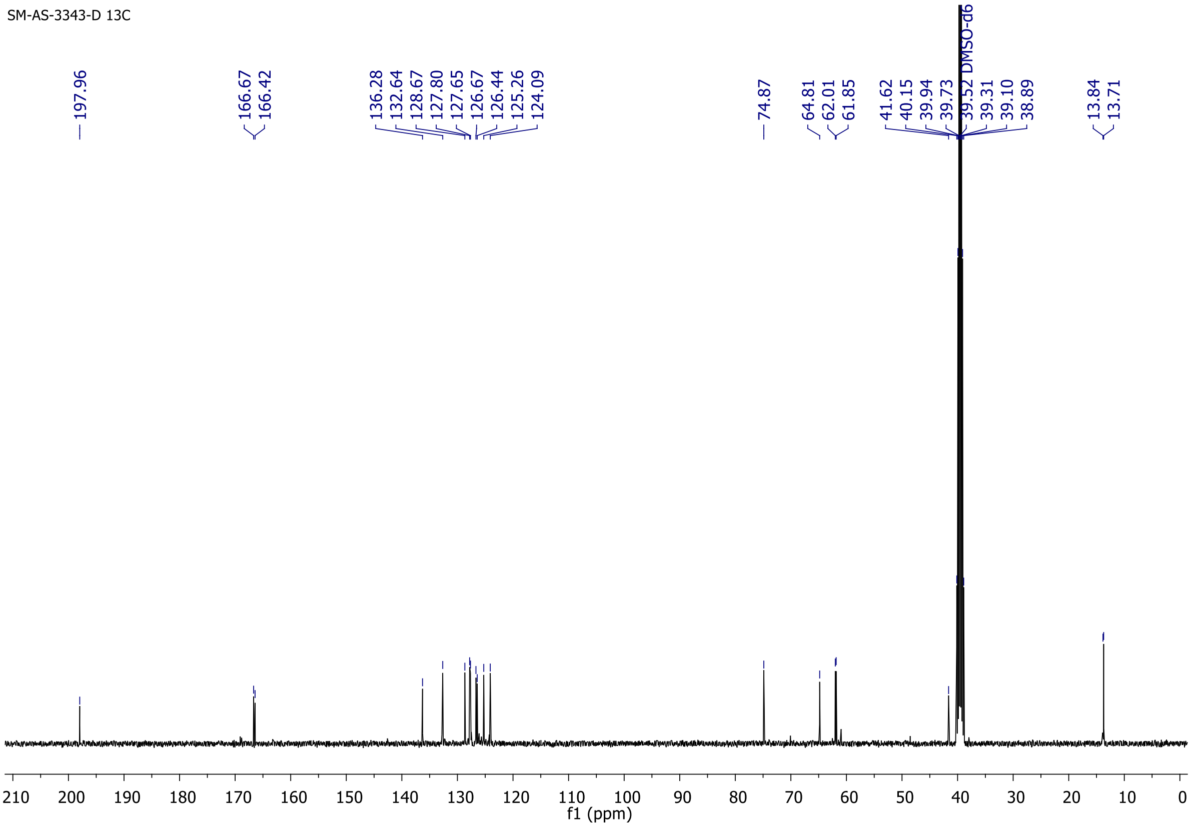

$^{1}H$ NMR of **8e** (400 MHz, $CDCl_3$):

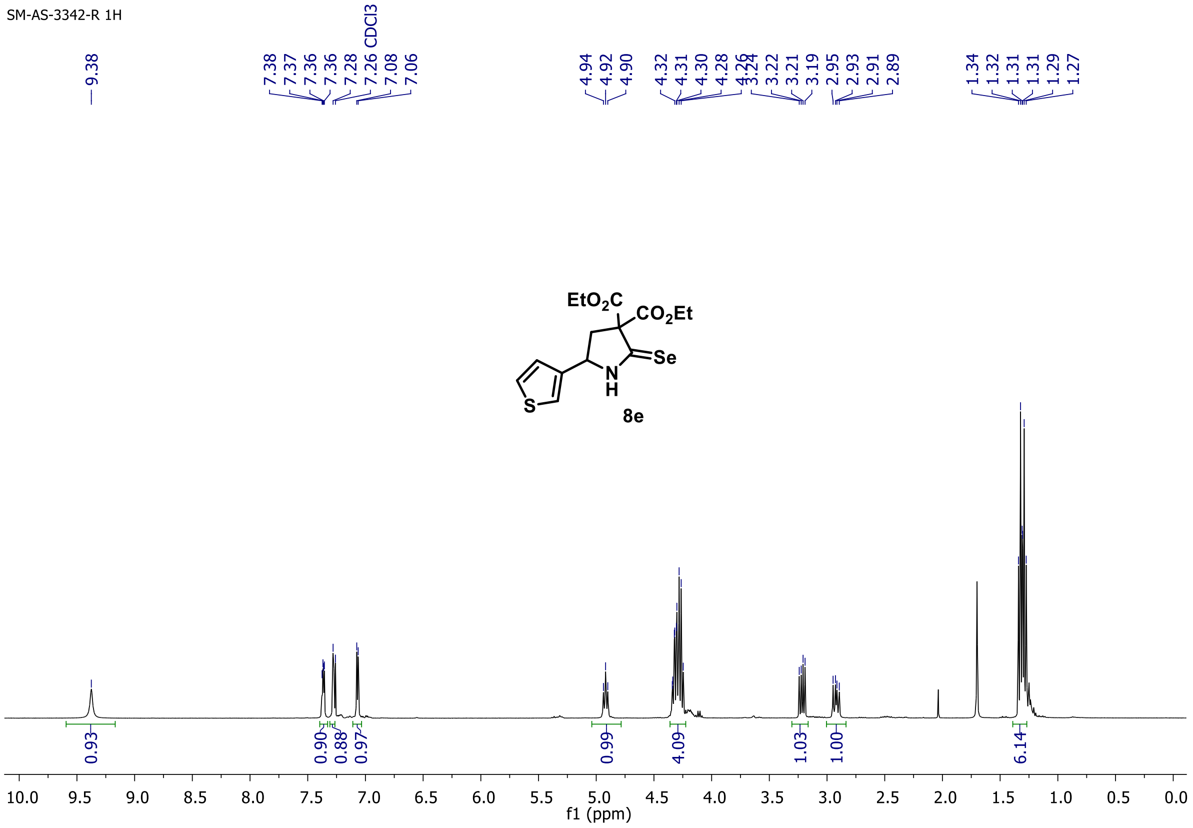


$^{13}C\{^{1}H\}$ NMR of **8e** (101 MHz, $CDCl_3$):

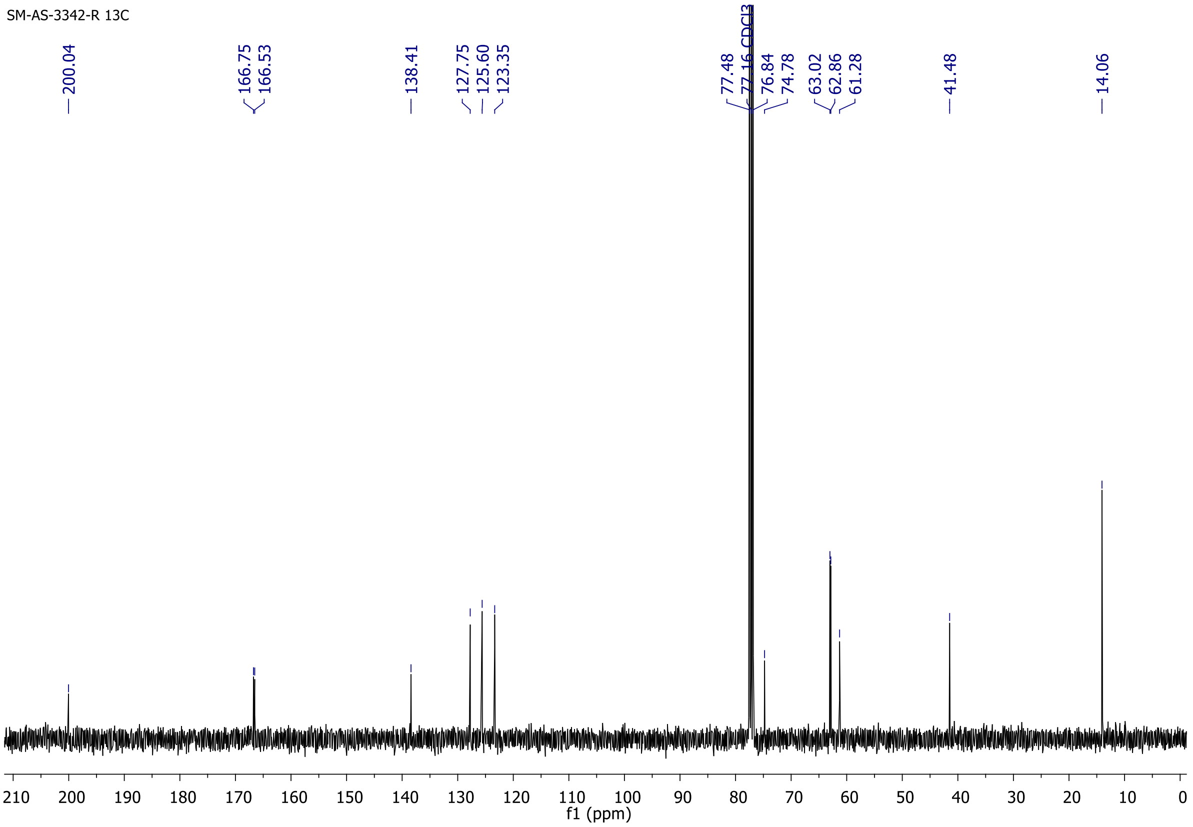

$^{1}H$ NMR of **8f** (400 MHz, DMSO-d$_6$):

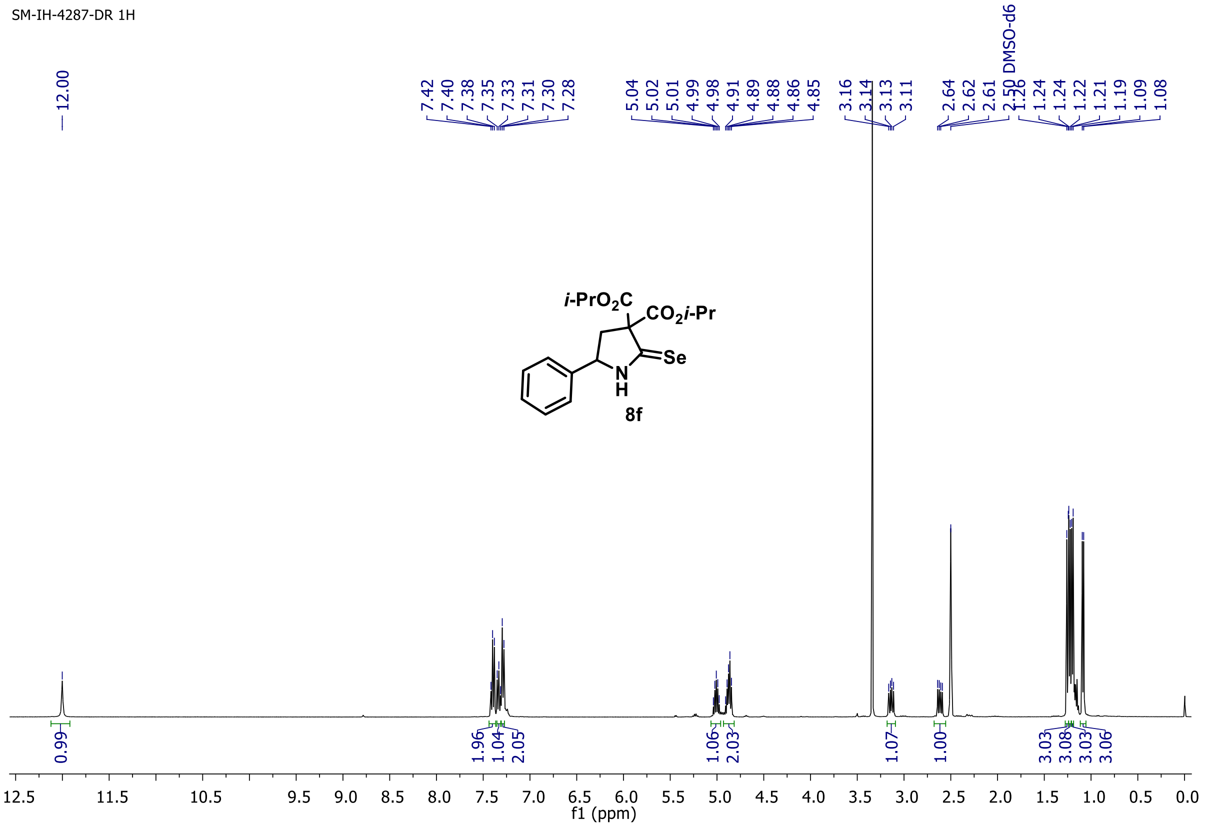


$^{13}C\{^{1}H\}$ NMR of **8f** (101 MHz, DMSO-d$_6$):

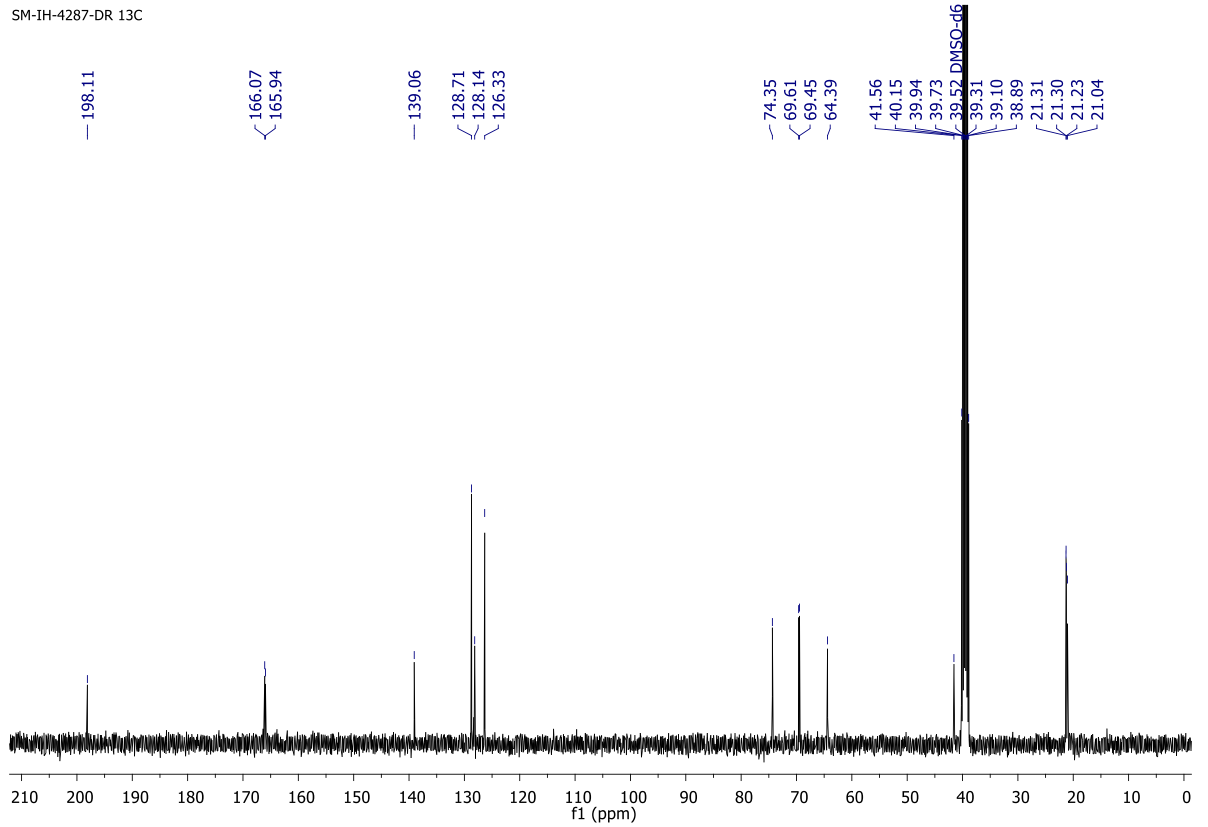

$^{1}H$ NMR of **8g** (400 MHz, $CDCl_3$):

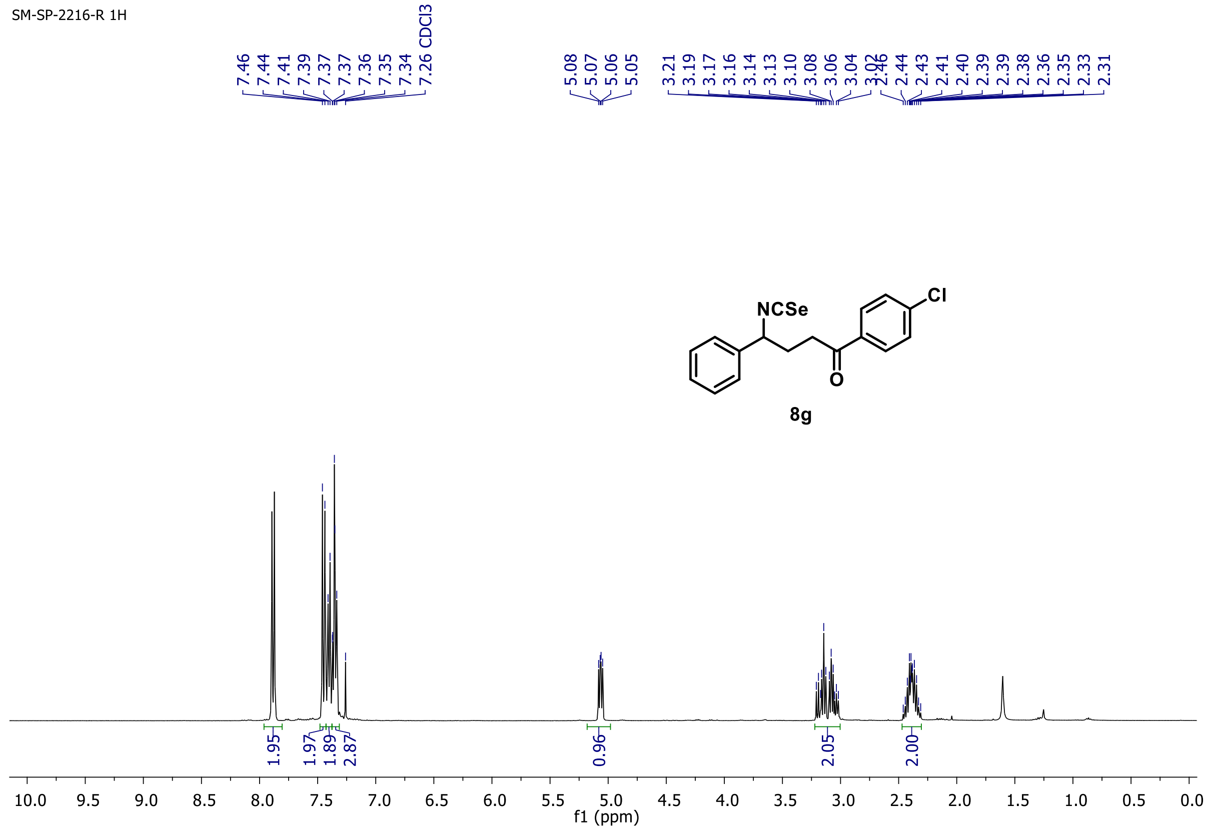


$^{13}C\{^{1}H\}$ NMR of **8g** (101 MHz, $CDCl_3$):

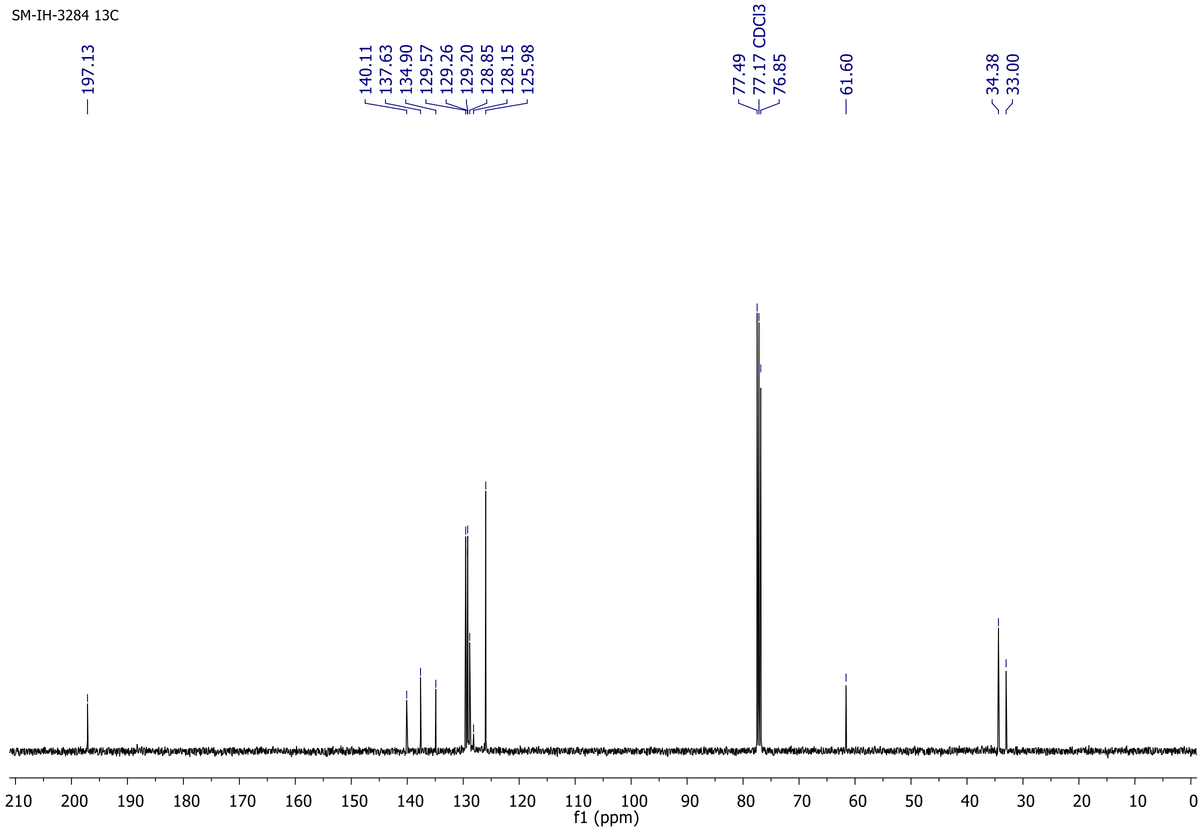

$^{1}H$ NMR of **9** (400 MHz, $CDCl_3$):

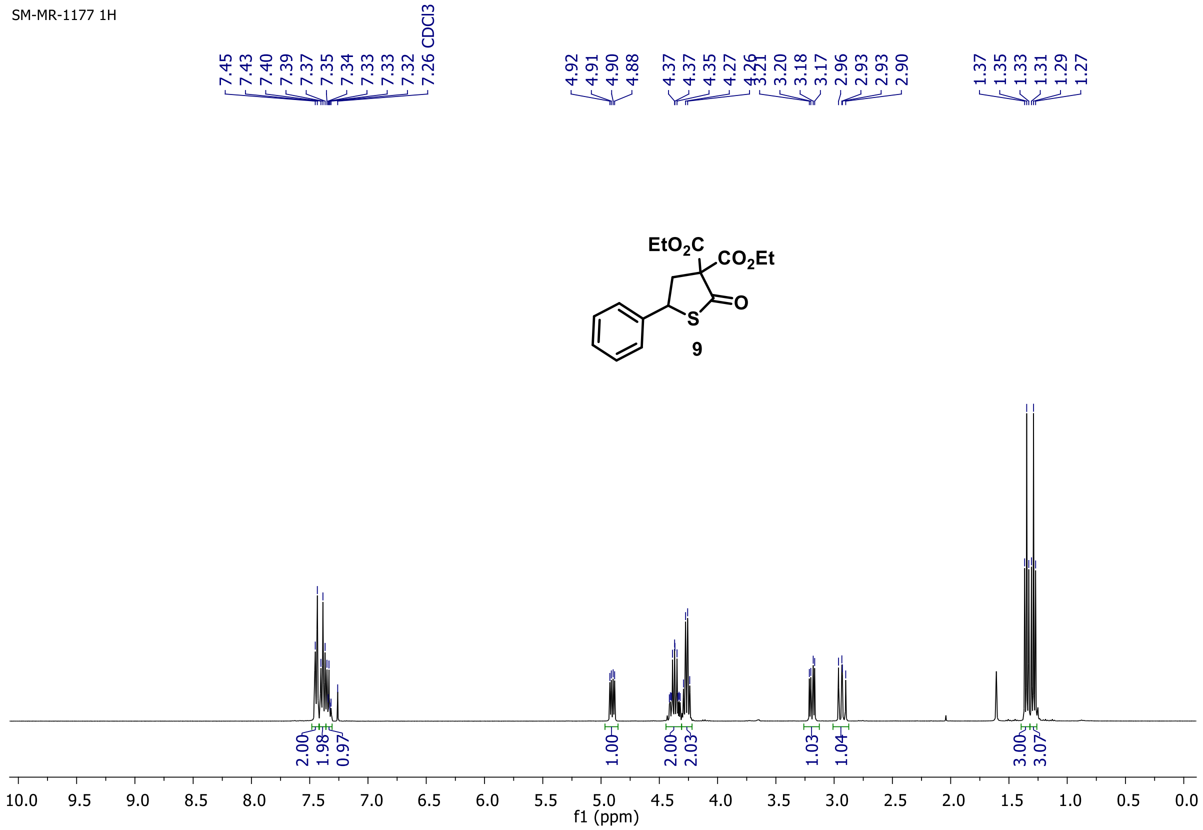


$^{13}C\{^{1}H\}$ NMR of **9** (101 MHz, $CDCl_3$):

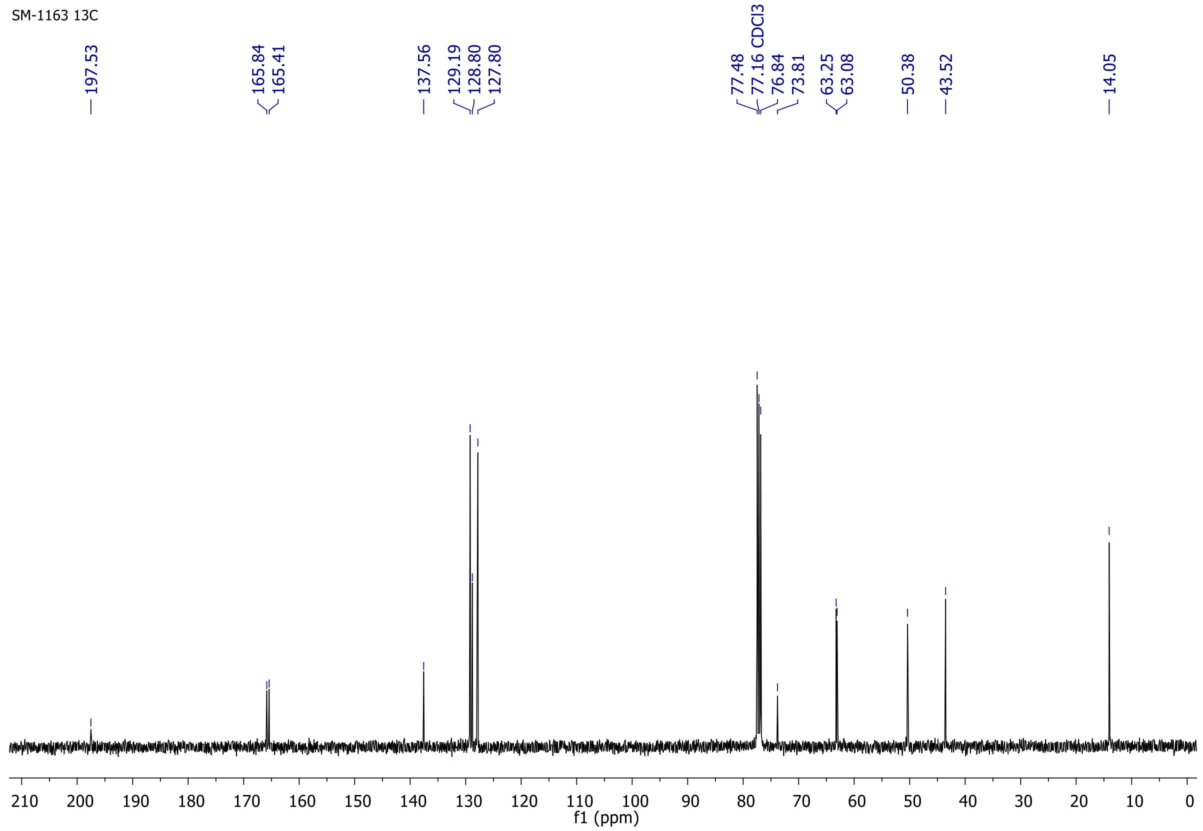

<sup></sup>

$^{1}$H NMR of **10** (400 MHz, $CDCl_3$):

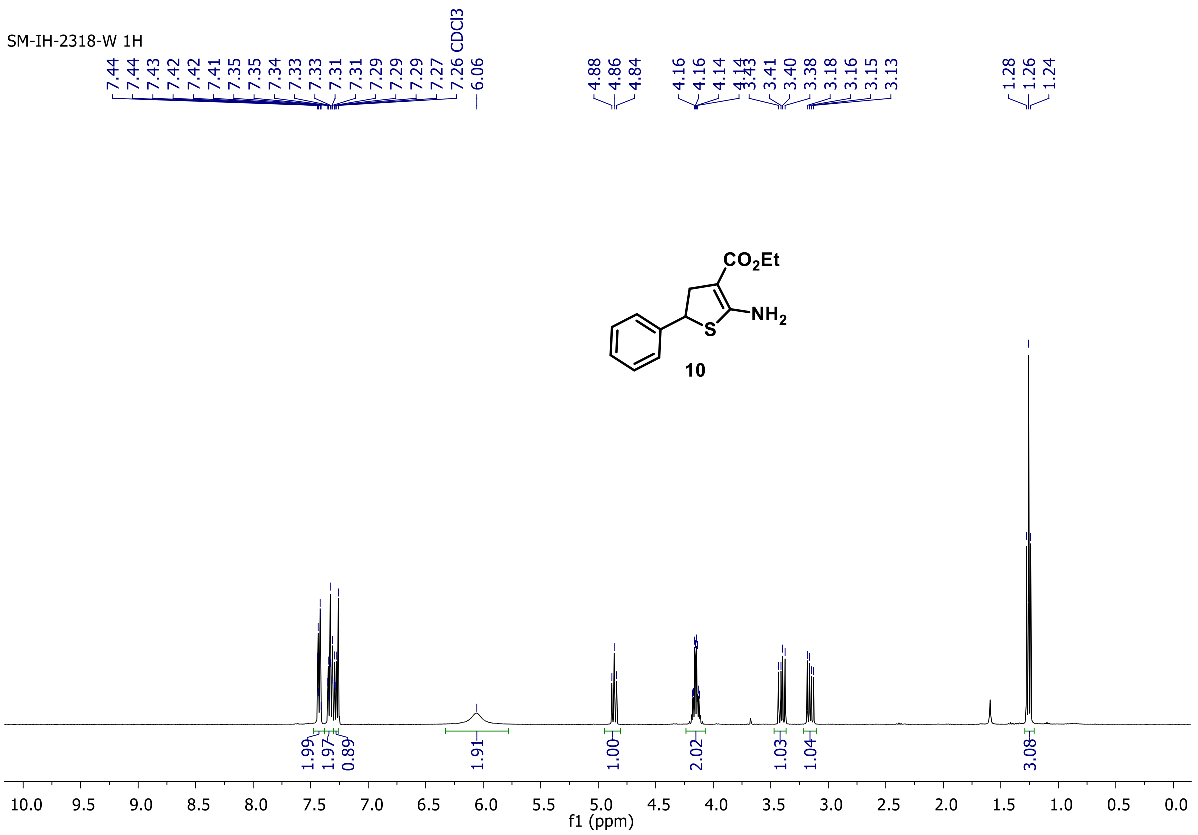


$^{13}$C{$^{1}$H} NMR of **10** (101 MHz, $CDCl_3$):

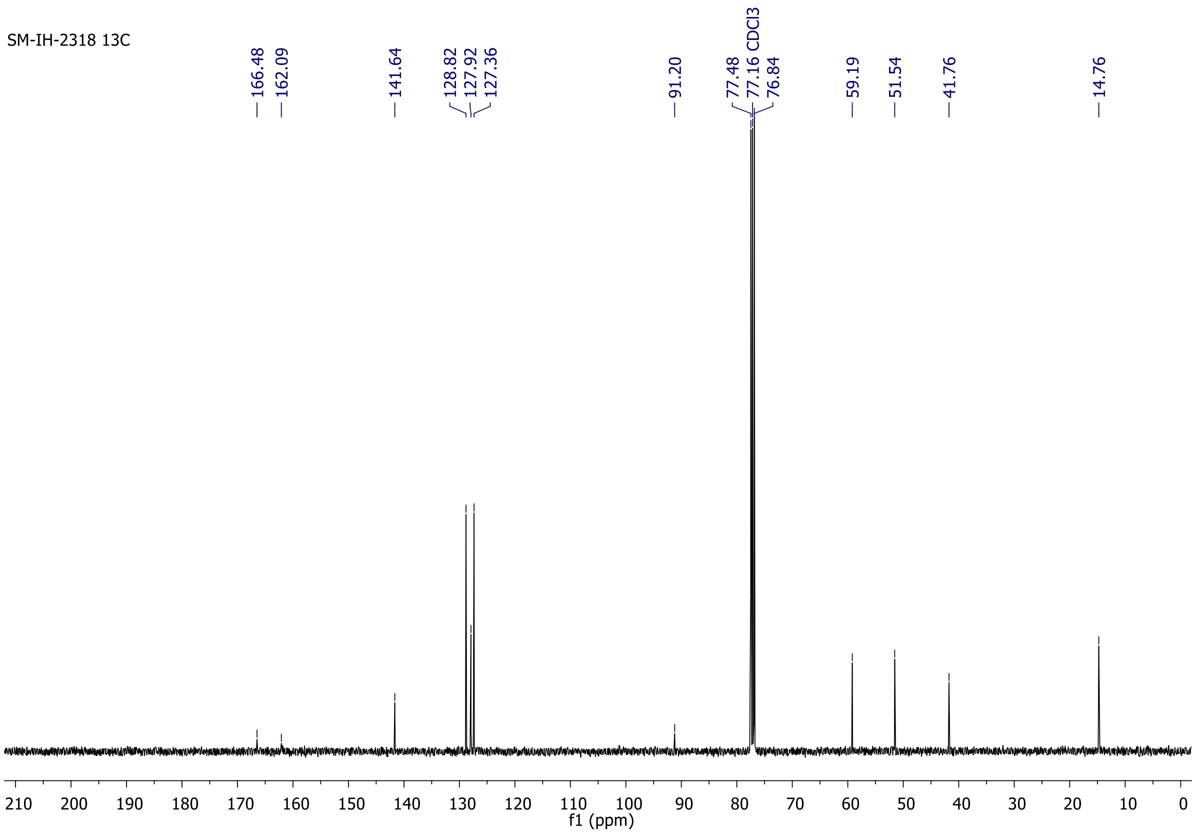

$^{1}H$ NMR of **11** (400 MHz, $CDCl_3$):

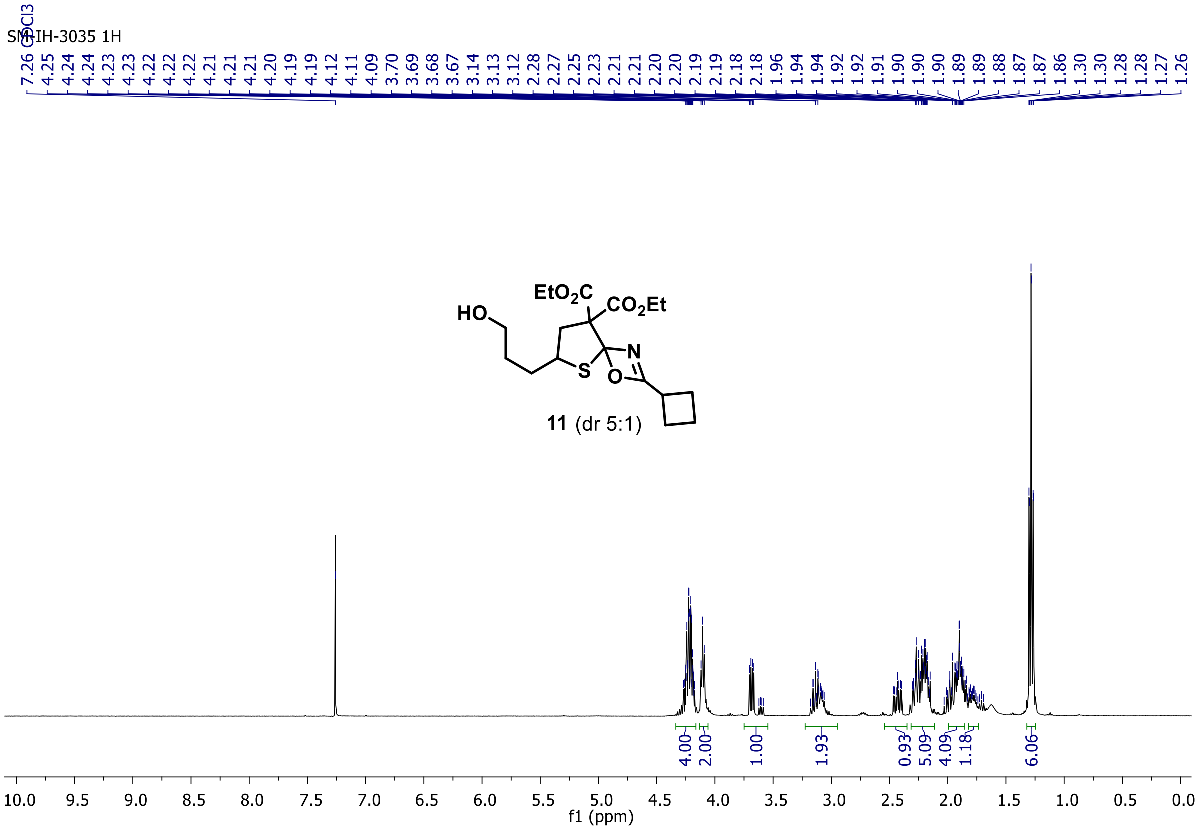


$^{13}C\{^{1}H\}$ NMR of **11** (101 MHz, $CDCl_3$):

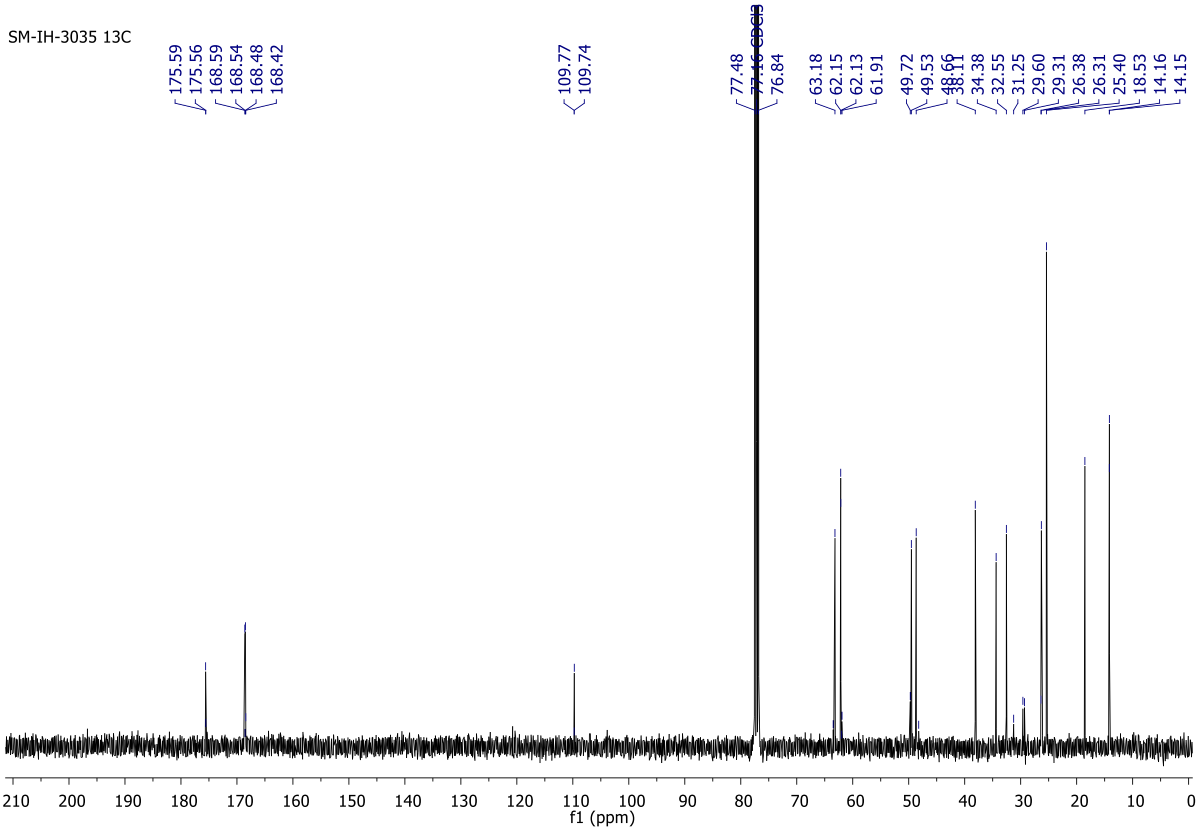

$^{1}H$ NMR of **12** (400 MHz, $CDCl_3$):

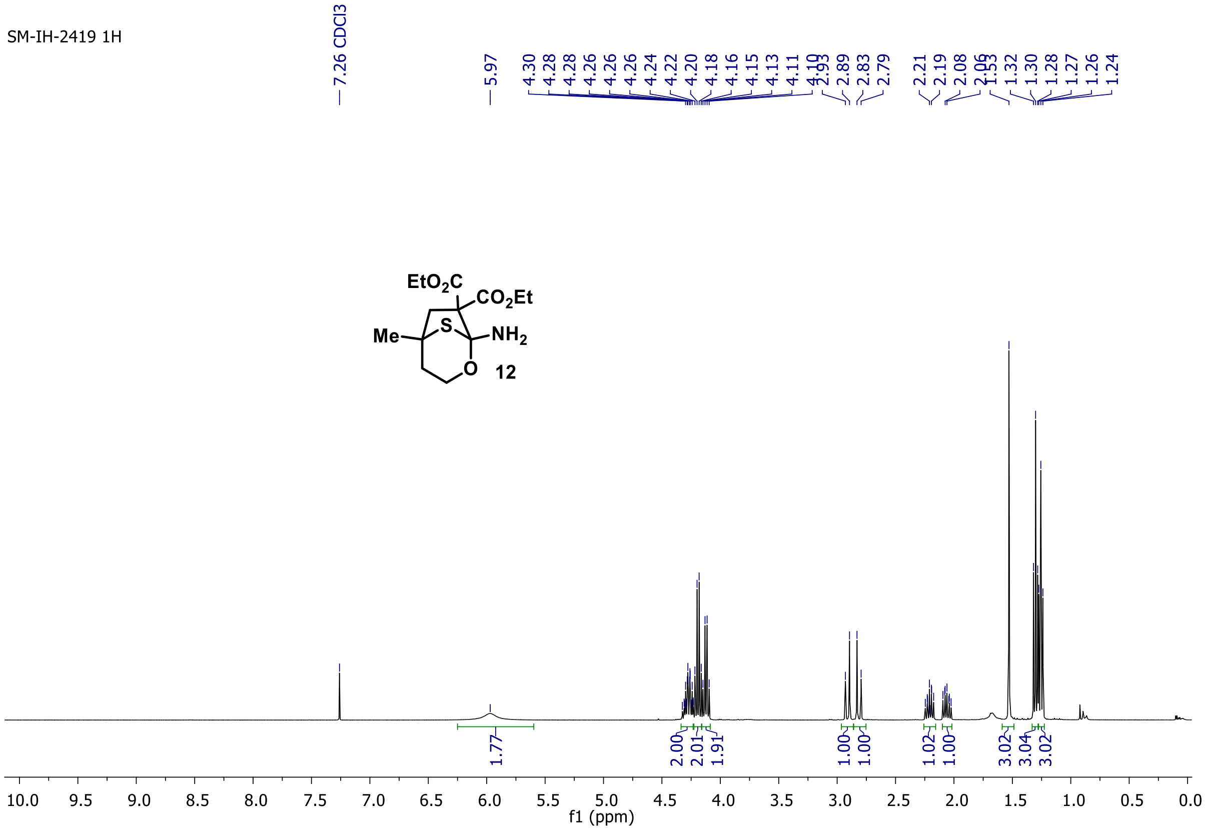


$^{13}C\{^{1}H\}$ NMR of **12** (101 MHz, $CDCl_3$):

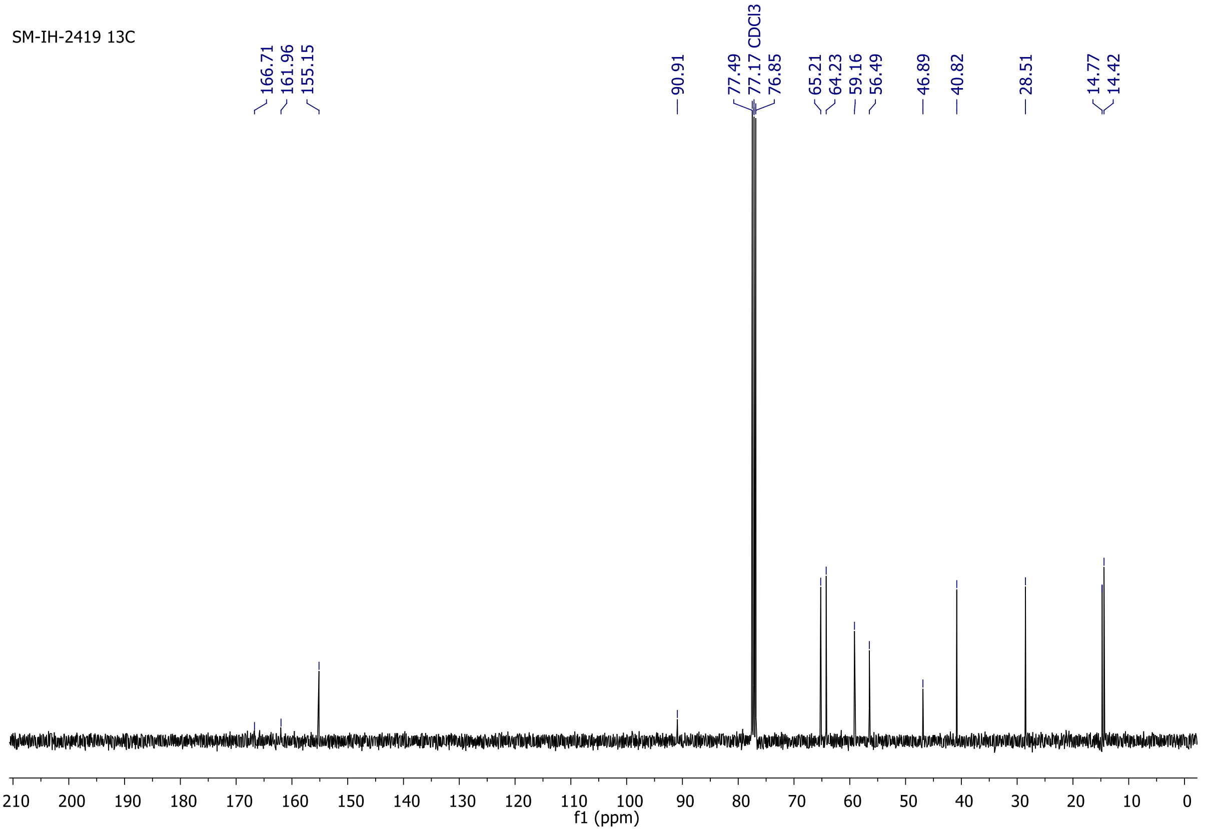

$^{1}H$ NMR of **13** (400 MHz, $CDCl_3$):

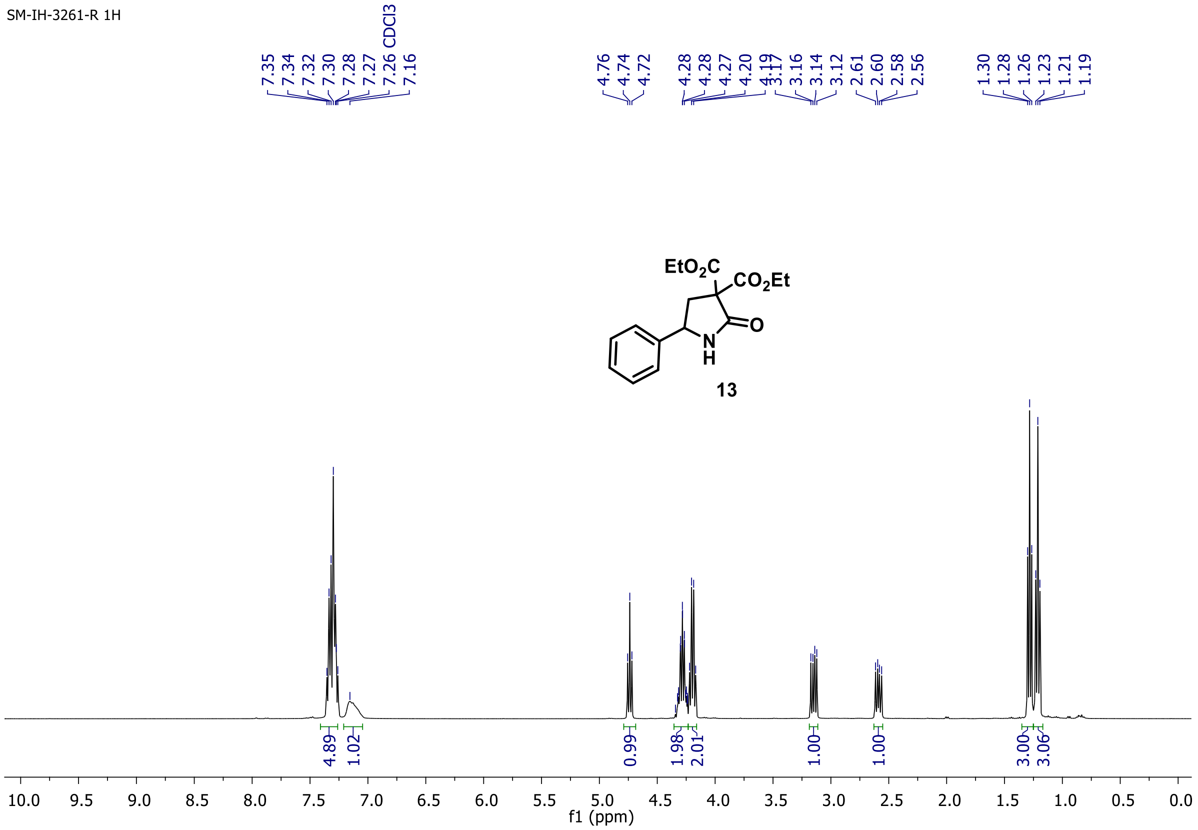


**13**

$^{13}C\{^{1}H\}$ NMR of **13** (101 MHz, $CDCl_3$):

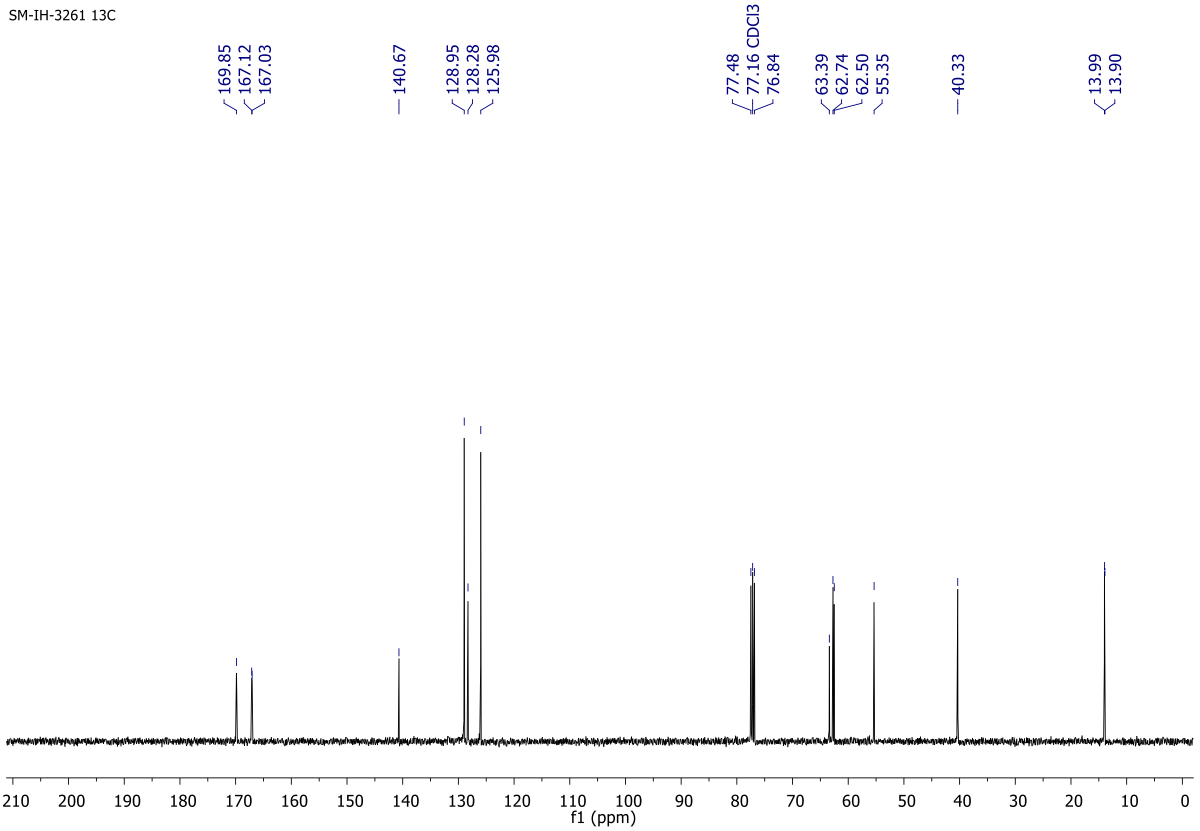

$^{1}$H NMR of **14** (400 MHz, $CDCl_3$):

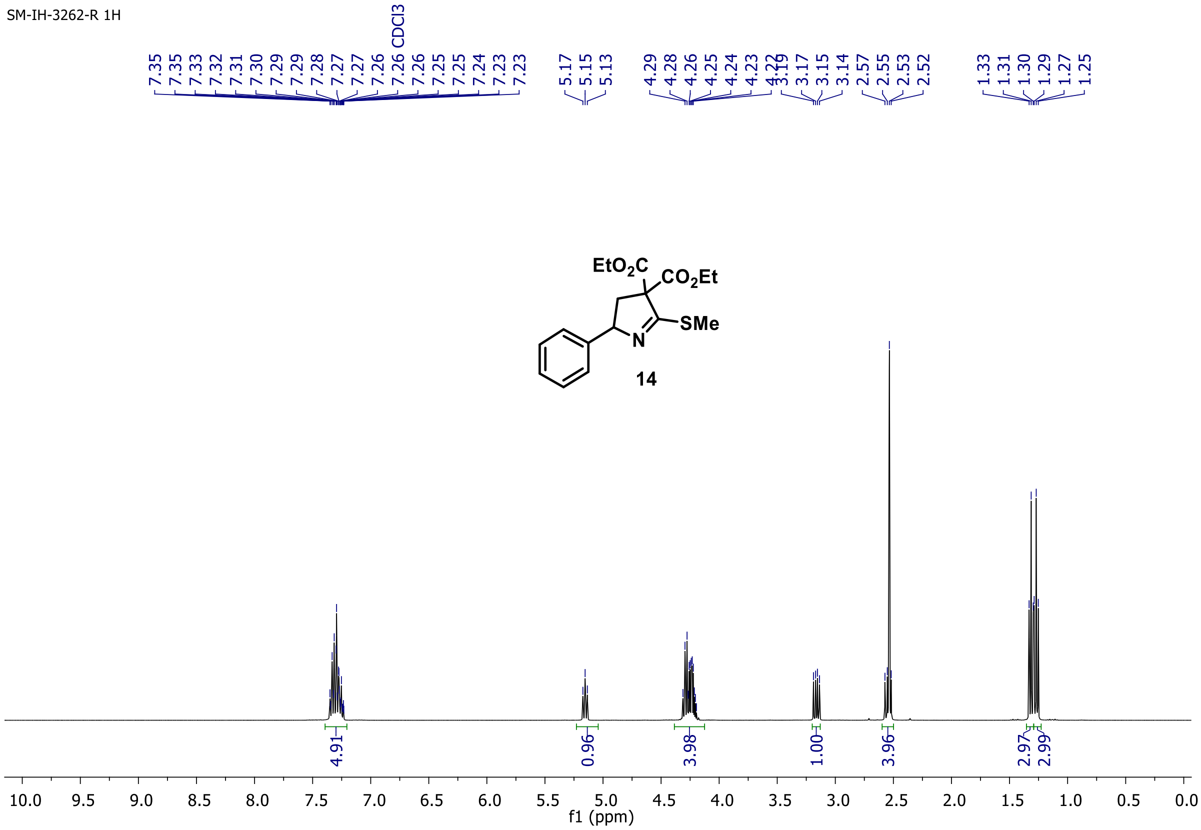


$^{13}$C{$^{1}$H} NMR of **14** (101 MHz, $CDCl_3$):

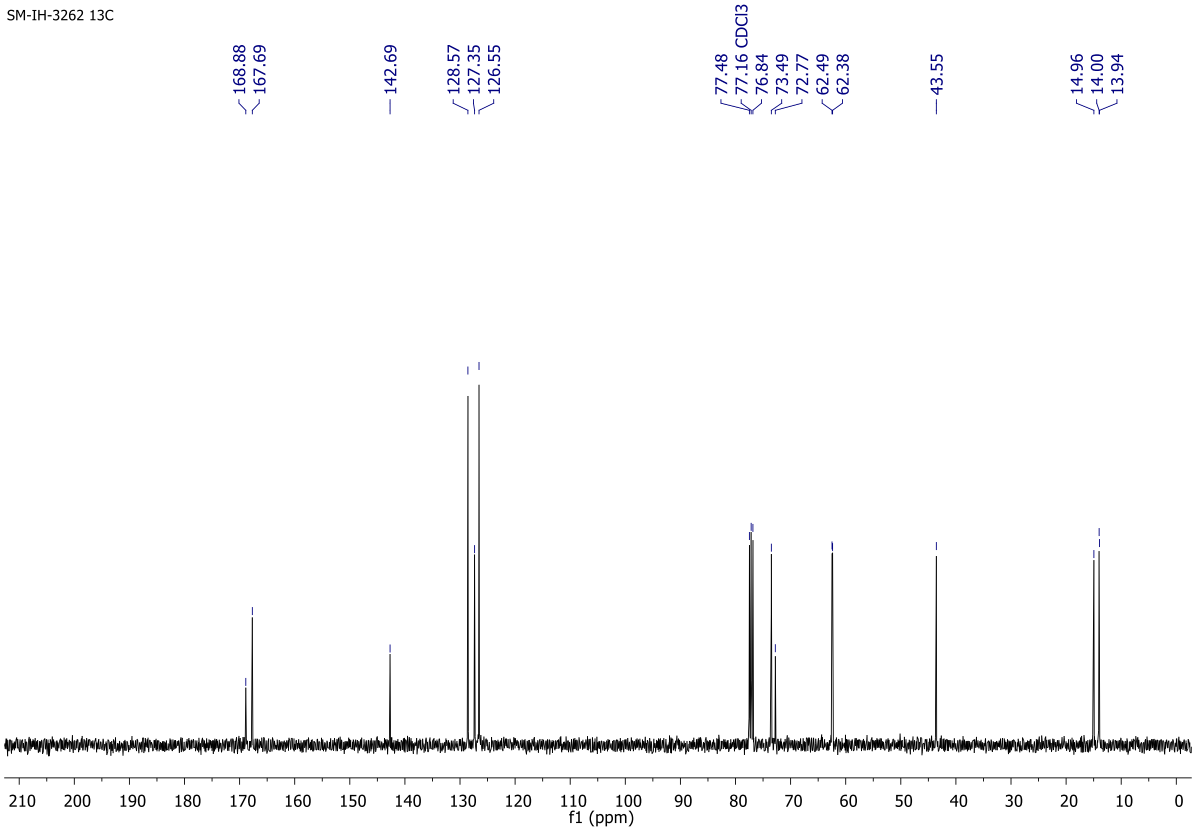

$^{1}$H NMR of **15** (400 MHz, $CDCl_3$):

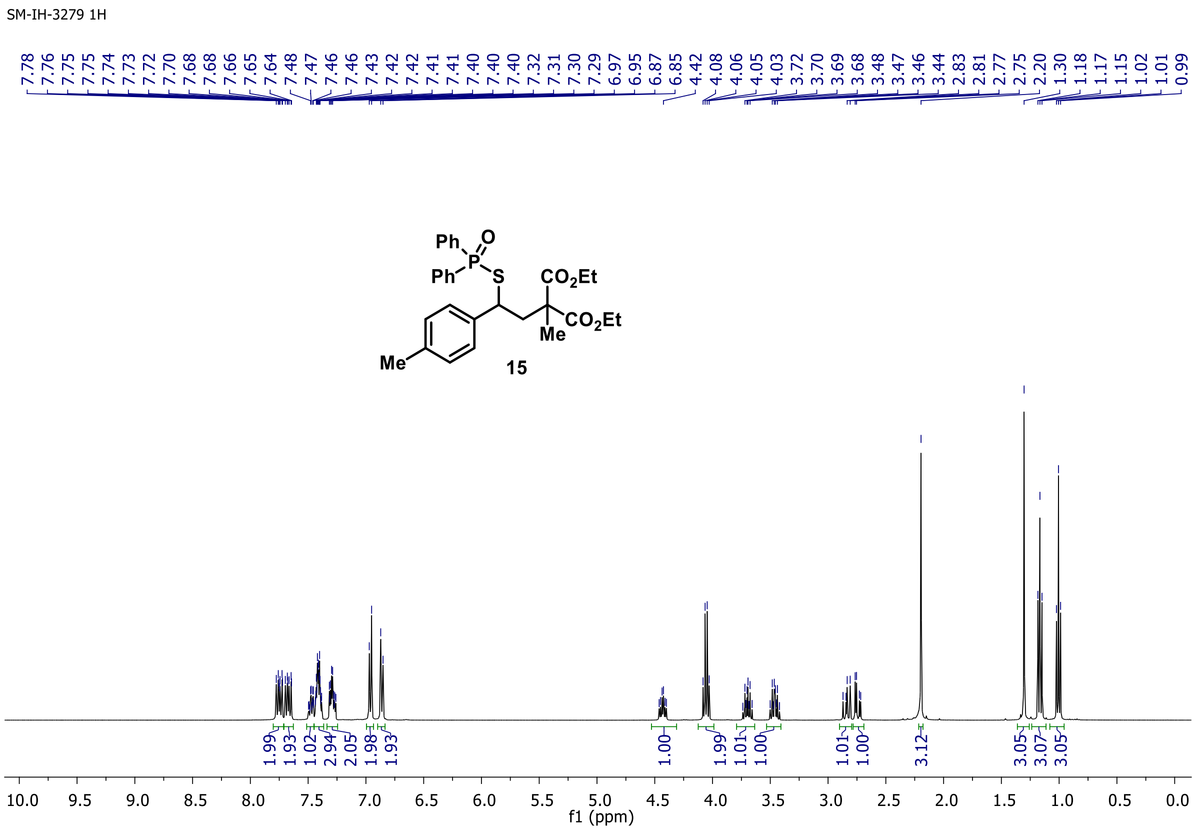


$^{13}C\{^{1}H\}$ NMR of **15** (101 MHz, $CDCl_3$):

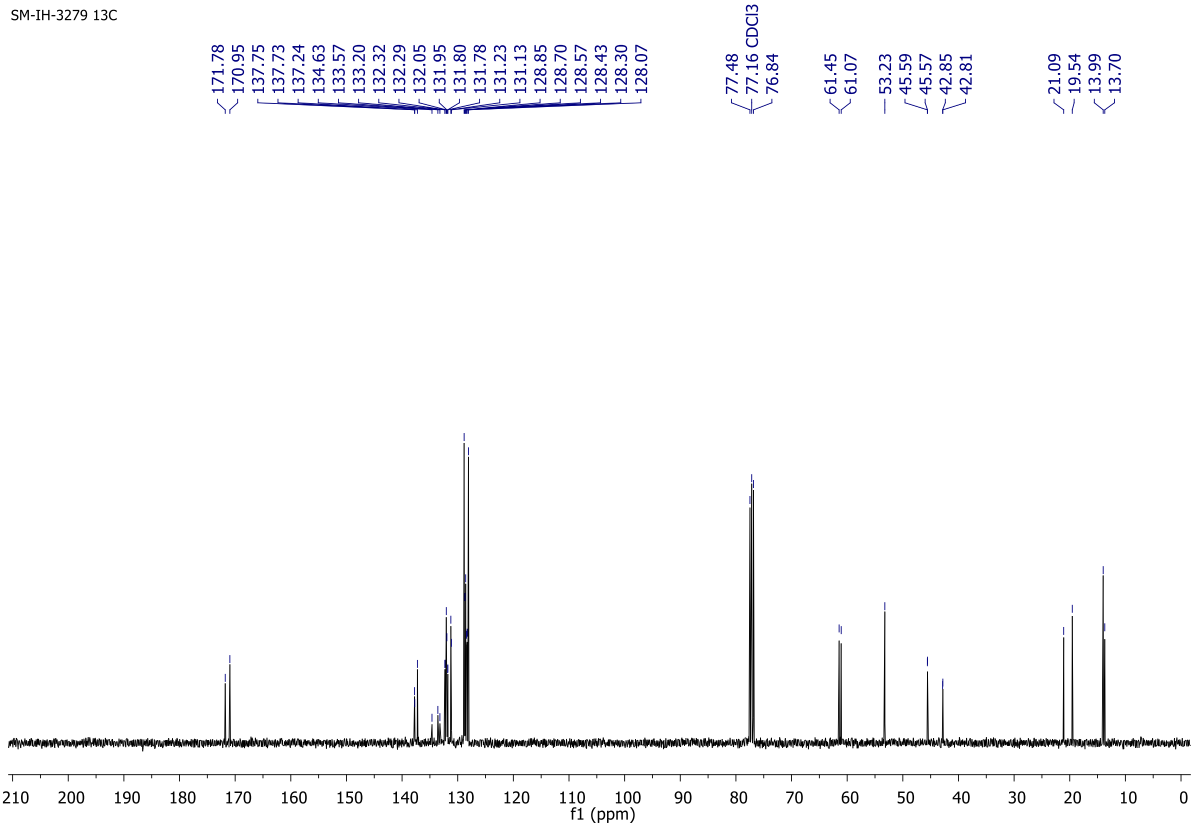

$^{31}$P NMR of **15** (162 MHz, $CDCl_3$):

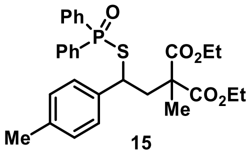


**15**

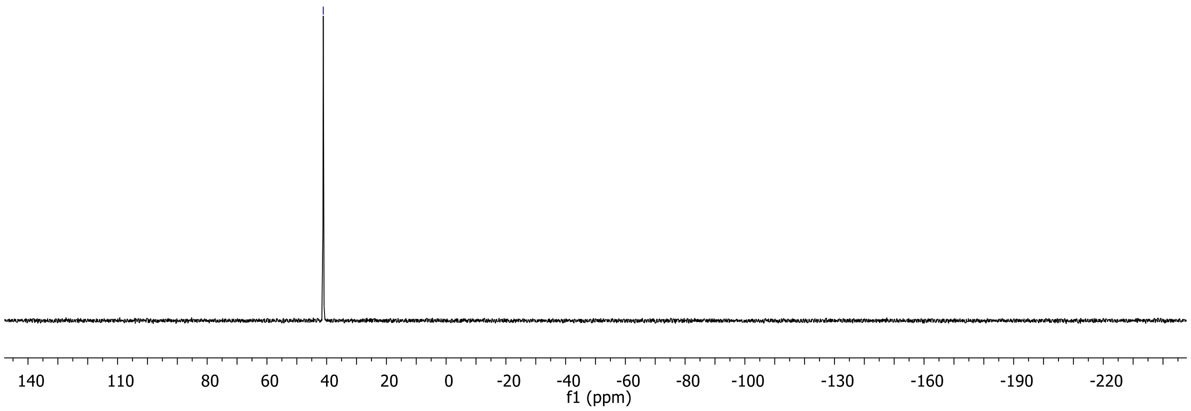

$^{1}H$ NMR of **16** (400 MHz, $CDCl_3$):

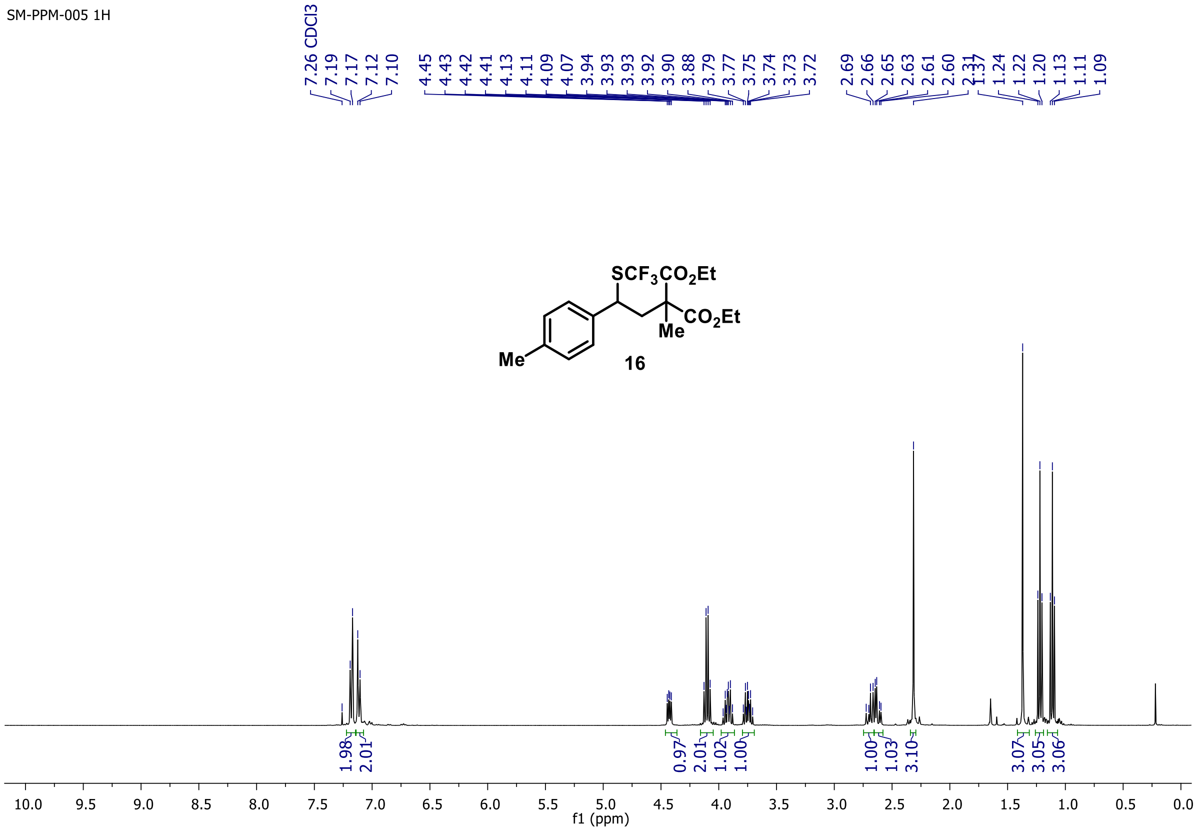


$^{13}C\{^{1}H\}$ NMR of **16** (101 MHz, $CDCl_3$):

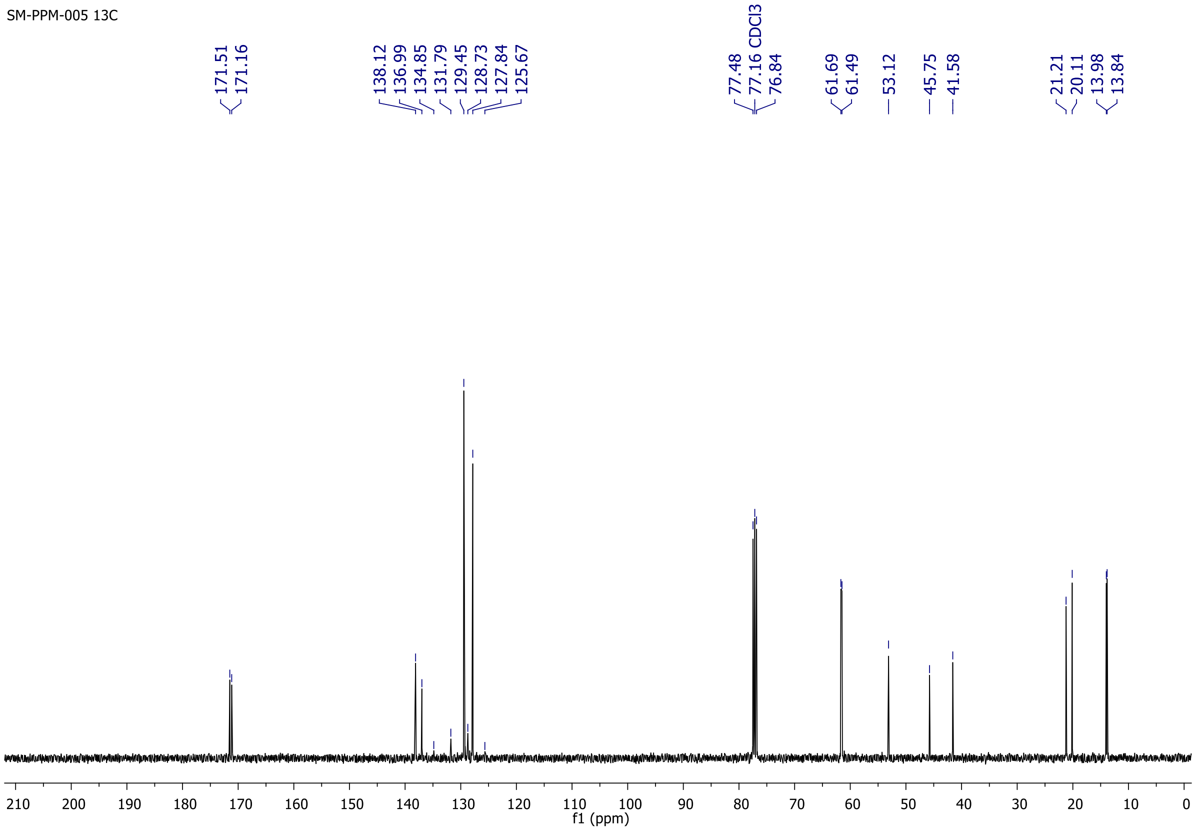

$^{19}$F NMR of **16** (377 MHz, $CDCl_3$):

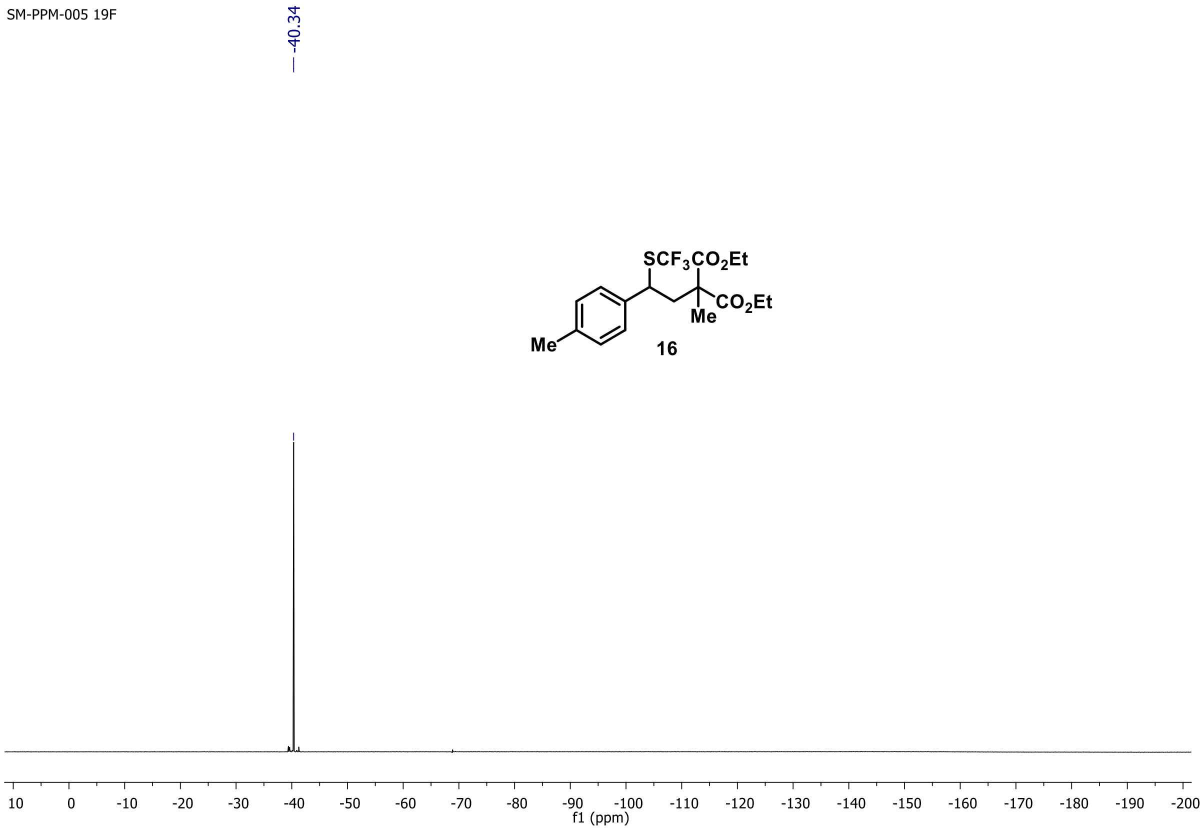

$^{1}H$ NMR of **17** (400 MHz, $CDCl_3$):

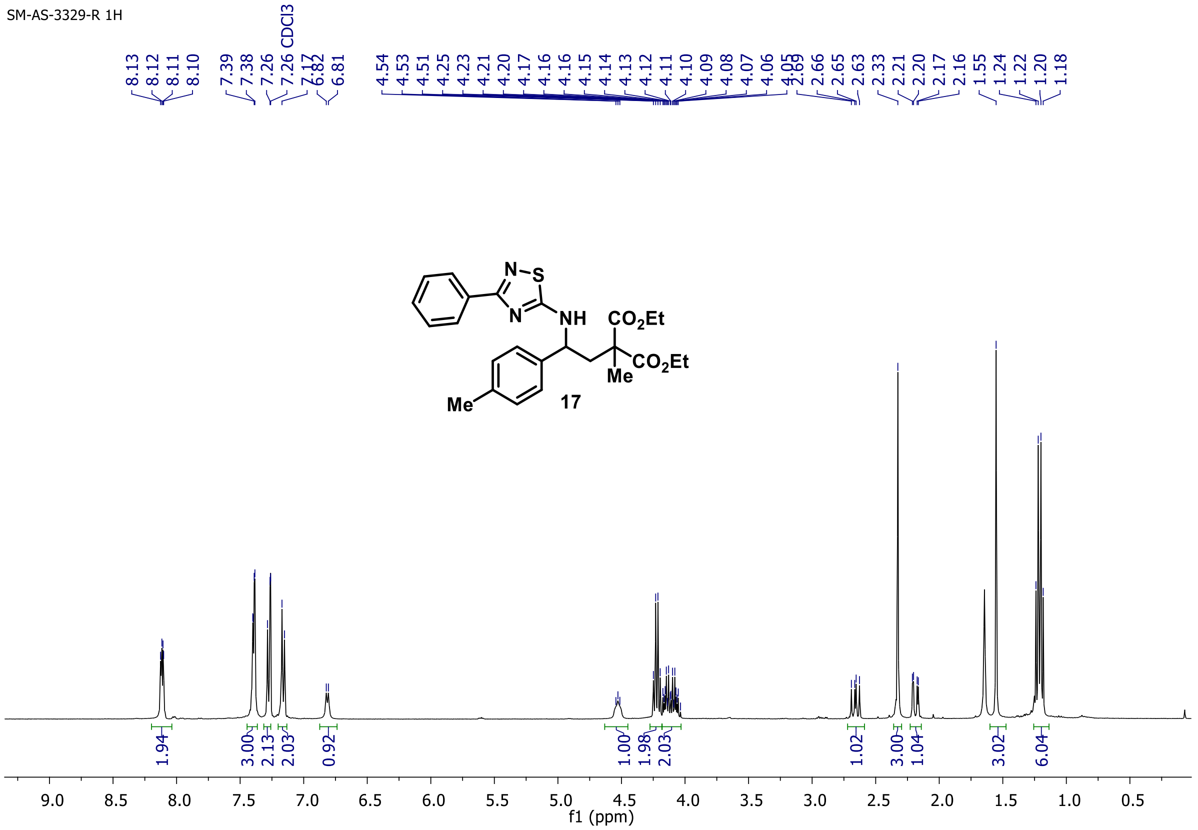


$^{13}C\{^{1}H\}$ NMR of **17** (101 MHz, $CDCl_3$):

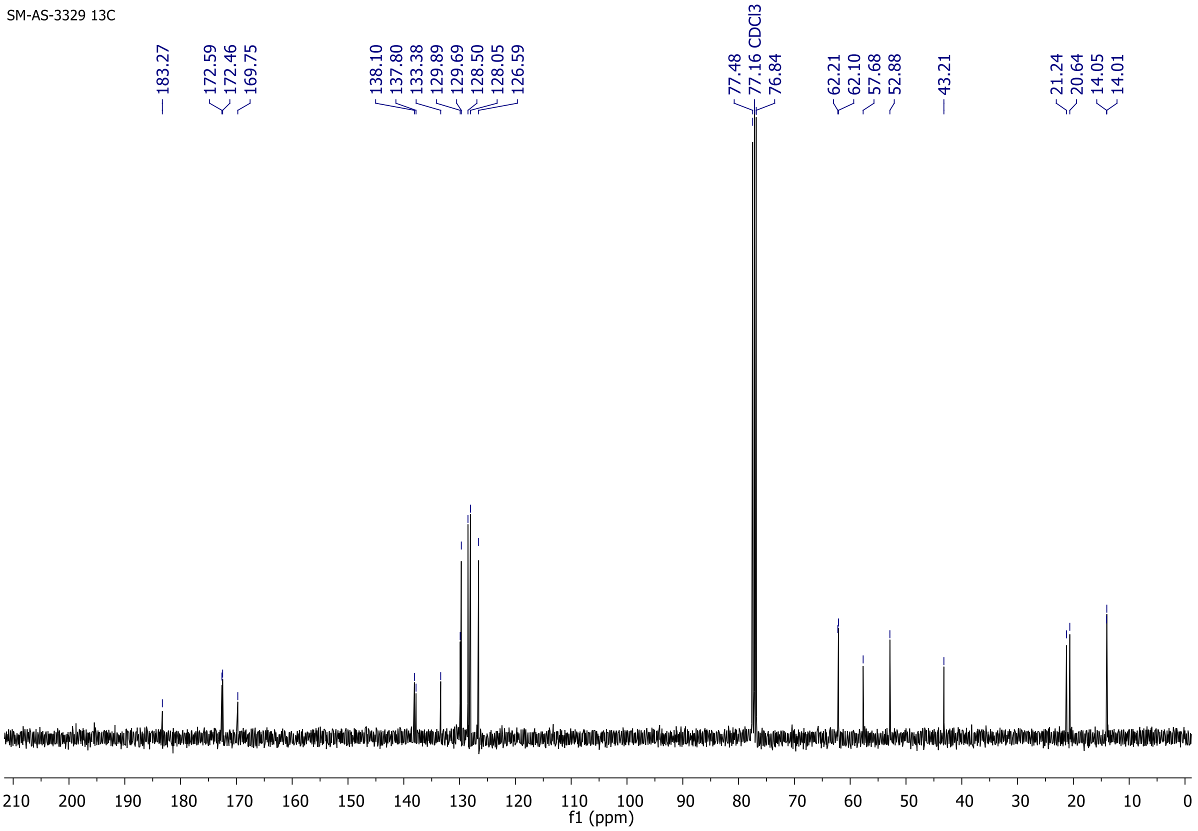

$^{1}H$ NMR of **18** (400 MHz, $CDCl_3$):

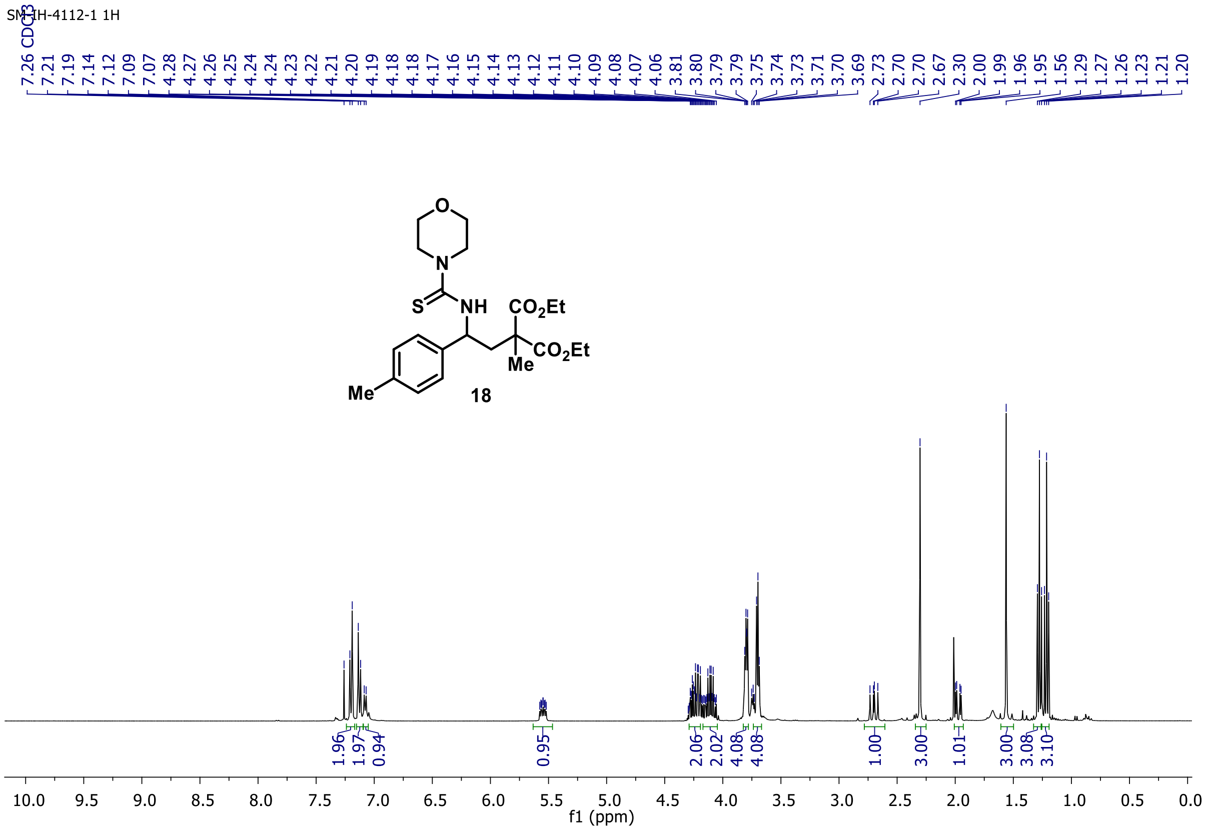


$^{13}C\{^{1}H\}$ NMR of **18** (101 MHz, $CDCl_3$):

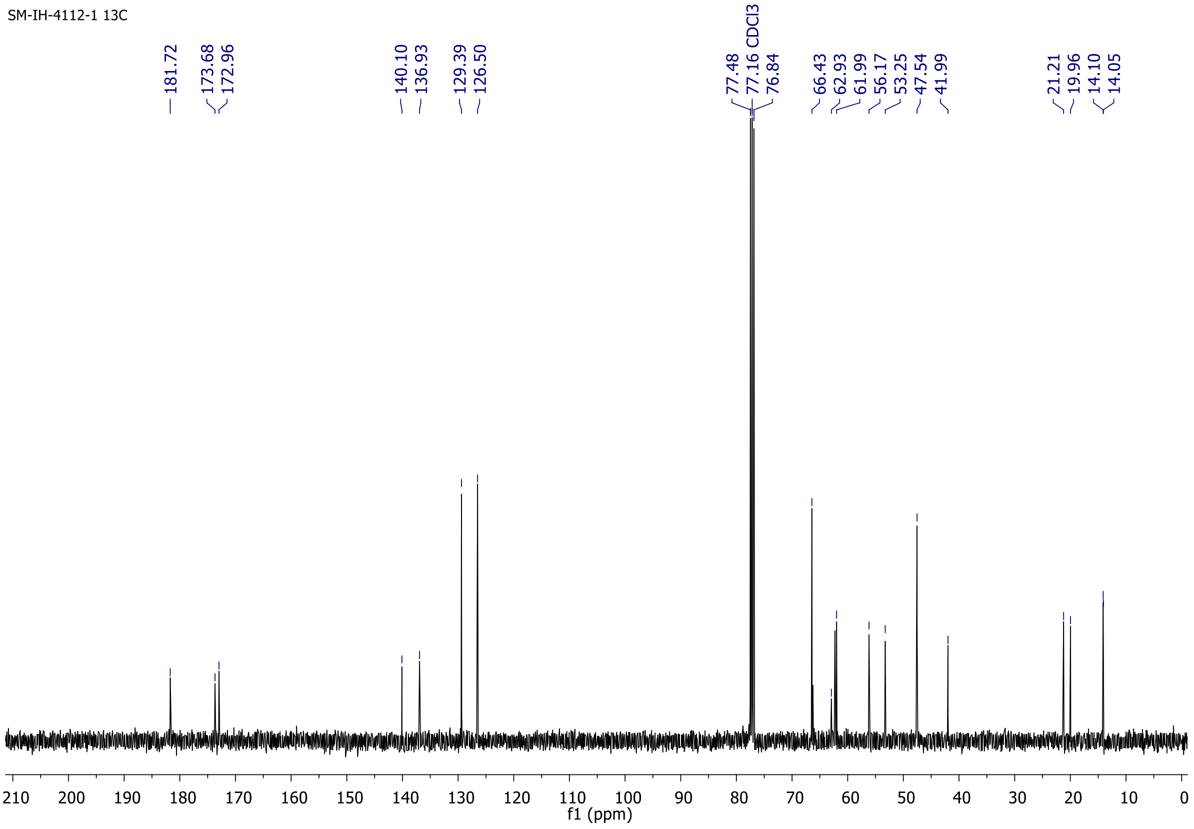

$^{1}$H NMR of **19** (400 MHz, $CDCl_3$):

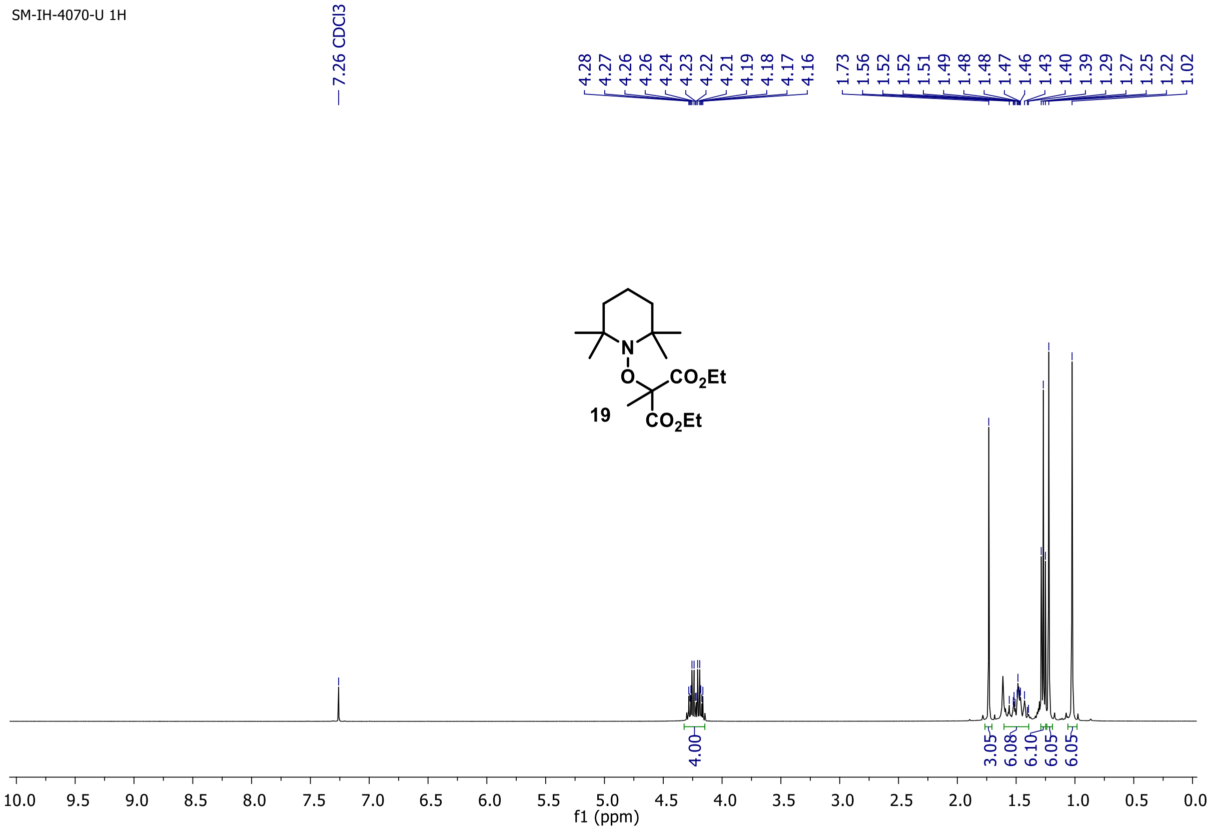


$^{13}$C{$^{1}$H} NMR of **19** (101 MHz, $CDCl_3$):

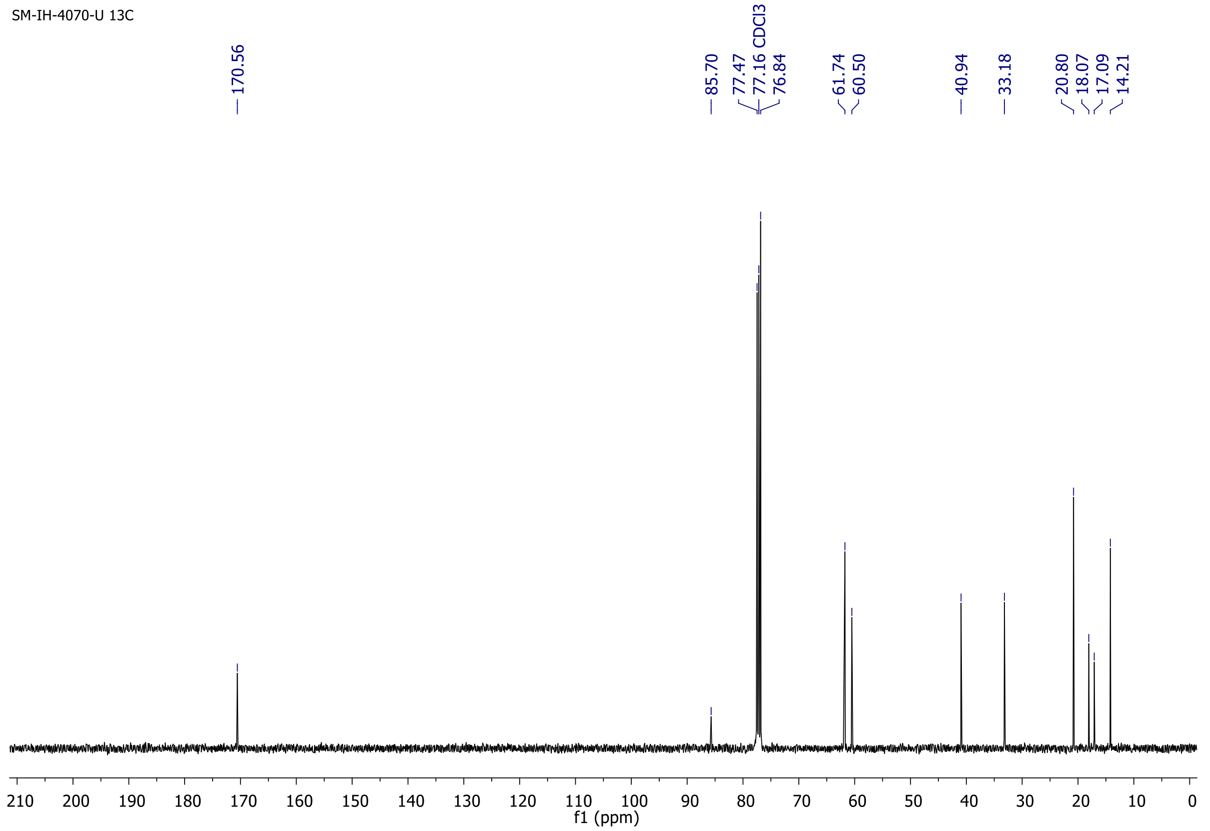

$^{1}H$ NMR of **19’** (400 MHz, $CDCl_3$):

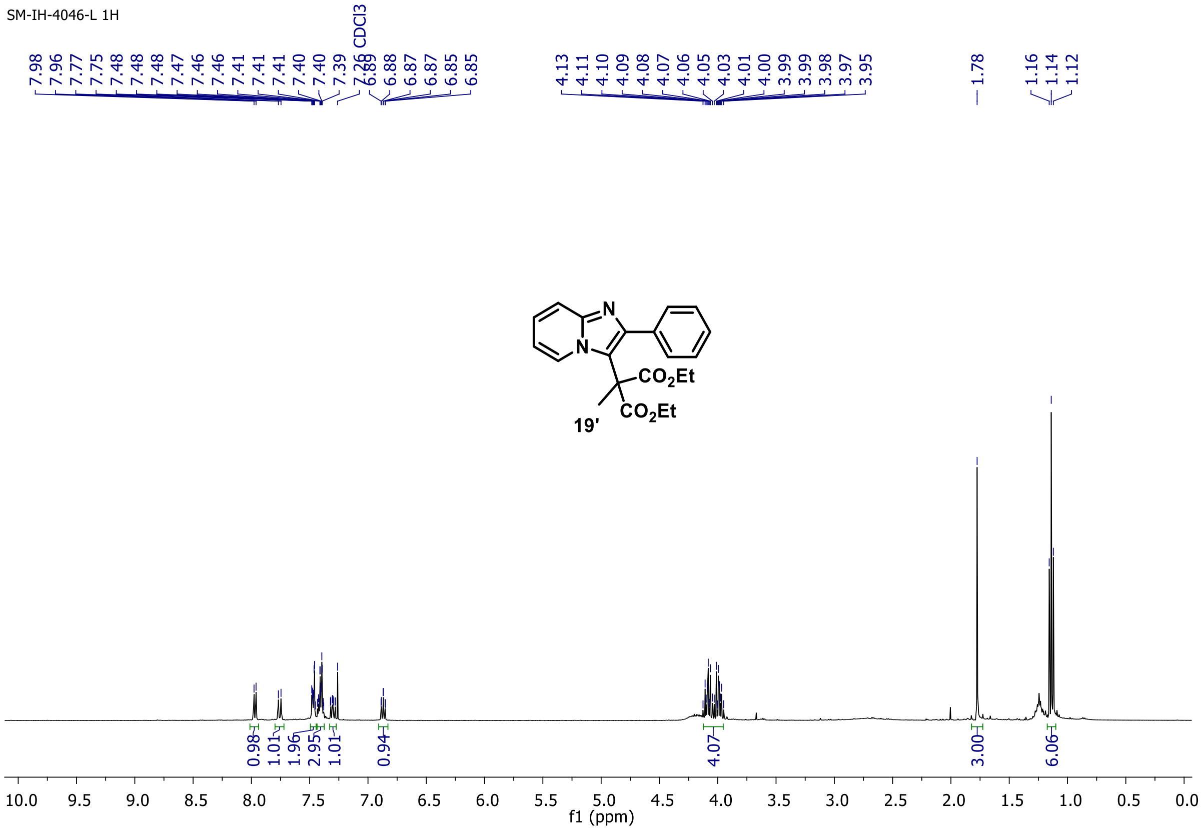


$^{13}C\{^{1}H\}$ NMR of **19’** (101 MHz, $CDCl_3$):

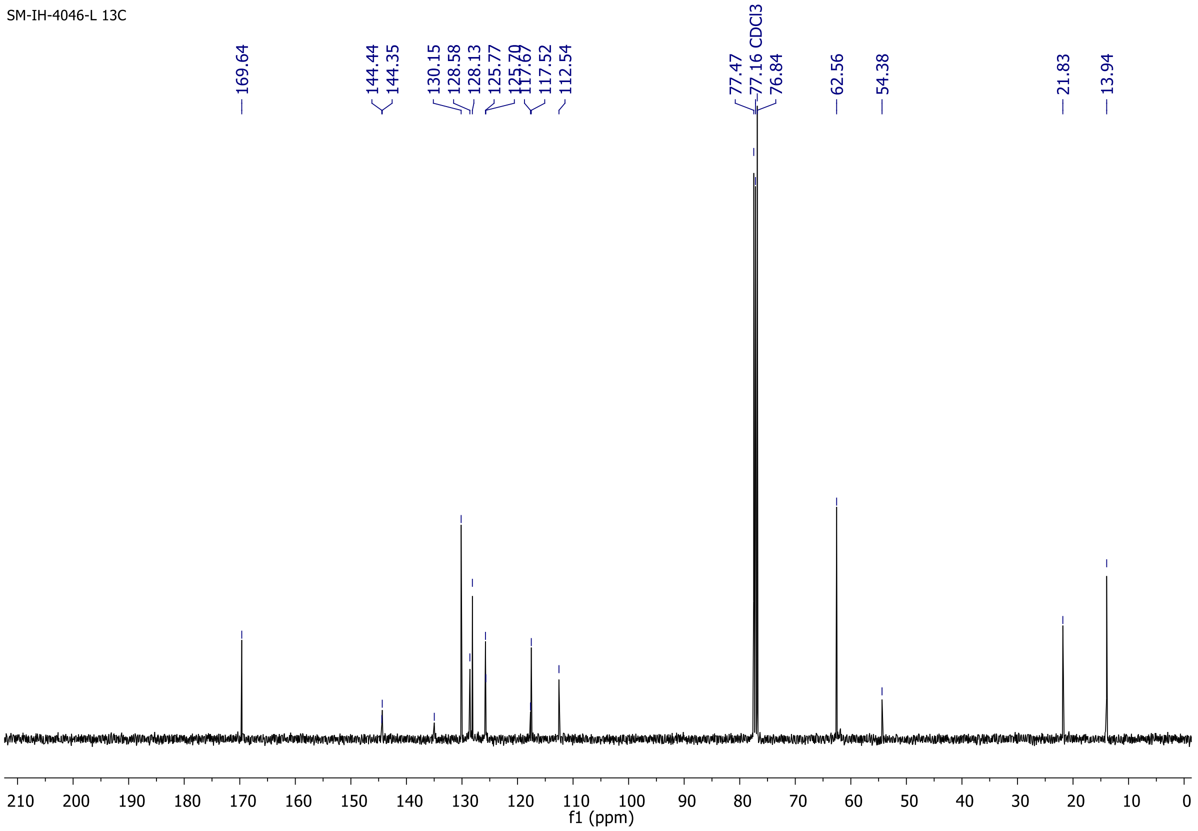

$^{1}H$ NMR of **21’** (400 MHz, $CDCl_3$):

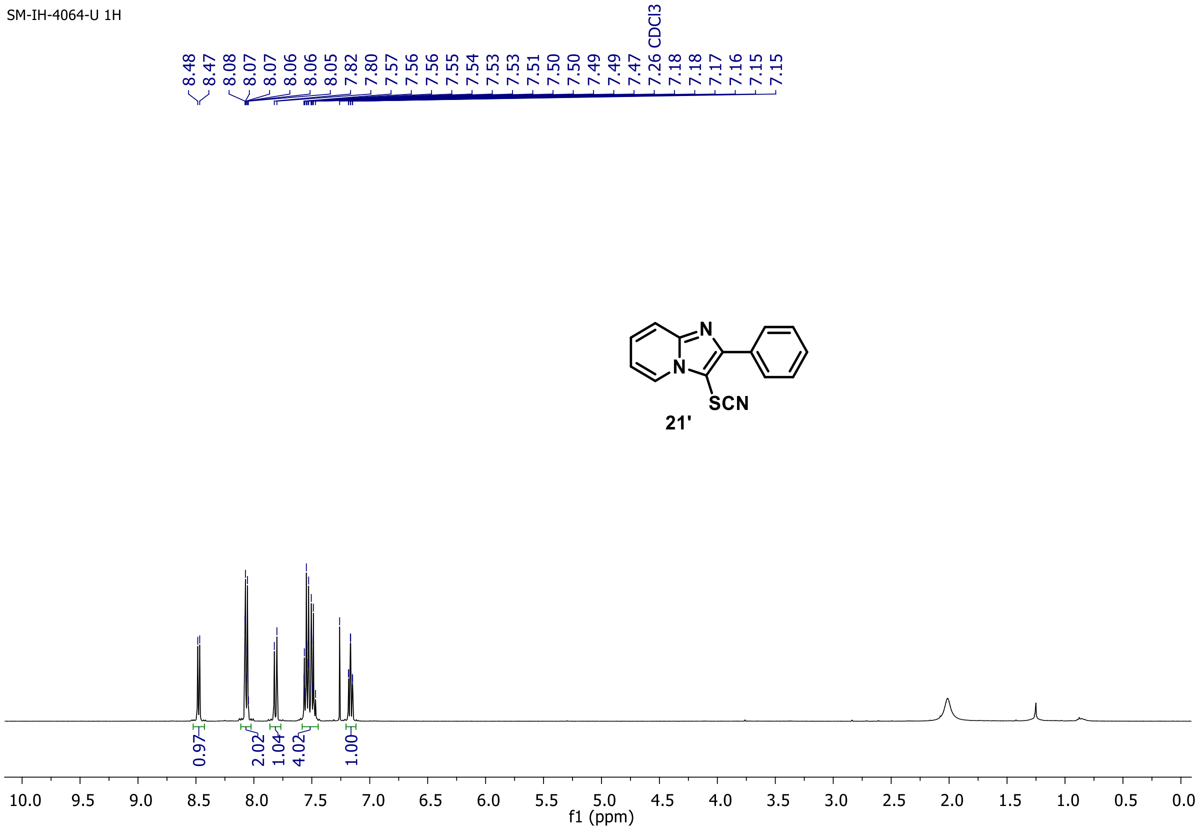


$^{13}C\{^{1}H\}$ NMR of **21’** (101 MHz, $CDCl_3$):

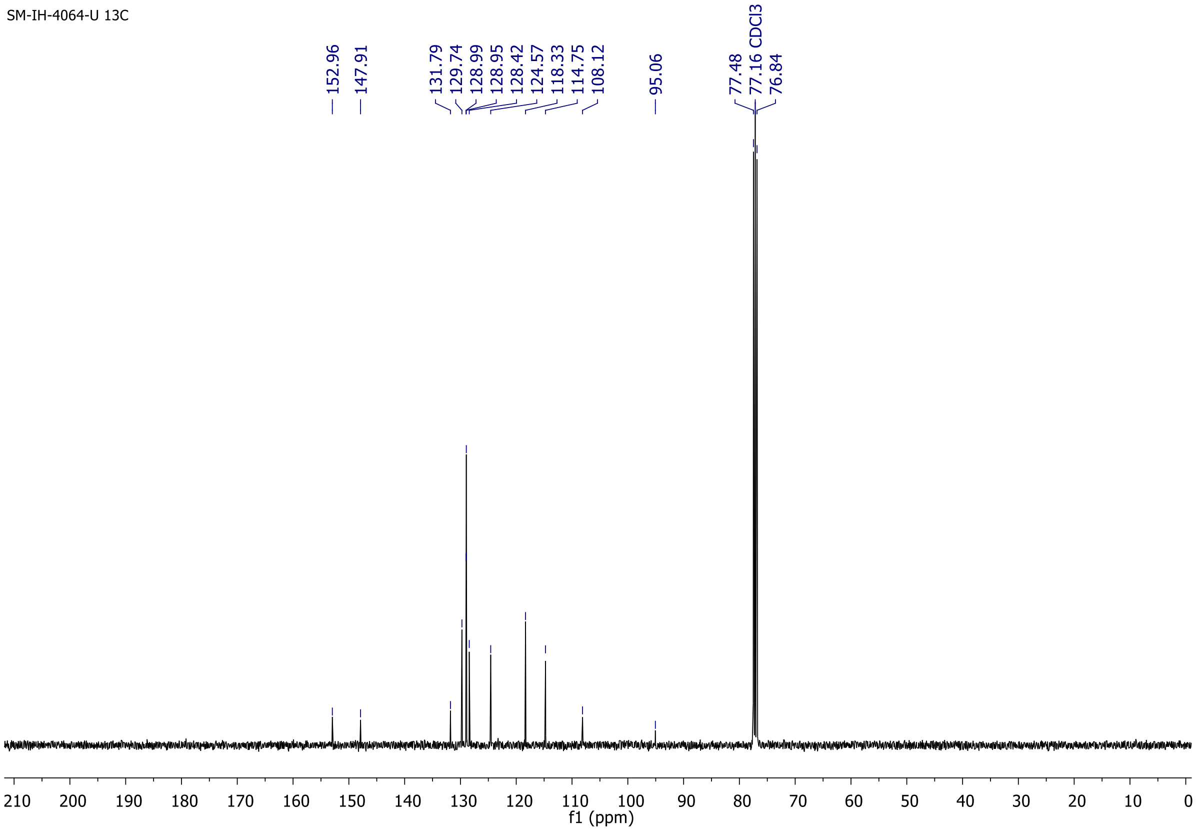

$^1H$ NMR of **23** (400 MHz, $CDCl_3$):

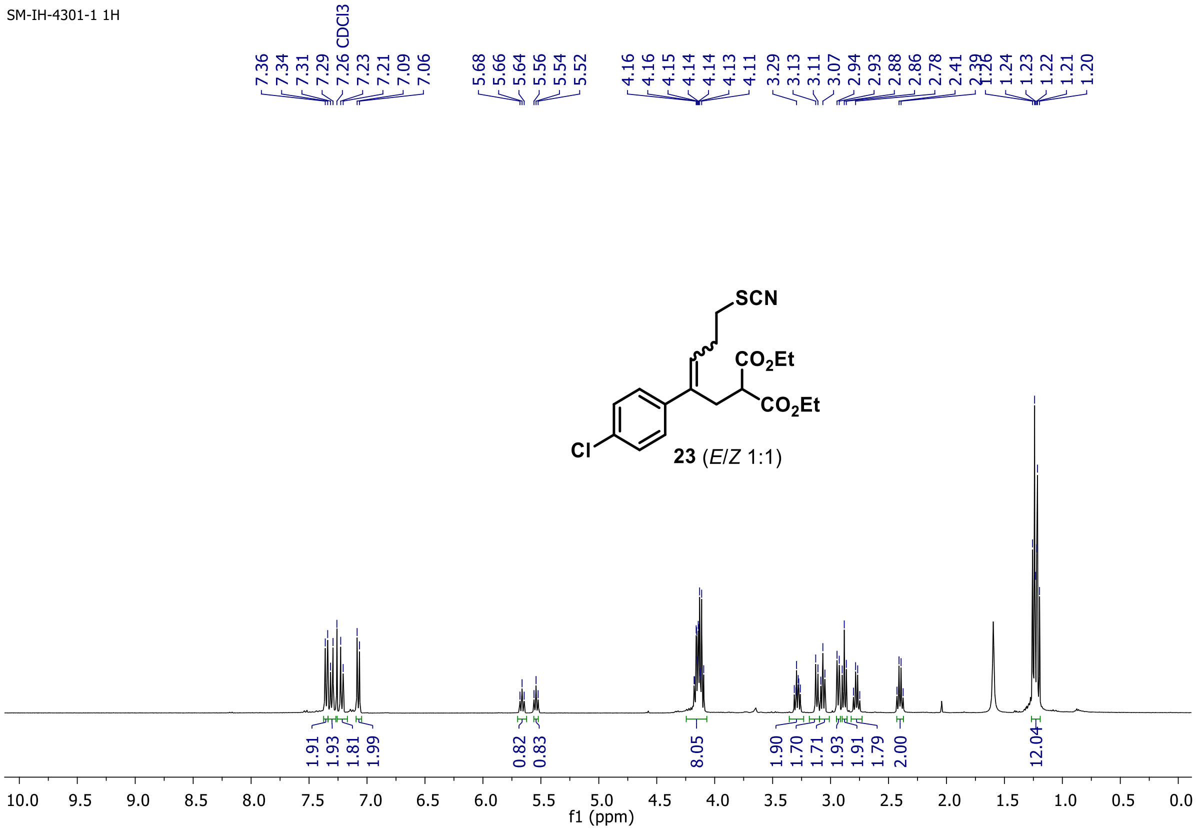


$^{13}C\{^1H\}$ NMR of **23** (101 MHz, $CDCl_3$):

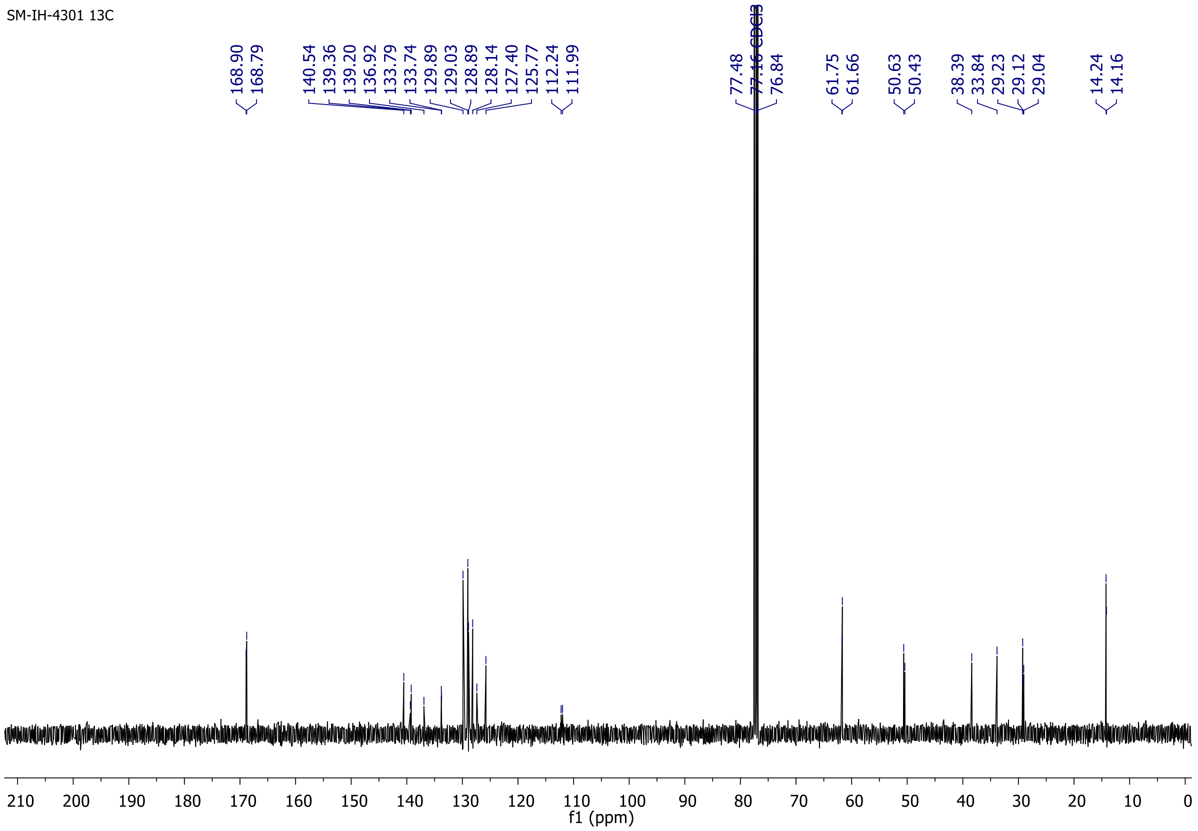

$^{1}H$ NMR of **25** (400 MHz, $CDCl_3$):

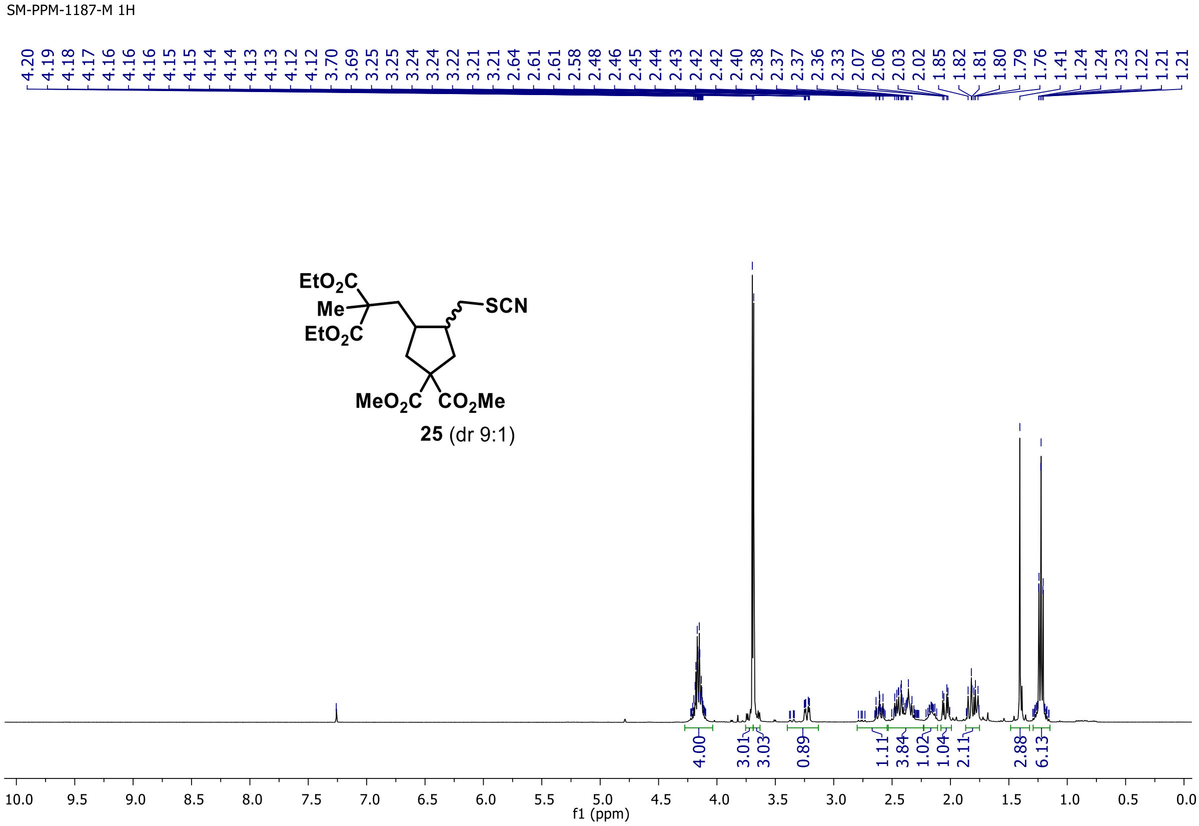


$^{13}C\{^{1}H\}$ NMR of **25** (101 MHz, $CDCl_3$):

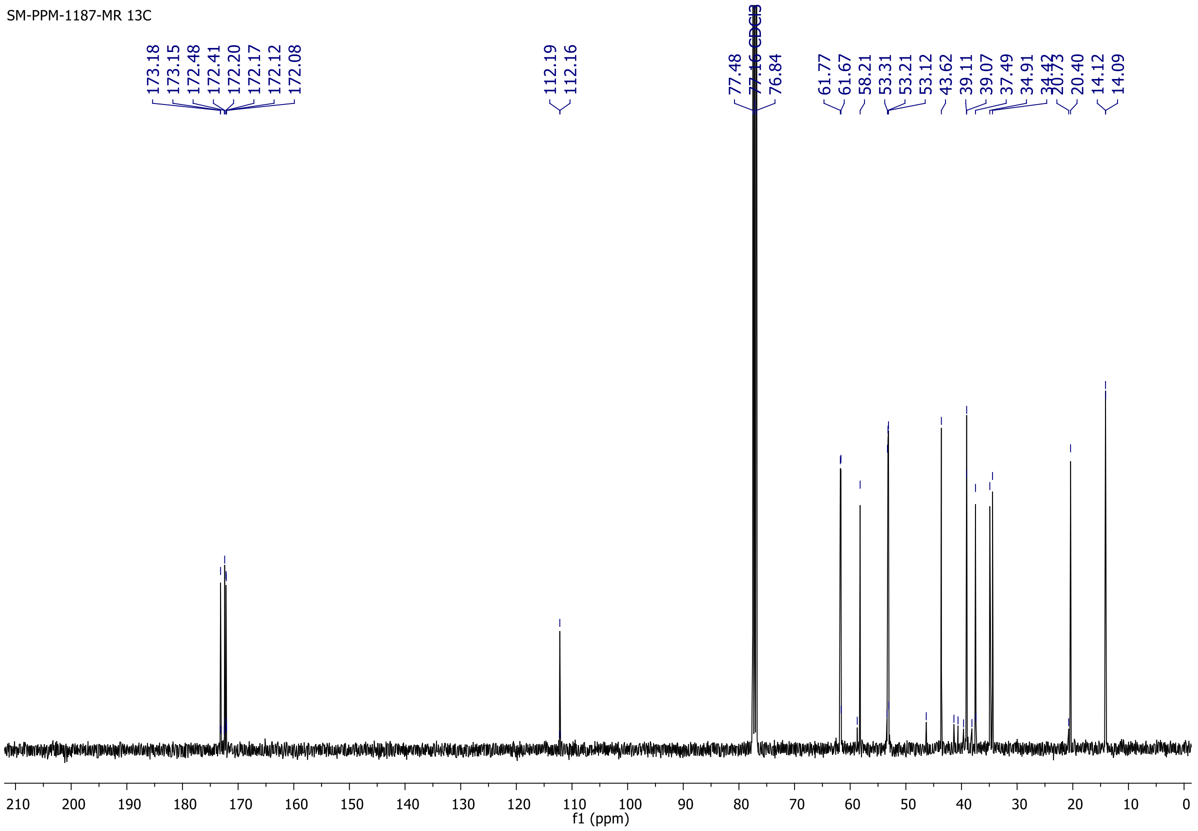

$^{1}$H NMR of **26a** (400 MHz, $CDCl_3$):

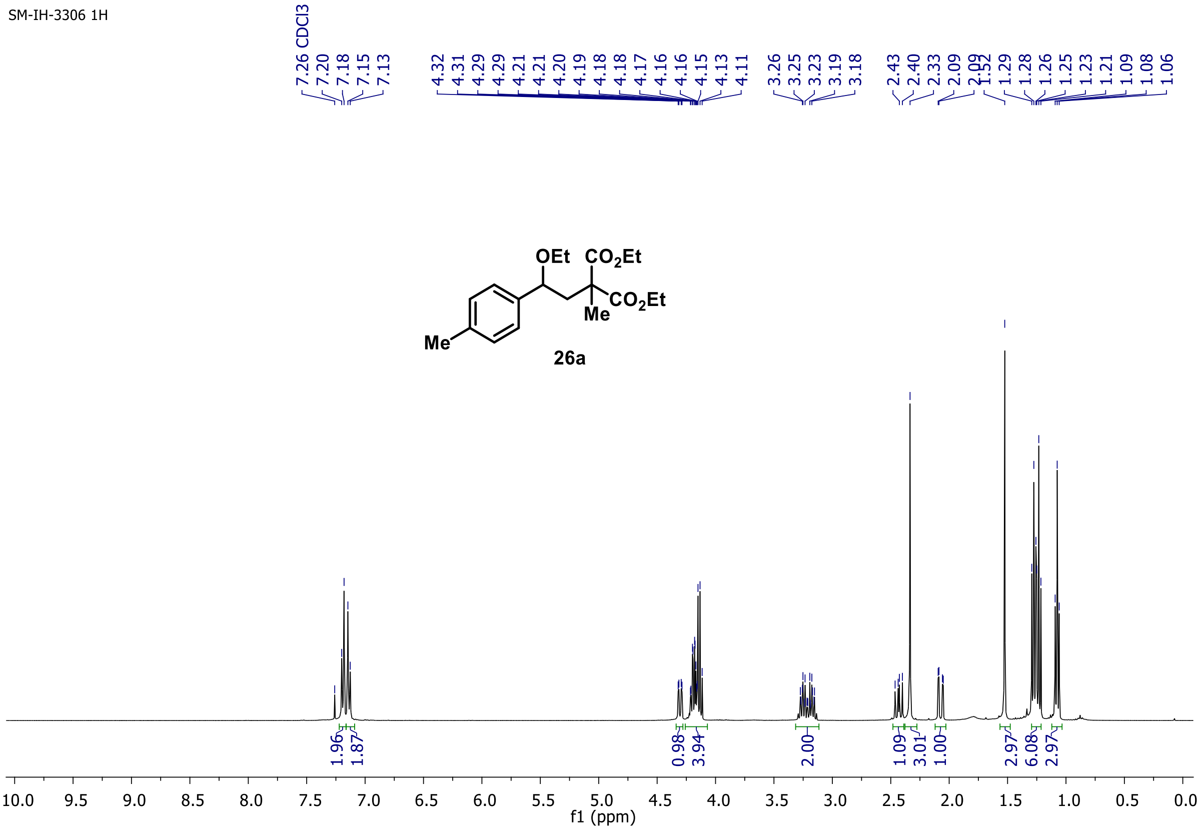


$^{13}$C{$^{1}$H} NMR of **26a** (101 MHz, $CDCl_3$):

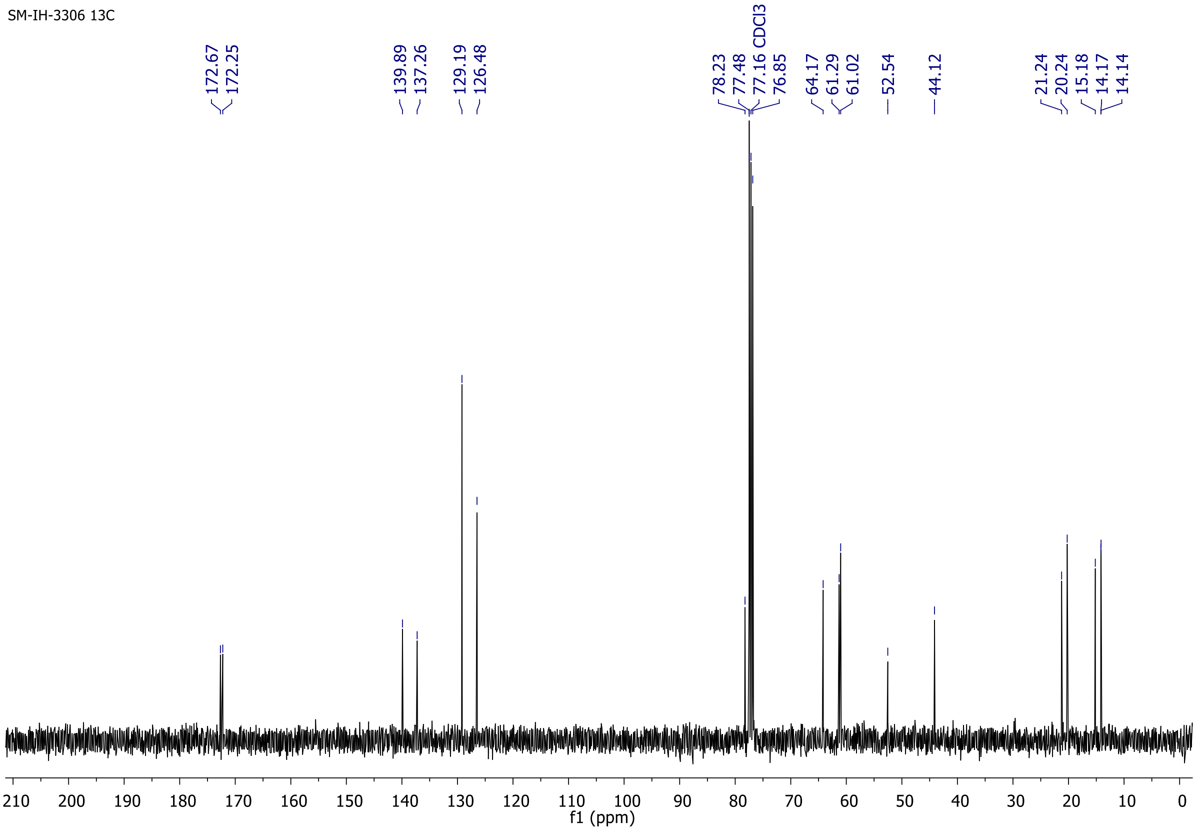

$^{1}H$ NMR of **26b** (400 MHz, $CDCl_3$):

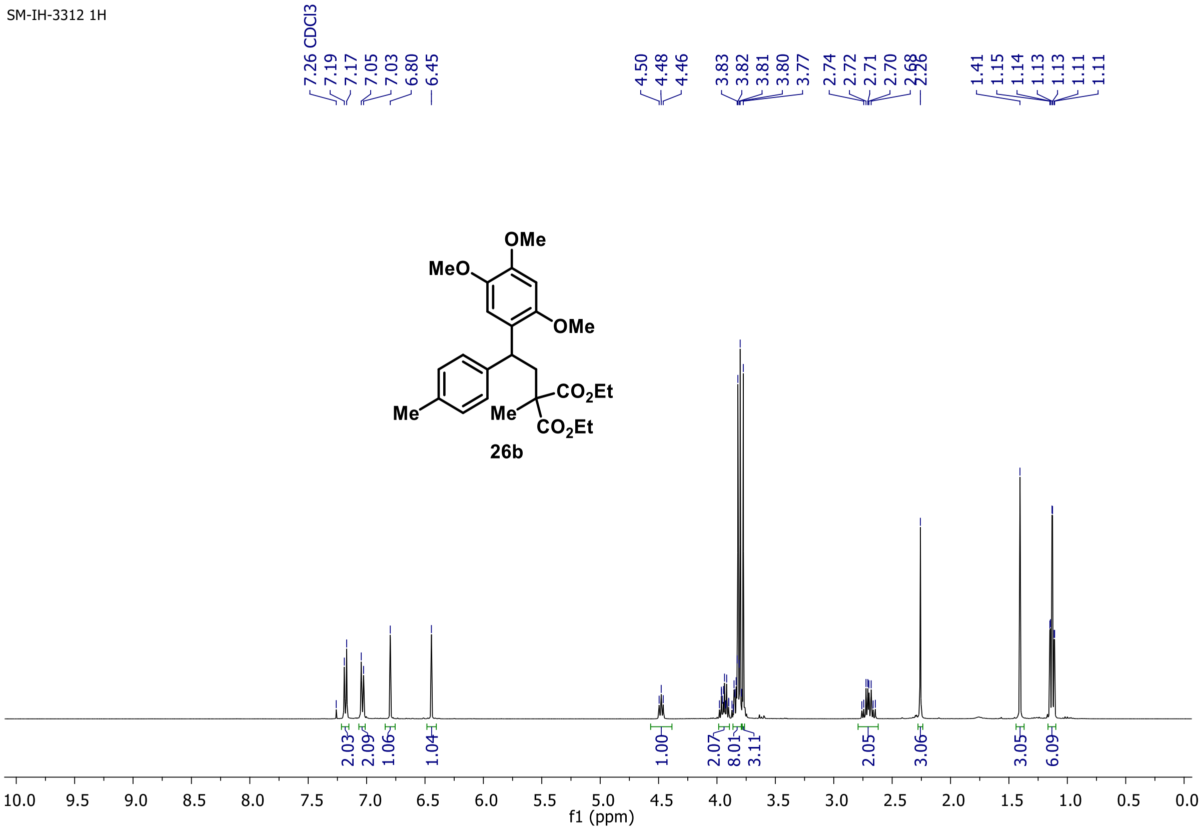


$^{13}C\{^{1}H\}$ NMR of **26b** (101 MHz, $CDCl_3$):

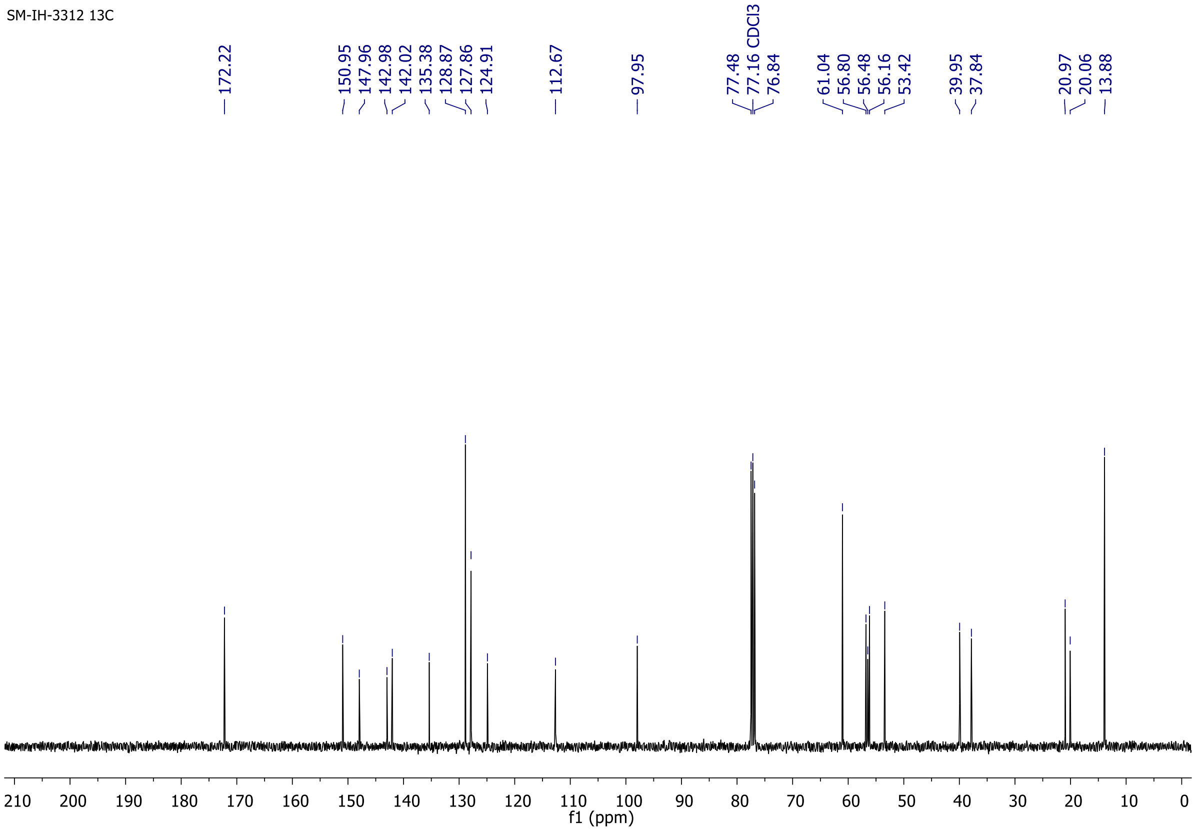

$^{1}$H NMR of **27** (400 MHz, $CDCl_3$):

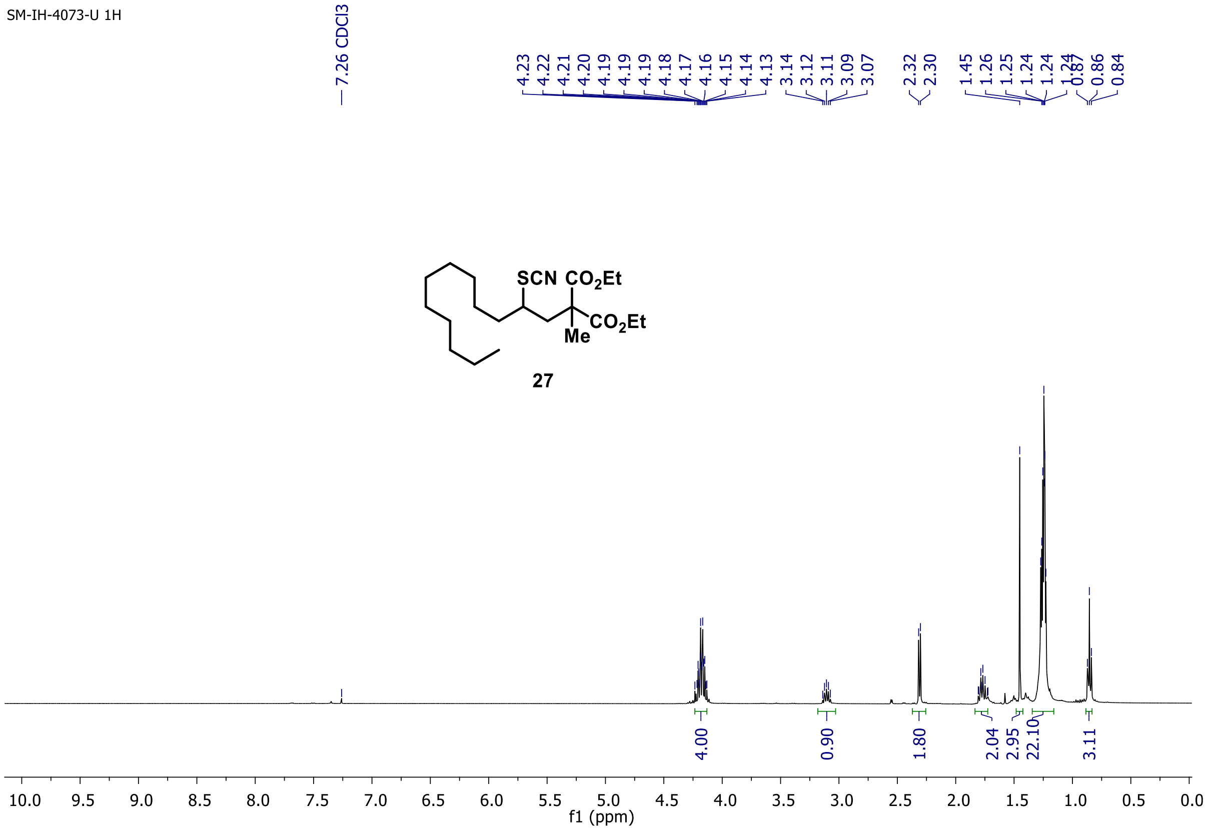


$^{13}C\{^{1}H\}$ NMR of **27** (101 MHz, $CDCl_3$):

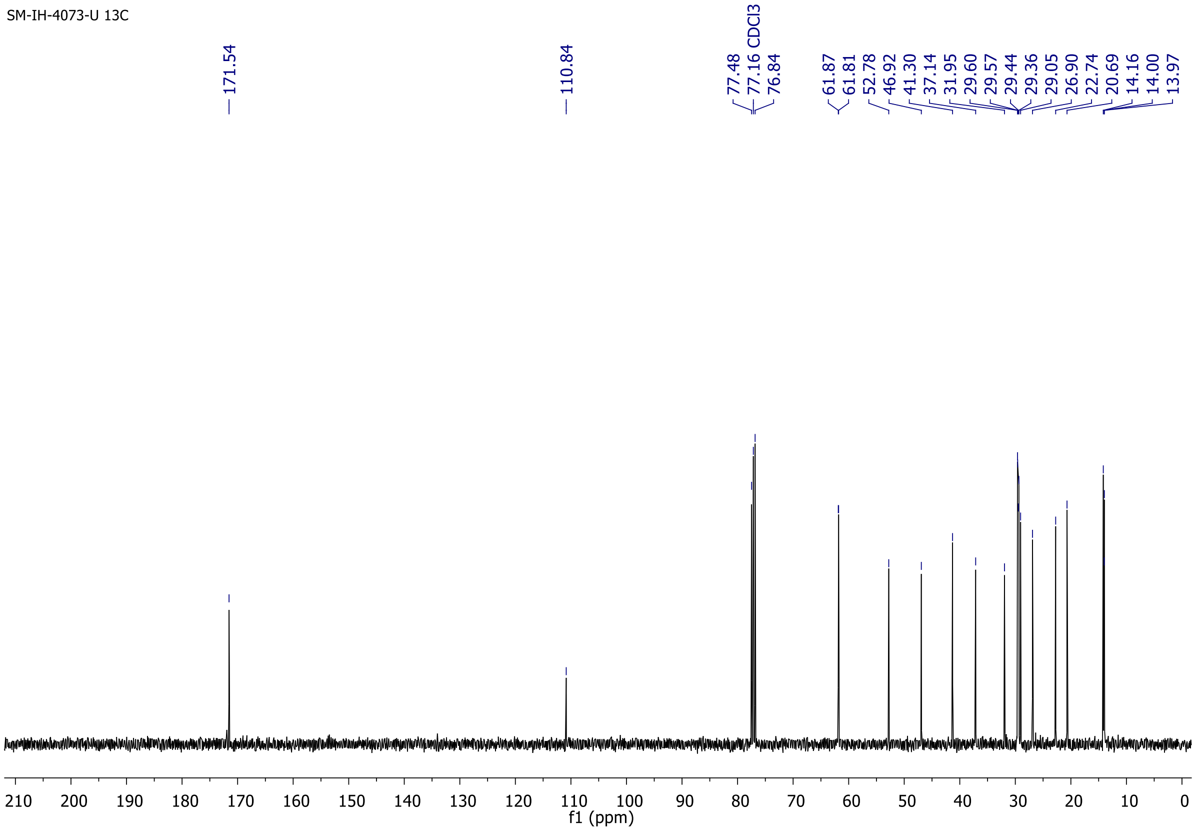

$^1$H NMR of **4s'** (400 MHz, $CDCl_3$):

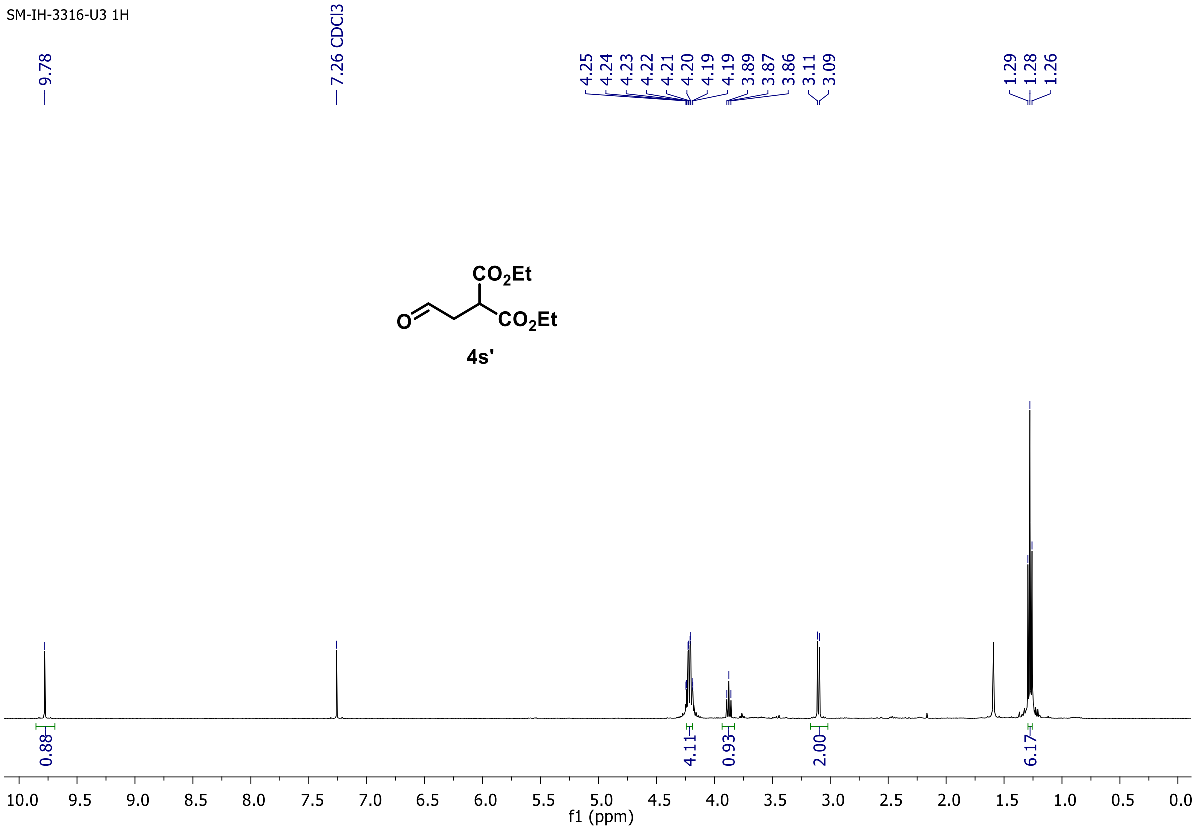


$^{13}C\{^1H\}$ NMR of **4s'** (101 MHz, $CDCl_3$):

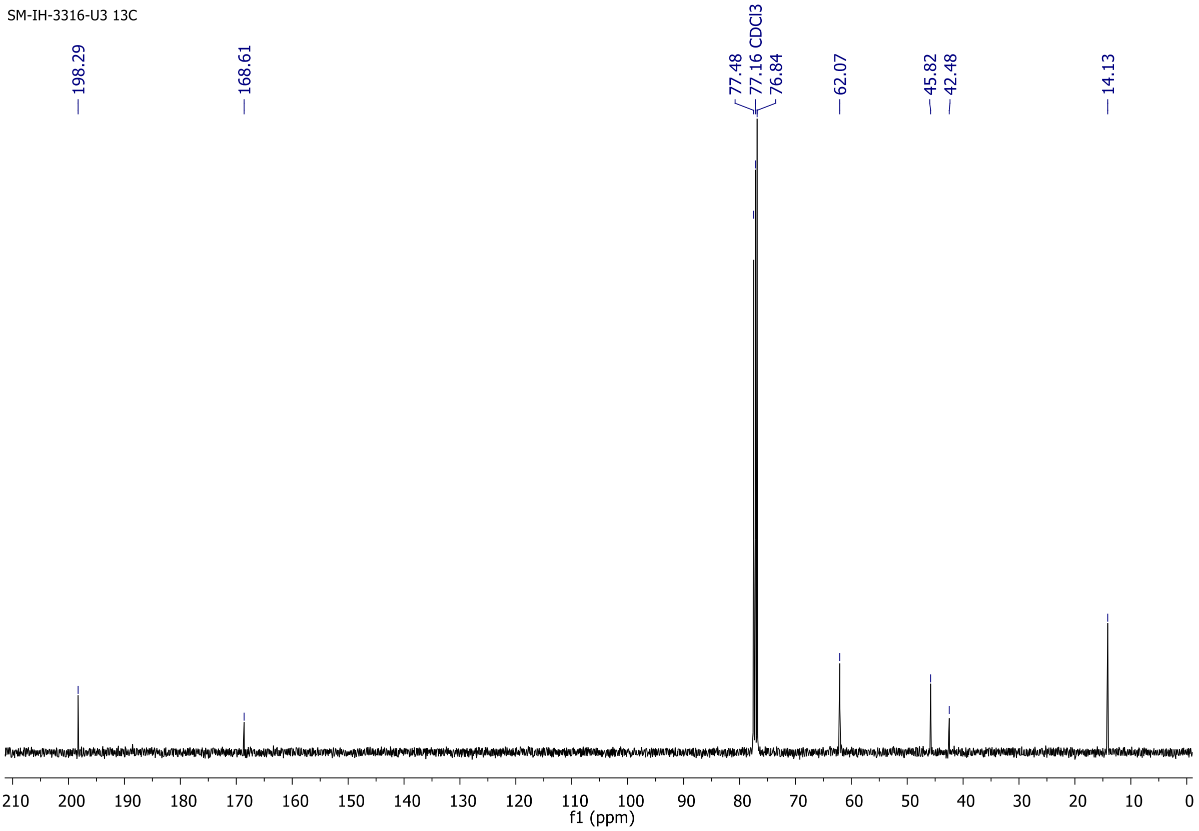

$^{1}$H NMR of **diethyl 2-phenylmalonate** (400 MHz, $CDCl_3$):

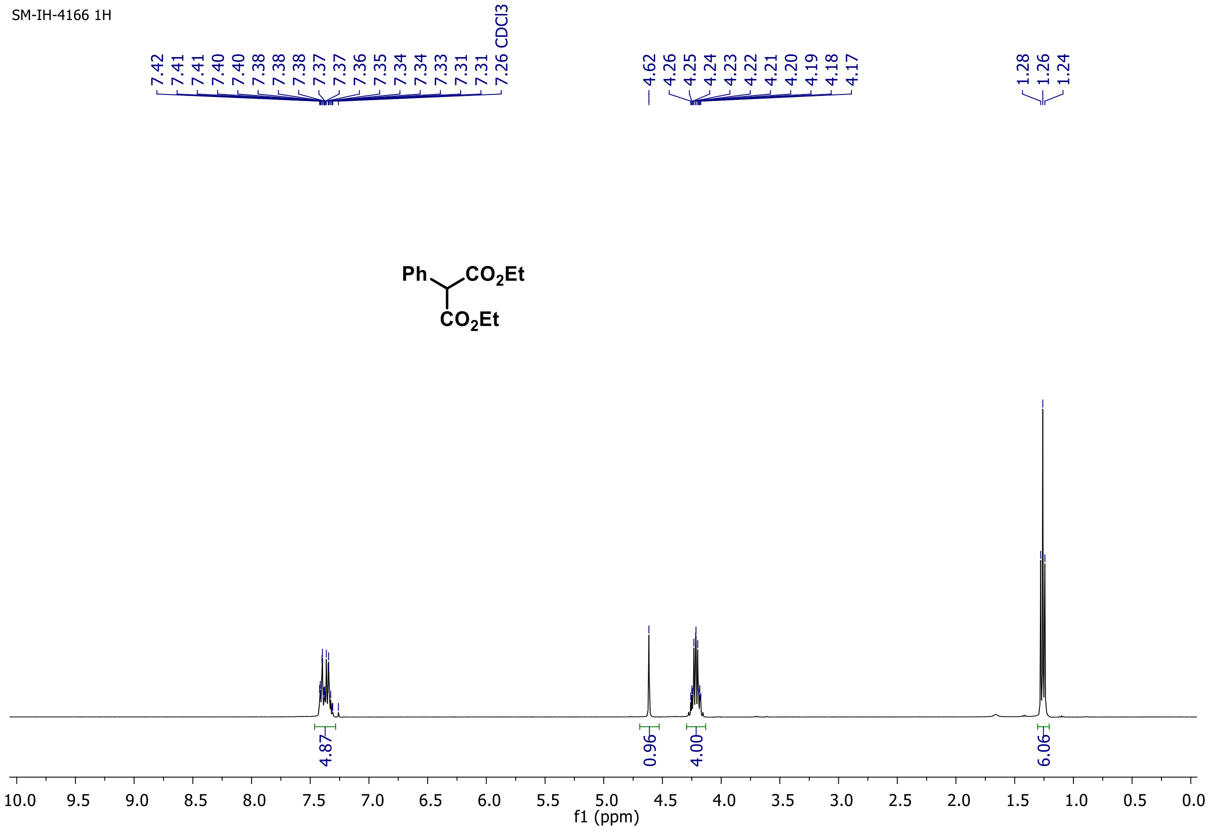


$^{13}$C{$^{1}$H} NMR of **diethyl 2-phenylmalonate** (101 MHz, $CDCl_3$):

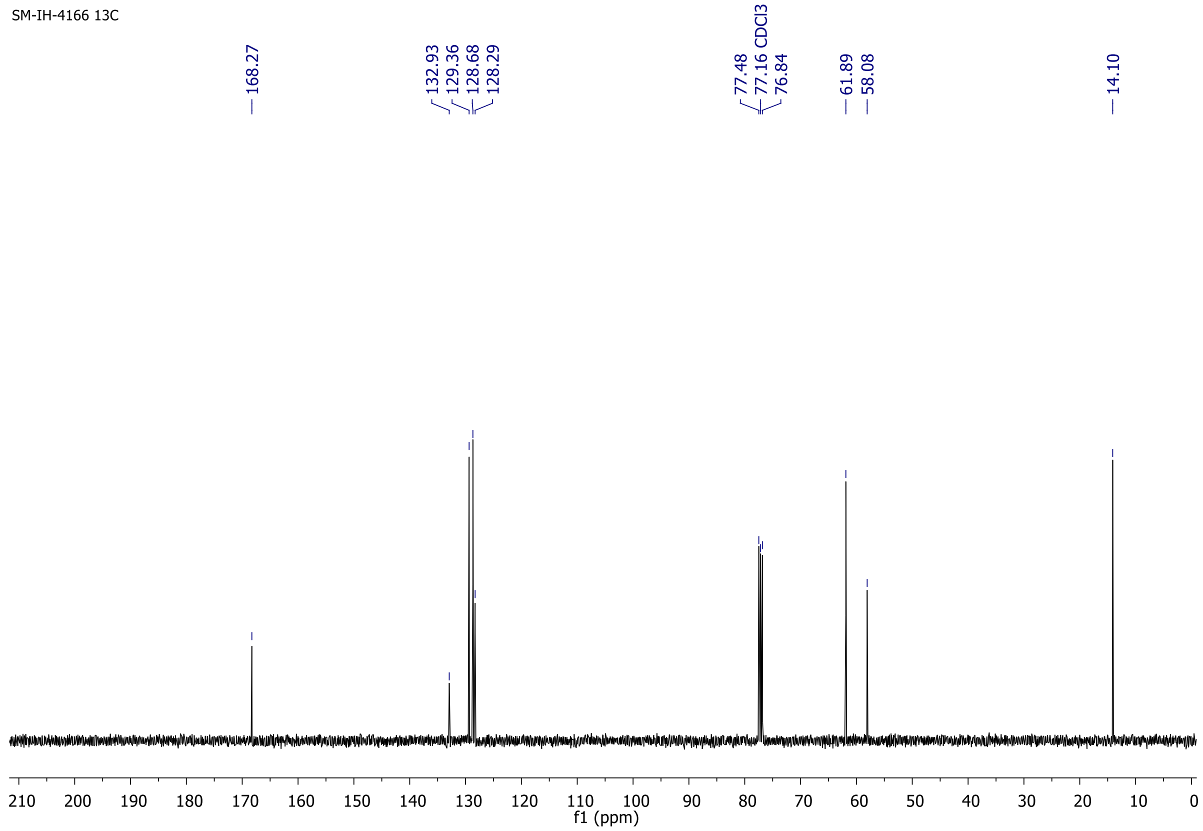

$^{1}H$ NMR of **(isothiocyanatomethylene)dibenzene** (400 MHz, $CDCl_3$):

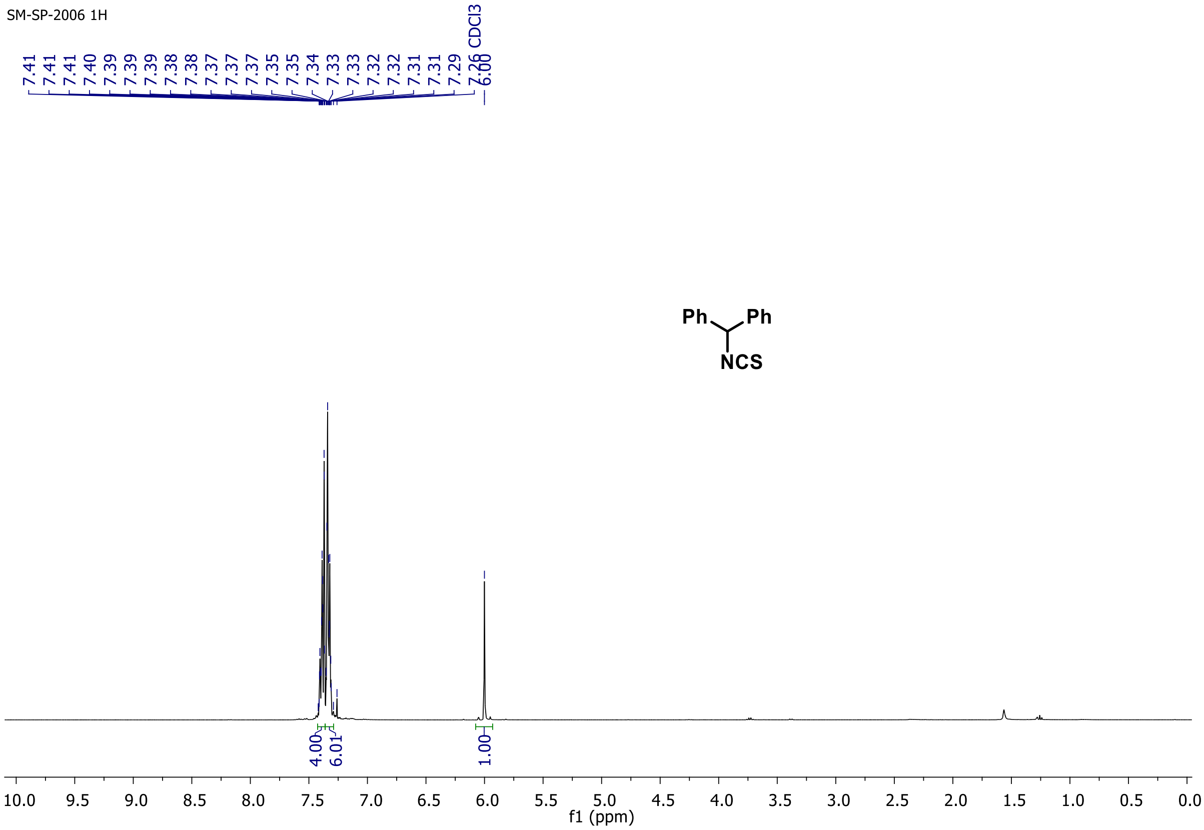


$^{13}C\{^{1}H\}$ NMR of **(isothiocyanatomethylene)dibenzene** (101 MHz, $CDCl_3$):

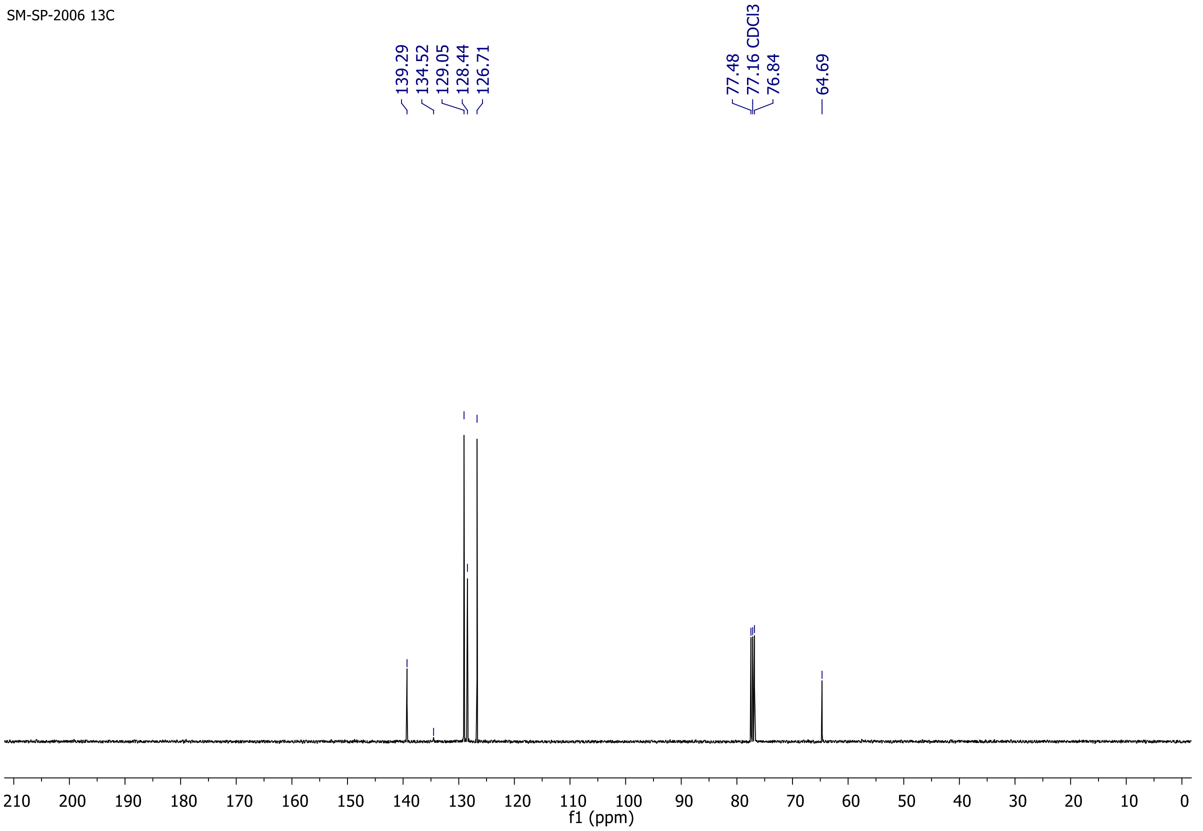

## 13. Supplementary References:


1 Armarego, W. L. F. *Purification of Laboratory Chemicals*. (Butterworth-Heinemann, Oxford, 2012).

2 Speck, F., Rombach, D. & Wagenknecht, H.-A. *N*-Arylphenothiazines as strong donors for photoredox catalysis – pushing the frontiers of nucleophilic addition of alcohols to alkenes. *Beilstein J. Org. Chem.* **15**, 52-59 (2019).

3 Shibutani, S., Nagao, K. & Ohmiya, H. Organophotoredox-catalyzed three-component coupling of heteroatom nucleophiles, alkenes, and aliphatic redox active esters. *Org. Lett.* **23**, 1798-1803 (2021).

4 Reddy, T. V. K., Devi, B. L. A. P., Prasad, R. B. N., Sujitha, P. & Kumar, C. G. Total synthesis and biological evaluation of clavaminol-G and its analogs. *Eur. J. Med. Chem.* **67**, 384-389 (2013).

5 Yazici, A. & Pyne, S. G. Sequential 1, 4- and 1, 2-addition reactions to α, β-unsaturated *N*-acyliminium ions: a new strategy for the synthesis of spiro and bridged heterocycles. *Org. Lett.* **15**, 5878-5881 (2013).

6 Siu, J. C., Parry, J. B. & Lin, S. Aminoxyl-catalyzed electrochemical diazidation of alkenes mediated by a metastable charge-transfer complex. *J. Am. Chem. Soc.* **141**, 2825-2831 (2019).

7 Amos, S. G. E., Nicolai, S. & Waser, J. Photocatalytic Umpolung of *N*-and *O*-substituted alkenes for the synthesis of 1, 2-amino alcohols and diols. *Chem. Sci.* **11**, 11274-11279 (2020).

8 Chen, Y., Ma, Y., Li, L., Jiang, H. & Li, Z. Nitration–Peroxidation of Alkenes: A Selective Approach to β-Peroxyl Nitroalkanes. *Org. Lett.* **21**, 1480-1483 (2019).

9 Matsubara, K. *et al.* Radical polymerization of styrene derivatives bearing *N*-free amino acid side chains, synergic effect of chirality, and hydrogen bonding for stereoselective polymerization. *J. Polym. Sci., Part A: Polym. Chem.* **48**, 5593-5602 (2010).

10 Yao, W. *et al.* Excited-state palladium-catalyzed radical migratory mizoroki–heck reaction enables C2-alkenylation of carbohydrates. *J. Am. Chem. Soc.* **144**, 3353-3359 (2022).

11 Fang, L.-Z., Shen, J.-M., Lv, Q.-H. & Yan, F.-l. Facile and Efficient Method for α-Monobromination of Dicarbonyl Compounds with *N*-Bromosuccinimide. *Asian J. Chem.* **23**, 3425 (2011).

12 Meshram, H. M., Thakur, P. B., Babu, B. M. & Bangade, V. M. A convenient, rapid, and general synthesis of α-oxo thiocyanates using clay supported ammonium thiocyanate. *Tetrahedron Lett.* **53**, 1780-1785 (2012).

13 Marx, L. B. & Burton, J. W. A total synthesis of Salinosporamide A. *Chem. –Eur. J.* **24**, 6747-6754 (2018).

14 Terent’ev, A. O. *et al.* Selective synthesis of α-thiocyanates from β-dicarbonyl compounds using cerium (IV) ammonium nitrate (CAN) and sodium thiocyanate. Striking difference in the thiocyanataion of malonates compared with β-diketones and β-oxo esters. *Mendeleev Commun.* **26**, 226-227 (2016).

15 Gomez Esteban, S., de la Cruz, P., Aljarilla, A., Arellano, L. M. & Langa, F. Panchromatic push–pull chromophores based on triphenylamine as donors for molecular solar cells. *Org. Lett.* **13**, 5362-5365 (2011).

16 Pirenne, V. *et al.* Eosin-mediated alkylsulfonyl cyanation of olefins. *Org. Lett.* **20**, 4521-4525 (2018).

17 Ando, T., Clark, J. H., Cork, D. G., Fujita, M. & Kimura, T. Inorganic-solid-supported potassium thiocyanate: study of reagent preparation and a convenient synthesis of tert-alkyl thiocyanates. *J. Org. Chem.* **52**, 681-685 (1987).

18 Al-Khalil, S. I., Bowman, W. R., Gaitonde, K., Marley, M. A. & Richardson, G. D. Radical-nucleophilic substitution ($S_{RN}1$) reactions. Part 7. Reactions of aliphatic α-substituted nitro compounds. *J. Chem. Soc., Perkin trans. 2*, 1557-1565 (2001).

19 Marpna, I. D., Wanniang, K., Lipon, T. M., Shangpliang, O. R. & Myrboh, B. Selenocyanation of aryl and styryl methyl ketones in the presence of selenium dioxide and malononitrile: an approach for the synthesis of α-carbonyl selenocyanates. *J. Org. Chem.* **86**, 1980-1986 (2020).

20 Wu, D., Duan, Y., Liang, K., Yin, H. & Chen, F.-X. AIBN-initiated direct thiocyanation of benzylic $sp^3$ C–H with *N*-thiocyanatosaccharin. *Chem. Commun.* **57**, 9938-9941 (2021).

21 Huang, S. *et al.* Organocatalytic asymmetric deoxygenation of sulfones to access chiral sulfinyl compounds. *Nature Chem.* **15**, 185-193 (2023).

22 Xie, M.-S. *et al.* Thiourea participation in [3+2] cycloaddition with donor–acceptor cyclopropanes: a domino process to 2-amino-dihydrothiophenes. *Chem. Commun.* **55**, 1580-1583 (2019).

23 Cao, X. T. *et al.* $H_2O_2$-Mediated Synthesis of 1,2,4-Thiadiazole Derivatives in Ethanol at Room Temperature. *Adv. Synth. Catal.* **364**, 689-694 (2022).

24 Shan, Q.-C., Hu, L.-M., Qin, W. & Hu, X.-H. Copper-Catalyzed Cross-Nucleophile Coupling of β-Allenyl Silanes with Tertiary C–H Bonds: A Radical Approach to Branched 1, 3-Dienes. *Org. Lett.* **23**, 6041-6045 (2021).

25 Mitra, S., Ghosh, M., Mishra, S. & Hajra, A. Metal-free thiocyanation of imidazoheterocycles through visible light photoredox catalysis. *J. Org. Chem.* **80**, 8275-8281 (2015).

26 López, F., Castedo, L. & Mascareñas, J. L. A Sulfinyl-Directed Asymmetric [5C + 2C] Intramolecular Acetoxypyranone−Alkene Cycloaddition. *Org. Lett.* **4**, 3683-3685 (2002).

27 Shibutani, S. *et al.* Organophotoredox-catalyzed decarboxylative C($sp^3$)–O bond formation. *J. Am. Chem. Soc.* **142**, 1211-1216 (2020).

28 Treat, N. J. *et al.* Metal-free atom transfer radical polymerization. *J. Am. Chem. Soc.* **136**, 16096-16101 (2014).

29 Bellotti, P. *et al.* Visible-Light Photocatalyzed peri-(3+2) Cycloadditions of Quinolines. *J. Am. Chem. Soc.* **144**, 15662-15671 (2022).

30 Speckmeier, E., Fischer, T. G. & Zeitler, K. A toolbox approach to construct broadly applicable metal-free catalysts for photoredox chemistry: deliberate tuning of redox potentials and importance of halogens in donor–acceptor cyanoarenes. *J. Am. Chem. Soc.* **140**, 15353-15365 (2018).

31 Sakakibara, Y. & Murakami, K. Switchable divergent synthesis using photocatalysis. *ACS Catal.* **12**, 1857-1878 (2022).

32 Cismesia, M. A. & Yoon, T. P. Characterizing chain processes in visible light photoredox catalysis. *Chem. Sci.* **6**, 5426-5434 (2015).

33 Hatchard, C. G. & Parker, C. A. A new sensitive chemical actinometer-II. Potassium ferrioxalate as a standard chemical actinometer. *Proc. R. Soc. A: Math. Phys. Eng.* **235**, 518-536 (1956).

34 Tang, X. J. & Dolbier Jr, W. R. Efficient Cu-catalyzed atom transfer radical addition reactions of fluoroalkylsulfonyl chlorides with electron-deficient alkenes induced by visible light. *Angew. Chem. Int. Ed.* **54**, 4246-4249 (2015).

35 Wayner, D. D. M., McPhee, D. J. & Griller, D. Oxidation and reduction potentials of transient free radicals. *J. Am. Chem. Soc.* **110**, 132-137 (1988).

36 Oukoloff, K. *et al.* Evaluation of the Structure–Activity Relationship of Microtubule-Targeting 1,2,4-Triazolo [1,5-*a*] Pyrimidines Identifies New Candidates for Neurodegenerative Tauopathies. *J. Med. Chem.* **64**, 1073-1102 (2021).

37 Fu, Z., Yuan, W., Chen, N., Yang, Z. & Xu, J. $Na_2S_2O_8$-mediated efficient synthesis of isothiocyanates from primary amines in water. *Green Chem.* **20**, 4484-4491 (2018).

38 Lee, C., Yang, W. & Parr, R. G. Development of the Colle-Salvetti correlation-energy formula into a functional of the electron density. *Phys. Rev. B* **37**, 785 (1988).

39 Becke, A. D. Density-functional thermochemistry. III. The role of exact exchange. *J. Chem. Phys.* **98**, 5648-5652 (1993).

40 Grimme, S. Accurate description of van der Waals complexes by density functional theory including empirical corrections. *J. Comput. Chem.* **25**, 1463-1473 (2004).

41 Grimme, S., Antony, J., Ehrlich, S. & Krieg, H. A consistent and accurate ab initio parametrization of density functional dispersion correction (DFT-D) for the 94 elements H-Pu. *J. Chem. Phys.* **132**, 154104 (2010).

42 Grimme, S. Density functional theory with London dispersion corrections. *Wiley Interdiscip. Rev. Comput. Mol. Sci.* **1**, 211-228 (2011).

43 Ehrlich, S., Moellmann, J. & Grimme, S. Dispersion-corrected density functional theory for aromatic interactions in complex systems. *Acc. Chem. Res.* **46**, 916-926 (2013).

44 Weigend, F. & Ahlrichs, R. Balanced basis sets of split valence, triple zeta valence and quadruple zeta valence quality for H to Rn: Design and assessment of accuracy. *Phys. Chem. Chem. Phys.* **7**, 3297-3305 (2005).

45 Weigend, F. Accurate Coulomb-fitting basis sets for H to Rn. *Phys. Chem. Chem. Phys.* **8**, 1057-1065 (2006).

46 Marenich, A. V., Cramer, C. J. & Truhlar, D. G. Universal solvation model based on solute electron density and on a continuum model of the solvent defined by the bulk dielectric constant and atomic surface tensions. *J. Phys. Chem. B* **113**, 6378-6396 (2009).

47 Frisch, M. J. E. *et al.* Gaussian 16, Revision A.03. (Gaussian, Inc., Wallingford CT, 2016).
