# Supplementary material for: Photocascade chemoselective controlling of ambident thio(seleno)cyanates with alkenes via catalyst modulation
Source: Nat Commun. 2024 Jul 9;15:5739. doi: 10.1038/s41467-024-49279-w (PMC11233607; doi:10.1038/s41467-024-49279-w)
Supplement: Supplementary file 3 — Description of Additional Supplementary Files [file 41467_2024_49279_MOESM3_ESM.docx]

**Supplementary data legends**

- Supplementary Information
- Supplementary Data 1 - Cartesian Coordinates of Optimized Geometry (excel file)
